# Conserved Residues Adjacent to ß-Barrel and Loop Intersection among Enterovirus VP1 Affect Viral Replication: Potential Target for Anti-Enteroviral Development

**DOI:** 10.3390/v14020364

**Published:** 2022-02-10

**Authors:** Ya-Ling Huang, Sheng-Wen Huang, Chun-Yu Shen, Dayna Cheng, Jen-Ren Wang

**Affiliations:** 1 Institute of Basic Medical Sciences, College of Medicine, National Cheng Kung University, Tainan 701, Taiwan; ed105471@edah.org.tw (Y.-L.H.); daynac_1201@hotmail.com (D.C.); 2 Department of Laboratory Medicine, E-Da Hospital, Kaohsiung 824, Taiwan; 3 Department of Medical Laboratory Science, I-Shou University, Kaohsiung 824, Taiwan; 4 National Mosquito-Borne Diseases Control Research Center, National Health Research Institutes, Tainan 701, Taiwan; joehuang@nhri.edu.tw; 5 Department of Medical Laboratory Science and Biotechnology, College of Medicine, National Cheng Kung University, Tainan 701, Taiwan; jacky3484382@gmail.com; 6 National Institute of Infectious Diseases and Vaccinology, National Health Research Institutes, Tainan 701, Taiwan; 7 Center of Infectious Disease and Signaling Research, National Cheng Kung University, Tainan 701, Taiwan

**Keywords:** *Enterovirus*, VP1, conserved residues, replication, antiviral

## Abstract

*Enterovirus* genus has over one hundred genotypes and could cause several kinds of severe animal and human diseases. Understanding the role of conserved residues in the VP1 capsid protein among the *enterovirus* genus may lead to anti-enteroviral drug development. The highly conserved residues were found to be located at the loop and ß-barrel intersections. To elucidate the role of these VP1 residues among the *enterovirus* genus, alanine substitution reverse genetics (rg) variants were generated, and virus properties were investigated for their impact. Six highly conserved residues were identified as located near the inside of the canyon, and four of them were close to the ß-barrel and loop intersection. The variants rgVP1-R86A, rgVP1-P193A, rgVP1-G231A, and rgVP1-K256A were unable to be obtained, which may be due to disruption in the virus replication process. In contrast, rgVP1-E134A and rgVP1-P157A replicated well and rgVP1-P157A showed smaller plaque size, lower viral growth kinetics, and thermal instability at 39.5°C when compared to the rg wild type virus. These findings showed that the conserved residues located at the ß-barrel and loop junction play roles in modulating viral replication, which may provide a pivotal role for pan-enteroviral inhibitor candidate.

## 1. Introduction

The *Enterovirus* genus is comprised of fifteen species of small, non-enveloped, icosahedral RNA viruses, whereby seven of them are important human pathogens [1]. In the past decades, many newly identified enteroviruses have been shown to be widely present in the environment and in various animals [2]. Enterovirus A71 (EV-A71) is considered the most pathogenic enterovirus in humans since it is highly associated with severe diseases including poliomyelitis-like paralysis, brainstem encephalitis, fatal cardiorespiratory failure, pulmonary edema, and even death [3]. Many types of drugs have been available in clinical trials, but the potency of the treatment was demonstrated to be ineffective or have side effects [4]. There are no effective drugs or vaccines against different types of enteroviruses currently [5]. Therefore, the development of a drug against all members of the *Enterovirus* genus is an unmet need.

Enteroviruses consist of four capsid proteins including VP1 to VP4. VP1, VP2, and VP3 are surface-exposed proteins and VP4 is an internal protein. The main focus of this report is VP1, the major capsid protein on the surface of enteroviruses. Once cellular receptors interact with the VP1 protein, VP1 undergoes a conformational change, leading to the disassembly of the viral particle and RNA release [6]. VP1 is one of the potential targets for enterovirus treatment. It possesses a “jelly-roll” topology of eight-stranded antiparallel ß-barrels (B to I) and its loops are named using two letters designating the ß strands that the loop connects to [7]. Combining the secondary structure and the degree of conservation, conserved residues were found in the canyon and interior areas of the viral particle, and most of the residues interacted with other capsid proteins (unpublished data). Previously in mouse-adapted EV-A71, VP1-145 was known as an important virulence residue [8,9]. Huang et. al. reported that VP1-Q145E enhances the binding of EV-A71 to mouse neuroblastoma. Despite many virulence residues being studied on VP1, the critical function during infection still remains unclear [10].

Due to environmental pressures, many RNA viruses have high spike diversity on the surface of viral particles. A broad-spectrum drug or vaccine development for RNA viruses is limited. For example, the influenza virus has available vaccines, however, the vaccines are targeted toward the most prevalent strains and thus require an annual update on their composition. A large number of studies focused on the highly conserved stem region of hemagglutinin. Targeting this conserved region could neutralize a broad range of all influenza viruses [11]. Another example is the E2 protein, which is found on the surface of hepatitis C virus (HCV) particles. Through structural models of E2 from different strains of HCV, computational simulation discovered that the highly conserved and flexible regions may be new vaccine candidates that could generate broad neutralizing antibodies [12]. Recently, with the SARS-CoV-2 pandemic, the virus’ ability to mutate and adapt is faster than drug or vaccine development [13]. Therefore, the development of broad-spectrum drug targeting conserved regions for long-term treatment strategies is an unmet need.

The high diversity of VP1 is the main obstacle for broad spectrum enteroviral drug development. Using a structure-based drug discovery approach, we can screen the potential small molecular compounds to target specific structures. To understand the roles of highly conserved residues, which may provide broad protection from infections and diseases, our study here began with the alignment of the *Enterovirus* genus conserved sequences. Following the exploration of the conserved residue distribution of VP1, four conserved residues found in the ß-barrel and loop junction were chosen as candidates for this study. A reverse genetics (rg) system was used to generate the rg variants of the selected conserved residues. Furthermore, the viral properties of the rg variants including growth rate, plaque size, temperature sensitivity, thermostability, and binding ability were investigated.

## 2. Materials and Methods

### 2.1. Alignment of GenBank Sequences

A total of 1632 VP1 of enterovirus sequences were download from Genbank. The sequences included 368 EV-A71, 1098 Coxsackievirus A16, 32 EV-D68, 2 Coxsackievirus A19, 2 Coxsackievirus A4, 1 Enterovirus C113, 1 Enterovirus G, and other enteroviruses. The sequences’ search keywords were [VP1, and Enterovirus]. The sequence length range was set from 296 to 297 to represent the full length VP1 sequences. The revision date was set from 1 January 2020 to 31 December 2021 to obtain state-of-art data. A *.fasta file was downloaded from GenBank. The multiple sequence alignment was analyzed by CLUSTALW (https://www.genome.jp/tools-bin/clustalw, accessed on 12 December 2021). The *.aln file was generated from CLUSTALW. The Easy Sequencing in Postscript 3.0 (ESPript 3.0) was applied to enhance the alignment and structure information graphically [14]. The output file was generated after submission. The PDF output file was saved for analysis (Supplementary File S1).

### 2.2. Predicting Consensus Sequences and Protein Structure Modeling

Conserved amino acid sequences of the *Enterovirus* genus were applied by Consurf as previously described [15,16]. A consensus sequence was obtained by WebLogo to generate a sequence logo (http://weblogo.threeplusone.com/, accessed on 10 June 2018) [17]. The dynamics of the protein structures were predicted using the CABS-flex 2.0 web server interface for simulations of protein structure fluctuations (http://biocomp.chem.uw.edu.pl/CABSflex2, accessed on 23 September 2021) [18]. The EV-A71 VP1 capsid protein structures (PDB code 3VBS) were viewed using the UCSF Chimera program, version 1.9 [19,20]. Potential interactions between the indicated amino acid residues were identified among neighboring atoms of the residues. All of the bioinformatics tool analysis conditions were set as the default.

### 2.3. Preparation of Reverse Genetics VP1 Variants 

EV-A71-4643-y12, generated from a C2 strain (rgVP1 WT) isolated in the 1998 outbreak from a child with fatal encephalitis in Taiwan, was used as the backbone [21]. The alanine substitutions were produced by overlapping PCR and site-directed mutagenesis as previously described [9], using the primers listed in Supplementary Table S1. Constructs were confirmed by Sanger sequencing. Following linearization with BbVCI and BspEI, viral RNA was in vitro transcribed using T7 polymerase (RiboMAX, Promega, Madison, WI, USA) and transfected into RD cells using the TransMessenger Transfection Reagent (Qiagen, Hilden, Germany). At 0, 2, 4, 6, 8, 24, 48, and 72 h after transfection, rgVP1 viruses were harvested and analyzed.

### 2.4. Plaque Forming Assay

Human rhabdomyosarcoma cells (RD cells, ATCC CCL-136) were cultured in Eagle’s Minimum Essential Medium (EMEM) supplemented with 10% fetal bovine serum and 1% penicillin-streptomycin solution at 35 °C, 5% CO_2_. Plaque forming assay was performed on RD cells as previously described [22]. Briefly, confluent RD cells were incubated at 35 °C with a ten-fold serially diluted virus for 1 h. After removing the medium, the infected cells were covered with 0.3% agarose for three to five days at 35 °C until the plaques could be visualized under the microscope. Culture plates were fixed with 4% paraformaldehyde and stained with crystal violet. The numbers and diameters of the plaques were measured with Image J2 software [23]. The titer of virus stocks was also determined using a cell culture infectious dose 50% (CCID_50_) assay by the Reed and Muench method [24].

### 2.5. Immunofluorescence Stain of rgVP1 Variants

Confluent RD cells cultured in 24-well plates with 10 mm^2^ coverslips were transfected with the RNA derived from infectious clones using the TransMessenger Transfection Reagent (Qiagen). After three days post-transfection, the coverslips were fixed with cold acetone for 10 min, then incubated with the anti-EV-A71 mab979 antibody (1:1000-fold diluted, Sigma-Aldrich, St. Louis, MO, USA) at 37 °C for 1 h. Afterward, a 1:1000 of Alexa-488-conjugated goat anti-mouse IgG (Jackson Immuno Research Laboratories, Inc., West Grove, PA, USA) was added and incubated for 1 h at 37 °C. The fluorescent cell numbers were counted by fluorescence microscope under 200× magnification, and an average of 25 fields per slide were analyzed.

### 2.6. Real-Time RT-PCR

The quantitative analysis of viral RNA replication was carried out by real-time TaqMan RT-PCR as previously described using the AgPath-ID™ One-Step RT-PCR Reagents with modification (Applied Biosystems™, Waltham, MA, USA) [25]. The forward 5′-GAG AGT TCT ATA GGG GAC AGT-3′ and reverse 5′-AGC TGT GCT ATG TGA ATT AGG AA-3′ primers were used. A TaqMan probe with 5′-FAM-ACT TAC CCA GGC CCT GCC AGC TCC- TAMRA-3′ quencher was used.

### 2.7. One-Step Growth Curves and Temperature Sensitivity Assay

The one-step growth curves for the rg wild type and variants were performed as previously described with a multiplicity of infection (MOI) of 10 and 0.1 [23]. RD cells were cultured in 12-well plates (1 × 10^5^ cells/well) and infected with the viruses at 35 °C or 39.5 °C. Virus titers were determined by the plaque forming assay after incubation at 1, 2, 3, and 4 days post-infection. Temperature sensitivity was expressed as the difference of Log_10_ PFU values at 35 °C and 39.5 °C (ΔPFU). Temperature resistant (tr) and temperature-sensitive (ts) phenotypes were defined as logarithmic difference <2.0, and >2.0, respectively [26]. 

### 2.8. Thermostability

Viruses of 10,000 PFU/0.1 mL were aliquoted per tube in 1.5 mL microtubes, and then heated in a water bath at 39.5 °C for 0, 0.5, 1.0, 1.5, or 2.0 h. The tube treated immediately (0 h) served as the control. Plaque forming assays were performed for virus titration.

### 2.9. Binding Assay

RD cells were seeded in 96-well plates (1 × 10^5^ cells/well) and incubated at 35 °C for 48 h. Cells were fixed with 4% paraformaldehyde and viruses of 1000 and 10,000 PFU per 100 μL were added into the cells and incubated at 4 °C overnight. Wells containing medium only and wild type served as the controls. After incubation with the anti-EV-A71 mab979 antibody (1:1000, Sigma-Aldrich) for 1 h, alkaline phosphatase conjugated anti-mouse IgG (1:1000, Thermo Fisher Scientific, Waltham, MA, USA) was then added and incubated at room temperature for 1 h. After washing, p-nitrophenyl phosphate was added and incubated at room temperature for 10 min. The reactions were quenched by adding 3.0 N NaOH and the absorbance at 405 nm was measured by an ELISA plate reader.

### 2.10. Statistics 

All statistical analysis was performed using the t-test. A *p*-value of <0.05 was considered statistically significant. Data are presented as the mean and standard deviation (mean ± SD). The data were analyzed by the program GraphPad Prism 5.0 (San Diego, CA, USA).

## 3. Results

### 3.1. Identification of Critical Highly Conserved Residues

**Figure 1:**
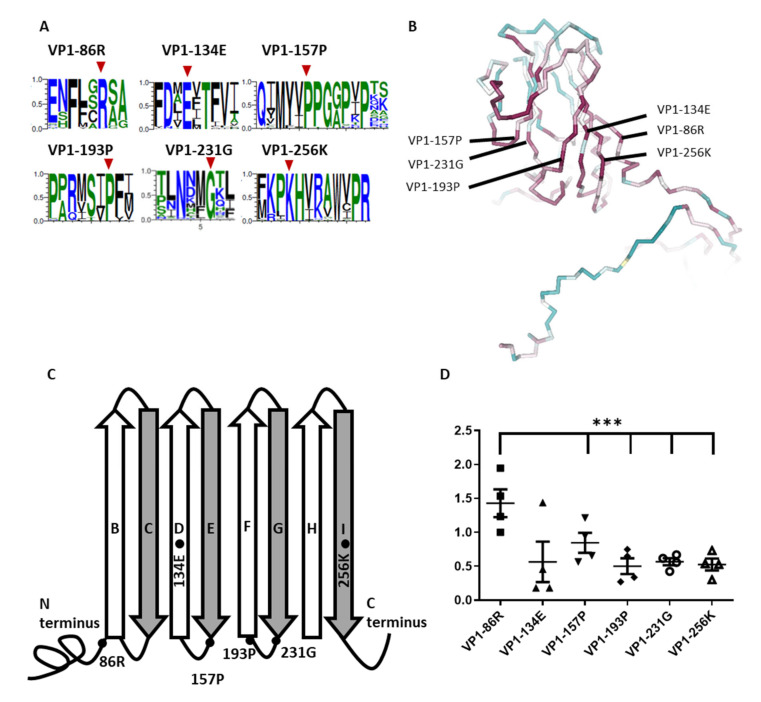
Diagram of highly conserved residues of VP1 among the *Enterovirus* genus. (**A**) After retrieving 90 reference sequences of enterovirus for alignment, amino acid conservation was demonstrated with WebLogo. Six conserved residues of this study are denoted with red arrowheads. (**B**) Consurf server with amino acid (PDB code 4AED) is shown in a relative steric position of the VP1 capsid protein structure of EV-A71. The six conserved residues are indicated. Highly conserved residues are shown in red and variable residues are shown in blue. (**C**) The representative cartoon diagram indicates the relative locations of the six conserved residues on VP1. (**D**) CABS-flex server was used to predict the protein backbone fluctuation profile among these conserved residues. Flexibility simulations in the VP1 protein structure of enteroviruses were produced. The 3-dimensional (3D) structures of the VP1 protein of four enterovirus strains (three EV-A71 and one echovirus 11) were generated using X-ray crystallography (PDB code 4AED, 4CDQ, 3VBS, and 1H8T). The fluctuation angles of the individual six residues are shown (***, *p* < 0.005).

To investigate the role of the conserved residues of VP1, we analyzed a total of 1632 protein sequences in VP1 of Enteroviruses from GenBank. Consistent with the Consurf server was used as per our previous unpublished data. A total of 28 residues was identified in EV-A71 VP1 (PDB code 4AED). To narrow down the study candidates of VP1 conserved residues, we further referred to the study of Wang et al. [27]. We then focused on the conserved residues that were located at the β-barrel and loop intersection as our study candidates. Six conserved residues where amino acids were over 99% identity among enteroviruses, VP1-86R, VP1-134E, VP1-157P, VP1-193P, VP1-231G, and VP1-256K, were used. To further visualize the six conserved residues, these designated sequences were confirmed by WebLogo (Figure 1A). To define the potential structure effect of the selected conserved residues in the VP1, the secondary structure was visualized by using the backbone style with NGL viewer [15,16]. Interestingly, all six conserved residues were close to each other and were located in the interior of the canyon (Figure 1B). According to the structure-based sequence alignment of VP1 of EV-A71 particles, six residues were shown in the cartoon representation, of which four residues (VP1-86R, VP1-157P, VP1-193P, and VP1-231G) were located at the β-barrel and loop intersection, except for VP1-134E and VP1-256K, which were located at ß-barrels D and I, respectively (Figure 1C). Conserved residues that are close to the ß-barrel may be associated with maintaining conformation stability and may play roles in modulating certain virus properties. According to our unpublished study, conserved residues and ß-barrel areas have low protein flexibility. To further examine the fluctuation situation of the six residues, we evaluated the fluctuation angle of individual residues in four VP1 capsid proteins of enterovirus (PDB code 4AED, 3VBS, 4CDQ, and 1H8T). The result demonstrated a low fluctuation angle (mean 0.61 ± 0.32 Å) in five conserved residues, which were located at the ß-barrel and at the junction. However, only VP1-86R located in the N-terminus and at the junction of ß-barrel B demonstrated a higher fluctuation angle (mean 1.43 ± 0.41 Å) (Figure 1D). According to our findings, conserved residues among the *Enterovirus* genus were located at the β-barrel and the junction, surrounding the inside of the canyon areas, which demonstrated low flexibility. 

### 3.2. Conserved Residues Adjacent to β-Barrel and Loop Intersection Affect Viral Replication

**Figure 2:**
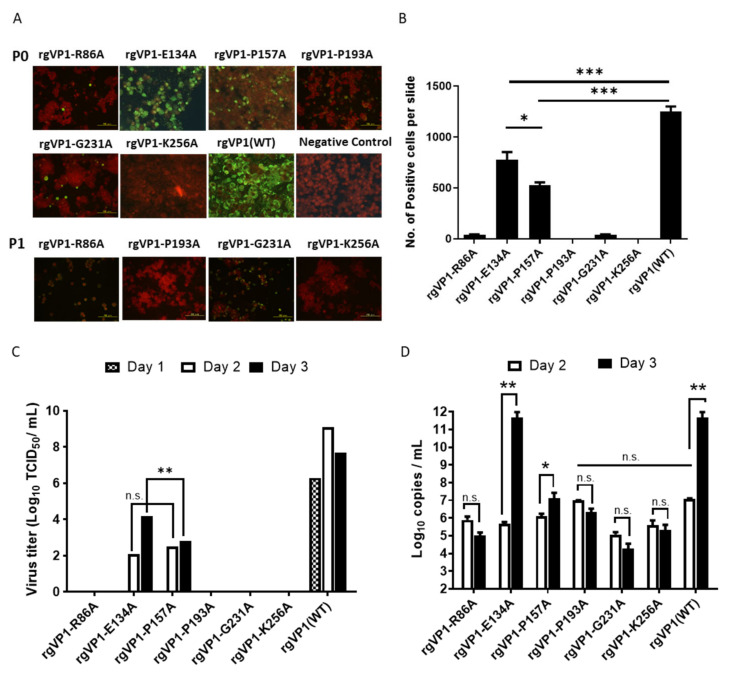
Analysis of alanine substitution of reverse genetics enterovirus. (**A**) Immunofluorescence stain of rgVP1 variants. RNA transcript was transfected into RD cells, the viral capsid protein expression of EV-A71 was detected by mab979 monoclonal antibody three days post-transfection. The green/red fluorescence indicating viral protein expression and Evan’s blues counter stain were examined under fluorescence microscope at a 200× magnification. (**B**) A total number of 25 fields per slide was used to analyze the number of positive green fluorescent cells under a fluorescence microscope. (**C**) Viral replication titers after RNA transcript transfection at days 1, 2, and 3, viral particles in the medium containing reverse genetics variants were measured by CCID_50_. (**D**) Viral RNA expression after RNA transcript transfection at days 2 and 3. The generative viral RNA from the medium was measured by real-time RT-PCR assay. The data were expressed as the mean ± SD of each group (n = 3). n.s., no significance, *, *p* < 0.05, **, *p* < 0.01, and ***, *p* < 0.005.

To investigate the role of the conserved residues of VP1 close to the β-barrel and loop intersection during infection, we used alanine substitution mutagenesis from the EV-A71 infectious clone. The primer sequences are listed in Table S1. Reverse genetics EV-A71 VP1 variants of the six identified conserved residues were generated by RNA transcript transfection. After transfection, the cytopathic effect (CPE) in the following days was observed. Obvious CPE was presented in both rgVP1-E134A and rgVP1-P157A at two to three days post-transfection. No obvious CPE was seen in rgVP1-R86A, rgVP1-P193A, rgVP1-G231A, and rgVP1-K256A at five days post-transfection. Additionally, no CPE was observed after passaging five times. The infected cells were confirmed by immunofluorescence (IF) staining, as shown in Figure 2A,B. As a result, no CPE and 0–1 positive fluorescence stains were observed in rgVP1-P193A and rgVP1-K256A. Only rgVP1-R86A and rgVP1-G231A presented weak fluorescence signals after one passage, but no signal in later passages (P2-P5). Through CPE and IF observations, the results suggest that the conserved residues located at the β-barrel and loop intersection affect viral replication (Table 1).

### 3.3. Conserved Residues Adjacent to β-Barrel and Loop Intersection Affect RNA Level and Viral Infectious Titer

**Figure 3:**
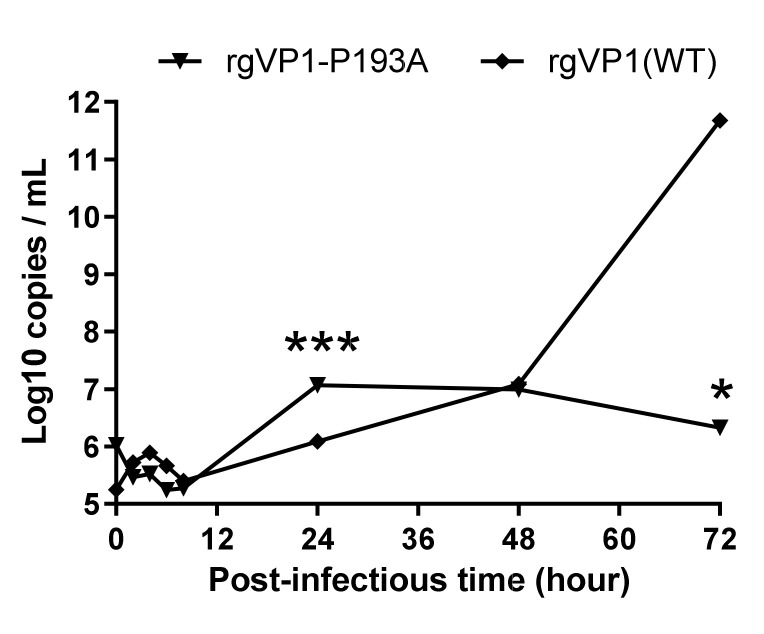
Viral RNA expression of rgVP1-P193A and rgVP1 wild type. Viral RNA replication after RNA transfection from reverse genetics variants were measured by real-time RT-PCR assay. The data were expressed as the mean ± SD of each group (n = 3). *, *p* < 0.05 and ***, *p* < 0.005.

To investigate the infectious titer and RNA level of the rg VP1 variants, CCID_50_ and real-time RT-PCR were performed after transfection. Results showed that infectious titer and RNA level significantly increased at days 2 and 3 in rgVP1-E134A and rgVP1-P157A (Figure 2C,D). There was no significant change in the RNA level in rgVP1-R86A, rgVP1-P193A, rgVP1-G231A, and rgVP1-K256A (Figure 2D). Notably, rgVP1-P193A had a higher RNA level compared to other variants on day 2. We further investigated rgVP1-P193A RNA levels in a time course of 0, 2, 4, 6, 24, 48, and 72 h (Figure 3). The results indicated that rgVP1-P193A rapidly plateaued 24 h after transfection, and no longer had RNA replication kinetics 48 and 72-h post-transfection. Of note, compared to rgVP1(WT), rgVP1-P193A had significantly lower RNA levels at 72 h. Three of four highly conserved residues adjacent to the β-barrel and loop intersection, VP1-86R, VP1-193P, and VP1-231G, play a role in viral infectious titer and RNA level in the viral replication process.

### 3.4. The Viral Properties of the rgVP1 Variants

**Figure 4:**
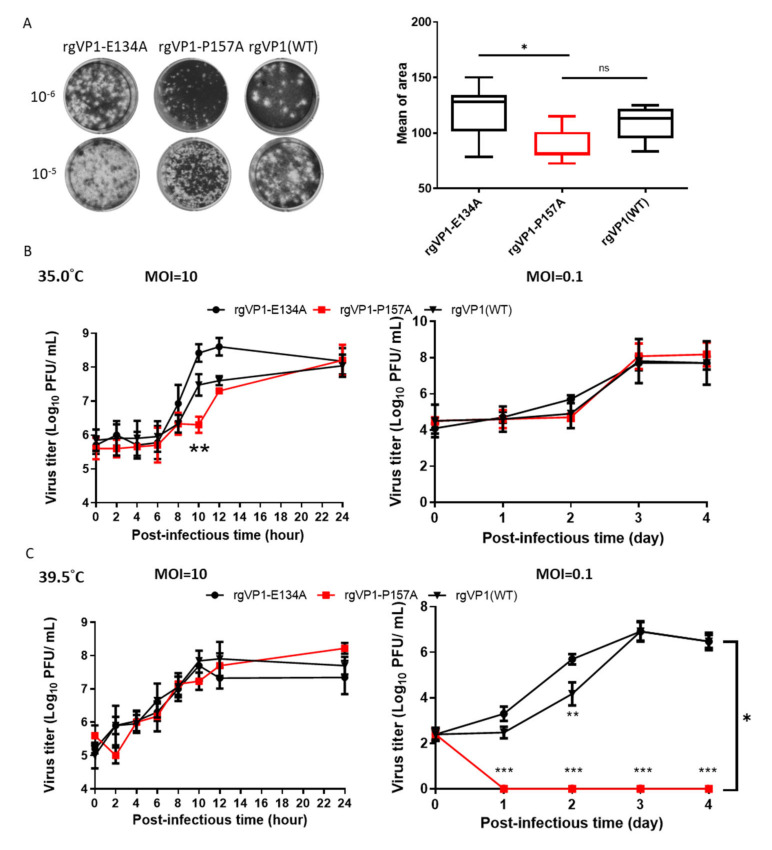
Viral plaque size, growth kinetics, temperature sensitivity, and stability of reverse genetics variants. (**A**) Diagram of the plaque size of 10^−5^- and 10^−6^-fold serially diluted viruses. The plaque area was measured by Image J2. Mean of plaque area is shown in the right figure. The rgVP1-P157A, rgVP1-E134A, and rgVP1(WT) growth kinetics assays were performed in triplicate at (**B**) 35 °C in MOI of 10 and 0.1 after the indicated time course and (**C**) at 39.5 °C. The data were expressed as the mean ± SD of each group (n = 3). n.s., no significance, *, *p* < 0.05, **, *p* < 0.01, and ***, *p* < 0.005.

To further elucidate, the first passage of the rg variants was prepared as viral stocks for the analysis of the viral properties. However, only the stocks of rgVP1-E134A and rgVP1-P157A were available, while stocks of the other four rgVP1 variants could not be obtained due to the lack of further replication ability. Plaque sizes of rgVP1-P157A variants were significantly smaller than rgVP1-E134A (Figure 4A). To investigate the effect of the substitutions in the rgVP1 variants on viral replication, we analyzed the virus growth kinetics at MOI of 10 and 0.1. At MOI of 10, the variants had similar endpoint titers at 24 h. The rgVP1-E134A had the highest titer log phase compared to rgVP1-P157A and rgVP1(WT). We also found that rgVP1-P157A had a significantly lower viral titer than rgVP1-E134A from 10 to 12 h post-infection at MOI of 10. However, the growth rate of rgVP1-E134A, rgVP1-P157A, and wild type were similar at MOI of 0.1 (Figure 4B).

### 3.5. The Viral Temperature Sensitivity and Stabilty of the rgVP1-P157A Variant of VP1

To observe the temperature sensitivity of rg variants during virus replication, we applied the same replication kinetic at 39.5 °C (Figure 4C). The results showed similar replication kinetics at MOI of 10, however, rgVP1-P157A was not able to further replicate at a MOI of 0.1 in the time course. This reveals that the low viral load of VP1-157P is sensitive at 39.5 °C during viral replication (Table 2).

**Figure 5:**
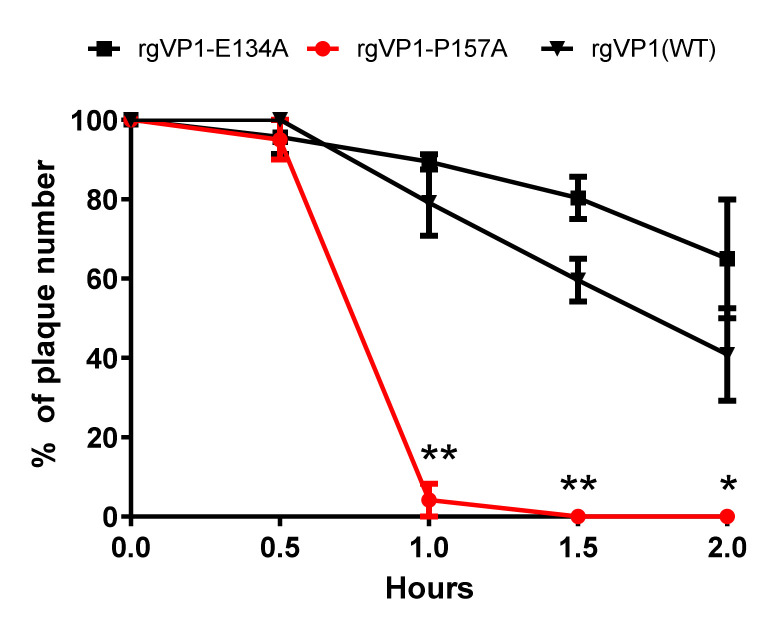
Effects of rgVP1-E134A and rgVP1-P157A variants on virus thermal stability. Thermostability assay was performed at 39.5 °C for 0.5, 1.0, 1.5, and 2.0 h. The data were expressed as the mean ± SD of each group (n = 3). *, *p* < 0.05, **, *p* < 0.01.

To elucidate whether rgVP1-P157A viral particles affect the thermal stability, we tested the thermal inactivation kinetics of the rg variants with the titers of 10,000 PFU/0.1 mL. Viruses were heated at 39.5 °C in a water bath for 0.5, 1, 1.5, and 2.0 h. Both rgVP1-E134A and rgVP1(WT) showed a decrease in plaque numbers from 8.6% to 29.2% after treatment for 1 h, while rgVP1-P157A dramatically decreased by 91.7% in titer (Figure 5). According to the results, highly conserved residues affect the plaque size, viral replication, thermal sensitivity, and stability. This indicates that the mutation of conserved residues might be lethal and could affect the viral properties.

### 3.6. Effect of Amino Acid Interactions after Alanine Substution

**Figure 6:**
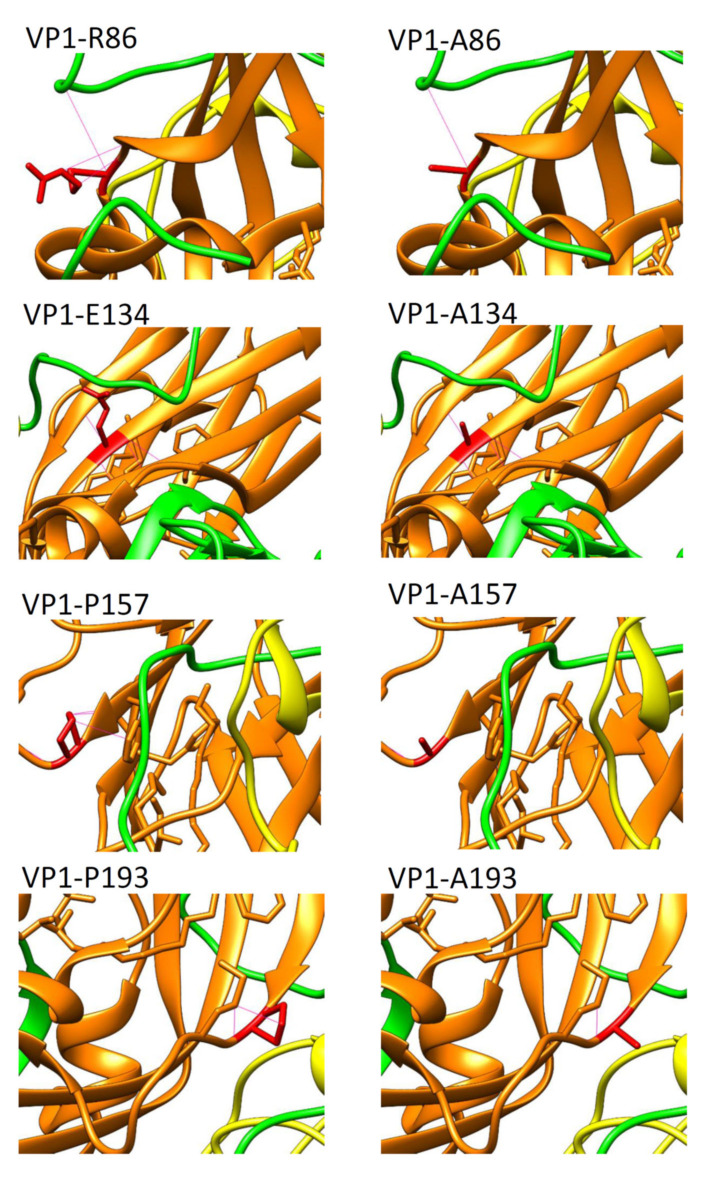

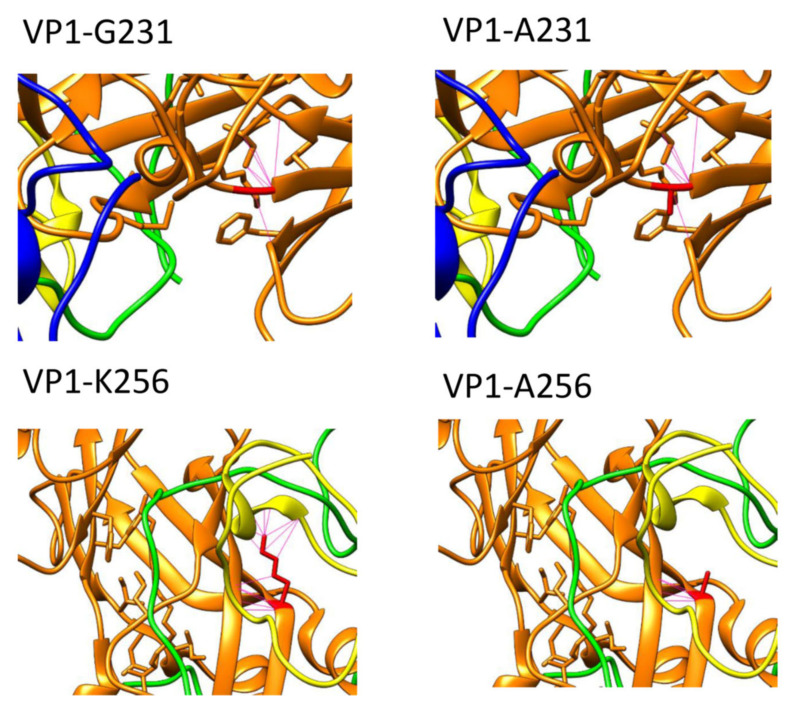
Effects of amino acid side-chain interaction after alanine substitution in six highly conserved residues of VP1. The hydrogen bonds interacting with other residues of six variants in UCSF chimera were shown. The wild-type residues are shown on the **left** and alanine substitutions on the **right**. The VP1 protein is shown in orange; the VP2 is shown in blue; the VP3 is shown in green; the VP4 is shown in yellow; the residues in the study are shown in red; the interaction bonds are shown as red lines.

To further examine the effects of alanine substitutions on capsid protein interaction, we utilized the UCSF chimera as a protein structure analysis tool to predict the capsid protein interaction. The results indicated that VP1-E134A and VP1-G231A showed no change in interaction with other residues; however, VP1-R86A, VP1-P157A, VP1-P193A, and VP1-K256A resulted in interaction alternation with the capsid proteins, as shown in Figure 6. Most of these residues demonstrated interaction loss after alanine substitution. The VP1-R86A and VP1-K256A not only presented a positive charge change, but also demonstrated interaction dismissed with VP3 and VP4, respectively. The data also demonstrated the loss of interactions with VP1 protein for the VP1-P157A, and the loss of one of the two interactions with the VP1 protein for the VP1-P193A (Figure 6).

## 4. Discussion

To date, there are no effective antiviral drugs against the highly diverse enteroviruses. According to our unpublished data, highly conserved residues of VP1 capsid protein among the *Enterovirus* genus (1) are important for viral structure stability; (2) interact with other capsid proteins; and (3) exist adjacent to the β-barrel and loop intersection. Additionally, most of these residues are close to the interior area. In this study, through reverse genetics variants of the conserved residues, we emphasized that the highly conserved residues in the junction of the ß-barrel and loop are important for virus replication kinetics, thermal sensitivity, and thermostability. We suggest that the enterovirus genus shares the same structure and conserved residues close to the inside of the canyon. According to the influenza virus study, focusing on a hemagglutinin stem structure may develop a broad spectrum drug for influenza virus infection. Small molecular drug development, which targets the inside of the canyon region, may also serve as a broad spectrum drug candidate for enterovirus treatment.

Our findings are consistent with a previous study by Yuan et al. (2016) using positive charged-to-alanine mutations of VP1-86R and VP1-256K. The study indicated that the VP1-86R mutation had a lower replication ability, and that the VP1-256K mutation had a lethal consequence [28]. Our results provided additional information based on the previous study such as the fact that not only positive charge amino acids are critical for replication, but the conserved residues in the ß-barrel and loop junction may also be important for the viral life cycle. VP1-86R had a higher flexibility than the other five residues. According to our previous data, a higher conservation score showed a lower flexibility. In addition, most highly conserved residues were located at the ß-barrel when compared with the loop (55% vs. 43%). In this study, we found that the highly conserved residues adjacent to the ß-barrel and loop intersection demonstrated a low fluctuation angle. The higher fluctuation angle of VP1-86R may represent its structure flexibility. VP1-86R was located at the N-terminus and ß-barrel B intersection, and the N-terminus did not connect to other rigid structures, which may lead to higher flexibility in VP1-86R. This observation indicates that not only the secondary structure, but the location of the residue also affects the structure flexibility. The data for rgVP1-P193A presented a higher RNA replication rate when compared with other rg variants, but no obvious CPE or viral titers were found on day 2. We believe that the RNA transcripts of VP1-P193A were packaged by lipofectamine and transfected into RD cells at the first replication cycle. The initial translation started immediately without an uncoating and RNA release process. The mature viral particles were subsequently released in a non-lytic way. The second round of the infectious process may fail due to receptor binding, uncoating, or RNA release [29]. This implies that the possible role of VP1-193P residues may relate to the initiation of viral infection or non-lytic release viral particles. In the analysis of the amino acid interaction simulation, we found that rgVP1-E134A demonstrated no effect on viral replication because there was no change in interaction with the other residues. Nevertheless, rgVP1-G231A also had no change in the interaction of the amino acid after alanine substitution, but the rgVP1-G231A variant was lethal. According to our study, the rgVP1-E134A was found located at the ß-barrel. Although there was no change in interaction after alanine substitution, the VP1-G231A was located in the junction between the ß-barrel and loop, which may have a pivotal role in capsid conformation stability.

In our unpublished data, a quarter of the highly conserved residues were found to be composed of arginine and proline in enterovirus VP1, where proline was located at the junction of the ß-barrel and loop and may maintain the capsid protein structure stability. VP1-157P and VP1-193P are located at the EF loop to ß-barrel F intersection and the ß-barrel G to GH loop intersection, respectively. It is worth noting that both are proline residues. In our experiment, we demonstrated that alanine substitutions of VP1-157P changed viral replication, thermal sensitivity, and stability. Proline residues are often found at the first residue of an alpha helix and in the edge strands of the ß-barrel of human proteomics [30]. The same phenomenon was observed in VP1 of the *Enterovirus* genus. Proline also plays important roles in viruses. For example, according to previous studies on mouse hepatitis virus (MHV; murine coronavirus), single and double proline mutations in fusion peptides of the spike protein are important for cell–cell fusion and pathogenesis [31,32]. Another previous study also showed that SCARB2 binds to the EV-A71 interactive complex structure by using cryo-electron microscopy, suggesting that conserved proline and glycine are key residues involved in the binding footprint of the EV-A71 canyon [33]. The critical amino acid proline residues in the conserved PXXXP motif of envelope glycoprotein I (gI) of herpes simplex virus 1 (HSV-1) are involved in viral spread and pathogenesis [34]. The identification of proline hot spots may facilitate the design of mimic therapeutics, which are less likely to generate resistance quickly. Proline is also important for vaccine efficiency. Pallesen et al. (2017) reported two proline substitutions (K986P and V987P) at the S protein apex of Middle East respiratory syndrome coronavirus (MERS-CoV), resulting in the induction of a much greater antibody titer than wild-type S protein in mice [35]. Nowadays, the design strategy of having two proline substitutions at the S protein is used in several vaccines against the coronavirus disease of 2019 (COVID-19). Our study suggests that the conserved VP1-157P and VP1-193P residues may play a role in *Enterovirus* genus replication and virulence.

Since the enterovirus species show remarkable structural similarity, we tried to identify the most conserved residues for efficient treatment strategies that may cover a wider range of enteroviruses. In this study, we identified that alanine substitutions at VP1-86R, VP1-193P, VP1-231G, or VP1-256K would lead to severely impaired viral replication. Alanine substitutions at VP1-157P resulted in thermal instability. Both rgVP1-E134A and rgVP1-P157A can replicate and produce infectious viral particles, even though rgVP1-P157A showed reduced replication, despite a normal binding ability. No significant differences in virus binding ability was observed among the variants, indicating that these residues may not be involved in the binding process during infection (data not shown). This may be due to the fact that the residues are not exposed on the surface of the capsid. Therefore, we believe that rgVP1-P157A is a low virulence variant and may bind to cells prior to initiating the first step of infection and potential antiviral immune response. This entails the possibility that the variant may serve as a potential backbone for future vaccine development. The data of VP1-157P have been shown to be temperature sensitive and unstable at 39.5 °C. Proline amino acid structure is important for protein thermostability and can be found in a high percentage of thermophilic organisms [36]. The loss of proline residues may be the reason as to why rgVP1-P157A is temperature sensitive. Although the link between temperature sensitivity and attenuation may not be straightforward, it could serve as an indicator of virulence in enterovirus [37].

The residues in the junction between the ß-barrel and loop play an important role in viral replication and structural stability. Cyclophilin A, peptidyl-prolyl cis-trans isomerase, is involved in the pathogenesis of EV-A71 infection [38]. Proline isomerase also modulates protein stability in oncogenic viruses, like HBV, HIV, and HTLV-1 [39]. Previous studies have reported that cyclophilin inhibitors could serve as potential novel HCV therapy drugs [40]. Our study suggests that highly conserved proline, adjacent to the ß-barrel and loop intersection, may participate in proline isomerization during the VP1 folding or assembling process. Proline isomerase inhibitors may be a potential tool for the broad spectrum of anti-enteroviral drug development.

## 5. Conclusions

In conclusion, highly conserved residues were distributed in the interior of the canyon of enteroviruses. According to the deduced amino acid sequence, six residues among the *Enterovirus* genus were identified by alignment. We identified four residues adjacent to the ß-barrel and loop intersection surrounding the interior canyon. Through alanine substitution, four residues affected viral replication. VP-157P was further identified to affect viral replication, plaque size, and thermostability. This study will help explore the importance of amino acid residues or areas in VP1 that may provide potential targets for developing new antiviral therapy in the future.

## Figures and Tables

**Table 1:** Cytopathic effect and immunofluorescence stain of the rgVP1 variants.

Variants		rgVP1- R86A	rgVP1- E134A	rgVP1- P157A	rgVP1- P193A	rgVP1- G231A	rgVP1- K256A
P0 ^1^	CPE	2/5 ^2^	5/5	5/5	1/5	3/5	0/5
	IFA ^1^	3/5	5/5	5/5	1/5	3/5	1/5
P1	CPE	1/5	5/5	5/5	—	1/5	—
	IFA	1/5	5/5	5/5	—	1/5	—
P2–P5 ^3^	CPE	—	5/5	5/5	—	—	—
	IFA	—	5/5	5/5	—	—	—

^1^ P, passage; CPE, cytopathic effect; IFA, immunofluorescence assay. ^2^ The total number of positive CPE or IFA results/The total number of experiments. ^3^—, no CPE or IFA positive results demonstrated after passaging five times.

**Table 2:** Analysis of the thermal sensitivity of rgVP1(WT) VP1 variants.

Viruses (MOI = 0.1)	Titer at: (log_10_ PFU/mL)		∆log ^1^	Phenotype
	35 °C	39.5 °C		
rgVP1-E134A	7.70	6.5	1.20	TR ^2^
rgVP1-P157A	8.18	0	8.18	TS ^2^
rgVP1(WT)	7.70	6.5	1.20	TR

^1^ ∆log = log(35 °C)–log(39.5 °C). ^2^ TR: temperature resistant, defined as logarithmic difference <2.00; TS: temperature-sensitive defined as logarithmic difference >2.00.

## Data Availability

Not applicable.

## Supplementary Materials

The following supporting information can be downloaded at: https://www.mdpi.com/article/10.3390/v14020364/s1, Table S1: Overlap extension PCR primers used for site-directed mutagenesis. File S1: Structure-based sequence alignment of VP1 in enteroviruses. The secondary structural elements for EV-A71 are shown, above the EV-A71 sequence are also symbols that show features described as follows. The residue numbers are for EV-A71. A total of 1632 protein sequences were aligned with the VP1 protein (PDB code 4AED). Red block marks conserved residues.
