# Supplementary material for: Conserved Residues Adjacent to ß-Barrel and Loop Intersection among Enterovirus VP1 Affect Viral Replication: Potential Target for Anti-Enteroviral Development
Source: Viruses. 2022 Feb 10;14(2):364. doi: 10.3390/v14020364 (PMC8877150; doi:10.3390/v14020364)
Supplement: Supplementary file 1 [file viruses-14-00364-s001.zip › Supplementary File S1.pdf]

```
                   α1                  βA                                         η1          η2
QUS47950.1
                 1        10         20        30        40        50        60        70
QUS47950.1  .....GDRVADVIESSIGDSVSR.ALTHALPAPTGQNTQVSSHRLDTGKVPALQAAEIGASSNASDESMIETRCVLNSHS
QWY16112.1  .....GDRVADVIESSIGDSVSR.ALTHALPAPTGQNTQVSSHRLDTGKVPALQAAEIGASSNASDESMIETRCVLNSHS
QWY16110.1  .....GDRVADVIESSIGDSVSR.ALTHALPAPTGQNTQVSSHRLDTGKVPALQAAEIGASSNASDESMIETRCVLNSHS
QWY16109.1  .....GDRVADVIESSIGDSVSR.ALTHALPAPTGQNTQVSSHRLDTGKVPALQAAEIGASSNASDESMIETRCVLNSHS
QWY16107.1  .....GDRVADVIESSIGDSVSR.ALTHALPAPTGQNTQVSSHRLDTGKVPALQAAEIGASSNASDESMIETRCVLNSHS
QWY16106.1  .....GDRVADVIESSIGDSVSR.ALTHALPAPTGQNTQVSSHRLDTGKVPALQAAEIGASSNASDESMIETRCVLNSHS
QWY16105.1  .....GDRVADVIESSIGDSMSR.ALTHALPAPTGQNTQVSSHRLDTGKVPALQAAEIGASSNASDESMIETRCVLNSHS
QWY16104.1  .....GDRVADVIESSIGDSMSR.ALTHALPAPTGQNTQVSSHRLDTGKVPALQAAEIGASSNASDESMIETRCVLNSHS
QNN25841.1  .....GDRVADVIESSIGDSVSR.ALTHVLPAPTGQNTQVSSHRLDTGKVPALQAAEIGASSNASDESMIETRCVLNSHS
QNN25838.1  .....GDRVADVIESSIGDSVSR.ALTHVLPAPTGQNTQVSSHRLDTGKVPALQAAEIGASSNASDESMIETRCVLNSHS
QNN25837.1  .....GDRVADVIESSIGDSVSR.ALTHVLPAPTGQNTQVSSHRLDTGKVPALQAAEIGASSNASDESMIETRCVLNSHS
QNN25842.1  .....GDRVADVIESSIGDSVSR.ALTHALPAPTGQNTQVSSHRLDTGKVPALQAAEIGASSNASDESMIETRCVLNSHS
QNN25843.1  .....GDRVADVIESSIGDSVSR.ALTHALPAPTGQNTQVSSHRLDTGKVPALQAAEIGASSNASDESMIETRCVLNSHS
QWY16092.1  .....GDRVADVIESSIGDSVSR.ALTHALPAPTGQNTQVSSHRLDTGKVPALQAAEIGASSNASDESMIETRCVLNSHS
QWY16090.1  .....GDRVADVIESSIGDSVSR.ALTHALPAPTGQNTQVSSHRLDTGKVPALQAAEIGASSNASDESMIETRCVLNSHS
QWY16084.1  .....GDRVADVIESSIGDSVSR.ALTHALPAPTGQNTQVSSHRLDTGKVPALQAAEIGASSNASDESMIETRCVLNSHS
QWY16083.1  .....GDRVADVIESSIGDSVSR.ALTHALPAPTGQNTQVSSHRLDTGKVPALQAAEIGASSNASDESMIETRCVLNSHS
QWY16081.1  .....GDRVADVIESSIGDSVSR.ALTHALPAPTGQNTQVSSHRLDTGKVPALQAAEIGASSNASDESMIETRCVLNSHS
QWY16080.1  .....GDRVADVIESSIGDSVSR.ALTHALPAPTGQNTQVSSHRLDTGKVPALQAAEIGASSNASDESMIETRCVLNSHS
QWY16079.1  .....GDRVADVIESSIGDSVSR.ALTHALPAPTGQNTQVSSHRLDTGKVPALQAAEIGASSNASDESMIETRCVLNSHS
QWY16078.1  .....GDRVADVIESSIGDSVSR.ALTHALPAPTGQNTQVSSHRLDTGKVPALQAAEIGASSNASDESMIETRCVLNSHS
QWY16075.1  .....GDRVADVIESSIGDSVSR.ALTHALPAPTGQNTQVSSHRLDTGKVPALQAAEIGASSNASDESMIETRCVLNSHS
QWY16074.1  .....GDRVADVIESSIGDSVSR.ALTHALPAPTGQNTQVSSHRLDTGKVPALQAAEIGASSNASDESMIETRCVLNSHS
QWY16056.1  .....GDRVADVIESSIGDSVSR.ALTHALPAPTGQNTQVSSHRLDTGKVPALQAAEIGASSNASDESMIETRCVLNSHS
QWY16147.1  .....GDRVADVIESSIGDSVSR.ALTHALPAPTGQNTQVSSHRLDTGKVPALQAAEIGASSNASDESMIETRCVLNSHS
QWY16146.1  .....GDRVADVIESSIGDSVSR.ALTHALPAPTGQNTQVSSHRLDTGKVPALQAAEIGASSNASDESMIETRCVLNSHS
QOP74909.1  .....GDRVADVIESSIGDSVSR.ALTHALPAPTGQNTQVSSHRLDTGKVPALQAAEIGASSNASDESMIETRCVLNSHS
QUS47948.1  .....GDRVADVIESSIGDSVSR.ALTHALPAPTGQNTQVSSHRLDTGKVPALQAAEIGASSNASDESMIETRCVLNSHS
QUS47947.1  .....GDRVADVIESSIGDSVSR.ALTHALPAPTGQNTQVSSHRLDTGKVPALQAAEIGASSNASDESMIETRCVLNSHS
QUS47946.1  .....GDRVADVIESSIGDSVSR.ALTHALPAPTGQNTQVSSHRLDTGKVPALQAAEIGASSNASDESMIETRCVLNSHS
QUS47944.1  .....GDRVADVIESSIGDSVSR.ALTHALPAPTGQNTQVSSHRLDTGKVPALQAAEIGASSNASDESMIETRCVLNSHS
QUS47942.1  .....GDRVADVIESSIGDSVSR.ALTHALPAPTGQNTQVSSHRLDTGKVPALQAAEIGASSNASDESMIETRCVLNSHS
QUS47941.1  .....GDRVADVIESSIGDSVSR.ALTHALPAPTGQNTQVSSHRLDTGKVPALQAAEIGASSNASDESMIETRCVLNSHS
QUS47940.1  .....GDRVADVIESSIGDSVSR.ALTHALPAPTGQNTQVSSHRLDTGKVPALQAAEIGASSNASDESMIETRCVLNSHS
QUS47939.1  .....GDRVADVIESSIGDSVSR.ALTHALPAPTGQNTQVSSHRLDTGKVPALQAAEIGASSNASDESMIETRCVLNSHS
QUS47928.1  .....GDRVADVIESSIGDSVSR.ALTHALPAPTGQNTQVSSHRLDTGKVPALQAAEIGASSNASDESMIETRCVLNSHS
QUS47926.1  .....GDRVADVIESSIGDSVSR.ALTHALPAPTGQNTQVSSHRLDTGKVPALQAAEIGASSNASDESMIETRCVLNSHS
QUS47921.1  .....GDRVADVIESSIGDSVSR.ALTHALPAPTGQNTQVSSHRLDTGKVPALQAAEIGASSNASDESMIETRCVLNSHS
QUS47920.1  .....GDRVADVIESSIGDSVSR.ALTHALPAPTGQNTQVSSHRLDTGKVPALQAAEIGASSNASDESMIETRCVLNSHS
QNN25845.1  .....GDRVADVIESSIGDSVSR.ALTHALPAPTGQNTQVSSHRLDTGKVPALQAAEIGASSNASDESMIETRCVLNSHS
QNN25836.1  .....GDRVADVIESSIGDSVSR.ALTHALPAPTGQNTQVSSHRLDTGKVPALQAAEIGASSNASDESMIETRCVLNSHS
QWY16162.1  .....GDRVADVIESSIGDSVSR.ALTHALPAPTGQNTQVSSHRLDTGKVPALQAAEIGASSNASDESMIETRCVLNSHS
QWY16161.1  .....GDRVADVIESSIGDSVSR.ALTHALPAPTGQNTQVSSHRLDTGKVPALQAAEIGASSNASDESMIETRCVLNSHS
QWY16159.1  .....GDRVADVIESSIGDSVSR.ALTHALPAPTGQNTQVSSHRLDTGKVPALQAAEIGASSNASDESMIETRCVLNSHS
QWY16158.1  .....GDRVADVIESSIGDSVSR.ALTHALPAPTGQNTQVSSHRLDTGKVPALQAAEIGASSNASDESMIETRCVLNSHS
QWY16155.1  .....GDRVADVIESSIGDSVSR.ALTHALPAPTGQNTQVSSHRLDTGKVPALQAAEIGASSNASDESMIETRCVLNSHS
QWY16153.1  .....GDRVADVIESSIGDSVSR.ALTHALPAPTGQNTQVSSHRLDTGKVPALQAAEIGASSNASDESMIETRCVLNSHS
QWY16151.1  .....GDRVADVIESSIGDSVSR.ALTHALPAPTGQNTQVSSHRLDTGKVPALQAAEIGASSNASDESMIETRCVLNSHS
QWY16145.1  .....GDRVADVIESSIGDSVSR.ALTHALPAPTGQNTQVSSHRLDTGKVPALQAAEIGASSNASDESMIETRCVLNSHS
QWY16143.1  .....GDRVADVIESSIGDSVSR.ALTHALPAPTGQNTQVSSHRLDTGKVPALQAAEIGASSNASDESMIETRCVLNSHS
QWY16142.1  .....GDRVADVIESSIGDSVSR.ALTHALPAPTGQNTQVSSHRLDTGKVPALQAAEIGASSNASDESMIETRCVLNSHS
QWY16139.1  .....GDRVADVIESSIGDSVSR.ALTHALPAPTGQNTQVSSHRLDTGKVPALQAAEIGASSNASDESMIETRCVLNSHS
QWY16138.1  .....GDRVADVIESSIGDSVSR.ALTHALPAPTGQNTQVSSHRLDTGKVPALQAAEIGASSNASDESMIETRCVLNSHS
QWY16135.1  .....GDRVADVIESSIGDSVSR.ALTHALPAPTGQNTQVSSHRLDTGKVPALQAAEIGASSNASDESMIETRCVLNSHS
QWY16134.1  .....GDRVADVIESSIGDSVSR.ALTHALPAPTGQNTQVSSHRLDTGKVPALQAAEIGASSNASDESMIETRCVLNSHS
QWY16133.1  .....GDRVADVIESSIGDSVSR.ALTHALPAPTGQNTQVSSHRLDTGKVPALQAAEIGASSNASDESMIETRCVLNSHS
QWY16132.1  .....GDRVADVIESSIGDSVSR.ALTHALPAPTGQNTQVSSHRLDTGKVPALQAAEIGASSNASDESMIETRCVLNSHS
QWY16131.1  .....GDRVADVIESSIGDSVSR.ALTHALPAPTGQNTQVSSHRLDTGKVPALQAAEIGASSNASDESMIETRCVLNSHS
QWY16130.1  .....GDRVADVIESSIGDSVSR.ALTHALPAPTGQNTQVSSHRLDTGKVPALQAAEIGASSNASDESMIETRCVLNSHS
QWY16129.1  .....GDRVADVIESSIGDSVSR.ALTHALPAPTGQNTQVSSHRLDTGKVPALQAAEIGASSNASDESMIETRCVLNSHS
QWY16127.1  .....GDRVADVIESSIGDSVSR.ALTHALPAPTGQNTQVSSHRLDTGKVPALQAAEIGASSNASDESMIETRCVLNSHS
QWY16125.1  .....GDRVADVIESSIGDSVSR.ALTHALPAPTGQNTQVSSHRLDTGKVPALQAAEIGASSNASDESMIETRCVLNSHS
QWY16123.1  .....GDRVADVIESSIGDSVSR.ALTHALPAPTGQNTQVSSHRLDTGKVPALQAAEIGASSNASDESMIETRCVLNSHS
QWY16121.1  .....GDRVADVIESSIGDSVSR.ALTHALPAPTGQNTQVSSHRLDTGKVPALQAAEIGASSNASDESMIETRCVLNSHS
QWY16118.1  .....GDRVADVIESSIGDSVSR.ALTHALPAPTGQNTQVSSHRLDTGKVPALQAAEIGASSNASDESMIETRCVLNSHS
QWY16117.1  .....GDRVADVIESSIGDSVSR.ALTHALPAPTGQNTQVSSHRLDTGKVPALQAAEIGASSNASDESMIETRCVLNSHS
QWY16116.1  .....GDRVADVIESSIGDSVSR.ALTHALPAPTGQNTQVSSHRLDTGKVPALQAAEIGASSNASDESMIETRCVLNSHS
QWY16099.1  .....GDRVADVIESSIGDSVSR.ALTHALPAPTGQNTQVSSHRLDTGKVPALQAAEIGASSNASDESMIETRCVLNSHS
QWY16096.1  .....GDRVADVIESSIGDSVSR.ALTHALPAPTGQNTQVSSHRLDTGKVPALQAAEIGASSNASDESMIETRCVLNSHS
QWY16095.1  .....GDRVADVIESSIGDSVSR.ALTHALPAPTGQNTQVSSHRLDTGKVPALQAAEIGASSNASDESMIETRCVLNSHS
QWY16085.1  .....GDRVADVIESSIGDSVSR.ALTHALPAPTGQNTQVSSHRLDTGKVPALQAAEIGASSNASDESMIETRCVLNSHS
QWY16077.1  .....GDRVADVIESSIGDSVSR.ALTHALPAPTGQNTQVSSHRLDTGKVPALQAAEIGASSNASDESMIETRCVLNSHS
QWY16072.1  .....GDRVADVIESSIGDSVSR.ALTHALPAPTGQNTQVSSHRLDTGKVPALQAAEIGASSNASDESMIETRCVLNSHS
QWY16071.1  .....GDRVADVIESSIGDSVSR.ALTHALPAPTGQNTQVSSHRLDTGKVPALQAAEIGASSNASDESMIETRCVLNSHS
QWY16064.1  .....GDRVADVIESSIGDSVSR.ALTHALPAPTGQNTQVSSHRLDTGKVPALQAAEIGASSNASDESMIETRCVLNSHS
QWY16059.1  .....GDRVADVIESSIGDSVSR.ALTHALPAPTGQNTQVSSHRLDTGKVPALQAAEIGASSNASDESMIETRCVLNSHS
QWY16054.1  .....GDRVADVIESSIGDSVSR.ALTHALPAPTGQNTQVSSHRLDTGKVPALQAAEIGASSNASDESMIETRCVLNSHS
QWY16052.1  .....GDRVADVIESSIGDSVSR.ALTHALPAPTGQNTQVSSHRLDTGKVPALQAAEIGASSNASDESMIETRCVLNSHS
QWY16051.1  .....GDRVADVIESSIGDSVSR.ALTHALPAPTGQNTQVSSHRLDTGKVPALQAAEIGASSNASDESMIETRCVLNSHS
QWY16050.1  .....GDRVADVIESSIGDSVSR.ALTHALPAPTGQNTQVSSHRLDTGKVPALQAAEIGASSNASDESMIETRCVLNSHS
QWY16046.1  .....GDRVADVIESSIGDSVSR.ALTHALPAPTGQNTQVSSHRLDTGKVPALQAAEIGASSNASDESMIETRCVLNSHS
QWY16045.1  .....GDRVADVIESSIGDSVSR.ALTHALPAPTGQNTQVSSHRLDTGKVPALQAAEIGASSNASDESMIETRCVLNSHS
QWY16041.1  .....GDRVADVIESSIGDSVSR.ALTHALPAPTGQNTQVSSHRLDTGKVPALQAAEIGASSNASDESMIETRCVLNSHS
QWY16040.1  .....GDRVADVIESSIGDSVSR.ALTHALPAPTGQNTQVSSHRLDTGKVPALQAAEIGASSNASDESMIETRCVLNSHS
QWY16032.1  .....GDRVADVIESSIGDSVSR.ALTHALPAPTGQNTQVSSHRLDTGKVPALQAAEIGASSNASDESMIETRCVLNSHS
QWY16017.1  .....GDRVADVIESSIGDSVSR.ALTHALPAPTGQNTQVSSHRLDTGKVPALQAAEIGASSNASDESMIETRCVLNSHS
QWY16016.1  .....GDRVADVIESSIGDSVSR.ALTHALPAPTGQNTQVSSHRLDTGKVPALQAAEIGASSNASDESMIETRCVLNSHS
QWY16015.1  .....GDRVADVIESSIGDSVSR.ALTHALPAPTGQNTQVSSHRLDTGKVPALQAAEIGASSNASDESMIETRCVLNSHS
QTA73890.1  .....GDRVADVIESSIGDSVSR.ALTHALPAPTGQNTQVSSHRLDTGKVPALQAAEIGASSNASDESMIETRCVLNSHS
QTA73885.1  .....GDRVADVIESSIGDSVSR.ALTHALPAPTGQNTQVSSHRLDTGKVPALQAAEIGASSNASDESMIETRCVLNSHS
BCG67033.1  .....GDRVADVIESSIGDSVSR.ALTHALPAPTGQNTQVSSHRLDTGKVPALQAAEIGASSNASDESMIETRCVLNSHS
BBU37474.1  .....GDRVADVIESSIGDSVSR.ALTHALPAPTGQNTQVSSHRLDTGKVPALQAAEIGASSNASDESMIETRCVLNSHS
BBU37473.1  .....GDRVADVIESSIGDSVSR.ALTHALPAPTGQNTQVSSHRLDTGKVPALQAAEIGASSNASDESMIETRCVLNSHS
BBU37471.1  .....GDRVADVIESSIGDSVSR.ALTHALPAPTGQNTQVSSHRLDTGKVPALQAAEIGASSNASDESMIETRCVLNSHS
BBU37469.1  .....GDRVADVIESSIGDSVSR.ALTHALPAPTGQNTQVSSHRLDTGKVPALQAAEIGASSNASDESMIETRCVLNSHS
BBU37468.1  .....GDRVADVIESSIGDSVSR.ALTHALPAPTGQNTQVSSHRLDTGKVPALQAAEIGASSNASDESMIETRCVLNSHS
BBU37467.1  .....GDRVADVIESSIGDSVSR.ALTHALPAPTGQNTQVSSHRLDTGKVPALQAAEIGASSNASDESMIETRCVLNSHS
BBU37466.1  .....GDRVADVIESSIGDSVSR.ALTHALPAPTGQNTQVSSHRLDTGKVPALQAAEIGASSNASDESMIETRCVLNSHS
BBU37465.1  .....GDRVADVIESSIGDSVSR.ALTHALPAPTGQNTQVSSHRLDTGKVPALQAAEIGASSNASDESMIETRCVLNSHS
BBU37464.1  .....GDRVADVIESSIGDSVSR.ALTHALPAPTGQNTQVSSHRLDTGKVPALQAAEIGASSNASDESMIETRCVLNSHS
BBU37463.1  .....GDRVADVIESSIGDSVSR.ALTHALPAPTGQNTQVSSHRLDTGKVPALQAAEIGASSNASDESMIETRCVLNSHS
QOP74908.1  .....GDRVADVIESSIGDSVSR.ALTHALPAPTGQNTQVSSHRLDTGKVPALQAAEIGASSNASDESMIETRCVLNSHS
QOP74907.1  .....GDRVADVIESSIGDSVSR.ALTHALPAPTGQNTQVSSHRLDTGKVPALQAAEIGASSNASDESMIETRCVLNSHS
QOP74903.1  .....GDRVADVIESSIGDSVSR.ALTHALPAPTGQNTQVSSHRLDTGKVPALQAAEIGASSNASDESMIETRCVLNSHS
QOP74902.1  .....GDRVADVIESSIGDSVSR.ALTHALPAPTGQNTQVSSHRLDTGKVPALQAAEIGASSNASDESMIETRCVLNSHS
QOP74899.1  .....GDRVADVIESSIGDSVSR.ALTHALPAPTGQNTQVSSHRLDTGKVPALQAAEIGASSNASDESMIETRCVLNSHS
QOP74896.1  .....GDRVADVIESSIGDSVSR.ALTHALPAPTGQNTQVSSHRLDTGKVPALQAAEIGASSNASDESMIETRCVLNSHS
QWY16156.1  .....GDRVADVIESSIGDSVSR.ALTHALPAPTGQNTQVSSHRLDTGKVPALQAAEIGASSNASDESMIETRCVLNSHS
QUS47932.1  .....GDRVADVIESSIGDSVSR.ALTHALPAPTGQNTQVSSHRLDTGKVPALQAAEIGASSNASDESMIETRCVLNSHS
QWY16136.1  .....GDRVADVIESSIGDSVSR.ALTHALPAPTGQNTQVSNHRLDTGKVPALQAAEIGASSNASDESMIETRCVLNSHS
QWY16044.1  .....GDRVADVIESSIGDSVSR.AFTHALPAPTGQNTQVSSHRLDTGKVPALQAAEIGASSNASDESMIETRCVLNSHS
QUS47923.1  .....GDRVADVIESSIGDSVSR.ALTHALPAPTGQNTQVSSHRLDTGKVPALQAAEIGVSSNASDESMIETRCVLNSHS
QWY16152.1  .....GDRVADVIESSIGDSVSR.ALTHALPAPTGQNTQVSSHRLDTGKVPALQAAEIGASSNASDESMIETRCVLNSHS
QWY16124.1  .....GDRVADVIESSIGDSVSR.ALTHALPAPTGQNTQVSSHRLDTGKVPALQAAEIGASSNASDESMIETRCVLNSHS
QWY16018.1  .....GDRVADVIESSIGDSVSR.ALTHALPAPTGQNTQVSSHRLDTGKVPALQAAEIGASSNASDESMIETRCVLNSHS
BBU37475.1  .....GDRVADVIESSIGDSVSR.ALTHALPAPTGQNTQVSSHRLDTGKVPALQAAEIGASSNASDESMIETRCVLNSHS
QUS47938.1  .....GDRVADVIESSIGDSVSR.ALTHALPAPTGQNTQVSSHRLDTGKVPALQAAEIGASSNASDESMIETRCVLNSHS
QWY16076.1  .....GDRVADVIESSIGDSVSR.ALTHALPAPTGQNTQVSSHRLDTGKVPALQAAEIGASSNASDESMIETRCVLNSHS
QWY16122.1  .....GDRVADVIESSIGDSVSR.ALTHALPAPTGQDTQVSSHRLDTGKVPALQAAEIGASSNASDESMIETRCVLNSHS
QOP74905.1  .....GDRVADVIESSIGDSVSR.ALTHALPAPTGQNTQVSSHRLDTGKVPALQAAEIGASSNASDESMIETRCVLNSHS
QWY16160.1  .....GDRVADVIESSIGDSVSR.ALTHALPAPTGQNTQVSSHRLDTGKVPALQAAEIGASSNASDESMIETRCVLNSHS
QWY16070.1  .....GDRVADVIESSIGDSVSR.ALTHALPAPTGQNTQVSSHRLDTGKVPALQAAEIGASSNASDESMIETRCVLNSHS
QWY16068.1  .....GDRVADVIESSIGDSVSR.ALTHALPAPTGQNTQVSSHRLDTGKVPALQAAEIGASSNASDESMIETRCVLNSHS
QWY16066.1  .....GDRVADVIESSIGDSVSR.ALTHALPAPTGQNTQVSSHRLDTGKVPALQAAEIGASSNASDESMIETRCVLNSHS
QWY16062.1  .....GDRVADVIESSIGDSVSR.ALTHALPAPTGQNTQVSSHRLDTGKVPALQAAEIGASSNASDESMIETRCVLNSHS
QWY16034.1  .....GDRVADVIESSIGDSVSR.ALTHALPAPTGQNTQVSSHRLDTGKVPALQAAEIGASSNASDESMIETRCVLNSHS
QTA73887.1  .....GDRVADVIESSIGDSVSR.ALTHALPAPTGQNTQVSSHRLDTGKVPALQAAEIGASSNASDESMIETRCVLNSHS
QTA73883.1  .....GDRVADVIESSIGDSVSR.ALTHALPAPTGQNTQVSSHRLDTGKVPALQAAEIGASSNASDESMIETRCVLNSHS
BBU37478.1  .....GDRVADVIESSIGDSVSR.ALTHALPAPTGQNTQVSSHRLDTGKVPALQAAEIGASSNASDESMIETRCVLNSHS
BBU37470.1  .....GDRVADVIESSIGDSVSR.ALTHALPAPTGQNTQVSSHRLDTGKVPALQAAEIGASSNASDESMIETRCVLNSHS
QWY16103.1  .....GDRVADVIESSIGDSVSR.ALTHALPAPTGQNTQVSSHRLDTGKVPALQAAEIGASSNASDESMIETRCVLNSHS
QWY16065.1  .....GDRVADVIESSIGDSVSR.ALTHALPAPTGQNTQVSSHRLDTGKVPALQAAEIGASSNASDESMIETRCVLNSHS
QTA73886.1  .....GDRVADVIESSIGDSVSR.ALTHALPAPTGQNTQVSSHRLDTGKVPALQAAEIGASSNASDESMIETRCVLNSHS
QWY16113.1  .....GDRVADVIESSIGDSVSR.ALTHALPAPTGQNTQVSSHRLDTGKVPALQAAEIGASSNASDESMIETRCVLNSHS
QWY16111.1  .....GDRVADVIESSIGDSVSR.ALTHALPAPTGQNTQVSSHRLDTGKVPALQAAEIGASSNASDESMIETRCVLNSHS
QWY16098.1  .....GDRVADVIESSIGDSVSR.ALTHALPAPTGQNTQVSSHRLDTGKVPALQAAEIGASSNASDESMIETRCVLNSHS
QWY16060.1  .....GDRVADVIESSIGDSVSR.ALTHALPAPTGQNTQVSSHRLDTGKVPALQAAEIGASSNASDESMIETRCVLNSHS
QUS47945.1  .....GDRVADVIESSIGDSVSR.ALTHALPAPTGQNTQVSSHRLDTGKVPALQAAEIGASSNASDESMIETRCVLNSHS
QOP74904.1  .....GDRVADVIESSIGDSVSR.ALTHALPAPTGQNTQVSSHRLDTGKVPALQAAEIGASSNASDESMIETRCVLNSHS
QUS47924.1  .....GDRVADVIESSIGDSVSR.ALTHALPAPTGQNTQVSSHRLDTGKVPALQAAEIGASSNASDESMIETRCVLNSHS
QWY15991.1  .....GDRVADVIESSIGDSVSR.ALTHALPAPTGQNTQVSSHRLDTGKVPALQAAEIGASSNASDESMIETRCVLNSHS
QWY15985.1  .....GDRVADVIESSIGDSVSR.ALTHALPAPTGQNTQVSSHRLDTGKVPALQAAEIGASSNASDESMIETRCVLNSHS
QWY16165.1  .....GDRVADVIESSIGDSVSR.ALTHALPAPTGQNTQVSSHRLDTGKVPALQAAEIGASSNASDESMIETRCVLNSHS
QWY16038.1  .....GDRVADVIESSIGDSVSR.ALTHALPAPTGQNTQVSSHRLDTGKVPALQAAEIGASSNASDESMIETRCVLNSHS
```

```
QWY16036.1   .....GDRVADVIESSIGDSVSR.ALTHALPAPTGQNTQVSSHRLDTGKVPALQAAEIGASSNASDESMIETRCVLNSHS
QWY16120.1   .....GDRVADVIESSIGDSVSR.ALTHALPAPTGQNTQVSSHRLDTGKVPALQAAEIGASSNASDESMIETRCVLNSHS
QWY16119.1   .....GDRVADVIESSIGDSVSR.ALTHALPAPTGQNTQVSSHRLDTGKVPALQAAEIGASSNASDESMIETRCVLNSHS
QWY16102.1   .....GDRVADVIESSIGDSVSR.ALTHALPAPTGQNTQVSSHRLDTGKVPALQAAEIGASSNASDESMIETRCVLNSHS
QWY16100.1   .....GDRVADVIESSIGDSVSR.ALTHALPAPTGQNTQVSSHRLDTGKVPALQAAEIGASSNASDESMIETRCVLNSHS
QWY16097.1   .....GDRVADVIESSIGDSVSR.ALTHALPAPTGQNTQVSSHRLDTGKVPALQAAEIGASSNASDESMIETRCVLNSHS
QWY16101.1   .....GDRVADVIESSIGDSVSR.ALTHALPAPTGQNTQVSSHRLDTGKVPALQAAEIGASSNASDESMIETRCVLNSHS
BBU37472.1   .....GDRVADVIESSIGDSVSR.ALTHALPAPTGQNTQVSSHRLDTGKVPALQAAEIGASSNASDESMIETRCVLNSHS
QWY16108.1   .....GDRVADVIESSIGDSVSR.ALTHALPAPTGQNTLVSSHRLDTGKVPALQAAEIGASSNASDESMIETRCVLNSHS
QWY16042.1   .....GDRVADVIESSIGDSVSR.ALTHAFPAPTGQNTQVSSHRLDTGKVPALQAAEIGASSNASDESMIETRCVLNSHS
QOP74910.1   .....GDRVADVIESSIGDSVSR.ALTHALPAPTGQNTQVSSHRLDTGKVPALQAAEIGASSNASDESMIETRCVLNSHS
QWY16128.1   .....GDRVADVIESSIGDSVSR.ALTHALPAPTGQNTQVSSHRLDTGKVPALQAAEIGASSNASDESMIETRCVLNSHS
QUS47905.1   .....GDRVADVIESSIGDSVSR.ALTQALPAPTGQNTQVSSHRLDTGKVPALQAAEIGASSNASDESMIETRCVLNSHS
QWY16154.1   .....GDRVADVIESSIGDSVSR.ALTHALPAPTGQNTQVSSHRLDTGKVPALQAAEIGASSNASDESMIETRCVLNSHS
QWY16126.1   .....GDRVADVIESSIGDSVSR.ALTHALPAPTGQNTQVSSHRLDTGKVPALQAAEIGASSNASDESMIETRCVLNSHS
QWY16049.1   .....GDRVADVIESSIGDSVSR.ALTHALPAPTGQNTQVSSHRLDTGKVPALQAAEIGASSNASDESMIETRCVLNSHS
QWY16043.1   .....GDRVPDVIESSIGDSVSR.ALTHALSAPTGQNTQVSSHRLDTGKVPALQAAEIGASSNASDESMIETRCVLNSHS
QUS47911.1   .....GDRVADVIESSIGDSVSR.ALTHALPAPTGQNTQVSSHRLDTGKVPALQAAEIGASSNASDESMIETRCVLNSHS
BBU37477.1   .....GDRVADVIESSIGDSVSR.ALTHALPAPTGQNTQVSSHRLDTGKVPALQAAEIGASSNASDESMIETRCVLNSHS
QWY16137.1   .....GDRVADVIESSIGDSVSR.ALTHALPAPTGQNTQVSSHRLDTGKVPALQAAEIGASSNASDESMIETRCVLNSHS
QWY16061.1   .....GDRVADVIESSIGDSVSR.ALTHALPAPTGQNTQVSSHRLDTGKVPALQAAEIGASSNASDESMIETRCVLNSHS
QTA73882.1   .....GDRVADVIESSIGDSVSR.ALTHALPAPTGQNTQVSSHRLDTGKVPALQAAEIGASSNASDESMIETRCVLNSHS
QWY16031.1   .....GDRVADVIESSIGDSVSR.ALTHALPAPTGQNTQVSSHRLDTGKVPALQAAEIGASSNASDESMIETRCVLNSHS
BBU37476.1   .....GDRVADVIESSIGDSVSR.ALTHALPAPTGQNTQVSSHRLDTGKVPALQAAEIGASSNASDESMIETRCVLNSHS
QUS47949.1   .....GDRVADVIESSIGDSVSR.ALTHALPAPTGQNTQVSSHRLDTGKVPALQAAEIGASSNASDESMIETRCVLNSHS
QNN25833.1   .....GDRVADVIESSIGDSVSR.ALTHALPAPTGQNTQVSSHRLDTGKVPALQAAEIGASSNASDESMIETRCVLNAHS
QWY16114.1   .....GDRVADVIESSIGDSVSR.ALTHALPAPTGQNTQVSSHRLDTGKVPALQAAEIGASSNASDESMIETRCVLNSHS
QWY16063.1   .....GDRVADVIESSIGDSVSR.ALTHALPAPTGQNTQVSSHRLGTGKVPALQAAEIGASSNASDESMIETRCVLNSHS
QTA73884.1   .....GDRVADVIESSIGDSVSR.ALTHALPAPTGQNTQVSSHRLGTGKVPALQAAEIGASSNASDESMIETRCVLNSHS
QWY16002.1   .....GDRVADVIESSIGDSVSR.ALTHALPAPTGQNTQVSSHRLGTGKVPALQAAEIGASSNASDESMIETRCVLNSHS
QWY15984.1   .....GDRVADVIESSIGDSVSR.ALTHALPAPTGQNTQVSSHRLDTGKVPALQAAEIGASSNASDENMIETRCVLNSHS
QUS47934.1   .....GDRVADVIESSIGDSVSR.ALTHALPAPTGQNTQVSSHRLDTGKVPALQAAEIGASSNASDESMIETRCVLNSHS
QUS47933.1   .....GDRVADVIESSIGDSVSR.ALTHALPAPTGQNTQVSSHRLDTGKVPALQAAEIGASSNASDESMIETRCVLNSHS
QUS47929.1   .....GDRVADVIESSIGDSVSR.ALTHALPAPTGQNTQVSSHRLDTGKVPALQAAEIGASSNASDESMIETRCVLNSHS
QUS47927.1   .....GDRVADVIESSIGDSVSR.ALTHALPAPTGQNTQVSSHRLDTGKVPALQAAEIGASSNASDESMIETRCVLNSHS
QUS47919.1   .....GDRVADVIESSIGDSVSR.ALTHALPAPTGQNTQVSSHRLDTGKVPALQAAEIGASSNASDESMIETRCVLNSHS
QUS47917.1   .....GDRVADVIESSIGDSVSR.ALTHALPAPTGQNTQVSSHRLDTGKVPALQAAEIGASSNASDESMIETRCVLNSHS
QUS47915.1   .....GDRVADVIESSIGDSVSR.ALTHALPAPTGQNTQVSSHRLDTGKVPALQAAEIGASSNASDESMIETRCVLNSHS
QUS47913.1   .....GDRVADVIESSIGDSVSR.ALTHALPAPTGQNTQVSSHRLDTGKVPALQAAEIGASSNASDESMIETRCVLNSHS
QWY16166.1   .....GDRVADVIESSIGDSVSR.ALTHALPAPTGQNTQVSSHRLDTGKVPALQAAEIGASSNASDESMIETRCVLNSHS
QWY16164.1   .....GDRVADVIESSIGDSVSR.ALTHALPAPTGQNTQVSSHRLDTGKVPALQAAEIGASSNASDESMIETRCVLNSHS
QWY16163.1   .....GDRVADVIESSIGDSVSR.ALTHALPAPTGQNTQVSSHRLDTGKVPALQAAEIGASSNASDESMIETRCVLNSHS
QWY16157.1   .....GDRVADVIESSIGDSVSR.ALTHALPAPTGQNTQVSSHRLDTGKVPALQAAEIGASSNASDESMIETRCVLNSHS
QWY16150.1   .....GDRVADVIESSIGDSVSR.ALTHALPAPTGQNTQVSSHRLDTGKVPALQAAEIGASSNASDESMIETRCVLNSHS
QWY16115.1   .....GDRVADVIESSIGDSVSR.ALTHALPAPTGQNTQVSSHRLDTGKVPALQAAEIGASSNASDESMIETRCVLNSHS
QWY16087.1   .....GDRVADVIESSIGDSVSR.ALTHALPAPTGQNTQVSSHRLDTGKVPALQAAEIGASSNASDESMIETRCVLNSHS
QWY16069.1   .....GDRVADVIESSIGDSVSR.ALTHALPAPTGQNTQVSSHRLDTGKVPALQAAEIGASSNASDESMIETRCVLNSHS
QWY16058.1   .....GDRVADVIESSIGDSVSR.ALTHALPAPTGQNTQVSSHRLDTGKVPALQAAEIGASSNASDESMIETRCVLNSHS
QWY16048.1   .....GDRVADVIESSIGDSVSR.ALTHALPAPTGQNTQVSSHRLDTGKVPALQAAEIGASSNASDESMIETRCVLNSHS
QWY16047.1   .....GDRVADVIESSIGDSVSR.ALTHALPAPTGQNTQVSSHRLDTGKVPALQAAEIGASSNASDESMIETRCVLNSHS
QWY16030.1   .....GDRVADVIESSIGDSVSR.ALTHALPAPTGQNTQVSSHRLDTGKVPALQAAEIGASSNASDESMIETRCVLNSHS
QWY16027.1   .....GDRVADVIESSIGDSVSR.ALTHALPAPTGQNTQVSSHRLDTGKVPALQAAEIGASSNASDESMIETRCVLNSHS
QWY16021.1   .....GDRVADVIESSIGDSVSR.ALTHALPAPTGQNTQVSSHRLDTGKVPALQAAEIGASSNASDESMIETRCVLNSHS
QWY16020.1   .....GDRVADVIESSIGDSVSR.ALTHALPAPTGQNTQVSSHRLDTGKVPALQAAEIGASSNASDESMIETRCVLNSHS
QWY16009.1   .....GDRVADVIESSIGDSVSR.ALTHALPAPTGQNTQVSSHRLDTGKVPALQAAEIGASSNASDESMIETRCVLNSHS
QWY16008.1   .....GDRVADVIESSIGDSVSR.ALTHALPAPTGQNTQVSSHRLDTGKVPALQAAEIGASSNASDESMIETRCVLNSHS
QWY16007.1   .....GDRVADVIESSIGDSVSR.ALTHALPAPTGQNTQVSSHRLDTGKVPALQAAEIGASSNASDESMIETRCVLNSHS
QWY16001.1   .....GDRVADVIESSIGDSVSR.ALTHALPAPTGQNTQVSSHRLDTGKVPALQAAEIGASSNASDESMIETRCVLNSHS
QWY16000.1   .....GDRVADVIESSIGDSVSR.ALTHALPAPTGQNTQVSSHRLDTGKVPALQAAEIGASSNASDESMIETRCVLNSHS
QWY15993.1   .....GDRVADVIESSIGDSVSR.ALTHALPAPTGQNTQVSSHRLDTGKVPALQAAEIGASSNASDESMIETRCVLNSHS
QWY15986.1   .....GDRVADVIESSIGDSVSR.ALTHALPAPTGQNTQVSSHRLDTGKVPALQAAEIGASSNASDESMIETRCVLNSHS
QWY15979.1   .....GDRVADVIESSIGDSVSR.ALTHALPAPTGQNTQVSSHRLDTGKVPALQAAEIGASSNASDESMIETRCVLNSHS
QTA73889.1   .....GDRVADVIESSIGDSVSR.ALTHALPAPTGQNTQVSSHRLDTGKVPALQAAEIGASSNASDESMIETRCVLNSHS
QTA73874.1   .....GDRVADVIESSIGDSVSR.ALTHALPAPTGQNTQVSSHRLDTGKVPALQAAEIGASSNASDESMIETRCVLNSHS
QTA73871.1   .....GDRVADVIESSIGDSVSR.ALTHALPAPTGQNTQVSSHRLDTGKVPALQAAEIGASSNASDESMIETRCVLNSHS
QOP74901.1   .....GDRVADVIESSIGDSVSR.ALTHALPAPTGQNTQVSSHRLDTGKVPALQAAEIGASSNASDESMIETRCVLNSHS
QOP74900.1   .....GDRVADVIESSIGDSVSR.ALTHALPAPTGQNTQVSSHRLDTGKVPALQAAEIGASSNASDESMIETRCVLNSHS
QOP74897.1   .....GDRVADVIESSIGDSVSR.ALTHALPAPTGQNTQVSSHRLDTGKVPALQAAEIGASSNASDESMIETRCVLNSHS
QOP74893.1   .....GDRVADVIESSIGDSVSR.ALTHALPAPTGQNTQVSSHRLDTGKVPALQAAEIGASSNASDESMIETRCVLNSHS
QOP74887.1   .....GDRVADVIESSIGDSVSR.ALTHALPAPTGQNTQVSSHRLDTGKVPALQAAEIGASSNASDESMIETRCVLNSHS
QOP74886.1   .....GDRVADVIESSIGDSVSR.ALTHALPAPTGQNTQVSSHRLDTGKVPALQAAEIGASSNASDESMIETRCVLNSHS
QOP74884.1   .....GDRVADVIESSIGDSVSR.ALTHALPAPTGQNTQVSSHRLDTGKVPALQAAEIGASSNASDESMIETRCVLNSHS
QOP74882.1   .....GDRVADVIESSIGDSVSR.ALTHALPAPTGQNTQVSSHRLDTGKVPALQAAEIGASSNASDESMIETRCVLNSHS
QOP74881.1   .....GDRVADVIESSIGDSVSR.ALTHALPAPTGQNTQVSSHRLDTGKVPALQAAEIGASSNASDESMIETRCVLNSHS
QOP74880.1   .....GDRVADVIESSIGDSVSR.ALTHALPAPTGQNTQVSSHRLDTGKVPALQAAEIGASSNASDESMIETRCVLNSHS
QOP74879.1   .....GDRVADVIESSIGDSVSR.ALTHALPAPTGQNTQVSSHRLDTGKVPALQAAEIGASSNASDESMIETRCVLNSHS
QOP74878.1   .....GDRVADVIESSIGDSVSR.ALTHALPAPTGQNTQVSSHRLDTGKVPALQAAEIGASSNASDESMIETRCVLNSHS
pdb|4YVS|M   .....GDRVADVIESSIGDSVSR.ALTHALPAPTGQNTQVSSHRLDTGKVPALQAAEIGASSNASDESMIETRCVLNSHS
pdb|4YVS|J   .....GDRVADVIESSIGDSVSR.ALTHALPAPTGQNTQVSSHRLDTGKVPALQAAEIGASSNASDESMIETRCVLNSHS
pdb|4YVS|G   .....GDRVADVIESSIGDSVSR.ALTHALPAPTGQNTQVSSHRLDTGKVPALQAAEIGASSNASDESMIETRCVLNSHS
pdb|4YVS|D   .....GDRVADVIESSIGDSVSR.ALTHALPAPTGQNTQVSSHRLDTGKVPALQAAEIGASSNASDESMIETRCVLNSHS
pdb|4YVS|A   .....GDRVADVIESSIGDSVSR.ALTHALPAPTGQNTQVSSHRLDTGKVPALQAAEIGASSNASDESMIETRCVLNSHS
pdb|4CEY|A   .....GDRVADVIESSIGDSVSR.ALTHALPAPTGQNTQVSSHRLDTGKVPALQAAEIGASSNASDESMIETRCVLNSHS
pdb|4CEW|A   .....GDRVADVIESSIGDSVSR.ALTHALPAPTGQNTQVSSHRLDTGKVPALQAAEIGASSNASDESMIETRCVLNSHS
pdb|4N53|A   .....GDRVADVIESSIGDSVSR.ALTHALPAPTGQNTQVSSHRLDTGKVPALQAAEIGASSNASDESMIETRCVLNSHS
pdb|4N43|A   .....GDRVADVIESSIGDSVSR.ALTHALPAPTGQNTQVSSHRLDTGKVPALQAAEIGASSNASDESMIETRCVLNSHS
pdb|4RQP|A   .....GDRVADVIESSIGDSVSR.ALTHALPAPTGQNTQVSSHRLDTGKVPALQAAEIGASSNASDESMIETRCVLNSHS
pdb|4RQP|M   .....GDRVADVIESSIGDSVSR.ALTHALPAPTGQNTQVSSHRLDTGKVPALQAAEIGASSNASDESMIETRCVLNSHS
pdb|4RQP|I   .....GDRVADVIESSIGDSVSR.ALTHALPAPTGQNTQVSSHRLDTGKVPALQAAEIGASSNASDESMIETRCVLNSHS
pdb|4RQP|Q   .....GDRVADVIESSIGDSVSR.ALTHALPAPTGQNTQVSSHRLDTGKVPALQAAEIGASSNASDESMIETRCVLNSHS
pdb|4RQP|E   .....GDRVADVIESSIGDSVSR.ALTHALPAPTGQNTQVSSHRLDTGKVPALQAAEIGASSNASDESMIETRCVLNSHS
QWY16033.1   .....GDRVADVIESSIGDSVSR.ALTHALPAPTGQNTQVSSHRLDTGKVPALQAAEIGASSNASDESMIETRCVLNSHS
QWY16029.1   .....GDRVADVIESSIGDSVSR.ALTHALPAPTGQNTQVSSHRLDTGKVPALQAAEIGASSNASDESMIETRCVLNSHS
QWY16028.1   .....GDRVADVIESSIGDSVSR.ALTHALPAPTGQNTQVSSHRLDTGKVPALQAAEIGASSNASDESMIETRCVLNSHS
QWY16025.1   .....GDRVADVIESSIGDSVSR.ALTHALPAPTGQNTQVSSHRLDTGKVPALQAAEIGASSNASDESMIETRCVLNSHS
QUS47943.1   .....GDRVADVIESSIGDSVSR.ALTHALPAPTGQNTQVSSHRLDTGKVPALQAAEIGASSNASDESMIETRCVLNSHS
QOP74914.1   .....GDRVADVIESSIGDSVSR.ALTHALPAPTGQNTQVSSHRLDTGKVPALQAAEIGASSNASDESMIETRCVLNSHS
QUS47916.1   .....GDRVADVIESSIGDSVSR.ALTHALPAPTGQNTQVSSHRLDTGKVPALQAAEIGASSNASDESMIETRCVLNSHS
QWY16148.1   .....GDRVADVIESSIGDSVSR.ALTHALPAPTGQNTQVSSHRLDTGKVPALQAAEIGASSNASDESMIETRCVLNSHS
QOP74906.1   .....GDRVADVIESSIGDSVSR.ALTHALPAPTGQNTQVSSHRLDTGKVPALQAAEIGASSNASDESMIETRCVLNSHS
QOP74895.1   .....GDRVADVIESSIGDSVSR.ALTHALPAPTGQNTQVSSHRLDTGKVPALQAAEIGASSNASDESMIETRCVLNSHS
QOP74890.1   .....GDRVADVIESSIGDSVSR.ALTHALPAPTGQNTQVSSHRLDTGKVPALQAAEIGASSNASDESMIETRCVLNSHS
QOP74889.1   .....GDRVADVIESSIGDSVSR.ALTHALPAPTGQNTQVSSHRLDTGKVPALQAAEIGASSNASDESMIETRCVLNSHS
QWY16149.1   .....GDRVADVIESSIGDSVSR.ALTHALPAPTGQNTQVSSHRLDTGKVPALQAAEIGASSNASDESMIETRCVLNSHS
QOP74888.1   .....GDRVADVIESSIGDSVSR.ALTHALPAPTGQNTQVSSLRLDTGKVPALQAAEIGASSNASDESMIETRCVLNSHS
QOP74894.1   .....GDRVADVIESSIGDSVSR.ALTHALPAPTGQNTQVSSHRLDTGKVPALQAAEIGASSNASDESMIETRCVLNSHS
QUS47930.1   .....GDRVADVIESSIGDSVSR.ALTRALPAPTGQNTQVSSHRLDTGKVPALQAAEIGASSNASDESMIETRCVLNSHS
QWY16053.1   .....GDRVADVIESSIGDSVSR.ALTRALPAPTGQNTQVSSHRLDTGKVPALQAAEIGASSNASDESMIETRCVLNSHS
QWY15997.1   .....GDRVADVIESSIGDSVSR.ALTRALPAPTGQNTQVSSHRLDTGKVPALQAAEIGASSNASDESMIETRCVLNSHS
QWY15996.1   .....GDRVADVIESSIGDSVSR.ALTRALPAPTGQNTQVSSHRLDTGKVPALQAAEIGASSNASDESMIETRCVLNSHS
QWY15995.1   .....GDRVADVIESSIGDSVSR.ALTRALPAPTGQNTQVSSHRLDTGKVPALQAAEIGASSNASDESMIETRCVLNSHS
QTA73878.1   .....GDRVADVIESSIGDSVSR.ALTRALPAPTGQNTQVSSHRLDTGKVPALQAAEIGASSNASDESMIETRCVLNSHS
QTA73877.1   .....GDRVADVIESSIGDSVSR.ALTRALPAPTGQNTQVSSHRLDTGKVPALQAAEIGASSNASDESMIETRCVLNSHS
QTA73876.1   .....GDRVADVIESSIGDSVSR.ALTRALPAPTGQNTQVSSHRLDTGKVPALQAAEIGASSNASDESMIETRCVLNSHS
QUS47914.1   .....GDRVADVIESSIGDSVSR.ALTHALPAPTGQNTQVSSHRLDTGKVPALQAAEIGASSNASDESMIETRCVLNSHS
QWY16003.1   .....GDRVADVIESSIGDSVSR.ALTHALPAPTGQNTQVSSHRLDTGKVPALQAAEIGASSNASDESMIETRCVLNSHS
QWY16067.1   .....GDRVADVIESSIGDSVSR.ALTHALPAPTGQNTQVSSHRLDTGKVPALQAAEIGASSNASDESMIETRCVLNLHS
QWY16057.1   .....GDRVADVIESSIGDSVSR.ALTHALPAPTGQNTQVSSHRLDTGKVPALQAAEIGASSNASDESMIETRCVLNLHS
QTA73888.1   .....GDRVADVIESSIGDSVSR.ALTHALPAPTGQNTQVSSHRLDTGKVPALQAAEIGASSNASDESMIETRCVLNLHS
QWY16086.1   .....GDRVADVIESSIGDSVSR.ALTHALSAPTGQNTQVSSHRLDTGKVPALQAAEIGASSNASDESMIETRCVLNSHS
pdb|4GMP|1   .....GDRVADVIESSIGDSVSR.ALTRALPAPTGQNTQVSSHRLDTGKVPALQAAEIGASSNASDESMIETRCVLNSHS
pdb|3J91|1   .....GDRVADVIESSIGDSVSR.ALTRALPAPTGQNTQVSSHRLDTGKVPALQAAEIGASSNASDESMIETRCVLNSHS
QWY16094.1   .....GDRVADVIESSIGDSVSR.ALTHALSAPTGQNTQVSSHRLDTGKVPALQAAEIGASSNASDESMIETRCVLNSHS
QWY16093.1   .....GDRVADVIESSIGDSVSR.ALTHALSAPTGQNTQVSSHRLDTGKVPALQAAEIGASSNASDESMIETRCVLNSHS
QWY16091.1   .....GDRVADVIESSIGDSVSR.ALTHALSAPTGQNTQVSSHRLDTGKVPALQAAEIGASSNASDESMIETRCVLNSHS
QWY16088.1   .....GDRVADVIESSIGDSVSR.ALTHALSAPTGQNTQVSSHRLDTGKVPALQAAEIGASSNASDESMIETRCVLNSHS
QWY16082.1   .....GDRVADVIESSIGDSVSR.ALTHALSAPTGQNTQVSSHRLDTGKVPALQAAEIGASSNASDESMIETRCVLNSHS
QWY16073.1   .....GDRVADVIESSIGDSVSR.ALTHALSAPTGQNTQVSSHRLDTGKVPALQAAEIGASSNASDESMIETRCVLNSHS
QWY16055.1   .....GDRVADVIESSIGDSVSR.ALTHALSAPTGQNTQVSSHRLDTGKVPALQAAEIGASSNASDESMIETRCVLNSHS
QUS47918.1   .....GDRVADVIESSIGDSVGR.ALTHALPAPTGQNTQVSSHRLDTGKVPALQAAEIGASSNASDESMIETRCVLNSHS
QWY16089.1   .....GDRVADVIESSIGDSVSR.ALTHALPAPTGQNTQVSSHRLDTGKVPALQAAEIGASSNASDESMIETRCVLNSHS
QWY16037.1   .....GDRVADVIESSIGDSVSR.ALTHALPAPTGQNTQVSSHRLDTGKVPALQAAEIGASSNASDESMIETRCVLNSHS
QOP74891.1   .....GDRVADVIESSIGDSVSR.ALTHALPAPTGQNTQVSSHRLDTGKVPALQAAEIGASSNASDESMIETRCVLNSHS
pdb|3VBS|A   .....GDRVADVIESSIGDSVSR.ALTHALPAPTGQNTQVSSHRLDTGKVPALQAAEIGASSNASDESMIETRCVLNSHS
pdb|3VBO|A   .....GDRVADVIESSIGDSVSR.ALTHALPAPTGQNTQVSSHRLDTGKVPALQAAEIGASSNASDESMIETRCVLNSHS
pdb|3VBH|A   .....GDRVADVIESSIGDSVSR.ALTHALPAPTGQNTQVSSHRLDTGKVPALQAAEIGASSNASDESMIETRCVLNSHS
pdb|3VBF|A   .....GDRVADVIESSIGDSVSR.ALTHALPAPTGQNTQVSSHRLDTGKVPALQAAEIGASSNASDESMIETRCVLNSHS
pdb|6LQD|A   .....GDRVADVIESSIGDSVSR.ALTHALPAPTGQNTQVSSHRLDTGKVPALQAAEIGASSNASDESMIETRCVLNSHS
pdb|5ZUF|A   .....GDRVADVIESSIEDSSSR.ALTHALPAPTGQNTQVSSHRLDTGKVPALQAAEIGASSNASDESMIETRCVLNSHS
pdb|5ZUD|A   .....GDRVADVIESSIEDSSSR.ALTHALPAPTGQNTQVSSHRLDTGKVPALQAAEIGASSNASDESMIETRCVLNSHS
pdb|4CDX|A   .....GDRVADVIESSIGDSVSR.ALTHALPAPTGQNTQVSSHRLDTGKVPALQAAEIGASSNASDESMIETRCVLNSHS
pdb|4CDW|A   .....GDRVADVIESSIGDSVSR.ALTHALPAPTGQNTQVSSHRLDTGKVPALQAAEIGASSNASDESMIETRCVLNSHS
pdb|4CDU|A   .....GDRVADVIESSIGDSVSR.ALTHALPAPTGQNTQVSSHRLDTGKVPALQAAEIGASSNASDESMIETRCVLNSHS
pdb|4CDQ|A   .....GDRVADVIESSIGDSVSR.ALTHALPAPTGQNTQVSSHRLDTGKVPALQAAEIGASSNASDESMIETRCVLNSHS
```

```
QOP74885.1    .....GDRVADVIESSIGDSVSR.ALTHALPAPTGQNTQVSSHRLDTGKVPALQAAEIGASSNASDESMIETRCVLNSHS
QWY16026.1    .....GDRVADVIESSIGDSVSR.ALTHALPAPTGQNTQVSSHRLDTGKVPALQAAEIGASSNASDESMIETRCVLNSHS
QWY15977.1    .....GDRVADVIESSIGDSVSR.ALTHALPAPTGQNTQVSSHRLDTGKVPALQAAEIGASSNASDESMIETRCVLNSHS
QTA73869.1    .....GDRVADVIESSIGDSVSR.ALTHALPAPTGQNTQVSSHRLDTGKVPALQAAEIGASSNASDESMIETRCVLNSHS
QUS47935.1    .....GDRVADVIESSIGDSVSR.ALTHALPAPTGQNTQVSSHRLDTGKVPALQAAEIGASSNASDESMIETRCVLNSHS
QWY16024.1    .....GDRVADVIESSIGDSVSR.ALTHALPAPTGQNTQVSSHRLDTGKVPALQAAEIGASSNASDESMIETRCVLNSHS
QWY16023.1    .....GDRVADVIESSIGDSVSR.ALTHALPAPTGQNTQVSSHRLDTGKVPALQAAEIGASSNASDESMIETRCVLNSHS
QWY16022.1    .....GDRVADVIESSIGDSVSR.ALTHALPAPTGQNTQVSSHRLDTGKVPALQAAEIGASSNASDESMIETRCVLNSHS
QWY16013.1    .....GDRVADVIESSIGDSVSR.ALTHALPAPTGQNTQVSSHRLDTGKVPALQAAEIGASSNASDESMIETRCVLNSHS
QWY16012.1    .....GDRVADVIESSIGDSVSR.ALTHALPAPTGQNTQVSSHRLDTGKVPALQAAEIGASSNASDESMIETRCVLNSHS
QWY15992.1    .....GDRVADVIESSIGDSVSR.ALTHALPAPTGQNTQVSSHRLDTGKVPALQAAEIGASSNASDESMIETRCVLNSHS
QWY15982.1    .....GDRVADVIESSIGDSVSR.ALTHALPAPTGQNTQVSSHRLDTGKVPALQAAEIGASSNASDESMIETRCVLNSHS
QWY15981.1    .....GDRVADVIESSIGDSVSR.ALTHALPAPTGQNTQVSSHRLDTGKVPALQAAEIGASSNASDESMIETRCVLNSHS
QWY15976.1    .....GDRVADVIESSIGDSVSR.ALTHALPAPTGQNTQVSSHRLDTGKVPALQAAEIGASSNASDESMIETRCVLNSHS
QTA73880.1    .....GDRVADVIESSIGDSVSR.ALTHALPAPTGQNTQVSSHRLDTGKVPALQAAEIGASSNASDESMIETRCVLNSHS
QTA73872.1    .....GDRVADVIESSIGDSVSR.ALTHALPAPTGQNTQVSSHRLDTGKVPALQAAEIGASSNASDESMIETRCVLNSHS
QTA73868.1    .....GDRVADVIESSIGDSVSR.ALTHALPAPTGQNTQVSSHRLDTGKVPALQAAEIGASSNASDESMIETRCVLNSHS
QOP74892.1    .....GDRVADVIESSIGDSVSR.ALTHALPAPTGQNTQVSSHRLDTGKVPALQAAEIGASSNASDESMIETRCVLNSHS
QWY15998.1    .....GDRVADVIESSIGDSVSR.ALTHALPAPTGQNTQVSSHRLDTGKVPALQAAEIGASSNASDESMIETRCVLNLHS
QWY15994.1    .....GDRVADVIESSIGDSVSR.ALTHALPAPTGQNTQVSSHRLDTGKVPALQAAEIGASSNASDESMIETRCVLNLHS
QTA73879.1    .....GDRVADVIESSIGDSVSR.ALTHALPAPTGQNTQVSSHRLDTGKVPALQAAEIGASSNASDESMIETRCVLNLHS
QTA73875.1    .....GDRVADVIESSIGDSVSR.ALTHALPAPTGQNTQVSSHRLDTGKVPALQAAEIGASSNASDESMIETRCVLNLHS
QWY16006.1    .....GDRVADVIESSIGDSVSR.ALTHALPAPTGQNTQVSSHRLDTGKVPALQAAEIGASSNASDESMIETRCVLNSHS
QWY16005.1    .....GDRVADVIESSIGDSVSR.ALTHALPAPTGQNTQVSSHRLDTGKVPALQAAEIGASSNASDESMIETRCVLNSHS
QWY16039.1    .....GDRVADVIESSIGDSVSR.ALTHALPAPTGQNTQVSSHRLDTGKVPALQAAEIGASSNASDESMIETRCVLNSHS
QWY15999.1    .....GDRVADVIESSIGDSVSR.ALTHALPAPTGQNTQVSSHRLDTGKVPALQAAEIGASSNASDESMIETRCVLNSHS
QWY16004.1    .....GDRVADVIESSIGDSVSR.ALTHALPAPTGQNTQVSSHRLDTGKVPALQAAEIGASSNASDESMIETRCVLNSHS
QOP74913.1    .....GDRVADVIESSIGDSVSR.ALTHALPAPTGQNTQVSSHRLDTGKVPALQAAEIGASSNASDESMIETRCVLNSHS
QOP74915.1    .....GDRVADVIESSIGDSVSR.ALTHALPAPTGQNTQVSSHRLDXGKVPALQAAEIGASSNASDESMIETRCVLNSHS
QWY16035.1    .....GDRVADVIESSIGDSVSR.ALTHALPAPTGQNTQVSSHRLDTGKVPALQAAEIGASSNASDESMIETRCVLNSHS
QWY15990.1    .....GDRVADVIESSIGDSVSR.ALTHALPAPTGQNTQVSSHRLDTGKVPALQAAEIGASSNASDESMIETRCVLNSHS
QWY15988.1    .....GDRVADVIESSIGDSVSR.ALTHALPAPTGQNTQVSSHRLDTGKVPALQAAEIGASSNASDESMIETRCVLNSHS
QWY15989.1    .....GDRVADVIESSIGDSVSR.ALTHALPAPTGQNTQVSSHRLDTGKVPALQAAEIGASSNASDESMIETRCVLNSHS
QWY15987.1    .....GDRVADVIESSIGDSVSR.ALTHALPAPTGQNTQVSSHRLDTGKVPALQAAEIGASSNASDESMIETRCVLNSHS
QWY15978.1    .....GDRVADVIESSIGDSVSR.ALTHALPAPTGQNTQVSSHRLDTGKVPALQAAEIGASSNASDESMIETRCVLNSHS
QTA73870.1    .....GDRVADVIESSIGDSVSR.ALTHALPAPTGQNTQVSSHRLDTGKVPALQAAEIGASSNASDESMIETRCVLNSHS
QWY15983.1    .....GDRVADVIESSIGDSVSR.ALTHALPAPTGQNTQVSSHRLDTGKVPALQAAEIGASSNASDESMIETRCVLNSHS
QUS47931.1    .....GDRVADVIESSIGDSVSR.ALTHALPAPTGQDTQVSSHRLDTGKVPALQAAEIGASSNASDESMIETRCVLNSHS
QUS47925.1    .....GDRVADVIESSIGDSVSR.ALTHALPAPTGQDTQVSSHRLDTGKVPALQAAEIGASSNASDESMIETRCVLNSHS
QUS47922.1    .....GDRVADVIESSIGDSVSR.ALTHALPAPTGQDTQVSSHRLDTGKVPALQAAEIGASSNASDESMIETRCVLNSHS
QWY16014.1    .....GDRVADVIESSIGDSVSR.ALTHALPAPTGQDTQVSSHRLDTGKVPALQAAEIGASSNASDESMIETRCVLNSHS
QTA73881.1    .....GDRVADVIESSIGDSVSR.ALTHALPAPTGQDTQVSSHRLDTGKVPALQAAEIGASSNASDESMIETRCVLNSHS
QOP74898.1    .....GDRVADVIESSIGDSVSR.ALTHALPAPTGQDTQVSSHRLDTGKVPALQAAEIGASSNASDESMIETRCVLNSHS
QWY15980.1    .....GDRVPDVIESSIGDSVSR.ALTHALPAPTGQDTQVSSHRLDTGKVPALQAAEIGASSNASDESMIETRCVLNSHS
QTA73873.1    .....GDRVPDVIESSIGDSVSR.ALTHALPAPTGQDTQVSSHRLDTGKVPALQAAEIGASSNASDESMIETRCVLNSHS
QWY16011.1    .....GDRVADVIESSIGDSVSR.ALTHALPAPTGQDTQVSSHRLDTGKVPALQAAEIGASSNASDESMIETRCVLNSHS
QWY16010.1    .....GDRVADVIESSIGDSVSR.ALTHALPAPTGQDTQVSSHRLDTGKVPALQAAEIGASSNASDESMIETRCVLNSHS
QOP74911.1    .....GDRVADVIESSIGDSVSR.ALTHALPAPTGQDTQVSSHRLDTGKVPALQAAEIGASSNASDESMIETRCVLNSHS
QWY16019.1    .....GDRVADVIESSIGDSVSR.ALTHALPAPTGQDTQVSSHRLDTGKVPALQAAEIGASSNASDESMIETRCVLNSHS
QOP74883.1    .....GDRVADVIESSIGDSVSR.ALTHALPAPTGQDTQVSSHRLDTGKVPALQAAEIGASSNASDESMIETRCVLNSHS
QUS47909.1    .....GDRVADVIESSIGDSVSR.ALTHALPAPTGQNTQVSSHRLDTGKVPALQAAEIGASSNASDESMIETRCVLNSHS
QWY16144.1    .....GDRVADVIESSIGDSVSR.ALTRALPAPTGQNTQVSSHRLDTGKVPALQAAEIGASSNASDESMIETRCVLNSHS
QUS47908.1    .....GDRVADVIESSIGDSVSR.ALTHALPAPTGQNTQVSSHRLDTGKVPALQAAEIGASSNASDESMIETRCVLNSHS
QNN25844.1    .....GDRVADVIESSIGDSMSR.ALTQALPAPTGQNTQVSSHRLDTGKVPALQAAEIGASSNASDESMIETRCVLNSHS
QNN25840.1    .....GDRVADVIESSIGDSMSR.ALTQALPAPTGQNTQVSSHRLDTGKVPALQAAEIGASSNASDESMIETRCVLNSHS
QNN25839.1    .....GDRVADVIESSIGDSMSR.ALTQALPAPTGQNTQVSSHRLDTGKVPALQAAEIGASSNASDESMIETRCVLNSHS
QNN25835.1    .....GDRVADVIESSIGDSMSR.ALTQALPAPTGQNTQVSSHRLDTGKVPALQAAEIGASSNASDESMIETRCVLNSHS
QNN25834.1    .....GDRVADVIESSIGDSMSR.ALTQALPAPTGQNTQVSSHRLDTGKVPALQAAEIGASSNASDESMIETRCVLNSHS
QOP74912.1    .....GDRVADVIESSIGDSVSR.ALTQALPAPTGQNTQVSSHRLDTGKVPALQAAEIGASSNASDESMIETRCVLNSHS
QUS47910.1    .....GDRVADVIESSIGDSVSR.ALTRALPAPTGQNTQVSSHRLDTGKVPALQAAEIGASSNASDESMIETRCVLNSHS
QUS47907.1    .....GDRVADVIESSIGDSVSR.ALTHALPAPTGQNTQVSSHRLDTGKVPALQAAEIGASSNASDESMIETRCVLNSHS
QWY16141.1    .....GDKVADVIESSIGDSVSR.ALTHALPAPTGQNTQASSHRLDTGKVPALQAAEIGASSNASDESMIETRCVLNSHS
QWY16140.1    .....GDKVADVIESSIGDSVSR.ALTHALSAPTGQNTQASSHRLDTGKVPALQAAEIGASSNASDESMIETRCVLNSHS
QUS47937.1    .....GDRVADVIESTIGDSVSR.ALTHALPAPTGQNTQVSSHRLDTGKVPALQAAEIGASSNASDESMIETRCVLNSHS
QUS47936.1    .....GDRVADVIESSIGDSVSR.ALTHALPAPTGQNTQVSSHRLDTGKVPALQAAEIGASSNASDESMIETRCVLNSHS
QUS47912.1    .....GDRVADVIESSIGDSVSR.ALTHALPAPTGQNTQVSSHQADAERVPVVQAAETGASSNDSDESMIETRCVLNSHS
QUS47906.1    .....GDRVADVIESSIGDSVSR.ALTHALPAPTGQNTQVSSHRLDTGKVPALQAAETGASSNASDESMIETRCVLNSHS
pdb|4YVW|M    .....GDRVADVIESSIGDSVSR.ALTHALPAPTGQNTQVSSHRLDTGKVPALQAAEIGASSNASDESMIETRCVLNSHS
pdb|4YVW|J    .....GDRVADVIESSIGDSVSR.ALTHALPAPTGQNTQVSSHRLDTGKVPALQAAEIGASSNASDESMIETRCVLNSHS
pdb|4YVW|D    .....GDRVADVIESSIGDSVSR.ALTHALPAPTGQNTQVSSHRLDTGKVPALQAAEIGASSNASDESMIETRCVLNSHS
pdb|4YVW|A    .....GDRVADVIESSIGDSVSR.ALTHALPAPTGQNTQVSSHRLDTGKVPALQAAEIGASSNASDESMIETRCVLNSHS
pdb|4YVW|E    .....GDRVADVIESSIGDSVSR.ALTHALPAPTGQNTQVSSHRLDTGKVPALQAAEIGASSNASDESMIETRCVLNSHS
QPI19797.1    .....GDRVADVIESSIGDSVSR.ALTQALPAPTGQNTQVSSHRLDTGEVPALQAAEIGASSNTSDESMIETRCVLNSHS
QPI19781.1    .....GDRVADVIESSIGDSVSR.ALTQALPAPTGQNTQVSSHRLDTGEVLALQAAEIGASSNTSDESMIETRCVLNSHS
QPI19795.1    .....GDRVADVIESSIGDSVSR.ALTQALPAPTGQNTQVSSHRLDTGEVPALQAAEIGASSNTSDESMIETRCVLNSHS
QPI19794.1    .....GDRVADVIESSIGDSVSR.ALTQALPAPTGQNTQVSSHRLDTGEVPALQAAEIGASSNTSDESMIETRCVLNSHS
QPI19793.1    .....GDRVADVIESSIGDSVSR.ALTQALPAPTGQNTQVSSHRLDTGEVPALQAAEIGASSNTSDESMIETRCVLNSHS
QPI19792.1    .....GDRVADVIESSIGDSVSR.ALTQALPAPTGQNTQVSSHRLDTGEVPALQAAEIGASSNTSDESMIETRCVLNSHS
QPI19790.1    .....GDRVADVIESSIGDSVSR.ALTQALPAPTGQNTQVSSHRLDTGEVPALQAAEIGASSNTSDESMIETRCVLNSHS
QPI19789.1    .....GDRVADVIESSIGDSVSR.ALTQALPAPTGQNTQVSSHRLDTGEVPALQAAEIGASSNTSDESMIETRCVLNSHS
QPI19788.1    .....GDRVADVIESSIGDSVSR.ALTQALPAPTGQNTQVSSHRLDTGEVPALQAAEIGASSNTSDESMIETRCVLNSHS
QPI19787.1    .....GDRVADVIESSIGDSVSR.ALTQALPAPTGQNTQVSSHRLDTGEVPALQAAEIGASSNTSDESMIETRCVLNSHS
QPI19785.1    .....GDRVADVIESSIGDSVSR.ALTQALPAPTGQNTQVSSHRLDTGEVPALQAAEIGASSNTSDESMIETRCVLNSHS
QPI19784.1    .....GDRVADVIESSIGDSVSR.ALTQALPAPTGQNTQVSSHRLDTGEVPALQAAEIGASSNTSDESMIETRCVLNSHS
QPI19782.1    .....GDRVADVIESSIGDSVSR.ALTQALPAPTGQNTQVSSHRLDTGEVPALQAAEIGASSNTSDESMIETRCVLNSHS
QPI19780.1    .....GDRVADVIESSIGDSVSR.ALTQALPAPTGQNTQVSSHRLDTGEVPALQAAEIGASSNTSDESMIETRCVLNSHS
QPI19777.1    .....GDRVADVIESSIGDSVSR.ALTQALPAPTGQNTQVSSHRLDTGEVPALQAAEIGASSNTSDESMIETRCVLNSHS
QPI19762.1    .....GDRVADVIESSIGDSVSR.ALTQALPAPTGQNTQVSSHRLDTGEVPALQAAEIGASSNTSDESMIETRCVLNSHS
QPI19755.1    .....GDRVADVIESSIGDSVSR.ALTQALPAPTGQNTQVSSHRLDTGEVPALQAAEIGASSNTSDESMIETRCVLNSHS
QPI19754.1    .....GDRVADVIESSIGDSVSR.ALTQALPAPTGQNTQVSSHRLDTGEVPALQAAEIGASSNTSDESMIETRCVLNSHS
QPI19748.1    .....GDRVADVIESSIGDSVSR.ALTQALPAPTGQNTQVSSHRLDTGEVPALQAAEIGASSNTSDESMIETRCVLNSHS
QPI19747.1    .....GDRVADVIESSIGDSVSR.ALTQALPAPTGQNTQVSSHRLDTGEVPALQAAEIGASSNTSDESMIETRCVLNSHS
QPI19744.1    .....GDRVADVIESSIGDSVSR.ALTQALPAPTGQNTQVSSHRLDTGEVPALQAAEIGASSNTSDESMIETRCVLNSHS
QPI19743.1    .....GDRVADVIESSIGDSVSR.ALTQALPAPTGQNTQVSSHRLDTGEVPALQAAEIGASSNTSDESMIETRCVLNSHS
QPI19739.1    .....GDRVADVIESSIGDSVSR.ALTQALPAPTGQNTQVSSHRLDTGEVPALQAAEIGASSNTSDESMIETRCVLNSHS
QPI19778.1    .....GDRVADVIESSIGDSVSR.ALTQALPAPTGQNTQVSSHRLDTGEVPALQAAEIGASSNTSDESMIETRCVLNSHS
QPI19796.1    .....GDRVADVIESSIGDSVSR.ALTQALPAPTGQNTQVSSHRLDTGEVPALQAAEIGASSNTSDESMIETRCVLNSHS
QPI19753.1    .....GDRVADVIESSIGDSVSR.ALTQALPAPTGLNTQVSSHRLDTGEVPALQAAEIGASSNTSDESMIETRCVLNSHS
QPI19791.1    .....GDRVADVIESSIGDSVSR.ALTQALPAPTGQNTQVSSHRLDTGEVPALQAAEIGASSNTSDESMIETRCVLNSHS
QPI19786.1    .....GDRVADVIESSIGDSVSR.ALTQAPPAPTGQNTQVSSHRLDTGEVPALQAAEIGASSNTSDESMIETRCVLNSHS
QPI19779.1    .....GDRVADVIESSIGDSVSR.ALTQALPAPTGQNTQMSSHRLDTGEVPALQAAEIRASSNTSDESMIETRCVLNSHS
QPI19742.1    .....GDRVADVIESSIGDSVSR.ALTQALPAPTGQNTQVSSHRLDTGEVPALQAAEIGASSNTSDESMIETRCVLNSHS
pdb|4AED|A    .....GDRVADVIESSIGDSVSR.ALTQALPAPTGQNTQVSSHRLDTGEVPALQAAEIGASSNTSDESMIETRCVLNSHS
pdb|3ZFF|A    .....GDRVADVIESSIGDSVSR.ALTQALPAPTGQNTQVSSHRLDTGEVPALQAAEIGASSNTSDESMIETRCVLNSHS
pdb|6DIZ|A    .....GDRVADVIESSIGDSVSR.ALTQALPAPTGQNTQVSSHRLDTGEVPALQAAEIGASSNTSDESMIETRCVLNSHS
pdb|6UH7|A    .....GDRVADVIESSIGDSVSR.ALTQALPAPTGQNTQVSSHRLDTGEVPALQAAEIGASSNTSDESMIETRCVLNSHS
pdb|6UH6|A    .....GDRVADVIESSIGDSVSR.ALTQALPAPTGQNTQVSSHRLDTGEVPALQAAEIGASSNTSDESMIETRCVLNSHS
pdb|6UH1|A    .....GDRVADVIESSIGDSVSR.ALTQALPAPTGQNTQVSSHRLDTGEVPALQAAEIGASSNTSDESMIETRCVLNSHS
QPI19783.1    .....GDRVADVIESSIGDSVSR.ALTQALPAPTGQNTQVSSHRLDTGEVPALQAAEIGASSNTSDESMIETRCVLNSHS
QPI19772.1    .....GDRVADVIESSIGDSVSR.ALTQALPAPTGQNTQVSSHRLDTGEVPALQAAEIGASSNTSDESMIETRCVLNSHS
QPI19771.1    .....GDRVADVIESSIGDSVSR.ALTQALPAPTGQNTQVSSHRLDTGEVPALQAAEIGASSNTSDESMIETRCVLNSHS
QPI19764.1    .....GDRVADVIESSIGDSVSR.ALTQALPAPTGQNTQVSSHRLDTGEVPALQAAEIGASSNTSDESMIETRCVLNSHS
QPI19760.1    .....GDRVADVIESSIGDSVSR.ALTQALPAPTGQNTQVSSHRLDTGEVPALQAAEIGASSNTSDESMIETRCVLNSHS
QPI19759.1    .....GDRVADVIESSIGDSVSR.ALTQALPAPTGQNTQVSSHRLDTGEVPALQAAEIGASSNTSDESMIETRCVLNSHS
QPI19756.1    .....GDRVADVIESSIGDSVSR.ALTQALPAPTGQNTQVSSHRLDTGEVPALQAAEIGASSNTSDESMIETRCVLNSHS
QPI19752.1    .....GDRVADVIESSIGDSVSR.ALTQALPAPTGQNTQVSSHRLDTGEVPALQAAEIGASSNTSDESMIETRCVLNSHS
QPI19740.1    .....GDRVADVIESSIGDSVSR.ALTQALPAPTGQNTQVSSHRLDTGEVPALQAAEIGASSNTSDESMIETRCVLNSHS
QPI19738.1    .....GDRVADVIESSIGDSVSR.ALTQALPAPTGQNTQVSSHRLDTGEVPALQAAEIGASSNTSDESMIETRCVLNSHS
QPI19757.1    .....GDRVADVIESSIGDSVSR.ALTQALPAPTGQNTQVSSHRLDTGEVPALQAAEIGASSNTSDESMIETRCVLNSHS
QPI19750.1    .....GDRVADVIESSIGDSVSR.ALTQALPAPTGQNTQVSSHRLDTGEVPALQAAEIGASSNTSDESMIETRCVLNSHS
QPI19751.1    .....GDRVADVIESSIGDSVSR.ALTQALPAPTGQNTQVSSHRLDTGEVPALQAAEIGASSNTSDESMIETRCVLNSHS
QPI19746.1    .....GDRVADVIESSIGDSVSR.ALTQALPAPTGQNTQVSSHRLDTGEVPALQAAEIGASSNTSDESMIETRCVLNSHS
QPI19737.1    .....GDRVADVIESSIGDSVSR.ALTQALPAPTGQNTQVSSHRLDTGEVPALQAAEIGASSNTSDESMIETRCVLNSHS
QPI19776.1    .....GDRVADVIESSIGDSVSR.ALTQALPAPTGQNTQVSSHRLDTGEVPALQAAEIGASSNTSDESMIETRCVLNSHS
QPI19774.1    .....GDRVADVIESSIGDSVSR.ALTQALPAPTGQNTQVSSHRLDTGEVPALQAAEIGASSNTSDESMIETRCVLNSHS
QPI19773.1    .....GDRVADVIESSIGDSVSR.ALTQALPAPTGQNTQVSSHRLDTGEVPALQAAEIGASSNTSDESMIETRCVLNSHS
QPI19770.1    .....GDRVADVIESSIGDSVSR.ALTQALPAPTGQNTQVSSHRLDTGEVPALQAAEIGASSNTSDESMIETRCVLNSHS
QPI19769.1    .....GDRVADVIESSIGDSVSR.ALTQALPAPTGQNTQVSSHRLDTGEVPALQAAEIGASSNTSDESMIETRCVLNSHS
QPI19768.1    .....GDRVADVIESSIGDSVSR.ALTQALPAPTGQNTQVSSHRLDTGEVPALQAAEIGASSNTSDESMIETRCVLNSHS
QPI19767.1    .....GDRVADVIESSIGDSVSR.ALTQALPAPTGQNTQVSSHRLDTGEVPALQAAEIGASSNTSDESMIETRCVLNSHS
QPI19766.1    .....GDRVADVIESSIGDSVSR.ALTQALPAPTGQNTQVSSHRLDTGEVPALQAAEIGASSNTSDESMIETRCVLNSHS
QPI19763.1    .....GDRVADVIESSIGDSVSR.ALTQALPAPTGQNTQVSSHRLDTGEVPALQAAEIGASSNTSDESMIETRCVLNSHS
QPI19761.1    .....GDRVADVIESSIGDSVSR.ALTQALPAPTGQNTQVSSHRLDTGEVPALQAAEIGASSNTSDESMIETRCVLNSHS
QPI19765.1    .....GDRVADVIESSIGDSVSR.ALTQALPAPTGQNTQVSSHRLDTGEVPALQAAEIGASSNTSDESMIETRCVLNSHS
QPI19775.1    .....GDRVADVIESSIGDSVSR.ALTQALPAPTGQNTQVSSHRLDTGEVPALQAAEIGASSNTSDESMIETRCVLNSHS
QPI19758.1    .....GDRVADVIESSIGDSVSR.ALTQALPAPTGQNTQVSSHRLDTGEVPALQAAEIGASSNTSDESMIETRCVLNSHS
QPI19736.1    .....GDRVADVIESSIGDSVSR.ALTQALPAPTGQNTQVSSHRLDTGEVPALQAAEIGASSNTSDESMIETRCVLNSHS
QPI19735.1    .....GDRVADVIESSIGDSVSR.ALTQALPAPTGQNTQVSSHRLDTGEVPALQAAEIGASSNTSDESMIETRCVLNSHS
QPI19749.1    .....GDRAADVIESSIGDSVSR.ALTQALPAPTGQNTQVSSHRLDTGEVPALQAAEIGASSNTSDESMIETRCVLNSHS
QPI19741.1    .....GDRVADVIESSIGDSVSR.ALTQALPAPTGQNTQVSSHRLDTGEVPALQAAEIGASSNTSDESMIETRCVLNSHS
QPI19745.1    .....GDRVADVIESSIGDSVSR.TLTQGLPAPTGQNTQVSSHRLDTGEVPALQAAEIGASSNTSDESMIETRCVLNSHS
pdb|6Z3Q|A    .....GDRVADMIESSIGNSVSR.ALTQALPAPTGQNTQVSSHRLDTGEVPALQAAEIGASSNTSDESMIETRCVLNSHS
pdb|6Z3P|A    .....GDRVADMIESSIGNSVSR.ALTQALPAPTGQNTQVSSHRLDTGEVPALQAAEIGASSNTSDESMIETRCVLNSHS
pdb|6I2K|A    .....GDRVADMIESSIGDSVSR.ALTQALPAPTGQNTQVSSHRLDTGEVPALQAAEIGASSNTSDESMIETRCVLNSHS
```

QWF08740.1	.....GDPIADMIDQTVNNQVNR.SLTAMQVLPTAANTEASSHRLGTGVVPALQAAETGASSNASDKNLIETRCVLNHHS
QWF08739.1	.....GDPIADMIDQTVNNQVNR.SLTAMQVLPTAANTEASSHRLGTGVVPALQAAETGASSNASDKNLIETRCVLNHHS
QWF08760.1	.....GDPIADMIDQTVNNQVNR.SLTAMQVLPTAANTEASSHRLGTGVVPALQAAETGASSNASDKNLIETRCVLNHHS
QWF08759.1	.....GDPIADMIDQTVNNQVNR.SLTAMQVLPTAANTEASSHRLGTGVVPALQAAETGASSNASDKNLIETRCVLNHHS
QWF08762.1	.....GDPIADMIDQTVNNQVNR.SLTAMQVLPTAANTEASSHRLGTGVVPALQAAETGASSNASDKNLIETRCVLNHHS
QWF08761.1	.....GDPIADMIDQTVNNQVNR.SLTAMQVLPTAANTEASSHRLGTGVVPALQAAETGASSNASDKNLIETRCVLNHHS
QWF08764.1	.....GDPIADMIDQTVNNQVNR.SLTAMQVLPTAANTEASSHRLGTGVVPALQAAETGASSNASDKNLIETRCVLNHHS
QWF08763.1	.....GDPIADMIDQTVNNQVNR.SLTAMQVLPTAANTEASSHRLGTGVVPALQAAETGASSNASDKNLIETRCVLNHHS
QWF08773.1	.....GDPIADMIDQTVNNQVNR.SLTAMQVLPTAANTEASSHRLGTGVVPALQAAETGASSNASDKNLIETRCVLNHHS
QWF08749.1	.....GDPIADMIDQTVNNQVNR.SLTAMQVLPTAANTEASSHRLGTGVVPALQAAETGASSNASDKNLIETRCVLNHHS
QWF08785.1	.....GDPIADMIDQTVNNQVNR.SLTAMQVLPTAANTEASSHRLGTGVVPALQAAETGASSNASDKNLIETRCVLNHHS
QWF08783.1	.....GDPIADMIDQTVNNQVNR.SLTAMQVLPTAANTEASSHRLGTGVVPALQAAETGASSNASDKNLIETRCVLNHHS
QWF08787.1	.....GDPIADMIDQTVNNQVNR.SLTAMQVLPTAANTEASSHRLGTGVVPALQAAETGASSNASDKNLIETRCVLNHHS
QWF08765.1	.....GDPIADMIDQTVNNQVNR.SLTAMQVLPTAANTEASSHRLGTGVVPALQAAETGASSNASDKNLIETRCVLNHHS
QWF08792.1	.....GDPIADMIDQTVNNQVNR.SLTAMQVLPTAANTEASSHRLGTGVVPALQAAETGASSNASDKNLIETRCVLNHHS
QWF08767.1	.....GDPIADMIDQTVNNQVNR.SLTAMQVLPTAANTEASSHRLGTGVVPALQAAETGASSNASDKNLIETRCVLNHHS
QWF08793.1	.....GDPIADMIDQTVNNQVNR.SLTAMQVLPTAANTEASSHRLGTGVVPALQAAETGASSNASDKNLIETRCVLNHHS
QWF08758.1	.....GDPIADMIDQTVNNQVNR.SLTAMQVLPTAANTEASSHRLGTGVVPALQAAETGASSNASDKNLIETRCVLNHHS
QWF08845.1	.....GDPIADMIDQTVNNQVNR.SLTAMQVLPTAANTEASSHRLGTGVVPALQAAETGASSNASDKNLIETRCVLNHHS
QWF08844.1	.....GDPIADMIDQTVNNQVNR.SLTAMQVLPTAANTEASSHRLGTGVVPALQAAETGASSNASDKNLIETRCVLNHHS
QWF08847.1	.....GDPIADMIDQTVNNQVNR.SLTAMQVLPTAANTEASSHRLGTGVVPALQAAETGASSNASDKNLIETRCVLNHHS
QWF08846.1	.....GDPIADMIDQTVNNQVNR.SLTAMQVLPTAANTEASSHRLGTGVVPALQAAETGASSNASDKNLIETRCVLNHHS
QWF08852.1	.....GDPIADMIDQTVNNQVNR.SLTAMQVLPTAANTEASSHRLGTGVVPALQAAETGASSNASDKNLIETRCVLNHHS
QWF08843.1	.....GDPIADMIDQTVNNQVNR.SLTAMQVLPTAANTEASSHRLGTGVVPALQAAETGASSNASDKNLIETRCVLNHHS
QWF08857.1	.....GDPIADMIDQTVNNQVNR.SLTAMQVLPTAANTEASSHRLGTGVVPALQAAETGASSNASDKNLIETRCVLNHHS
QWF08856.1	.....GDPIADMIDQTVNNQVNR.SLTAMQVLPTAANTEASSHRLGTGVVPALQAAETGASSNASDKNLIETRCVLNHHS
QWF08865.1	.....GDPIADMIDQTVNNQVNR.SLTAMQVLPTAANTEASSHRLGTGVVPALQAAETGASSNASDKNLIETRCVLNHHS
QWF08738.1	.....GDPIADMIDQTVNNQVNR.SLTAMQVLPTAANTEASSHRLGTGVVPALQAAETGASSNASDKNLIETRCVLNHHS
QWF08872.1	.....GDPIADMIDQTVNNQVNR.SLTAMQVLPTAANTEASSHRLGTGVVPALQAAETGASSNASDKNLIETRCVLNHHS
QWF08871.1	.....GDPIADMIDQTVNNQVNR.SLTAMQVLPTAANTEASSHRLGTGVVPALQAAETGASSNASDKNLIETRCVLNHHS
QWF08879.1	.....GDPIADMIDQTVNNQVNR.SLTAMQVLPTAANTEASSHRLGTGVVPALQAAETGASSNASDKNLIETRCVLNHHS
QWF08877.1	.....GDPIADMIDQTVNNQVNR.SLTAMQVLPTAANTEASSHRLGTGVVPALQAAETGASSNASDKNLIETRCVLNHHS
QWF08880.1	.....GDPIADMIDQTVNNQVNR.SLTAMQVLPTAANTEASSHRLGTGVVPALQAAETGASSNASDKNLIETRCVLNHHS
QWF08851.1	.....GDPIADMIDQTVNNQVNR.SLTAMQVLPTAANTEASSHRLGTGVVPALQAAETGASSNASDKNLIETRCVLNHHS
QWF08882.1	.....GDPIADMIDQTVNNQVNR.SLTAMQVLPTAANTEASSHRLGTGVVPALQAAETGASSNASDKNLIETRCVLNHHS
QWF08867.1	.....GDPIADMIDQTVNNQVNR.SLTAMQVLPTAANTEASSHRLGTGVVPALQAAETGASSNASDKNLIETRCVLNHHS
QWF08884.1	.....GDPIADMIDQTVNNQVNR.SLTAMQVLPTAANTEASSHRLGTGVVPALQAAETGASSNASDKNLIETRCVLNHHS
QWF08875.1	.....GDPIADMIDQTVNNQVNR.SLTAMQVLPTAANTEASSHRLGTGVVPALQAAETGASSNASDKNLIETRCVLNHHS
QWF08885.1	.....GDPIADMIDQTVNNQVNR.SLTAMQVLPTAANTEASSHRLGTGVVPALQAAETGASSNASDKNLIETRCVLNHHS
QWF08863.1	.....GDPIADMIDQTVNNQVNR.SLTAMQVLPTAANTEASSHRLGTGVVPALQAAETGASSNASDKNLIETRCVLNHHS
QWF08890.1	.....GDPIADMIDQTVNNQVNR.SLTAMQVLPTAANTEASSHRLGTGVVPALQAAETGASSNASDKNLIETRCVLNHHS
QWF08889.1	.....GDPIADMIDQTVNNQVNR.SLTAMQVLPTAANTEASSHRLGTGVVPALQAAETGASSNASDKNLIETRCVLNHHS
QWF08896.1	.....GDPIADMIDQTVNNQVNR.SLTAMQVLPTAANTEASSHRLGTGVVPALQAAETGASSNASDKNLIETRCVLNHHS
QWF08752.1	.....GDPIADMIDQTVNNQVNR.SLTAMQVLPTAANTEASSHRLGTGVVPALQAAETGASSNASDKNLIETRCVLNHHS
QWF08900.1	.....GDPIADMIDQTVNNQVNR.SLTAMQVLPTAANTEASSHRLGTGVVPALQAAETGASSNASDKNLIETRCVLNHHS
QWF08899.1	.....GDPIADMIDQTVNNQVNR.SLTAMQVLPTAANTEASSHRLGTGVVPALQAAETGASSNASDKNLIETRCVLNHHS
QWF08908.1	.....GDPIADMIDQTVNNQVNR.SLTAMQVLPTAANTEASSHRLGTGVVPALQAAETGASSNASDKNLIETRCVLNHHS
QWF08906.1	.....GDPIADMIDQTVNNQVNR.SLTAMQVLPTAANTEASSHRLGTGVVPALQAAETGASSNASDKNLIETRCVLNHHS
QWF08910.1	.....GDPIADMIDQTVNNQVNR.SLTAMQVLPTAANTEASSHRLGTGVVPALQAAETGASSNASDKNLIETRCVLNHHS
QWF08870.1	.....GDPIADMIDQTVNNQVNR.SLTAMQVLPTAANTEASSHRLGTGVVPALQAAETGASSNASDKNLIETRCVLNHHS
QWF08915.1	.....GDPIADMIDQTVNNQVNR.SLTAMQVLPTAANTEASSHRLGTGVVPALQAAETGASSNASDKNLIETRCVLNHHS
QWF08750.1	.....GDPIADMIDQTVNNQVNR.SLTAMQVLPTAANTEASSHRLGTGVVPALQAAETGASSNASDKNLIETRCVLNHHS
QWF08917.1	.....GDPIADMIDQTVNNQVNR.SLTAMQVLPTAANTEASSHRLGTGVVPALQAAETGASSNASDKNLIETRCVLNHHS
QWF08916.1	.....GDPIADMIDQTVNNQVNR.SLTAMQVLPTAANTEASSHRLGTGVVPALQAAETGASSNASDKNLIETRCVLNHHS
QWF08923.1	.....GDPIADMIDQTVNNQVNR.SLTAMQVLPTAANTEASSHRLGTGVVPALQAAETGASSNASDKNLIETRCVLNHHS
QWF08921.1	.....GDPIADMIDQTVNNQVNR.SLTAMQVLPTAANTEASSHRLGTGVVPALQAAETGASSNASDKNLIETRCVLNHHS
QWF08926.1	.....GDPIADMIDQTVNNQVNR.SLTAMQVLPTAANTEASSHRLGTGVVPALQAAETGASSNASDKNLIETRCVLNHHS
QWF08780.1	.....GDPIADMIDQTVNNQVNR.SLTAMQVLPTAANTEASSHRLGTGVVPALQAAETGASSNASDKNLIETRCVLNHHS
QWF08932.1	.....GDPIADMIDQTVNNQVNR.SLTAMQVLPTAANTEASSHRLGTGVVPALQAAETGASSNASDKNLIETRCVLNHHS
QWF08931.1	.....GDPIADMIDQTVNNQVNR.SLTAMQVLPTAANTEASSHRLGTGVVPALQAAETGASSNASDKNLIETRCVLNHHS
QWF08945.1	.....GDPIADMIDQTVNNQVNR.SLTAMQVLPTAANTEASSHRLGTGVVPALQAAETGASSNASDKNLIETRCVLNHHS
QWF08938.1	.....GDPIADMIDQTVNNQVNR.SLTAMQVLPTAANTEASSHRLGTGVVPALQAAETGASSNASDKNLIETRCVLNHHS
QWF08955.1	.....GDPIADMIDQTVNNQVNR.SLTAMQVLPTAANTEASSHRLGTGVVPALQAAETGASSNASDKNLIETRCVLNHHS
QWF08954.1	.....GDPIADMIDQTVNNQVNR.SLTAMQVLPTAANTEASSHRLGTGVVPALQAAETGASSNASDKNLIETRCVLNHHS
QWF08967.1	.....GDPIADMIDQTVNNQVNR.SLTAMQVLPTAANTEASSHRLGTGVVPALQAAETGASSNASDKNLIETRCVLNHHS
QWF08959.1	.....GDPIADMIDQTVNNQVNR.SLTAMQVLPTAANTEASSHRLGTGVVPALQAAETGASSNASDKNLIETRCVLNHHS
QYC65038.1	.....GDPIADMIDQTVNNQVNR.SLTAMQVLPTAANTEASSHRLGTGVVPALQAAETGASSNASDKNLIETRCVLNHHS
QYC65037.1	.....GDPIADMIDQTVNNQVNR.SLTAMQVLPTAANTEASSHRLGTGVVPALQAAETGASSNASDKNLIETRCVLNHHS
QYC65039.1	.....GDPIADMIDQTVNNQVNR.SLTAMQVLPTAANTEASSHRLGTGVVPALQAAETGASSNASDKNLIETRCVLNHHS
QYC65034.1	.....GDPIADMIDQTVNNQVNR.SLTAMQVLPTAANTEASSHRLGTGVVPALQAAETGASSNASDKNLIETRCVLNHHS
QYC65043.1	.....GDPIADMIDQTVNNQVNR.SLTAMQVLPTAANTEASSHRLGTGVVPALQAAETGASSNASDKNLIETRCVLNHHS
QYC65041.1	.....GDPIADMIDQTVNNQVNR.SLTAMQVLPTAANTEASSHRLGTGVVPALQAAETGASSNASDKNLIETRCVLNHHS
QYC65047.1	.....GDPIADMIDQTVNNQVNR.SLTAMQVLPTAANTEASSHRLGTGVVPALQAAETGASSNASDKNLIETRCVLNHHS
QYC65046.1	.....GDPIADMIDQTVNNQVNR.SLTAMQVLPTAANTEASSHRLGTGVVPALQAAETGASSNASDKNLIETRCVLNHHS
QYC65050.1	.....GDPIADMIDQTVNNQVNR.SLTAMQVLPTAANTEASSHRLGTGVVPALQAAETGASSNASDKNLIETRCVLNHHS
QYC65049.1	.....GDPIADMIDQTVNNQVNR.SLTAMQVLPTAANTEASSHRLGTGVVPALQAAETGASSNASDKNLIETRCVLNHHS
QYC65051.1	.....GDPIADMIDQTVNNQVNR.SLTAMQVLPTAANTEASSHRLGTGVVPALQAAETGASSNASDKNLIETRCVLNHHS
QYC65045.1	.....GDPIADMIDQTVNNQVNR.SLTAMQVLPTAANTEASSHRLGTGVVPALQAAETGASSNASDKNLIETRCVLNHHS
QYC65058.1	.....GDPIADMIDQTVNNQVNR.SLTAMQVLPTAANTEASSHRLGTGVVPALQAAETGASSNASDKNLIETRCVLNHHS
QYC65055.1	.....GDPIADMIDQTVNNQVNR.SLTAMQVLPTAANTEASSHRLGTGVVPALQAAETGASSNASDKNLIETRCVLNHHS
QYC65060.1	.....GDPIADMIDQTVNNQVNR.SLTAMQVLPTAANTEASSHRLGTGVVPALQAAETGASSNASDKNLIETRCVLNHHS
QYC65059.1	.....GDPIADMIDQTVNNQVNR.SLTAMQVLPTAANTEASSHRLGTGVVPALQAAETGASSNASDKNLIETRCVLNHHS
QYC65062.1	.....GDPIADMIDQTVNNQVNR.SLTAMQVLPTAANTEASSHRLGTGVVPALQAAETGASSNASDKNLIETRCVLNHHS
QYC65061.1	.....GDPIADMIDQTVNNQVNR.SLTAMQVLPTAANTEASSHRLGTGVVPALQAAETGASSNASDKNLIETRCVLNHHS
QYC65063.1	.....GDPIADMIDQTVNNQVNR.SLTAMQVLPTAANTEASSHRLGTGVVPALQAAETGASSNASDKNLIETRCVLNHHS
QYC65053.1	.....GDPIADMIDQTVNNQVNR.SLTAMQVLPTAANTEASSHRLGTGVVPALQAAETGASSNASDKNLIETRCVLNHHS
QYC65069.1	.....GDPIADMIDQTVNNQVNR.SLTAMQVLPTAANTEASSHRLGTGVVPALQAAETGASSNASDKNLIETRCVLNHHS
QYC65068.1	.....GDPIADMIDQTVNNQVNR.SLTAMQVLPTAANTEASSHRLGTGVVPALQAAETGASSNASDKNLIETRCVLNHHS
QYC65071.1	.....GDPIADMIDQTVNNQVNR.SLTAMQVLPTAANTEASSHRLGTGVVPALQAAETGASSNASDKNLIETRCVLNHHS
QYC65070.1	.....GDPIADMIDQTVNNQVNR.SLTAMQVLPTAANTEASSHRLGTGVVPALQAAETGASSNASDKNLIETRCVLNHHS
QYC65074.1	.....GDPIADMIDQTVNNQVNR.SLTAMQVLPTAANTEASSHRLGTGVVPALQAAETGASSNASDKNLIETRCVLNHHS
QYC65066.1	.....GDPIADMIDQTVNNQVNR.SLTAMQVLPTAANTEASSHRLGTGVVPALQAAETGASSNASDKNLIETRCVLNHHS
QYC65079.1	.....GDPIADMIDQTVNNQVNR.SLTAMQVLPTAANTEASSHRLGTGVVPALQAAETGASSNASDKNLIETRCVLNHHS
QYC65073.1	.....GDPIADMIDQTVNNQVNR.SLTAMQVLPTAANTEASSHRLGTGVVPALQAAETGASSNASDKNLIETRCVLNHHS
QYC65081.1	.....GDPIADMIDQTVNNQVNR.SLTAMQVLPTAANTEASSHRLGTGVVPALQAAETGASSNASDKNLIETRCVLNHHS
QYC65078.1	.....GDPIADMIDQTVNNQVNR.SLTAMQVLPTAANTEASSHRLGTGVVPALQAAETGASSNASDKNLIETRCVLNHHS
QYC65086.1	.....GDPIADMIDQTVNNQVNR.SLTAMQVLPTAANTEASSHRLGTGVVPALQAAETGASSNASDKNLIETRCVLNHHS
QYC65085.1	.....GDPIADMIDQTVNNQVNR.SLTAMQVLPTAANTEASSHRLGTGVVPALQAAETGASSNASDKNLIETRCVLNHHS
QYC65088.1	.....GDPIADMIDQTVNNQVNR.SLTAMQVLPTAANTEASSHRLGTGVVPALQAAETGASSNASDKNLIETRCVLNHHS
QYC65087.1	.....GDPIADMIDQTVNNQVNR.SLTAMQVLPTAANTEASSHRLGTGVVPALQAAETGASSNASDKNLIETRCVLNHHS
QYC65101.1	.....GDPIADMIDQTVNNQVNR.SLTAMQVLPTAANTEASSHRLGTGVVPALQAAETGASSNASDKNLIETRCVLNHHS
QYC65093.1	.....GDPIADMIDQTVNNQVNR.SLTAMQVLPTAANTEASSHRLGTGVVPALQAAETGASSNASDKNLIETRCVLNHHS
QYC65107.1	.....GDPIADMIDQTVNNQVNR.SLTAMQVLPTAANTEASSHRLGTGVVPALQAAETGASSNASDKNLIETRCVLNHHS
QYC65104.1	.....GDPIADMIDQTVNNQVNR.SLTAMQVLPTAANTEASSHRLGTGVVPALQAAETGASSNASDKNLIETRCVLNHHS
QYC65113.1	.....GDPIADMIDQTVNNQVNR.SLTAMQVLPTAANTEASSHRLGTGVVPALQAAETGASSNASDKNLIETRCVLNHHS
QYC65106.1	.....GDPIADMIDQTVNNQVNR.SLTAMQVLPTAANTEASSHRLGTGVVPALQAAETGASSNASDKNLIETRCVLNHHS
QYC65120.1	.....GDPIADMIDQTVNNQVNR.SLTAMQVLPTAANTEASSHRLGTGVVPALQAAETGASSNASDKNLIETRCVLNHHS
QYC65118.1	.....GDPIADMIDQTVNNQVNR.SLTAMQVLPTAANTEASSHRLGTGVVPALQAAETGASSNASDKNLIETRCVLNHHS
QYC65121.1	.....GDPIADMIDQTVNNQVNR.SLTAMQVLPTAANTEASSHRLGTGVVPALQAAETGASSNASDKNLIETRCVLNHHS
QYC65111.1	.....GDPIADMIDQTVNNQVNR.SLTAMQVLPTAANTEASSHRLGTGVVPALQAAETGASSNASDKNLIETRCVLNHHS
QYC65125.1	.....GDPIADMIDQTVNNQVNR.SLTAMQVLPTAANTEASSHRLGTGVVPALQAAETGASSNASDKNLIETRCVLNHHS
QYC65117.1	.....GDPIADMIDQTVNNQVNR.SLTAMQVLPTAANTEASSHRLGTGVVPALQAAETGASSNASDKNLIETRCVLNHHS
QYC65135.1	.....GDPIADMIDQTVNNQVNR.SLTAMQVLPTAANTEASSHRLGTGVVPALQAAETGASSNASDKNLIETRCVLNHHS
QYC65134.1	.....GDPIADMIDQTVNNQVNR.SLTAMQVLPTAANTEASSHRLGTGVVPALQAAETGASSNASDKNLIETRCVLNHHS
QYC65151.1	.....GDPIADMIDQTVNNQVNR.SLTAMQVLPTAANTEASSHRLGTGVVPALQAAETGASSNASDKNLIETRCVLNHHS
QYC65150.1	.....GDPIADMIDQTVNNQVNR.SLTAMQVLPTAANTEASSHRLGTGVVPALQAAETGASSNASDKNLIETRCVLNHHS
QYC65153.1	.....GDPIADMIDQTVNNQVNR.SLTAMQVLPTAANTEASSHRLGTGVVPALQAAETGASSNASDKNLIETRCVLNHHS
QYC65152.1	.....GDPIADMIDQTVNNQVNR.SLTAMQVLPTAANTEASSHRLGTGVVPALQAAETGASSNASDKNLIETRCVLNHHS
QYC65155.1	.....GDPIADMIDQTVNNQVNR.SLTAMQVLPTAANTEASSHRLGTGVVPALQAAETGASSNASDKNLIETRCVLNHHS
QYC65154.1	.....GDPIADMIDQTVNNQVNR.SLTAMQVLPTAANTEASSHRLGTGVVPALQAAETGASSNASDKNLIETRCVLNHHS
QYC65156.1	.....GDPIADMIDQTVNNQVNR.SLTAMQVLPTAANTEASSHRLGTGVVPALQAAETGASSNASDKNLIETRCVLNHHS
QYC65142.1	.....GDPIADMIDQTVNNQVNR.SLTAMQVLPTAANTEASSHRLGTGVVPALQAAETGASSNASDKNLIETRCVLNHHS
QYC65161.1	.....GDPIADMIDQTVNNQVNR.SLTAMQVLPTAANTEASSHRLGTGVVPALQAAETGASSNASDKNLIETRCVLNHHS
QYC65160.1	.....GDPIADMIDQTVNNQVNR.SLTAMQVLPTAANTEASSHRLGTGVVPALQAAETGASSNASDKNLIETRCVLNHHS
QYC65165.1	.....GDPIADMIDQTVNNQVNR.SLTAMQVLPTAANTEASSHRLGTGVVPALQAAETGASSNASDKNLIETRCVLNHHS
QYC65164.1	.....GDPIADMIDQTVNNQVNR.SLTAMQVLPTAANTEASSHRLGTGVVPALQAAETGASSNASDKNLIETRCVLNHHS
QYC65167.1	.....GDPIADMIDQTVNNQVNR.SLTAMQVLPTAANTEASSHRLGTGVVPALQAAETGASSNASDKNLIETRCVLNHHS
QYC65166.1	.....GDPIADMIDQTVNNQVNR.SLTAMQVLPTAANTEASSHRLGTGVVPALQAAETGASSNASDKNLIETRCVLNHHS
BCB92151.1	.....GDPIADMIDQTVNNQVNR.SLTAMQVLPTAANTEASSHRLGTGVVPALQAAETGASSNASDKNLIETRCVLNHHS
BBU37462.1	.....GDPIADMIDQTVNNQVNR.SLTAMQVLPTAANTEASSHRLGTGVVPALQAAETGASSNASDKNLIETRCVLNHHS
BBU37461.1	.....GDPIADMIDQTVNNQVNR.SLTAMQVLPTAANTEASSHRLGTGVVPALQAAETGASSNASDKNLIETRCVLNHHS
BBU37459.1	.....GDPIADMIDQTVNNQVNR.SLTAMQVLPTAANTEASSHRLGTGVVPALQAAETGASSNASDKNLIETRCVLNHHS
BBU37458.1	.....GDPIADMIDQTVNNQVNR.SLTAMQVLPTAANTEASSHRLGTGVVPALQAAETGASSNASDKNLIETRCVLNHHS
BBU37457.1	.....GDPIADMIDQTVNNQVNR.SLTAMQVLPTAANTEASSHRLGTGVVPALQAAETGASSNASDKNLIETRCVLNHHS
QWF08838.1	.....GDPIADMIDQTVNNQVNR.SLTAMQVLPTAANTEASSHRLGTGVVPALQAAETGASSNASDKNLIETRCVLNHHS
QWF08828.1	.....GDPIADMIDQTVNNQVNR.SLTAMQVLPTAANTEASSHRLGTGVVPALQAAETGASSNASDKNLIETRCVLNHHS
BCG66989.1	.....GDPIADMIDQTVNNQVNR.SLTAMQVLPTAANTEASSHRLGTGVVPALQAAETGASSNASDKNLIETRCVLNHHS
BCG66987.1	.....GDPIADMIDQTVNNQVNR.SLTAMQVLPTAANTEASSHRLGTGVVPALQAAETGASSNASDKNLIETRCVLNHHS
BCB92159.1	.....GDPIADMIDQTVNNQVNR.SLTAMQVLPTAANTEASSHRLGTGVVPALQAAETGASSNASDKNLIETRCVLNHHS
BBU37456.1	.....GDPIADMIDQTVNNQVNR.SLTAMQVLPTAANTEASSHRLGTGVVPALQAAETGASSNASDKNLIETRCVLNHHS
QWY15683.1	.....GDPIADMIDQTVNNQVNR.SLTAMQVLPTAANTEASSHRLGTGVVPALQAAETGASSNASDKNLIETRCVLNHHS
QWY15682.1	.....GDPIADMIDQTVNNQVNR.SLTAMQVLPTAANTEASSHRLGTGVVPALQAAETGASSNASDKNLIETRCVLNHHS
QWY15681.1	.....GDPIADMIDQTVNNQVNR.SLTAMQVLPTAANTEASSHRLGTGVVPALQAAETGASSNASDKNLIETRCVLNHHS
QWY15680.1	.....GDPIADMIDQTVNNQVNR.SLTAMQVLPTAANTEASSHRLGTGVVPALQAAETGASSNASDKNLIETRCVLNHHS

```
QWY15692.1     .....GDPIADMIDQTVNNQVNR.SLTAMQVLPTAANTEASSHRLGTGVVPALQAAETGASSNASDKNLIETRCVLNHHS
QWY15671.1     .....GDPIADMIDQTVNNQVNR.SLTAMQVLPTAANTEASSHRLGTGVVPALQAAETGASSNASDKNLIETRCVLNHHS
QWY15691.1     .....GDPIADMIDQTVNNQVNR.SLTAMQVLPTAANTEASSHRLGTGVVPALQAAETGASSNASDKNLIETRCVLNHHS
QWY15690.1     .....GDPIADMIDQTVNNQVNR.SLTAMQVLPTAANTEASSHRLGTGVVPALQAAETGASSNASDKNLIETRCVLNHHS
QWY15696.1     .....GDPIADMIDQTVNNQVNR.SLTAMQVLPTAANTEASSHRLGTGVVPALQAAETGASSNASDKNLIETRCVLNHHS
QWY15695.1     .....GDPIADMIDQTVNNQVNR.SLTAMQVLPTAANTEASSHRLGTGVVPALQAAETGASSNASDKNLIETRCVLNHHS
QWY15694.1     .....GDPIADMIDQTVNNQVNR.SLTAMQVLPTAANTEASSHRLGTGVVPALQAAETGASSNASDKNLIETRCVLNHHS
QWY15678.1     .....GDPIADMIDQTVNNQVNR.SLTAMQVLPTAANTEASSHRLGTGVVPALQAAETGASSNASDKNLIETRCVLNHHS
QWY15703.1     .....GDPIADMIDQTVNNQVNR.SLTAMQVLPTAANTEASSHRLGTGVVPALQAAETGASSNASDKNLIETRCVLNHHS
QWY15701.1     .....GDPIADMIDQTVNNQVNR.SLTAMQVLPTAANTEASSHRLGTGVVPALQAAETGASSNASDKNLIETRCVLNHHS
QWY15699.1     .....GDPIADMIDQTVNNQVNR.SLTAMQVLPTAANTEASSHRLGTGVVPALQAAETGASSNASDKNLIETRCVLNHHS
QWY15673.1     .....GDPIADMIDQTVNNQVNR.SLTAMQVLPTAANTEASSHRLGTGVVPALQAAETGASSNASDKNLIETRCVLNHHS
QWY15715.1     .....GDPIADMIDQTVNNQVNR.SLTAMQVLPTAANTEASSHRLGTGVVPALQAAETGASSNASDKNLIETRCVLNHHS
QWY15714.1     .....GDPIADMIDQTVNNQVNR.SLTAMQVLPTAANTEASSHRLGTGVVPALQAAETGASSNASDKNLIETRCVLNHHS
QWY15706.1     .....GDPIADMIDQTVNNQVNR.SLTAMQVLPTAANTEASSHRLGTGVVPALQAAETGASSNASDKNLIETRCVLNHHS
QWY15705.1     .....GDPIADMIDQTVNNQVNR.SLTAMQVLPTAANTEASSHRLGTGVVPALQAAETGASSNASDKNLIETRCVLNHHS
QTA73914.1     .....GDPIADMIDQTVNNQVNR.SLTAMQVLPTAANTEASSHRLGTGVVPALQAAETGASSNASDKNLIETRCVLNHHS
QWY15948.1     .....GDPIADMIDQTVNNQVNR.SLTAMQVLPTAANTEASSHRLGTGVVPALQAAETGASSNASDKNLIETRCVLNHHS
QWF08837.1     .....GDPIADMIDQTVNNQVNR.SLTAMQVLPTAANTEASSHRLGTGVVPALQAAETGASSNASDKNLIETRCVLNHHS
QWF08804.1     .....GDPIADMIDQTVNNQVNR.SLTAMQVLPTAANTEASSHRLGTGVVPALQAAETGASSNASDKNLIETRCVLNHHS
QWF08805.1     .....GDPIADMIDQTVNNQVNR.SLTAMQVLPTAANTEASSHRLGTGVVPALQAAETGASSNASDKNLIETRCVLNHHS
QWF08840.1     .....GDPIADMIDQTVNNQVNR.SLTAMQVLPTAANTEASSHRLGTGVVPALQAAETGASSNASDKNLIETRCVLNHHS
QWY15792.1     .....GDPIADMIDQTVNNQVNR.SLTAMQVLPTAANTEASSHRLGTGVVPALQAAETGASSNASDKNLIETRCVLNHHS
QTA73903.1     .....GDPIADMIDQTVNNQVNR.SLTAMQVLPTAANTEASSHRLGTGVVPALQAAETGASSNASDKNLIETRCVLNHHS
QTA73902.1     .....GDPIADMIDQTVNNQVNR.SLTAMQVLPTAANTEASSHRLGTGVVPALQAAETGASSNASDKNLIETRCVLNHHS
QTA73896.1     .....GDPIADMIDQTVNNQVNR.SLTAMQVLPTAANTEASSHRLGTGVVPALQAAETGASSNASDKNLIETRCVLNHHS
QTA73894.1     .....GDPIADMIDQTVNNQVNR.SLTAMQVLPTAANTEASSHRLGTGVVPALQAAETGASSNASDKNLIETRCVLNHHS
QWY15940.1     .....GDPIADMIDQTVNNQVNR.SLTAMQVLPTAANTEASSHRLGTGVVPALQAAETGASSNASDKNLIETRCVLNHHS
QWY15928.1     .....GDPIADMIDQTVNNQVNR.SLTAMQVLPTAANTEASSHRLGTGVVPALQAAETGASSNASDKNLIETRCVLNHHS
QWY15894.1     .....GDPIADMIDQTVNNQVNR.SLTAMQVLPTAANTEASSHRLGTGVVPALQAAETGASSNASDKNLIETRCVLNHHS
BCG67001.1     .....GDPIADMIDQTVNNQVNR.SLTAMQVLPTAANTEASSHRLGTGVVPALQAAETGASSNASDKNLIETRCVLNHHS
QWY15872.1     .....GDPIADMIDQTVNNQVNR.SLTAMQVLPTAANTEASSHRLGTGVVPALQAAETGASSNASDKNLIETRCVLNHHS
QWY15871.1     .....GDPIADMIDQTVNNQVNR.SLTAMQVLPTAANTEASSHRLGTGVVPALQAAETGASSNASDKNLIETRCVLNHHS
QWY15870.1     .....GDPIADMIDQTVNNQVNR.SLTAMQVLPTAANTEASSHRLGTGVVPALQAAETGASSNASDKNLIETRCVLNHHS
QWY15869.1     .....GDPIADMIDQTVNNQVNR.SLTAMQVLPTAANTEASSHRLGTGVVPALQAAETGASSNASDKNLIETRCVLNHHS
QWY15866.1     .....GDPIADMIDQTVNNQVNR.SLTAMQVLPTAANTEASSHRLGTGVVPALQAAETGASSNASDKNLIETRCVLNHHS
BCB92155.1     .....GDPIADMIDQTVNNQVNR.SLTAMQVLPTAANTEASSHRLGTGVVPALQAAETGASSNASDKNLIETRCVLNHHS
QWY15862.1     .....GDPIADMIDQTVNNQVNR.SLTAMQVLPTAANTEASSHRLGTGVVPALQAAETGASSNASDKNLIETRCVLNHHS
QWY15861.1     .....GDPIADMIDQTVNNQVNR.SLTAMQVLPTAANTEASSHRLGTGVVPALQAAETGASSNASDKNLIETRCVLNHHS
QWY15852.1     .....GDPIADMIDQTVNNQVNR.SLTAMQVLPTAANTEASSHRLGTGVVPALQAAETGASSNASDKNLIETRCVLNHHS
QWY15828.1     .....GDPIADMIDQTVNNQVNR.SLTAMQVLPTAANTEASSHRLGTGVVPALQAAETGASSNASDKNLIETRCVLNHHS
QWY15839.1     .....GDPIADMIDQTVNNQVNR.SLTAMQVLPTAANTEASSHRLGTGVVPALQAAETGASSNASDKNLIETRCVLNHHS
QWY15834.1     .....GDPIADMIDQTVNNQVNR.SLTAMQVLPTAANTEASSHRLGTGVVPALQAAETGASSNASDKNLIETRCVLNHHS
QWY15810.1     .....GDPIADMIDQTVNNQVNR.SLTAMQVLPTAANTEASSHRLGTGVVPALQAAETGASSNASDKNLIETRCVLNHHS
QWY15809.1     .....GDPIADMIDQTVNNQVNR.SLTAMQVLPTAANTEASSHRLGTGVVPALQAAETGASSNASDKNLIETRCVLNHHS
QWY15807.1     .....GDPIADMIDQTVNNQVNR.SLTAMQVLPTAANTEASSHRLGTGVVPALQAAETGASSNASDKNLIETRCVLNHHS
QWY15806.1     .....GDPIADMIDQTVNNQVNR.SLTAMQVLPTAANTEASSHRLGTGVVPALQAAETGASSNASDKNLIETRCVLNHHS
QWY15805.1     .....GDPIADMIDQTVNNQVNR.SLTAMQVLPTAANTEASSHRLGTGVVPALQAAETGASSNASDKNLIETRCVLNHHS
QWY15803.1     .....GDPIADMIDQTVNNQVNR.SLTAMQVLPTAANTEASSHRLGTGVVPALQAAETGASSNASDKNLIETRCVLNHHS
QWY15793.1     .....GDPIADMIDQTVNNQVNR.SLTAMQVLPTAANTEASSHRLGTGVVPALQAAETGASSNASDKNLIETRCVLNHHS
QYC65185.1     .....GDPIADMIDQTVNNQVNR.SLTAMQVLPTAANTEASSHRLGTGVVPALQAAETGASSNASDKNLIETRCVLNHHS
QWY15754.1     .....GDPIADMIDQTVNNQVNR.SLTAMQVLPTAANTEASSHRLGTGVVPALQAAETGASSNASDKNLIETRCVLNHHS
QWY15736.1     .....GDPIADMIDQTVNNQVNR.SLTAMQVLPTAANTEASSHRLGTGVVPALQAAETGASSNASDKNLIETRCVLNHHS
QWY15753.1     .....GDPIADMIDQTVNNQVNR.SLTAMQVLPTAANTEASSHRLGTGVVPALQAAETGASSNASDKNLIETRCVLNHHS
QYC65174.1     .....GDPIADMIDQTVNNQVNR.SLTAMQVLPTAANTEASSHRLGTGVVPALQAAETGASSNASDKNLIETRCVLNHHS
QWY15730.1     .....GDPIADMIDQTVNNQVNR.SLTAMQVLPTAANTEASSHRLGTGVVPALQAAETGASSNASDKNLIETRCVLNHHS
QWY15729.1     .....GDPIADMIDQTVNNQVNR.SLTAMQVLPTAANTEASSHRLGTGVVPALQAAETGASSNASDKNLIETRCVLNHHS
QWY15727.1     .....GDPIADMIDQTVNNQVNR.SLTAMQVLPTAANTEASSHRLGTGVVPALQAAETGASSNASDKNLIETRCVLNHHS
QWY15726.1     .....GDPIADMIDQTVNNQVNR.SLTAMQVLPTAANTEASSHRLGTGVVPALQAAETGASSNASDKNLIETRCVLNHHS
QWY15725.1     .....GDPIADMIDQTVNNQVNR.SLTAMQVLPTAANTEASSHRLGTGVVPALQAAETGASSNASDKNLIETRCVLNHHS
QWY15724.1     .....GDPIADMIDQTVNNQVNR.SLTAMQVLPTAANTEASSHRLGTGVVPALQAAETGASSNASDKNLIETRCVLNHHS
QWY15723.1     .....GDPIADMIDQTVNNQVNR.SLTAMQVLPTAANTEASSHRLGTGVVPALQAAETGASSNASDKNLIETRCVLNHHS
QWY15721.1     .....GDPIADMIDQTVNNQVNR.SLTAMQVLPTAANTEASSHRLGTGVVPALQAAETGASSNASDKNLIETRCVLNHHS
QWY15746.1     .....GDPIADMIDQTVNNQVNR.SLTAMQVLPTAANTEASSHRLGTGVVPALQAAETGASSNASDKNLIETRCVLNHHS
QWY15745.1     .....GDPIADMIDQTVNNQVNR.SLTAMQVLPTAANTEASSHRLGTGVVPALQAAETGASSNASDKNLIETRCVLNHHS
QWY15733.1     .....GDPIADMIDQTVNNQVNR.SLTAMQVLPTAANTEASSHRLGTGVVPALQAAETGASSNASDKNLIETRCVLNHHS
QYC65131.1     .....GDPIADMIDQTVNNQVNR.SLTAMQVLPTAANTEASSHRLGTGVVPALQAAETGASSNASDKNLIETRCVLNHHS
QWY15813.1     .....GDPIADMIDQTVNNQVNR.SLTAMQVLPTAANTEASSHRLGTGVVPALQAAETGASSNASDKNLIETRCVLNHHS
QWY15811.1     .....GDPIADMIDQTVNNQVNR.SLTAMQVLPTAANTEASSHRLGTGVVPALQAAETGASSNASDKNLIETRCVLNHHS
QTA73907.1     .....GDPIADMIDQTVNNQVNR.SLTAMQVLPTAANTEASSHRLGTGVVPALQAAETGASSNASDKNLIETRCVLNHHS
QTA73906.1     .....GDPIADMIDQTVNNQVNR.SLTAMQVLPTAANTEASSHRLGTGVVPALQAAETGASSNASDKNLIETRCVLNHHS
QOP74940.1     .....GDPIADMIDQTVNNQVNR.SLTAMQVLPTAANTEASSHRLGTGVVPALQAAETGASSNASDKNLIETRCVLNHHS
QOP74938.1     .....GDPIADMIDQTVNNQVNR.SLTAMQVLPTAANTEASSHRLGTGVVPALQAAETGASSNASDKNLIETRCVLNHHS
QOP74937.1     .....GDPIADMIDQTVNNQVNR.SLTAMQVLPTAANTEASSHRLGTGVVPALQAAETGASSNASDKNLIETRCVLNHHS
QOP74934.1     .....GDPIADMIDQTVNNQVNR.SLTAMQVLPTAANTEASSHRLGTGVVPALQAAETGASSNASDKNLIETRCVLNHHS
QOP74929.1     .....GDPIADMIDQTVNNQVNR.SLTAMQVLPTAANTEASSHRLGTGVVPALQAAETGASSNASDKNLIETRCVLNHHS
QOP74924.1     .....GDPIADMIDQTVNNQVNR.SLTAMQVLPTAANTEASSHRLGTGVVPALQAAETGASSNASDKNLIETRCVLNHHS
QOP74921.1     .....GDPIADMIDQTVNNQVNR.SLTAMQVLPTAANTEASSHRLGTGVVPALQAAETGASSNASDKNLIETRCVLNHHS
QOP74932.1     .....GDPIADMIDQTVNNQVNR.SLTAMQVLPTAANTEASSHRLGTGVVPALQAAETGASSNASDKNLIETRSVLNHHS
QOP74931.1     .....GDPIADMIDQTVNNQVNR.SLTAMQVLPTAANTDASSHRLGTGVVPALQAAETGASSNASDKNLIETRCVLNHHS
QIL87531.1     .....GDPIADMIDQTVNNQVNR.SLTAMQVLPTAANTEASSHRLGTGVVPALQAAETGASSNASDKNLIETRCVLNHHS
QIL87521.1     .....GDPIADMIDQTVNNQVNR.SLTAMQVLPTAANTEASSHRLGTGVVPALQAAETGASSNASDKNLIETRCVLNHHS
QIL87523.1     .....GDPIADMIDQTVNNQVNR.SLTAMQVLPTAANTEASSHRLGTGVVPALQAAETGASSNASDKNLIETRCVLNHHS
QIL87517.1     .....GDPIADMIDQTVNNQVNR.SLTAMQVLPTAANTEASSHRLGTGVVPALQAAETGASSNASDKNLIETRCVLNHHS
QIL87540.1     .....GDPIADMIDQTVNNQVNR.SLTAMQVLPTAANTEASSHRLGTGVVPALQAAETGASSNASDKNLIETRCVLNHHS
QIL87539.1     .....GDPIADMIDQTVNNQVNR.SLTAMQVLPTAANTEASSHRLGTGVVPALQAAETGASSNASDKNLIETRCVLNHHS
QIH53921.1     .....GDPIADMIDQTVNNQVNR.SLTAMQVLPTAANTEASSHRLGTGVVPALQAAETGASSNASDKNLIETRCVLNHHS
QIH53916.1     .....GDPIADMIDQTVNNQVNR.SLTAMQVLPTAANTEASSHRLGTGVVPALQAAETGASSNASDKNLIETRCVLNHHS
BBH72194.1     .....GDPIADMIDQTVNNQVNR.SLTAMQVLPTAANTEASSHRLGTGVVPALQAAETGASSNASDKNLIETRCVLNHHS
BBH72183.1     .....GDPIADMIDQTVNNQVNR.SLTAMQVLPTAANTEASSHRLGTGVVPALQAAETGASSNASDKNLIETRCVLNHHS
BBH72107.1     .....GDPIADMIDQTVNNQVNR.SLTAMQVLPTAANTEASSHRLGTGVVPALQAAETGASSNASDKNLIETRCVLNHHS
QWF08825.1     .....GDPIADMIDQTVNNQVNR.SLTAMQVLPTAANTEASSHRLGTGVVPALQAAETGASSNASDKNLIETRCVLNHHS
QWF08823.1     .....GDPIADMIDQTVNNQVNR.SLTAMQVLPTAANTEASSHRLGTGVVPALQAAETGASSNASDKNLIETRCVLNHHS
QWF08821.1     .....GDPIADMIDQTVNNQVNR.SLTAMQVLPTAANTEASSHRLGTGVVPALQAAETGASSNASDKNLIETRCVLNHHS
QWF08818.1     .....GDPIADMIDQTVNNQVNR.SLTAMQVLPTAANTEASSHRLGTGVVPALQAAETGASSNASDKNLIETRCVLNHHS
QWF08815.1     .....GDPIADMIDQTVNNQVNR.SLTAMQVLPTAANTEASSHRLGTGVVPALQAAETGASSNASDKNLIETRCVLNHHS
QWF08813.1     .....GDPIADMIDQTVNNQVNR.SLTAMQVLPTAANTEASSHRLGTGVVPALQAAETGASSNASDKNLIETRCVLNHHS
QWF08812.1     .....GDPIADMIDQTVNNQVNR.SLTAMQVLPTAANTEASSHRLGTGVVPALQAAETGASSNASDKNLIETRCVLNHHS
QWF08810.1     .....GDPIADMIDQTVNNQVNR.SLTAMQVLPTAANTEASSHRLGTGVVPALQAAETGASSNASDKNLIETRCVLNHHS
QWF08809.1     .....GDPIADMIDQTVNNQVNR.SLTAMQVLPTAANTEASSHRLGTGVVPALQAAETGASSNASDKNLIETRCVLNHHS
QWF08808.1     .....GDPIADMIDQTVNNQVNR.SLTAMQVLPTAANTEASSHRLGTGVVPALQAAETGASSNASDKNLIETRCVLNHHS
QWF08807.1     .....GDPIADMIDQTVNNQVNR.SLTAMQVLPTAANTEASSHRLGTGVVPALQAAETGASSNASDKNLIETRCVLNHHS
QWF08797.1     .....GDPIADMIDQTVNNQVNR.SLTAMQVLPTAANTEASSHRLGTGVVPALQAAETGASSNASDKNLIETRCVLNHHS
QWF08841.1     .....GDPIADMIDQTVNNQVNR.SLTAMQVLPTAANTEASSHRLGTGVVPALQAAETGASSNASDKNLIETRCVLNHHS
QWF08830.1     .....GDPIADMIDQTVNNQVNR.SLTAMQVLPTAANTEASSHRLGTGVVPALQAAETGASSNASDKNLIETRCVLNHHS
QTA73892.1     .....GDPIADMIDQTVNNQVNR.SLTAMQVLPTAANTEASSHRLGTGVVPALQAAETGASSNASDKNLIETRCVLNHHS
QTA73901.1     .....GDPIADMIDQTVNNQVNR.SLTAMQVLPTAANTEASSHRLGTGVVPALQAAETGASSNASDKNLIETRCVLNHHS
QWY15957.1     .....GDPIADMIDQTVNNQVNR.SLTAMQVLPTAANTEASSHRLGTGVVPALQAAETGASSNASDKNLIETRCVLNHHS
UEP53406.1     .....GDPIADMIDQTVNNQVNR.SLTAMQVLPTAANTEASSHRLGTGVVPALQAAETGASSNASDKNLIETRCVLNHHS
UEC49858.1     .....GDPIADMIDQTVNNQVNR.SLTAMQVLPTAANTEASSHRLGTGVVPALQAAETGASSNASDKNLIETRCVLNHHS
QWY15931.1     .....GDPIADMIDQTVNNQVNR.SLTAMQVLPTAANTEASSHRLGTGVVPALQAAETGASSNASDKNLIETRCVLNHHS
QWY15929.1     .....GDPIADMIDQTVNNQVNR.SLTAMQVLPTAANTEASSHRLGTGVVPALQAAETGASSNASDKNLIETRCVLNHHS
QWY15880.1     .....GDPIADMIDQTVNNQVNR.SLTAMQVLPTAANTEASSHRLGTGVVPALQAAETGASSNASDKNLIETRCVLNHHS
QWY15868.1     .....GDPIADMIDQTVNNQVNR.SLTAMQVLPTAANTEASSHRLGTGVVPALQAAETGASSNASDKNLIETRCVLNHHS
BCI65368.1     .....GDPIADMIDQTVNNQVNR.SLTAMQVLPTAANTEASSHRLGTGVVPALQAAETGASSNASDKNLIETRCVLNHHS
BCR43097.1     .....GDPIADMIDQTVNNQVNR.SLTAMQVLPTAANTEASSHRLGTGVVPALQAAETGASSNASDKNLIETRCVLNHHS
BCG67047.1     .....GDPIADMIDQTVNNQVNR.SLTAMQVLPTAANTEASSHRLGTGVVPALQAAETGASSNASDKNLIETRCVLNHHS
BCG67046.1     .....GDPIADMIDQTVNNQVNR.SLTAMQVLPTAANTEASSHRLGTGVVPALQAAETGASSNASDKNLIETRCVLNHHS
BCG67041.1     .....GDPIADMIDQTVNNQVNR.SLTAMQVLPTAANTEASSHRLGTGVVPALQAAETGASSNASDKNLIETRCVLNHHS
BCG67040.1     .....GDPIADMIDQTVNNQVNR.SLTAMQVLPTAANTEASSHRLGTGVVPALQAAETGASSNASDKNLIETRCVLNHHS
BCG67039.1     .....GDPIADMIDQTVNNQVNR.SLTAMQVLPTAANTEASSHRLGTGVVPALQAAETGASSNASDKNLIETRCVLNHHS
BCG67038.1     .....GDPIADMIDQTVNNQVNR.SLTAMQVLPTAANTEASSHRLGTGVVPALQAAETGASSNASDKNLIETRCVLNHHS
BCG67032.1     .....GDPIADMIDQTVNNQVNR.SLTAMQVLPTAANTEASSHRLGTGVVPALQAAETGASSNASDKNLIETRCVLNHHS
BCG67030.1     .....GDPIADMIDQTVNNQVNR.SLTAMQVLPTAANTEASSHRLGTGVVPALQAAETGASSNASDKNLIETRCVLNHHS
BCG66995.1     .....GDPIADMIDQTVNNQVNR.SLTAMQVLPTAANTEASSHRLGTGVVPALQAAETGASSNASDKNLIETRCVLNHHS
BCG66994.1     .....GDPIADMIDQTVNNQVNR.SLTAMQVLPTAANTEASSHRLGTGVVPALQAAETGASSNASDKNLIETRCVLNHHS
QWY15757.1     .....GDPIADMIDQTVNNQVNR.SLTAMQVLPTAANTEASSHRLGTGVVPALQAAETGASSNASDKNLIETRCVLNHHS
QWY15756.1     .....GDPIADMIDQTVNNQVNR.SLTAMQVLPTAANTEASSHRLGTGVVPALQAAETGASSNASDKNLIETRCVLNHHS
QWY15748.1     .....GDPIADMIDQTVNNQVNR.SLTAMQVLPTAANTEASSHRLGTGVVPALQAAETGASSNASDKNLIETRCVLNHHS
QWY15747.1     .....GDPIADMIDQTVNNQVNR.SLTAMQVLPTAANTEASSHRLGTGVVPALQAAETGASSNASDKNLIETRCVLNHHS
QWY15720.1     .....GDPIADMIDQTVNNQVNR.SLTAMQVLPTAANTEASSHRLGTGVVPALQAAETGASSNASDKNLIETRCVLNHHS
QWY15717.1     .....GDPIADMIDQTVNNQVNR.SLTAMQVLPTAANTEASSHRLGTGVVPALQAAETGASSNASDKNLIETRCVLNHHS
QWY15697.1     .....GDPIADMIDQTVNNQVNR.SLTAMQVLPTAANTEASSHRLGTGVVPALQAAETGASSNASDKNLIETRCVLNHHS
QWY15689.1     .....GDPIADMIDQTVNNQVNR.SLTAMQVLPTAANTEASSHRLGTGVVPALQAAETGASSNASDKNLIETRCVLNHHS
QWY15761.1     .....GDPIADMIDQTVNNQVNR.SLTAMQVLPTAANTEASSHRLGTGVVPALQAAETGASSNASDKNLIETRCVLNHHS
QWY15743.1     .....GDPIADMIDQTVNNQVNR.SLTAMQVLPTAANTEASSHRLGTGVVPALQAAETGASSNASDKNLIETRCVLNHHS
QWY15759.1     .....GDPIADMIDQTVNNQVNR.SLTAMQVLPTAANTEASSHRLGTGVVPALQAAETGASSNASDKNLIETRCVLNHHS
QWY15758.1     .....GDPIADMIDQTVNNQVNR.SLTAMQVLPTAANTEASSHRLGTGVVPALQAAETGASSNASDKNLIETRCVLNHHS
QWY15677.1     .....GDPIADMIDQTVNNQVNR.SLTAMQVLPTAANTEASSHRLGTGVVPALQAAETGASSNASDKNLIETRCVLNHHS
QWY15675.1     .....GDPIADMIDQTVNNQVNR.SLTAMQVLPTAANTEASSHRLGTGVVPALQAAETGASSNASDKNLIETRCVLNHHS
QWY15776.1     .....GDPIADMIDQTVNNQVNR.SLTAMQVLPTAANTEASSHRLGTGVVPALQAAETGASSNASDKNLIETRCVLNHHS
QWY15772.1     .....GDPIADMIDQTVNNQVNR.SLTAMQVLPTAANTEASSHRLGTGVVPALQAAETGASSNASDKNLIETRCVLNHHS
QWY15770.1     .....GDPIADMIDQTVNNQVNR.SLTAMQVLPTAANTEASSHRLGTGVVPALQAAETGASSNASDKNLIETRCVLNHHS
QWY15762.1     .....GDPIADMIDQTVNNQVNR.SLTAMQVLPTAANTEASSHRLGTGVVPALQAAETGASSNASDKNLIETRCVLNHHS
```

```
QWY15732.1     .....GDPIADMIDQTVNNQVNR.SLTAMQVLPTAANTEASSHRLGTGVVPALQAAETGASSNASDKNLIETRCVLNHHS
QWY15731.1     .....GDPIADMIDQTVNNQVNR.SLTAMQVLPTAANTEASSHRLGTGVVPALQAAETGASSNASDKNLIETRCVLNHHS
QWY15780.1     .....GDPIADMIDQTVNNQVNR.SLTAMQVLPTAANTEASSHRLGTGVVPALQAAETGASSNASDKNLIETRCVLNHHS
QWY15779.1     .....GDPIADMIDQTVNNQVNR.SLTAMQVLPTAANTEASSHRLGTGVVPALQAAETGASSNASDKNLIETRCVLNHHS
BCG67006.1     .....GDPIADMIDQTVNNQVNR.SLTAMQVLPTAANTEASSHRLGTGVVPALQAAETGASSNASDKNLIETRCVLNHHS
BCG67004.1     .....GDPIADMIDQTVNNQVNR.SLTAMQVLPTAANTEASSHRLGTGVVPALQAAETGASSNASDKNLIETRCVLNHHS
BCG67019.1     .....GDPIADMIDQTVNNQVNR.SLTAMQVLPTAANTEASSHRLGTGVVPALQAAETGASSNASDKNLIETRCVLNHHS
BCG67002.1     .....GDPIADMIDQTVNNQVNR.SLTAMQVLPTAANTEASSHRLGTGVVPALQAAETGASSNASDKNLIETRCVLNHHS
BCG67036.1     .....GDPIADMIDQTVNNQVNR.SLTAMQVLPTAANTEASSHRLGTGVVPALQAAETGASSNASDKNLIETRCVLNHHS
BCG67035.1     .....GDPIADMIDQTVNNQVNR.SLTAMQVLPTAANTEASSHRLGTGVVPALQAAETGASSNASDKNLIETRCVLNHHS
QWY15785.1     .....GDPIADMIDQTVNNQVNR.SLTAMQVLPTAANTEASSHRLGTGVVPALQAAETGASSNASDKNLIETRCVLNHHS
QWY15784.1     .....GDPIADMIDQTVNNQVNR.SLTAMQVLPTAANTEASSHRLGTGVVPALQAAETGASSNASDKNLIETRCVLNHHS
QWY15789.1     .....GDPIADMIDQTVNNQVNR.SLTAMQVLPTAANTEASSHRLGTGVVPALQAAETGASSNASDKNLIETRCVLNHHS
QYC65159.1     .....GDPIADMIDQTVNNQVNR.SLTAMQVLPTAANTEASSHRLGTGVVPALQAAETGASSNASDKNLIETRCVLNHHS
BCR43101.1     .....GDPIADMIDQTVNNQVNR.SLTAMQVLPTAANTEASSHRLGTGVVPALQAAETGASSNASDKNLIETRCVLNHHS
BCR43098.1     .....GDPIADMIDQTVNNQVNR.SLTAMQVLPTAANTEASSHRLGTGVVPALQAAETGASSNASDKNLIETRCVLNHHS
QWY15873.1     .....GDPIADMIDQTVNNQVNR.SLTAMQVLPTAANTEASSHRLGTGVVPALQAAETGASSNASDKNLIETRCVLNHHS
QWY15865.1     .....GDPIADMIDQTVNNQVNR.SLTAMQVLPTAANTEASSHRLGTGVVPALQAAETGASSNASDKNLIETRCVLNHHS
QWY15900.1     .....GDPIADMIDQTVNNQVNR.SLTAMQVLPTAANTEASSHRLGTGVVPALQAAETGASSNASDKNLIETRCVLNHHS
BCG67049.1     .....GDPIADMIDQTVNNQVNR.SLTAMQVLPTAANTEASSHRLGTGVVPALQAAETGASSNASDKNLIETRCVLNHHS
UEC49853.1     .....GDPIADMIDQTVNNQVNR.SLTAMQVLPTAANTEASSHRLGTGVVPALQAAETGASSNASDKNLIETRCVLNHHS
UEC49848.1     .....GDPIADMIDQTVNNQVNR.SLTAMQVLPTAANTEASSHRLGTGVVPALQAAETGASSNASDKNLIETRCVLNHHS
QTA73899.1     .....GDPIADMIDQTVNNQVNR.SLTAMQVLPTAANTEASSHRLGTGVVPALQAAETGASSNASDKNLIETRCVLNHHS
QTA73898.1     .....GDPIADMIDQTVNNQVNR.SLTAMQVLPTAANTEASSHRLGTGVVPALQAAETGASSNASDKNLIETRCVLNHHS
QTA73905.1     .....GDPIADMIDQTVNNQVNR.SLTAMQVLPTAANTEASSHRLGTGVVPALQAAETGASSNASDKNLIETRCVLNHHS
QTA73904.1     .....GDPIADMIDQTVNNQVNR.SLTAMQVLPTAANTEASSHRLGTGVVPALQAAETGASSNASDKNLIETRCVLNHHS
QTA73909.1     .....GDPIADMIDQTVNNQVNR.SLTAMQVLPTAANTEASSHRLGTGVVPALQAAETGASSNASDKNLIETRCVLNHHS
QTA73908.1     .....GDPIADMIDQTVNNQVNR.SLTAMQVLPTAANTEASSHRLGTGVVPALQAAETGASSNASDKNLIETRCVLNHHS
UEP53408.1     .....GDPIADMIDQTVNNQVNR.SLTAMQVLPTAANTEASSHRLGTGVVPALQAAETGASSNASDKNLIETRCVLNHHS
QWF08839.1     .....GDPIADMIDQTVNNQVNR.SLTAMQVLPTAANTEASSHRLGTGVVPALQAAETGASSNASDKNLIETRCVLNHHS
QWF08799.1     .....GDPIADMIDQTVNNQVNR.SLTAMQVLPTAANTEASSHRLGTGVVPALQAAETGASSNASDKNLIETRCVLNHHS
QWY15771.1     .....GDPIADMIDQTVNNQVNR.SLTAMQVLPTAANTEASSHRLGTGVVPALQAAETGASSNASDKNLIETRCVLNHHS
QTA73891.1     .....GDPIADMIDQTVNNQVNR.SLTAMQVLPTAANTEASSHRLGTGVVPALQAAETGASSNASDKNLIETRCVLNHHS
QWY15670.1     .....GDPIADMIDQTVNNQVNR.SLTAMQVLPTAANTEASSHRLGTGVVPALQAAETGASSNASDKNLIETRCVLNHHS
BBH72149.1     .....GDPIAHMIDQTVNNQVNR.SLTAMQVLPTAANTEASSHRLGTGVVPALQAAETGASSNASDKNLIETRCVLNHHS
BBH72143.1     .....GDPIAHMIDQTVNNQVNR.SLTAMQVLPTAANTEASSHRLGTGVVPALQAAETGASSNASDKNLIETRCVLNHHS
BBH72163.1     .....GDPIADMIDQTVNNQVNR.SLTAMQVLPTAANTEASSHRLGTGVVPALQAAETGASSNASDKNLIETRCVLNHHS
QTA73895.1     .....GDPIADMIDQTVNNQVNR.SLTAMQVLPTAANTEASSHRLGTGVVPALQAAETGASSNASDKNLIETRCVLNHHS
QWY15674.1     .....GDPIADMIDQTVNNQVNR.SLTAMQVLPTAANTEASSHRLGTGVVPALQAAETGASSNASDKNLIETRCVLNHHS
QWY15710.1     .....GDPIADMIDQTVNNQVNR.SLTAMQVLPTAANTEASSHRLGTGVVPALQAAETGASSNASDKNLIETRCVLNHHS
QWY15709.1     .....GDPIADMIDQTVNNQVNR.SLTAMQVLPTAANTEASSHRLGTGVVPALQAAETGASSNASDKNLIETRCVLNHHS
QWF08934.1     .....GDPIADMIDQTVNNQVNH.SLTAMQVLPTAANTEASSHRLGTGVVPALQAAETGASSNASDKNLIETRCVLNHHS
QWF08836.1     .....GDPIADMIDQTVNNQLNR.SLTAMQVLPTAANTEASSHRLGTGVVPALQAAETGASSNASDKNLIETRCVLNHHS
QLH02014.1     .....GDPIADMIDQTVNNQVNR.SLTAMQVLPTAANTEASSHRLGTGVVPALQAAETGASSNASDKNLIETRCVLNHHS
QWF08796.1     .....GDPIADMIDQTVNNQVNR.SLTAMQVLPTAANTEASSHRLGTGVVPALQAAETGASSNASDKNLIETRCVLNHHS
QYC65090.1     .....GDPIADMINQTVNNQVNR.SLTAMQVLPTAANTEASSHRLGTGVVPALQAAETGASSNASDKNLIETRCVLNHHS
QTA73900.1     .....GDPIADMIDQTVNNQVNR.SLTAMQVLPTAANTEASSHRLGTGVVPALQAAETGASSNASDKNLIETRCVLNHHS
QWY15679.1     .....GDPIADMIDQTVNNQVNR.SLTAMQVLPTAANTEASSHRLGTGVVPALQAAETGASSNASDKNLIETRCVLNHHS
QWY15751.1     .....GDPIADMIDQTVNNQVNR.SLTAMQVLPTAANTEASSHRLGTGVVPALQAAETGASSNASDKNLIETRCVLNHHS
QWF08795.1     .....GDPIADMIDQTVNNQVNR.SLTAMQVLPTAANTEASSHRLGTGVVPALQAAETGASSNASDKNLIETRCVLNHHS
QWF08789.1     .....GDPIADMIDQTVNNQVNR.SLTAMQVLPTAANTEASSHRLGTGVVPALQAAETGASSNASDKNLIETRCVLNHHS
QYC65158.1     .....GDPIADMIDQTVNNQVNR.SLTAMQVLPTAANTEASSHRLGTGVVPALQAAETGASSNASDKNLIETRCVLNHHS
QYC65109.1     .....GDPIADMIDQTVNNQVNR.SLTAMQVLPTAANTEASSHRLGTGVVPALQAAETGASSNASDKNLIETRCVLNHHS
QWF08883.1     .....GDPIADMIDQTVNNQVNR.SLTAMQVLPTAANTEASSHRLGTGVVPALQAAETGASSNASDKNLIETRCVLNHHS
QWF08866.1     .....GDPIADMIDQTVNNQVNR.SLTAMQVLPTAANTEASSHRLGTGVVPALQAAETGASSNASDKNLIETRCVLNHHS
QWF08833.1     .....GDPIADMIDQTVNNQVNR.SLTAMQVLPTAANTEASSHRLGTGVVPALQAAETGASSNASDKNLIETRCVLNHHS
QWF08801.1     .....GDPIADMIDQTVNNQVNR.SLTAMQVLPTAANTEASSHRLGTGVVPALQAAETGASSNASDKNLIETRCVLNHHS
QWF08842.1     .....GDPIADMIDQTVNNQVNR.SLTAMQVLPTAANTEASSHRLGTGVVPALQAAETGASSNASDKNLIETRCVLNHHS
QWF08806.1     .....GDPIADMIDQTVNNQVNR.SLTAMQVLPTAANTEASSHRLGTGVVPALQAAETGASSNASDKNLIETRCVLNHHS
QYC65065.1     .....GDPIADMIDQTVNNQVNR.SLTAMQVLPTAANTEASSHRLGTGVVPALQAAETGASSNASDKNLIETRCVLNHHS
QYC65044.1     .....GDPIADMIDQTVNNQVNR.SLTAMQVLPTAANTEASSHRLGTGVVPALQAAETGASSNASDKNLIETRCVLNHHS
QWY15863.1     .....GDPIADMIDQTVNNQVNR.SLTAMQVLPTAANTEASSHRLGTGVVPALQAAETGASSNASDKNLIETRCVLNHHS
QTA73916.1     .....GDPIADMIDQTVNNQVNR.SLTAMQVLPTAANTEASSHRLGTGIVPALQAAETGASSNASDKNLIETRCVLNHHS
BCG66990.1     .....GDPIADMIDQTVNNQVNR.SLTAMQVLPTAANTEASSHRLGTGIVPALQAAETGASSNASDKNLIETRCVLNHHS
QTA73915.1     .....GDPIADMIDQTVNNQVNR.SLTAMQVLPTAANTEASSHRLGTGIVPALQAAETGASSNASDKNLIETRCVLNHHS
QWY15932.1     .....GDPIADMIDQTVNNQVNR.SLTAMQVLPTAANTEASSHRLGTGIVPALQAAETGASSNASDKNLIETRCVLNHHS
QWY15867.1     .....GDPIADMIDQTVNNQVNR.SLTAMQVLPTAANTEASSHRLGTGIVPALQAAETGASSNASDKNLIETRCVLNHHS
QWY15859.1     .....GDPIADMIDQTVNNQVNR.SLTAMQVLPTAANTEASSHRLGTGIVPALQAAETGASSNASDKNLIETRCVLNHHS
QWY15858.1     .....GDPIADMIDQTVNNQVNR.SLTAMQVLPTAANTEASSHRLGTGIVPALQAAETGASSNASDKNLIETRCVLNHHS
QWY15782.1     .....GDPIADMIDQTVNNQVNR.SLTAMQVLPTAANTEASSHRLGTGIVPALQAAETGASSNASDKNLIETRCVLNHHS
QWY15781.1     .....GDPIADMIDQTVNNQVNR.SLTAMQVLPTAANTEASSHRLGTGIVPALQAAETGASSNASDKNLIETRCVLNHHS
BCI65355.1     .....GDPIADMIDQTVNNQVNR.SLTAMQVLPTAANTEASSHRLGTGIVPALQAAETGASSNASDKNLIETRCVLNHHS
BCI65374.1     .....GDPIADMIDQTVNNQVNR.SLTAMQVLPTAANTEASSHRLGTGIVPALQAAETGASSNASDKNLIETRCVLNHHS
BCI65373.1     .....GDPIADMIDQTVNNQVNR.SLTAMQVLPTAANTEASSHRLGTGIVPALQAAETGASSNASDKNLIETRCVLNHHS
BCI65372.1     .....GDPIADMIDQTVNNQVNR.SLTAMQVLPTAANTEASSHRLGTGIVPALQAAETGASSNASDKNLIETRCVLNHHS
BCI65371.1     .....GDPIADMIDQTVNNQVNR.SLTAMQVLPTAANTEASSHRLGTGIVPALQAAETGASSNASDKNLIETRCVLNHHS
BCI65369.1     .....GDPIADMIDQTVNNQVNR.SLTAMQVLPTAANTEASSHRLGTGIVPALQAAETGASSNASDKNLIETRCVLNHHS
BCI65367.1     .....GDPIADMIDQTVNNQVNR.SLTAMQVLPTAANTEASSHRLGTGIVPALQAAETGASSNASDKNLIETRCVLNHHS
BCI65365.1     .....GDPIADMIDQTVNNQVNR.SLTAMQVLPTAANTEASSHRLGTGIVPALQAAETGASSNASDKNLIETRCVLNHHS
BCI65364.1     .....GDPIADMIDQTVNNQVNR.SLTAMQVLPTAANTEASSHRLGTGIVPALQAAETGASSNASDKNLIETRCVLNHHS
BCI65363.1     .....GDPIADMIDQTVNNQVNR.SLTAMQVLPTAANTEASSHRLGTGIVPALQAAETGASSNASDKNLIETRCVLNHHS
BCI65362.1     .....GDPIADMIDQTVNNQVNR.SLTAMQVLPTAANTEASSHRLGTGIVPALQAAETGASSNASDKNLIETRCVLNHHS
BCI65361.1     .....GDPIADMIDQTVNNQVNR.SLTAMQVLPTAANTEASSHRLGTGIVPALQAAETGASSNASDKNLIETRCVLNHHS
BCI65360.1     .....GDPIADMIDQTVNNQVNR.SLTAMQVLPTAANTEASSHRLGTGIVPALQAAETGASSNASDKNLIETRCVLNHHS
BCI65359.1     .....GDPIADMIDQTVNNQVNR.SLTAMQVLPTAANTEASSHRLGTGIVPALQAAETGASSNASDKNLIETRCVLNHHS
BCI65358.1     .....GDPIADMIDQTVNNQVNR.SLTAMQVLPTAANTEASSHRLGTGIVPALQAAETGASSNASDKNLIETRCVLNHHS
BCI65356.1     .....GDPIADMIDQTVNNQVNR.SLTAMQVLPTAANTEASSHRLGTGIVPALQAAETGASSNASDKNLIETRCVLNHHS
BCI65354.1     .....GDPIADMIDQTVNNQVNR.SLTAMQVLPTAANTEASSHRLGTGIVPALQAAETGASSNASDKNLIETRCVLNHHS
BCI65353.1     .....GDPIADMIDQTVNNQVNR.SLTAMQVLPTAANTEASSHRLGTGIVPALQAAETGASSNASDKNLIETRCVLNHHS
BCI65352.1     .....GDPIADMIDQTVNNQVNR.SLTAMQVLPTAANTEASSHRLGTGIVPALQAAETGASSNASDKNLIETRCVLNHHS
BCI65351.1     .....GDPIADMIDQTVNNQVNR.SLTAMQVLPTAANTEASSHRLGTGIVPALQAAETGASSNASDKNLIETRCVLNHHS
BCI65350.1     .....GDPIADMIDQTVNNQVNR.SLTAMQVLPTAANTEASSHRLGTGIVPALQAAETGASSNASDKNLIETRCVLNHHS
BCI65349.1     .....GDPIADMIDQTVNNQVNR.SLTAMQVLPTAANTEASSHRLGTGIVPALQAAETGASSNASDKNLIETRCVLNHHS
BCI65348.1     .....GDPIADMIDQTVNNQVNR.SLTAMQVLPTAANTEASSHRLGTGIVPALQAAETGASSNASDKNLIETRCVLNHHS
BCI65347.1     .....GDPIADMIDQTVNNQVNR.SLTAMQVLPTAANTEASSHRLGTGIVPALQAAETGASSNASDKNLIETRCVLNHHS
BCI65346.1     .....GDPIADMIDQTVNNQVNR.SLTAMQVLPTAANTEASSHRLGTGIVPALQAAETGASSNASDKNLIETRCVLNHHS
BCI65345.1     .....GDPIADMIDQTVNNQVNR.SLTAMQVLPTAANTEASSHRLGTGIVPALQAAETGASSNASDKNLIETRCVLNHHS
BCI65344.1     .....GDPIADMIDQTVNNQVNR.SLTAMQVLPTAANTEASSHRLGTGIVPALQAAETGASSNASDKNLIETRCVLNHHS
BCI65343.1     .....GDPIADMIDQTVNNQVNR.SLTAMQVLPTAANTEASSHRLGTGIVPALQAAETGASSNASDKNLIETRCVLNHHS
BCI65342.1     .....GDPIADMIDQTVNNQVNR.SLTAMQVLPTAANTEASSHRLGTGIVPALQAAETGASSNASDKNLIETRCVLNHHS
BCI65341.1     .....GDPIADMIDQTVNNQVNR.SLTAMQVLPTAANTEASSHRLGTGIVPALQAAETGASSNASDKNLIETRCVLNHHS
BCI65340.1     .....GDPIADMIDQTVNNQVNR.SLTAMQVLPTAANTEASSHRLGTGIVPALQAAETGASSNASDKNLIETRCVLNHHS
BCI65339.1     .....GDPIADMIDQTVNNQVNR.SLTAMQVLPTAANTEASSHRLGTGIVPALQAAETGASSNASDKNLIETRCVLNHHS
BCI65338.1     .....GDPIADMIDQTVNNQVNR.SLTAMQVLPTAANTEASSHRLGTGIVPALQAAETGASSNASDKNLIETRCVLNHHS
BCI65337.1     .....GDPIADMIDQTVNNQVNR.SLTAMQVLPTAANTEASSHRLGTGIVPALQAAETGASSNASDKNLIETRCVLNHHS
BCI65336.1     .....GDPIADMIDQTVNNQVNR.SLTAMQVLPTAANTEASSHRLGTGIVPALQAAETGASSNASDKNLIETRCVLNHHS
BCI65335.1     .....GDPIADMIDQTVNNQVNR.SLTAMQVLPTAANTEASSHRLGTGIVPALQAAETGASSNASDKNLIETRCVLNHHS
BCI65334.1     .....GDPIADMIDQTVNNQVNR.SLTAMQVLPTAANTEASSHRLGTGIVPALQAAETGASSNASDKNLIETRCVLNHHS
BCI65333.1     .....GDPIADMIDQTVNNQVNR.SLTAMQVLPTAANTEASSHRLGTGIVPALQAAETGASSNASDKNLIETRCVLNHHS
BCI65332.1     .....GDPIADMIDQTVNNQVNR.SLTAMQVLPTAANTEASSHRLGTGIVPALQAAETGASSNASDKNLIETRCVLNHHS
BCI65331.1     .....GDPIADMIDQTVNNQVNR.SLTAMQVLPTAANTEASSHRLGTGIVPALQAAETGASSNASDKNLIETRCVLNHHS
BCI65330.1     .....GDPIADMIDQTVNNQVNR.SLTAMQVLPTAANTEASSHRLGTGIVPALQAAETGASSNASDKNLIETRCVLNHHS
BCI65328.1     .....GDPIADMIDQTVNNQVNR.SLTAMQVLPTAANTEASSHRLGTGIVPALQAAETGASSNASDKNLIETRCVLNHHS
BCI65327.1     .....GDPIADMIDQTVNNQVNR.SLTAMQVLPTAANTEASSHRLGTGIVPALQAAETGASSNASDKNLIETRCVLNHHS
BCI65326.1     .....GDPIADMIDQTVNNQVNR.SLTAMQVLPTAANTEASSHRLGTGIVPALQAAETGASSNASDKNLIETRCVLNHHS
BCI65325.1     .....GDPIADMIDQTVNNQVNR.SLTAMQVLPTAANTEASSHRLGTGIVPALQAAETGASSNASDKNLIETRCVLNHHS
BCI65324.1     .....GDPIADMIDQTVNNQVNR.SLTAMQVLPTAANTEASSHRLGTGIVPALQAAETGASSNASDKNLIETRCVLNHHS
BCI65323.1     .....GDPIADMIDQTVNNQVNR.SLTAMQVLPTAANTEASSHRLGTGIVPALQAAETGASSNASDKNLIETRCVLNHHS
BCI65322.1     .....GDPIADMIDQTVNNQVNR.SLTAMQVLPTAANTEASSHRLGTGIVPALQAAETGASSNASDKNLIETRCVLNHHS
BCI65321.1     .....GDPIADMIDQTVNNQVNR.SLTAMQVLPTAANTEASSHRLGTGIVPALQAAETGASSNASDKNLIETRCVLNHHS
BCI65320.1     .....GDPIADMIDQTVNNQVNR.SLTAMQVLPTAANTEASSHRLGTGIVPALQAAETGASSNASDKNLIETRCVLNHHS
BCI65319.1     .....GDPIADMIDQTVNNQVNR.SLTAMQVLPTAANTEASSHRLGTGIVPALQAAETGASSNASDKNLIETRCVLNHHS
BCI65318.1     .....GDPIADMIDQTVNNQVNR.SLTAMQVLPTAANTEASSHRLGTGIVPALQAAETGASSNASDKNLIETRCVLNHHS
BCI65317.1     .....GDPIADMIDQTVNNQVNR.SLTAMQVLPTAANTEASSHRLGTGIVPALQAAETGASSNASDKNLIETRCVLNHHS
QYC65119.1     .....GDPIADMIDQTVNNQVNR.SLTAMQVLPTAANTEASSHRLGTGIVPALQAAETGASSNASDKNLIETRCVLNHHS
QWF08905.1     .....GDPIADMIDQTVNNQVNR.SLTAMQVLPTAANTEASSHRLGTGIVPALQAAETGASSNASDKNLIETRCVLNHHS
QWF08814.1     .....GDPIADMIDQTVNNQVNR.SLTAMQVLPTAANTEASSHRLGTGIVPALQAAETGASSNASDKNLIETRCVLNHHS
QWF08776.1     .....GDPIADMIDQTVNNQVNR.SLTAMQVLPTAANTEASSHRLGTGIVPALQAAETGASSNASDKNLIETRCVLNHHS
BCI65370.1     .....GDPIADMIDQTVNNQVNR.SLTAMQVLPTAANTEASSHRLGTGIVPALQAAETGASSNASDKNLIETRCVLNHHS
BCG67048.1     .....GDPIADMIDQTVNNQVNR.SLTAMRVLPTAANTEASSHRLGTGIVPALQAAETGASSNASDKNLIETRCVLNHHS
QWY15933.1     .....GDPIVDMIDQTVNNQVNR.SLTAMQVLPTAANTEASSHRLGTGIVPALQAAETGASSNASDKNLIETRCVLNHHS
UEP53404.1     .....GDPIADMIDQTVNSQVNR.SLTAMQVLPTAANTEASSHRLGTGIVPALQAAETGASSNASDKNLIETRCVLNHHS
UEC49857.1     .....GDPIADMIDQTVNSQVNR.SLTAMQVLPTAANTEASSHRLGTGIVPALQAAETGASSNASDKNLIETRCVLNHHS
QYC65076.1     .....GDPIADMIDQTVNSQVNR.SLTAMQVLPTAANTEASSHRLGTGVVPALQAAETGASSNASDKNLIETRCVLNHHS
QWF08819.1     .....GDPIADMIDQTVNSQVNR.SLTAMQVLPTAANTEASSHRLGTGVVPALQAAETGASSNASDKNLIETRCVLNHHS
QYC65082.1     .....GDPIADMIDQTVNSQVNR.SLTAMQVLPTAANTEASSHRLGTGIVPALQAAETGASSNASDKNLIETRCVLNHHS
QWY15927.1     .....GDPIADMIDQTVNNQVNR.SLTAMQVLPTAANTEASSHRLGTGIVPALQAAETGASSNASDKNLIETRCVLNHHS
QOP74944.1     .....GDPIADMIDQTVNNQVNR.SLTAMQVLPTAANTEASSHRLGTGIVPALQAAETGASSNASDKNLIETRCVLNHHS
QYC65139.1     .....GDPIADMIDQTVNNQVNR.SLTAMQVLPTAANTEASSHRLGTGIVPALQAAETGASSNASDKNLIETRCVLNHHS
QYC65136.1     .....GDPIADMIDQTVNNQVNR.SLTAMQVLPTAANTEASSHRLGTGIVPALQAAETGASSNASDKNLIETRCVLNHHS
QYC65130.1     .....GDPIADMIDQTVNNQVNR.SLTAMQVLPTAANTEASSHRLGTGIVPALQAAETGASSNASDKNLIETRCVLNHHS
QYC65115.1     .....GDPIADMIDQTVNNQVNR.SLTAMQVLPTAANTEASSHRLGTGIVPALQAAETGASSNASDKNLIETRCVLNHHS
QYC65114.1     .....GDPIADMIDQTVNNQVNR.SLTAMQVLPTAANTEASSHRLGTGIVPALQAAETGASSNASDKNLIETRCVLNHHS
QYC65105.1     .....GDPIADMIDQTVNNQVNR.SLTAMQVLPTAANTEASSHRLGTGIVPALQAAETGASSNASDKNLIETRCVLNHHS
```

```
QWF08949.1    .....GDPIADMIDQTVNNQVNR.SLTAMQVLPTAANTEASSHRLGTGIVPALQAAETGASSNASDKNLIETRCVLNHHS
QWF08928.1    .....GDPIADMIDQTVNNQVNR.SLTAMQVLPTAANTEASSHRLGTGIVPALQAAETGASSNASDKNLIETRCVLNHHS
QWY15921.1    .....GDPIADMIDQTVNNQVNR.SLTAMQVLPTAANTEASSHRLGTGIVPALQAAETGASSNASDKNLIETRCVLNHHS
QWY15919.1    .....GDPIADMIDQTVNNQVNR.SLTAMQVLPTAANTEASSHRLGTGIVPALQAAETGASSNASDKNLIETRCVLNHHS
QYC65102.1    .....GDPIADMIDQTVNNQVNR.SLTATQVLPTAANTEASSHRLGTGIVPALQAAETGASSNASDKNLIETRCVLNHHS
BCR43082.1    .....GDPIADMIDQTVNSQVNR.SLTAMQVLPTAANTEASSHRLGTGIVPALQAAETGASSNASDKNLIETRCVLNHHS
QWY15713.1    .....GDPIADMIDQTINNQVNR.SLTAMQVLPTAANTEASSHRLGTGVVPALQAAETGASSNASDKNLIETRCVLNHHS
BCI65329.1    .....GDPIADMIDQTINNQVNR.SLTAMQVLPTAANTEASSHRLGTGIVPALQAAETGASSNASDKNLIETRCVLNHHS
QWF08887.1    .....GDPIADMIDQTVNNQVNR.SLTAMQVLPTAANTEASSHRLGTGVVPALQAAETGASSNASDKNLIETRCVLNHHS
QYC65036.1    .....GDPITDMIDQTVNNQVNR.SLTAMQVLPTAANTEASSHRLGTGVVPALQAAETGASSNASDKNLIETRCVLNHHS
QTA73913.1    .....GDPIADMIDQTVNNQVNR.SLTAMQVLPTAANTEASSHRLGTGVVPALQAAETGASSNASDKNLIETRCVLNHHS
QWY15837.1    .....GDPIADMIDQTVNNQVNR.SLTAMQVLPTAANTEASSHRLGTGVVPALQAAETGASSNASDKNLIETRCVLNHHS
QWY15738.1    .....GDPIADMIDQTVNNQVNR.SLTAMQVLPTAANTEASSHRLGTGVVPALQAAETGASSNASDKNLIETRCVLNHHS
QTA73911.1    .....GDPIADMIDQTVNNQVNR.SLTAMQVLPTAANTEASSHRLGTGVVPALQAAETGASSNASDKNLIETRCVLNHHS
QTA73910.1    .....GDPIADMIDQTVNNQVNR.SLTAMQVLPTAANTEASSHRLGTGVVPALQAAETGASSNASDKNLIETRCVLNHHS
QWY15916.1    .....GDPIADMIDQTVNNQVNR.SLTAMQVLPTAANTEASSHRLGTGVVPALQAAETGASSNASDKNLIETRCVLNHHS
QWY15915.1    .....GDPIADMIDQTVNNQVNR.SLTAMQVLPTAANTEASSHRLGTGVVPALQAAETGASSNASDKNLIETRCVLNHHS
QWY15835.1    .....GDPIADMIDQTVNNQVNR.SLTAMQVLPTAANTEASSHRLGTGVVPALQAAETGASSNASDKNLIETRCVLNHHS
QWY15832.1    .....GDPIADMIDQTVNNQVNR.SLTAMQVLPTAANTEASSHRLGTGVVPALQAAETGASSNASDKNLIETRCVLNHHS
QWY15830.1    .....GDPIADMIDQTVNNQVNR.SLTAMQVLPTAANTEASSHRLGTGVVPALQAAETGASSNASDKNLIETRCVLNHHS
QWY15829.1    .....GDPIADMIDQTVNNQVNR.SLTAMQVLPTAANTEASSHRLGTGVVPALQAAETGASSNASDKNLIETRCVLNHHS
QWY15826.1    .....GDPIADMIDQTVNNQVNR.SLTAMQVLPTAANTEASSHRLGTGVVPALQAAETGASSNASDKNLIETRCVLNHHS
QWY15802.1    .....GDPIADMIDQTVNNQVNR.SLTAMQVLPTAANTEASSHRLGTGVVPALQAAETGASSNASDKNLIETRCVLNHHS
QWY15799.1    .....GDPIADMIDQTVNNQVNR.SLTAMQVLPTAANTEASSHRLGTGVVPALQAAETGASSNASDKNLIETRCVLNHHS
QWY15788.1    .....GDPIADMIDQTVNNQVNR.SLTAMQVLPTAANTEASSHRLGTGVVPALQAAETGASSNASDKNLIETRCVLNHHS
QWY15783.1    .....GDPIADMIDQTVNNQVNR.SLTAMQVLPTAANTEASSHRLGTGVVPALQAAETGASSNASDKNLIETRCVLNHHS
QWY15768.1    .....GDPIADMIDQTVNNQVNR.SLTAMQVLPTAANTEASSHRLGTGVVPALQAAETGASSNASDKNLIETRCVLNHHS
QWY15766.1    .....GDPIADMIDQTVNNQVNR.SLTAMQVLPTAANTEASSHRLGTGVVPALQAAETGASSNASDKNLIETRCVLNHHS
QWY15764.1    .....GDPIADMIDQTVNNQVNR.SLTAMQVLPTAANTEASSHRLGTGVVPALQAAETGASSNASDKNLIETRCVLNHHS
QWY15763.1    .....GDPIADMIDQTVNNQVNR.SLTAMQVLPTAANTEASSHRLGTGVVPALQAAETGASSNASDKNLIETRCVLNHHS
QWY15760.1    .....GDPIADMIDQTVNNQVNR.SLTAMQVLPTAANTEASSHRLGTGVVPALQAAETGASSNASDKNLIETRCVLNHHS
QWF08768.1    .....GDPIADMIDQTVNNQVNR.SLTAMQVLPTAANTEASSHRLGTGVVPALQAAETGASSNASDKNLIETRCVLNHHS
QWF08766.1    .....GDPIADMIDQTVNNQVNR.SLTAMQVLPTAANTEASSHRLGTGVVPALQAAETGASSNASDKNLIETRCVLNHHS
QWY15790.1    .....GDPIADMIDQTVNNQVNR.SLTAMQVLPTAANTEASSHRLGTGVVPALQAAETGASSNASDKNLIETRCVLNHHS
QWF08769.1    .....GDPIADMIDQTVNNQVNR.SLTAMRVLPTAANTEASSHRLGTGVVPALQAAETGASSNASDKNLIETRCVLNHHS
QWY15930.1    .....GDPIADMIDQTVNNQVNR.SLTAMQVLPTAANTEASSHRLGTGIVPALQAAETGASSNASDKNLIETRCVLNHHS
QWY15833.1    .....GDPIADMIDQTVNNQVNR.SLTAMQVLPTAANTEASSHRLGTGVVPALQAAETGASSNASDKNLIETRCVLNHHS
QWY15827.1    .....GDPIADMIDQTVNNQVNR.SLTAMQVLPTAANTEASSHRLGTGVVPALQAAETGASSNASDKNLIETRCVLNHHS
QWY15838.1    .....GDPIADMIDQTVNNQVNR.SLTAMQVLPTAANTEASSHRLGTGVVPALQAAETGASSNASDKNLIETRCVLNHHS
BBU37460.1    .....GDPIADMIDQTVNNQVNR.SLTAMQVLPTAANTEASSHRLGTGVVPALQAAETGASSNASDKNLIETRCVLNHHS
QWF08920.1    .....GDPIADMIDQTVNNQVNR.SLTAMQVLPTAANTEASSHRLGTGVVPALQAAETGASSNASDKNLIETRCVLNHHS
QWY15769.1    .....GDPIADMIDQTVNNQVNR.SLTAMQVLPTAANTEASSHRLGTGVVPALQAAETGASSNASDKNLIETRCVLNHHS
QWY15755.1    .....GDPIADMIDQTVNNQVNR.SLTAMQVLPTAANTEASSHRLGTGVVPALQAAETGASSNASDKNLIETRCVLNHHS
QWY15702.1    .....GDPIADMIDQTVNNQVNR.SLTAMQVLPTAANTEASSHRLGTGVVPALQAAETGASSNASDKNLIETRCVLNHHS
QWY15739.1    .....GDPIADMIDQTVNNQVNR.SLTAMQVLPTAANTEASSHRLGTGVVPALQAAETGASSNASDKNLIETRCVLNHHS
QWY15737.1    .....GDPIADMIDQTVNNQVNR.SLTAMQVLPTAANTEASSHRLGTGVVPALQAAETGASSNASDKNLIETRCVLNHHS
QWF08788.1    .....GDPIADMIDQTVNNQVNR.SLTAMQVLPTAANTEASSHRLRTGVVPALQAAETGASSNASDKNLIETRCVLNHHS
QYC65122.1    .....GDPIADMIDQTVNNQVNR.SLTAMQVLPTAANTEASSHRLGTGVVPALQAAETGASSNASDKNLIETRCVLNHHS
QWF08891.1    .....GDPIADMIDQTVNNQVNR.SLTAMQVLPTAANTEASSHRLGTGVVPALQAAETGTSSNASDKNLIETRCVLNHHS
QWY15903.1    .....GDPIADMIDQTVNNQVNR.SLTAMQVLPTAANTEASSHRLGTGVVPALQAAETGASSNASDKNLIETRCVLNHHS
BCI65366.1    .....GDPIADMIDQTVNNQVNR.SLTAMQVLPTAANTEASSHRLGTGVVPALQAAETGASSNASDKNLIETRCVLNHHS
BCG67045.1    .....GDPIADMIDQTVNNQVNR.SLTAMQVLPTAANTEASSHRLGTGVVPALQAAETGASSNASDKNLIETRCVLNHHS
BCG67044.1    .....GDPIADMIDQTVNNQVNR.SLTAMQVLPTAANTEASSHRLGTGVVPALQAAETGASSNASDKNLIETRCVLNHHS
QWY15819.1    .....GDPIADMIDQAVNNQVNR.SLTAMQVLPTAANTEASSHRLGTGVVPALQAAETGASSNASDKNLIETRCVLNHHS
QWY15818.1    .....GDPIADMIDQAVNNQVNR.SLTAMQVLPTAANTEASSHRLGTGVVPALQAAETGASSNASDKNLIETRCVLNHHS
QTA73912.1    .....GDPIADMIDQTVNSQVNR.SLTAMQVLPTAANTEASSHRLGTGVVPALQAAETGASSNASDKNLIETRCVLNHHS
QWY15914.1    .....GDPIADMIDQTVNSQVNR.SLTAMQVLPTAANTEASSHRLGTGVVPALQAAETGASSNASDKNLIETRCVLNHHS
QWY15909.1    .....GDPIADMIDQTVNSQVNR.SLTAMQVLPTAANTEASSHRLGTGVVPALQAAETGASSNASDKNLIETRCVLNHHS
QWY15905.1    .....GDPIADMIDQTVNSQVNR.SLTAMQVLPTAANTEASSHRLGTGVVPALQAAETGASSNASDKNLIETRCVLNHHS
QWY15856.1    .....GDPIADMIDQTVNSQVNR.SLTAMQVLPTAANTEASSHRLGTGVVPALQAAETGASSNASDKNLIETRCVLNHHS
QWY15850.1    .....GDPIADMIDQTVNSQVNR.SLTAMQVLPTAANTEASSHRLGTGVVPALQAAETGASSNASDKNLIETRCVLNHHS
QWY15849.1    .....GDPIADMIDQTVNSQVNR.SLTAMQVLPTAANTEASSHRLGTGVVPALQAAETGASSNASDKNLIETRCVLNHHS
QWY15847.1    .....GDPIADMIDQTVNSQVNR.SLTAMQVLPTAANTEASSHRLGTGVVPALQAAETGASSNASDKNLIETRCVLNHHS
QWY15845.1    .....GDPIADMIDQTVNSQVNR.SLTAMQVLPTAANTEASSHRLGTGVVPALQAAETGASSNASDKNLIETRCVLNHHS
QWY15836.1    .....GDPIADMIDQTVNSQVNR.SLTAMQVLPTAANTEASSHRLGTGVVPALQAAETGASSNASDKNLIETRCVLNHHS
QWY15765.1    .....GDPIADMIDQTVNSQVNR.SLTAMQVLPTAANTEASSHRLGTGVVPALQAAETGASSNASDKNLIETRCVLNHHS
QWY15734.1    .....GDPIADMIDQTVNSQVNR.SLTAMQVLPTAANTEASSHRLGTGVVPALQAAETGASSNASDKNLIETRCVLNHHS
QWY15855.1    .....GDPIADMIDQTVNSQVNR.SLTAMQVLPTASNTEASSHRLGTGVVPALQAAETGASSNASDKNLIETRCVLNHHS
QWY15831.1    .....GDPIADMIDQTVNSQVNR.SLTAMQVLPTAANTEASSHRLGTGVVPALQAAETGASSNASDKNLIETRCVLNHHS
QWY15798.1    .....GDPIADMIDQTVNSQVNR.SLTAMQVLPTAANTEASSHRLGTGVVPALQAAETGASSNASDKNLIETRCVLNHHS
QWY15797.1    .....GDPIADMIDQTVNSQVNR.SLTAMQVLPTAANTEASSHRLGTGVVPALQAAETGASSNASDKNLIETRCVLNHHS
QWY15796.1    .....GDPIADMIDQTVNSQVNR.SLTAMQVLPTAANTEASSHRLGTGVVPALQAAETGASSNASDKNLIETRCVLNHHS
QWY15795.1    .....GDPIADMIDQTVNSQVNR.SLTAMQVLPTAANTEASSHRLGTGVVPALQAAETGASSNASDKNLIETRCVLNHHS
QWY15794.1    .....GDPIADMIDQTVNSQVNR.SLTAMQVLPTAANTEASSHRLGTGVVPALQAAETGASSNASDKNLIETRCVLNHHS
QWY15971.1    .....GDPIADMIDQTVNSQVNR.SLTAMQVLPTAADTEASSHRLGTGVVPALQAAETGASSNASDKNLIETRCVLNHHS
QWY15897.1    .....GDPIADMIDQTVNSQVNR.SLTAMQVLPTAADTEASSHRLGTGVVPALQAAETGASSNASDKNLIETRCVLNHHS
QWY15742.1    .....GDPIADMIDQTVNSQVNR.SLTAMQVLPTAADTEASSHRLGTGVVPALQAAETGASSNASDKNLIETRCVLNHHS
QWY15741.1    .....GDPIADMIDQTVNSQVNR.SLTAMQVLPTAADTEASSHRLGTGVVPALQAAETGASSNASDKNLIETRCVLNHHS
QWY15923.1    .....GDPIADMIDQTVNSQVNR.SLTAMQVLPTAANTEASSHRLGTGVVPALQAAETGASSNASDKNLIETRCVLNHHS
QWF08957.1    .....GDPIADMIDQTVNSQVNR.SLTAMQVLPTAANTEASSHRLGTGVVPALQAAETGASSNASDKNLIETRCVLNHHS
UEP53407.1    .....GDPIADMIDQTVNSQVNR.SLTAMQVLPTAANTEASSHRLGTGVVPALQAAETGASSNASDKNLIETRCVLNHHS
QWY15922.1    .....GDPIADMIDQTVNSQVNR.SLTAMQVLPTAANTEASSHRLGTGVVPALQAAETGASSNASDKNLIETRCVLNHHS
QWY15848.1    .....GDPIADMIDQTVNNQVNR.SLTAMQVLPTAANTEASSHRLGTGVVPALQAAETGASSNASDKNLIETRCVLNHHS
UEP53405.1    .....GDPIADMIDQTVNSQVNR.SLTAMQVLPTAANTEASSHRLGTGVVPALQAAETGASSNASDKNLIETRCVLNHHS
UEC49855.1    .....GDPIADMIDQTVNSQVNR.SLTAMQVLPTAANTEASSHRLGTGVVPALQAAETGASSNASDKNLIETRCVLNHHS
QWY15851.1    .....GDPIADMIDQTVNSQVNR.SLTAMQVLPTAANTEASSHRLGTGVVPALQAAETGASSNASDKNLIETRCVLNHHS
QYC65112.1    .....GDPIADMIDQTVNSQVNR.SLTAMQVLPTAANTEASSHRLGTGVVPALQAAETGASSNASDKNLIETRCVLNHHS
QOP74942.1    .....GDPIADMIDQTVNSQVNR.SLTAMQVLPTAANTEASSHRLGTGVVPALQAAETGASSNASDKNLIETRCVLNHHS
QLH02013.1    .....GDPIADMIDQTVNSQVNR.SLTAMQVLPTAANTEASSHRLGTGVVPALQAAETGASSNASDKNLIETRCVLNHHS
QIH53923.1    .....GDPIADMIDQTVNSQVNR.SLTAMQVLPTAANTEASSHRLGTGVVPALQAAETGASSNASDKNLIETRCVLNHHS
QIH53918.1    .....GDPIADMIDQTVNSQVNR.SLTAMQVLPTAANTEASSHRLGTGVVPALQAAETGASSNASDKNLIETRCVLNHHS
QIH53915.1    .....GDPIADMIDQTVNSQVNR.SLTAMQVLPTAANTEASSHRLGTGVVPALQAAETGASSNASDKNLIETRCVLNHHS
QIH53914.1    .....GDPIADMIDQTVNSQVNR.SLTAMQVLPTAANTEASSHRLGTGVVPALQAAETGASSNASDKNLIETRCVLNHHS
QYC65127.1    .....GDPIADMIDQTVNSQVNR.SLTAMQVLPTAANTEASSHRLGTGVVPALQAAETGASSNASDKNLIETRCVLNHHS
QIL87534.1    .....GDPIADMIDQTVNSQVNR.SLTAMQVLPTAANTEASSHRLGTGVVPALQAAETGASSNASDKNLIETRCVLNHHS
QWF08936.1    .....GDPIADMIDQTVNSQVNR.SLTAMQVLPTAANTEASSHRLGTGVVPALQAAETGASSNASDKNLIETRCVLNHHS
QIL87532.1    .....GDPIADMIDQTVNSQVNR.SLTAMQVLPTAANTEASSHRLGTGVVPALQAAETGASSNASDKNLIETRCVLNHHS
QYC65089.1    .....GDPIADMIDQTVNSQVNR.SLTAMQVLPTAANTEASSHRLGTGVVPALQAAETGASSNASDKNLIETRCVLNHHS
QWF08950.1    .....GDPIADMIDQTVNSQVNR.SLTAMQVLPTAANTEASSHRLGTGVVPALQAAETGASSNASDKNLIETRCVLNHHS
QIL87530.1    .....GDPIADMIDQTVNSQVNR.SLTAMQVLPTAANTEASSHRLGTGVVPALQAAETGASSNASDKNLIETRCVLNHHS
QWF08820.1    .....GDPIADMIDQTVNSQVNR.SLTAMQVLPTAANTEASSHRLGTGVVPALQAAETGASSNASDKNLIETRCVLNHHS
QIL87529.1    .....GDPIADMIDQTVNSQVNR.SLTAMQVLPTAANTEASSHRLGTGVVPALQAAETGASSNASDKNLIETRCVLNHHS
QIL87528.1    .....GDPIADMIDQTVNSQVNR.SLTAMQVLPTAANTEASSHRLGTGVVPALQAAETGASSNASDKNLIETRCVLNHHS
QYC65143.1    .....GDPIADMIDQTVNSQVNR.SLTAMQVLPTAANTEASSHRLGTGVVPALQAAETGASSNASDKNLIETRCVLNHHS
QIL87525.1    .....GDPIADMIDQTVNSQVNR.SLTAMQVLPTAANTEASSHRLGTGVVPALQAAETGASSNASDKNLIETRCVLNHHS
QYC65141.1    .....GDPIADMIDQTVNSQVNR.SLTAMQVLPTAANTEASSHRLGTGVVPALQAAETGASSNASDKNLIETRCVLNHHS
QYC65084.1    .....GDPIADMIDQTVNSQVNR.SLTAMQVLPTAANTEASSHRLGTGVVPALQAAETGASSNASDKNLIETRCVLNHHS
QWF08947.1    .....GDPIADMIDQTVNSQVNR.SLTAMQVLPTAANTEASSHRLGTGVVPALQAAETGASSNASDKNLIETRCVLNHHS
QWF08922.1    .....GDPIADMIDQTVNSQVNR.SLTAMQVLPTAANTEASSHRLGTGVVPALQAAETGASSNASDKNLIETRCVLNHHS
QIL87524.1    .....GDPIADMIDQTVNSQVNR.SLTAMQVLPTAANTEASSHRLGTGVVPALQAAETGASSNASDKNLIETRCVLNHHS
QIL87520.1    .....GDPIADMIDQTVNSQVNR.SLTAMQVLPTAANTEASSHRLGTGVVPALQAAETGASSNASDKNLIETRCVLNHHS
QIL87518.1    .....GDPIADMIDQTVNSQVNR.SLTAMQVLPTAANTEASSHRLGTGVVPALQAAETGASSNASDKNLIETRCVLNHHS
QYC65103.1    .....GDPIADMIDQTVNSQVNR.SLTAMQVLPTAANTEASSHRLGTGVVPALQAAETGASSNASDKNLIETRCVLNHHS
QWF08944.1    .....GDPIADMIDQTVNSQVNR.SLTAMQVLPTAANTEASSHRLGTGVVPALQAAETGASSNASDKNLIETRCVLNHHS
QWF08943.1    .....GDPIADMIDQTVNSQVNR.SLTAMQVLPTAANTEASSHRLGTGVVPALQAAETGASSNASDKNLIETRCVLNHHS
QWF08927.1    .....GDPIADMIDQTVNSQVNR.SLTAMQVLPTAANTEASSHRLGTGVVPALQAAETGASSNASDKNLIETRCVLNHHS
QWF08942.1    .....GDPIADMIDQTVNSQVNR.SLTAMQVLPTAANTEASSHRLGTGVVPALQAAETGASSNASDKNLIETRCVLNHHS
QWF08940.1    .....GDPIADMIDQTVNSQVNR.SLTAMQVLPTAANTEASSHRLGTGVVPALQAAETGASSNASDKNLIETRCVLNHHS
QWF08912.1    .....GDPIADMIDQTVNSQVNR.SLTAMQVLPTAANTEASSHRLGTGVVPALQAAETGASSNASDKNLIETRCVLNHHS
QWF08907.1    .....GDPIADMIDQTVNSQVNR.SLTAMQVLPTAANTEASSHRLGTGVVPALQAAETGASSNASDKNLIETRCVLNHHS
QWF08904.1    .....GDPIADMIDQTVNSQVNR.SLTAMQVLPTAANTEASSHRLGTGVVPALQAAETGASSNASDKNLIETRCVLNHHS
QWF08886.1    .....GDPIADMIDQTVNSQVNR.SLTAMQVLPTAANTEASSHRLGTGVVPALQAAETGASSNASDKNLIETRCVLNHHS
QWY15877.1    .....GDPIADMIDQTVNSQVNR.SLTAMQVLPTAANTEASSHRLGTGVVPALQAAETGASSNASDKNLIETRCVLNHHS
QWY15965.1    .....GDPIADMIDQTVNSQVNR.SLTAMQVLPTAANTEASSHRLGTGVVPALQAAETGASSNASDKNLIETRCVLNHHS
UEC49851.1    .....GDPIADMIDQTVNSQVNR.SLTAMQVLPTAANTEASSHRLGTGVVPALQAAETGASSNASDKNLIETRCVLNHHS
UEC49850.1    .....GDPIADMIDQTVNSQVNR.SLTAMQVLPTAANTEASSHRLGTGVVPALQAAETGASSNASDKNLIETRCVLNHHS
UEC49849.1    .....GDPIADMIDQTVNSQVNR.SLTAMQVLPTAANTEASSHRLGTGVVPALQAAETGASSNASDKNLIETRCVLNHHS
QWY15963.1    .....GDPIADMIDQTVNSQVNR.SLTAMQVLPTAANTEASSHRLGTGVVPALQAAETGASSNASDKNLIETRCVLNHHS
QWY15972.1    .....GDPIADMIDQTVNSQVNR.SLTAMQVLPTAANTEASSHRLGTGVVPALQAAETGASSNASDKNLIETRCVLNHHS
QWY15958.1    .....GDPIADMIDQTVNSQVNR.SLTAMQVLPTAANTEASSHRLGTGVVPALQAAETGASSNASDKNLIETRCVLNHHS
QWY15899.1    .....GDPIADMIDQTVNSQVNR.SLTAMQVLPTAANTEASSHRLGTGVVPALQAAETGASSNASDKNLIETRCVLNHHS
QWY15898.1    .....GDPIADMIDQTVNSQVNR.SLTAMQVLPTAANTEASSHRLGTGVVPALQAAETGASSNASDKNLIETRCVLNHHS
QWY15895.1    .....GDPIADMIDQTVNSQVNR.SLTAMQVLPTAANTEASSHRLGTGVVPALQAAETGASSNASDKNLIETRCVLNHHS
QWY15893.1    .....GDPIADMIDQTVNSQVNR.SLTAMQVLPTAANTEASSHRLGTGVVPALQAAETGASSNASDKNLIETRCVLNHHS
QWY15892.1    .....GDPIADMIDQTVNSQVNR.SLTAMQVLPTAANTEASSHRLGTGVVPALQAAETGASSNASDKNLIETRCVLNHHS
QWY15891.1    .....GDPIADMIDQTVNSQVNR.SLTAMQVLPTAANTEASSHRLGTGVVPALQAAETGASSNASDKNLIETRCVLNHHS
QWY15970.1    .....GDPIADMIDQTVNSQVNR.SLTAMQVLPTAANTEASSHRLGTGVVPALQAAETGASSNASDKNLIETRCVLNHHS
QWY15966.1    .....GDPIADMIDQTVNSQVNR.SLTAMQVLPTAANTEASSHRLGTGVVPALQAAETGASSNASDKNLIETRCVLNHHS
QWY15962.1    .....GDPIADMIDQTVNSQVNR.SLTAMQVLPTAANTEASSHRLGTGVVPALQAAETGASSNASDKNLIETRCVLNHHS
QWY15961.1    .....GDPIADMIDQTVNSQVNR.SLTAMQVLPTAANTEASSHRLGTGVVPALQAAETGASSNASDKNLIETRCVLNHHS
QWY15925.1    .....GDPIADMIDQTVNSQVNR.SLTAMQVLPTAANTEASSHRLGTGVVPALQAAETGASSNASDKNLIETRCVLNHHS
QWY15773.1    .....GDPIADMIDQTVNSQVNR.SLTAMQVLPTAANTEASSHRLGTGVVPALQAAETGASSNASDKNLIETRCVLNHHS
QWY15924.1    .....GDPIADMIDQTVNSQVNR.SLTAMQVLPTAANTEASSHRLGTGVVPALQAAETGASSNASDKNLIETRCVLNHHS
QWY15910.1    .....GDPIADMIDQTVNSQVNR.SLTAMQVLPTAANTEASSHRLGTGVVPALQAAETGASSNASDKNLIETRCVLNHHS
```

```
QWY15824.1    .....GDPIADMIDQTVNSQVNR.SLTAMQVLPTAANTEASSHRLGTGVVPALQAAETGASSNASDKNLIETRCVLNHHS
QWY15823.1    .....GDPIADMIDQTVNSQVNR.SLTAMQVLPTAANTEASSHRLGTGVVPALQAAETGASSNASDKNLIETRCVLNHHS
QWY15822.1    .....GDPIADMIDQTVNSQVNR.SLTAMQVLPTAANTEASSHRLGTGVVPALQAAETGASSNASDKNLIETRCVLNHHS
QWY15821.1    .....GDPIADMIDQTVNSQVNR.SLTAMQVLPTAANTEASSHRLGTGVVPALQAAETGASSNASDKNLIETRCVLNHHS
QWY15816.1    .....GDPIADMIDQTVNSQVNR.SLTAMQVLPTAANTEASSHRLGTGVVPALQAAETGASSNASDKNLIETRCVLNHHS
QWY15778.1    .....GDPIADMIDQTVNSQVNR.SLTAMQVLPTAANTEASSHRLGTGVVPALQAAETGASSNASDKNLIETRCVLNHHS
QWY15801.1    .....GDPIADMIDQTVNSQVNR.SLTAMQVLPTAANTEASSHRLGTGVVPALQAAETGASSNASDKNLIETRCVLNHHS
QWY15791.1    .....GDPIADMIDQTVNSQVNR.SLTAMQVLPTAANTEASSHRLGTGVVPALQAAETGASSNASDKNLIETRCVLNHHS
QWY15775.1    .....GDPIADMIDQTVNSQVNR.SLTAMQVLPTAANTEASSHRLGTGVVPALQAAETGASSNASDKNLIETRCVLNHHS
QWY15774.1    .....GDPIADMIDQTVNSQVNR.SLTAMQVLPTAANTEASSHRLGTGVVPALQAAETGASSNASDKNLIETRCVLNHHS
BCR43107.1    .....GDPIADMIDQTVNSQVNR.SLTAMQVLPTAANTEASSHRLGTGVVPALQAAETGASSNASDKNLIETRCVLNHHS
QYC65183.1    .....GDPIADMIDQTVNSQVNR.SLTAMQVLPTAANTEASSHRLGTGVVPALQAAETGASSNASDKNLIETRCVLNHHS
BCR43094.1    .....GDPIADMIDQTVNSQVNR.SLTAMQVLPTAANTEASSHRLGTGVVPALQAAETGASSNASDKNLIETRCVLNHHS
BCR43081.1    .....GDPIADMIDQTVNSQVNR.SLTAMQVLPTAANTEASSHRLGTGVVPALQAAETGASSNASDKNLIETRCVLNHHS
BCG67017.1    .....GDPIADMIDQTVNSQVNR.SLTAMQVLPTAANTEASSHRLGTGVVPALQAAETGASSNASDKNLIETRCVLNHHS
BCG67015.1    .....GDPIADMIDQTVNSQVNR.SLTAMQVLPTAANTEASSHRLGTGVVPALQAAETGASSNASDKNLIETRCVLNHHS
BCG67007.1    .....GDPIADMIDQTVNSQVNR.SLTAMQVLPTAANTEASSHRLGTGVVPALQAAETGASSNASDKNLIETRCVLNHHS
BCG67005.1    .....GDPIADMIDQTVNSQVNR.SLTAMQVLPTAANTEASSHRLGTGVVPALQAAETGASSNASDKNLIETRCVLNHHS
QYC65184.1    .....GDPIADMIDQTVNSQVNR.SLTAMQVLPTAANTEASSHRLGTGVVPALQAAETGASSNASDKNLIETRCVLNHHS
QWF08853.1    .....GDPIADMIDQTVNSQVNR.SLTAMQVLPTAANTEASSHRLGTGVVPALQAAETGASSNASDKNLIETRCVLNHHS
QYC65140.1    .....GDPIADMIDQTVNSQVNR.SLTAMQVLPTAANTEASSHRLGTGVVPALQAAETGASSNASDKNLIETRCVLNHHS
QYC65133.1    .....GDPIADMIDQTVNSQVNR.SLTAMQVLPTAANTEASSHRLGTGVVPALQAAETGASSNASDKNLIETRCVLNHHS
QYC65110.1    .....GDPIADMIDQTVNSQVNR.SLTAMQVLPTAANTEASSHRLGTGVVPALQAAETGASSNASDKNLIETRCVLNHHS
QYC65108.1    .....GDPIADMIDQTVNSQVNR.SLTAMQVLPTAANTEASSHRLGTGVVPALQAAETGASSNASDKNLIETRCVLNHHS
QYC65098.1    .....GDPIADMIDQTVNSQVNR.SLTAMQVLPTAANTEASSHRLGTGVVPALQAAETGASSNASDKNLIETRCVLNHHS
QYC65097.1    .....GDPIADMIDQTVNSQVNR.SLTAMQVLPTAANTEASSHRLGTGVVPALQAAETGASSNASDKNLIETRCVLNHHS
QYC65094.1    .....GDPIADMIDQTVNSQVNR.SLTAMQVLPTAANTEASSHRLGTGVVPALQAAETGASSNASDKNLIETRCVLNHHS
QYC65091.1    .....GDPIADMIDQTVNSQVNR.SLTAMQVLPTAANTEASSHRLGTGVVPALQAAETGASSNASDKNLIETRCVLNHHS
QYC65057.1    .....GDPIADMIDQTVNSQVNR.SLTAMQVLPTAANTEASSHRLGTGVVPALQAAETGASSNASDKNLIETRCVLNHHS
QWF08850.1    .....GDPIADMIDQTVNSQVNR.SLTAMQVLPTAANTEASSHRLGTGVVPALQAAETGASSNASDKNLIETRCVLNHHS
QYC65056.1    .....GDPIADMIDQTVNSQVNR.SLTAMQVLPTAANTEASSHRLGTGVVPALQAAETGASSNASDKNLIETRCVLNHHS
QWF08849.1    .....GDPIADMIDQTVNSQVNR.SLTAMQVLPTAANTEASSHRLGTGVVPALQAAETGASSNASDKNLIETRCVLNHHS
QWF08969.1    .....GDPIADMIDQTVNSQVNR.SLTAMQVLPTAANTEASSHRLGTGVVPALQAAETGASSNASDKNLIETRCVLNHHS
QWF08848.1    .....GDPIADMIDQTVNSQVNR.SLTAMQVLPTAANTEASSHRLGTGVVPALQAAETGASSNASDKNLIETRCVLNHHS
QWF08968.1    .....GDPIADMIDQTVNSQVNR.SLTAMQVLPTAANTEASSHRLGTGVVPALQAAETGASSNASDKNLIETRCVLNHHS
QWF08965.1    .....GDPIADMIDQTVNSQVNR.SLTAMQVLPTAANTEASSHRLGTGVVPALQAAETGASSNASDKNLIETRCVLNHHS
QWF08956.1    .....GDPIADMIDQTVNSQVNR.SLTAMQVLPTAANTEASSHRLGTGVVPALQAAETGASSNASDKNLIETRCVLNHHS
QWF08952.1    .....GDPIADMIDQTVNSQVNR.SLTAMQVLPTAANTEASSHRLGTGVVPALQAAETGASSNASDKNLIETRCVLNHHS
QWF08903.1    .....GDPIADMIDQTVNSQVNR.SLTAMQVLPTAANTEASSHRLGTGVVPALQAAETGASSNASDKNLIETRCVLNHHS
QWF08902.1    .....GDPIADMIDQTVNSQVNR.SLTAMQVLPTAANTEASSHRLGTGVVPALQAAETGASSNASDKNLIETRCVLNHHS
QWF08895.1    .....GDPIADMIDQTVNSQVNR.SLTAMQVLPTAANTEASSHRLGTGVVPALQAAETGASSNASDKNLIETRCVLNHHS
QWF08888.1    .....GDPIADMIDQTVNSQVNR.SLTAMQVLPTAANTEASSHRLGTGVVPALQAAETGASSNASDKNLIETRCVLNHHS
QWF08893.1    .....GDPIADMIDQTVNSQVNR.SLTAMQVLPTAANTEASSHRLGTGVVPALQAAETGASSNASDKNLIETRCVLNHHS
QWF08892.1    .....GDPIADMIDQTVNSQVNR.SLTAMQVLPTAANTEASSHRLGTGVVPALQAAETGASSNASDKNLIETRCVLNHHS
QWF08876.1    .....GDPIADMIDQTVNSQVNR.SLTAMQVLPTAANTEASSHRLGTGVVPALQAAETGASSNASDKNLIETRCVLNHHS
QWF08868.1    .....GDPIADMIDQTVNSQVNR.SLTAMQVLPTAANTEASSHRLGTGVVPALQAAETGASSNASDKNLIETRCVLNHHS
QWF08862.1    .....GDPIADMIDQTVNSQVNR.SLTAMQVLPTAANTEASSHRLGTGVVPALQAAETGASSNASDKNLIETRCVLNHHS
QWF08861.1    .....GDPIADMIDQTVNSQVNR.SLTAMQVLPTAANTEASSHRLGTGVVPALQAAETGASSNASDKNLIETRCVLNHHS
QWF08860.1    .....GDPIADMIDQTVNSQVNR.SLTAMQVLPTAANTEASSHRLGTGVVPALQAAETGASSNASDKNLIETRCVLNHHS
QWF08859.1    .....GDPIADMIDQTVNSQVNR.SLTAMQVLPTAANTEASSHRLGTGVVPALQAAETGASSNASDKNLIETRCVLNHHS
QWF08858.1    .....GDPIADMIDQTVNSQVNR.SLTAMQVLPTAANTEASSHRLGTGVVPALQAAETGASSNASDKNLIETRCVLNHHS
QWF08854.1    .....GDPIADMIDQTVNSQVNR.SLTAMQVLPTAANTEASSHRLGTGVVPALQAAETGASSNASDKNLIETRCVLNHHS
QWF08929.1    .....GDPIADMIDQTVNSQVNR.SLIAMQVLPTAANTEASSHRLGTGVVPALQAAETGASSNASDKNLIETRCVLNHHS
QYC65137.1    .....GDPIADMIDQTVNSQVNR.SLTAMQVLPTAANTEASSHRLGTGVVPALQAAETGASSNASDKNLIETRCVLNHHS
QYC65100.1    .....GDPIADMIDQTVNSQVNR.SLTAMQVLPTAANTEASSHRLGTGVVPALQAAETGASSNASDKNLIETRCVLNHHS
QWY15926.1    .....GDPIADMIDQTVNSQVNR.SLTAMQVLPTAANTEASSHRLGTGVVPALQAAETGASSNASDKNLIETRCVLNHHS
QWF08962.1    .....GDPIADMIDQTVNSQVNR.SLTAMQVLPTAANTEASSHRLGTGVVPALQAAETGASSNASDKNLIETRCVLNHHS
QWF08909.1    .....GDPIADMIDQTVNSQVNR.SLTAMQVLPTAANTEASSHRLGTGVVPALQAAETGASSNASDKNLIETRCVLNHHS
QWY15854.1    .....GDPIADMIDQTVNSQVNR.SLTAMQVLPTAANTEASSHRLGTGVVPALQAAETGASSNASDKNLIETRCVLNHHS
QWY15808.1    .....GDPIADMIDQAVNNQVNR.SLTAMQVLPTAANTEASSHRLGTGVVPALQAAETGASSNASDKNLIETRCVLNHHS
QWF08770.1    .....GDPIADMIDQAVNNQVNR.SLTAMQVLPTAANTEASSHRLGTGVVPALQAAETGASSNASDKNLIETRCVLNHHS
QWY15728.1    .....GDPIADMIDQAVNNQVNR.SLTAMQVLPTAANTEASSHRLGTGVVPALQAAETGASSNASDKNLIETRCVLNHHS
QYC65126.1    .....GDPIADMIDQAVNNQVNR.SLTAMQVLPTAANTEASSHRLGTGVVPALQAAETGASSNASDKNLIETRCVLNHHS
QWF08827.1    .....GDPIADMIDQAVNNQVNR.SLTAMQVLPTAANTEASSHRLGTGVVPALQAAETGASSNASDKNLIETRCVLNHHS
QYC65072.1    .....GDPIADMIDQAVNNQVNR.SLTAMQVLPTAANTEASSHRLGTGVVPALQAAETGASSNASDKNLIETRCVLNHHS
QYC65067.1    .....GDPIADMIDQAVNNQVNR.SLTAMQVLPTAANTEASSHRLGTGVVPALQAAETGASSNASDKNLIETRCVLNHHS
QWY15767.1    .....GDPIADMIDQAVNNQVNR.SLTAMQVLPTAANTEASSHRLGTGVVPALQAAETGASSNASDKNLIETRCVLNHHS
QWY15735.1    .....GDPIADMIDQAVNNQVNR.SLTAMQVLPTAANTEASSHRLGTGVVPALQAAETGASSNASDKNLIETRCVLNHHS
BCB92153.1    .....GDPIADMIDQAVNSQVNR.SLTAMQVLPTAANTEASSHRLGTGVVPALQAAETGASSNASDKNLIETRCVLNHHS
QWF08966.1    .....GDPIADMIDQTVNSQVNR.SLTAMQVLPTAANTEASSHRLGTGVVPALQAAETGASSNASDNNLIETRCVLNHHS
QIH53917.1    .....GDPIADMIDQTVNSQVNR.SLTAMQVLPTAANTEASSHRLGTGVVPALQAAETGASSNASDRNLIETRCVLNHHS
QYC65092.1    .....GDPIADMIDQTVNNQVNR.SLTAMQVLPTAANTEASSHRLGTGVVPALQAAETGASSNASDNNLIETRCVLNHHS
QWY15879.1    .....GDPIADMIDQTVNSQVNR.SLTAMQILPTAANTEASSHRLGTGVVPALQAAETGASSNASDKNLIETRCVLNHHS
QWY15777.1    .....GDPIADMIDQTVNSQVNR.SLTAMQILPTAANTEASSHRLGTGVVPALQAAETGASSNASDKNLIETRCVLNHHS
BCR43105.1    .....GDPIADMIDQTVNSQVNR.SLTAMQGLPTAANTEASSHRLGTGVVPALQAAETGASSNASDKNLIETRCVLNHHS
QWF08826.1    .....GDPIADMIDQTVNNQVNR.SLTAMQILPTAANTEASSHRLGTGVVPALQAAETGASSNASDKNLIETRCVLNHHS
BCG67024.1    .....GDPIADMIDQTVSKQVNR.SLTAMQVLPTAANTEASSHRLGTGVVPALQAAETGASSNASDKNLIETRCVLNHHS
BCG66999.1    .....GDPIADMIDQTVSKQVNR.SLTAMQVLPTAANTEASSHRLGTGVVPALQAAETGASSNASDKNLIETRCVLNHHS
QWY15752.1    .....GDPIADMIDQTVSNQVSR.SLTAMQVLPTAANTEASSHRLGTGVVPALQAAETGASSNASDKNLIETRCVLNHHS
BCG67028.1    .....GDPIADMIDQTVNKQVNR.SLTAMQVLPTAANTEASSHRLGTGVVPALQAAETGASSNASDKNLIETRCVLNHHS
BCG67027.1    .....GDPIADMIDQTVNKQVNR.SLTAMQVLPTAANTEASSHRLGTGVVPALQAAETGASSNASDKNLIETRCVLNHHS
BCG66996.1    .....GDPIADMIDQTVNKQVNR.SLTAMQVLPTAANTEASSHRLGTGVVPALQAAETGASSNASDKNLIETRCVLNHHS
BCG67026.1    .....GDPIADMIDQTVNKQVNR.SLTAMQVLPTAANTEASSHRLGTGVVPALQAAETGASSNASDKNLIETRCVLNHHS
BCG67025.1    .....GDPIADMIDQTVNKQVNR.SLTAMQVLPTAANTEASSHRLGTGVVPALQAAETGASSNASDKNLIETRCVLNHHS
BCG67023.1    .....GDPIADMIDQTVNKQVNR.SLTAMQVLPTAANTEASSHRLGTGVVPALQAAETGASSNASDKNLIETRCVLNHHS
BCG67022.1    .....GDPIADMIDQTVNKQVNR.SLTAMQVLPTAANTEASSHRLGTGVVPALQAAETGASSNASDKNLIETRCVLNHHS
BCG67021.1    .....GDPIADMIDQTVNKQVNR.SLTAMQVLPTAANTEASSHRLGTGVVPALQAAETGASSNASDKNLIETRCVLNHHS
BCG67013.1    .....GDPIADMIDQTVNKQVNR.SLTAMQVLPTAANTEASSHRLGTGVVPALQAAETGASSNASDKNLIETRCVLNHHS
BCG67000.1    .....GDPIADMIDQTVNKQVNR.SLTAMQVLPTAANTEASSHRLGTGVVPALQAAETGASSNASDKNLIETRCVLNHHS
BCG66997.1    .....GDPIADMIDQTVNKQVNR.SLTAMQVLPTAANTEASSHRLGTGVVPALQAAETGASSNASDKNLIETRCVLNHHS
BCG66998.1    .....GDPIADMIDQTVNKQVNR.SLTAMQVLPTAANTEASSHRLGTGVVPALQAAETGASSNASDKSLIETRCVLNHHS
QWF08924.1    .....GDPIADMIDQTVNGQVNR.SLTAMQVLPTAANTEASSHRLGTGVVPALQAAETGASSNASDKNLIETRCVLNHHS
QIL87522.1    .....GDPIADMIDQTVNGQVNR.SLTAMQVLPTAANTEASSHRLGTGVVPALQAAETGASSNASDKNLIETRCVLNHHS
QYC65075.1    .....GDPIADMIDQTVNTQVNR.SLTAMQVLPTAANTEASSHRLGTGVVPALQAAETGASSNASDKNLIETRCVLNHHS
QWF08781.1    .....GDPIADMIDQTVNDQVNR.SLTAMQVLPTAANTEASSHRLGTGVVPALQAAETGASSNASDKNLIETRCVLNHHS
QWY15817.1    .....GDPIADMIDQTVNSQVNR.SLTAMQVLPTAANTEASSHRLGTGVVPALQAAETGASSNASDKNLIETRCVLNHHS
QWF08897.1    .....GDPIADMIDQTVNSQVSR.SLTAMQVLPTAANTEASSHRLGTGVVPALQAAETGASSNASDKNLIETRCVLNHHS
QWY15815.1    .....GDPIADMIDQTVNNQVNR.SLTAMQVLPTAANTEASSHRLGTGVVPALQAAETGASSNASDKNLIETRCVLNHHS
QWY15814.1    .....GDPIADMIDQTVNNQVNR.SLTAMQVLPTAANTEASSHRLGTGVVPALQAAETGASSNASDKNLIETRCVLNHHS
QWY15718.1    .....GDPIADMIDQTVNNQVNR.SLTALQVLPTAANTEASSHRLGTGVVPALQAAETGASSNASDKNLIETRCVLNHHS
QYC65077.1    .....GDPIADMIDQTVNNQVNR.SLTAMQVLPTAANTEASSHRLGTGVVPALQAAETGASSNASDKNLIETRCVLNHHS
QTA73897.1    .....GDSIADMIDQTVNNQVNR.SLTAMQVLPTAANTEASSHRLGTGVVPALQAAETGASSNASDKNLIETRCVLNHHS
QWY15676.1    .....GDSIADMIDQTVNNQVNR.SLTAMQVLPTAANTEASSHRLGTGVVPALQAAETGASSNASDKNLIETRCVLNHHS
QOP74939.1    .....GDPIADMIDQTVNNQVNR.SLTAMQVLPTAANTEASSHRLGTGVVPALQAAETGASSNASDKNLIETRCVLNHHS
QOP74936.1    .....GDPIADMIDQTVNNQVNR.SLTAMQVLPTAANTEASSHRLGTGVVPALQAAETGASSNASDKNLIETRCVLNHHS
QWF08729.1    .....GDPIADMIDQTVNNQVNR.SLTAMQVLPTAANTEASSHRLGTGVVPALQAAETGASSNASDKNLIETRCVLNHHS
QYC65064.1    .....GDPIADMIDQTVNNQVNR.SLTAMQVLPTAANTEASSHRLGTGVVPALQAAETGASSNASDKNLIETRCVLNHHS
QWF08775.1    .....GDPIADMIDQTVNNQVNR.SLTAMQVLPTAANTEASSHRLGTGVVPALQAAETGASSNASDKNLIETRCVLNHHS
QWF08774.1    .....GDPIADMIDQTVNNQVNR.SLTAMQVLPTAANTEASSHRLGTGVVPALQAAETGASSNASDKNLIETRCVLNHHS
QWF08753.1    .....GDPIADMIDQTVNNQVNR.SLTAMQVLPTAANTEASSHRLGTGVVPALQAAETGASSNASDKNLIETRCVLNHHS
QWY15804.1    .....GDPIADMIDQTVNNQVNR.SLTAMQVLPTAANTEASSHRLGTGVVPALQAAETGASSNASDKNLIETRCVLNHHS
QOP74943.1    .....GDPIADMIDQTVNNQVNR.SLTAMQVLPTAANTEASSHRLGTGVVPALQAAETGASSNASDKNLIETRCVLNHHS
QIH53920.1    .....GDPIADMIDQTVNNQVNR.SLTAMQVLPTAANTEASSHRLGTGVVPALQAAETGASSNASDKNLIETRCVLNHHS
QIH53919.1    .....GDPIADMIDQTVNNQVNR.SLTAMQVLPTAANTEASSHRLGTGVVPALQAAETGASSNASDKNLIETRCVLNHHS
QIL87536.1    .....GDPIADMIDQTVNNQVNR.SLTAMQVLPTAANTEASSHRLGTGVVPALQAAETGASSNASDKNLIETRCVLNHHS
UEC49854.1    .....GDPIADMIDQTVNNQVNR.SLTAMQVLPTAANTEASSHRLGTGVVPALQAAETGASSNASDKNLIETRCVLNHHS
UEC49852.1    .....GDPIADMIDQTVNNQVNR.SLTAMQVLPTAANTEASSHRLGTGVVPALQAAETGASSNASDKNLIETRCVLNHHS
QWY15841.1    .....GDPIADMIDQTVNNQVNR.SLTAMQVLPTAANTEASSHRLGTGVVPALQAAETGASSNASDKNLIETRCVLNHHS
QWY15787.1    .....GDPIADMIDQTVNNQVNR.SLTAMQVLPTAANTEASSHRLGTGVVPALQAAETGASSNASDKNLIETRCVLNHHS
QYC65128.1    .....GDPIADMIDQTVNNQVNR.SLTAMQVLPTAANTEASSHRLGTGVVPALQAAETGASSNASDKNLIETRCVLNHHS
QYC65116.1    .....GDPIADMIDQTVNNQVNR.SLTAMQVLPTAANTEASSHRLGTGVVPALQAAETGASSNASDKNLIETRCVLNHHS
QYC65083.1    .....GDPIADMIDQTVNNQVNR.SLTAMQVLPTAANTEASSHRLGTGVVPALQAAETGASSNASDKNLIETRCVLNHHS
QYC65029.1    .....GDPIADMIDQTVNNQVNR.SLTAMQVLPTAANTEASSHRLGTGVVPALQAAETGASSNASDKNLIETRCVLNHHS
QWF08951.1    .....GDPIADMIDQTVNNQVNR.SLTAMQVLPTAANTEASSHRLGTGVVPALQAAETGASSNASDKNLIETRCVLNHHS
QWF08937.1    .....GDPIADMIDQTVNNQVNR.SLTAMQVLPTAANTEASSHRLGTGVVPALQAAETGASSNASDKNLIETRCVLNHHS
QWF08918.1    .....GDPIADMIDQTVNNQVNR.SLTAMQVLPTAANTEASSHRLGTGVVPALQAAETGASSNASDKNLIETRCVLNHHS
QWF08901.1    .....GDPIADMIDQTVNNQVNR.SLTAMQVLPTAANTEASSHRLGTGVVPALQAAETGASSNASDKNLIETRCVLNHHS
QWF08834.1    .....GDPIADMIDQTVNNQVNR.SLTAMQVLPTAANTEASSHRLGTGVVPALQAAETGASSNASDKNLIETRCVLNHHS
QWF08832.1    .....GDPIADMIDQTVNNQVNR.SLTAMQVLPTAANTEASSHRLGTGVVPALQAAETGASSNASDKNLIETRCVLNHHS
QWF08802.1    .....GDPIADMIDQTVNNQVNR.SLTAMQVLPTAANTEASSHRLGTGVVPALQAAETGASSNASDKNLIETRCVLNHHS
QWF08800.1    .....GDPIADMIDQTVNNQVNR.SLTAMQVLPTAANTEASSHRLGTGVVPALQAAETGASSNASDKNLIETRCVLNHHS
QYC65123.1    .....GDPIADMIDQTVNNQVNR.SLTAMQVLPTAANTEASSHRLGTGVVPALQAAETGASSNASDKNLIETRCVLNHHS
QWY15820.1    .....GDPIADMIDQTVNNQVNR.SLTAMQVLPTAANTEASSHRLGTGVVPALQAAETGASSNASDKNLIETRCVLNHHS
QWY15864.1    .....GDPIADMIDQTVNNQVNR.SLTAMQVLPTAANTEASSHRLGTGVVPALQAAETGASSNASDKNLIETRCVLNHHS
QWY15840.1    .....GDPIADMIDQTVNNQVNR.SLTAMQVLPTAANTEASSHRLGTGVVPALQAAETGASSNASDKNLIETRCVLNHHS
QWY15786.1    .....GDPIADMIDQTVNNQVNR.SLTAMQVLPTAANTEASSHRLGTGVVPALQAAETGASSNASDKNLIETRCVLNHHS
QWY15842.1    .....GDPIADMIDQTVNNQVNR.SLTAMQVLPTAANTEASSHRLGTGVVPALQAAETGASSNASDKNLIETRCVLNHHS
QWY15704.1    .....GDPIADMIDQTVNNQVNR.SLTALQVLPTAANTEASSHRLGTGVVPALQAAETGASSNASDKNLIETRCVLNHHS
QWY15750.1    .....GDPIADMIDQTVNNQVNR.SLTALQVLPTAANTEASSHRLGTGVVPALQAAETGASSNASDKNLIETRCVLNHHS
QWF08733.1    .....GDPIADMIDQTMSNQVNR.SLTALQVLPTAANTEASSHRLGTGVVPALQAAETGASSNASDKNLIETRCVLNHHS
QNL13228.1    .....GDSIADMIDQTVNNQVNR.SLTALQVLPTAANTEASSHRLGTGVVPALQAAETGASSNASDKNLIETRCVLNHHS
QNL13216.1    .....GDSIADMIDQTVNNQVNR.SLTALQVLPTAANTEASSPRLGTGVVPALQAAETGASSNASDKNLIEPRCVLNHHS
QNL13226.1    .....GDSIADMIDQTVNNQVNR.SLTALQVLPTAANTEASSHRLGTGVVPALQAAETGASSNASDKNLIETRCVLNHHS
QNL13203.1    .....GDSIADMIDQTVNNQVNR.SLTALQVLPTAANTEASSHRLGTGVVPALQAAETGASSNASDKNLIETRCVLNHHS
```

```
QYC65080.1      .....GDSIADMIDQTVNNQVNR.SLTALQVLPTAANTEASSHRLGTGVVPALQAAETGASSNASDKNLIETRCVLNHHS
QNL13225.1      .....GDSIADMIDQTVNNQVNR.SLTALQVLPTAANTEASSHRLGTGVVPALQAAETGASSNASDKNLIETRCVLNHHS
QNL13224.1      .....GDSIADMIDQTVNNQVNR.SLTALQVLPTAANTEASSHRLGTGVVPALQAAETGASSNASDKNLIETRCVLNHHS
QNL13223.1      .....GDSIADMIDQTVNNQVNR.SLTALQVLPTAANTEASSHRLGTGVVPALQAAETGASSNASDKNLIETRCVLNHHS
QNL13222.1      .....GDSIADMIDQTVNNQVNR.SLTALQVLPTAANTEASSHRLGTGVVPALQAAETGASSNASDKNLIETRCVLNHHS
QNL13221.1      .....GDSIADMIDQTVNNQVNR.SLTALQVLPTAANTEASSHRLGTGVVPALQAAETGASSNASDKNLIETRCVLNHHS
QNL13220.1      .....GDSIADMIDQTVNNQVNR.SLTALQVLPTAANTEASSHRLGTGVVPALQAAETGASSNASDKNLIETRCVLNHHS
QNL13219.1      .....GDSIADMIDQTVNNQVNR.SLTALQVLPTAANTEASSHRLGTGVVPALQAAETGASSNASDKNLIETRCVLNHHS
QWF08894.1      .....GDSIADMIDQTVNNQVNR.SLTALQVLPTAANTEASSHRLGTGVVPALQAAETGASSNASDKNLIETRCVLNHHS
QNL13218.1      .....GDSIADMIDQTVNNQVNR.SLTALQVLPTAANTEASSHRLGTGVVPALQAAETGASSNASDKNLIETRCVLNHHS
QNL13217.1      .....GDSIADMIDQTVNNQVNR.SLTALQVLPTAANTEASSHRLGTGVVPALQAAETGASSNASDKNLIETRCVLNHHS
QNL13215.1      .....GDSIADMIDQTVNNQVNR.SLTALQVLPTAANTEASSHRLGTGVVPALQAAETGASSNASDKNLIETRCVLNHHS
QNL13214.1      .....GDSIADMIDQTVNNQVNR.SLTALQVLPTAANTEASSHRLGTGVVPALQAAETGASSNASDKNLIETRCVLNHHS
QNL13213.1      .....GDSIADMIDQTVNNQVNR.SLTALQVLPTAANTEASSHRLGTGVVPALQAAETGASSNASDKNLIETRCVLNHHS
QNL13212.1      .....GDSIADMIDQTVNNQVNR.SLTALQVLPTAANTEASSHRLGTGVVPALQAAETGASSNASDKNLIETRCVLNHHS
QNL13209.1      .....GDSIADMIDQTVNNQVNR.SLTALQVLPTAANTEASSHRLGTGVVPALQAAETGASSNASDKNLIETRCVLNHHS
QIL87537.1      .....GDSIADMIDQTVNNQVNR.SLTALQVLPTAANTEASSHRLGTGVVPALQAAETGASSNASDKNLIETRCVLNHHS
QNL13208.1      .....GDSIADMIDQTVNNQVNR.SLTALQVLPTAANTEASSHRLGTGVVPALQAAETGASSNASDKNLIETRCVLNHHS
QNL13207.1      .....GDSIADMIDQTVNNQVNR.SLTALQVLPTAANTEASSHRLGTGVVPALQAAETGASSNASDKNLIETRCVLNHHS
QNL13206.1      .....GDSIADMIDQTVNNQVNR.SLTALQVLPTAANTEASSHRLGTGVVPALQAAETGASSNASDKNLIETRCVLNHHS
QNL13202.1      .....GDSIADMIDQTVNNQVNR.SLTALQVLPTAANTEASSHRLGTGVVPALQAAETGASSNASDKNLIETRCVLNHHS
QNL13196.1      .....GDSIADMIDQTVNNQVNR.SLTALQVLPTAANTEASSHRLGTGVVPALQAAETGASSNASDKNLIETRCVLNHHS
QNL13195.1      .....GDSIADMIDQTVNNQVNR.SLTALQVLPTAANTEASSHRLGTGVVPALQAAETGASSNASDKNLIETRCVLNHHS
QNL13194.1      .....GDSIADMIDQTVNNQVNR.SLTALQVLPTAANTEASSHRLGTGVVPALQAAETGASSNASDKNLIETRCVLNHHS
QNL13193.1      .....GDSIADMIDQTVNNQVNR.SLTALQVLPTAANTEASSHRLGTGVVPALQAAETGASSNASDKNLIETRCVLNHHS
QNL13192.1      .....GDSIADMIDQTVNNQVNR.SLTALQVLPTAANTEASSHRLGTGVVPALQAAETGASSNASDKNLIETRCVLNHHS
QNL13191.1      .....GDSIADMIDQTVNNQVNR.SLTALQVLPTAANTEASSHRLGTGVVPALQAAETGASSNASDKNLIETRCVLNHHS
QNL13190.1      .....GDSIADMIDQTVNNQVNR.SLTALQVLPTAANTEASSHRLGTGVVPALQAAETGASSNASDKNLIETRCVLNHHS
QNL13189.1      .....GDSIADMIDQTVNNQVNR.SLTALQVLPTAANTEASSHRLGTGVVPALQAAETGASSNASDKNLIETRCVLNHHS
QNL13188.1      .....GDSIADMIDQTVNNQVNR.SLTALQVLPTAANTEASSHRLGTGVVPALQAAETGASSNASDKNLIETRCVLNHHS
QCY53846.1      .....GDSIADMIDQTVNNQVNR.SLTALQVLPTAANTEASSHRLGTGVVPALQAAETGASSNASDKNLIETRCVLNHHS
BCI65357.1      .....GDSIADMIDQTVNNQVNR.SLTALQVLPTAANTEASSHRLGTGVVPALQAAETGASSNASDKNLIETRCVLNHHS
QNL13227.1      .....GDSIADMIDQTVNNQVNR.SLTALQVLPTAANTEASSHRLGTGVVPALQAAETGASSNASDKNLIETRCVLNHHS
QNL13211.1      .....GDSIADMIDQTVNNQVNR.SLTALQVLPTAANTEASSHRLGTGVVPALQAAETGASSNASDKNLIETRCVLNHHS
QNL13198.1      .....GDSIANMIDQTVNNQVNR.SLTALQVLPTAANTEASSHRLGTGVVPALQAAETGASSNASDKNLIETRCVLNHHS
QNL13197.1      .....GDSIANMIDQTVNNQVNR.SLTALQVLPTAANTEASSHRLGTGVVPALQAAETGASSNASDKNLIETRCVLNHHS
QNL13200.1      .....GDSIANMIDQTVNNQVNR.SLTALQVLSTAANTEASSHRLGTGVVPALQAAETGASSNASDKNLIETRCVLNHHS
QNL13210.1      .....GDSIADMIDQAVNNQVNR.SLTALQVLPTAANTEASSHRLGTGVVPALQAAETGASSNASDKNLIETRCVLNHHS
QNL13205.1      .....GDSIADMIDQTVNSQVNR.SLTALQVLPTAANTEASSHRLGTGVVPALQAAETGASSNASDKNLIETRCVLNHHS
QNL13204.1      .....GDSIADMIDQTVNSQVNR.SLTALQVLPTAANTEASSHRLGTGVVPALQAAETGASSNASDKNLIETRCVLNHHS
QNL13201.1      .....GDPIADMIDQTVNNQVNR.SLTALQVLPTAANTEASSHRLGTGVVPALQAAETGASSNASDKNLIETRCVLNHHS
QNL13199.1      .....GDPIADMIDQTVNNQVNR.SLTALQVLPTAANTEASSHRLGTGVVPALQAAETGASSNASDKNLIETRCVLNHHS
QYC65157.1      .....GDPIADMIDQTVNNQVNR.SLTAMQVLPTAANTEASSHRLGTGVVPALQAAETGASSNASDKNLIETRCVLNHHS
QYC65138.1      .....GDPIADMIDQTVNNQVNR.SLTAMQVLPTAANTEASSHRLGTGVVPALQAAETGASSNASDKNLIETRCVLNHHS
QWF08724.1      .....GDPIADMIDQTVNNQVNR.SLTAMQVLPTAANTEASSHRLGTGVVPALQAAETGASSNASDKNLIETRCVLNHHS
UEP53403.1      .....GDPIADMIDQAVNNQVNR.SLTAMQVLPTAANTEASSHRLGTGVVPALQAAETGASSNASDKNLIETRCVLNHHS
QWY15843.1      .....GDPIADMIDQTVNNQVNR.SLTAMQVLPTAANTEASSHRLGTGVVPALQAAETGASSNASDKNLIETRCVLNHHS
QWF08794.1      .....GDSIADMIDQTVNNQVNR.SLTAMQVLPTAANTEASSHRLGTGVVPALQAAETGASSNASDKNLIETRCVLNHHS
QYC65031.1      .....GDSIADMIDQTVNNQVNR.SLTALQVLPTAANTEASSHRLGTGVVPALQAAETGASSNASDKNLIETRCVLNHHS
UEC49856.1      .....GDSIADMIDQTVNNQVNR.SLTAMQVLPTAANTEASSHRLGTGVVPALQAAETGASSNASDKNLIETRCVLNHHS
BBH72184.1      .....GDSIADMIDQTVNNQVNR.SLTAMQVLPTAANTEASSHRLGTGVVPALQAAETGASSNASDKNLIETRCVLNHHS
QWF08874.1      .....GDSIADMIDQTVNNQVNR.SLTAMQVLPTAANTEASSHRLGTGVVPALQAAETGASSNASDKNLIETRCVLNHHS
QWF08873.1      .....GDSIADMIDQTVNNQVNR.SLTAMQVLPTAANTEASSHRLGTGVVPALQAAETGASSNASDKNLIETRCVLNHHS
BCG67003.1      .....GDSIADMIDQTVNKQVNR.SLTAMQVLPTAANTEASSHRLGTGVVPALQAAETGASSNASDKNLIETRCVLNHHS
QYC65095.1      .....GDLIADMIDQTVNNQVNR.SLTAMQVLPTAANTEASSHRLGTGVVPALQAAETGASSNASDKNLIETRCVLNHHS
QOP74933.1      .....GDPIADMIDQTVNNQVNR.SLTALQVLPTAANTEASSHRLGTGVVPALQAAETGASSNASDKNLVETRCVLNHHS
QYC65096.1      .....GDPIADMIDQTVNNQVNR.SLTAMQVLPTAANTEASSHRLGTGVVPALQAAETGASSNASDKNLVETRCVLNHHS
BCG67034.1      .....GDPIADMIDQTVNNQVNR.SLTAVQVLPTAANTEASSHRLGTGVVPALQAAETGASSNASDKNLIETRCVLNHHS
BCG67031.1      .....GDPIADMIDQTVNNQVNR.SLTAVQVLPTAANTEASSHRLGTGVVPALQAAETGASSNASDKNLIETRCVLNHHS
QYC65052.1      .....GDPIADMIDQTVNNQVNR.SLTATQVLPTAANTEASSHRLGTGVVPALQAAETGASSNASDKNLIETRCVLNHHS
QWF08963.1      .....GDPIADMIDQTVNSQVNR.SLTAIQVLPTAANTEASSHRLGTGVVPALQAAETGASSNASDKNLIETRCVLNHHS
QWF08939.1      .....GDPIADMIDQTVNSQVNR.SLTAIQVLPTAANTEASSHRLGTGVVPALQAAETGASSNASDKNLIETRCVLNHHS
QIL87538.1      .....GDPIADMIDQTVNSQVNR.SLTAIQVLPTAANTEASSHRLGTGVVPALQAAETGASSNASDKNLIETRCVLNHHS
QIL87535.1      .....GDPIADMIDQTVNSQVNR.SLTAIQVLPTAANTEASSHRLGTGVVPALQAAETGASSNASDKNLIETRCVLNHHS
QWF08964.1      .....GDPIADMIDQTVNSQVNR.SLTAIQVLPTAANTEASSHRLGTGVVPALQAAETGASSNASDKNLIETRCVLNHHS
QYC65132.1      .....GDPIADMIDQTVNNQVNR.SLTAIQVLPTAANTEASSHRLGTGVVPALQAAETGASSNASDKNLIETRCVLNHHS
QTA73893.1      .....GDPIADMIDQTVNNQVNR.SLTALQVLPTAANTEASSHRLGTGVVPALQAAETGASSNASDKNLIETRCVLNHHS
QWF08751.1      .....GDPIADMIDQTVNNQVNR.SLTALQVLPTAANTEASSHRLGTGVVPALQAAETGASSNASDKNLIETRCVLNHHS
QWY15860.1      .....GDPIADMIDQTVNNQVNR.SLTALQVLPTAANTEASSHRLGTGVVPALQAAETGASSNASDKNLIETRCVLNHHS
QWY15749.1      .....GDPIADMIDQTVNNQVNR.SLTALQVLPTAANTEASSHRLGTGVVPALQAAETGASSNASDKNLIETRCVLNHHS
QWY15744.1      .....GDPIADMIDQTVNNQVNR.SLTALQVLPTAANTEASSHRLGTGVVPALQAAETGASSNASDKNLIETRCVLNHHS
QWY15740.1      .....GDPIADMIDQTVNNQVNR.SLTALQVLPTAANTEASSHRLGTGVVPALQAAETGASSNASDKNLIETRCVLNHHS
QWY15700.1      .....GDPIADMIDQTVNNQVNR.SLTALQVLPTAANTEASSHRLGTGVVPALQAAETGASSNASDKNLIETRCVLNHHS
QWY15698.1      .....GDPIADMIDQTVNNQVNR.SLTALQVLPTAANTEASSHRLGTGVVPALQAAETGASSNASDKNLIETRCVLNHHS
QWY15672.1      .....GDPIADMIDQTVNNQVNR.SLTALQVLPTAANTEASSHRLGTGVVPALQAAETGASSNASDKNLIETRCVLNHHS
pdb|6LHT|B      .....GDPIADMIDQTVNNQVNR.SLTALQVLPTAANTEASSHRLGTGVVPALQAAETGASSNASDKNLIETRCVLNHHS
pdb|6LHT|E      .....GDPIADMIDQTVNNQVNR.SLTALQVLPTAANTEASSHRLGTGVVPALQAAETGASSNASDKNLIETRCVLNHHS
pdb|6LHT|D      .....GDPIADMIDQTVNNQVNR.SLTALQVLPTAANTEASSHRLGTGVVPALQAAETGASSNASDKNLIETRCVLNHHS
pdb|6LHT|C      .....GDPIADMIDQTVNNQVNR.SLTALQVLPTAANTEASSHRLGTGVVPALQAAETGASSNASDKNLIETRCVLNHHS
pdb|6LHT|A      .....GDPIADMIDQTVNNQVNR.SLTALQVLPTAANTEASSHRLGTGVVPALQAAETGASSNASDKNLIETRCVLNHHS
pdb|6LHQ|A      .....GDPIADMIDQTVNNQVNR.SLTALQVLPTAANTEASSHRLGTGVVPALQAAETGASSNASDKNLIETRCVLNHHS
pdb|6LHP|A      .....GDPIADMIDQTVNNQVNR.SLTALQVLPTAANTEASSHRLGTGVVPALQAAETGASSNASDKNLIETRCVLNHHS
pdb|6LHO|A      .....GDPIADMIDQTVNNQVNR.SLTALQVLPTAANTEASSHRLGTGVVPALQAAETGASSNASDKNLIETRCVLNHHS
pdb|6LHL|A      .....GDPIADMIDQTVNNQVNR.SLTALQVLPTAANTEASSHRLGTGVVPALQAAETGASSNASDKNLIETRCVLNHHS
pdb|6LHK|A      .....GDPIADMIDQTVNNQVNR.SLTALQVLPTAANTEASSHRLGTGVVPALQAAETGASSNASDKNLIETRCVLNHHS
pdb|6LHC|A      .....GDPIADMIDQTVNNQVNR.SLTALQVLPTAANTEASSHRLGTGVVPALQAAETGASSNASDKNLIETRCVLNHHS
pdb|6LHB|A      .....GDPIADMIDQTVNNQVNR.SLTALQVLPTAANTEASSHRLGTGVVPALQAAETGASSNASDKNLIETRCVLNHHS
pdb|6LHA|A      .....GDPIADMIDQTVNNQVNR.SLTALQVLPTAANTEASSHRLGTGVVPALQAAETGASSNASDKNLIETRCVLNHHS
QWY15708.1      .....GDPIADMIDQTVNNQVDR.SLTALQVLPTAANTEASSHRLGTGVVPALQAAETGASSNASDKNLIETRCVLNHHS
BBH72101.1      .....GDPIGDMIYQTGNNQQNR.SLTALQVLPTAANTEASSHRLGTGVVPALQAAETGASSNASDKNLIETRCVLNHHS
BBH72090.1      .....GDPIGNMIYQTVNNQQNR.SLTALQVLPTAANTEASSHRLGTGVVPALQAAETGASSNASDKNLIETRCVLNHHS
BBH72097.1      ......DPIGNMIDQTVNNQENR.SLTALQVLPTAANTEASSHRLGTGVVPALQAAETGASSNASDKNLIETRCVLNHHS
BBH72089.1      .....GDPIGDMIHQTVNNQVNR.SLTALQVLPTAANTEASSHRLGTGVVPALQAAETGASSNASDKNLIETRCVLNHHS
BBH72099.1      .....GDPIADMIDQTVNNQVNR.SLTALQVLPTAANTEASSHRLGTGVVPALQAAETGASSNASDKNLIETRCVLNHHS
BBH72093.1      .....GDPIADMIDQTVNNQVNR.SLTALQVLPTAANTEASSHRLGTGVVPALQAAETGASSNASDKNLIETRCVLNHHS
BBH72096.1      .....GDPIADMIDQTVNNQVNR.SLTALQVLPTAANTEASSHRLGTGVVPALQAAETGASSNASDKNLIETRCVLNHHS
BBH72094.1      .....GDPIADMIDQTVNNQVNR.SLTALQVLPTAANTEASSHRLGTGVVPALQAAETGASSNASDKNLIETRCVLNHHS
BBH72091.1      .....GDPIADMIDQTVNNQVNR.SLTALQVLPTAANTEASSHRLGTGVVPALQAAETGASSNASDKNLIETRCVLNHHS
QOP74916.1      .....GDPIADMIDQTVNNQVNR.SLTALQVLPTAANTEASSHRLGTGVVPALQAAETGASSNASDKNLIETRCVLNHHS
QYC65163.1      .....GDPIADMIDQTVNNQVNR.SLTALQVLPTAANTEASSHRLGTGVVPALQAAETGASSNASDKNLIETRCVLNHHS
QWF08736.1      .....GDPIADMIDQTVNNQVNR.SLTALQVLPTAANTEASSHRLGTGVVPALQAAETGASSNASDKNLIETRCVLNHHS
QWF08735.1      .....GDPIADMIDQTVNNQVNR.SLTALQVLPTAANTEASSHRLGTGVVPALQAAETGASSNASDKNLIETRCVLNHHS
pdb|5TSK|A      .....GDPIADMIDQTVNNQVNR.SLTALQVLPTAANTEASSHRLGTGVVPALQAAETGASSNASDKNLIETRCVLNHHS
pdb|5C9A|A      .....GDPIADMIDQTVNNQVNR.SLTALQVLPTAANTEASSHRLGTGVVPALQAAETGASSNASDKNLIETRCVLNHHS
pdb|5C4W|A      .....GDPIADMIDQTVNNQVNR.SLTALQVLPTAANTEASSHRLGTGVVPALQAAETGASSNASDKNLIETRCVLNHHS
pdb|4JGZ|A      .....GDPIADMIDQTVNNQVNR.SLTALQVLPTAANTEASSHRLGTGVVPALQAAETGASSNASDKNLIETRCVLNHHS
pdb|4JGY|A      .....GDPIADMIDQTVNNQVNR.SLTALQVLPTAANTEASSHRLGTGVVPALQAAETGASSNASDKNLIETRCVLNHHS
QWY15956.1      .....GDPIADMIDQTVNNQVNR.SLTALQVLPTAANTEASSHRLGTGVVPALQAAETGASSNASDKNLIETRCVLNHHS
QWF08811.1      .....GDPIADMIDQTVNNQVNR.SLSALQVLPTAANTEASSHRLGTGVVPALQAAETGASSNASDKNLIETRCVLNHHS
QWF08878.1      .....GDPIADMIDQTVNNQVNR.SLSALQVLPTAANTEASSHRLGTGVVPALQAAETGASSNASDKNLIETRCVLNHHS
QWF08869.1      .....GDPIADMIDQTVNNQVNR.SLSALQVLPTAANTEASSHRLGTGVVPALQAAETGASSNASDKNLIETRCVLNHHS
QWF08831.1      .....GDPIADMIDQTVNNQVNR.SLSALQVLPTAANTEASSHRLGTGVVPALQAAETGASSNASDKNLIETRCVLNHHS
QWF08829.1      .....GDPIADMIDQTVNNQVNR.SLSALQVLPTAANTEASSHRLGTGVVPALQAAETGASSNASDKNLIETRCVLNHHS
QWF08824.1      .....GDPIADMIDQTVNNQVNR.SLSALQVLPTAANTEASSHRLGTGVVPALQAAETGASSNASDKNLIETRCVLNHHS
QWF08822.1      .....GDPIADMIDQTVNNQVNR.SLSALQVLPTAANTEASSHRLGTGVVPALQAAETGASSNASDKNLIETRCVLNHHS
QWF08817.1      .....GDPIADMIDQTVNNQVNR.SLSALQVLPTAANTEASSHRLGTGVVPALQAAETGASSNASDKNLIETRCVLNHHS
QWF08816.1      .....GDPIADMIDQTVNNQVNR.SLSALQVLPTAANTEASSHRLGTGVVPALQAAETGASSNASDKNLIETRCVLNHHS
QWF08798.1      .....GDPIADMIDQTVNNQVNR.SLSALQVLPTAANTEASSHRLGTGVVPALQAAETGASSNASDKNLIETRCVLNHHS
QWF08779.1      .....GDPIADMIDQTVNNQVNR.SLSALQVLPTAANTEASSHRLGTGVVPALQAAETGASSNASDKNLIETRCVLNHHS
QWF08778.1      .....GDPIADMIDQTVNNQVNR.SLSALQVLPTAANTEASSHRLGTGVVPALQAAETGASSNASDKNLIETRCVLNHHS
QWF08772.1      .....GDPIADMIDQTVNNQVNR.SLSALQVLPTAANTEASSHRLGTGVVPALQAAETGASSNASDKNLIETRCVLNHHS
QWF08771.1      .....GDPIADMIDQTVNNQVNR.SLSALQVLPTAANTEASSHRLGTGVVPALQAAETGASSNASDKNLIETRCVLNHHS
QWF08745.1      .....GDPIADMIDQTVNNQVNR.SLSALQVLPTAANTEASSHRLGTGVVPALQAAETGASSNASDKNLIETRCVLNHHS
QWF08744.1      .....GDPIADMIDQTVNNQVNR.SLSALQVLPTAANTEASSHRLGTGVVPALQAAETGASSNASDKNLIETRCVLNHHS
QIH53922.1      .....GDPIADMIDQTVNNQVNR.SLSALQVLPTAANTEASSHRLGTGVVPALQAAETGASSNASDKNLIETRCVLNHHS
QOP74941.1      .....GDPIADMIDQTVNNQVSR.SLSALQVLPTAANTEASSHRLGTGVVPALQAAETGASSNASDKNLIETRCVLNHHS
QWY15812.1      .....GDPIADMIDQTVNNQVNR.SLTALQVLPTAANTEASSHRLGTGIVPALQAAETGASSNASDKNLIETRCVLNHHS
QOP74926.1      .....GDPIADMIDQTVNNQVNR.SLTALQVLPTAANTEASSHRLGTGIVPALQAAETGASSNASDKNLIETRCVLNHHS
QYC65176.1      .....GDPIADMIDQTVNNQVNR.SLTALQVLPTAANTEASSHRLGTGIVPALQAAETGASSNASDKNLIETRCVLNHHS
QYC65172.1      .....GDPIADMIDQTVNNQVNR.SLTALQVLPTAANTEASSHRLGTGIVPALQAAETGASSNASDKNLIETRCVLNHHS
QYC65168.1      .....GDPIADMIDQTVNNQVNR.SLTALQVLPTAANTEASSHRLGTGIVPALQAAETGASSNASDKNLIETRCVLNHHS
QWF08725.1      .....GDPIADMIDQTVNNQVNR.SLTALQVLPTAANTEASSHRLGTGIVPALQAAETGASSNASDKNLIETRCVLNHHS
QYC65028.1      .....GDPIADMIDQTVNNQVSR.SLTALQVLPTAANTEASSHRLGTGIVPALQAAETGASSNASDKNLIETRCVLNHHS
QWY15686.1      .....GDPIADMIDQTVNNQVNR.SLTALQVLPTAANTEASSHRLGTGVVPALQAAETGASSNASDKNLIETRCVLNHHS
QYC65023.1      .....GDPIADMIDQTVNNQVNR.SLTALQVLPTAANTEASSHRLGTGIVPAVQAAETGASSNASDKNLIETRCVLNHHS
QYC65025.1      .....GDPIADMIDQTVNNQVNR.SLTALQVLPTAANTEASSHRLGTGVVPAMQAAETGASSNASDKNLIETRCVLNHHS
QWY15946.1      .....GDPIADMIDQTVNNQVNR.SLTALQVLPTAANTEASSHRLGTGVVPALQAAETGASSNASDKNLIETRCVLNHHS
BCB92164.1      .....GDPIADMIDQTVNNQVNR.SLTALQVLPTAANTEASSHRLGTGVVPALQAAETGASSNASDKNLIETRCVLNHHS
QOP74935.1      .....GDPIADMIDQTVNNQVNR.SLTALQVLPTAANTEASSHRLGTGVVPALQAAETGASSNASDKNLIETRCVLNHHS
QOP74930.1      .....GDPIADMIDQTVNNQVNR.SLTALQVLPTAANTEASSHRLGTGVVPALQAAETGASSNASDKNLIETRCVLNHHS
QOP74928.1      .....GDPIADMIDQTVNNQVNR.SLTALQVLPTAANTEASSHRLGTGVVPALQAAETGASSNASDKNLIETRCVLNHHS
QOP74927.1      .....GDPIADMIDQTVNNQVNR.SLTALQVLPTAANTEASSHRLGTGVVPALQAAETGASSNASDKNLIETRCVLNHHS
QOP74925.1      .....GDPIADMIDQTVNNQVNR.SLTALQVLPTAANTEASSHRLGTGVVPALQAAETGASSNASDKNLIETRCVLNHHS
QOP74923.1      .....GDPIADMIDQTVNNQVNR.SLTALQVLPTAANTEASSHRLGTGVVPALQAAETGASSNASDKNLIETRCVLNHHS
```

```
QOP74922.1    .....GDPIADMIDQTVNNQVNR.SLTALQVLPTAANTEASSHRLGTGVVPALQAAETGASSNASDKNLIETRCVLNHHS
QOP74920.1    .....GDPIADMIDQTVNNQVNR.SLTALQVLPTAANTEASSHRLGTGVVPALQAAETGASSNASDKNLIETRCVLNHHS
QOP74919.1    .....GDPIADMIDQTVNNQVNR.SLTALQVLPTAANTEASSHRLGTGVVPALQAAETGASSNASDKNLIETRCVLNHHS
QOP74917.1    .....GDPIADMIDQTVNNQVNR.SLTALQVLPTAANTEASSHRLGTGVVPALQAAETGASSNASDKNLIETRCVLNHHS
QHB49260.1    .....GDPIADMIDQTVNNQVNR.SLTALQVLPTAANTEASSHRLGTGVVPALQAAETGASSNASDKNLIETRCVLNHHS
QWF08784.1    .....GDPIADMIDQTVNNQVNR.SLTALQVLPTAANTEASSHRLGTGVVPALQAAETGASSNASDKNLIETRCVLNHHS
QWF08782.1    .....GDPIADMIDQTVNNQVNR.SLTALQVLPTAANTEASSHRLGTGVVPALQAAETGASSNASDKNLIETRCVLNHHS
QWF08777.1    .....GDPIADMIDQTVNNQVNR.SLTALQVLPTAANTEASSHRLGTGVVPALQAAETGASSNASDKNLIETRCVLNHHS
QWF08757.1    .....GDPIADMIDQTVNNQVNR.SLTALQVLPTAANTEASSHRLGTGVVPALQAAETGASSNASDKNLIETRCVLNHHS
QWF08756.1    .....GDPIADMIDQTVNNQVNR.SLTALQVLPTAANTEASSHRLGTGVVPALQAAETGASSNASDKNLIETRCVLNHHS
QWF08755.1    .....GDPIADMIDQTVNNQVNR.SLTALQVLPTAANTEASSHRLGTGVVPALQAAETGASSNASDKNLIETRCVLNHHS
QWF08791.1    .....GDPIADMIDQTVNNQVNR.SLTALQVLPTAANTEASSHRLGTGVVPALQAAETGASSNASDKNLIETRCVLNHHS
QWF08786.1    .....GDPIADMIDQTVNNQVNR.SLTALQVLPTAANTEASSHRLGTGVVPALQAAETGASSNASDKNLIETRCVLNHHS
QYC65186.1    .....GDPIADMIDQTVNNQVNR.SLTALQVLPTAANTEASSHRLGTGVVPALQAAETGASSNASDKNLIETRCVLNHHS
QWY15722.1    .....GDPIADMIDQTVNNQVNR.SLTALQVLPTAANTEASSHRLGTGVVPALQAAETGASSNASDKNLIETRCVLNHHS
QYC65182.1    .....GDPIADMIDQTVNNQVNR.SLTALQVLPTAANTEASSHRLGTGVVPALQAAETGASSNASDKNLIETRCVLNHHS
QWF08754.1    .....GDPIADMIDQTVNNQVNR.SLTALQVLPTAANTEASSHRLGTGVVPALQAAETGASSNASDKNLIETRCVLNHHS
QWF08748.1    .....GDPIADMIDQTVNNQVNR.SLTALQVLPTAANTEASSHRLGTGVVPALQAAETGASSNASDKNLIETRCVLNHHS
QYC65181.1    .....GDPIADMIDQTVNNQVNR.SLTALQVLPTAANTEASSHRLGTGVVPALQAAETGASSNASDKNLIETRCVLNHHS
QWY15719.1    .....GDPIADMIDQTVNNQVNR.SLTALQVLPTAANTEASSHRLGTGVVPALQAAETGASSNASDKNLIETRCVLNHHS
QYC65180.1    .....GDPIADMIDQTVNNQVNR.SLTALQVLPTAANTEASSHRLGTGVVPALQAAETGASSNASDKNLIETRCVLNHHS
QWY15693.1    .....GDPIADMIDQTVNNQVNR.SLTALQVLPTAANTEASSHRLGTGVVPALQAAETGASSNASDKNLIETRCVLNHHS
QYC65179.1    .....GDPIADMIDQTVNNQVNR.SLTALQVLPTAANTEASSHRLGTGVVPALQAAETGASSNASDKNLIETRCVLNHHS
BCB92156.1    .....GDPIADMIDQTVNNQVNR.SLTALQVLPTAANTEASSHRLGTGVVPALQAAETGASSNASDKNLIETRCVLNHHS
QYC65178.1    .....GDPIADMIDQTVNNQVNR.SLTALQVLPTAANTEASSHRLGTGVVPALQAAETGASSNASDKNLIETRCVLNHHS
pdb|5C8C|A    .....GDPIADMIDQTVNNQVNR.SLTALQVLPTAANTEASSHRLGTGVVPALQAAETGASSNASDKNLIETRCVLNHHS
QWF08864.1    .....GDPIADMIDQTVNNQVNR.SLTALQVLPTAANTEASSHRLGTGVVPALQAAETGASSNASDKNLIETRCVLNHHS
QWF08747.1    .....GDPIADMIDQTVNNQVNR.SLTALQVLPTAANTEASSHRLGTGVVPALQAAETGASSNASDKNLIETRCVLNHHS
QWF08743.1    .....GDPIADMIDQTVNNQVNR.SLTALQVLPTAANTEASSHRLGTGVVPALQAAETGASSNASDKNLIETRCVLNHHS
QWF08741.1    .....GDPIADMIDQTVNNQVNR.SLTALQVLPTAANTEASSHRLGTGVVPALQAAETGASSNASDKNLIETRCVLNHHS
QWF08737.1    .....GDPIADMIDQTVNNQVNR.SLTALQVLPTAANTEASSHRLGTGVVPALQAAETGASSNASDKNLIETRCVLNHHS
QWF08734.1    .....GDPIADMIDQTVNNQVNR.SLTALQVLPTAANTEASSHRLGTGVVPALQAAETGASSNASDKNLIETRCVLNHHS
BBH72119.1    .....GDPIADMIDQTVNNQVNR.SLTALQVLPTAANTEASSHRLGTGVVPALQAAETGASSNASDKNLIETRCVLNHHS
QYC65177.1    .....GDPIADMIDQTVNNQVNR.SLTALQVLPTAANTEASSHRLGTGVVPALQAAETGASSNASDKNLIETRCVLNHHS
QWF08732.1    .....GDPIADMIDQTVNNQVNR.SLTALQVLPTAANTEASSHRLGTGVVPALQAAETGASSNASDKNLIETRCVLNHHS
QWF08730.1    .....GDPIADMIDQTVNNQVNR.SLTALQVLPTAANTEASSHRLGTGVVPALQAAETGASSNASDKNLIETRCVLNHHS
QYC65175.1    .....GDPIADMIDQTVNNQVNR.SLTALQVLPTAANTEASSHRLGTGVVPALQAAETGASSNASDKNLIETRCVLNHHS
QWF08727.1    .....GDPIADMIDQTVNNQVNR.SLTALQVLPTAANTEASSHRLGTGVVPALQAAETGASSNASDKNLIETRCVLNHHS
QYC65173.1    .....GDPIADMIDQTVNNQVNR.SLTALQVLPTAANTEASSHRLGTGVVPALQAAETGASSNASDKNLIETRCVLNHHS
BBH72105.1    .....GDPIADMIDQTVNNQVNR.SLTALQVLPTAANTEASSHRLGTGVVPALQAAETGASSNASDKNLIETRCVLNHHS
QYC65171.1    .....GDPIADMIDQTVNNQVNR.SLTALQVLPTAANTEASSHRLGTGVVPALQAAETGASSNASDKNLIETRCVLNHHS
pdb|5ABJ|A    .....GDPIADMIDQTVNNQVNR.SLTALQVLPTAANTEASSHRLGTGVVPALQAAETGASSNASDKNLIETRCVLNHHS
QWY15716.1    .....GDPIADMIDQTVNNQVNR.SLTALQVLPTAANTEASSHRLGTGVVPALQAAETGASSNASDKNLIETRCVLNHHS
QWY15800.1    .....GDPIADMIDQTVNNQVNR.SLTALQVLPTAANTEASSHRLGTGVVPALQAAETGASSNASDKNLIETRCVLNHHS
QYC65018.1    .....GDPIADMIDQTVNNQVNR.SLTALQVLPTAANTEASSHRLGTGVVPALQAAETGASSNASDKNLIETRCVLNHHS
QWY15712.1    .....GDPIADMIDQTVNNQVNR.SLTALQVLPTAANTEASSHRLGTGVVPALQAAETGASSNASDKNLIETRCVLNHHS
QWY15711.1    .....GDPIADMIDQTVNNQVNR.SLTALQVLPTAANTEASSHRLGTGVVPALQAAETGASSNASDKNLIETRCVLNHHS
QWY15707.1    .....GDPIADMIDQTVNNQVNR.SLTALQVLPTAANTEASSHRLGTGVVPALQAAETGASSNASDKNLIETRCVLNHHS
QWY15688.1    .....GDPIADMIDQTVNNQVNR.SLTALQVLPTAANTEASSHRLGTGVVPALQAAETGASSNASDKNLIETRCVLNHHS
QWY15685.1    .....GDPIADMIDQTVNNQVNR.SLTALQVLPTAANTEASSHRLGTGVVPALQAAETGASSNASDKNLIETRCVLNHHS
QWY15684.1    .....GDPIADMIDQTVNNQVNR.SLTALQVLPTAANTEASSHRLGTGVVPALQAAETGASSNASDKNLIETRCVLNHHS
QYC65170.1    .....GDPIADMIDQTVNNQVNR.SLTALQVLPTAANTEASSHRLGTGVVPALQAAETGASSNASDKNLIETRCVLNHHS
QYC65169.1    .....GDPIADMIDQTVNNQVNR.SLTALQVLPTAANTEASSHRLGTGVVPALQAAETGASSNASDKNLIETRCVLNHHS
QYC65162.1    .....GDPIADMIDQTVNNQVNR.SLTALQVLPTAANTEASSHRLGTGVVPALQAAETGASSNASDKNLIETRCVLNHHS
QYC65099.1    .....GDPIADMIDQTVNNQVNR.SLTALQVLPTAANTEASSHRLGTGVVPALQAAETGASSNASDKNLIETRCVLNHHS
QYC65048.1    .....GDPIADMIDQTVNNQVNR.SLTALQVLPTAANTEASSHRLGTGVVPALQAAETGASSNASDKNLIETRCVLNHHS
QYC65040.1    .....GDPIADMIDQTVNNQVNR.SLTALQVLPTAANTEASSHRLGTGVVPALQAAETGASSNASDKNLIETRCVLNHHS
QYC65035.1    .....GDPIADMIDQTVNNQVNR.SLTALQVLPTAANTEASSHRLGTGVVPALQAAETGASSNASDKNLIETRCVLNHHS
QYC65032.1    .....GDPIADMIDQTVNNQVNR.SLTALQVLPTAANTEASSHRLGTGVVPALQAAETGASSNASDKNLIETRCVLNHHS
QYC65030.1    .....GDPIADMIDQTVNNQVNR.SLTALQVLPTAANTEASSHRLGTGVVPALQAAETGASSNASDKNLIETRCVLNHHS
QYC65027.1    .....GDPIADMIDQTVNNQVNR.SLTALQVLPTAANTEASSHRLGTGVVPALQAAETGASSNASDKNLIETRCVLNHHS
QYC65026.1    .....GDPIADMIDQTVNNQVNR.SLTALQVLPTAANTEASSHRLGTGVVPALQAAETGASSNASDKNLIETRCVLNHHS
QYC65024.1    .....GDPIADMIDQTVNNQVNR.SLTALQVLPTAANTEASSHRLGTGVVPALQAAETGASSNASDKNLIETRCVLNHHS
QYC65022.1    .....GDPIADMIDQTVNNQVNR.SLTALQVLPTAANTEASSHRLGTGVVPALQAAETGASSNASDKNLIETRCVLNHHS
QYC65021.1    .....GDPIADMIDQTVNNQVNR.SLTALQVLPTAANTEASSHRLGTGVVPALQAAETGASSNASDKNLIETRCVLNHHS
QYC65020.1    .....GDPIADMIDQTVNNQVNR.SLTALQVLPTAANTEASSHRLGTGVVPALQAAETGASSNASDKNLIETRCVLNHHS
QYC65019.1    .....GDPIADMIDQTVNNQVNR.SLTALQVLPTAANTEASSHRLGTGVVPALQAAETGASSNASDKNLIETRCVLNHHS
QWF08742.1    .....GDPIADMIDQTVNNQVNR.SLTALQVLPTAANTEASSHRLGTGVVPALQAAETGASSNASDKNLIETRCVLNHHS
QWF08728.1    .....GEPIADMIDQTVNNQVNR.SLTALQVLPTAANTEASSHRLGTGVVPALQAAETGASSNASDKNLIETRCVLNHHS
QWF08726.1    .....GDPIADMIDQTVNNQVNR.SLTALQVLPTAANTEASSHRLGTGVVPALQAAETGASSNASDKNLIETRCVLNHHS
QYC65054.1    .....GDPIADMIDQTVNNQVNR.SLTALQVLPTAANTEASSHRLGTGVVPALQAAETGASSNASDKNLIETRCVLNHHS
QWF08746.1    .....GDPIADMIDQTVNNQVNR.SLTALQVLPTAANTEASSHRLGTGVVPALQAAETGASSNASDKNLIETRCVLNHHS
QYC65033.1    .....GDPIADMIDQTVNNQVNR.SLTALQVLPTAANTEASSHRLGTGVVPALQAAETGASSNASDKSLIETRCVLNHHS
QWY15952.1    .....GDPIADMIDQTVNNQVNR.SLTALQVLPTAANTEASSHRLGTGVVPALQAAETGASSNASDRNLIETRCVLNHHS
QYC65042.1    .....GDPIADMIDQTVNNQVNR.SLTALQILPTAANTEASSHRLGTGVVPALQAAETGASSNASDKNLIETRCVLNHHS
QYC65017.1    .....GDPIADMIDQTVNNQVNR.SLTALQILPTAANTEASSHRLGTGVVPALQAAETGASSNASDKNLIETRCVLNHHS
QOP74918.1    .....GDPIADMIDQTVNNQVNR.SLTALQVLPTAANTEASSHRLGTGVVPALQAAETGASSNASDKNLIETRCVLNHHS
QWF08731.1    .....GDTIADMIDQTVNNQVNR.SLTALQVLPTAANTEASSHRLGTGVVPALQAAETGASSNASDKNLIETRCVLNHHS
BBH72123.1    .....GDPIADMIHQTVNNQVNR.SLTALQVLPTAANTEASSHRLGTGVVPALQAAETGASSNASDKNLIETRCVLNHHS
BBH72114.1    .....GDPIGDMIHQTVNNQVNR.SLTAMQVLPTAANTEASSHRLGTGVVPALQAAETGASSNASDKNLIETRCVLNHHS
BBH72092.1    .....GDPIADMIHQTVNNQVNR.SLTALQVLPTAANTEASSHRLGTGVVPALQAAETGASSNASDKNLIETRCVLNHHS
BBH72095.1    .....GDPIADMIDQTVNNQVNR.SLTALQVLPTAANTEASSHRLGTGVVPALQAAETGASSNASDKNLIETRCVLNHHS
BBH72186.1    .....GDPIADMIDQTVNNQVNR.SLTALQVLPTAANTEASSHRLGTGVVPALQAAETGASSNASDKNLIETRCVLNHHS
QWY15975.1    .....GDPIADMIDQTVNNQVNR.SLTTLQVLPTAANTEASSHRLGTGVVPALQAAETGASSNASDKNLIETRCVLNHHS
QWY15974.1    .....GDPIADMIDQTVNNQVNR.SLTTLQVLPTAANTEASSHRLGTGVVPALQAAETGASSNASDKNLIETRCVLNHHS
QWY15968.1    .....GDPIADMIDQTVNNQVNR.SLTTLQVLPTAANTEASSHRLGTGVVPALQAAETGASSNASDKNLIETRCVLNHHS
QWY15960.1    .....GDPIADMIDQTVNNQVNR.SLTTLQVLPTAANTEASSHRLGTGVVPALQAAETGASSNASDKNLIETRCVLNHHS
QWY15959.1    .....GDPIADMIDQTVNNQVNR.SLTTLQVLPTAANTEASSHRLGTGVVPALQAAETGASSNASDKNLIETRCVLNHHS
QWY15953.1    .....GDPIADMIDQTVNNQVNR.SLTTLQVLPTAANTEASSHRLGTGVVPALQAAETGASSNASDKNLIETRCVLNHHS
QWY15951.1    .....GDPIADMIDQTVNNQVNR.SLTTLQVLPTAANTEASSHRLGTGVVPALQAAETGASSNASDKNLIETRCVLNHHS
QWY15950.1    .....GDPIADMIDQTVNNQVNR.SLTTLQVLPTAANTEASSHRLGTGVVPALQAAETGASSNASDKNLIETRCVLNHHS
QWY15949.1    .....GDPIADMIDQTVNNQVNR.SLTTLQVLPTAANTEASSHRLGTGVVPALQAAETGASSNASDKNLIETRCVLNHHS
QWY15944.1    .....GDPIADMIDQTVNNQVNR.SLTTLQVLPTAANTEASSHRLGTGVVPALQAAETGASSNASDKNLIETRCVLNHHS
QWY15943.1    .....GDPIADMIDQTVNNQVNR.SLTTLQVLPTAANTEASSHRLGTGVVPALQAAETGASSNASDKNLIETRCVLNHHS
QWY15942.1    .....GDPIADMIDQTVNNQVNR.SLTTLQVLPTAANTEASSHRLGTGVVPALQAAETGASSNASDKNLIETRCVLNHHS
QWY15939.1    .....GDPIADMIDQTVNNQVNR.SLTTLQVLPTAANTEASSHRLGTGVVPALQAAETGASSNASDKNLIETRCVLNHHS
QWY15938.1    .....GDPIADMIDQTVNNQVNR.SLTTLQVLPTAANTEASSHRLGTGVVPALQAAETGASSNASDKNLIETRCVLNHHS
QWY15937.1    .....GDPIADMIDQTVNNQVNR.SLTTLQVLPTAANTEASSHRLGTGVVPALQAAETGASSNASDKNLIETRCVLNHHS
QWY15936.1    .....GDPIADMIDQTVNNQVNR.SLTTLQVLPTAANTEASSHRLGTGVVPALQAAETGASSNASDKNLIETRCVLNHHS
QWY15934.1    .....GDPIADMIDQTVNNQVNR.SLTTLQVLPTAANTEASSHRLGTGVVPALQAAETGASSNASDKNLIETRCVLNHHS
QWY15896.1    .....GDPIADMIDQTVNNQVNR.SLTTLQVLPTAANTEASSHRLGTGVVPALQAAETGASSNASDKNLIETRCVLNHHS
BCU41098.1    .....GDPIADMIDQTVNNQVNR.SLTTLQVLPTAANTEASSHRLGTGVVPALQAAETGASSNASDKNLIETRCVLNHHS
BCR43090.1    .....GDPIADMIDQTVNNQVNR.SLTTLQVLPTAANTEASSHRLGTGVVPALQAAETGASSNASDKNLIETRCVLNHHS
QYC65129.1    .....GDPIADMIDQTVNNQVNR.SLTTLQVLPTAANTEASSHRLGTGVVPALQAAETGASSNASDKNLIETRCVLNHHS
QWF08961.1    .....GDPIADMIDQTVNNQVNR.SLTTLQVLPTAANTEASSHRLGTGVVPALQAAETGASSNASDKNLIETRCVLNHHS
QIL87527.1    .....GDPIADMIDQTVNNQVNR.SLTTLQVLPTAANTEASSHRLGTGVVPALQAAETGASSNASDKNLIETRCVLNHHS
QWY15955.1    .....GDPIADMIDQAVNNQVNR.SLTTLQVLPTAANTEASSHRLGTGVVPALQAAETGASSNASDKNLIETRCVLNHHS
QWY15954.1    .....GDPIADMIDQAVNNQVNR.SLTTLQVLPTAANTEASSHRLGTGVVPALQAAETGASSNASDKNLIETRCVLNHHS
QWY15945.1    .....GDPIADMIDQTVNDQVNR.SLTTFQVLPTAANTEASSHRLGTGVVPALQAAETGASSNASDKNLIETRCVLNHHS
QWY15935.1    .....GDPIADMIDQTVNDQVNR.SLTTLQVLPTAANTEASSHRLGTGVVPALQAAETGASSNASDKNLIETRCVLNHHS
BCB92163.1    .....GDPIADMIDQTVNNQVNR.SLTALQVLPTAANTEASSHRLGTGVVPALQAAETGASSNASDKNLIETRCVLNHHS
BCB92158.1    .....GDPIADMIDQTVNNQVNR.SLTALQVLPTAANTEASSHRLGTGVVPALQAAETGASSNASDKNLIETRCVLNHHS
BCB92154.1    .....GDPIADMIDQTVNNQVNR.SLTALQVLPTAANTEASSHRLGTGVVPALQAAETGASSNASDKNLIETRCVLNHHS
BCB92152.1    .....GDPIADMIDQTVNNQVNR.SLTALQVLPTAANTEASSHRLGTGVVPALQAAETGASSNASDKNLIETRCVLNHHS
BCB92162.1    .....GDPIADMIDQTVNNQVNR.SLTALQVLPTAANTEASSHRLGTGVVPALQAAETGASSNASDKNLIETRCVLNHHS
BCB92161.1    .....GDPIADMIDQTVNNQVNR.SLTALQVLPTAANTEASSHRLGTGVVPALQAAETGASSNASDKNLIETRCVLNHHS
BCB92160.1    .....GDPIADMIDQTVNNQVNR.SLTALQVLPTAANTEASSHRLGTGVVPALQAAETGASSNASDKNLIETRCVLNHHS
BBH72129.1    .....GDPIADMIDQTVNNQVNR.SLTALQVLPTAANTEASSHRLGTGVVPALQAAETGASSNASDKNLIETRCVLNHHS
QIL87533.1    .....GDPIADMIDQTVNNQVNR.SLTALQVLPTAANTEASSHRLGTGVVPALQAAETGASSNASDKNLIETRCVLNHHS
BBH72150.1    .....LDPIADMIDQTVNNQVNR.SLTALQVLPTAANTEASSHRLGTGVVPALQAAETGASSNASDKNLIETRCVLNHHS
BBH72137.1    .....LDSFADMIDQTVNNQVNR.SLTALQVLPTAANTEASSHRLGTGVVPALQAAETGASSNASDKNLIETRCVLNHHS
BBH72145.1    ......DPIADMIDQTVNNQVNR.SLTALQVLPTAANTEASSHRLGTGVVPALQAAETGASSNASDKNLIETRCVLNHHS
BCB92157.1    .....GDPIADMIDQTVNNQVNR.SLTALQVLPTAANTEASSHRLGTGVVPALQAAETGASSNASDKNLIETRCVLNHHS
QYC65124.1    .....GDPIADMIDQTVNNQVNR.SLTALQVLPTAANTEASSHRLGTGVVPALQAAETGASSNASDKNLIETRCVLNHHS
QWY15920.1    .....GDPIADMIDQTVNNQVNR.SLTALQVLPTAANTEASSHRLGTGVVPALQAAETGASSNASDKNLIETRCVLNHHS
QWY15973.1    .....GDPIADMIDQTVNNQVNR.SLTALQVLPTAANTEASSHRLGTGVVPALQAAETGASSNASDKNLIETRCVLNHHS
BCU41116.1    .....GDPIADMIDQTVNNQVNR.SLTALQVLPTAANTEASSHRLGTGVVPALQAAETGASSNASDKNLIETRCVLNHHS
QWF08960.1    .....GDPIADMIDQTVNNQVNR.SLTALQVLPTAANTEASSHRLGTGVVPALQAAETGASSNASDKNLIETRCVLNHHS
QWF08958.1    .....GDPIADMIDQTVNNQVNR.SLTALQVLPTAANTEASSHRLGTGVVPALQAAETGASSNASDKNLIETRCVLNHHS
QWF08919.1    .....GDPIADMIDQTVNNQVNR.SLTALQVLPTAANTEASSHRLGTGVVPALQAAETGASSNASDKNLIETRCVLNHHS
QWY15825.1    .....GDPIADMIDQTVNNQVNR.SLTALQVLPTAANTEASSHRLGTGVVPALQAAETGASSNASDKNLIETRCVLNHHS
QWF08953.1    .....GDPIADMIDQTVNNQVNR.SLTALQVLPTAANTEASSHRLGTGVVPALQAAETGASSNASDKNLIETRCVLNHHS
QWF08948.1    .....GDPIADMIDQTVNNQVNR.SLTALQVLPTAANTEASSHRLGTGVVPALQAAETGASSNASDKNLIETRCVLNHHS
QWY15884.1    .....GDPIADMIDQTVNNQVNR.SLTALQVLPTAANTEASSHRLGTGVVPALQAAETGASSNASDKNLIETRCVLNHHS
QWF08946.1    .....GDPIADMIDQTVNNQVNR.SLTALQVLPTAANTEASSHRLGTGVVPALQAAETGASSNASDKNLIETRCVLNHHS
QWF08941.1    .....GDPIADMIDQTVNNQVNR.SLTALQVLPTAANTEASSHRLGTGVVPALQAAETGASSNASDKNLIETRCVLNHHS
QWF08914.1    .....GDPIADMIDQTVNNQVNR.SLTALQVLPTAANTEASSHRLGTGVVPALQAAETGASSNASDKNLIETRCVLNHHS
BCU41097.1    .....GDPIADMIDQTVNNQVNR.SLTALQVLPTAANTEASSHRLGTGVVPALQAAETGASSNASDKNLIETRCVLNHHS
QWY15918.1    .....GDPIADMIDQTVNNQVNR.SLTALQVLPTAANTEASSHRLGTGVVPALQAAETGASSNASDKNLIETRCVLNHHS
BCR43086.1    .....GDPIADMIDQTVNNQVNR.SLTALQVLPTAANTEASSHRLGTGVVPALQAAETGASSNASDKNLIETRCVLNHHS
QWF08935.1    .....GDPIADMIDQTVNNQVNR.SLTALQVLPTAANTEASSHRLGTGVVPALQAAETGASSNASDKNLIETRCVLNHHS
QWY15912.1    .....GDPIADMIDQTVNNQVNR.SLTALQVLPTAANTEASSHRLGTGVVPALQAAETGASSNASDKNLIETRCVLNHHS
QWY15911.1    .....GDPIADMIDQTVNNQVNR.SLTALQVLPTAANTEASSHRLGTGVVPALQAAETGASSNASDKNLIETRCVLNHHS
QWY15887.1    .....GDPIADMIDQTVNNQVNR.SLTALQVLPTAANTEASSHRLGTGVVPALQAAETGASSNASDKNLIETRCVLNHHS
```

```
QWY15882.1    .....GDPIADMIDQTVNNQVNR.SLTALQVLPTAANTEASSHRLGTGVVPALQAAETGASSNASDKNLIETRCVLNHHS
BCG67018.1    .....GDPIADMIDQTVNNQVNR.SLTALQVLPTAANTEASSHRLGTGVVPALQAAETGASSNASDKNLIETRCVLNHHS
QWY15881.1    .....GDPIADMIDQTVNNQVNR.SLTALQVLPTAANTEASSHRLGTGVVPALQAAETGASSNASDKNLIETRCVLNHHS
QWY15875.1    .....GDPIADMIDQTVNNQVNR.SLTALQVLPTAANTEASSHRLGTGVVPALQAAETGASSNASDKNLIETRCVLNHHS
BCG67016.1    .....GDPIADMIDQTVNNQVNR.SLTALQVLPTAANTEASSHRLGTGVVPALQAAETGASSNASDKNLIETRCVLNHHS
QWY15874.1    .....GDPIADMIDQTVNNQVNR.SLTALQVLPTAANTEASSHRLGTGVVPALQAAETGASSNASDKNLIETRCVLNHHS
QWY15853.1    .....GDPIADMIDQTVNNQVNR.SLTALQVLPTAANTEASSHRLGTGVVPALQAAETGASSNASDKNLIETRCVLNHHS
BCG67014.1    .....GDPIADMIDQTVNNQVNR.SLTALQVLPTAANTEASSHRLGTGVVPALQAAETGASSNASDKNLIETRCVLNHHS
BCG67009.1    .....GDPIADMIDQTVNNQVNR.SLTALQVLPTAANTEASSHRLGTGVVPALQAAETGASSNASDKNLIETRCVLNHHS
QWF08933.1    .....GDPIADMIDQTVNNQVNR.SLTALQVLPTAANTEASSHRLGTGVVPALQAAETGASSNASDKNLIETRCVLNHHS
BCG66992.1    .....GDPIADMIDQTVNNQVNR.SLTALQVLPTAANTEASSHRLGTGVVPALQAAETGASSNASDKNLIETRCVLNHHS
BCG66991.1    .....GDPIADMIDQTVNNQVNR.SLTALQVLPTAANTEASSHRLGTGVVPALQAAETGASSNASDKNLIETRCVLNHHS
QWF08898.1    .....GDPIADMIDQTVNNQVNR.SLTALQVLPTAANTEASSHRLGTGVVPALQAAETGASSNASDKNLIETRCVLNHHS
QWF08930.1    .....GDPIADMIDQTVNNQVNR.SLTALQVLPTAANTEASSHRLGTGVVPALQAAETGASSNASDKNLIETRCVLNHHS
QWF08925.1    .....GDPIADMIDQTVNNQVNR.SLTALQVLPTAANTEASSHRLGTGVVPALQAAETGASSNASDKNLIETRCVLNHHS
QWF08913.1    .....GDPIADMIDQTVNNQVNR.SLTALQVLPTAANTEASSHRLGTGVVPALQAAETGASSNASDKNLIETRCVLNHHS
QWF08911.1    .....GDPIADMIDQTVNNQVNR.SLTALQVLPTAANTEASSHRLGTGVVPALQAAETGASSNASDKNLIETRCVLNHHS
BBH72117.1    .....GDPIADMIDQTVNNQVNR.SLTALQVLPTAANTEASSHRLGTGVVPALQAAETGASSNASDKNLIETRCVLNHHS
BBH72116.1    .....GDPIADMIDQTVNNQVNR.SLTALQVLPTAANTEASSHRLGTGVVPALQAAETGASSNASDKNLIETRCVLNHHS
BBH72113.1    .....GDPIADMIDQTVNNQVNR.SLTALQVLPTAANTEASSHRLGTGVVPALQAAETGASSNASDKNLIETRCVLNHHS
BBH72112.1    .....GDPIADMIDQTVNNQVNR.SLTALQVLPTAANTEASSHRLGTGVVPALQAAETGASSNASDKNLIETRCVLNHHS
BBH72108.1    .....GDPIADMIDQTVNNQVNR.SLTALQVLPTAANTEASSHRLGTGVVPALQAAETGASSNASDKNLIETRCVLNHHS
BBH72106.1    .....GDPIADMIDQTVNNQVNR.SLTALQVLPTAANTEASSHRLGTGVVPALQAAETGASSNASDKNLIETRCVLNHHS
BBH72104.1    .....GDPIADMIDQTVNNQVNR.SLTALQVLPTAANTEASSHRLGTGVVPALQAAETGASSNASDKNLIETRCVLNHHS
BBH72211.1    .....GDPIADMIDQTVNNQVNR.SLTALQVLPTAANTEASSHRLGTGVVPALQAAETGASSNASDKNLIETRCVLNHHS
BBH72210.1    .....GDPIADMIDQTVNNQVNR.SLTALQVLPTAANTEASSHRLGTGVVPALQAAETGASSNASDKNLIETRCVLNHHS
BBH72209.1    .....GDPIADMIDQTVNNQVNR.SLTALQVLPTAANTEASSHRLGTGVVPALQAAETGASSNASDKNLIETRCVLNHHS
BBH72208.1    .....GDPIADMIDQTVNNQVNR.SLTALQVLPTAANTEASSHRLGTGVVPALQAAETGASSNASDKNLIETRCVLNHHS
BBH72207.1    .....GDPIADMIDQTVNNQVNR.SLTALQVLPTAANTEASSHRLGTGVVPALQAAETGASSNASDKNLIETRCVLNHHS
BBH72205.1    .....GDPIADMIDQTVNNQVNR.SLTALQVLPTAANTEASSHRLGTGVVPALQAAETGASSNASDKNLIETRCVLNHHS
BBH72201.1    .....GDPIADMIDQTVNNQVNR.SLTALQVLPTAANTEASSHRLGTGVVPALQAAETGASSNASDKNLIETRCVLNHHS
BBH72193.1    .....GDPIADMIDQTVNNQVNR.SLTALQVLPTAANTEASSHRLGTGVVPALQAAETGASSNASDKNLIETRCVLNHHS
BBH72192.1    .....GDPIADMIDQTVNNQVNR.SLTALQVLPTAANTEASSHRLGTGVVPALQAAETGASSNASDKNLIETRCVLNHHS
BBH72190.1    .....GDPIADMIDQTVNNQVNR.SLTALQVLPTAANTEASSHRLGTGVVPALQAAETGASSNASDKNLIETRCVLNHHS
BBH72189.1    .....GDPIADMIDQTVNNQVNR.SLTALQVLPTAANTEASSHRLGTGVVPALQAAETGASSNASDKNLIETRCVLNHHS
BBH72188.1    .....GDPIADMIDQTVNNQVNR.SLTALQVLPTAANTEASSHRLGTGVVPALQAAETGASSNASDKNLIETRCVLNHHS
BBH72187.1    .....GDPIADMIDQTVNNQVNR.SLTALQVLPTAANTEASSHRLGTGVVPALQAAETGASSNASDKNLIETRCVLNHHS
BBH72185.1    .....GDPIADMIDQTVNNQVNR.SLTALQVLPTAANTEASSHRLGTGVVPALQAAETGASSNASDKNLIETRCVLNHHS
BBH72182.1    .....GDPIADMIDQTVNNQVNR.SLTALQVLPTAANTEASSHRLGTGVVPALQAAETGASSNASDKNLIETRCVLNHHS
BBH72181.1    .....GDPIADMIDQTVNNQVNR.SLTALQVLPTAANTEASSHRLGTGVVPALQAAETGASSNASDKNLIETRCVLNHHS
BBH72179.1    .....GDPIADMIDQTVNNQVNR.SLTALQVLPTAANTEASSHRLGTGVVPALQAAETGASSNASDKNLIETRCVLNHHS
BBH72178.1    .....GDPIADMIDQTVNNQVNR.SLTALQVLPTAANTEASSHRLGTGVVPALQAAETGASSNASDKNLIETRCVLNHHS
BBH72177.1    .....GDPIADMIDQTVNNQVNR.SLTALQVLPTAANTEASSHRLGTGVVPALQAAETGASSNASDKNLIETRCVLNHHS
BBH72175.1    .....GDPIADMIDQTVNNQVNR.SLTALQVLPTAANTEASSHRLGTGVVPALQAAETGASSNASDKNLIETRCVLNHHS
BBH72174.1    .....GDPIADMIDQTVNNQVNR.SLTALQVLPTAANTEASSHRLGTGVVPALQAAETGASSNASDKNLIETRCVLNHHS
BBH72172.1    .....GDPIADMIDQTVNNQVNR.SLTALQVLPTAANTEASSHRLGTGVVPALQAAETGASSNASDKNLIETRCVLNHHS
BBH72170.1    .....GDPIADMIDQTVNNQVNR.SLTALQVLPTAANTEASSHRLGTGVVPALQAAETGASSNASDKNLIETRCVLNHHS
BBH72169.1    .....GDPIADMIDQTVNNQVNR.SLTALQVLPTAANTEASSHRLGTGVVPALQAAETGASSNASDKNLIETRCVLNHHS
BBH72168.1    .....GDPIADMIDQTVNNQVNR.SLTALQVLPTAANTEASSHRLGTGVVPALQAAETGASSNASDKNLIETRCVLNHHS
BBH72158.1    .....GDPIADMIDQTVNNQVNR.SLTALQVLPTAANTEASSHRLGTGVVPALQAAETGASSNASDKNLIETRCVLNHHS
BBH72155.1    .....GDPIADMIDQTVNNQVNR.SLTALQVLPTAANTEASSHRLGTGVVPALQAAETGASSNASDKNLIETRCVLNHHS
BBH72154.1    .....GDPIADMIDQTVNNQVNR.SLTALQVLPTAANTEASSHRLGTGVVPALQAAETGASSNASDKNLIETRCVLNHHS
BBH72151.1    .....GDPIADMIDQTVNNQVNR.SLTALQVLPTAANTEASSHRLGTGVVPALQAAETGASSNASDKNLIETRCVLNHHS
BBH72148.1    .....GDPIADMIDQTVNNQVNR.SLTALQVLPTAANTEASSHRLGTGVVPALQAAETGASSNASDKNLIETRCVLNHHS
BBH72144.1    .....GDPIADMIDQTVNNQVNR.SLTALQVLPTAANTEASSHRLGTGVVPALQAAETGASSNASDKNLIETRCVLNHHS
BBH72142.1    .....GDPIADMIDQTVNNQVNR.SLTALQVLPTAANTEASSHRLGTGVVPALQAAETGASSNASDKNLIETRCVLNHHS
BBH72141.1    .....GDPIADMIDQTVNNQVNR.SLTALQVLPTAANTEASSHRLGTGVVPALQAAETGASSNASDKNLIETRCVLNHHS
BBH72140.1    .....GDPIADMIDQTVNNQVNR.SLTALQVLPTAANTEASSHRLGTGVVPALQAAETGASSNASDKNLIETRCVLNHHS
BBH72132.1    .....GDPIADMIDQTVNNQVNR.SLTALQVLPTAANTEASSHRLGTGVVPALQAAETGASSNASDKNLIETRCVLNHHS
BBH72131.1    .....GDPIADMIDQTVNNQVNR.SLTALQVLPTAANTEASSHRLGTGVVPALQAAETGASSNASDKNLIETRCVLNHHS
BBH72130.1    .....GDPIADMIDQTVNNQVNR.SLTALQVLPTAANTEASSHRLGTGVVPALQAAETGASSNASDKNLIETRCVLNHHS
BBH72128.1    .....GDPIADMIDQTVNNQVNR.SLTALQVLPTAANTEASSHRLGTGVVPALQAAETGASSNASDKNLIETRCVLNHHS
BDC16283.1    .....GDPIADMIDQTVNNQVNR.SLTALQVLPTAANTEASSHRLGTGVVPALQAAETGASSNASDKNLIETRCVLNHHS
BDC16271.1    .....GDPIADMIDQTVNNQVNR.SLTALQVLPTAANTEASSHRLGTGVVPALQAAETGASSNASDKNLIETRCVLNHHS
BDC16270.1    .....GDPIADMIDQTVNNQVNR.SLTALQVLPTAANTEASSHRLGTGVVPALQAAETGASSNASDKNLIETRCVLNHHS
BDC16258.1    .....GDPIADMIDQTVNNQVNR.SLTALQVLPTAANTEASSHRLGTGVVPALQAAETGASSNASDKNLIETRCVLNHHS
QIL87526.1    .....GDPIADMIDQTVNNQVNR.SLTALQVLPTAANTEASSHRLGTGVVPALQAAETGASSNASDKNLIETRCVLNHHS
BBH72180.1    .....GDPIADMIDQTVNNQVNR.SLTALQVLPTAANTEASSHRLGTGVVPALQAAETGASSNASDKNLIETRCVLNHHS
BBH72109.1    .....GDPIADMIDQTVNNQVNR.SLTALQVLPTAANTEASSHRLGTGVVPALQAAETGASSNASDKNLIETRCVLNHHS
BBH72115.1    .....GDPIADMIDQTVNNQVNR.SLTALQVLPTAANTEASSHRLGTGVVPALQAAETGASSNASDKNLIETRCVLNHHS
BBH72152.1    .....GDPIADMIDQTVNNQVNR.SLTALQVLPTAANTEASSHRLGTGVVPALQAAETGASSNASDKNLIETRCVLNHHS
BBH72200.1    .....GDPIADMIDQTVNNQVNR.SLTALQVLPTAANTEASSHRLGTGVVPALQAAETGASSNASDKNLIETRCVLNHHS
QWY15844.1    .....GDPIADMIDQTVNNQVNR.SLTALQVLPTAANTEASSHRLGTGVVPALQAAETGASSNASDKNLIETRCVLNHHS
BBH72146.1    .....GDPIADMIDQTVNNQVNR.SLTALQVLPTAANTEASSHRLSTGVVPALQAAETGASSNASDKNLIETRCVLNHHS
BBH72173.1    .....GDPIADMIDQTVNNQVNR.SLTALQVLPTAANTEASSHRLGTGVVPALQAAETGASSNASDKSLIETRCVLNHHS
QWY15947.1    .....GDPIADMIDQTVNNQVNR.SLTALQVLPTAANTEASSHRLGTGVVPALQAAETGASSNASDKNLIETRCVLNHHS
BDC16281.1    .....GDPIADMIDQTVNNQVNR.SLTALQVLPTAANTEASSHRLGTGVVPALQAAETGASSNASDKNLIETRCVLNHHS
BBH72167.1    .....GDPIADMIDQTVNNQVNR.SLTALQVLPTAANTEASSHRLGTGVVPALQAAETGASSNASDKNLIETRCVLNHHS
BBH72160.1    .....GDPIADMIDQTVNNQVNR.SLTALQVLPTAANTEASSHRLGTGVVPALQAAETGASSNASDKNLIETRCVLNHHS
BCU41096.1    .....GDPIADMIDQTVNNQVNR.SLTALQVLPTAADTEASSHRLGTGVVPALQAAETGASSNASDKNLIETRCVLNHHS
BCR43102.1    .....GDPIADMIDQTVNNQVNR.SLTALQVLPTAADTEASSHRLGTGVVPALQAAETGASSNASDKNLIETRCVLNHHS
QWF08881.1    .....GDPIADMIDQTVNNQVNR.SLTALQVLPTAANTEASSHRLGTGVVPALQAAETGASSNASDRNLIETRCVLNHHS
QWF08855.1    .....GDPIADMIDQTVNNQVNR.SLTALQVLPTAANTEASSHRLGTGVVPALQAAETGASSNASDRNLIETRCVLNHHS
BBH72195.1    .....GDPIADIIDQTVNNQVNR.SLTALQVLPTAANTEASSHRLGTGVVPALQAAETGASSNASDRNLIETRCVLNHHS
BBH72118.1    .....GEPIGDMIHQTVNNQVNR.SLTALQVLPTAANTEASSHRLGTGVVPALQAAETGASSNASDKNLIETRCVLNHHS
BBH72126.1    .....GDPIGDMIDQTVNNQVNR.SLTALQVLPTAANTEASSHRLGTGVVPALQAAETGASSNASDKNLIETRCVLNHHS
BBH72127.1    .....GDPIGDMIHHTVNNQVNR.SLTALQVLPTAANTEASSHRLGTGVVPALQAAETGASSNASDKNLIETRCVLNHHS
BBH72206.1    .....GDPIADMIDQTVNNQVNR.SLTALQVLPTAANTEASSHRLGTGVVPALQAAETGASSNASDKNLIETRCVLNHHS
BBH72196.1    .....GERIADSLDQTVNNQVNR.SLTALQVLPTAANTEASSHRLGTGVVPALQAAETGASSNASDKNLIETRCVLNHHS
BBH72171.1    .....GEPIADMIDQTVNNQVNR.SLTALQVLPTAANTEASSHRLGTGVVPALQAAETGASSNASDKNLIETRCVLNHHS
BBH72191.1    .....GDPIADMIDQTVNNQVNR.SLTALQVLPTAANTEASSHRLGTGVVPALQAAETGASSNASDKNLIETRCVLNHHS
BBH72133.1    .....GDPIADMIDQTVNNQVNR.SLTALQVLPTAANTEASSHRLGTGVVPALQAAETGASSNASDKNLIETRCVLNHHS
BBH72139.1    .....GDPIADMIDQTVNNQVNR.SLTALQVLPTAANTEASSHRLGTGVVPALQAAETGASSNASDKNLIETRCVLNHHS
BBH72166.1    .....GDPIADMIDQTVNNQVNR.SLTALQVLPTAANTEAGSHRLGTGVVPALQAAETGASSNASDKNLIETRCVLNHHS
BBH72161.1    .....GDPIADMIDQTVNNQVNR.SLTALQVLPTAANTEAGSHRLGTGVVPALQAAETGASSNASDKNLIETRCVLNHHS
BBH72165.1    .....GDPIADMIDQTGNNQVNR.CLTALQVLPTAANTEASSHRLGTGVVPALQAAETGASSNASDKNLIETRCVLNHHS
BBH72164.1    .....GDPIADMIDQTVNNQVNR.SLTALQVLPTAANTEASSHRLGTGVVPALQAAETGASSNASDKNLIETRCVLNHHS
BBH72159.1    .....GDPIADMIDQTVSNQVNR.SLTALQVLPTAANTEASSHRLGTGVVPALQAAETGASSNASDKNLIETRCVLNHHS
BBH72157.1    .....GDPIADMIDQTVNNQVNR.SLTALQVLPTAANTEASSHRLGTGVVPALQAAETGASSNASDKNLIETRCVLNHHS
BBH72162.1    .....GDPIADMIDQTVNNQVNR.SLTALQVLPTAANTEASSHRLGTGVVPALQAAETGASSNASDKNLIETRCVLNHHS
BBH72153.1    .....GDPIADMIDQTVNNQVNR.SLTALQVLPTAANTEASSHRLGTGVVPALQAAETGASSNASDKNLIETRCVLNHHS
BBH72156.1    .....GDPIADMIDQTVNNQVNR.SLTALQVLPTAANTEASSHRLGTGVVPALQAAETGASSNASDKNLIETRCVLNHHS
BBH72120.1    .....GDPIADMIDQTVNNQVNR.SLTALQVLPTAANTEASSHRLGTGVVPALQAAETGASSNASDKNLIETRCVLNHHS
BBH72198.1    .....GDPIADMIDQTVNNQVNR.SLTALQVLPTAANTEASSHRLGTGVVPALQAAETGASSNASDKNLIETRCVLNHHS
BBH72197.1    .....GDPIADMIDQTVSNQVNR.SLTALQVLPTAANTEASSHRLGTGVVPALQAAETGASSNASDKNLIETRCVLNHHS
QWY15941.1    .....GDPIADMIDQTVNNQVNR.SLTALQVLPTAANTEASSHRLGTGVVPALQAAETGASSNASDKNLIETRCVLNHHS
BBH72121.1    .....GDPIADMIDQTVNNQVNR.SLTALQVLPTAANTEASSHRLGTGVVPALQAAETGASSNASDKNLIETRCVLNHHS
BBH72135.1    .....GDPIADMIDQTVNNQVNR.SLTALQVLPTAANTEASSHRLGTGVVPALQAAETGASSNASDKNLIETRCVLNHHS
QWY15967.1    .....GDPIADMIDQTVNNQVNH.SLTALQVLPTAANTEASSHRLGTGVVPALQAAETGASSNASDKNLIETRCVLNHHS
QWY15964.1    .....GDPIADMIDQTVNNQVNH.SLTALQVLPTAANTEASSHRLGTGVVPALQAAETGASSNASDKNLIETRCVLNHHS
QWY15969.1    .....GDPIADMIDQTVNNQVNH.SLTALQVLPTAANTEASSHRLGTGVVPALQAAETGASSNASDKNLIETRCVLNHHS
QWY15913.1    .....GDPIADMIDQTVNNQVNR.SLTALQVLPTAANTEASSHRLGTGVVPALQAAETGASSNASDKNLIETRCVLNHHS
QWY15908.1    .....GDPIADMIDQTVNNQVNR.SLTALQVLPTAANTEASSHRLGTGVVPALQAAETGASSNASDKNLIETRCVLNHHS
QWY15907.1    .....GDPIADMIDQTVNNQVNR.SLTALQVLPTAANTEASSHRLGTGVVPALQAAETGASSNASDKNLIETRCVLNHHS
QWY15890.1    .....GDPIADMIDQTVNNQVNR.SLTALQVLPTAANTEASSHRLGTGVVPALQAAETGASSNASDKNLIETRCVLNHHS
QWY15889.1    .....GDPIADMIDQTVNNQVNR.SLTALQVLPTAANTEASSHRLGTGVVPALQAAETGASSNASDKNLIETRCVLNHHS
QWY15888.1    .....GDPIADMIDQTVNNQVNR.SLTALQVLPTAANTEASSHRLGTGVVPALQAAETGASSNASDKNLIETRCVLNHHS
QWY15886.1    .....GDPIADMIDQTVNNQVNR.SLTALQVLPTAANTEASSHRLGTGVVPALQAAETGASSNASDKNLIETRCVLNHHS
QWY15885.1    .....GDPIADMIDQTVNNQVNR.SLTALQVLPTAANTEASSHRLGTGVVPALQAAETGASSNASDKNLIETRCVLNHHS
QWY15883.1    .....GDPIADMIDQTVNNQVNR.SLTALQVLPTAANTEASSHRLGTGVVPALQAAETGASSNASDKNLIETRCVLNHHS
QWY15878.1    .....GDPIADMIDQTVNNQVNR.SLTALQVLPTAANTEASSHRLGTGVVPALQAAETGASSNASDKNLIETRCVLNHHS
QWY15876.1    .....GDPIADMIDQTVNNQVNR.SLTALQVLPTAANTEASSHRLGTGVVPALQAAETGASSNASDKNLIETRCVLNHHS
BBH72176.1    .....GDPIADMIDQTVNNQVNR.SLTALQVLPTAANTEASSHRLGTGVVPALQAAETGASSNASDKNLIETRCVLNHHS
QIL87519.1    .....GDPIADMIDQTVNNQVNR.SLTALQVLPTAANTEASSHRLGTGVVPALQAAETGASSNASDKNLIETRCVLNHHS
QWY15906.1    .....GDPIADMIDQTVNNQVNR.SLTALQVLPTAANTEASSHRLGTGVVPALQAAETGASSNASDKNLIETRCVLNHHS
QWY15917.1    .....GDPIADMIDQTVNNQVNR.SLTTLQVLPTAANTEASSHRLGTGVVPALQAAETGASSNASDKNLIETRCVLNHHS
QWY15846.1    .....GDPIADMIDQTVNNQVNR.SLTALQVLPTAANTEASSHRLGTGVVPALQAAETGASSNASDKNLIETRCVLNHHS
QWY15901.1    .....GDPIADMIDQTVNNQVNR.SLTALQVLPTAANTEASSHRLGTGVVPALQAAETGASSNASDKNLIETRCVLNHHS
QWY15857.1    .....GDPIADMIDQTVNNQVNR.SLTALQVLPTAANTEASSHRLGTGVVPALQAAETGASSNASDKNLIETRCVLNHHS
QWY15904.1    .....GDPIADMIDQTVNNQVNR.SLTALQVLPTAANTEASSHRLGTGVVPALQAAETGASSNASDKNLIETRCVLNHHS
BBH72204.1    .....GDPIADMIDQTVNNQVNR.SLTALQVLPTAANTEASSHRLGTGVVPALQAAETGASSNASDKNLIETRCVLNHHS
BBH72203.1    .....GDPIADMIDQTVNNQVNR.SLTALQVLPTAANTEASSHRLGTGVVPALQAAETGASSNASDKNLIETRCVLNHHS
BBH72202.1    .....GDPIADMIDQTVNNQVNR.SLTALQVLPTAANTEASSHRLGTGVVPALQAAETGASSNASDKNLIETRCVLNHHS
BDC16266.1    .....GDPIADMIDQTVNNQVNR.SLTALQVLPTAANTEASSHRLGTGVVPALQAAETGASSNASDKNLIETRCVLNHHS
BCR43091.1    .....GDPIADMIDQTVNNQVSR.SLTALQVLPTAANTEASSHRLGTGVVPALQAAETGASSNASDKNLIETRCVLNHHS
QWY15902.1    .....GDPIADMMDQTGNNQVNR.SLTAFQVLPTAANTEASSHRLGTGVVPALQAAETGASSNASDKNLIETRCVLNHHS
BBH72226.1    .....GDAIADAIQNTVTSTIQRVATSAVGQDATAANTASSTHSLNTGLVPALQAAETGASSTATDGNLIETRCVVNSNG
BBH72222.1    .....GDAIADAIQNTVTSTIQRVATSAVGQDATAANTASSTHSLNTGLVPALQAAETGASSTATDGNLIETRCVVNSNG
BBH72012.1    .........NDPITNAVESAVSALADTTISR.VTAANTAASTHSLGTGRVPALQAAETGASSNASDENLIETRCVMNRNG
BBH71965.1    .........NDPITNAVESAVSALADTTISR.VTAANTAASTHSLGTGRVPALQAAETGASSNASDENLIETRCVMNRNG
BBH71934.1    .........NDPITNAVESRVSALADTTISR.VTAANTAASTHSLGTGRVPALQAAETGASSNASDENLIETRCVMNRNG
BBH72009.1    .........NDPITNAVESAVSALADTTISR.VTAANTAASTHSLGTGRVPALQAAETGASSNASDENLIETRCVMNRNG
QWY16175.1    .....GDPIADIIEGAVTQTANR.AISGSIQPVTAANTQPSSHRLGTGQVPALQAAETGATSNATDESMIETRCVVNRHG
QWY16167.1    .....GDPIADIIEGAVTQTTNR.AISGSIQPVTAANTQPSSHRLGTGQVPALQAAETGATSNATDESMIETRCVVNRHG
```

```
QWY16174.1    .....GDPIADIIEGAVTQTTNR.AISGPIQPVTAANTQPSSHRLGTGQVPALQAAETGATSNATDESMIETRCVVNRHG
QWY16171.1    .....GDPIADIIEGAVTQTTNR.AISGPIQPVTAANTQPSSHRLGTGQVPALQAAETGATSNATDESMIETRCVVNRHG
QWY16173.1    .....GDPIADIIEGAVTQTTNR.AISGPIQPVTAANTQPSSHRLGTGQVPALQAAETGATSNATDESMIETRCVVNRHG
QWY16172.1    .....GDPIADIIEGAVIQTTNR.AISGPIQPVTAANTQPSSHRLGTGQVPALQAAETGATSNATDESMIETRCVVNRHG
QWY16170.1    .....GDPIADIIEGAVIQTTNR.AISGPIQPVTAANTQPSSHRLGTGQVPALQAAETGATSNATDESMIETRCVVNRHG
QWY16169.1    .....GDPIADIIEGAVTQTTNR.AISGSIQPVTAANTQPSSHRLGTGQVPALQAAETGATSNATDESMIETRCVVNRHG
QWY16168.1    .....GDPIADIIEGAVTQTTNR.AISGSIQPVTAANTQPSSHRLGTGQVPALQAAETGATSNATDESMIETRCVVNRHG
QXF31588.1    .....GDTVSDMIENSINRITN..AISTTQTHQTAADTRVSNHRLGTGEVPALQAAETGATSNATDESMIETRCVVNKHG
QXF31587.1    .....GDTVSDMIENSINRITN..AISTTQTHQTAADTRVSNHRLGTGEVPALQAAETGATSNATDESMIETRCVVNKHG
pdb|6IIO|A    .....GDPVEDIIHDALGNTARR.AISGATNVESAADTTPSSHRLETGRVPALQAAETGATSNATDENMIETRCVINRNG
pdb|6IIJ|A    .....GDPVEDIIHDALGNTARR.AISGATNVESAADTTPSSHRLETGRVPALQAAETGATSNATDENMIETRCVINRNG
pdb|6AKS|A    .....GDPVEDIIHDALGSTARR.AISSATNVESAANTTPSSHRLETGRVPALQAAETGATSNATDENMIETRCVVNRNG
BCD33944.1    ....RPGISQEAVLQAPEDAINGALTGIINNIPTAANTTESHHNISTSATPALQAAETGATSNTSDEGMIETRHVVNTNT
pdb|7ECY|A    ............IESIIKTATDT.......VKSEIN..AELG......VVPSLNAVETGATSNTEPEEAIQTRTVINQHG
pdb|7EC5|A    ............IESIIKTATDT.......VKSEIN..AELG......VVPSLNAVETGATSNTEPEEAIQTRTVINQHG
pdb|7EBZ|A    ............IESIIKTATDT.......VKSEIN..AELG......VVPSLNAVETGATSNTEPEEAIQTRTVINQHG
pdb|7EBR|A    ............IESIIKTATDT.......VKSEIN..AELG......VVPSLNAVETGATSNTEPEEAIQTRTVINQHG
pdb|6MZI|A    ............IESIIKTATDT.......VKSEIN..AELG......VVPSLNAVETGATSNTEPEEAIQTRTVINQHG
pdb|6CSH|A    ............IESIIKTATDT.......VKSEIN..AELG......VVPSLNAVETGATSNTEPEEAIQTRTVINQHG
pdb|6CSG|A    ............IESIIKTATDT.......VKSEIN..AELG......VVPSLNAVETGATSNTEPEEAIQTRTVINQHG
pdb|6CSA|A    ............IESIIKTATDT.......VKSEIN..AELG......VVPSLNAVETGATSNTEPEEAIQTRTVINQHG
pdb|6CS6|A    ............IESIIKTATDT.......VKSEIN..AELG......VVPSLNAVETGATSNTEPEEAIQTRTVINQHG
pdb|6CS5|A    ............IESIIKTATDT.......VKSEIN..AELG......VVPSLNAVETGATSNTEPEEAIQTRTVINQHG
pdb|6CS4|A    ............IESIIKTATDT.......VKSEIN..AELG......VVPSLNAVETGATSNTEPEEAIQTRTVINQHG
pdb|6CS3|A    ............IESIIKTATDT.......VKSEIN..AELG......VVPSLNAVETGATSNTEPEEAIQTRTVINQHG
pdb|6CRU|A    ............IESIIKTATDT.......VKSEIN..AELG......VVPSLNAVETGATSNTEPEEAIQTRTVINQHG
pdb|6CRS|A    ............IESIIKTATDT.......VKSEIN..AELG......VVPSLNAVETGATSNTEPEEAIQTRTVINQHG
pdb|6CRR|A    ............IESIIKTATDT.......VKSEIN..AELG......VVPSLNAVETGATSNTEPEEAIQTRTVINQHG
pdb|6CRP|A    ............IESIIKTATDT.......VKSEIN..AELG......VVPSLNAVETGATSNTEPEEAIQTRTVINQHG
QNO39042.1    ............IESIIKTATDT.......VKSEIN..AELG......VVPSLNAVETGATSNTEPEEAIQTRTVINQHG
QNO39041.1    ............IESIIKTATDT.......VKSEIN..AELG......VVPSLNAVETGATSNTEPEEAIQTRTVINQHG
QNO39040.1    ............IESIIKTATDT.......VKSEIN..AELG......VVPSLNAVETGATSNTEPEEAIQTRTVINQHG
QNO39038.1    ............IESIIKTATDT.......VKSEIN..AELG......VVPSLNAVETGATSNTEPEEAIQTRTVINQHG
QNO39039.1    ............IENIIKTATDT.......VKSEIN..AELG......VVPSLNAVETGATSNTEPEEAIQTRTVINQHG
CAA1539354.1  ............IESIIKTATDT.......VKSEIN..AELG......VVPSLNAVETGATSNTEPEEAIQTRTVINQHG
CAA1527138.1  ............IESIIKTATDT.......VKSEIN..AELG......VVPSLNAVETGATSNTEPEEAIQTRTVINQHG
CAA1539353.1  ............IESIIKTATDT.......VKSEIN..AELG......VVPSLNAVETGATSNTEPEEAIQTRTVINQHG
CAA1527136.1  ............IESIIKTATDT.......VKSEIN..AELG......VVPSLNAVETGATSNTEPEEAIQTRTVINQHG
QNO39037.1    ............IESIIKTATDT.......VKSEIN..AELG......VVPSLNAVETGATSNTEPEEAIQTRTVINQHG
QKX95883.1    LDHLHGAEAAYQIESIIKTATDT.......VKSEIN..AELG......VVPSLNAVETGATSNTEPEEAIQTRTVINQHG
pdb|5BNP|A    ...........VESIIKTATDT.......VKSEIN..AELG......VVPSLNAVETGATSNTEPEEAIQTRTVINQHG
pdb|5BNO|A    ...........VESIIKTATDT.......VKSEIN..AELG......VVPSLNAVETGATSNTEPEEAIQTRTVINQHG
pdb|5BNN|A    ...........VESIIKTATDT.......VKSEIN..AELG......VVPSLNAVETGATSNTEPEEAIQTRTVINQHG
pdb|4WM8|A    ...........VESIIKTATDT.......VKSEIN..AELG......VVPSLNAVETGATSNTEPEEAIQTRTVINQHG
pdb|4WM7|A    ...........VESIIKTATDT.......VKSEIN..AELG......VVPSLNAVETGATSNTEPEEAIQTRTVINQHG
QIC50353.1    .......GIDDVIDTVVANALKV.......SMPQVQNTQSSGP.VNSKEVPALTAVETGATSQIEPSDVIETRHVINQRL
QIC50352.1    .......GIDDVIDTVVANALKV.......SMPQVQNTQSSGP.VNSKEVPALTAVETGATSQIEPSDVIETRHVINQRL
QIC50356.1    .......GIEDTIDKVVDNALAL.......SMPRVQDTQQSGP.VNSKEVPALTAVETGATSQVTPEDLIETRHVINNRL
```

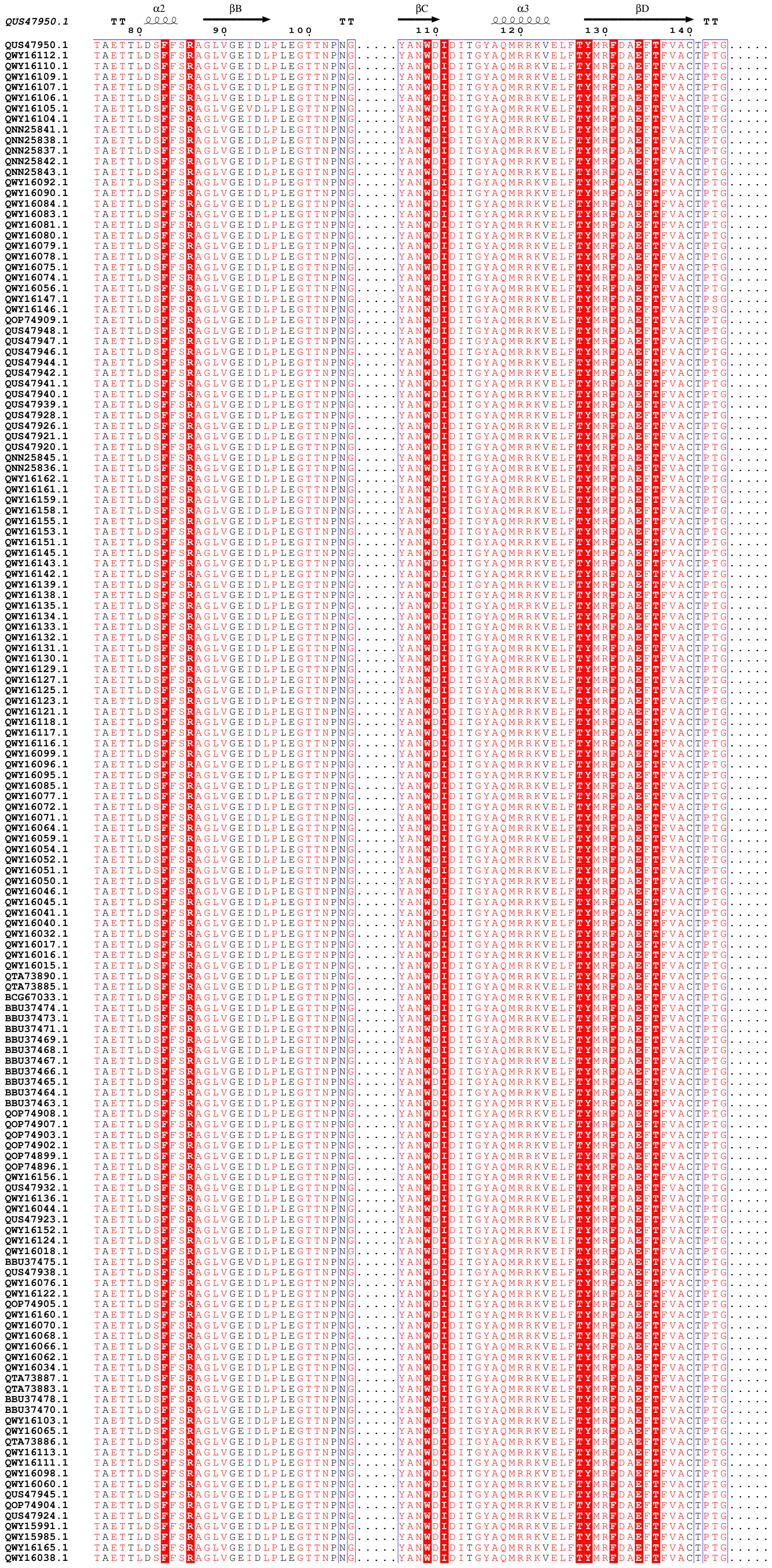

α2 βB βC α3 βD
QUS47950.1 TT TT TT
80 90 100 110 120 130 140
QUS47950.1 TAETTLDSFFSRAGLVGEIDLPLEGTTNPNG.....YANWDIDITGYAQMRRKVELFTYMRFDAEFTFVACTPTG.....
QWY16112.1 TAETTLDSFFSRAGLVGEIDLPLEGTTNPNG.....YANWDIDITGYAQMRRKVELFTYMRFDAEFTFVACTPTG.....
QWY16110.1 TAETTLDSFFSRAGLVGEIDLPLEGTTNPNG.....YANWDIDITGYAQMRRKVELFTYMRFDAEFTFVACTPTG.....
QWY16109.1 TAETTLDSFFSRAGLVGEIDLPLEGTTNPNG.....YANWDIDITGYAQMRRKVELFTYMRFDAEFTFVACTPTG.....
QWY16107.1 TAETTLDSFFSRAGLVGEIDLPLEGTTNPNG.....YANWDIDITGYAQMRRKVELFTYMRFDAEFTFVACTPTG.....
QWY16106.1 TAETTLDSFFSRAGLVGEIDLPLEGTTNPNG.....YANWDIDITGYAQMRRKVELFTYMRFDAEFTFVACTPTG.....
QWY16105.1 TAETTLDSFFSRAGLVGEVDLPLEGTTNPNG.....YANWDIDITGYAQMRRKVELFTYMRFDAEFTFVACTPTG.....
QWY16104.1 TAETTLDSFFSRAGLVGEIDLPLEGTTNPNG.....YANWDIDITGYAQMRRKVELFTYMRFDAEFTFVACTPTG.....
QNN25841.1 TAETTLDSFFSRAGLVGEIDLPLEGTTNPNG.....YANWDIDITGYAQMRRKVELFTYMRFDAEFTFVACTPTG.....
QNN25838.1 TAETTLDSFFSRAGLVGEIDLPLEGTTNPNG.....YANWDIDITGYAQMRRKVELFTYMRFDAEFTFVACTPTG.....
QNN25837.1 TAETTLDSFFSRAGLVGEIDLPLEGTTNPNG.....YANWDIDITGYAQMRRKVELFTYMRFDAEFTFVACTPTG.....
QNN25842.1 TAETTLDSFFSRAGLVGEIDLPLEGTTNPNG.....YANWDIDITGYAQMRRKVELFTYMRFDAEFTFVACTPTG.....
QNN25843.1 TAETTLDSFFSRAGLVGEIDLPLEGTTNPNG.....YANWDIDITGYAQMRRKVELFTYMRFDAEFTFVACTPTG.....
QWY16092.1 TAETTLDSFFSRAGLVGEIDLPLEGTTNPNG.....YANWDIDITGYAQMRRKVELFTYMRFDAEFTFVACTPTG.....
QWY16090.1 TAETTLDSFFSRAGLVGEIDLPLEGTTNPNG.....YANWDIDITGYAQMRRKVELFTYMRFDAEFTFVACTPTG.....
QWY16084.1 TAETTLDSFFSRAGLVGEIDLPLEGTTNPNG.....YANWDIDITGYAQMRRKVELFTYMRFDAEFTFVACTPTG.....
QWY16083.1 TAETTLDSFFSRAGLVGEIDLPLEGTTNPNG.....YANWDIDITGYAQMRRKVELFTYMRFDAEFTFVACTPTG.....
QWY16081.1 TAETTLDSFFSRAGLVGEIDLPLEGTTNPNG.....YANWDIDITGYAQMRRKVELFTYMRFDAEFTFVACTPTG.....
QWY16080.1 TAETTLDSFFSRAGLVGEIDLPLEGTTNPNG.....YANWDIDITGYAQMRRKVELFTYMRFDAEFTFVACTPTG.....
QWY16079.1 TAETTLDSFFSRAGLVGEIDLPLEGTTNPNG.....YANWDIDITGYAQMRRKVELFTYMRFDAEFTFVACTPTG.....
QWY16078.1 TAETTLDSFFSRAGLVGEIDLPLEGTTNPNG.....YANWDIDITGYAQMRRKVELFTYMRFDAEFTFVACTPTG.....
QWY16075.1 TAETTLDSFFSRAGLVGEIDLPLEGTTNPNG.....YANWDIDITGYAQMRRKVELFTYMRFDAEFTFVACTPTG.....
QWY16074.1 TAETTLDSFFSRAGLVGEIDLPLEGTTNPNG.....YANWDIDITGYAQMRRKVELFTYMRFDAEFTFVACTPTG.....
QWY16056.1 TAETTLDSFFSRAGLVGEIDLPLEGTTNPNG.....YANWDIDITGYAQMRRKVELFTYMRFDAEFTFVACTPTG.....
QWY16147.1 TAETTLDSFFSRAGLVGEIDLPLEGTTNPNG.....YANWDIDITGYAQMRRKVELFTYMRFDAEFTFVACTPSG.....
QWY16146.1 TAETTLDSFFSRAGLVGEIDLPLEGTTNPNG.....YANWDIDITGYAQMRRKVELFTYMRFDAEFTFVACTPSG.....
QOP74909.1 TAETTLDSFFSRAGLVGEIDLPLEGTTNPNG.....YANWDIDITGYAQMRRKVELFTYMRFDAEFTFVACTPTG.....
QUS47948.1 TAETTLDSFFSRAGLVGEIDLPLEGTTNPNG.....YANWDIDITGYAQMRRKVELFTYMRFDAEFTFVACTPTG.....
QUS47947.1 TAETTLDSFFSRAGLVGEIDLPLEGTTNPNG.....YANWDIDITGYAQMRRKVELFTYMRFDAEFTFVACTPTG.....
QUS47946.1 TAETTLDSFFSRAGLVGEIDLPLEGTTNPNG.....YANWDIDITGYAQMRRKVELFTYMRFDAEFTFVACTPTG.....
QUS47944.1 TAETTLDSFFSRAGLVGEIDLPLEGTTNPNG.....YANWDIDITGYAQMRRKVELFTYMRFDAEFTFVACTPTG.....
QUS47942.1 TAETTLDSFFSRAGLVGEIDLPLEGTTNPNG.....YANWDIDITGYAQMRRKVELFTYMRFDAEFTFVACTPTG.....
QUS47941.1 TAETTLDSFFSRAGLVGEIDLPLEGTTNPNG.....YANWDIDITGYAQMRRKVELFTYMRFDAEFTFVACTPTG.....
QUS47940.1 TAETTLDSFFSRAGLVGEIDLPLEGTTNPNG.....YANWDIDITGYAQMRRKVELFTYMRFDAEFTFVACTPTG.....
QUS47939.1 TAETTLDSFFSRAGLVGEIDLPLEGTTNPNG.....YANWDIDITGYAQMRRKVELFTYMRFDAEFTFVACTPTG.....
QUS47928.1 TAETTLDSFFSRAGLVGEIDLPLEGTTNPNG.....YANWDIDITGYAQMRRKVELFTYMRFDAEFTFVACTPTG.....
QUS47926.1 TAETTLDSFFSRAGLVGEIDLPLEGTTNPNG.....YANWDIDITGYAQMRRKVELFTYMRFDAEFTFVACTPTG.....
QUS47921.1 TAETTLDSFFSRAGLVGEIDLPLEGTTNPNG.....YANWDIDITGYAQMRRKVELFTYMRFDAEFTFVACTPTG.....
QUS47920.1 TAETTLDSFFSRAGLVGEIDLPLEGTTNPNG.....YANWDIDITGYAQMRRKVELFTYMRFDAEFTFVACTPTG.....
QNN25845.1 TAETTLDSFFSRAGLVGEIDLPLEGTTNPNG.....YANWDIDITGYAQMRRKVELFTYMRFDAEFTFVACTPTG.....
QNN25836.1 TAETTLDSFFSRAGLVGEIDLPLEGTTNPNG.....YANWDIDITGYAQMRRKVELFTYMRFDAEFTFVACTPTG.....
QWY16162.1 TAETTLDSFFSRAGLVGEIDLPLEGTTNPNG.....YANWDIDITGYAQMRRKVELFTYMRFDAEFTFVACTPTG.....
QWY16161.1 TAETTLDSFFSRAGLVGEIDLPLEGTTNPNG.....YANWDIDITGYAQMRRKVELFTYMRFDAEFTFVACTPTG.....
QWY16159.1 TAETTLDSFFSRAGLVGEIDLPLEGTTNPNG.....YANWDIDITGYAQMRRKVELFTYMRFDAEFTFVACTPTG.....
QWY16158.1 TAETTLDSFFSRAGLVGEIDLPLEGTTNPNG.....YANWDIDITGYAQMRRKVELFTYMRFDAEFTFVACTPTG.....
QWY16155.1 TAETTLDSFFSRAGLVGEIDLPLEGTTNPNG.....YANWDIDITGYAQMRRKVELFTYMRFDAEFTFVACTPTG.....
QWY16153.1 TAETTLDSFFSRAGLVGEIDLPLEGTTNPNG.....YANWDIDITGYAQMRRKVELFTYMRFDAEFTFVACTPTG.....
QWY16151.1 TAETTLDSFFSRAGLVGEIDLPLEGTTNPNG.....YANWDIDITGYAQMRRKVELFTYMRFDAEFTFVACTPTG.....
QWY16145.1 TAETTLDSFFSRAGLVGEIDLPLEGTTNPNG.....YANWDIDITGYAQMRRKVELFTYMRFDAEFTFVACTPTG.....
QWY16143.1 TAETTLDSFFSRAGLVGEIDLPLEGTTNPNG.....YANWDIDITGYAQMRRKVELFTYMRFDAEFTFVACTPTG.....
QWY16142.1 TAETTLDSFFSRAGLVGEIDLPLEGTTNPNG.....YANWDIDITGYAQMRRKVELFTYMRFDAEFTFVACTPTG.....
QWY16139.1 TAETTLDSFFSRAGLVGEIDLPLEGTTNPNG.....YANWDIDITGYAQMRRKVELFTYMRFDAEFTFVACTPTG.....
QWY16138.1 TAETTLDSFFSRAGLVGEIDLPLEGTTNPNG.....YANWDIDITGYAQMRRKVELFTYMRFDAEFTFVACTPTG.....
QWY16135.1 TAETTLDSFFSRAGLVGEIDLPLEGTTNPNG.....YANWDIDITGYAQMRRKVELFTYMRFDAEFTFVACTPTG.....
QWY16134.1 TAETTLDSFFSRAGLVGEIDLPLEGTTNPNG.....YANWDIDITGYAQMRRKVELFTYMRFDAEFTFVACTPTG.....
QWY16133.1 TAETTLDSFFSRAGLVGEIDLPLEGTTNPNG.....YANWDIDITGYAQMRRKVELFTYMRFDAEFTFVACTPTG.....
QWY16132.1 TAETTLDSFFSRAGLVGEIDLPLEGTTNPNG.....YANWDIDITGYAQMRRKVELFTYMRFDAEFTFVACTPTG.....
QWY16131.1 TAETTLDSFFSRAGLVGEIDLPLEGTTNPNG.....YANWDIDITGYAQMRRKVELFTYMRFDAEFTFVACTPTG.....
QWY16130.1 TAETTLDSFFSRAGLVGEIDLPLEGTTNPNG.....YANWDIDITGYAQMRRKVELFTYMRFDAEFTFVACTPTG.....
QWY16129.1 TAETTLDSFFSRAGLVGEIDLPLEGTTNPNG.....YANWDIDITGYAQMRRKVELFTYMRFDAEFTFVACTPTG.....
QWY16127.1 TAETTLDSFFSRAGLVGEIDLPLEGTTNPNG.....YANWDIDITGYAQMRRKVELFTYMRFDAEFTFVACTPTG.....
QWY16125.1 TAETTLDSFFSRAGLVGEIDLPLEGTTNPNG.....YANWDIDITGYAQMRRKVELFTYMRFDAEFTFVACTPTG.....
QWY16123.1 TAETTLDSFFSRAGLVGEIDLPLEGTTNPNG.....YANWDIDITGYAQMRRKVELFTYMRFDAEFTFVACTPTG.....
QWY16121.1 TAETTLDSFFSRAGLVGEIDLPLEGTTNPNG.....YANWDIDITGYAQMRRKVELFTYMRFDAEFTFVACTPTG.....
QWY16118.1 TAETTLDSFFSRAGLVGEIDLPLEGTTNPNG.....YANWDIDITGYAQMRRKVELFTYMRFDAEFTFVACTPTG.....
QWY16117.1 TAETTLDSFFSRAGLVGEIDLPLEGTTNPNG.....YANWDIDITGYAQMRRKVELFTYMRFDAEFTFVACTPTG.....
QWY16116.1 TAETTLDSFFSRAGLVGEIDLPLEGTTNPNG.....YANWDIDITGYAQMRRKVELFTYMRFDAEFTFVACTPTG.....
QWY16099.1 TAETTLDSFFSRAGLVGEIDLPLEGTTNPNG.....YANWDIDITGYAQMRRKVELFTYMRFDAEFTFVACTPTG.....
QWY16096.1 TAETTLDSFFSRAGLVGEIDLPLEGTTNPNG.....YANWDIDITGYAQMRRKVELFTYMRFDAEFTFVACTPTG.....
QWY16095.1 TAETTLDSFFSRAGLVGEIDLPLEGTTNPNG.....YANWDIDITGYAQMRRKVELFTYMRFDAEFTFVACTPTG.....
QWY16085.1 TAETTLDSFFSRAGLVGEIDLPLEGTTNPNG.....YANWDIDITGYAQMRRKVELFTYMRFDAEFTFVACTPTG.....
QWY16077.1 TAETTLDSFFSRAGLVGEIDLPLEGTTNPNG.....YANWDIDITGYAQMRRKVELFTYMRFDAEFTFVACTPTG.....
QWY16072.1 TAETTLDSFFSRAGLVGEIDLPLEGTTNPNG.....YANWDIDITGYAQMRRKVELFTYMRFDAEFTFVACTPTG.....
QWY16071.1 TAETTLDSFFSRAGLVGEIDLPLEGTTNPNG.....YANWDIDITGYAQMRRKVELFTYMRFDAEFTFVACTPTG.....
QWY16064.1 TAETTLDSFFSRAGLVGEIDLPLEGTTNPNG.....YANWDIDITGYAQMRRKVELFTYMRFDAEFTFVACTPTG.....
QWY16059.1 TAETTLDSFFSRAGLVGEIDLPLEGTTNPNG.....YANWDIDITGYAQMRRKVELFTYMRFDAEFTFVACTPTG.....
QWY16054.1 TAETTLDSFFSRAGLVGEIDLPLEGTTNPNG.....YANWDIDITGYAQMRRKVELFTYMRFDAEFTFVACTPTG.....
QWY16052.1 TAETTLDSFFSRAGLVGEIDLPLEGTTNPNG.....YANWDIDITGYAQMRRKVELFTYMRFDAEFTFVACTPTG.....
QWY16051.1 TAETTLDSFFSRAGLVGEIDLPLEGTTNPNG.....YANWDIDITGYAQMRRKVELFTYMRFDAEFTFVACTPTG.....
QWY16050.1 TAETTLDSFFSRAGLVGEIDLPLEGTTNPNG.....YANWDIDITGYAQMRRKVELFTYMRFDAEFTFVACTPTG.....
QWY16046.1 TAETTLDSFFSRAGLVGEIDLPLEGTTNPNG.....YANWDIDITGYAQMRRKVELFTYMRFDAEFTFVACTPTG.....
QWY16045.1 TAETTLDSFFSRAGLVGEIDLPLEGTTNPNG.....YANWDIDITGYAQMRRKVELFTYMRFDAEFTFVACTPTG.....
QWY16041.1 TAETTLDSFFSRAGLVGEIDLPLEGTTNPNG.....YANWDIDITGYAQMRRKVELFTYMRFDAEFTFVACTPTG.....
QWY16040.1 TAETTLDSFFSRAGLVGEIDLPLEGTTNPNG.....YANWDIDITGYAQMRRKVELFTYMRFDAEFTFVACTPTG.....
QWY16032.1 TAETTLDSFFSRAGLVGEIDLPLEGTTNPNG.....YANWDIDITGYAQMRRKVELFTYMRFDAEFTFVACTPTG.....
QWY16017.1 TAETTLDSFFSRAGLVGEIDLPLEGTTNPNG.....YANWDIDITGYAQMRRKVELFTYMRFDAEFTFVACTPTG.....
QWY16016.1 TAETTLDSFFSRAGLVGEIDLPLEGTTNPNG.....YANWDIDITGYAQMRRKVELFTYMRFDAEFTFVACTPTG.....
QWY16015.1 TAETTLDSFFSRAGLVGEIDLPLEGTTNPNG.....YANWDIDITGYAQMRRKVELFTYMRFDAEFTFVACTPTG.....
QTA73890.1 TAETTLDSFFSRAGLVGEIDLPLEGTTNPNG.....YANWDIDITGYAQMRRKVELFTYMRFDAEFTFVACTPTG.....
QTA73885.1 TAETTLDSFFSRAGLVGEIDLPLEGTTNPNG.....YANWDIDITGYAQMRRKVELFTYMRFDAEFTFVACTPTG.....
BCG67033.1 TAETTLDSFFSRAGLVGEIDLPLEGTTNPNG.....YANWDIDITGYAQMRRKVELFTYMRFDAEFTFVACTPTG.....
BBU37474.1 TAETTLDSFFSRAGLVGEIDLPLEGTTNPNG.....YANWDIDITGYAQMRRKVELFTYMRFDAEFTFVACTPTG.....
BBU37473.1 TAETTLDSFFSRAGLVGEIDLPLEGTTNPNG.....YANWDIDITGYAQMRRKVELFTYMRFDAEFTFVACTPTG.....
BBU37471.1 TAETTLDSFFSRAGLVGEIDLPLEGTTNPNG.....YANWDIDITGYAQMRRKVELFTYMRFDAEFTFVACTPTG.....
BBU37469.1 TAETTLDSFFSRAGLVGEIDLPLEGTTNPNG.....YANWDIDITGYAQMRRKVELFTYMRFDAEFTFVACTPTG.....
BBU37468.1 TAETTLDSFFSRAGLVGEIDLPLEGTTNPNG.....YANWDIDITGYAQMRRKVELFTYMRFDAEFTFVACTPTG.....
BBU37467.1 TAETTLDSFFSRAGLVGEIDLPLEGTTNPNG.....YANWDIDITGYAQMRRKVELFTYMRFDAEFTFVACTPTG.....
BBU37466.1 TAETTLDSFFSRAGLVGEIDLPLEGTTNPNG.....YANWDIDITGYAQMRRKVELFTYMRFDAEFTFVACTPTG.....
BBU37465.1 TAETTLDSFFSRAGLVGEIDLPLEGTTNPNG.....YANWDIDITGYAQMRRKVELFTYMRFDAEFTFVACTPTG.....
BBU37464.1 TAETTLDSFFSRAGLVGEIDLPLEGTTNPNG.....YANWDIDITGYAQMRRKVELFTYMRFDAEFTFVACTPTG.....
BBU37463.1 TAETTLDSFFSRAGLVGEIDLPLEGTTNPNG.....YANWDIDITGYAQMRRKVELFTYMRFDAEFTFVACTPTG.....
QOP74908.1 TAETTLDSFFSRAGLVGEIDLPLEGTTNPNG.....YANWDIDITGYAQMRRKVELFTYMRFDAEFTFVACTPTG.....
QOP74907.1 TAETTLDSFFSRAGLVGEIDLPLEGTTNPNG.....YANWDIDITGYAQMRRKVELFTYMRFDAEFTFVACTPTG.....
QOP74903.1 TAETTLDSFFSRAGLVGEIDLPLEGTTNPNG.....YANWDIDITGYAQMRRKVELFTYMRFDAEFTFVACTPTG.....
QOP74902.1 TAETTLDSFFSRAGLVGEIDLPLEGTTNPNG.....YANWDIDITGYAQMRRKVELFTYMRFDAEFTFVACTPTG.....
QOP74899.1 TAETTLDSFFSRAGLVGEIDLPLEGTTNPNG.....YANWDIDITGYAQMRRKVELFTYMRFDAEFTFVACTPTG.....
QOP74896.1 TAETTLDSFFSRAGLVGEIDLPLEGTTNPNG.....YANWDIDITGYAQMRRKVELFTYMRFDAEFTFVACTPTG.....
QWY16156.1 TAETTLDSFFSRAGLVGEIDLPLEGTTNPNG.....YANWDIDITGYAQMRRKVELFTYMRFDAEFTFVACTPTG.....
QUS47932.1 TAETTLDSFFSRAGLVGEIDLPLEGTTNPNG.....YANWDIDITGYAQMRRKVELFTYMRFDAEFTFVACTPTG.....
QWY16136.1 TAETTLDSFFSRAGLVGEIDLPLEGTTNPNG.....YANWDIDITGYAQMRRKVELFTYMRFDAEFTFVACTPTG.....
QWY16044.1 TAETTLDSFFSRAGLVGEIDLPLEGTTNPNG.....YANWDIDITGYAQMRRKVELFTYMRFDAEFTFVACTPTG.....
QUS47923.1 TAETTLDSFFSRAGLVGEIDLPLEGTTNPNG.....YANWDIDITGYAQMRRKVELFTYMRFDAEFTFVACTPTG.....
QWY16152.1 TAETTLDSFFSRAGLVGEIDLPLEGTTNPNG.....YANWDIDITGYAQMRRKVEIFTYMRFDAEFTFVACTPTG.....
QWY16124.1 TAETTLDSFFSRAGLVGEIDLPLEGTTNPNG.....YANWDIDITGYAQMRRKVEIFTYMRFDAEFTFVACTPTG.....
QWY16018.1 TAETTLDSFFSRAGLVGEIDLPLEGTTNPNG.....YANWDIDITGYAQMRRKVEIFTYMRFDAEFTFVACTPTG.....
BBU37475.1 TAETTLDSFFSRAGLVGEVDLPLEGTTNPNG.....YANWDIDITGYAQMRRKVELFTYMRFDAEFTFVACTPTG.....
QUS47938.1 TAETTLDSFFSRAGLVGEIDLPLEGTTNPNG.....YANWDIDITGYAQMRRKVELFTYMRFDAEFTFVACTPTG.....
QWY16076.1 TAETTLDSFFSRAGLVGEIDLPLEGTTNPNG.....YANWDIDITGYAQMRRKVELFTYMRFDAEFTFVACTPTG.....
QWY16122.1 TAETTLDSFFSRAGLVGEIDLPLEGTTNPNG.....YANWDIDITGYAQMRRKVELFTYMRFDAEFTFVACTPTG.....
QOP74905.1 TAETTLDSFFSRAGLVGEIDLPLEGTTNPNG.....YANWDIDITGYAQMRRKVELFTYMRFDAEFTFVACTPTG.....
QWY16160.1 TAETTLDSFFSRAGLVGEIDLPLEGTTNPNG.....YANWDIDITGYAQMRRKVELFTYMRFDAEFTFVACTPTG.....
QWY16070.1 TAETTLDSFFSRAGLVGEIDLPLEGTTNPNG.....YANWDIDITGYAQMRRKVELFTYMRFDAEFTFVACTPTG.....
QWY16068.1 TAETTLDSFFSRAGLVGEIDLPLEGTTNPNG.....YANWDIDITGYAQMRRKVELFTYMRFDAEFTFVACTPTG.....
QWY16066.1 TAETTLDSFFSRAGLVGEIDLPLEGTTNPNG.....YANWDIDITGYAQMRRKVELFTYMRFDAEFTFVACTPTG.....
QWY16062.1 TAETTLDSFFSRAGLVGEIDLPLEGTTNPNG.....YANWDIDITGYAQMRRKVELFTYMRFDAEFTFVACTPTG.....
QWY16034.1 TAETTLDSFFSRAGLVGEIDLPLEGTTNPNG.....YANWDIDITGYAQMRRKVELFTYMRFDAEFTFVACTPTG.....
QTA73887.1 TAETTLDSFFSRAGLVGEIDLPLEGTTNPNG.....YANWDIDITGYAQMRRKVELFTYMRFDAEFTFVACTPTG.....
QTA73883.1 TAETTLDSFFSRAGLVGEIDLPLEGTTNPNG.....YANWDIDITGYAQMRRKVELFTYMRFDAEFTFVACTPTG.....
BBU37478.1 TAETTLDSFFSRAGLVGEIDLPLEGTTNPNG.....YANWDIDITGYAQMRRKVELFTYMRFDAEFTFVACTPTG.....
BBU37470.1 TAETTLDSFFSRAGLVGEIDLPLEGTTNPNG.....YANWDIDITGYAQMRRKVELFTYMRFDAEFTFVACTPTG.....
QWY16103.1 TAETTLDSFFSRAGLVGEIDLPLEGTTNPNG.....YANWDIDITGYAQMRRKVELFTYMRFDAEFTFVACTPTG.....
QWY16065.1 TAETTLDSFFSRAGLVGEIDLPLEGTTNPNG.....YANWDIDITGYAQMRRKVELFTYMRFDAEFTFVACTPTG.....
QTA73886.1 TAETTLDSFFSRAGLVGEIDLPLEGTTNPNG.....YANWDIDITGYAQMRRKVELFTYMRFDAEFTFVACTPTG.....
QWY16113.1 TAETTLDSFFSRAGLVGEIDLPLEGTTNPNG.....YANWDIDITGYAQMRRKVELFTYMRFDAEFTFVACTPTG.....
QWY16111.1 TAETTLDSFFSRAGLVGEIDLPLEGTTNPNG.....YANWDIDITGYAQMRRKVELFTYMRFDAEFTFVACTPTG.....
QWY16098.1 TAETTLDSFFSRAGLVGEIDLPLEGTTNPNG.....YANWDIDITGYAQMRRKVELFTYMRFDAEFTFVACTPTG.....
QWY16060.1 TAETTLDSFFSRAGLVGEIDLPLEGTTNPNG.....YANWDIDITGYAQMRRKVELFTYMRFDAEFTFVACTPTG.....
QUS47945.1 TAETTLDSFFSRAGLVGEIDLPLEGTTNPNG.....YANWDIDITGYAQMRRKVELFTYMRFDAEFTFVACTPTG.....
QOP74904.1 TAETTLDSFFSRAGLVGEIDLPLEGTTNPNG.....YANWDIDITGYAQMRRKVELFTYMRFDAEFTFVACTPTG.....
QUS47924.1 TAETTLDSFFSRAGLVGEIDLPLEGTTNPNG.....YANWDIDITGYAQMRRKVELFTYMRFDAEFTFVACTPTG.....
QWY15991.1 TAETTLDSFFSRAGLVGEIDLPLEGTTNPNG.....YANWDIDITGYAQMRRKVELFTYMRFDAEFTFVACTPTG.....
QWY15985.1 TAETTLDSFFSRAGLVGEIDLPLEGTTNPNG.....YANWDIDITGYAQMRRKVELFTYMRFDAEFTFVACTPTG.....
QWY16165.1 TAETTLDSFFSRAGLVGEIDLPLEGTTNPNG.....YANWDIDITGYAQMRRKVELFTYMRFDAEFTFVACTPTG.....
QWY16038.1 TAETTLDSFFSRAGLVGEIDLPLEGTTNPNG.....YANWDIDITGYAQMRRKVELFTYMRFDAEFTFVACTPTG.....

```
QWY16036.1    TAETTLDSFFSRAGLVGEIDLPLEGTTNPNG.....YANWDIDITGYAQMRRKVELFTYMRFDAEFTFVACTPTG.....
QWY16120.1    TAETTLDSFFSRAGLVGEIDLPLEGTTNPNG.....YANWDIDITGYAQMRRKVELFTYMRFDAEFTFVACTPTG.....
QWY16119.1    TAETTLDSFFSRAGLVGEIDLPLEGTTNPNG.....YANWDIDITGYAQMRRKVELFTYMRFDAEFTFVACTPTG.....
QWY16102.1    TAETTLDSFFSRAGLVGEIDLPLEGTTNPNG.....YANWDIDITGYAQMRRKVELFTYMRFDAEFTFVACTPTG.....
QWY16100.1    TAETTLDSFFSRAGLVGEIDLPLEGTTNPNG.....YANWDIDITGYAQMRRKVELFTYMRFDAEFTFVACTPTG.....
QWY16097.1    TAETTLDSFFSRAGLVGEIDLPLEGTTNPNG.....YANWDIDITGYAQMRRKVELFTYMRFDAEFTFVACTPTG.....
QWY16101.1    TAETTLDSFFSRAGLVGEIDLPLEGTTNPNG.....YANWDIDITGYAQMRRKVELFTYMRFDAEFTFVACTPTG.....
BBU37472.1    TAETTLDSFFSRAGLVGEIDLPLEGTTNPNG.....YANWDIDITGYAQMRRKVELFTYMRFDAEFTFVACTPTG.....
QWY16108.1    TAETTLDSFFSRAGLVGEIDLPLEGTTNPNG.....YANWDIDITGYAQMRRKVELFTYMRFDAEFTFVACTPTG.....
QWY16042.1    TAETTLDSFFSRAGLVGEIDLPLEGTTNPNG.....YANWDIDITGYAQMRRKVELFTYMRFDAEFTFVACTPTG.....
QOP74910.1    TAETTLDSFFSRAGLVGEIDLPLEGTTNPNG.....YANWDIDITGYAQMRRKVELFTYMRFDAEFTFVACTPTG.....
QWY16128.1    TAETTLDSFFSRAGLVGEIDLPLEGTTNPNG.....YANWDIDITGYAQMRRKVELFTYMRFDAEFTFVACTPTG.....
QUS47905.1    TAETTLDSFFSRAGLVGEIDLPLEGTTNPNG.....YANWDIDITGYAQMRRKVELFTYMRFDAEFTFVACTPTG.....
QWY16154.1    TAETTLDSFFSRAGLVGEIDLPLEGTTNPNG.....YANWDIDITGYAQMRRKVELFTYMRFDAEFTFVACTPTG.....
QWY16126.1    TAETTLDSFFSRAGLVGEIDLPLEGTTNPNG.....YANWDIDITGYAQMRRKVELFTYMRFDAEFTFVACTPTG.....
QWY16049.1    TAETTLDSFFSRAGLVGEIDLPLEGTTNPNG.....YANWDIDITGYAQMRRKVELFTYMRFDAEFTFVACTPTG.....
QWY16043.1    TAETTLDSFFSRAGLVGEIDLPLEGTTNPNG.....YANWDIDITGYAQMRRKVELFTYMRFDAEFTFVACTPTG.....
QUS47911.1    TAETTLDSFFSRAGLVGEIDLPLKGTTNPNG.....YANWDIDITGYAQMRRKVELFTYMRFDAEFTFVACTPTG.....
BBU37477.1    TAETTLDSFFSRAGLVGEIDLPLKGTTNPNG.....YANWDIDITGYAQMRRKVELFTYMRFDAEFTFVACTPTG.....
QWY16137.1    TAETTLDSFFSRAGLVGEIDLPLEGTTNPNG.....YANWDIDITGYAQMRRKVELFTYMRFDAEFTFVACTPTG.....
QWY16061.1    TAETTLDSFFSRAGLVGEIDLPLEGTTNPNG.....YANWDIDITGYAQMRRKVELFTYMRFDAEFTFVACTPTG.....
QTA73882.1    TAETTLDSFFSRAGLVGEIDLPLEGTTNPNG.....YANWDIDITGYAQMRRKVELFTYMRFDAEFTFVACTPTG.....
QWY16031.1    TAETTLDSFFSRAGLVGEIDLPLEGTTNPNG.....YANWDIDITGYAQLRRKVELFTYMRFDAEFTFVACTPTG.....
BBU37476.1    TAETALDSFFSRAGLVGEIDLPLEGTTNPNG.....YANWDIDITGYAQMRRKVELFTYMRFDAEFTFVACTPTG.....
QUS47949.1    TAETTLDSFFSRAGLVGEIDLPLEGTTNPNG.....YANWDIDITGYAQMRRKVELFTYMRFDAEFTFVACTPTG.....
QNN25833.1    TAETTLDSFFSRAGLVGEIDLPLEGTTNPNG.....YANWDIDVTGYAQMRRKVELFTYMRFDAEFTFVACTPTG.....
QWY16114.1    TAETTLDSFFSRAGLVGEIDLPLEGTTNPNG.....YANWDIDITGYAQMRRKVELFTYMRFDAEFTFVACTPTG.....
QWY16063.1    TAETTLDSFFSRAGLVGEIDLPLEGTTNPNG.....YANWDIDITGYAQMRRKVELFTYMRFDAEFTFVACTPTG.....
QTA73884.1    TAETTLDSFFSRAGLVGEIDLPLEGTTNPNG.....YANWDIDITGYAQMRRKVELFTYMRFDAEFTFVACTPTG.....
QWY16002.1    TAETTLDSFFSRAGLVGEIDLPLEGTTNPNG.....YANWDIDITGYAQMRRKVELFTYMRFDAEFTFVACTPTG.....
QWY15984.1    TAETTLDSFFSRAGLVGEIDLPLEGTTNPNG.....YANWDIDITGYAQMRRKVELFTYMRFDAEFTFVACTPTG.....
QUS47934.1    TAETTLDSFFSRAGLVGEIDLPLEGTTNPNG.....YANWDIDITGYAQMRRKVELFTYMRFDAEFTFVACTPTG.....
QUS47933.1    TAETTLDSFFSRAGLVGEIDLPLEGTTNPNG.....YANWDIDITGYAQMRRKVELFTYMRFDAEFTFVACTPTG.....
QUS47929.1    TAETTLDSFFSRAGLVGEIDLPLEGTTNPNG.....YANWDIDITGYAQMRRKVELFTYMRFDAEFTFVACTPTG.....
QUS47927.1    TAETTLDSFFSRAGLVGEIDLPLEGTTNPNG.....YANWDIDITGYAQMRRKVELFTYMRFDAEFTFVACTPTG.....
QUS47919.1    TAETTLDSFFSRAGLVGEIDLPLEGTTNPNG.....YANWDIDITGYAQMRRKVELFTYMRFDAEFTFVACTPTG.....
QUS47917.1    TAETTLDSFFSRAGLVGEIDLPLEGTTNPNG.....YANWDIDITGYAQMRRKVELFTYMRFDAEFTFVACTPTG.....
QUS47915.1    TAETTLDSFFSRAGLVGEIDLPLEGTTNPNG.....YANWDIDITGYAQMRRKVELFTYMRFDAEFTFVACTPTG.....
QUS47913.1    TAETTLDSFFSRAGLVGEIDLPLEGTTNPNG.....YANWDIDITGYAQMRRKVELFTYMRFDAEFTFVACTPTG.....
QWY16166.1    TAETTLDSFFSRAGLVGEIDLPLEGTTNPNG.....YANWDIDITGYAQMRRKVELFTYMRFDAEFTFVACTPTG.....
QWY16164.1    TAETTLDSFFSRAGLVGEIDLPLEGTTNPNG.....YANWDIDITGYAQMRRKVELFTYMRFDAEFTFVACTPTG.....
QWY16163.1    TAETTLDSFFSRAGLVGEIDLPLEGTTNPNG.....YANWDIDITGYAQMRRKVELFTYMRFDAEFTFVACTPTG.....
QWY16157.1    TAETTLDSFFSRAGLVGEIDLPLEGTTNPNG.....YANWDIDITGYAQMRRKVELFTYMRFDAEFTFVACTPTG.....
QWY16150.1    TAETTLDSFFSRAGLVGEIDLPLEGTTNPNG.....YANWDIDITGYAQMRRKVELFTYMRFDAEFTFVACTPTG.....
QWY16115.1    TAETTLDSFFSRAGLVGEIDLPLEGTTNPNG.....YANWDIDITGYAQMRRKVELFTYMRFDAEFTFVACTPTG.....
QWY16087.1    TAETTLDSFFSRAGLVGEIDLPLEGTTNPNG.....YANWDIDITGYAQMRRKVELFTYMRFDAEFTFVACTPTG.....
QWY16069.1    TAETTLDSFFSRAGLVGEIDLPLEGTTNPNG.....YANWDIDITGYAQMRRKVELFTYMRFDAEFTFVACTPTG.....
QWY16058.1    TAETTLDSFFSRAGLVGEIDLPLEGTTNPNG.....YANWDIDITGYAQMRRKVELFTYMRFDAEFTFVACTPTG.....
QWY16048.1    TAETTLDSFFSRAGLVGEIDLPLEGTTNPNG.....YANWDIDITGYAQMRRKVELFTYMRFDAEFTFVACTPTG.....
QWY16047.1    TAETTLDSFFSRAGLVGEIDLPLEGTTNPNG.....YANWDIDITGYAQMRRKVELFTYMRFDAEFTFVACTPTG.....
QWY16030.1    TAETTLDSFFSRAGLVGEIDLPLEGTTNPNG.....YANWDIDITGYAQMRRKVELFTYMRFDAEFTFVACTPTG.....
QWY16027.1    TAETTLDSFFSRAGLVGEIDLPLEGTTNPNG.....YANWDIDITGYAQMRRKVELFTYMRFDAEFTFVACTPTG.....
QWY16021.1    TAETTLDSFFSRAGLVGEIDLPLEGTTNPNG.....YANWDIDITGYAQMRRKVELFTYMRFDAEFTFVACTPTG.....
QWY16020.1    TAETTLDSFFSRAGLVGEIDLPLEGTTNPNG.....YANWDIDITGYAQMRRKVELFTYMRFDAEFTFVACTPTG.....
QWY16009.1    TAETTLDSFFSRAGLVGEIDLPLEGTTNPNG.....YANWDIDITGYAQMRRKVELFTYMRFDAEFTFVACTPTG.....
QWY16008.1    TAETTLDSFFSRAGLVGEIDLPLEGTTNPNG.....YANWDIDITGYAQMRRKVELFTYMRFDAEFTFVACTPTG.....
QWY16007.1    TAETTLDSFFSRAGLVGEIDLPLEGTTNPNG.....YANWDIDITGYAQMRRKVELFTYMRFDAEFTFVACTPTG.....
QWY16001.1    TAETTLDSFFSRAGLVGEIDLPLEGTTNPNG.....YANWDIDITGYAQMRRKVELFTYMRFDAEFTFVACTPTG.....
QWY16000.1    TAETTLDSFFSRAGLVGEIDLPLEGTTNPNG.....YANWDIDITGYAQMRRKVELFTYMRFDAEFTFVACTPTG.....
QWY15993.1    TAETTLDSFFSRAGLVGEIDLPLEGTTNPNG.....YANWDIDITGYAQMRRKVELFTYMRFDAEFTFVACTPTG.....
QWY15986.1    TAETTLDSFFSRAGLVGEIDLPLEGTTNPNG.....YANWDIDITGYAQMRRKVELFTYMRFDAEFTFVACTPTG.....
QWY15979.1    TAETTLDSFFSRAGLVGEIDLPLEGTTNPNG.....YANWDIDITGYAQMRRKVELFTYMRFDAEFTFVACTPTG.....
QTA73889.1    TAETTLDSFFSRAGLVGEIDLPLEGTTNPNG.....YANWDIDITGYAQMRRKVELFTYMRFDAEFTFVACTPTG.....
QTA73874.1    TAETTLDSFFSRAGLVGEIDLPLEGTTNPNG.....YANWDIDITGYAQMRRKVELFTYMRFDAEFTFVACTPTG.....
QTA73871.1    TAETTLDSFFSRAGLVGEIDLPLEGTTNPNG.....YANWDIDITGYAQMRRKVELFTYMRFDAEFTFVACTPTG.....
QOP74901.1    TAETTLDSFFSRAGLVGEIDLPLEGTTNPNG.....YANWDIDITGYAQMRRKVELFTYMRFDAEFTFVACTPTG.....
QOP74900.1    TAETTLDSFFSRAGLVGEIDLPLEGTTNPNG.....YANWDIDITGYAQMRRKVELFTYMRFDAEFTFVACTPTG.....
QOP74897.1    TAETTLDSFFSRAGLVGEIDLPLEGTTNPNG.....YANWDIDITGYAQMRRKVELFTYMRFDAEFTFVACTPTG.....
QOP74893.1    TAETTLDSFFSRAGLVGEIDLPLEGTTNPNG.....YANWDIDITGYAQMRRKVELFTYMRFDAEFTFVACTPTG.....
QOP74887.1    TAETTLDSFFSRAGLVGEIDLPLEGTTNPNG.....YANWDIDITGYAQMRRKVELFTYMRFDAEFTFVACTPTG.....
QOP74886.1    TAETTLDSFFSRAGLVGEIDLPLEGTTNPNG.....YANWDIDITGYAQMRRKVELFTYMRFDAEFTFVACTPTG.....
QOP74884.1    TAETTLDSFFSRAGLVGEIDLPLEGTTNPNG.....YANWDIDITGYAQMRRKVELFTYMRFDAEFTFVACTPTG.....
QOP74882.1    TAETTLDSFFSRAGLVGEIDLPLEGTTNPNG.....YANWDIDITGYAQMRRKVELFTYMRFDAEFTFVACTPTG.....
QOP74881.1    TAETTLDSFFSRAGLVGEIDLPLEGTTNPNG.....YANWDIDITGYAQMRRKVELFTYMRFDAEFTFVACTPTG.....
QOP74880.1    TAETTLDSFFSRAGLVGEIDLPLEGTTNPNG.....YANWDIDITGYAQMRRKVELFTYMRFDAEFTFVACTPTG.....
QOP74879.1    TAETTLDSFFSRAGLVGEIDLPLEGTTNPNG.....YANWDIDITGYAQMRRKVELFTYMRFDAEFTFVACTPTG.....
QOP74878.1    TAETTLDSFFSRAGLVGEIDLPLEGTTNPNG.....YANWDIDITGYAQMRRKVELFTYMRFDAEFTFVACTPTG.....
pdb|4YVS|M    TAETTLDSFFSRAGLVGEIDLPLEGTTNPNG.....YANWDIDITGYAQMRRKVELFTYMRFDAEFTFVACTPTG.....
pdb|4YVS|J    TAETTLDSFFSRAGLVGEIDLPLEGTTNPNG.....YANWDIDITGYAQMRRKVELFTYMRFDAEFTFVACTPTG.....
pdb|4YVS|G    TAETTLDSFFSRAGLVGEIDLPLEGTTNPNG.....YANWDIDITGYAQMRRKVELFTYMRFDAEFTFVACTPTG.....
pdb|4YVS|D    TAETTLDSFFSRAGLVGEIDLPLEGTTNPNG.....YANWDIDITGYAQMRRKVELFTYMRFDAEFTFVACTPTG.....
pdb|4YVS|A    TAETTLDSFFSRAGLVGEIDLPLEGTTNPNG.....YANWDIDITGYAQMRRKVELFTYMRFDAEFTFVACTPTG.....
pdb|4CEY|A    TAETTLDSFFSRAGLVGEIDLPLEGTTNPNG.....YANWDIDITGYAQMRRKVELFTYMRFDAEFTFVACTPTG.....
pdb|4CEW|A    TAETTLDSFFSRAGLVGEIDLPLEGTTNPNG.....YANWDIDITGYAQMRRKVELFTYMRFDAEFTFVACTPTG.....
pdb|4N53|A    TAETTLDSFFSRAGLVGEIDLPLEGTTNPNG.....YANWDIDITGYAQMRRKVELFTYMRFDAEFTFVACTPTG.....
pdb|4N43|A    TAETTLDSFFSRAGLVGEIDLPLEGTTNPNG.....YANWDIDITGYAQMRRKVELFTYMRFDAEFTFVACTPTG.....
pdb|4RQP|A    TAETTLDSFFSRAGLVGEIDLPLEGTTNPNG.....YANWDIDITGYAQMRRKVELFTYMRFDAEFTFVACTPTG.....
pdb|4RQP|M    TAETTLDSFFSRAGLVGEIDLPLEGTTNPNG.....YANWDIDITGYAQMRRKVELFTYMRFDAEFTFVACTPTG.....
pdb|4RQP|I    TAETTLDSFFSRAGLVGEIDLPLEGTTNPNG.....YANWDIDITGYAQMRRKVELFTYMRFDAEFTFVACTPTG.....
pdb|4RQP|Q    TAETTLDSFFSRAGLVGEIDLPLEGTTNPNG.....YANWDIDITGYAQMRRKVELFTYMRFDAEFTFVACTPTG.....
pdb|4RQP|E    TAETTLDSFFSRAGLVGEIDLPLEGTTNPNG.....YANWDIDITGYAQMRRKVELFTYMRFDAEFTFVACTPTG.....
QWY16033.1    TAETTLDSFLSRAGLVGEIDLPLEGTTNPNG.....YANWDIDITGYAQMRRKVELFTYMRFDAEFTFVACTPTG.....
QWY16029.1    TAETTLDSFLSRAGLVGEIDLPLEGTTNPNG.....YANWDIDITGYAQMRRKVELFTYMRFDAEFTFVACTPTG.....
QWY16028.1    TAETTLDSFLSRAGLVGEIDLPLEGTTNPNG.....YANWDIDITGYAQMRRKVELFTYMRFDAEFTFVACTPTG.....
QWY16025.1    TAETTLDSFLSRAGLVGEIDLPLEGTTNPNG.....YANWDIDITGYAQMRRKVELFTYMRFDAEFTFVACTPTG.....
QUS47943.1    TAETTLDSFFSRAGLVGEIDLPLEGTTNPNG.....YANWDIDITGYAQMRRKVEIFTYMRFDAEFTFVACTPTG.....
QOP74914.1    TAETTLDSFFSRAGLVGEIDLPLEGTTNPNG.....YANWDIDITGYAQMRRKVELFTYMRFDAEFTFVACTPTG.....
QUS47916.1    TAETTLDSFFSRAGLVGEIDLPLEGTTNPNG.....YANWDIDITGYAQMRRKVELFTYMRFDAEFTFVACTPTG.....
QWY16148.1    TAETTLDSFFSRAGLVGEIDLPLEGTTNPNG.....YANWDIDITGYAQMRRKVELFTYMRFDAEFTFVACTPTG.....
QOP74906.1    TAETTLDSFFSRAGLVGEIDLPLEGTTNPNG.....YANWDIDITGYAQMRRKVELFTYMRFDAEFTFVACTPTG.....
QOP74895.1    TAETTLDSFFSRAGLVGEIDLPLEGTTNPNG.....YANWDIDITGYAQMRRKVELFTYMRFDAEFTFVACTPTG.....
QOP74890.1    TAETTLDSFFSRAGLVGEIDLPLEGTTNPNG.....YANWDIDITGYAQMRRKVELFTYMRFDAEFTFVACTPTG.....
QOP74889.1    TAETTLDSFFSRAGLVGEIDLPLEGTTNPNG.....YANWDIDITGYAQMRRKVELFTYMRFDAEFTFVACTPTG.....
QWY16149.1    TAETTLDSFFSRAGLVGEIDLPLEGTTNPNG.....YANWDIDITGYAQMRRKVELFTYMRFDAEFTFVACTPTG.....
QOP74888.1    TAETTLDSFFSRAGLVGEIDLPLEGTTNPNG.....YANWDIDITGYAQMRRKVELFTYMRFDAEFTFVACTPTG.....
QOP74894.1    TAETTLDSFFSRAGLVGEIDLPLEGTTNPNG.....YANWDIDITGYAQMRRKVELFTYMRFDAEFTFVACTPTG.....
QUS47930.1    TAETTLDSFFSRAGLVGEIDLPLEGTTNPNG.....YANWDIDITGYAQMRRKVELFTYMRFDAEFTFVACTPTG.....
QWY16053.1    TAETTLDSFFSRAGLVGEIDLPLEGTTNPNG.....YANWDIDITGYAQMRRKVELFTYMRFDAEFTFVACTPTG.....
QWY15997.1    TAETTLDSFFSRAGLVGEIDLPLEGTTNPNG.....YANWDIDITGYAQMRRKVELFTYMRFDAEFTFVACTPTG.....
QWY15996.1    TAETTLDSFFSRAGLVGEIDLPLEGTTNPNG.....YANWDIDITGYAQMRRKVELFTYMRFDAEFTFVACTPTG.....
QWY15995.1    TAETTLDSFFSRAGLVGEIDLPLEGTTNPNG.....YANWDIDITGYAQMRRKVELFTYMRFDAEFTFVACTPTG.....
QTA73878.1    TAETTLDSFFSRAGLVGEIDLPLEGTTNPNG.....YANWDIDITGYAQMRRKVELFTYMRFDAEFTFVACTPTG.....
QTA73877.1    TAETTLDSFFSRAGLVGEIDLPLEGTTNPNG.....YANWDIDITGYAQMRRKVELFTYMRFDAEFTFVACTPTG.....
QTA73876.1    TAETTLDSFFSRAGLVGEIDLPLEGTTNPNG.....YANWDIDITGYAQMRRKVELFTYMRFDAEFTFVACTPTG.....
QUS47914.1    TAETTLDSFFSRAGLVGEIDLPLEGTTNPNG.....YANWDIDITGYAQMRRKVELFTYMRFDAEFTFVACTPTG.....
QWY16003.1    TAETTLDSFFSRAGLVGEIDLPLEGTTNPNG.....YANWDIDITGYAQMRRKVELFTYMRFDAEFTFVACTPTG.....
QWY16067.1    TAETTLDSFFSRAGLVGEIDLPLEGTTNPNG.....YANWDIDITGYAQMRRKVELFTYMRFDAEFTFVACTPTG.....
QWY16057.1    TAETTLDSFFSRAGLVGEIDLPLEGTTNPNG.....YANWDIDITGYAQMRRKVELFTYMRFDAEFTFVACTPTG.....
QTA73888.1    TAETTLDSFFSRAGLVGEIDLPLEGTTNPNG.....YANWDIDITGYAQMRRKVELFTYMRFDAEFTFVACTPTG.....
QWY16086.1    TAETTLDSFFSRAGLVGEIDLPLEGTTNPNG.....YANWDIDITGYAQMRRKVELFTYMRFDAEFTFVACTPTG.....
pdb|4GMP|1    TAETTLDSFFSRAGLVGEIDLPLEGTTNPNG.....YANWDIDITGYAQMRRKVELFTYMRFDAEFTFVACTPTG.....
pdb|3J91|1    TAETTLDSFFSRAGLVGEIDLPLEGTTNPNG.....YANWDIDITGYAQMRRKVELFTYMRFDAEFTFVACTPTG.....
QWY16094.1    TAETTLDSFFSRAGLVGEIDLPLEGTTNPNG.....YANWDIDITGYAQMRRKVELFTYMRFDAEFTFVACTPTG.....
QWY16093.1    TAETTLDSFFSRAGLVGEIDLPLEGTTNPNG.....YANWDIDITGYAQMRRKVELFTYMRFDAEFTFVACTPTG.....
QWY16091.1    TAETTLDSFFSRAGLVGEIDLPLEGTTNPNG.....YANWDIDITGYAQMRRKVELFTYMRFDAEFTFVACTPTG.....
QWY16088.1    TAETTLDSFFSRAGLVGEIDLPLEGTTNPNG.....YANWDIDITGYAQMRRKVELFTYMRFDAEFTFVACTPTG.....
QWY16082.1    TAETTLDSFFSRAGLVGEIDLPLEGTTNPNG.....YANWDIDITGYAQMRRKVELFTYMRFDAEFTFVACTPTG.....
QWY16073.1    TAETTLDSFFSRAGLVGEIDLPLEGTTNPNG.....YANWDIDITGYAQMRRKVELFTYMRFDAEFTFVACTPTG.....
QWY16055.1    TAETTLDSFFSRAGLVGEIDLPLEGTTNPNG.....YANWDIDITGYAQMRRKVELFTYMRFDAEFTFVACTPTG.....
QUS47918.1    TAETTLDSFFSRAGLVGEIDLPLEGTTNPNG.....YANWDIDITGYAQMRRKVELFTYMRFDAEFTFVACTPTG.....
QWY16089.1    TAETTLDSFFSRAGLVGEIDLPLEGTTNPNG.....YANWDIDITGYAQMRRKVELFTYMRFDAEFTFVACTPTG.....
QWY16037.1    TAETTLDSFFSRAGLVGEIDLPLEGTTNPNG.....YANWDIDITGYAQMRRKVELFTYMRFDAEFTFVACTPTG.....
QOP74891.1    TAETTLDSFFSRAGLVGEIDLPLEGTTNPNG.....YANWDIDITGYAQMRRKVELFTYMRFDAEFTFVACTPTG.....
pdb|3VBS|A    TAETTLDSFFSRAGLVGEIDLPLKGTTNPNG.....YANWDIDITGYAQMRRKVELFTYMRFDAEFTFVACTPTG.....
pdb|3VBO|A    TAETTLDSFFSRAGLVGEIDLPLKGTTNPNG.....YANWDIDITGYAQMRRKVELFTYMRFDAEFTFVACTPTG.....
pdb|3VBH|A    TAETTLDSFFSRAGLVGEIDLPLKGTTNPNG.....YANWDIDITGYAQMRRKVELFTYMRFDAEFTFVACTPTG.....
pdb|3VBF|A    TAETTLDSFFSRAGLVGEIDLPLKGTTNPNG.....YANWDIDITGYAQMRRKVELFTYMRFDAEFTFVACTPTG.....
pdb|6LQD|A    TAETTLDSFFSRAGLVGEIDLPLKGTTNPNG.....YANWDIDITGYAQMRRKVELFTYMRFDAEFTFVACTPTG.....
pdb|5ZUF|A    TAETTLDSFFSRAGLVGEIDLPLKGTTNPNG.....YANWDIDITGYAQMRRKVELFTYMRFDAEFTFVACTPTG.....
pdb|5ZUD|A    TAETTLDSFFSRAGLVGEIDLPLKGTTNPNG.....YANWDIDITGYAQMRRKVELFTYMRFDAEFTFVACTPTG.....
pdb|4CDX|A    TAETTLDSFFSRAGLVGEIDLPLKGTTNPNG.....YANWDIDITGYAQMRRKVELFTYMRFDAEFTFVACTPTG.....
pdb|4CDW|A    TAETTLDSFFSRAGLVGEIDLPLKGTTNPNG.....YANWDIDITGYAQMRRKVELFTYMRFDAEFTFVACTPTG.....
pdb|4CDU|A    TAETTLDSFFSRAGLVGEIDLPLKGTTNPNG.....YANWDIDITGYAQMRRKVELFTYMRFDAEFTFVACTPTG.....
pdb|4CDQ|A    TAETTLDSFFSRAGLVGEIDLPLKGTTNPNG.....YANWDIDITGYAQMRRKVELFTYMRFDAEFTFVACTPTG.....
```

```
QOP74885.1    TAETTLDSFFSRAGLVGEIDLPLDGTTNPNG.....YANWDIDITGYAQMRRKVELFTYMRFDAEFTFVACTPTG.....
QWY16026.1    TAETTLDSFFSRAGLVGEIDLPLGGTTNPNG.....YANWDIDITGYAQMRRKVELFTYMRFDAEFTFVACTPTG.....
QWY15977.1    TAETTLDSFFSRAGLVGEIDLPLEGTTNPNG.....YANWDIDITGYAQMRRKVELFTYMRFDAEFTFVACTPTG.....
QTA73869.1    TAETTLDSFFSRAGLVGEIDLPLEGTTNPNG.....YANWDIDITGYAQMRRKVELFTYMRFDAEFTFVACTPTG.....
QUS47935.1    TAETTLDSFFSRAGLVGEIDLPLEGTTNPNG.....YANWDIDITGYAQMRRKVELFTYMRFDAEFTFVACTPTG.....
QWY16024.1    TAETTLDSFFSRAGLVGEIDLPLEGTTNPNG.....YANWDIDITGYAQMRRKVELFTYMRFDAEFTFVACTPTG.....
QWY16023.1    TAETTLDSFFSRAGLVGEIDLPLEGTTNPNG.....YANWDIDITGYAQMRRKVELFTYMRFDAEFTFVACTPTG.....
QWY16022.1    TAETTLDSFFSRAGLVGEIDLPLEGTTNPNG.....YANWDIDITGYAQMRRKVELFTYMRFDAEFTFVACTPTG.....
QWY16013.1    TAETTLDSFFSRAGLVGEIDLPLEGTTNPNG.....YANWDIDITGYAQMRRKVELFTYMRFDAEFTFVACTPTG.....
QWY16012.1    TAETTLDSFFSRAGLVGEIDLPLEGTTNPNG.....YANWDIDITGYAQMRRKVELFTYMRFDAEFTFVACTPTG.....
QWY15992.1    TAETTLDSFFSRAGLVGEIDLPLEGTTNPNG.....YANWDIDITGYAQMRRKVELFTYMRFDAEFTFVACTPTG.....
QWY15982.1    TAETTLDSFFSRAGLVGEIDLPLEGTTNPNG.....YANWDIDITGYAQMRRKVELFTYMRFDAEFTFVACTPTG.....
QWY15981.1    TAETTLDSFFSRAGLVGEIDLPLEGTTNPNG.....YANWDIDITGYAQMRRKVELFTYMRFDAEFTFVACTPTG.....
QWY15976.1    TAETTLDSFFSRAGLVGEIDLPLEGTTNPNG.....YANWDIDITGYAQMRRKVELFTYMRFDAEFTFVACTPTG.....
QTA73880.1    TAETTLDSFFSRAGLVGEIDLPLEGTTNPNG.....YANWDIDITGYAQMRRKVELFTYMRFDAEFTFVACTPTG.....
QTA73872.1    TAETTLDSFFSRAGLVGEIDLPLEGTTNPNG.....YANWDIDITGYAQMRRKVELFTYMRFDAEFTFVACTPTG.....
QTA73868.1    TAETTLDSFFSRAGLVGEIDLPLEGTTNPNG.....YANWDIDITGYAQMRRKVELFTYMRFDAEFTFVACTPTG.....
QOP74892.1    TAETTLDSFFSRAGLVGEIDLPLEGTTNPNG.....YANWDIDITGYAQMRRKVELFTYMRFDAEFTFVACTPTG.....
QWY15998.1    TAETTLDSFFSRAGLVGEIDLPLEGTTNPNG.....YANWDIDITGYAQMRRKVELFTYMRFDAEFTFVACTPTG.....
QWY15994.1    TAETTLDSFFSRAGLVGEIDLPLEGTTNPNG.....YANWDIDITGYAQMRRKVELFTYMRFDAEFTFVACTPTG.....
QTA73879.1    TAETTLDSFFSRAGLVGEIDLPLEGTTNPNG.....YANWDIDITGYAQMRRKVELFTYMRFDAEFTFVACTPTG.....
QTA73875.1    TAETTLDSFFSRAGLVGEIDLPLEGTTNPNG.....YANWDIDITGYAQMRRKVELFTYMRFDAEFTFVACTPTG.....
QWY16006.1    TAETTLDSFFSRAGLVGEIDLPLEGTTNPNG.....YANWDIDITGYAQMRRKVELFTYMRFDAEFTFVACTPTG.....
QWY16005.1    TAETTLDSFFSRAGLVGEIDLPLEGTTNPNG.....YANWDIDITGYAQMRRKVELFTYMRFDAEFTFVACTPTG.....
QWY16039.1    TAETTLDSFFSRAGLVGEIDLPLEGTTNPNG.....YANWDIDITGYAQMRRKVELFTYMRFDAEFTFVACTPTG.....
QWY15999.1    TAETTLDSFFSRAGLVGEIDLPLEGTTNPNG.....YANWDIDITGYAQMRRKVELFTYMRFDAEFTFVACTPTG.....
QWY16004.1    TAETTLDSFFSRAGLVGEIDLPLEGTTNPNG.....YANWDIDITGYAQMRRKVELFTYMRFDAEFTFVACTPTG.....
QOP74913.1    TAETTLDSFFSRAGLVGEIDLPLEGTTNPNG.....YANWDIDITGYAQMRRKVELFTYMRFDAEFTFVACTPTG.....
QOP74915.1    TAETTLDSFFSRAGLVGEIDLPLEGTTNPNG.....YANWDIDITGYAQMRRKVELFTYMRFDAEFTFVACTPTG.....
QWY16035.1    TAETTLDSFFSRAGLVGEIDLPLEGTTNPNG.....YANWDIDITGYAQMRRKVELFTYMRFDAEFTFVACTPTG.....
QWY15990.1    TAETTLDSFFSRAGLVGEIDLPLEGTTNPNG.....YANWDIDITGYAQMRRKVELFTYMRFDAEFTFVACTPTG.....
QWY15988.1    TAETTLDSFFSRAGLVGEIDLPLEGTTNPNG.....YANWDIDITGYAQMRRKVELFTYMRFDAEFTFVACTPTG.....
QWY15989.1    TAETTLDSFFSRAGLVGEIDLPLEGTTNPNG.....YANWDIDITGYAQMRRKVELFTYMRFDAEFTFVACTPTG.....
QWY15987.1    TAETTLDSFFSRAGLVGEIDLPLEGTTNPNG.....YANWDIDITGYAQMRRKVELFTYMRFDAEFTFVACTPTG.....
QWY15978.1    TAETTLDSFFSRAGLVGEIDLPLEGTTNPNG.....YANWDIDITGYAQMRRKVELFTYMRFDAEFTFVACTPTG.....
QTA73870.1    TAETTLDSFFSRAGLVGEIDLPLEGTTNPNG.....YANWDIDITGYAQMRRKVELFTYMRFDAEFTFVACTPTG.....
QWY15983.1    TAETTLDSFFSRAGLVGEIDLPLEGTTNPNG.....YANWDIDITGYAQMRRKVELFTYMRFDAEFTFVACTPTG.....
QUS47931.1    TAETTLDSFFSRAGLVGEIDLPLEGTTNPNG.....YANWDIDITGYAQMRRKVELFTYMRFDAEFTFVACTPTG.....
QUS47925.1    TAETTLDSFFSRAGLVGEIDLPLEGTTNPNG.....YANWDIDITGYAQMRRKVELFTYMRFDAEFTFVACTPTG.....
QUS47922.1    TAETTLDSFFSRAGLVGEIDLPLEGTTNPNG.....YANWDIDITGYAQMRRKVELFTYMRFDAEFTFVACTPTG.....
QWY16014.1    TAETTLDSFFSRAGLVGEIDLPLEGTTNPNG.....YANWDIDITGYAQMRRKVELFTYMRFDAEFTFVACTPTG.....
QTA73881.1    TAETTLDSFFSRAGLVGEIDLPLEGTTNPNG.....YANWDIDITGYAQMRRKVELFTYMRFDAEFTFVACTPTG.....
QOP74898.1    TAETTLDSFFSRAGLVGEIDLPLEGTTNPNG.....YANWDIDITGYAQMRRKVELFTYMRFDAEFTFVACTPTG.....
QWY15980.1    TAETTLDSFFSRAGLVGEIDLPLEGTTNPNG.....YANWDIDITGYAQMRRKVELFTYMRFDAEFTFVACTPTG.....
QTA73873.1    TAETTLDSFFSRAGLVGEIDLPLEGTTNPNG.....YANWDIDITGYAQMRRKVELFTYMRFDAEFTFVACTPTG.....
QWY16011.1    TAETTLDSFFSRAGLVGEIDLPLEGTTNPNG.....YANWDIDITGYAQMRRKVELFTYMRFDAEFTFVACTPTG.....
QWY16010.1    TAETTLDSFFSRAGLVGEIDLPLEGTTNPNG.....YANWDIDITGYAQMRRKVELFTYMRFDAEFTFVACTPTG.....
QOP74911.1    TAETTLDSFFSRAGLVGEIDLPLEGTTNPNG.....YANWDIDITGYAQMRRKVELFTYMRFDAEFTFVACTPTG.....
QWY16019.1    TAETALDSFFSRAGLVGEIDLPLEGTTNPNG.....YANWDIDITGYAQMRRKVELFTYMRFDAEFTFVACTPTG.....
QOP74883.1    TAETTLDSFFSRAGLVGEIDLPLEGTTNPNG.....YANWDIDITGYAQMRRKVELFTYMRFDAEFTFVACTPTG.....
QUS47909.1    TAETTLDSFFSRAGLVGEIDLPLEGTTNPNG.....YANWDIDITGYAQMRRKVELFTYMRFDAEFTFVACTPTG.....
QWY16144.1    TAETTLDSFFSRAGLVGEIDLPLEGTTNPNG.....YANWDIDITGYAQMRRKVELFTYMRFDAEFTFVACTPTG.....
QUS47908.1    TAETTLDSFFSRAGLVGEIDLPLEGTTNPNG.....YANWDIDITGYAQMRRKVELFTYMRFDAEFTFVACTPTG.....
QNN25844.1    TAETTLDSFFSRAGLVGEIDLPLEGTTNPNG.....YANWDIDITGYAQMRRKVELFTYMRFDAEFTFVACTPTG.....
QNN25840.1    TAETTLDSFFSRAGLVGEIDLPLEGTTNPNG.....YANWDIDITGYAQMRRKVELFTYMRFDAEFTFVACTPTG.....
QNN25839.1    TAETTLDSFFSRAGLVGEIDLPLEGTTNPNG.....YANWDIDITGYAQMRRKVELFTYMRFDAEFTFVACTPTG.....
QNN25835.1    TAETTLDSFFSRAGLVGEIDLPLEGTTNPNG.....YANWDIDITGYAQMRRKVELFTYMRFDAEFTFVACTPTG.....
QNN25834.1    TAETTLDSFFSRAGLVGEIDLPLEGTTNPNG.....YANWDIDITGYAQMRRKVELFTYMRFDAEFTFVACTPTG.....
QOP74912.1    TAETTLDSFFSRAGLVGEIDLPLEGTTNPNG.....YANWDIDITGYAQMRRKVELFTYMRFDAEFTFVACTPTG.....
QUS47910.1    TAETTLDSFFSRAGLVGEIDLPLEGTTNPNG.....YANWDIDITGYAQMRRKVELFTYMRFDAEFTFVACTPTG.....
QUS47907.1    TAETTLDSFFSRAGLVGEIDLPLEGTTNPNG.....YANWDIDITGYAQMRRKVELFTYMRFDAEFTFVACTPTG.....
QWY16141.1    TAETTLDSFFSRAGLVGEIDLPLEGTTNPNG.....YANWDIDITGYAQMRRKVELFTYMRFDAEFTFVACTPTG.....
QWY16140.1    TAETTLDSFFSRAGLVGEIDLPLEGTTNPNG.....YANWDIDITGYAQMRRKVELFTYMRFDAEFTFVACTPTG.....
QUS47937.1    TAETTLDSFFSRAGLVGEIDLPLEGTTNPNG.....YANWDIDITGYAQMRRKVELFTYMRFDAEFTFVACTPTG.....
QUS47936.1    TAETALDSFFSRAGLVGEIDLPLEGTTNPNG.....YANWDIDITGYAQMRRKVELFTYMRFDAEFTFVACTPTG.....
QUS47912.1    TAETTLDSFFSRAGLVGEIDLPLEGTTNPNG.....YANWDIDITGYAQMRRKVELFTYMRFDAEFTFVACTPTG.....
QUS47906.1    TAETTLDSFFSRAGLVGEIDLPLKGTTNPNG.....YANWDIDITGYAQMRRKVELFTYMRFDAEFTFVACTPTG.....
pdb|4YVW|M    TAETTLDSFFSRAGLVGEIDLPLEGTTNPNG.....YANWDIDITGYAQMRRKVELFTYMRFDAEFTFVACTPTG.....
pdb|4YVW|J    TAETTLDSFFSRAGLVGEIDLPLEGTTNPNG.....YANWDIDITGYAQMRRKVELFTYMRFDAEFTFVACTPTG.....
pdb|4YVW|D    TAETTLDSFFSRAGLVGEIDLPLEGTTNPNG.....YANWDIDITGYAQMRRKVELFTYMRFDAEFTFVACTPTG.....
pdb|4YVW|A    TAETTLDSFFSRAGLVGEIDLPLEGTTNPNG.....YANWDIDITGYAQMRRKVELFTYMRFDAEFTFVACTPTG.....
pdb|4YVW|E    TAETTLDSFFSRAGLVGEIDLPLEGTTNPNG.....YANWDIDITGYAQMRRKVELFTYMRFDAEFTFVACTPTG.....
QPI19797.1    TAETTLDSFFSRAGLVGEIDLPLEGTTNPNG.....YANWDIDITGYAQMRRKVELFTYMRFDAEFTFVACTPTG.....
QPI19781.1    TAETTLDSFFSRAGLVGEIDLPLEGTTNPNG.....YANWDIDITGYAQMRRKVELFTYMRFDAEFTFVACTPTG.....
QPI19795.1    TAETTLDSFFSRAGLVGEIDLPLEGTTNPNG.....YANWDIDITGYAQMRRKVELFTYMRFDAEFTFVACTPTG.....
QPI19794.1    TAETTLDSFFSRAGLVGEIDLPLEGTTNPNG.....YANWDIDITGYAQMRRKVELFTYMRFDAEFTFVACTPTG.....
QPI19793.1    TAETTLDSFFSRAGLVGEIDLPLEGTTNPNG.....YANWDIDITGYAQMRRKVELFTYMRFDAEFTFVACTPTG.....
QPI19792.1    TAETTLDSFFSRAGLVGEIDLPLEGTTNPNG.....YANWDIDITGYAQMRRKVELFTYMRFDAEFTFVACTPTG.....
QPI19790.1    TAETTLDSFFSRAGLVGEIDLPLEGTTNPNG.....YANWDIDITGYAQMRRKVELFTYMRFDAEFTFVACTPTG.....
QPI19789.1    TAETTLDSFFSRAGLVGEIDLPLEGTTNPNG.....YANWDIDITGYAQMRRKVELFTYMRFDAEFTFVACTPTG.....
QPI19788.1    TAETTLDSFFSRAGLVGEIDLPLEGTTNPNG.....YANWDIDITGYAQMRRKVELFTYMRFDAEFTFVACTPTG.....
QPI19787.1    TAETTLDSFFSRAGLVGEIDLPLEGTTNPNG.....YANWDIDITGYAQMRRKVELFTYMRFDAEFTFVACTPTG.....
QPI19785.1    TAETTLDSFFSRAGLVGEIDLPLEGTTNPNG.....YANWDIDITGYAQMRRKVELFTYMRFDAEFTFVACTPTG.....
QPI19784.1    TAETTLDSFFSRAGLVGEIDLPLEGTTNPNG.....YANWDIDITGYAQMRRKVELFTYMRFDAEFTFVACTPTG.....
QPI19782.1    TAETTLDSFFSRAGLVGEIDLPLEGTTNPNG.....YANWDIDITGYAQMRRKVELFTYMRFDAEFTFVACTPTG.....
QPI19780.1    TAETTLDSFFSRAGLVGEIDLPLEGTTNPNG.....YANWDIDITGYAQMRRKVELFTYMRFDAEFTFVACTPTG.....
QPI19777.1    TAETTLDSFFSRAGLVGEIDLPLEGTTNPNG.....YANWDIDITGYAQMRRKVELFTYMRFDAEFTFVACTPTG.....
QPI19762.1    TAETTLDSFFSRAGLVGEIDLPLEGTTNPNG.....YANWDIDITGYAQMRRKVELFTYMRFDAEFTFVACTPTG.....
QPI19755.1    TAETTLDSFFSRAGLVGEIDLPLEGTTNPNG.....YANWDIDITGYAQMRRKVELFTYMRFDAEFTFVACTPTG.....
QPI19754.1    TAETTLDSFFSRAGLVGEIDLPLEGTTNPNG.....YANWDIDITGYAQMRRKVELFTYMRFDAEFTFVACTPTG.....
QPI19748.1    TAETTLDSFFSRAGLVGEIDLPLEGTTNPNG.....YANWDIDITGYAQMRRKVELFTYMRFDAEFTFVACTPTG.....
QPI19747.1    TAETTLDSFFSRAGLVGEIDLPLEGTTNPNG.....YANWDIDITGYAQMRRKVELFTYMRFDAEFTFVACTPTG.....
QPI19744.1    TAETTLDSFFSRAGLVGEIDLPLEGTTNPNG.....YANWDIDITGYAQMRRKVELFTYMRFDAEFTFVACTPTG.....
QPI19743.1    TAETTLDSFFSRAGLVGEIDLPLEGTTNPNG.....YANWDIDITGYAQMRRKVELFTYMRFDAEFTFVACTPTG.....
QPI19739.1    TAETTLDSFFSRAGLVGEIDLPLEGTTNPNG.....YANWDIDITGYAQMRRKVELFTYMRFDAEFTFVACTPTG.....
QPI19778.1    TAETTLDSFFSRAGLVGEIDLPLEGTTNPNG.....YANWDIDITGYAQMRRKVELFTYMRFDAEFTFVACTPTG.....
QPI19796.1    TAETTLDSFFSRAGLVGEIDLPLEGTTNPSG.....YANWDIDITGYAQMRRKVELFTYMRFDAEFTFVACTPTG.....
QPI19753.1    TAETTLDSFFSRAGLVGEIDLPLEGTTNPNG.....YANWDIDITGYAQMRRKVELFTYMRFDAEFTFVACTPTG.....
QPI19791.1    TAETTLDSFFSRAGLVGGIDLPLEGTTNPNG.....YANWDIDITGYAQMRRKVELFTYMRFDAEFTFVACTPTG.....
QPI19786.1    TAETTLDSFFSRAGLVGEIDLPLEGTTNPNG.....YANWDIDITGYAQMRRKVELFTYMRFDAEFTFVACTPTG.....
QPI19779.1    TAETTLDSFFSRAGLVGEIDLPLEGTTNPNG.....YANWDIDITGYAQMRRKVELFTYMRFDAEFTFVACTPTG.....
QPI19742.1    TAETTLDSFFSRAGLVGEIDLPLEGTTNPNG.....YANWDIDVTGYAQMRRKVELFTYMRFDAEFTFVACTPTG.....
pdb|4AED|A    TAETTLDSFFSRAGLVGEIDLPLEGTTNPNG.....YANWDIDITGYAQMRRKVELFTYMRFDAEFTFVACTPTG.....
pdb|3ZFF|A    TAETTLDSFFSRAGLVGEIDLPLEGTTNPNG.....YANWDIDITGYAQMRRKVELFTYMRFDAEFTFVACTPTG.....
pdb|6DIZ|A    TAETTLDSFFSRAGLVGEIDLPLEGTTNPNG.....YANWDIDITGYAQMRRKVELFTYMRFDAEFTFVACTPTG.....
pdb|6UH7|A    TAETTLDSFFSRAGLVGEIDLPLEGTTNPNG.....YANWDIDITGYAQMRRKVELFTYMRFDAEFTFVACTPTG.....
pdb|6UH6|A    TAETTLDSFFSRAGLVGEIDLPLEGTTNPNG.....YANWDIDITGYAQMRRKVELFTYMRFDAEFTFVACTPTG.....
pdb|6UH1|A    TAETTLDSFFSRAGLVGEIDLPLEGTTNPNG.....YANWDIDITGYAQMRRKVELFTYMRFDAEFTFVACTPTG.....
QPI19783.1    TAETTLDSFFSRAGLVGEIDLPLEGTTNPNG.....YANWDIDITGYAQMRRKVELFTYMRFDAEFTFVACTPTG.....
QPI19772.1    TAETTLDSFFSRAGLVGEIDLPLEGTTNPNG.....YANWDIDITGYAQMRRKVELFTYMRFDAEFTFVACTPTG.....
QPI19771.1    TAETTLDSFFSRAGLVGEIDLPLEGTTNPNG.....YANWDIDITGYAQMRRKVELFTYMRFDAEFTFVACTPTG.....
QPI19764.1    TAETTLDSFFSRAGLVGEIDLPLEGTTNPNG.....YANWDIDITGYAQMRRKVELFTYMRFDAEFTFVACTPTG.....
QPI19760.1    TAETTLDSFFSRAGLVGEIDLPLEGTTNPNG.....YANWDIDITGYAQMRRKVELFTYMRFDAEFTFVACTPTG.....
QPI19759.1    TAETTLDSFFSRAGLVGEIDLPLEGTTNPNG.....YANWDIDITGYAQMRRKVELFTYMRFDAEFTFVACTPTG.....
QPI19756.1    TAETTLDSFFSRAGLVGEIDLPLEGTTNPNG.....YANWDIDITGYAQMRRKVELFTYMRFDAEFTFVACTPTG.....
QPI19752.1    TAETTLDSFFSRAGLVGEIDLPLEGTTNPNG.....YANWDIDITGYAQMRRKVELFTYMRFDAEFTFVACTPTG.....
QPI19740.1    TAETTLDSFFSRAGLVGEIDLPLEGTTNPNG.....YANWDIDITGYAQMRRKVELFTYMRFDAEFTFVACTPTG.....
QPI19738.1    TAETTLDSFFSRAGLVGEIDLPLEGTTNPNG.....YANWDIDITGYAQMRRKVELFTYMRFDAEFTFVACTPTG.....
QPI19757.1    TAETTLDSFFSRAGLVGEIDLPLEGTTNPNG.....CANWDIDITGYAQMRRKVELFTYMRFDAEFTFVACTPTG.....
QPI19750.1    TAETTLDSFFSRAGLVGEIDLPLEGTTNPNG.....CANWDIDITGYAQMRRKVELFTYMRFDAEFTFVACTPTG.....
QPI19751.1    TAETTLDSFFSRAGLVGEIDLPLEGTTNPNG.....YANWDIDITGYAQMRRKVELFTYMRFDAEFTFVACTPTG.....
QPI19746.1    TAETTLDSFFSRAGLVGEIDLPLEGTTNPNG.....YANWDIDITGYAQMRRKVELFTYMRFDAEFTFVACTPTG.....
QPI19737.1    TAETTLDSFFSRAGLVGEIDLPLEGTTNPNG.....YANWDIDITGYAQMRRKVELFTYMRFDAEFTFVACTPTG.....
QPI19776.1    TAETTLDSFFSRAGLVGEIDLPLEGTTNPNG.....YANWDIDITGYAQMRRKVELFTYMRFDAEFTFVACTPTG.....
QPI19774.1    TAETTLDSFFSRAGLVGEIDLPLEGTTNPNG.....YANWDIDITGYAQMRRKVELFTYMRFDAEFTFVACTPTG.....
QPI19773.1    TAETTLDSFFSRAGLVGEIDLPLEGTTNPNG.....YANWDIDITGYAQMRRKVELFTYMRFDAEFTFVACTPTG.....
QPI19770.1    TAETTLDSFFSRAGLVGEIDLPLEGTTNPNG.....YANWDIDITGYAQMRRKVELFTYMRFDAEFTFVACTPTG.....
QPI19769.1    TAETTLDSFFSRAGLVGEIDLPLEGTTNPNG.....YANWDIDITGYAQMRRKVELFTYMRFDAEFTFVACTPTG.....
QPI19768.1    TAETTLDSFFSRAGLVGEIDLPLEGTTNPNG.....YANWDIDITGYAQMRRKVELFTYMRFDAEFTFVACTPTG.....
QPI19767.1    TAETTLDSFFSRAGLVGEIDLPLEGTTNPNG.....YANWDIDITGYAQMRRKVELFTYMRFDAEFTFVACTPTG.....
QPI19766.1    TAETTLDSFFSRAGLVGEIDLPLEGTTNPNG.....YANWDIDITGYAQMRRKVELFTYMRFDAEFTFVACTPTG.....
QPI19763.1    TAETTLDSFFSRAGLVGEIDLPLEGTTNPNG.....YANWDIDITGYAQMRRKVELFTYMRFDAEFTFVACTPTG.....
QPI19761.1    TAETTLDSFFSRAGLVGEIDLPLEGTTNPNG.....YANWDIDITGYAQMRRKVELFTYMRFDAEFTFVACTPTG.....
QPI19765.1    TAETTLDSFFSRAGLVGEIDLPLEGTTNPNG.....YANWDIDITGYAQMRRKVELFTYMRFDAEFTLVACTPTG.....
QPI19775.1    TAETTLDSFFSRAGLVGEIDLPPEGTTNPNG.....YANWDIDITGYAQMRRKVELFTYMRFDAEFTFVACTPTG.....
QPI19758.1    TAETTLDSFFSRAGLVGEIDLPLEGTTNPNG.....YANWDIDITGYAQMRRKVELFTYMRFDAEFTFVACTPTG.....
QPI19736.1    TAETTLDSFFSRAGLVGEIDLPLEGTTNPNG.....YANWDIDITGYAQMRRKVELFTYMRFDAEFTFVACTPTG.....
QPI19735.1    TAETTLDSFFSRAGLVGEIDLPLEGTTNPNG.....YANWDIDITGYAQMRRKVELFTYMRFDAEFTFVACTPTG.....
QPI19749.1    TAETTLDSFFSRAGLVGEIDLPLEGTTNPNG.....YANWDIDITGYAQMRRKVELFTYMRFDAEFTFVACTPTG.....
QPI19741.1    TAETTLDSFFSRAGLVGEIDLPLEGTTNPNG.....YANWDIDITGYAQMRRKVELFTYMRFDAEFTFVACTPTG.....
QPI19745.1    TAETTLDSFFSRAGLVGEIDLPLEGTTNPNG.....YANWDIDITGYAQMRRKVELFTYMRFDAEFTFVACTPTG.....
pdb|6Z3Q|A    TAETTLDSFFSRAGLVGEIDLPLEGTTNPNG.....YANWDIDITGYAQMRRKVELFTYMRFDAEFTFVACTPTG.....
pdb|6Z3P|A    TAETTLDSFFSRAGLVGEIDLPLEGTTNPNG.....YANWDIDITGYAQMRRKVELFTYMRFDAEFTFVACTPTG.....
pdb|6I2K|A    TAETTLDSFFSRAGLVGEIDLPLEGTTNPSG.....YANWDIDITGYAQMRRKVELFTYMRFDAEFTFVACTPTG.....
```

```
QWF08740.1    TQETAIGNFFSRAGLVSIITMPTTGTQNTDG.....YVNWDIDLMGYAQLRRKCELFTYMRFDAEFTFVVAKPNG.....
QWF08739.1    TQETAIGNFFSRAGLVSIITMPTTGTQNTDG.....YVNWDIDLMGYAQLRRKCELFTYMRFDAEFTFVVAKPNG.....
QWF08760.1    TQETAIGNFFSRAGLVSIITMPTTGTQNTDG.....YVNWDIDLMGYAQLRRKCELFTYMRFDAEFTFVVAKPNG.....
QWF08759.1    TQETAIGNFFSRAGLVSIITMPTTGTQNTDG.....YVNWDIDLMGYAQLRRKCELFTYMRFDAEFTFVVAKPNG.....
QWF08762.1    TQETAIGNFFSRAGLVSIITMPTTGTQNTDG.....YVNWDIDLMGYAQLRRKCELFTYMRFDAEFTFVVAKPNG.....
QWF08761.1    TQETAIGNFFSRAGLVSIITMPTTGTQNTDG.....YVNWDIDLMGYAQLRRKCELFTYMRFDAEFTFVVAKPNG.....
QWF08764.1    TQETAIGNFFSRAGLVSIITMPTTGTQNTDG.....YVNWDIDLMGYAQLRRKCELFTYMRFDAEFTFVVAKPNG.....
QWF08763.1    TQETAIGNFFSRAGLVSIITMPTTGTQNTDG.....YVNWDIDLMGYAQLRRKCELFTYMRFDAEFTFVVAKPNG.....
QWF08773.1    TQETAIGNFFSRAGLVSIITMPTTGTQNTDG.....YVNWDIDLMGYAQLRRKCELFTYMRFDAEFTFVVAKPNG.....
QWF08749.1    TQETAIGNFFSRAGLVSIITMPTTGTQNTDG.....YVNWDIDLMGYAQLRRKCELFTYMRFDAEFTFVVAKPNG.....
QWF08785.1    TQETAIGNFFSRAGLVSIITMPTTGTQNTDG.....YVNWDIDLMGYAQLRRKCELFTYMRFDAEFTFVVAKPNG.....
QWF08783.1    TQETAIGNFFSRAGLVSIITMPTTGTQNTDG.....YVNWDIDLMGYAQLRRKCELFTYMRFDAEFTFVVAKPNG.....
QWF08787.1    TQETAIGNFFSRAGLVSIITMPTTGTQNTDG.....YVNWDIDLMGYAQLRRKCELFTYMRFDAEFTFVVAKPNG.....
QWF08765.1    TQETAIGNFFSRAGLVSIITMPTTGTQNTDG.....YVNWDIDLMGYAQLRRKCELFTYMRFDAEFTFVVAKPNG.....
QWF08792.1    TQETAIGNFFSRAGLVSIITMPTTGTQNTDG.....YVNWDIDLMGYAQLRRKCELFTYMRFDAEFTFVVAKPNG.....
QWF08767.1    TQETAIGNFFSRAGLVSIITMPTTGTQNTDG.....YVNWDIDLMGYAQLRRKCELFTYMRFDAEFTFVVAKPNG.....
QWF08793.1    TQETAIGNFFSRAGLVSIITMPTTGTQNTDG.....YVNWDIDLMGYAQLRRKCELFTYMRFDAEFTFVVAKPNG.....
QWF08758.1    TQETAIGNFFSRAGLVSIITMPTTGTQNTDG.....YVNWDIDLMGYAQLRRKCELFTYMRFDAEFTFVVAKPNG.....
QWF08845.1    TQETAIGNFFSRAGLVSIITMPTTGTQNTDG.....YVNWDIDLMGYAQLRRKCELFTYMRFDAEFTFVVAKPNG.....
QWF08844.1    TQETAIGNFFSRAGLVSIITMPTTGTQNTDG.....YVNWDIDLMGYAQLRRKCELFTYMRFDAEFTFVVAKPNG.....
QWF08847.1    TQETAIGNFFSRAGLVSIITMPTTGTQNTDG.....YVNWDIDLMGYAQLRRKCELFTYMRFDAEFTFVVAKPNG.....
QWF08846.1    TQETAIGNFFSRAGLVSIITMPTTGTQNTDG.....YVNWDIDLMGYAQLRRKCELFTYMRFDAEFTFVVAKPNG.....
QWF08852.1    TQETAIGNFFSRAGLVSIITMPTTGTQNTDG.....YVNWDIDLMGYAQLRRKCELFTYMRFDAEFTFVVAKPNG.....
QWF08843.1    TQETAIGNFFSRAGLVSIITMPTTGTQNTDG.....YVNWDIDLMGYAQLRRKCELFTYMRFDAEFTFVVAKPNG.....
QWF08857.1    TQETAIGNFFSRAGLVSIITMPTTGTQNTDG.....YVNWDIDLMGYAQLRRKCELFTYMRFDAEFTFVVAKPNG.....
QWF08856.1    TQETAIGNFFSRAGLVSIITMPTTGTQNTDG.....YVNWDIDLMGYAQLRRKCELFTYMRFDAEFTFVVAKPNG.....
QWF08865.1    TQETAIGNFFSRAGLVSIITMPTTGTQNTDG.....YVNWDIDLMGYAQLRRKCELFTYMRFDAEFTFVVAKPNG.....
QWF08738.1    TQETAIGNFFSRAGLVSIITMPTTGTQNTDG.....YVNWDIDLMGYAQLRRKCELFTYMRFDAEFTFVVAKPNG.....
QWF08872.1    TQETAIGNFFSRAGLVSIITMPTTGTQNTDG.....YVNWDIDLMGYAQLRRKCELFTYMRFDAEFTFVVAKPNG.....
QWF08871.1    TQETAIGNFFSRAGLVSIITMPTTGTQNTDG.....YVNWDIDLMGYAQLRRKCELFTYMRFDAEFTFVVAKPNG.....
QWF08879.1    TQETAIGNFFSRAGLVSIITMPTTGTQNTDG.....YVNWDIDLMGYAQLRRKCELFTYMRFDAEFTFVVAKPNG.....
QWF08877.1    TQETAIGNFFSRAGLVSIITMPTTGTQNTDG.....YVNWDIDLMGYAQLRRKCELFTYMRFDAEFTFVVAKPNG.....
QWF08880.1    TQETAIGNFFSRAGLVSIITMPTTGTQNTDG.....YVNWDIDLMGYAQLRRKCELFTYMRFDAEFTFVVAKPNG.....
QWF08851.1    TQETAIGNFFSRAGLVSIITMPTTGTQNTDG.....YVNWDIDLMGYAQLRRKCELFTYMRFDAEFTFVVAKPNG.....
QWF08882.1    TQETAIGNFFSRAGLVSIITMPTTGTQNTDG.....YVNWDIDLMGYAQLRRKCELFTYMRFDAEFTFVVAKPNG.....
QWF08867.1    TQETAIGNFFSRAGLVSIITMPTTGTQNTDG.....YVNWDIDLMGYAQLRRKCELFTYMRFDAEFTFVVAKPNG.....
QWF08884.1    TQETAIGNFFSRAGLVSIITMPTTGTQNTDG.....YVNWDIDLMGYAQLRRKCELFTYMRFDAEFTFVVAKPNG.....
QWF08875.1    TQETAIGNFFSRAGLVSIITMPTTGTQNTDG.....YVNWDIDLMGYAQLRRKCELFTYMRFDAEFTFVVAKPNG.....
QWF08885.1    TQETAIGNFFSRAGLVSIITMPTTGTQNTDG.....YVNWDIDLMGYAQLRRKCELFTYMRFDAEFTFVVAKPNG.....
QWF08863.1    TQETAIGNFFSRAGLVSIITMPTTGTQNTDG.....YVNWDIDLMGYAQLRRKCELFTYMRFDAEFTFVVAKPNG.....
QWF08890.1    TQETAIGNFFSRAGLVSIITMPTTGTQNTDG.....YVNWDIDLMGYAQLRRKCELFTYMRFDAEFTFVVAKPNG.....
QWF08889.1    TQETAIGNFFSRAGLVSIITMPTTGTQNTDG.....YVNWDIDLMGYAQLRRKCELFTYMRFDAEFTFVVAKPNG.....
QWF08896.1    TQETAIGNFFSRAGLVSIITMPTTGTQNTDG.....YVNWDIDLMGYAQLRRKCELFTYMRFDAEFTFVVAKPNG.....
QWF08752.1    TQETAIGNFFSRAGLVSIITMPTTGTQNTDG.....YVNWDIDLMGYAQLRRKCELFTYMRFDAEFTFVVAKPNG.....
QWF08900.1    TQETAIGNFFSRAGLVSIITMPTTGTQNTDG.....YVNWDIDLMGYAQLRRKCELFTYMRFDAEFTFVVAKPNG.....
QWF08899.1    TQETAIGNFFSRAGLVSIITMPTTGTQNTDG.....YVNWDIDLMGYAQLRRKCELFTYMRFDAEFTFVVAKPNG.....
QWF08908.1    TQETAIGNFFSRAGLVSIITMPTTGTQNTDG.....YVNWDIDLMGYAQLRRKCELFTYMRFDAEFTFVVAKPNG.....
QWF08906.1    TQETAIGNFFSRAGLVSIITMPTTGTQNTDG.....YVNWDIDLMGYAQLRRKCELFTYMRFDAEFTFVVAKPNG.....
QWF08910.1    TQETAIGNFFSRAGLVSIITMPTTGTQNTDG.....YVNWDIDLMGYAQLRRKCELFTYMRFDAEFTFVVAKPNG.....
QWF08870.1    TQETAIGNFFSRAGLVSIITMPTTGTQNTDG.....YVNWDIDLMGYAQLRRKCELFTYMRFDAEFTFVVAKPNG.....
QWF08915.1    TQETAIGNFFSRAGLVSIITMPTTGTQNTDG.....YVNWDIDLMGYAQLRRKCELFTYMRFDAEFTFVVAKPNG.....
QWF08750.1    TQETAIGNFFSRAGLVSIITMPTTGTQNTDG.....YVNWDIDLMGYAQLRRKCELFTYMRFDAEFTFVVAKPNG.....
QWF08917.1    TQETAIGNFFSRAGLVSIITMPTTGTQNTDG.....YVNWDIDLMGYAQLRRKCELFTYMRFDAEFTFVVAKPNG.....
QWF08916.1    TQETAIGNFFSRAGLVSIITMPTTGTQNTDG.....YVNWDIDLMGYAQLRRKCELFTYMRFDAEFTFVVAKPNG.....
QWF08923.1    TQETAIGNFFSRAGLVSIITMPTTGTQNTDG.....YVNWDIDLMGYAQLRRKCELFTYMRFDAEFTFVVAKPNG.....
QWF08921.1    TQETAIGNFFSRAGLVSIITMPTTGTQNTDG.....YVNWDIDLMGYAQLRRKCELFTYMRFDAEFTFVVAKPNG.....
QWF08926.1    TQETAIGNFFSRAGLVSIITMPTTGTQNTDG.....YVNWDIDLMGYAQLRRKCELFTYMRFDAEFTFVVAKPNG.....
QWF08780.1    TQETAIGNFFSRAGLVSIITMPTTGTQNTDG.....YVNWDIDLMGYAQLRRKCELFTYMRFDAEFTFVVAKPNG.....
QWF08932.1    TQETAIGNFFSRAGLVSIITMPTTGTQNTDG.....YVNWDIDLMGYAQLRRKCELFTYMRFDAEFTFVVAKPNG.....
QWF08931.1    TQETAIGNFFSRAGLVSIITMPTTGTQNTDG.....YVNWDIDLMGYAQLRRKCELFTYMRFDAEFTFVVAKPNG.....
QWF08945.1    TQETAIGNFFSRAGLVSIITMPTTGTQNTDG.....YVNWDIDLMGYAQLRRKCELFTYMRFDAEFTFVVAKPNG.....
QWF08938.1    TQETAIGNFFSRAGLVSIITMPTTGTQNTDG.....YVNWDIDLMGYAQLRRKCELFTYMRFDAEFTFVVAKPNG.....
QWF08955.1    TQETAIGNFFSRAGLVSIITMPTTGTQNTDG.....YVNWDIDLMGYAQLRRKCELFTYMRFDAEFTFVVAKPNG.....
QWF08954.1    TQETAIGNFFSRAGLVSIITMPTTGTQNTDG.....YVNWDIDLMGYAQLRRKCELFTYMRFDAEFTFVVAKPNG.....
QWF08967.1    TQETAIGNFFSRAGLVSIITMPTTGTQNTDG.....YVNWDIDLMGYAQLRRKCELFTYMRFDAEFTFVVAKPNG.....
QWF08959.1    TQETAIGNFFSRAGLVSIITMPTTGTQNTDG.....YVNWDIDLMGYAQLRRKCELFTYMRFDAEFTFVVAKPNG.....
QYC65038.1    TQETAIGNFFSRAGLVSIITMPTTGTQNTDG.....YVNWDIDLMGYAQLRRKCELFTYMRFDAEFTFVVAKPNG.....
QYC65037.1    TQETAIGNFFSRAGLVSIITMPTTGTQNTDG.....YVNWDIDLMGYAQLRRKCELFTYMRFDAEFTFVVAKPNG.....
QYC65039.1    TQETAIGNFFSRAGLVSIITMPTTGTQNTDG.....YVNWDIDLMGYAQLRRKCELFTYMRFDAEFTFVVAKPNG.....
QYC65034.1    TQETAIGNFFSRAGLVSIITMPTTGTQNTDG.....YVNWDIDLMGYAQLRRKCELFTYMRFDAEFTFVVAKPNG.....
QYC65043.1    TQETAIGNFFSRAGLVSIITMPTTGTQNTDG.....YVNWDIDLMGYAQLRRKCELFTYMRFDAEFTFVVAKPNG.....
QYC65041.1    TQETAIGNFFSRAGLVSIITMPTTGTQNTDG.....YVNWDIDLMGYAQLRRKCELFTYMRFDAEFTFVVAKPNG.....
QYC65047.1    TQETAIGNFFSRAGLVSIITMPTTGTQNTDG.....YVNWDIDLMGYAQLRRKCELFTYMRFDAEFTFVVAKPNG.....
QYC65046.1    TQETAIGNFFSRAGLVSIITMPTTGTQNTDG.....YVNWDIDLMGYAQLRRKCELFTYMRFDAEFTFVVAKPNG.....
QYC65050.1    TQETAIGNFFSRAGLVSIITMPTTGTQNTDG.....YVNWDIDLMGYAQLRRKCELFTYMRFDAEFTFVVAKPNG.....
QYC65049.1    TQETAIGNFFSRAGLVSIITMPTTGTQNTDG.....YVNWDIDLMGYAQLRRKCELFTYMRFDAEFTFVVAKPNG.....
QYC65051.1    TQETAIGNFFSRAGLVSIITMPTTGTQNTDG.....YVNWDIDLMGYAQLRRKCELFTYMRFDAEFTFVVAKPNG.....
QYC65045.1    TQETAIGNFFSRAGLVSIITMPTTGTQNTDG.....YVNWDIDLMGYAQLRRKCELFTYMRFDAEFTFVVAKPNG.....
QYC65058.1    TQETAIGNFFSRAGLVSIITMPTTGTQNTDG.....YVNWDIDLMGYAQLRRKCELFTYMRFDAEFTFVVAKPNG.....
QYC65055.1    TQETAIGNFFSRAGLVSIITMPTTGTQNTDG.....YVNWDIDLMGYAQLRRKCELFTYMRFDAEFTFVVAKPNG.....
QYC65060.1    TQETAIGNFFSRAGLVSIITMPTTGTQNTDG.....YVNWDIDLMGYAQLRRKCELFTYMRFDAEFTFVVAKPNG.....
QYC65059.1    TQETAIGNFFSRAGLVSIITMPTTGTQNTDG.....YVNWDIDLMGYAQLRRKCELFTYMRFDAEFTFVVAKPNG.....
QYC65062.1    TQETAIGNFFSRAGLVSIITMPTTGTQNTDG.....YVNWDIDLMGYAQLRRKCELFTYMRFDAEFTFVVAKPNG.....
QYC65061.1    TQETAIGNFFSRAGLVSIITMPTTGTQNTDG.....YVNWDIDLMGYAQLRRKCELFTYMRFDAEFTFVVAKPNG.....
QYC65063.1    TQETAIGNFFSRAGLVSIITMPTTGTQNTDG.....YVNWDIDLMGYAQLRRKCELFTYMRFDAEFTFVVAKPNG.....
QYC65053.1    TQETAIGNFFSRAGLVSIITMPTTGTQNTDG.....YVNWDIDLMGYAQLRRKCELFTYMRFDAEFTFVVAKPNG.....
QYC65069.1    TQETAIGNFFSRAGLVSIITMPTTGTQNTDG.....YVNWDIDLMGYAQLRRKCELFTYMRFDAEFTFVVAKPNG.....
QYC65068.1    TQETAIGNFFSRAGLVSIITMPTTGTQNTDG.....YVNWDIDLMGYAQLRRKCELFTYMRFDAEFTFVVAKPNG.....
QYC65071.1    TQETAIGNFFSRAGLVSIITMPTTGTQNTDG.....YVNWDIDLMGYAQLRRKCELFTYMRFDAEFTFVVAKPNG.....
QYC65070.1    TQETAIGNFFSRAGLVSIITMPTTGTQNTDG.....YVNWDIDLMGYAQLRRKCELFTYMRFDAEFTFVVAKPNG.....
QYC65074.1    TQETAIGNFFSRAGLVSIITMPTTGTQNTDG.....YVNWDIDLMGYAQLRRKCELFTYMRFDAEFTFVVAKPNG.....
QYC65066.1    TQETAIGNFFSRAGLVSIITMPTTGTQNTDG.....YVNWDIDLMGYAQLRRKCELFTYMRFDAEFTFVVAKPNG.....
QYC65079.1    TQETAIGNFFSRAGLVSIITMPTTGTQNTDG.....YVNWDIDLMGYAQLRRKCELFTYMRFDAEFTFVVAKPNG.....
QYC65073.1    TQETAIGNFFSRAGLVSIITMPTTGTQNTDG.....YVNWDIDLMGYAQLRRKCELFTYMRFDAEFTFVVAKPNG.....
QYC65081.1    TQETAIGNFFSRAGLVSIITMPTTGTQNTDG.....YVNWDIDLMGYAQLRRKCELFTYMRFDAEFTFVVAKPNG.....
QYC65078.1    TQETAIGNFFSRAGLVSIITMPTTGTQNTDG.....YVNWDIDLMGYAQLRRKCELFTYMRFDAEFTFVVAKPNG.....
QYC65086.1    TQETAIGNFFSRAGLVSIITMPTTGTQNTDG.....YVNWDIDLMGYAQLRRKCELFTYMRFDAEFTFVVAKPNG.....
QYC65085.1    TQETAIGNFFSRAGLVSIITMPTTGTQNTDG.....YVNWDIDLMGYAQLRRKCELFTYMRFDAEFTFVVAKPNG.....
QYC65088.1    TQETAIGNFFSRAGLVSIITMPTTGTQNTDG.....YVNWDIDLMGYAQLRRKCELFTYMRFDAEFTFVVAKPNG.....
QYC65087.1    TQETAIGNFFSRAGLVSIITMPTTGTQNTDG.....YVNWDIDLMGYAQLRRKCELFTYMRFDAEFTFVVAKPNG.....
QYC65101.1    TQETAIGNFFSRAGLVSIITMPTTGTQNTDG.....YVNWDIDLMGYAQLRRKCELFTYMRFDAEFTFVVAKPNG.....
QYC65093.1    TQETAIGNFFSRAGLVSIITMPTTGTQNTDG.....YVNWDIDLMGYAQLRRKCELFTYMRFDAEFTFVVAKPNG.....
QYC65107.1    TQETAIGNFFSRAGLVSIITMPTTGTQNTDG.....YVNWDIDLMGYAQLRRKCELFTYMRFDAEFTFVVAKPNG.....
QYC65104.1    TQETAIGNFFSRAGLVSIITMPTTGTQNTDG.....YVNWDIDLMGYAQLRRKCELFTYMRFDAEFTFVVAKPNG.....
QYC65113.1    TQETAIGNFFSRAGLVSIITMPTTGTQNTDG.....YVNWDIDLMGYAQLRRKCELFTYMRFDAEFTFVVAKPNG.....
QYC65106.1    TQETAIGNFFSRAGLVSIITMPTTGTQNTDG.....YVNWDIDLMGYAQLRRKCELFTYMRFDAEFTFVVAKPNG.....
QYC65120.1    TQETAIGNFFSRAGLVSIITMPTTGTQNTDG.....YVNWDIDLMGYAQLRRKCELFTYMRFDAEFTFVVAKPNG.....
QYC65118.1    TQETAIGNFFSRAGLVSIITMPTTGTQNTDG.....YVNWDIDLMGYAQLRRKCELFTYMRFDAEFTFVVAKPNG.....
QYC65121.1    TQETAIGNFFSRAGLVSIITMPTTGTQNTDG.....YVNWDIDLMGYAQLRRKCELFTYMRFDAEFTFVVAKPNG.....
QYC65111.1    TQETAIGNFFSRAGLVSIITMPTTGTQNTDG.....YVNWDIDLMGYAQLRRKCELFTYMRFDAEFTFVVAKPNG.....
QYC65125.1    TQETAIGNFFSRAGLVSIITMPTTGTQNTDG.....YVNWDIDLMGYAQLRRKCELFTYMRFDAEFTFVVAKPNG.....
QYC65117.1    TQETAIGNFFSRAGLVSIITMPTTGTQNTDG.....YVNWDIDLMGYAQLRRKCELFTYMRFDAEFTFVVAKPNG.....
QYC65135.1    TQETAIGNFFSRAGLVSIITMPTTGTQNTDG.....YVNWDIDLMGYAQLRRKCELFTYMRFDAEFTFVVAKPNG.....
QYC65134.1    TQETAIGNFFSRAGLVSIITMPTTGTQNTDG.....YVNWDIDLMGYAQLRRKCELFTYMRFDAEFTFVVAKPNG.....
QYC65151.1    TQETAIGNFFSRAGLVSIITMPTTGTQNTDG.....YVNWDIDLMGYAQLRRKCELFTYMRFDAEFTFVVAKPNG.....
QYC65150.1    TQETAIGNFFSRAGLVSIITMPTTGTQNTDG.....YVNWDIDLMGYAQLRRKCELFTYMRFDAEFTFVVAKPNG.....
QYC65153.1    TQETAIGNFFSRAGLVSIITMPTTGTQNTDG.....YVNWDIDLMGYAQLRRKCELFTYMRFDAEFTFVVAKPNG.....
QYC65152.1    TQETAIGNFFSRAGLVSIITMPTTGTQNTDG.....YVNWDIDLMGYAQLRRKCELFTYMRFDAEFTFVVAKPNG.....
QYC65155.1    TQETAIGNFFSRAGLVSIITMPTTGTQNTDG.....YVNWDIDLMGYAQLRRKCELFTYMRFDAEFTFVVAKPNG.....
QYC65154.1    TQETAIGNFFSRAGLVSIITMPTTGTQNTDG.....YVNWDIDLMGYAQLRRKCELFTYMRFDAEFTFVVAKPNG.....
QYC65156.1    TQETAIGNFFSRAGLVSIITMPTTGTQNTDG.....YVNWDIDLMGYAQLRRKCELFTYMRFDAEFTFVVAKPNG.....
QYC65142.1    TQETAIGNFFSRAGLVSIITMPTTGTQNTDG.....YVNWDIDLMGYAQLRRKCELFTYMRFDAEFTFVVAKPNG.....
QYC65161.1    TQETAIGNFFSRAGLVSIITMPTTGTQNTDG.....YVNWDIDLMGYAQLRRKCELFTYMRFDAEFTFVVAKPNG.....
QYC65160.1    TQETAIGNFFSRAGLVSIITMPTTGTQNTDG.....YVNWDIDLMGYAQLRRKCELFTYMRFDAEFTFVVAKPNG.....
QYC65165.1    TQETAIGNFFSRAGLVSIITMPTTGTQNTDG.....YVNWDIDLMGYAQLRRKCELFTYMRFDAEFTFVVAKPNG.....
QYC65164.1    TQETAIGNFFSRAGLVSIITMPTTGTQNTDG.....YVNWDIDLMGYAQLRRKCELFTYMRFDAEFTFVVAKPNG.....
QYC65167.1    TQETAIGNFFSRAGLVSIITMPTTGTQNTDG.....YVNWDIDLMGYAQLRRKCELFTYMRFDAEFTFVVAKPNG.....
QYC65166.1    TQETAIGNFFSRAGLVSIITMPTTGTQNTDG.....YVNWDIDLMGYAQLRRKCELFTYMRFDAEFTFVVAKPNG.....
BCB92151.1    TQETAIGNFFSRAGLVSIITMPTTGTQNTDG.....YVNWDIDLMGYAQLRRKCELFTYMRFDAEFTFVVAKPNG.....
BBU37462.1    TQETAIGNFFSRAGLVSIITMPTTGTQNTDG.....YVNWDIDLMGYAQLRRKCELFTYMRFDAEFTFVVAKPNG.....
BBU37461.1    TQETAIGNFFSRAGLVSIITMPTTGTQNTDG.....YVNWDIDLMGYAQLRRKCELFTYMRFDAEFTFVVAKPNG.....
BBU37459.1    TQETAIGNFFSRAGLVSIITMPTTGTQNTDG.....YVNWDIDLMGYAQLRRKCELFTYMRFDAEFTFVVAKPNG.....
BBU37458.1    TQETAIGNFFSRAGLVSIITMPTTGTQNTDG.....YVNWDIDLMGYAQLRRKCELFTYMRFDAEFTFVVAKPNG.....
BBU37457.1    TQETAIGNFFSRAGLVSIITMPTTGTQNTDG.....YVNWDIDLMGYAQLRRKCELFTYMRFDAEFTFVVAKPNG.....
QWF08838.1    TQETAIGNFFSRAGLVSIITMPTTGTQNTDG.....YVNWDIDLMGYAQLRRKCELFTYMRFDAEFTFVVAKPNG.....
QWF08828.1    TQETAIGNFFSRAGLVSIITMPTTGTQNTDG.....YVNWDIDLMGYAQLRRKCELFTYMRFDAEFTFVVAKPNG.....
BCG66989.1    TQETAIGNFFSRAGLVSIITMPTTGTQNTDG.....YVNWDIDLMGYAQLRRKCELFTYMRFDAEFTFVVAKPNG.....
BCG66987.1    TQETAIGNFFSRAGLVSIITMPTTGTQNTDG.....YVNWDIDLMGYAQLRRKCELFTYMRFDAEFTFVVAKPNG.....
BCB92159.1    TQETAIGNFFSRAGLVSIITMPTTGTQNTDG.....YVNWDIDLMGYAQLRRKCELFTYMRFDAEFTFVVAKPNG.....
BBU37456.1    TQETAIGNFFSRAGLVSIITMPTTGTQNTDG.....YVNWDIDLMGYAQLRRKCELFTYMRFDAEFTFVVAKPNG.....
QWY15683.1    TQETAIGNFFSRAGLVSIITMPTTGTQNTDG.....YVNWDIDLMGYAQLRRKCELFTYMRFDAEFTFVVAKPNG.....
QWY15682.1    TQETAIGNFFSRAGLVSIITMPTTGTQNTDG.....YVNWDIDLMGYAQLRRKCELFTYMRFDAEFTFVVAKPNG.....
QWY15681.1    TQETAIGNFFSRAGLVSIITMPTTGTQNTDG.....YVNWDIDLMGYAQLRRKCELFTYMRFDAEFTFVVAKPNG.....
QWY15680.1    TQETAIGNFFSRAGLVSIITMPTTGTQNTDG.....YVNWDIDLMGYAQLRRKCELFTYMRFDAEFTFVVAKPNG.....
```

```
QWY15692.1   TQETAIGNFFSRAGLVSIITMPTTGTQNTDG.....YVNWDIDLMGYAQLRRKCELFTYMRFDAEFTFVVAKPNG.....
QWY15671.1   TQETAIGNFFSRAGLVSIITMPTTGTQNTDG.....YVNWDIDLMGYAQLRRKCELFTYMRFDAEFTFVVAKPNG.....
QWY15691.1   TQETAIGNFFSRAGLVSIITMPTTGTQNTDG.....YVNWDIDLMGYAQLRRKCELFTYMRFDAEFTFVVAKPNG.....
QWY15690.1   TQETAIGNFFSRAGLVSIITMPTTGTQNTDG.....YVNWDIDLMGYAQLRRKCELFTYMRFDAEFTFVVAKPNG.....
QWY15696.1   TQETAIGNFFSRAGLVSIITMPTTGTQNTDG.....YVNWDIDLMGYAQLRRKCELFTYMRFDAEFTFVVAKPNG.....
QWY15695.1   TQETAIGNFFSRAGLVSIITMPTTGTQNTDG.....YVNWDIDLMGYAQLRRKCELFTYMRFDAEFTFVVAKPNG.....
QWY15694.1   TQETAIGNFFSRAGLVSIITMPTTGTQNTDG.....YVNWDIDLMGYAQLRRKCELFTYMRFDAEFTFVVAKPNG.....
QWY15678.1   TQETAIGNFFSRAGLVSIITMPTTGTQNTDG.....YVNWDIDLMGYAQLRRKCELFTYMRFDAEFTFVVAKPNG.....
QWY15703.1   TQETAIGNFFSRAGLVSIITMPTTGTQNTDG.....YVNWDIDLMGYAQLRRKCELFTYMRFDAEFTFVVAKPNG.....
QWY15701.1   TQETAIGNFFSRAGLVSIITMPTTGTQNTDG.....YVNWDIDLMGYAQLRRKCELFTYMRFDAEFTFVVAKPNG.....
QWY15699.1   TQETAIGNFFSRAGLVSIITMPTTGTQNTDG.....YVNWDIDLMGYAQLRRKCELFTYMRFDAEFTFVVAKPNG.....
QWY15673.1   TQETAIGNFFSRAGLVSIITMPTTGTQNTDG.....YVNWDIDLMGYAQLRRKCELFTYMRFDAEFTFVVAKPNG.....
QWY15715.1   TQETAIGNFFSRAGLVSIITMPTTGTQNTDG.....YVNWDIDLMGYAQLRRKCELFTYMRFDAEFTFVVAKPNG.....
QWY15714.1   TQETAIGNFFSRAGLVSIITMPTTGTQNTDG.....YVNWDIDLMGYAQLRRKCELFTYMRFDAEFTFVVAKPNG.....
QWY15706.1   TQETAIGNFFSRAGLVSIITMPTTGTQNTDG.....YVNWDIDLMGYAQLRRKCELFTYMRFDAEFTFVVAKPNG.....
QWY15705.1   TQETAIGNFFSRAGLVSIITMPTTGTQNTDG.....YVNWDIDLMGYAQLRRKCELFTYMRFDAEFTFVVAKPNG.....
QTA73914.1   TQETAIGNFFSRAGLVSIITMPTTGTQNTDG.....YVNWDIDLMGYAQLRRKCELFTYMRFDAEFTFVVAKPNG.....
QWY15948.1   TQETAIGNFFSRAGLVSIITMPTTGTQNTDG.....YVNWDIDLMGYAQLRRKCELFTYMRFDAEFTFVVAKPNG.....
QWF08837.1   TQETAIGNFFSRAGLVSIITMPTTGTQNTDG.....YVNWDIDLMGYAQLRRXCELFTYMRFDAEFTFVVAKPNG.....
QWF08804.1   TQETAIGNFFSRAGLVSIITMPTTGTQNTDG.....YVNWDIDLMGYAQLRRXCELFTYMRFDAEFTFVVAKPNG.....
QWF08805.1   TQETAIGNFFSRAGLVSIITMPTTGTQNTDG.....YVNWDIDLMGYAQLRRKCELFTYMRFDAEFTFVVAKPNG.....
QWF08840.1   TQETAIGNFFSRAGLVSIITMPTTGTQNTDG.....YVNWDIDLMGYAQLRRKCELFTYMRFDAEFTFVVAKPNG.....
QWY15792.1   TQETAIGNFFSRAGLVSIITMPTTGTQNTDG.....YVNWDIDLMGYAQLRRKCELFTYMRFDAEFTFVVAKPNG.....
QTA73903.1   TQETAIGNFFSRAGLVSIITMPTTGTQNTDG.....YVNWDIDLMGYAQLRRKCELFTYMRFDAEFTFVVAKPNG.....
QTA73902.1   TQETAIGNFFSRAGLVSIITMPTTGTQNTDG.....YVNWDIDLMGYAQLRRKCELFTYMRFDAEFTFVVAKPNG.....
QTA73896.1   TQETAIGNFFSRAGLVSIITMPTTGTQNTDG.....YVNWDIDLMGYAQLRRKCELFTYMRFDAEFTFVVAKPNG.....
QTA73894.1   TQETAIGNFFSRAGLVSIITMPTTGTQNTDG.....YVNWDIDLMGYAQLRRKCELFTYMRFDAEFTFVVAKPNG.....
QWY15940.1   TQETAIGNFFSRAGLVSIITMPTTGTQNTDG.....YVNWDIDLMGYAQLRRKCELFTYMRFDAEFTFVVAKPNG.....
QWY15928.1   TQETAIGNFFSRAGLVSIITMPTTGTQNTDG.....YVNWDIDLMGYAQLRRKCELFTYMRFDAEFTFVVAKPNG.....
QWY15894.1   TQETAIGNFFSRAGLVSIITMPTTGTQNTDG.....YVNWDIDLMGYAQLRRKCELFTYMRFDAEFTFVVAKPNG.....
BCG67001.1   TQETAIGNFFSRAGLVSIITMPTTGTQNTDG.....YVNWDIDLMGYAQLRRKCELFTYMRFDAEFTFVVAKPNG.....
QWY15872.1   TQETAIGNFFSRAGLVSIITMPTTGTQNTDG.....YVNWDIDLMGYAQLRRKCELFTYMRFDAEFTFVVAKPNG.....
QWY15871.1   TQETAIGNFFSRAGLVSIITMPTTGTQNTDG.....YVNWDIDLMGYAQLRRKCELFTYMRFDAEFTFVVAKPNG.....
QWY15870.1   TQETAIGNFFSRAGLVSIITMPTTGTQNTDG.....YVNWDIDLMGYAQLRRKCELFTYMRFDAEFTFVVAKPNG.....
QWY15869.1   TQETAIGNFFSRAGLVSIITMPTTGTQNTDG.....YVNWDIDLMGYAQLRRKCELFTYMRFDAEFTFVVAKPNG.....
QWY15866.1   TQETAIGNFFSRAGLVSIITMPTTGTQNTDG.....YVNWDIDLMGYAQLRRKCELFTYMRFDAEFTFVVAKPNG.....
BCB92155.1   TQETAIGNFFSRAGLVSIITMPTTGTQNTDG.....YVNWDIDLMGYAQLRRKCELFTYMRFDAEFTFVVAKPNG.....
QWY15862.1   TQETAIGNFFSRAGLVSIITMPTTGTQNTDG.....YVNWDIDLMGYAQLRRKCELFTYMRFDAEFTFVVAKPNG.....
QWY15861.1   TQETAIGNFFSRAGLVSIITMPTTGTQNTDG.....YVNWDIDLMGYAQLRRKCELFTYMRFDAEFTFVVAKPNG.....
QWY15852.1   TQETAIGNFFSRAGLVSIITMPTTGTQNTDG.....YVNWDIDLMGYAQLRRKCELFTYMRFDAEFTFVVAKPNG.....
QWY15828.1   TQETAIGNFFSRAGLVSIITMPTTGTQNTDG.....YVNWDIDLMGYAQLRRKCELFTYMRFDAEFTFVVAKPNG.....
QWY15839.1   TQETAIGNFFSRAGLVSIITMPTTGTQNTDG.....YVNWDIDLMGYAQLRRKCELFTYMRFDAEFTFVVAKPNG.....
QWY15834.1   TQETAIGNFFSRAGLVSIITMPTTGTQNTDG.....YVNWDIDLMGYAQLRRKCELFTYMRFDAEFTFVVAKPNG.....
QWY15810.1   TQETAIGNFFSRAGLVSIITMPTTGTQNTDG.....YVNWDIDLMGYAQLRRKCELFTYMRFDAEFTFVVAKPNG.....
QWY15809.1   TQETAIGNFFSRAGLVSIITMPTTGTQNTDG.....YVNWDIDLMGYAQLRRKCELFTYMRFDAEFTFVVAKPNG.....
QWY15807.1   TQETAIGNFFSRAGLVSIITMPTTGTQNTDG.....YVNWDIDLMGYAQLRRKCELFTYMRFDAEFTFVVAKPNG.....
QWY15806.1   TQETAIGNFFSRAGLVSIITMPTTGTQNTDG.....YVNWDIDLMGYAQLRRKCELFTYMRFDAEFTFVVAKPNG.....
QWY15805.1   TQETAIGNFFSRAGLVSIITMPTTGTQNTDG.....YVNWDIDLMGYAQLRRKCELFTYMRFDAEFTFVVAKPNG.....
QWY15803.1   TQETAIGNFFSRAGLVSIITMPTTGTQNTDG.....YVNWDIDLMGYAQLRRKCELFTYMRFDAEFTFVVAKPNG.....
QWY15793.1   TQETAIGNFFSRAGLVSIITMPTTGTQNTDG.....YVNWDIDLMGYAQLRRKCELFTYMRFDAEFTFVVAKPNG.....
QYC65185.1   TQETAIGNFFSRAGLVSIITMPTTGTQNTDG.....YVNWDIDLMGYAQLRRKCELFTYMRFDAEFTFVVAKPNG.....
QWY15754.1   TQETAIGNFFSRAGLVSIITMPTTGTQNTDG.....YVNWDIDLMGYAQLRRKCELFTYMRFDAEFTFVVAKPNG.....
QWY15736.1   TQETAIGNFFSRAGLVSIITMPTTGTQNTDG.....YVNWDIDLMGYAQLRRKCELFTYMRFDAEFTFVVAKPNG.....
QWY15753.1   TQETAIGNFFSRAGLVSIITMPTTGTQNTDG.....YVNWDIDLMGYAQLRRKCELFTYMRFDAEFTFVVAKPNG.....
QYC65174.1   TQETAIGNFFSRAGLVSIITMPTTGTQNTDG.....YVNWDIDLMGYAQLRRKCELFTYMRFDAEFTFVVAKPNG.....
QWY15730.1   TQETAIGNFFSRAGLVSIITMPTTGTQNTDG.....YVNWDIDLMGYAQLRRKCELFTYMRFDAEFTFVVAKPNG.....
QWY15729.1   TQETAIGNFFSRAGLVSIITMPTTGTQNTDG.....YVNWDIDLMGYAQLRRKCELFTYMRFDAEFTFVVAKPNG.....
QWY15727.1   TQETAIGNFFSRAGLVSIITMPTTGTQNTDG.....YVNWDIDLMGYAQLRRKCELFTYMRFDAEFTFVVAKPNG.....
QWY15726.1   TQETAIGNFFSRAGLVSIITMPTTGTQNTDG.....YVNWDIDLMGYAQLRRKCELFTYMRFDAEFTFVVAKPNG.....
QWY15725.1   TQETAIGNFFSRAGLVSIITMPTTGTQNTDG.....YVNWDIDLMGYAQLRRKCELFTYMRFDAEFTFVVAKPNG.....
QWY15724.1   TQETAIGNFFSRAGLVSIITMPTTGTQNTDG.....YVNWDIDLMGYAQLRRKCELFTYMRFDAEFTFVVAKPNG.....
QWY15723.1   TQETAIGNFFSRAGLVSIITMPTTGTQNTDG.....YVNWDIDLMGYAQLRRKCELFTYMRFDAEFTFVVAKPNG.....
QWY15721.1   TQETAIGNFFSRAGLVSIITMPTTGTQNTDG.....YVNWDIDLMGYAQLRRKCELFTYMRFDAEFTFVVAKPNG.....
QWY15746.1   TQETAIGNFFSRAGLVSIITMPTTGTQNTDG.....YVNWDIDLMGYAQLRRKCELFTYMRFDAEFTFVVAKPNG.....
QWY15745.1   TQETAIGNFFSRAGLVSIITMPTTGTQNTDG.....YVNWDIDLMGYAQLRRKCELFTYMRFDAEFTFVVAKPNG.....
QWY15733.1   TQETAIGNFFSRAGLVSIITMPTTGTQNTDG.....YVNWDIDLMGYAQLRRKCELFTYMRFDAEFTFVVAKPNG.....
QYC65131.1   TQETAIGNFFSRAGLVSIITMPTTGTQNTDG.....YVNWDIDLMGYAQLRRKCELFTYMRFDAEFTFVVAKPNG.....
QWY15813.1   TQETAIGNFFSRAGLVSIITMPTTGTQNTDG.....YVNWDIDLMGYAQLRRKCELFTYMRFDAEFTFVVAKPNG.....
QWY15811.1   TQETAIGNFFSRAGLVSIITMPTTGTQNTDG.....YVNWDIDLMGYAQLRRKCELFTYMRFDAEFTFVVAKPNG.....
QTA73907.1   TQETAIGNFFSRAGLVSIITMPTTGTQNTDG.....YVNWDIDLMGYAQLRRKCELFTYMRFDAEFTFVVAKPNG.....
QTA73906.1   TQETAIGNFFSRAGLVSIITMPTTGTQNTDG.....YVNWDIDLMGYAQLRRKCELFTYMRFDAEFTFVVAKPNG.....
QOP74940.1   TQETAIGNFFSRAGLVSIITMPTTGTQNTDG.....YVNWDIDLMGYAQLRRKCELFTYMRFDAEFTFVVAKPNG.....
QOP74938.1   TQETAIGNFFSRAGLVSIITMPTTGTQNTDG.....YVNWDIDLMGYAQLRRKCELFTYMRFDAEFTFVVAKPNG.....
QOP74937.1   TQETAIGNFFSRAGLVSIITMPTTGTQNTDG.....YVNWDIDLMGYAQLRRKCELFTYMRFDAEFTFVVAKPNG.....
QOP74934.1   TQETAIGNFFSRAGLVSIITMPTTGTQNTDG.....YVNWDIDLMGYAQLRRKCELFTYMRFDAEFTFVVAKPNG.....
QOP74929.1   TQETAIGNFFSRAGLVSIITMPTTGTQNTDG.....YVNWDIDLMGYAQLRRKCELFTYMRFDAEFTFVVAKPNG.....
QOP74924.1   TQETAIGNFFSRAGLVSIITMPTTGTQNTDG.....YVNWDIDLMGYAQLRRKCELFTYMRFDAEFTFVVAKPNG.....
QOP74921.1   TQETAIGNFFSRAGLVSIITMPTTGTQNTDG.....YVNWDIDLMGYAQLRRKCELFTYMRFDAEFTFVVAKPNG.....
QOP74932.1   TQETAIGNFFSRAGLVSIITMPTTGTQNTDG.....YVNWDIDLMGYAQLRRKCELFTYMRFDAEFTFVVAKPNG.....
QOP74931.1   TQETAIGNFFSRAGLVSIITMPTTGTQNTDG.....YVNWDIDLMGYAQLRRKCELFTYMRFDAEFTFVVAKPNG.....
QIL87531.1   TQETAIGNFFSRAGLVSIITMPTTGTQNTDG.....YVNWDIDLMGYAQLRRKCELFTYMRFDAEFTFVVAKPNG.....
QIL87521.1   TQETAIGNFFSRAGLVSIITMPTTGTQNTDG.....YVNWDIDLMGYAQLRRKCELFTYMRFDAEFTFVVAKPNG.....
QIL87523.1   TQETAIGNFFSRAGLVSIITMPTTGTQNTDG.....YVNWDIDLMGYAQLRRKCELFTYMRFDAEFTFVVAKPNG.....
QIL87517.1   TQETAIGNFFSRAGLVSIITMPTTGTQNTDG.....YVNWDIDLMGYAQLRRKCELFTYMRFDAEFTFVVAKPNG.....
QIL87540.1   TQETAIGNFFSRAGLVSIITMPTTGTQNTDG.....YVNWDIDLMGYAQLRRKCELFTYMRFDAEFTFVVAKPNG.....
QIL87539.1   TQETAIGNFFSRAGLVSIITMPTTGTQNTDG.....YVNWDIDLMGYAQLRRKCELFTYMRFDAEFTFVVAKPNG.....
QIH53921.1   TQETAIGNFFSRAGLVSIITMPTTGTQNTDG.....YVNWDIDLMGYAQLRRKCELFTYMRFDAEFTFVVAKPNG.....
QIH53916.1   TQETAIGNFFSRAGLVSIITMPTTGTQNTDG.....YVNWDIDLMGYAQLRRKCELFTYMRFDAEFTFVVAKPNG.....
BBH72194.1   TQETAIGNFFSRAGLVSIITMPTTGTQNTDG.....YVNWDIDLMGYAQLRRKCELFTYMRFDAEFTFVVAKPNG.....
BBH72183.1   TQETAIGNFFSRAGLVSIITMPTTGTQNTDG.....YVNWDIDLMGYAQLRRKCELFTYMRFDAEFTFVVAKPNG.....
BBH72107.1   TQETAIGNFFSRAGLVSIITMPTTGTQNTDG.....YVNWDIDLMGYAQLRRKCELFTYMRFDAEFTFVVAKPNG.....
QWF08825.1   TQETAIGNFFSRAGLVSIITMPTTGTQNTDG.....YVNWDIDLMGYAQLRRKCELFTYMRFDAEFTFVVAKPNG.....
QWF08823.1   TQETAIGNFFSRAGLVSIITMPTTGTQNTDG.....YVNWDIDLMGYAQLRRKCELFTYMRFDAEFTFVVAKPNG.....
QWF08821.1   TQETAIGNFFSRAGLVSIITMPTTGTQNTDG.....YVNWDIDLMGYAQLRRKCELFTYMRFDAEFTFVVAKPNG.....
QWF08818.1   TQETAIGNFFSRAGLVSIITMPTTGTQNTDG.....YVNWDIDLMGYAQLRRKCELFTYMRFDAEFTFVVAKPNG.....
QWF08815.1   TQETAIGNFFSRAGLVSIITMPTTGTQNTDG.....YVNWDIDLMGYAQLRRKCELFTYMRFDAEFTFVVAKPNG.....
QWF08813.1   TQETAIGNFFSRAGLVSIITMPTTGTQNTDG.....YVNWDIDLMGYAQLRRKCELFTYMRFDAEFTFVVAKPNG.....
QWF08812.1   TQETAIGNFFSRAGLVSIITMPTTGTQNTDG.....YVNWDIDLMGYAQLRRKCELFTYMRFDAEFTFVVAKPNG.....
QWF08810.1   TQETAIGNFFSRAGLVSIITMPTTGTQNTDG.....YVNWDIDLMGYAQLRRKCELFTYMRFDAEFTFVVAKPNG.....
QWF08809.1   TQETAIGNFFSRAGLVSIITMPTTGTQNTDG.....YVNWDIDLMGYAQLRRKCELFTYMRFDAEFTFVVAKPNG.....
QWF08808.1   TQETAIGNFFSRAGLVSIITMPTTGTQNTDG.....YVNWDIDLMGYAQLRRKCELFTYMRFDAEFTFVVAKPNG.....
QWF08807.1   TQETAIGNFFSRAGLVSIITMPTTGTQNTDG.....YVNWDIDLMGYAQLRRKCELFTYMRFDAEFTFVVAKPNG.....
QWF08797.1   TQETAIGNFFSRAGLVSIITMPTTGTQNTDG.....YVNWDIDLMGYAQLRRKCELFTYMRFDAEFTFVVAKPNG.....
QWF08841.1   TQETAIGNFFSRAGLVSIITMPTTGTQNTDG.....YVNWDIDLMGYAQLRRKCELFTYMRFDAEFTFVVAKPNG.....
QWF08830.1   TQETAIGNFFSRAGLVSIITMPTTGTQNTDG.....YVNWDIDLMGYAQLRRKCELFTYMRFDAEFTFVVAKPNG.....
QTA73892.1   TQETAIGNFFSRAGLVSIITMPTTGTQNTDG.....YVNWDIDLMGYAQLRRKCELFTYMRFDAEFTFVVAKPNG.....
QTA73901.1   TQETAIGNFFSRAGLVSIITMPTTGTQNTDG.....YVNWDIDLMGYAQLRRKCELFTYMRFDAEFTFVVAKPNG.....
QWY15957.1   TQETAIGNFFSRAGLVSIITMPTTGTQNTDG.....YVNWDIDLMGYAQLRRKCELFTYMRFDAEFTFVVAKPNG.....
UEP53406.1   TQETAIGNFFSRAGLVSIITMPTTGTQNTDG.....YVNWDIDLMGYAQLRRKCELFTYMRFDAEFTFVVAKPNG.....
UEC49858.1   TQETAIGNFFSRAGLVSIITMPTTGTQNTDG.....YVNWDIDLMGYAQLRRKCELFTYMRFDAEFTFVVAKPNG.....
QWY15931.1   TQETAIGNFFSRAGLVSIITMPTTGTQNTDG.....YVNWDIDLMGYAQLRRKCELFTYMRFDAEFTFVVAKPNG.....
QWY15929.1   TQETAIGNFFSRAGLVSIITMPTTGTQNTDG.....YVNWDIDLMGYAQLRRKCELFTYMRFDAEFTFVVAKPNG.....
QWY15880.1   TQETAIGNFFSRAGLVSIITMPTTGTQNTDG.....YVNWDIDLMGYAQLRRKCELFTYMRFDAEFTFVVAKPNG.....
QWY15868.1   TQETAIGNFFSRAGLVSIITMPTTGTQNTDG.....YVNWDIDLMGYAQLRRKCELFTYMRFDAEFTFVVAKPNG.....
BCI65368.1   TQETAIGNFFSRAGLVSIITMPTTGTQNTDG.....YVNWDIDLMGYAQLRRKCELFTYMRFDAEFTFVVAKPNG.....
BCR43097.1   TQETAIGNFFSRAGLVSIITMPTTGTQNTDG.....YVNWDIDLMGYAQLRRKCELFTYMRFDAEFTFVVAKPNG.....
BCG67047.1   TQETAIGNFFSRAGLVSIITMPTTGTQNTDG.....YVNWDIDLMGYAQLRRKCELFTYMRFDAEFTFVVAKPNG.....
BCG67046.1   TQETAIGNFFSRAGLVSIITMPTTGTQNTDG.....YVNWDIDLMGYAQLRRKCELFTYMRFDAEFTFVVAKPNG.....
BCG67041.1   TQETAIGNFFSRAGLVSIITMPTTGTQNTDG.....YVNWDIDLMGYAQLRRKCELFTYMRFDAEFTFVVAKPNG.....
BCG67040.1   TQETAIGNFFSRAGLVSIITMPTTGTQNTDG.....YVNWDIDLMGYAQLRRKCELFTYMRFDAEFTFVVAKPNG.....
BCG67039.1   TQETAIGNFFSRAGLVSIITMPTTGTQNTDG.....YVNWDIDLMGYAQLRRKCELFTYMRFDAEFTFVVAKPNG.....
BCG67038.1   TQETAIGNFFSRAGLVSIITMPTTGTQNTDG.....YVNWDIDLMGYAQLRRKCELFTYMRFDAEFTFVVAKPNG.....
BCG67032.1   TQETAIGNFFSRAGLVSIITMPTTGTQNTDG.....YVNWDIDLMGYAQLRRKCELFTYMRFDAEFTFVVAKPNG.....
BCG67030.1   TQETAIGNFFSRAGLVSIITMPTTGTQNTDG.....YVNWDIDLMGYAQLRRKCELFTYMRFDAEFTFVVAKPNG.....
BCG66995.1   TQETAIGNFFSRAGLVSIITMPTTGTQNTDG.....YVNWDIDLMGYAQLRRKCELFTYMRFDAEFTFVVAKPNG.....
BCG66994.1   TQETAIGNFFSRAGLVSIITMPTTGTQNTDG.....YVNWDIDLMGYAQLRRKCELFTYMRFDAEFTFVVAKPNG.....
QWY15757.1   TQETAIGNFFSRAGLVSIITMPTTGTQNTDG.....YVNWDIDLMGYAQLRRKCELFTYMRFDAEFTFVVAKPNG.....
QWY15756.1   TQETAIGNFFSRAGLVSIITMPTTGTQNTDG.....YVNWDIDLMGYAQLRRKCELFTYMRFDAEFTFVVAKPNG.....
QWY15748.1   TQETAIGNFFSRAGLVSIITMPTTGTQNTDG.....YVNWDIDLMGYAQLRRKCELFTYMRFDAEFTFVVAKPNG.....
QWY15747.1   TQETAIGNFFSRAGLVSIITMPTTGTQNTDG.....YVNWDIDLMGYAQLRRKCELFTYMRFDAEFTFVVAKPNG.....
QWY15720.1   TQETAIGNFFSRAGLVSIITMPTTGTQNTDG.....YVNWDIDLMGYAQLRRKCELFTYMRFDAEFTFVVAKPNG.....
QWY15717.1   TQETAIGNFFSRAGLVSIITMPTTGTQNTDG.....YVNWDIDLMGYAQLRRKCELFTYMRFDAEFTFVVAKPNG.....
QWY15697.1   TQETAIGNFFSRAGLVSIITMPTTGTQNTDG.....YVNWDIDLMGYAQLRRKCELFTYMRFDAEFTFVVAKPNG.....
QWY15689.1   TQETAIGNFFSRAGLVSIITMPTTGTQNTDG.....YVNWDIDLMGYAQLRRKCELFTYMRFDAEFTFVVAKPNG.....
QWY15761.1   TQETAIGNFFSRAGLVSIITMPTTGTQNTDG.....YVNWDIDLMGYAQLRRKCELFTYMRFDAEFTFVVAKPNG.....
QWY15743.1   TQETAIGNFFSRAGLVSIITMPTTGTQNTDG.....YVNWDIDLMGYAQLRRKCELFTYMRFDAEFTFVVAKPNG.....
QWY15759.1   TQETAIGNFFSRAGLVSIITMPTTGTQNTDG.....YVNWDIDLMGYAQLRRKCELFTYMRFDAEFTFVVAKPNG.....
QWY15758.1   TQETAIGNFFSRAGLVSIITMPTTGTQNTDG.....YVNWDIDLMGYAQLRRKCELFTYMRFDAEFTFVVAKPNG.....
QWY15677.1   TQETAIGNFFSRAGLVSIITMPTTGTQNTDG.....YVNWDIDLMGYAQLRRKCELFTYMRFDAEFTFVVAKPNG.....
QWY15675.1   TQETAIGNFFSRAGLVSIITMPTTGTQNTDG.....YVNWDIDLMGYAQLRRKCELFTYMRFDAEFTFVVAKPNG.....
QWY15776.1   TQETAIGNFFSRAGLVSIITMPTTGTQNTDG.....YVNWDIDLMGYAQLRRKCELFTYMRFDAEFTFVVAKPNG.....
QWY15772.1   TQETAIGNFFSRAGLVSIITMPTTGTQNTDG.....YVNWDIDLMGYAQLRRKCELFTYMRFDAEFTFVVAKPNG.....
QWY15770.1   TQETAIGNFFSRAGLVSIITMPTTGTQNTDG.....YVNWDIDLMGYAQLRRKCELFTYMRFDAEFTFVVAKPNG.....
QWY15762.1   TQETAIGNFFSRAGLVSIITMPTTGTQNTDG.....YVNWDIDLMGYAQLRRKCELFTYMRFDAEFTFVVAKPNG.....
```

```
QWY15732.1    TQETAIGNFFSRAGLVSIITMPTTGTQNTDG.....YVNWDIDLMGYAQLRRKCELFTYMRFDAEFTFVVAKPNG.....
QWY15731.1    TQETAIGNFFSRAGLVSIITMPTTGTQNTDG.....YVNWDIDLMGYAQLRRKCELFTYMRFDAEFTFVVAKPNG.....
QWY15780.1    TQETAIGNFFSRAGLVSIITMPTTGTQNTDG.....YVNWDIDLMGYAQLRRKCELFTYMRFDAEFTFVVAKPNG.....
QWY15779.1    TQETAIGNFFSRAGLVSIITMPTTGTQNTDG.....YVNWDIDLMGYAQLRRKCELFTYMRFDAEFTFVVAKPNG.....
BCG67006.1    TQETAIGNFFSRAGLVSIITMPTTGTQNTDG.....YVNWDIDLMGYAQLRRKCELFTYMRFDAEFTFVVAKPNG.....
BCG67004.1    TQETAIGNFFSRAGLVSIITMPTTGTQNTDG.....YVNWDIDLMGYAQLRRKCELFTYMRFDAEFTFVVAKPNG.....
BCG67019.1    TQETAIGNFFSRAGLVSIITMPTTGTQNTDG.....YVNWDIDLMGYAQLRRKCELFTYMRFDAEFTFVVAKPNG.....
BCG67002.1    TQETAIGNFFSRAGLVSIITMPTTGTQNTDG.....YVNWDIDLMGYAQLRRKCELFTYMRFDAEFTFVVAKPNG.....
BCG67036.1    TQETAIGNFFSRAGLVSIITMPTTGTQNTDG.....YVNWDIDLMGYAQLRRKCELFTYMRFDAEFTFVVAKPNG.....
BCG67035.1    TQETAIGNFFSRAGLVSIITMPTTGTQNTDG.....YVNWDIDLMGYAQLRRKCELFTYMRFDAEFTFVVAKPNG.....
QWY15785.1    TQETAIGNFFSRAGLVSIITMPTTGTQNTDG.....YVNWDIDLMGYAQLRRKCELFTYMRFDAEFTFVVAKPNG.....
QWY15784.1    TQETAIGNFFSRAGLVSIITMPTTGTQNTDG.....YVNWDIDLMGYAQLRRKCELFTYMRFDAEFTFVVAKPNG.....
QWY15789.1    TQETAIGNFFSRAGLVSIITMPTTGTQNTDG.....YVNWDIDLMGYAQLRRKCELFTYMRFDAEFTFVVAKPNG.....
QYC65159.1    TQETAIGNFFSRAGLVSIITMPTTGTQNTDG.....YVNWDIDLMGYAQLRRKCELFTYMRFDAEFTFVVAKPNG.....
BCR43101.1    TQETAIGNFFSRAGLVSIITMPTTGTQNTDG.....YVNWDIDLMGYAQLRRKCELFTYMRFDAEFTFVVAKPNG.....
BCR43098.1    TQETAIGNFFSRAGLVSIITMPTTGTQNTDG.....YVNWDIDLMGYAQLRRKCELFTYMRFDAEFTFVVAKPNG.....
QWY15873.1    TQETAIGNFFSRAGLVSIITMPTTGTQNTDG.....YVNWDIDLMGYAQLRRKCELFTYMRFDAEFTFVVAKPNG.....
QWY15865.1    TQETAIGNFFSRAGLVSIITMPTTGTQNTDG.....YVNWDIDLMGYAQLRRKCELFTYMRFDAEFTFVVAKPNG.....
QWY15900.1    TQETAIGNFFSRAGLVSIITMPTTGTQNTDG.....YVNWDIDLMGYAQLRRKCELFTYMRFDAEFTFVVAKPNG.....
BCG67049.1    TQETAIGNFFSRAGLVSIITMPTTGTQNTDG.....YVNWDIDLMGYAQLRRKCELFTYMRFDAEFTFVVAKPNG.....
UEC49853.1    TQETAIGNFFSRAGLVSIITMPTTGTQNTDG.....YVNWDIDLMGYAQLRRKCELFTYMRFDAEFTFVVAKPNG.....
UEC49848.1    TQETAIGNFFSRAGLVSIITMPTTGTQNTDG.....YVNWDIDLMGYAQLRRKCELFTYMRFDAEFTFVVAKPNG.....
QTA73899.1    TQETAIGNFFSRAGLVSIITMPTTGTQNTDG.....YVNWDIDLMGYAQLRRKCELFTYMRFDAEFTFVVAKPNG.....
QTA73898.1    TQETAIGNFFSRAGLVSIITMPTTGTQNTDG.....YVNWDIDLMGYAQLRRKCELFTYMRFDAEFTFVVAKPNG.....
QTA73905.1    TQETAIGNFFSRAGLVSIITMPTTGTQNTDG.....YVNWDIDLMGYAQLRRKCELFTYMRFDAEFTFVVAKPNG.....
QTA73904.1    TQETAIGNFFSRAGLVSIITMPTTGTQNTDG.....YVNWDIDLMGYAQLRRKCELFTYMRFDAEFTFVVAKPNG.....
QTA73909.1    TQETAIGNFFSRAGLVSIITMPTTGTQNTDG.....YVNWDIDLMGYAQLRRKCELFTYMRFDAEFTFVVAKPNG.....
QTA73908.1    TQETAIGNFFSRAGLVSIITMPTTGTQNTDG.....YVNWDIDLMGYAQLRRKCELFTYMRFDAEFTFVVAKPNG.....
UEP53408.1    TQETAIGNFFSRAGLVSIITMPTTGTQNTDG.....YVNWDIDLMGYAQLRRKCELFTYMRFDAEFTFVVAKPNG.....
QWF08839.1    TQETAIGNFFSRAGLVSIITMPTTGTQNTDG.....YVNWDIDLMGYAQLRRKCELFTYMRFDAEFTFVVAKPNG.....
QWF08799.1    TQETAIGNFFSRAGLVSIITMPTTGTQNTDG.....YVNWDIDLMGYAQLRRKCELFTYMRFDAEFTFVVAKPNG.....
QWY15771.1    TQETAIGNFFSRAGLVSIITMPTTGTQNTDG.....YVNWDIDLMGYAQLRRKCELFTYMRFDAEFTFVVAKPNG.....
QTA73891.1    TQETAIGNFFSRAGLVSIITMPTTGTQNTDG.....YVNWDIDLMGYAQLRRKCELFTYMRFDAEFTFVVAKPNG.....
QWY15670.1    TQETAIGNFFSRAGLVSIITMPTTGTQNTDG.....YVNWDIDLMGYAQLRRKCELFTYMRFDAEFTFVVAKPNG.....
BBH72149.1    TQETAIGNFFSRAGLVSIITMPTTGTQNTDG.....YVNWDIDLMGYAQLRRKCELFTYMRFDAEFTFVVAKPNG.....
BBH72143.1    TQETAIGNFFSRAGLVSIITMPTTGTQNTDG.....YVNWDIDLMGYAQLRRKCELFTYMRFDAEFTFVVAKPNG.....
BBH72163.1    TQETAIGNFFSRAGLVSIITMPTTGTQNTDG.....YVNWDIDLMGYAQLRRKCELFTYMRFDAEFTFVVAKPNG.....
QTA73895.1    TQETAIGNFFSRAGLVSIITMPTTGTQNTDG.....YVNWDIDLMGYAQLRRKCELFTYMRFDAEFTFVVAKPNG.....
QWY15674.1    TQETAIGNFFSRAGLVSIITMPTTGTQNTDG.....YVNWDIDLMGYAQLRRKCELFTYMRFDAEFTFVVAKPNG.....
QWY15710.1    TQETAIGNFFSRAGLVSIITMPTTGTQNTDG.....YVNWDIDLMGYAQLRRKCELFTYMRFDAEFTFVVAKPNG.....
QWY15709.1    TQETAIGNFFSRAGLVSIITMPTTGTQNTDG.....YVNWDIDLMGYAQLRRKCELFTYMRFDAEFTFVVAKPNG.....
QWF08934.1    TQETAIGNFFSRAGLVSIITMPTTGTQNTDG.....YVNWDIDLMGYAQLRRKCELFTYMRFDAEFTFVVAKPNG.....
QWF08836.1    TQETAIGNFFSRAGLVSIITMPTTGTQNTDG.....YVNWDIDLMGYAQLRRKCELFTYMRFDAEFTFVVAKPNG.....
QLH02014.1    TQETAIGNFFSRAGLVSIITMPTTGTQNTDG.....YVNWDIDLMGYAQLRRKCELFTYMRFDAEFTFVVAKPNG.....
QWF08796.1    TQETAIGNFFSRAGLVSIITMPTTGTQNTDG.....YVNWDIDLMGYAQLRRKCELFTYMRFDAEFTFVVAKPNG.....
QYC65090.1    TQETAIGNFFSRAGLVSIITMPTTGTQNTDG.....YVNWDIDLMGYAQLRRKCELFTYMRFDAEFTFVVAKPNG.....
QTA73900.1    TQETAIGNFFSRAGLVSIITMPTTGTQNTDG.....YVNWDIDLMGYAQLRRKCELFTYMRFDAEFTFVVAKPNG.....
QWY15679.1    TQETAIGNFFSRAGLVSIITMPTTGTQNTDG.....YVNWDIDLMGYAQLRRKCELFTYMRFDAEFTFVVAKPNG.....
QWY15751.1    TQETAIGNFFSRAGLVSIITMPTTGTQNTDG.....YVNWDIDLMGYAQLRRKCELFTYMRFDAEFTFVVAKPNG.....
QWF08795.1    TQETAIGNFFSRAGLVSIITMPTTGTQNTDG.....YVNWDIDLMGYAQLRRKCELFTYMRFDAEFTFVVAKPNG.....
QWF08789.1    TQETAIGNFFSRAGLVSIITMPTTGTQNTDG.....YVNWDIDLMGYAQLRRKCELFTYMRFDAEFTFVVAKPNG.....
QYC65158.1    TQETAIGNFFSRAGLVSIITMPTTGTQNTDG.....YVNWDIDLMGYAQLRRKCELFTYMRFDAEFTFVVAKPNG.....
QYC65109.1    TQETAIGNFFSRAGLVSIITMPTTGTQNTDG.....YVNWDIDLMGYAQLRRKCELFTYMRFDAEFTFVVAKPNG.....
QWF08883.1    TQETAIGNFFSRAGLVSIITMPTTGTQNTDG.....YVNWDIDLMGYAQLRRKCELFTYMRFDAEFTFVVAKPNG.....
QWF08866.1    TQETAIGNFFSRAGLVSIITMPTTGTQNTDG.....YVNWDIDLMGYAQLRRKCELFTYMRFDAEFTFVVAKPTG.....
QWF08833.1    TQETAIGNFFSRAGLVSIITMPTTGTQNTDG.....YVNWDIDLMGYAQLRRKCELFTYMRFDAEFTFVVAKPTG.....
QWF08801.1    TQETAIGNFFSRAGLVSIITMPTTGTQNTDG.....YVNWDIDLMGYAQLRRKCELFTYMRFDAEFTFVVAKPTG.....
QWF08842.1    TQETAIGNFFSRAGLVSIITMPTTGTQNTDG.....YVNWDIDLMGYAQLRRKCELFTYMRFDAEFTFVVTQPHG.....
QWF08806.1    TQETAIGNFFSRAGLVSIITMPTTGTQNTDG.....YVNWDIDLMGYAQLRRKCELFTYMRFDAEFTFVVTQPHG.....
QYC65065.1    TQETAIGNFFSRAGLVSIITMPTTGTQNTDG.....YVNWDIDLMGYAQLRRKCELFTYMRFDAEFTFVVAKPNG.....
QYC65044.1    TQETAIGNFFSRAGLVSIITMPTTGTQNTDG.....YVNWDIDLMGYAQLRRKCELFTYMRFDAEFTFVVAKPNG.....
QWY15863.1    TQETAIGNFFSRAGLVSIITMPTTGTQNTDG.....YVNWDIDLMGYAQLRRKCELFTYMRFDAEFTFVVAKPNG.....
QTA73916.1    TQETAIGNFFSRAGLVSIITMPTTGTQNTDG.....YVNWDIDLMGYAQLRRKCELFTYMRFDAEFTFVVAKPNG.....
BCG66990.1    TQETAIGNFFSRAGLVSIITMPTTGTQNTDG.....YVNWDIDLMGYAQLRRKCELFTYMRFDAEFTFVVAKPNG.....
QTA73915.1    TQETAIGNFFSRAGLVSIITMPTTGTQNTDG.....YVNWDIDLMGYAQLRRKCELFTYMRFDAEFTFVVAKPNG.....
QWY15932.1    TQETAIGNFFSRAGLVSIITMPTTGTQNTDG.....YVNWDIDLMGYAQLRRKCELFTYMRFDAEFTFVVAKPNG.....
QWY15867.1    TQETAIGNFFSRAGLVSIITMPTTGTQNTDG.....YVNWDIDLMGYAQLRRKCELFTYMRFDAEFTFVVAKPNG.....
QWY15859.1    TQETAIGNFFSRAGLVSIITMPTTGTQNTDG.....YVNWDIDLMGYAQLRRKCELFTYMRFDAEFTFVVAKPNG.....
QWY15858.1    TQETAIGNFFSRAGLVSIITMPTTGTQNTDG.....YVNWDIDLMGYAQLRRKCELFTYMRFDAEFTFVVAKPNG.....
QWY15782.1    TQETAIGNFFSRAGLVSIITMPTTGTQNTDG.....YVNWDIDLMGYAQLRRKCELFTYMRFDAEFTFVVAKPNG.....
QWY15781.1    TQETAIGNFFSRAGLVSIITMPTTGTQNTDG.....YVNWDIDLMGYAQLRRKCELFTYMRFDAEFTFVVAKPNG.....
BCI65355.1    TQETAIGNFFSRAGLVSIITMPTTGTQNTDG.....YVNWDIDLMGYAQLRRKCELFTYMRFDAEFTFVVAKPNG.....
BCI65374.1    TQETAIGNFFSRAGLVSIITMPTTGTQNTDG.....YVNWDIDLMGYAQLRRKCELFTYMRFDAEFTFVVAKPNG.....
BCI65373.1    TQETAIGNFFSRAGLVSIITMPTTGTQNTDG.....YVNWDIDLMGYAQLRRKCELFTYMRFDAEFTFVVAKPNG.....
BCI65372.1    TQETAIGNFFSRAGLVSIITMPTTGTQNTDG.....YVNWDIDLMGYAQLRRKCELFTYMRFDAEFTFVVAKPNG.....
BCI65371.1    TQETAIGNFFSRAGLVSIITMPTTGTQNTDG.....YVNWDIDLMGYAQLRRKCELFTYMRFDAEFTFVVAKPNG.....
BCI65369.1    TQETAIGNFFSRAGLVSIITMPTTGTQNTDG.....YVNWDIDLMGYAQLRRKCELFTYMRFDAEFTFVVAKPNG.....
BCI65367.1    TQETAIGNFFSRAGLVSIITMPTTGTQNTDG.....YVNWDIDLMGYAQLRRKCELFTYMRFDAEFTFVVAKPNG.....
BCI65365.1    TQETAIGNFFSRAGLVSIITMPTTGTQNTDG.....YVNWDIDLMGYAQLRRKCELFTYMRFDAEFTFVVAKPNG.....
BCI65364.1    TQETAIGNFFSRAGLVSIITMPTTGTQNTDG.....YVNWDIDLMGYAQLRRKCELFTYMRFDAEFTFVVAKPNG.....
BCI65363.1    TQETAIGNFFSRAGLVSIITMPTTGTQNTDG.....YVNWDIDLMGYAQLRRKCELFTYMRFDAEFTFVVAKPNG.....
BCI65362.1    TQETAIGNFFSRAGLVSIITMPTTGTQNTDG.....YVNWDIDLMGYAQLRRKCELFTYMRFDAEFTFVVAKPNG.....
BCI65361.1    TQETAIGNFFSRAGLVSIITMPTTGTQNTDG.....YVNWDIDLMGYAQLRRKCELFTYMRFDAEFTFVVAKPNG.....
BCI65360.1    TQETAIGNFFSRAGLVSIITMPTTGTQNTDG.....YVNWDIDLMGYAQLRRKCELFTYMRFDAEFTFVVAKPNG.....
BCI65359.1    TQETAIGNFFSRAGLVSIITMPTTGTQNTDG.....YVNWDIDLMGYAQLRRKCELFTYMRFDAEFTFVVAKPNG.....
BCI65358.1    TQETAIGNFFSRAGLVSIITMPTTGTQNTDG.....YVNWDIDLMGYAQLRRKCELFTYMRFDAEFTFVVAKPNG.....
BCI65356.1    TQETAIGNFFSRAGLVSIITMPTTGTQNTDG.....YVNWDIDLMGYAQLRRKCELFTYMRFDAEFTFVVAKPNG.....
BCI65354.1    TQETAIGNFFSRAGLVSIITMPTTGTQNTDG.....YVNWDIDLMGYAQLRRKCELFTYMRFDAEFTFVVAKPNG.....
BCI65353.1    TQETAIGNFFSRAGLVSIITMPTTGTQNTDG.....YVNWDIDLMGYAQLRRKCELFTYMRFDAEFTFVVAKPNG.....
BCI65352.1    TQETAIGNFFSRAGLVSIITMPTTGTQNTDG.....YVNWDIDLMGYAQLRRKCELFTYMRFDAEFTFVVAKPNG.....
BCI65351.1    TQETAIGNFFSRAGLVSIITMPTTGTQNTDG.....YVNWDIDLMGYAQLRRKCELFTYMRFDAEFTFVVAKPNG.....
BCI65350.1    TQETAIGNFFSRAGLVSIITMPTTGTQNTDG.....YVNWDIDLMGYAQLRRKCELFTYMRFDAEFTFVVAKPNG.....
BCI65349.1    TQETAIGNFFSRAGLVSIITMPTTGTQNTDG.....YVNWDIDLMGYAQLRRKCELFTYMRFDAEFTFVVAKPNG.....
BCI65348.1    TQETAIGNFFSRAGLVSIITMPTTGTQNTDG.....YVNWDIDLMGYAQLRRKCELFTYMRFDAEFTFVVAKPNG.....
BCI65347.1    TQETAIGNFFSRAGLVSIITMPTTGTQNTDG.....YVNWDIDLMGYAQLRRKCELFTYMRFDAEFTFVVAKPNG.....
BCI65346.1    TQETAIGNFFSRAGLVSIITMPTTGTQNTDG.....YVNWDIDLMGYAQLRRKCELFTYMRFDAEFTFVVAKPNG.....
BCI65345.1    TQETAIGNFFSRAGLVSIITMPTTGTQNTDG.....YVNWDIDLMGYAQLRRKCELFTYMRFDAEFTFVVAKPNG.....
BCI65344.1    TQETAIGNFFSRAGLVSIITMPTTGTQNTDG.....YVNWDIDLMGYAQLRRKCELFTYMRFDAEFTFVVAKPNG.....
BCI65343.1    TQETAIGNFFSRAGLVSIITMPTTGTQNTDG.....YVNWDIDLMGYAQLRRKCELFTYMRFDAEFTFVVAKPNG.....
BCI65342.1    TQETAIGNFFSRAGLVSIITMPTTGTQNTDG.....YVNWDIDLMGYAQLRRKCELFTYMRFDAEFTFVVAKPNG.....
BCI65341.1    TQETAIGNFFSRAGLVSIITMPTTGTQNTDG.....YVNWDIDLMGYAQLRRKCELFTYMRFDAEFTFVVAKPNG.....
BCI65340.1    TQETAIGNFFSRAGLVSIITMPTTGTQNTDG.....YVNWDIDLMGYAQLRRKCELFTYMRFDAEFTFVVAKPNG.....
BCI65339.1    TQETAIGNFFSRAGLVSIITMPTTGTQNTDG.....YVNWDIDLMGYAQLRRKCELFTYMRFDAEFTFVVAKPNG.....
BCI65338.1    TQETAIGNFFSRAGLVSIITMPTTGTQNTDG.....YVNWDIDLMGYAQLRRKCELFTYMRFDAEFTFVVAKPNG.....
BCI65337.1    TQETAIGNFFSRAGLVSIITMPTTGTQNTDG.....YVNWDIDLMGYAQLRRKCELFTYMRFDAEFTFVVAKPNG.....
BCI65336.1    TQETAIGNFFSRAGLVSIITMPTTGTQNTDG.....YVNWDIDLMGYAQLRRKCELFTYMRFDAEFTFVVAKPNG.....
BCI65335.1    TQETAIGNFFSRAGLVSIITMPTTGTQNTDG.....YVNWDIDLMGYAQLRRKCELFTYMRFDAEFTFVVAKPNG.....
BCI65334.1    TQETAIGNFFSRAGLVSIITMPTTGTQNTDG.....YVNWDIDLMGYAQLRRKCELFTYMRFDAEFTFVVAKPNG.....
BCI65333.1    TQETAIGNFFSRAGLVSIITMPTTGTQNTDG.....YVNWDIDLMGYAQLRRKCELFTYMRFDAEFTFVVAKPNG.....
BCI65332.1    TQETAIGNFFSRAGLVSIITMPTTGTQNTDG.....YVNWDIDLMGYAQLRRKCELFTYMRFDAEFTFVVAKPNG.....
BCI65331.1    TQETAIGNFFSRAGLVSIITMPTTGTQNTDG.....YVNWDIDLMGYAQLRRKCELFTYMRFDAEFTFVVAKPNG.....
BCI65330.1    TQETAIGNFFSRAGLVSIITMPTTGTQNTDG.....YVNWDIDLMGYAQLRRKCELFTYMRFDAEFTFVVAKPNG.....
BCI65328.1    TQETAIGNFFSRAGLVSIITMPTTGTQNTDG.....YVNWDIDLMGYAQLRRKCELFTYMRFDAEFTFVVAKPNG.....
BCI65327.1    TQETAIGNFFSRAGLVSIITMPTTGTQNTDG.....YVNWDIDLMGYAQLRRKCELFTYMRFDAEFTFVVAKPNG.....
BCI65326.1    TQETAIGNFFSRAGLVSIITMPTTGTQNTDG.....YVNWDIDLMGYAQLRRKCELFTYMRFDAEFTFVVAKPNG.....
BCI65325.1    TQETAIGNFFSRAGLVSIITMPTTGTQNTDG.....YVNWDIDLMGYAQLRRKCELFTYMRFDAEFTFVVAKPNG.....
BCI65324.1    TQETAIGNFFSRAGLVSIITMPTTGTQNTDG.....YVNWDIDLMGYAQLRRKCELFTYMRFDAEFTFVVAKPNG.....
BCI65323.1    TQETAIGNFFSRAGLVSIITMPTTGTQNTDG.....YVNWDIDLMGYAQLRRKCELFTYMRFDAEFTFVVAKPNG.....
BCI65322.1    TQETAIGNFFSRAGLVSIITMPTTGTQNTDG.....YVNWDIDLMGYAQLRRKCELFTYMRFDAEFTFVVAKPNG.....
BCI65321.1    TQETAIGNFFSRAGLVSIITMPTTGTQNTDG.....YVNWDIDLMGYAQLRRKCELFTYMRFDAEFTFVVAKPNG.....
BCI65320.1    TQETAIGNFFSRAGLVSIITMPTTGTQNTDG.....YVNWDIDLMGYAQLRRKCELFTYMRFDAEFTFVVAKPNG.....
BCI65319.1    TQETAIGNFFSRAGLVSIITMPTTGTQNTDG.....YVNWDIDLMGYAQLRRKCELFTYMRFDAEFTFVVAKPNG.....
BCI65318.1    TQETAIGNFFSRAGLVSIITMPTTGTQNTDG.....YVNWDIDLMGYAQLRRKCELFTYMRFDAEFTFVVAKPNG.....
BCI65317.1    TQETAIGNFFSRAGLVSIITMPTTGTQNTDG.....YVNWDIDLMGYAQLRRKCELFTYMRFDAEFTFVVAKPNG.....
QYC65119.1    TQETAIGNFFSRAGLVSIITMPTTGTQNTDG.....YVNWDIDLMGYAQLRRKCELFTYMRFDAEFTFVVAKPNG.....
QWF08905.1    TQETAIGNFFSRAGLVSIITMPTTGTQNTDG.....YVNWDIDLMGYAQLRRKCELFTYMRFDAEFTFVVAKPNG.....
QWF08814.1    TQETAIGNFFSRAGLVSIITMPTTGTQNTDG.....YVNWDIDLMGYAQLRRKCELFTYMRFDAEFTFVVAKPNG.....
QWF08776.1    TQETAIGNFFSRAGLVSIITMPTTGTQNTDG.....YVNWDIDLMGYAQLRRKCELFTYMRFDAEFTFVVAKPNG.....
BCI65370.1    TQETAIGNFFSRAGLVSIITMPTTGTQNTDG.....YVNWDIDLMGYAQLRRKCELFTYMRFDAEFTFVVAKPNG.....
BCG67048.1    TQETAIGNFFSRAGLVSIITMPTTGTQNTDG.....YVNWDIDLMGYAQLRRKCELFTYMRFDAEFTFVVAKPNG.....
QWY15933.1    TQETAIGNFFSRAGLVSIITMPTTGTQNTDG.....YVNWDIDLMGYAQLRRKCELFTYMRFDAEFTFVVAKPNG.....
UEP53404.1    TQETAIGNFFSRAGLVSIITMPTTGTQNTDG.....YVNWDIDLMGYAQLRRKCELFTYMRFDAEFTFVVAKPNG.....
UEC49857.1    TQETAIGNFFSRAGLVSIITMPTTGTQNTDG.....YVNWDIDLMGYAQLRRKCELFTYMRFDAEFTFVVAKPNG.....
QYC65076.1    TQETAIGNFFSRAGLVSIIAMPTTGTQNTDG.....YVNWDIDLMGYAQLRRKCELFTYMRFDAEFTFVVAKPNG.....
QWF08819.1    TQETAIGNFFSRAGLVSIIAMPTTGTQNTDG.....YVNWDIDLMGYAQLRRKCELFTYMRFDAEFTFVVAKPNG.....
QYC65082.1    TQETAIGNFFSRAGLVSIIAMPTTGTQNTDG.....YVNWDIDLMGYAQLRRKCELFTYMRFDAEFTFVVAKPNG.....
QWY15927.1    TQETAIGNFFSRAGLVSIITMPTTGTQNTDG.....YVNWDIDLMGYAQLRRKCELFTYMRFDAEFTFVVAKPNG.....
QOP74944.1    TQETAIGNFFSRAGLVSIITMPTTGTQNTDG.....YVNWDIDLMGYAQLRRKCELFTYMRFDAEFTFVVAKPNG.....
QYC65139.1    TQETAIGNFFSRAGLVSIITMPTTGTQNTDG.....YVNWDIDLMGYAQLRRKCELFTYMRFDAEFTFVVAKPNG.....
QYC65136.1    TQETAIGNFFSRAGLVSIITMPTTGTQNTDG.....YVNWDIDLMGYAQLRRKCELFTYMRFDAEFTFVVAKPNG.....
QYC65130.1    TQETAIGNFFSRAGLVSIITMPTTGTQNTDG.....YVNWDIDLMGYAQLRRKCELFTYMRFDAEFTFVVAKPNG.....
QYC65115.1    TQETAIGNFFSRAGLVSIITMPTTGTQNTDG.....YVNWDIDLMGYAQLRRKCELFTYMRFDAEFTFVVAKPNG.....
QYC65114.1    TQETAIGNFFSRAGLVSIITMPTTGTQNTDG.....YVNWDIDLMGYAQLRRKCELFTYMRFDAEFTFVVAKPNG.....
QYC65105.1    TQETAIGNFFSRAGLVSIITMPTTGTQNTDG.....YVNWDIDLMGYAQLRRKCELFTYMRFDAEFTFVVAKPNG.....
```

```
QWF08949.1    TQETAIGNFFSRAGLVSIITMPTTGTQNTDG.....YVNWDIDLMGYAQLRRKCELFTYMRFDAEFTFVVAKPNG.....
QWF08928.1    TQETAIGNFFSRAGLVSIITMPTTGTQNTDG.....YVNWDIDLMGYAQLRRKCELFTYMRFDAEFTFVVAKPNG.....
QWY15921.1    TQETAIGNFFSRAGLVSIITMPTTGTQNTDG.....YVNWDIDLMGYAQLRRKCELFTYMRFDAEFTFVVAKPNG.....
QWY15919.1    TQETAIGNFFSRAGLVSIITMPTTGTQNTDG.....YVNWDIDLMGYAQLRRKCELFTYMRFDAEFTFVVAKPNG.....
QYC65102.1    TQETAIGNFFSRAGLVSIITMPTTGTQNTDG.....YVNWDIDLMGYAQLRRKCELFTYMRFDAEFTFVVAKPNG.....
BCR43082.1    TQETAIGNFFSRAGLVSIITMPTTGTQNTDG.....YVNWDIDLMGYAQLRRKCELFTYMRFDAEFTFVVAKPNG.....
QWY15713.1    TQETAIGNFFSRAGLVSIIIMPTTGTQNTDG.....YVNWDIDLMGYAQLRRKCELFTYMRFDAEFTFVVAKPNG.....
BCI65329.1    TQETAIGNFFSRAGLVSIITMPTTGTQNTDG.....YVNWDIDLMGYAQLRRKCELFTYMRFDAEFTFVVAKPNG.....
QWF08887.1    TQETAIGNFFSRAGLVSIIAMPTTGTQNTDG.....YVNWDIDLMGYAQLRRKCELFTYMRFDAEFTFVVAKPNG.....
QYC65036.1    TQETAIGNFFSRAGLVSIITMPTTGTQNTDG.....YVNWDIDLMGYAQLRRKCELFTYMRFDAEFTFVVAKPNG.....
QTA73913.1    TQETAIGNFFSRAGLVSIITMPTTGTQNTDG.....YVNWDIDLMGYAQLRRKCELFTYMRFDAEFTFVVAKPNG.....
QWY15837.1    TQETAIGNFFSRAGLVSIITMPTTGTQNTDG.....YVNWDIDLMGYAQLRRKCELFTYMRFDAEFTFVVAKPNG.....
QWY15738.1    TQETAIGNFFSRAGLVSIITMPTTGTQNTDG.....YVNWDIDLMGYAQLRRKCELFTYMRFDAEFTFVVAKPNG.....
QTA73911.1    TQETAIGNFFSRAGLVSIITMPTTGTQNTDG.....YVNWDIDLMGYAQLRRKCELFTYMRFDAEFTFVVAKPNG.....
QTA73910.1    TQETAIGNFFSRAGLVSIITMPTTGTQNTDG.....YVNWDIDLMGYAQLRRKCELFTYMRFDAEFTFVVAKPNG.....
QWY15916.1    TQETAIGNFFSRAGLVSIITMPTTGTQNTDG.....YVNWDIDLMGYAQLRRKCELFTYMRFDAEFTFVVAKPNG.....
QWY15915.1    TQETAIGNFFSRAGLVSIITMPTTGTQNTDG.....YVNWDIDLMGYAQLRRKCELFTYMRFDAEFTFVVAKPNG.....
QWY15835.1    TQETAIGNFFSRAGLVSIITMPTTGTQNTDG.....YVNWDIDLMGYAQLRRKCELFTYMRFDAEFTFVVAKPNG.....
QWY15832.1    TQETAIGNFFSRAGLVSIITMPTTGTQNTDG.....YVNWDIDLMGYAQLRRKCELFTYMRFDAEFTFVVAKPNG.....
QWY15830.1    TQETAIGNFFSRAGLVSIITMPTTGTQNTDG.....YVNWDIDLMGYAQLRRKCELFTYMRFDAEFTFVVAKPNG.....
QWY15829.1    TQETAIGNFFSRAGLVSIITMPTTGTQNTDG.....YVNWDIDLMGYAQLRRKCELFTYMRFDAEFTFVVAKPNG.....
QWY15826.1    TQETAIGNFFSRAGLVSIITMPTTGTQNTDG.....YVNWDIDLMGYAQLRRKCELFTYMRFDAEFTFVVAKPNG.....
QWY15802.1    TQETAIGNFFSRAGLVSIITMPTTGTQNTDG.....YVNWDIDLMGYAQLRRKCELFTYMRFDAEFTFVVAKPNG.....
QWY15799.1    TQETAIGNFFSRAGLVSIITMPTTGTQNTDG.....YVNWDIDLMGYAQLRRKCELFTYMRFDAEFTFVVAKPNG.....
QWY15788.1    TQETAIGNFFSRAGLVSIITMPTTGTQNTDG.....YVNWDIDLMGYAQLRRKCELFTYMRFDAEFTFVVAKPNG.....
QWY15783.1    TQETAIGNFFSRAGLVSIITMPTTGTQNTDG.....YVNWDIDLMGYAQLRRKCELFTYMRFDAEFTFVVAKPNG.....
QWY15768.1    TQETAIGNFFSRAGLVSIITMPTTGTQNTDG.....YVNWDIDLMGYAQLRRKCELFTYMRFDAEFTFVVAKPNG.....
QWY15766.1    TQETAIGNFFSRAGLVSIITMPTTGTQNTDG.....YVNWDIDLMGYAQLRRKCELFTYMRFDAEFTFVVAKPNG.....
QWY15764.1    TQETAIGNFFSRAGLVSIITMPTTGTQNTDG.....YVNWDIDLMGYAQLRRKCELFTYMRFDAEFTFVVAKPNG.....
QWY15763.1    TQETAIGNFFSRAGLVSIITMPTTGTQNTDG.....YVNWDIDLMGYAQLRRKCELFTYMRFDAEFTFVVAKPNG.....
QWY15760.1    TQETAIGNFFSRAGLVSIITMPTTGTQNTDG.....YVNWDIDLMGYAQLRRKCELFTYMRFDAEFTFVVAKPNG.....
QWF08768.1    TQETAIGNFFSRAGLVSIITMPTTGTQNTDG.....YVNWDIDLMGYAQLRRKCELFTYMRFDAEFTFVVAKPNG.....
QWF08766.1    TQETAIGNFFSRAGLVSIITMPTTGTQNTDG.....YVNWDIDLMGYAQLRRKCELFTYMRFDAEFTFVVAKPNG.....
QWY15790.1    TQETAIGNFFSRAGLVSIITMPTTGTQNTDG.....YVNWDIDLMGYAQLRRKCELFTYMRFDAEFTFVAAKPNG.....
QWF08769.1    TQETAIGNFFSRAGLVSIITMPTTGTQNTDG.....YVNWDIDLMGYAQLRRKCELFTYMRFDAEFTFVVAKPNG.....
QWY15930.1    TQETAIGNFFSRAGLVSIITMPTTGTQNTDG.....YVNWDIDLMGYAQLRRKCELFTYMRFDAEFTFVVAKPNG.....
QWY15833.1    TQETAIGNFFSRAGLVSIITMPTTGTQNTDG.....YVNWDIDLMGYAQLRRKCELFTYMRFDAEFTFVVAKPNG.....
QWY15827.1    TQETAIGNFFSRAGLVSIITMPTTGTQNTDG.....YVNWDIDLMGYAQLRRKCELFTYMRFDAEFTFVVAKPNG.....
QWY15838.1    TQETAIGNFFSRAGLVSIITMPTTGTQNTDG.....YVNWDIDLMGYAQLRRKCELFTYMRFDAEFTFVVAKPNG.....
BBU37460.1    TQETAIGNFFSRAGLVSIITMPTTGTQNTDG.....YANWDIDLMGYAQLRRKCELFTYMRFDAEFTFVVAKPNG.....
QWF08920.1    TQETAIGNFFSRAGLVSIITMPTTGTQNTDG.....YANWDIDLMGYAQLRRKCELFTYMRFDAEFTFVVAKPNG.....
QWY15769.1    TQETAIGNFFSRAGLVSIITMPTTGTQNTDG.....YVNWDIDLMGYAQLRRKCELFTYMRFDAEFTFVVAKPNG.....
QWY15755.1    TQETAIGNFFSRAGLVSIITMPTTGTQNTDG.....YVNWDIDLMGYAQLRRKCELFTYMRFDAEFTFVVAKPNG.....
QWY15702.1    TQETAIGNFFSRAGLVSIITMPTTGTQNTDG.....YVNWDIDLMGYAQLRRKCELFTYMRFDAEFTFVVAKPNG.....
QWY15739.1    TQETAIGNFFSRAGLVSIITMPTTGTQNTDG.....YVNWDIDLMGYAQLRRKCELFTYMRFDAEFTFVVAKPNG.....
QWY15737.1    TQETAIGNFFSRAGLVSIITMPTTGTQNTDG.....YVNWDIDLMGYAQLRRKCELFTYMRFDAEFTFVVAKPNG.....
QWF08788.1    TQETAIGNFFSRAGLVSIITMPTTGTQNTDG.....YVNWDIELMGYAQLRRKCELFTYMRFDAEFTFVVAKPNG.....
QYC65122.1    TQETAIGNFFSRAGLVSIITMPTTGTQNTDG.....YVNWDIDLMGYAQLRRKCELFTYMRFDAEFTFVVAKPNG.....
QWF08891.1    TQETAIGNFFSRAGLVSIITMPTTGTQNTDG.....YVNWDIDLMGYAQLRRKCELFTYMRFDAEFTFVVAKPNG.....
QWY15903.1    TQETAIGNFFSRAGLVSIITMPTTGTQNTDG.....YVNWDIDLMGYAQLRRKCELFTYMRFDAEFTFVVAKPNG.....
BCI65366.1    TQETAIGNFFSRAGLVSIITMPTTGTQNTDG.....YVNWDIDLMGYAQLRRKCELFTYMRFDAEFTFVVAKPNG.....
BCG67045.1    TQETAIGNFFSRAGLVSIITMPTTGTQNTDG.....YVNWDIDLMGYAQLRRKCELFTYMRFDAEFTFVVAKPNG.....
BCG67044.1    TQETAIGNFFSRAGLVSIITMPTTGTQNTDG.....YVNWDIDLMGYAQLRRKCELFTYMRFDAEFTFVVAKPNG.....
QWY15819.1    TQETAIGNFFSRAGLVSIITMPTTGTQNTDG.....YVNWDIDLMGYAQLRRKCELFTYMRFDAEFTFVVAKPNG.....
QWY15818.1    TQETAIGNFFSRAGLVSIITMPTTGTQNTDG.....YVNWDIDLMGYAQLRRKCELFTYMRFDAEFTFVVAKPNG.....
QTA73912.1    TQETAIGNFFSRAGLVSIITMPTTGTQNTDG.....YVNWDIDLMGYAQLRRKCELFTYMRFDAEFTFVVAKPNG.....
QWY15914.1    TQETAIGNFFSRAGLVSIITMPTTGTQNTDG.....YVNWDIDLMGYAQLRRKCELFTYMRFDAEFTFVVAKPNG.....
QWY15909.1    TQETAIGNFFSRAGLVSIITMPTTGTQNTDG.....YVNWDIDLMGYAQLRRKCELFTYMRFDAEFTFVVAKPNG.....
QWY15905.1    TQETAIGNFFSRAGLVSIITMPTTGTQNTDG.....YVNWDIDLMGYAQLRRKCELFTYMRFDAEFTFVVAKPNG.....
QWY15856.1    TQETAIGNFFSRAGLVSIITMPTTGTQNTDG.....YVNWDIDLMGYAQLRRKCELFTYMRFDAEFTFVVAKPNG.....
QWY15850.1    TQETAIGNFFSRAGLVSIITMPTTGTQNTDG.....YVNWDIDLMGYAQLRRKCELFTYMRFDAEFTFVVAKPNG.....
QWY15849.1    TQETAIGNFFSRAGLVSIITMPTTGTQNTDG.....YVNWDIDLMGYAQLRRKCELFTYMRFDAEFTFVVAKPNG.....
QWY15847.1    TQETAIGNFFSRAGLVSIITMPTTGTQNTDG.....YVNWDIDLMGYAQLRRKCELFTYMRFDAEFTFVVAKPNG.....
QWY15845.1    TQETAIGNFFSRAGLVSIITMPTTGTQNTDG.....YVNWDIDLMGYAQLRRKCELFTYMRFDAEFTFVVAKPNG.....
QWY15836.1    TQETAIGNFFSRAGLVSIITMPTTGTQNTDG.....YVNWDIDLMGYAQLRRKCELFTYMRFDAEFTFVVAKPNG.....
QWY15765.1    TQETAIGNFFSRAGLVSIITMPTTGTQNTDG.....YVNWDIDLMGYAQLRRKCELFTYMRFDAEFTFVVAKPNG.....
QWY15734.1    TQETAIGNFFSRAGLVSIITMPTTGTQNTDG.....YVNWDIDLMGYAQLRRKCELFTYMRFDAEFTFVVAKPNG.....
QWY15855.1    TQETAIGNFFSRAGLVSIITMPTTGTQNTDG.....YVNWDIDLMGYAQLRRKCELFTYMRFDAEFTFVVAKPNG.....
QWY15831.1    TQETAIGNFFSRAGLVSIITMPTTGTQNTDG.....YVNWDIDLMGYAQLRRKCELFTYMRFDAEFTFVVAKPNG.....
QWY15798.1    TQETAIGNFFSRAGLVSIITMPTTGTQNTDG.....YVNWDIDLMGYAQLRRKCELFTYMRFDAEFTFVVAKPNG.....
QWY15797.1    TQETAIGNFFSRAGLVSIITMPTTGTQNTDG.....YVNWDIDLMGYAQLRRKCELFTYMRFDAEFTFVVAKPNG.....
QWY15796.1    TQETAIGNFFSRAGLVSIITMPTTGTQNTDG.....YVNWDIDLMGYAQLRRKCELFTYMRFDAEFTFVVAKPNG.....
QWY15795.1    TQETAIGNFFSRAGLVSIITMPTTGTQNTDG.....YVNWDIDLMGYAQLRRKCELFTYMRFDAEFTFVVAKPNG.....
QWY15794.1    TQETAIGNFFSRAGLVSIITMPTTGTQNTDG.....YVNWDIDLMGYAQLRRKCELFTYMRFDAEFTFVVAKPNG.....
QWY15971.1    TQETAIGNFFSRAGLVSIITMPTTGTQNTDG.....YVNWDIDLMGYAQLRRKCELFTYMRFDAEFTFVVAKPNG.....
QWY15897.1    TQETAIGNFFSRAGLVSIITMPTTGTQNTDG.....YVNWDIDLMGYAQLRRKCELFTYMRFDAEFTFVVAKPNG.....
QWY15742.1    TQETAIGNFFSRAGLVSIITMPTTGTQNTDG.....YVNWDIDLMGYAQLRRKCELFTYMRFDAEFTFVVAKPNG.....
QWY15741.1    TQETAIGNFFSRAGLVSIITMPTTGTQNTDG.....YVNWDIDLMGYAQLRRKCELFTYMRFDAEFTFVVAKPNG.....
QWY15923.1    TQETAIGNFFSRAGLVSIITMPTTGTQNIDG.....YVNWDIDLMGYAQLRRKCELFTYMRFDAEFTFVVAKPNG.....
QWF08957.1    TQETAIGNFFSRAGLVSIITMPTTGTQNIDG.....YVNWDIDLMGYAQLRRKCELFTYMRFDAEFTFVVAKPNG.....
UEP53407.1    TQETAIGNFFSRAGLVSIITMPTTGTQNIDG.....YVNWDIDLMGYAQLRRKCELFTYMRFDAEFTFVVAKPNG.....
QWY15922.1    TQETAIGNFFSRAGLVSIITMPTTGTQNIDG.....YVNWDIDLMGYAQLRRKCELFTYMRFDAEFTFVVAKPNG.....
QWY15848.1    TQETAIGNFFSRAGLVSIITMPTTGTQNIDG.....YVNWDIDLMGYAQLRRKCELFTYMRFDAEFTFVVAKPNG.....
UEP53405.1    TQETAIGNFFSRAGLVSIITMPTTGTQNTDG.....YVNWDIDLMGYAQLRRKCELFTYMRFDAEFTFVVAKPNG.....
UEC49855.1    TQETAIGNFFSRAGLVSIITMPTTGTQNTDG.....YVNWDIDLMGYAQLRRKCELFTYMRFDAEFTFVVAKPNG.....
QWY15851.1    TQETAIGNFFSRAGLVSIITMPTTGTQNTDG.....YVNWDIDLMGYAQLRRKCELFTYMRFDAEFTFVVAKPNG.....
QYC65112.1    TQETAIGNFFSRAGLVSIITMPTTGTQNTDG.....YVNWDIDLMGYAQLRRKCELFTYMRFDAEFTFVVAKPNG.....
QOP74942.1    TQETAIGNFFSRAGLVSIITMPTTGTQNTDG.....YVNWDIDLMGYAQLRRKCELFTYMRFDAEFTFVVAKPNG.....
QLH02013.1    TQETAIGNFFSRAGLVSIITMPTTGTQNTDG.....YVNWDIDLMGYAQLRRKCELFTYMRFDAEFTFVVAKPNG.....
QIH53923.1    TQETAIGNFFSRAGLVSIITMPTTGTQNTDG.....YVNWDIDLMGYAQLRRKCELFTYMRFDAEFTFVVAKPNG.....
QIH53918.1    TQETAIGNFFSRAGLVSIITMPTTGTQNTDG.....YVNWDIDLMGYAQLRRKCELFTYMRFDAEFTFVVAKPNG.....
QIH53915.1    TQETAIGNFFSRAGLVSIITMPTTGTQNTDG.....YVNWDIDLMGYAQLRRKCELFTYMRFDAEFTFVVAKPNG.....
QIH53914.1    TQETAIGNFFSRAGLVSIITMPTTGTQNTDG.....YVNWDIDLMGYAQLRRKCELFTYMRFDAEFTFVVAKPNG.....
QYC65127.1    TQETAIGNFFSRAGLVSIITMPTTGTQNTDG.....YVNWDIDLMGYAQLRRKCELFTYMRFDAEFTFVVAKPNG.....
QIL87534.1    TQETAIGNFFSRAGLVSIITMPTTGTQNTDG.....YVNWDIDLMGYAQLRRKCELFTYMRFDAEFTFVVAKPNG.....
QWF08936.1    TQETAIGNFFSRAGLVSIITMPTTGTQNTDG.....YVNWDIDLMGYAQLRRKCELFTYMRFDAEFTFVVAKPNG.....
QIL87532.1    TQETAIGNFFSRAGLVSIITMPTTGTQNTDG.....YVNWDIDLMGYAQLRRKCELFTYMRFDAEFTFVVAKPNG.....
QYC65089.1    TQETAIGNFFSRAGLVSIITMPTTGTQNTDG.....YVNWDIDLMGYAQLRRKCELFTYMRFDAEFTFVVAKPNG.....
QWF08950.1    TQETAIGNFFSRAGLVSIITMPTTGTQNTDG.....YVNWDIDLMGYAQLRRKCELFTYMRFDAEFTFVVAKPNG.....
QIL87530.1    TQETAIGNFFSRAGLVSIITMPTTGTQNTDG.....YVNWDIDLMGYAQLRRKCELFTYMRFDAEFTFVVAKPNG.....
QWF08820.1    TQETAIGNFFSRAGLVSIITMPTTGTQNTDG.....YVNWDIDLMGYAQLRRKCELFTYMRFDAEFTFVVAKPNG.....
QIL87529.1    TQETAIGNFFSRAGLVSIITMPTTGTQNTDG.....YVNWDIDLMGYAQLRRKCELFTYMRFDAEFTFVVAKPNG.....
QIL87528.1    TQETAIGNFFSRAGLVSIITMPTTGTQNTDG.....YVNWDIDLMGYAQLRRKCELFTYMRFDAEFTFVVAKPNG.....
QYC65143.1    TQETAIGNFFSRAGLVSIITMPTTGTQNTDG.....YVNWDIDLMGYAQLRRKCELFTYMRFDAEFTFVVAKPNG.....
QIL87525.1    TQETAIGNFFSRAGLVSIITMPTTGTQNTDG.....YVNWDIDLMGYAQLRRKCELFTYMRFDAEFTFVVAKPNG.....
QYC65141.1    TQETAIGNFFSRAGLVSIITMPTTGTQNTDG.....YVNWDIDLMGYAQLRRKCELFTYMRFDAEFTFVVAKPNG.....
QYC65084.1    TQETAIGNFFSRAGLVSIITMPTTGTQNTDG.....YVNWDIDLMGYAQLRRKCELFTYMRFDAEFTFVVAKPNG.....
QWF08947.1    TQETAIGNFFSRAGLVSIITMPTTGTQNTDG.....YVNWDIDLMGYAQLRRKCELFTYMRFDAEFTFVVAKPNG.....
QWF08922.1    TQETAIGNFFSRAGLVSIITMPTTGTQNTDG.....YVNWDIDLMGYAQLRRKCELFTYMRFDAEFTFVVAKPNG.....
QIL87524.1    TQETAIGNFFSRAGLVSIITMPTTGTQNTDG.....YVNWDIDLMGYAQLRRKCELFTYMRFDAEFTFVVAKPNG.....
QIL87520.1    TQETAIGNFFSRAGLVSIITMPTTGTQNTDG.....YVNWDIDLMGYAQLRRKCELFTYMRFDAEFTFVVAKPNG.....
QIL87518.1    TQETAIGNFFSRAGLVSIITMPTTGTQNTDG.....YVNWDIDLMGYAQLRRKCELFTYMRFDAEFTFVVAKPNG.....
QYC65103.1    TQETAIGNFFSRAGLVSIITMPTTGTQNTDG.....YVNWDIDLMGYAQLRRKCELFTYMRFDAEFTFVVAKPNG.....
QWF08944.1    TQETAIGNFFSRAGLVSIITMPTTGTQNTDG.....YVNWDIDLMGYAQLRRKCELFTYMRFDAEFTFVVAKPNG.....
QWF08943.1    TQETAIGNFFSRAGLVSIITMPTTGTQNTDG.....YVNWDIDLMGYAQLRRKCELFTYMRFDAEFTFVVAKPNG.....
QWF08927.1    TQETAIGNFFSRAGLVSIITMPTTGTQNTDG.....YVNWDIDLMGYAQLRRKCELFTYMRFDAEFTFVVAKPNG.....
QWF08942.1    TQETAIGNFFSRAGLVSIITMPTTGTQNTDG.....YVNWDIDLMGYAQLRRKCELFTYMRFDAEFTFVVAKPNG.....
QWF08940.1    TQETAIGNFFSRAGLVSIITMPTTGTQNTDG.....YVNWDIDLMGYAQLRRKCELFTYMRFDAEFTFVVAKPNG.....
QWF08912.1    TQETAIGNFFSRAGLVSIITMPTTGTQNTDG.....YVNWDIDLMGYAQLRRKCELFTYMRFDAEFTFVVAKPNG.....
QWF08907.1    TQETAIGNFFSRAGLVSIITMPTTGTQNTDG.....YVNWDIDLMGYAQLRRKCELFTYMRFDAEFTFVVAKPNG.....
QWF08904.1    TQETAIGNFFSRAGLVSIITMPTTGTQNTDG.....YVNWDIDLMGYAQLRRKCELFTYMRFDAEFTFVVAKPNG.....
QWF08886.1    TQETAIGNFFSRAGLVSIITMPTTGTQNTDG.....YVNWDIDLMGYAQLRRKCELFTYMRFDAEFTFVVAKPNG.....
QWY15877.1    TQETAIGNFFSRAGLVSIITMPTTGTQNTDG.....YVNWDIDLMGYAQLRRKCELFTYMRFDAEFTFVVAKPNG.....
QWY15965.1    TQETAIGNFFSRAGLVSIITMPTTGTQNTDG.....YVNWDIDLMGYAQLRRKCELFTYMRFDAEFTFVVAKPNG.....
UEC49851.1    TQETAIGNFFSRAGLVSIITMPTTGTQNTDG.....YVNWDIDLMGYAQLRRKCELFTYMRFDAEFTFVVAKPNG.....
UEC49850.1    TQETAIGNFFSRAGLVSIITMPTTGTQNTDG.....YVNWDIDLMGYAQLRRKCELFTYMRFDAEFTFVVAKPNG.....
UEC49849.1    TQETAIGNFFSRAGLVSIITMPTTGTQNTDG.....YVNWDIDLMGYAQLRRKCELFTYMRFDAEFTFVVAKPNG.....
QWY15963.1    TQETAIGNFFSRAGLVSIITMPTTGTQNTDG.....YVNWDIDLMGYAQLRRKCELFTYMRFDAEFTFVVAKPNG.....
QWY15972.1    TQETAIGNFFSRAGLVSIITMPTTGTQNTDG.....YVNWDIDLMGYAQLRRKCELFTYMRFDAEFTFVVAKPNG.....
QWY15958.1    TQETAIGNFFSRAGLVSIITMPTTGTQNTDG.....YVNWDIDLMGYAQLRRKCELFTYMRFDAEFTFVVAKPNG.....
QWY15899.1    TQETAIGNFFSRAGLVSIITMPTTGTQNTDG.....YVNWDIDLMGYAQLRRKCELFTYMRFDAEFTFVVAKPNG.....
QWY15898.1    TQETAIGNFFSRAGLVSIITMPTTGTQNTDG.....YVNWDIDLMGYAQLRRKCELFTYMRFDAEFTFVVAKPNG.....
QWY15895.1    TQETAIGNFFSRAGLVSIITMPTTGTQNTDG.....YVNWDIDLMGYAQLRRKCELFTYMRFDAEFTFVVAKPNG.....
QWY15893.1    TQETAIGNFFSRAGLVSIITMPTTGTQNTDG.....YVNWDIDLMGYAQLRRKCELFTYMRFDAEFTFVVAKPNG.....
QWY15892.1    TQETAIGNFFSRAGLVSIITMPTTGTQNTDG.....YVNWDIDLMGYAQLRRKCELFTYMRFDAEFTFVVAKPNG.....
QWY15891.1    TQETAIGNFFSRAGLVSIITMPTTGTQNTDG.....YVNWDIDLMGYAQLRRKCELFTYMRFDAEFTFVVAKPNG.....
QWY15970.1    TQETAIGNFFSRAGLVSIITMPTTGTQNTDG.....YVNWDIDLMGYAQLRRKCELFTYMRFDAEFTFVVAKPNG.....
QWY15966.1    TQETAIGNFFSRAGLVSIITMPTTGTQNTDG.....YVNWDIDLMGYAQLRRKCELFTYMRFDAEFTFVVAKPNG.....
QWY15962.1    TQETAIGNFFSRAGLVSIITMPTTGTQNTDG.....YVNWDIDLMGYAQLRRKCELFTYMRFDAEFTFVVAKPNG.....
QWY15961.1    TQETAIGNFFSRAGLVSIITMPTTGTQNTDG.....YVNWDIDLMGYAQLRRKCELFTYMRFDAEFTFVVAKPNG.....
QWY15925.1    TQETAIGNFFSRAGLVSIITMPTTGTQNTDG.....YVNWDIDLMGYAQLRRKCELFTYMRFDAEFTFVVAKPNG.....
QWY15773.1    TQETAIGNFFSRAGLVSIITMPTTGTQNTDG.....YVNWDIDLMGYAQLRRKCELFTYMRFDAEFTFVVAKPNG.....
QWY15924.1    TQETAIGNFFSRAGLVSIITMPTTGTQNTDG.....YVNWDIDLMGYAQLRRKCELFTYMRFDAEFTFVVAKPNG.....
QWY15910.1    TQETAIGNFFSRAGLVSIITMPTTGTQNTDG.....YVNWDIDLMGYAQLRRKCELFTYMRFDAEFTFVVAKPNG.....
```

```
QWY15824.1   TQETAIGNFFSRAGLVSIITMPTTGTQNTDG.....YVNWDIDLMGYAQLRRKCELFTYMRFDAEFTFVVAKPNG.....
QWY15823.1   TQETAIGNFFSRAGLVSIITMPTTGTQNTDG.....YVNWDIDLMGYAQLRRKCELFTYMRFDAEFTFVVAKPNG.....
QWY15822.1   TQETAIGNFFSRAGLVSIITMPTTGTQNTDG.....YVNWDIDLMGYAQLRRKCELFTYMRFDAEFTFVVAKPNG.....
QWY15821.1   TQETAIGNFFSRAGLVSIITMPTTGTQNTDG.....YVNWDIDLMGYAQLRRKCELFTYMRFDAEFTFVVAKPNG.....
QWY15816.1   TQETAIGNFFSRAGLVSIITMPTTGTQNTDG.....YVNWDIDLMGYAQLRRKCELFTYMRFDAEFTFVVAKPNG.....
QWY15778.1   TQETAIGNFFSRAGLVSIITMPTTGTQNTDG.....YVNWDIDLMGYAQLRRKCELFTYMRFDAEFTFVVAKPNG.....
QWY15801.1   TQETAIGNFFSRAGLVSIITMPTTGTQNTDG.....YVNWDIDLMGYAQLRRKCELFTYMRFDAEFTFVVAKPNG.....
QWY15791.1   TQETAIGNFFSRAGLVSIITMPTTGTQNTDG.....YVNWDIDLMGYAQLRRKCELFTYMRFDAEFTFVVAKPNG.....
QWY15775.1   TQETAIGNFFSRAGLVSIITMPTTGTQNTDG.....YVNWDIDLMGYAQLRRKCELFTYMRFDAEFTFVVAKPNG.....
QWY15774.1   TQETAIGNFFSRAGLVSIITMPTTGTQNTDG.....YVNWDIDLMGYAQLRRKCELFTYMRFDAEFTFVVAKPNG.....
BCR43107.1   TQETAIGNFFSRAGLVSIITMPTTGTQNTDG.....YVNWDIDLMGYAQLRRKCELFTYMRFDAEFTFVVAKPNG.....
QYC65183.1   TQETAIGNFFSRAGLVSIITMPTTGTQNTDG.....YVNWDIDLMGYAQLRRKCELFTYMRFDAEFTFVVAKPNG.....
BCR43094.1   TQETAIGNFFSRAGLVSIITMPTTGTQNTDG.....YVNWDIDLMGYAQLRRKCELFTYMRFDAEFTFVVAKPNG.....
BCR43081.1   TQETAIGNFFSRAGLVSIITMPTTGTQNTDG.....YVNWDIDLMGYAQLRRKCELFTYMRFDAEFTFVVAKPNG.....
BCG67017.1   TQETAIGNFFSRAGLVSIITMPTTGTQNTDG.....YVNWDIDLMGYAQLRRKCELFTYMRFDAEFTFVVAKPNG.....
BCG67015.1   TQETAIGNFFSRAGLVSIITMPTTGTQNTDG.....YVNWDIDLMGYAQLRRKCELFTYMRFDAEFTFVVAKPNG.....
BCG67007.1   TQETAIGNFFSRAGLVSIITMPTTGTQNTDG.....YVNWDIDLMGYAQLRRKCELFTYMRFDAEFTFVVAKPNG.....
BCG67005.1   TQETAIGNFFSRAGLVSIITMPTTGTQNTDG.....YVNWDIDLMGYAQLRRKCELFTYMRFDAEFTFVVAKPNG.....
QYC65184.1   TQETAIGNFFSRAGLVSIITMPTTGTQNTDG.....YVNWDIDLMGYAQLRRKCELFTYMRFDAEFTFVVAKPNG.....
QWF08853.1   TQETAIGNFFSRAGLVSIITMPTTGTQNTDG.....YVNWDIDLMGYAQLRRKCELFTYMRFDAEFTFVVAKPNG.....
QYC65140.1   TQETAIGNFFSRAGLVSIITMPTTGTQNTDG.....YVNWDIDLMGYAQLRRKCELFTYMRFDAEFTFVVAKPNG.....
QYC65133.1   TQETAIGNFFSRAGLVSIITMPTTGTQNTDG.....YVNWDIDLMGYAQLRRKCELFTYMRFDAEFTFVVAKPNG.....
QYC65110.1   TQETAIGNFFSRAGLVSIITMPTTGTQNTDG.....YVNWDIDLMGYAQLRRKCELFTYMRFDAEFTFVVAKPNG.....
QYC65108.1   TQETAIGNFFSRAGLVSIITMPTTGTQNTDG.....YVNWDIDLMGYAQLRRKCELFTYMRFDAEFTFVVAKPNG.....
QYC65098.1   TQETAIGNFFSRAGLVSIITMPTTGTQNTDG.....YVNWDIDLMGYAQLRRKCELFTYMRFDAEFTFVVAKPNG.....
QYC65097.1   TQETAIGNFFSRAGLVSIITMPTTGTQNTDG.....YVNWDIDLMGYAQLRRKCELFTYMRFDAEFTFVVAKPNG.....
QYC65094.1   TQETAIGNFFSRAGLVSIITMPTTGTQNTDG.....YVNWDIDLMGYAQLRRKCELFTYMRFDAEFTFVVAKPNG.....
QYC65091.1   TQETAIGNFFSRAGLVSIITMPTTGTQNTDG.....YVNWDIDLMGYAQLRRKCELFTYMRFDAEFTFVVAKPNG.....
QYC65057.1   TQETAIGNFFSRAGLVSIITMPTTGTQNTDG.....YVNWDIDLMGYAQLRRKCELFTYMRFDAEFTFVVAKPNG.....
QWF08850.1   TQETAIGNFFSRAGLVSIITMPTTGTQNTDG.....YVNWDIDLMGYAQLRRKCELFTYMRFDAEFTFVVAKPNG.....
QYC65056.1   TQETAIGNFFSRAGLVSIITMPTTGTQNTDG.....YVNWDIDLMGYAQLRRKCELFTYMRFDAEFTFVVAKPNG.....
QWF08849.1   TQETAIGNFFSRAGLVSIITMPTTGTQNTDG.....YVNWDIDLMGYAQLRRKCELFTYMRFDAEFTFVVAKPNG.....
QWF08969.1   TQETAIGNFFSRAGLVSIITMPTTGTQNTDG.....YVNWDIDLMGYAQLRRKCELFTYMRFDAEFTFVVAKPNG.....
QWF08848.1   TQETAIGNFFSRAGLVSIITMPTTGTQNTDG.....YVNWDIDLMGYAQLRRKCELFTYMRFDAEFTFVVAKPNG.....
QWF08968.1   TQETAIGNFFSRAGLVSIITMPTTGTQNTDG.....YVNWDIDLMGYAQLRRKCELFTYMRFDAEFTFVVAKPNG.....
QWF08965.1   TQETAIGNFFSRAGLVSIITMPTTGTQNTDG.....YVNWDIDLMGYAQLRRKCELFTYMRFDAEFTFVVAKPNG.....
QWF08956.1   TQETAIGNFFSRAGLVSIITMPTTGTQNTDG.....YVNWDIDLMGYAQLRRKCELFTYMRFDAEFTFVVAKPNG.....
QWF08952.1   TQETAIGNFFSRAGLVSIITMPTTGTQNTDG.....YVNWDIDLMGYAQLRRKCELFTYMRFDAEFTFVVAKPNG.....
QWF08903.1   TQETAIGNFFSRAGLVSIITMPTTGTQNTDG.....YVNWDIDLMGYAQLRRKCELFTYMRFDAEFTFVVAKPNG.....
QWF08902.1   TQETAIGNFFSRAGLVSIITMPTTGTQNTDG.....YVNWDIDLMGYAQLRRKCELFTYMRFDAEFTFVVAKPNG.....
QWF08895.1   TQETAIGNFFSRAGLVSIITMPTTGTQNTDG.....YVNWDIDLMGYAQLRRKCELFTYMRFDAEFTFVVAKPNG.....
QWF08888.1   TQETAIGNFFSRAGLVSIITMPTTGTQNTDG.....YVNWDIDLMGYAQLRRKCELFTYMRFDAEFTFVVAKPNG.....
QWF08893.1   TQETAIGNFFSRAGLVSIITMPTTGTQNTDG.....YVNWDIDLMGYAQLRRKCELFTYMRFDAEFTFVVAKPNG.....
QWF08892.1   TQETAIGNFFSRAGLVSIITMPTTGTQNTDG.....YVNWDIDLMGYAQLRRKCELFTYMRFDAEFTFVVAKPNG.....
QWF08876.1   TQETAIGNFFSRAGLVSIITMPTTGTQNTDG.....YVNWDIDLMGYAQLRRKCELFTYMRFDAEFTFVVAKPNG.....
QWF08868.1   TQETAIGNFFSRAGLVSIITMPTTGTQNTDG.....YVNWDIDLMGYAQLRRKCELFTYMRFDAEFTFVVAKPNG.....
QWF08862.1   TQETAIGNFFSRAGLVSIITMPTTGTQNTDG.....YVNWDIDLMGYAQLRRKCELFTYMRFDAEFTFVVAKPNG.....
QWF08861.1   TQETAIGNFFSRAGLVSIITMPTTGTQNTDG.....YVNWDIDLMGYAQLRRKCELFTYMRFDAEFTFVVAKPNG.....
QWF08860.1   TQETAIGNFFSRAGLVSIITMPTTGTQNTDG.....YVNWDIDLMGYAQLRRKCELFTYMRFDAEFTFVVAKPNG.....
QWF08859.1   TQETAIGNFFSRAGLVSIITMPTTGTQNTDG.....YVNWDIDLMGYAQLRRKCELFTYMRFDAEFTFVVAKPNG.....
QWF08858.1   TQETAIGNFFSRAGLVSIITMPTTGTQNTDG.....YVNWDIDLMGYAQLRRKCELFTYMRFDAEFTFVVAKPNG.....
QWF08854.1   TQETAIGNFFSRAGLVSIITMPTTGTQNTDG.....YVNWDIDLMGYAQLRRKCELFTYMRFDAEFTFVVAKPNG.....
QWF08929.1   TQETAIGNFFSRAGLVSIITMPTTGTQNTDG.....YVNWDIDLMGYAQLRRKCELFTYMRFDAEFTFVVAKPNG.....
QYC65137.1   TQETAIGNFFSRAGLVSIITMPTTGTQNTDG.....YVNWDIDLMGYAQLRRKCELFTYMRFDAEFTFVVAKPNG.....
QYC65100.1   TQETAIGNFFSRAGLVSIITMPTTGTQNTDG.....YVNWDIDLMGYAQLRRKCELFTYMRFDAEFTFVVAKPNG.....
QWY15926.1   TQETAIGNFFSRAGLVSIITMPSTGTQNTDG.....YVNWDIDLMGYAQLRRKCELFTYMRFDAEFTFVVAKPNG.....
QWF08962.1   TQETAIGNFFSRAGLVSIITMPTTGTQNTDG.....YVNWDIDLMGYAQLRRKCELFTYMRFDAEFTFVVAKPNG.....
QWF08909.1   TQETAIGNFFSRAGLVSIITMPTTGTQNTDG.....YVNWDIDLMGYAQLRRKCELFTYMRFDAEFTFVVAKPNG.....
QWY15854.1   TQETAIGNFFSRAGLVSIITMPTMGTQNTDG.....YVNWDIDLMGYAQLRRKCELFTYMRFDAEFTFVVAKPNG.....
QWY15808.1   TQETAIGNFFSRAGLVSIITMPTTGTQNTDG.....YVNWDIDLMGYAQLRRKCELFTYMRFDAEFTFVVAKPNG.....
QWF08770.1   TQETAIGNFFSRAGLVSIITMPTTGTQNTDG.....YVNWDIDLMGYAQLRRKCELFTYMRFDAEFTFVVAKPNG.....
QWY15728.1   TQETAIGNFFSRAGLVSIITMPTTGTQNTDG.....YVNWDIDLMGYAQLRRKCELFTYMRFDAEFTFVVAKPNG.....
QYC65126.1   TQETAIGNFFSRAGLVSIITMPTTGTQNTDG.....YVNWDIDLMGYAQLRRKCELFTYMRFDAEFTFVVAKPNG.....
QWF08827.1   TQETAIGNFFSRAGLVSIITMPTTGTQNTDG.....YVNWDIDLMGYAQLRRKCELFTYMRFDAEFTFVVAKPNG.....
QYC65072.1   TQETAIGNFFSRAGLVSIITMPTTGTQNTDG.....YVNWDIDLMGYAQLRRKCELFTYMRFDAEFTFVVAKPNG.....
QYC65067.1   TQETAIGNFFSRAGLVSIITMPTTGTQNTDG.....YVNWDIDLMGYAQLRRKCELFTYMRFDAEFTFVVAKPNG.....
QWY15767.1   TQETAIGNFFSRAGLVSIITMPTTGTQNTDG.....YVNWDIDLMGYAQLRRKCELFTYMRFDAEFTFVVAKPNG.....
QWY15735.1   TQETAIGNFFSRAGLVSIITMPTTGTQNTDG.....YVNWDIDLMGYAQLRRKCELFTYMRFDAEFTFVVAKPNG.....
BCB92153.1   TQETAIGNFFSRAGLVSIITMPTTGTQNTDG.....YVNWDIDLMGYAQLRRKCELFTYMRFDAEFTFVVAKPNG.....
QWF08966.1   TQETAIGNFFSRAGLVSIITMPTTGTQNTDG.....YVNWDIDLMGYAQLRRKCELFTYMRFDAEFTFVVAKPNG.....
QIH53917.1   TQETAIGNFFSRAGLVSIITMPTTGTQNTDG.....YVNWDIDLMGYAQLRRKCELFTYMRFDAEFTFVVAKPNG.....
QYC65092.1   TQETAIGNFFSRAGLVSIITMPTTGTQNTDG.....YVNWDIDLMGYAQLRRKCELFTYMRFDAEFTFVVAKPNG.....
QWY15879.1   TQETAIGNFFSRAGLVSIITMPTTGTQNTDG.....YVNWDIDLMGYAQLRRKCELFTYMRFDAEFTFVVAKPNG.....
QWY15777.1   TQETAIGNFFSRAGLVSIITMPTTGTQNTDG.....YVNWDIDLMGYAQLRRKCELFTYMRFDAEFTFVVAKPNG.....
BCR43105.1   TQETAIGNFFSRAGLVSIITMPTTGTQNTDG.....YVNWDIDLMGYAQLRRKCELFTYMRFDAEFTFVVAKPNG.....
QWF08826.1   TQETAIGNFFSRAGLVSIITMPTTGTQNTDG.....YVNWDIDLMGYAQLRRKCELFTYMRFDAEFTFVVAKPNG.....
BCG67024.1   TQETAIGNFFSRAGLVSIITMPTTGTQNTDG.....YVNWDIDLMGYAQLRRKCELFTYMRFDAEFTFVVAKPNG.....
BCG66999.1   TQETAIGNFFSRAGLVSIITMPTTGTQNTDG.....YVNWDIDLMGYAQLRRKCELFTYMRFDAEFTFVVAKPNG.....
QWY15752.1   TQETAIGNFFSRAGLVSIITMPTTGTQNTDG.....YVNWDIDLMGYAQLRRKCELFTYMRFDAEFTFVVAKPNG.....
BCG67028.1   TQETAIGNFFSRAGLVSIITMPTTGTQNTDG.....YVNWDIDLMGYAQLRRKCELFTYMRFDAEFTFVVAKPNG.....
BCG67027.1   TQETAIGNFFSRAGLVSIITMPTTGTQNTDG.....YVNWDIDLMGYAQLRRKCELFTYMRFDAEFTFVVAKPNG.....
BCG66996.1   TQETAIGNFFSRAGLVSIITMPTTGTQNTDG.....YVNWDIDLMGYAQLRRKCELFTYMRFDAEFTFVVAKPNG.....
BCG67026.1   TQETAIGNFFSRAGLVSIITMPTTGTQNTDG.....YVNWDIDLMGYAQLRRKCELFTYMRFDAEFTFVVAKPNG.....
BCG67025.1   TQETAIGNFFSRAGLVSIITMPTTGTQNTDG.....YVNWDIDLMGYAQLRRKCELFTYMRFDAEFTFVVAKPNG.....
BCG67023.1   TQETAIGNFFSRAGLVSIITMPTTGTQNTDG.....YVNWDIDLMGYAQLRRKCELFTYMRFDAEFTFVVAKPNG.....
BCG67022.1   TQETAIGNFFSRAGLVSIITMPTTGTQNTDG.....YVNWDIDLMGYAQLRRKCELFTYMRFDAEFTFVVAKPNG.....
BCG67021.1   TQETAIGNFFSRAGLVSIITMPTTGTQNTDG.....YVNWDIDLMGYAQLRRKCELFTYMRFDAEFTFVVAKPNG.....
BCG67013.1   TQETAIGNFFSRAGLVSIITMPTTGTQNTDG.....YVNWDIDLMGYAQLRRKCELFTYMRFDAEFTFVVAKPNG.....
BCG67000.1   TQETAIGNFFSRAGLVSIITMPTTGTQNTDG.....YVNWDIDLMGYAQLRRKCELFTYMRFDAEFTFVVAKPNG.....
BCG66997.1   TQETAIGNFFSRAGLVSIITMPTTGTQNTDG.....YVNWDIDLMGYAQLRRKCELFTYMRFDAEFTFVVAKPNG.....
BCG66998.1   TQETAIGNFFSRAGLVSIITMPTTGTQNTDG.....YVNWDIDLMGYAQLRRKCELFTYMRFDAEFTFVVAKPNG.....
QWF08924.1   TQETAIGNFFSRAGLVSIITMPTTGTQNTDG.....YVNWDIDLMGYAQLRRKCELFTYMRFDAEFTFVVAKPNG.....
QIL87522.1   TQETAIGNFFSRAGLVSIITMPTTGTQNTDG.....YVNWDIDLMGYAQLRRKCELFTYMRFDAEFTFVVAKPNG.....
QYC65075.1   TQETAIGNFFSRAGLVSIITMPTTGTQNTDG.....YVNWDIDLMGYAQLRRKCELFTYMRFDAEFTFVVAKPNG.....
QWF08781.1   TQETAIGNFFSRAGLVSIITMPTTGTQNTDG.....YVNWDIDLMGYAQLRRKCELFTYMRFDAEFTFVVAKPNG.....
QWY15817.1   TQETAIGNFFSRAGLVSIITMPTTGTQNTDG.....YVNWDIDLMGYAQLRRKCELFTYMRFDAEFTFVVAKPNG.....
QWF08897.1   TQETAIGNFFSRAGLVSIITMPTTGTQNTDG.....YVNWDIDLMGYAQLRRKCELFTYMRFDAEFTFVVAKPNG.....
QWY15815.1   TQETAIGNFFSRAGLVSIITMPTTGTQNTDG.....YVNWDIDLMGYAQLRRKCELFTYMRFDAEFTFVVAKPNG.....
QWY15814.1   TQETAIGNFFSRAGLVSIITMPTTGTQNTDG.....YVNWDIDLMGYAQLRRKCELFTYMRFDAEFTFVVAKPNG.....
QWY15718.1   TQETAIGNFFSRAGLVSIITMPTTGTQNTDG.....YVNWDIDLMGYAQLRRKCELFTYMRFDAEFTFVVAKPNG.....
QYC65077.1   TQETAIGNFFSRAGLVSIITMPTTGTQNTDG.....YVNWDIDLMGYAQLRRKCELFTYMRFDAEFTFVVAKPNG.....
QTA73897.1   TQETAIGNFFSRAGLVSIITMPTTGTQNTDG.....YVNWDIDLMGYAQLRRKCELFTYMRFDAEFTFVVAKPNG.....
QWY15676.1   TQETAIGNFFSRAGLVSIITMPTTGTQNTDG.....YVNWDIDLMGYAQLRRKCELFTYMRFDAEFTFVVAKPNG.....
QOP74939.1   TQETAIGNFFSRAGLVSIIMMPTTGTQNTDG.....YVNWDIDLMGYAQLRRKCELFTYMRFDAEFTFVVAKPNG.....
QOP74936.1   TQETAIGNFFSRAGLVSIITMPTTGTQNTDG.....YVNWDIDLMGYAQLRRKCELFTYMRFDAEFTFVVAKPNG.....
QWF08729.1   TQETAIGNFFSRAGLVSIITMPTTGTQNTDG.....YVNWDIDLMGYAQLRRKCELFTYMRFDAEFTFVVAKPNG.....
QYC65064.1   TQETAIGNFFSRAGLVSIITMPTTGTQNTDG.....YVNWDIDLMGYAQLRRKCELFTYMRFDAEFTFVVAKPNG.....
QWF08775.1   TQETAIGNFFSRAGLVSIITMPTTGTQNTDG.....YVNWDIDLMGYAQLRRKCELFTYMRFDAEFTFVVAKPNG.....
QWF08774.1   TQETAIGNFFSRAGLVSIITMPTTGTQNTDG.....YVNWDIDLMGYAQLRRKCELFTYMRFDAEFTFVVAKPNG.....
QWF08753.1   TQETAIGNFFSRAGLVSIITMPTTGTQNTDG.....YVNWDIDLMGYAQLRRKCELFTYMRFDAEFTFVVAKPNG.....
QWY15804.1   TQETAIGNFFSRAGLVSIITMPTTGTQNTDG.....YVNWDIDLMGYAQLRRKCELFTYMRFDAEFTFVVAKPNG.....
QOP74943.1   TQETAIGNFFSRAGLVSIITMPTTGTQNTDG.....YVNWDIDLMGYAQLRRKCELFTYMRFDAEFTFVVAKPNG.....
QIH53920.1   TQETAIGNFFSRAGLVSIITMPTTGTQNTDG.....YVNWDIDLMGYAQLRRKCELFTYMRFDAEFTFVVAKPNG.....
QIH53919.1   TQETAIGNFFSRAGLVSIITMPTTGTQNTDG.....YVNWDIDLMGYAQLRRKCELFTYMRFDAEFTFVVAKPNG.....
QIL87536.1   TQETAIGNFFSRAGLVSIITMPTTGTQNTDG.....YVNWDIDLMGYAQLRRKCELFTYMRFDAEFTFVVAKPNG.....
UEC49854.1   TQETAIGNFFSRAGLVSIITMPTTGTQNTDG.....YVNWDIDLMGYAQLRRKCELFTYMRFDAEFTFVVAKPNG.....
UEC49852.1   TQETAIGNFFSRAGLVSIITMPTTGTQNTDG.....YVNWDIDLMGYAQLRRKCELFTYMRFDAEFTFVVAKPNG.....
QWY15841.1   TQETAIGNFFSRAGLVSIITMPTTGTQNTDG.....YVNWDIDLMGYAQLRRKCELFTYMRFDAEFTFVVAKPNG.....
QWY15787.1   TQETAIGNFFSRAGLVSIITMPTTGTQNTDG.....YVNWDIDLMGYAQLRRKCELFTYMRFDAEFTFVVAKPNG.....
QYC65128.1   TQETAIGNFFSRAGLVSIITMPTTGTQNTDG.....YVNWDIDLMGYAQLRRKCELFTYMRFDAEFTFVVAKPNG.....
QYC65116.1   TQETAIGNFFSRAGLVSIITMPTTGTQNTDG.....YVNWDIDLMGYAQLRRKCELFTYMRFDAEFTFVVAKPNG.....
QYC65083.1   TQETAIGNFFSRAGLVSIITMPTTGTQNTDG.....YVNWDIDLMGYAQLRRKCELFTYMRFDAEFTFVVAKPNG.....
QYC65029.1   TQETAIGNFFSRAGLVSIITMPTTGTQNTDG.....YVNWDIDLMGYAQLRRKCELFTYMRFDAEFTFVVAKPNG.....
QWF08951.1   TQETAIGNFFSRAGLVSIITMPTTGTQNTDG.....YVNWDIDLMGYAQLRRKCELFTYMRFDAEFTFVVAKPNG.....
QWF08937.1   TQETAIGNFFSRAGLVSIITMPTTGTQNTDG.....YVNWDIDLMGYAQLRRKCELFTYMRFDAEFTFVVAKPNG.....
QWF08918.1   TQETAIGNFFSRAGLVSIITMPTTGTQNTDG.....YVNWDIDLMGYAQLRRKCELFTYMRFDAEFTFVVAKPNG.....
QWF08901.1   TQETAIGNFFSRAGLVSIITMPTTGTQNTDG.....YVNWDIDLMGYAQLRRKCELFTYMRFDAEFTFVVAKPNG.....
QWF08834.1   TQETAIGNFFSRAGLVSIITMPTTGTQNTDG.....YVNWDIDLMGYAQLRRKCELFTYMRFDAEFTFVVAKPNG.....
QWF08832.1   TQETAIGNFFSRAGLVSIITMPTTGTQNTDG.....YVNWDIDLMGYAQLRRKCELFTYMRFDAEFTFVVAKPNG.....
QWF08802.1   TQETAIGNFFSRAGLVSIITMPTTGTQNTDG.....YVNWDIDLMGYAQLRRKCELFTYMRFDAEFTFVVAKPNG.....
QWF08800.1   TQETAIGNFFSRAGLVSIITMPTTGTQNTDG.....YVNWDIDLMGYAQLRRKCELFTYMRFDAEFTFVVAKPNG.....
QYC65123.1   TQETAIGNFFSRAGLVSIITMPTTGTQNTDG.....YVNWDIDLMGYAQLRRKCELFTYMRFDAEFTFVVAKPNG.....
QWY15820.1   TQETAIGNFFSRAGLVSIITMPTTGTQNTGG.....YVNWDIDLMGYAQLRRKCELFTYMRFDAEFTFVVAKPNG.....
QWY15864.1   TQETAIGNFFSRAGLVSIITMPTTGTQNTDG.....YVNWDIDLMGYAQLRRKCELFTYMRFDAEFTFVVAKPNG.....
QWY15840.1   TQETAIGNFFSRAGLVSIITMPTTGTQNTDG.....YVNWDIDLMGYAQLRRKCELFTYMRFDAEFTFVVAKPNG.....
QWY15786.1   TQETAIGNFFSRAGLVSIITMPTTGTQNTDG.....YVNWDIDLMGYAQLRRKCELFTYMRFDAEFTFVVAKPNG.....
QWY15842.1   TQETAIGNFFSRAGLVSIITMPTTGTQNTDG.....YVNWDIDLMGYAQLRRKCELFTYMRFDAEFTFVVAKPNG.....
QWY15704.1   TQETAIGNFFSRAGLVSIITMPTTGTQNTDG.....YVNWDIDLMGYAQLRRKCELFTYMRFDAEFTFVVAKPNG.....
QWY15750.1   TQETAIGNFFSRAGLVSIITMPTTGTQNTDG.....YVNWDIDLMGYAQLRRKCELFTYMRFDAEFTFVVAKPNG.....
QWF08733.1   TQETAIGNFFSRAGLVSIITMPTTGTQNTDG.....YVNWDIDLMGYAQLRRKCELFTYMRFDAEFTFVVAKPNG.....
QNL13228.1   TQETAIGNFFSRAGLVSIITMPTTGTQNTDG.....YVNWDIDLMGYAQLRRKCELFTYMRFDAEFTFVVAKPNG.....
QNL13216.1   TQETAIGNFFSRAGLVSIITMPTTGTQNTDG.....YVNWDIDLMGYAQLRRKCELFTYMRFDAEFTFVVAKPNG.....
QNL13226.1   TQETAIGNFFSRAGLVSIITMPTTGTQNTDG.....YVNWDIDLMGYAQLRRKCELFTYMRFDAEFTFVVAKPNG.....
QNL13203.1   TQETAIGNFFSRAGLVSIITMPTTGTQNTDG.....YVNWDIDLMGYAQLRRKCELFTYMRFDAEFTFVVAKPNG.....
```

```
QYC65080.1   TQETAIGNFFSRAGLVSIITMPTTGTQNTDG.....YVNWDIDLMGYAQLRRKCELFTYMRFDAEFTFVVAKPNG.....
QNL13225.1   TQETAIGNFFSRAGLVSIITMPTTGTQNTDG.....YVNWDIDLMGYAQLRRKCELFTYMRFDAEFTFVVAKPNG.....
QNL13224.1   TQETAIGNFFSRAGLVSIITMPTTGTQNTDG.....YVNWDIDLMGYAQLRRKCELFTYMRFDAEFTFVVAKPNG.....
QNL13223.1   TQETAIGNFFSRAGLVSIITMPTTGTQNTDG.....YVNWDIDLMGYAQLRRKCELFTYMRFDAEFTFVVAKPNG.....
QNL13222.1   TQETAIGNFFSRAGLVSIITMPTTGTQNTDG.....YVNWDIDLMGYAQLRRKCELFTYMRFDAEFTFVVAKPNG.....
QNL13221.1   TQETAIGNFFSRAGLVSIITMPTTGTQNTDG.....YVNWDIDLMGYAQLRRKCELFTYMRFDAEFTFVVAKPNG.....
QNL13220.1   TQETAIGNFFSRAGLVSIITMPTTGTQNTDG.....YVNWDIDLMGYAQLRRKCELFTYMRFDAEFTFVVAKPNG.....
QNL13219.1   TQETAIGNFFSRAGLVSIITMPTTGTQNTDG.....YVNWDIDLMGYAQLRRKCELFTYMRFDAEFTFVVAKPNG.....
QWF08894.1   TQETAIGNFFSRAGLVSIITMPTTGTQNTDG.....YVNWDIDLMGYAQLRRKCELFTYMRFDAEFTFVVAKPNG.....
QNL13218.1   TQETAIGNFFSRAGLVSIITMPTTGTQNTDG.....YVNWDIDLMGYAQLRRKCELFTYMRFDAEFTFVVAKPNG.....
QNL13217.1   TQETAIGNFFSRAGLVSIITMPTTGTQNTDG.....YVNWDIDLMGYAQLRRKCELFTYMRFDAEFTFVVAKPNG.....
QNL13215.1   TQETAIGNFFSRAGLVSIITMPTTGTQNTDG.....YVNWDIDLMGYAQLRRKCELFTYMRFDAEFTFVVAKPNG.....
QNL13214.1   TQETAIGNFFSRAGLVSIITMPTTGTQNTDG.....YVNWDIDLMGYAQLRRKCELFTYMRFDAEFTFVVAKPNG.....
QNL13213.1   TQETAIGNFFSRAGLVSIITMPTTGTQNTDG.....YVNWDIDLMGYAQLRRKCELFTYMRFDAEFTFVVAKPNG.....
QNL13212.1   TQETAIGNFFSRAGLVSIITMPTTGTQNTDG.....YVNWDIDLMGYAQLRRKCELFTYMRFDAEFTFVVAKPNG.....
QNL13209.1   TQETAIGNFFSRAGLVSIITMPTTGTQNTDG.....YVNWDIDLMGYAQLRRKCELFTYMRFDAEFTFVVAKPNG.....
QIL87537.1   TQETAIGNFFSRAGLVSIITMPTTGTQNTDG.....YVNWDIDLMGYAQLRRKCELFTYMRFDAEFTFVVAKPNG.....
QNL13208.1   TQETAIGNFFSRAGLVSIITMPTTGTQNTDG.....YVNWDIDLMGYAQLRRKCELFTYMRFDAEFTFVVAKPNG.....
QNL13207.1   TQETAIGNFFSRAGLVSIITMPTTGTQNTDG.....YVNWDIDLMGYAQLRRKCELFTYMRFDAEFTFVVAKPNG.....
QNL13206.1   TQETAIGNFFSRAGLVSIITMPTTGTQNTDG.....YVNWDIDLMGYAQLRRKCELFTYMRFDAEFTFVVAKPNG.....
QNL13202.1   TQETAIGNFFSRAGLVSIITMPTTGTQNTDG.....YVNWDIDLMGYAQLRRKCELFTYMRFDAEFTFVVAKPNG.....
QNL13196.1   TQETAIGNFFSRAGLVSIITMPTTGTQNTDG.....YVNWDIDLMGYAQLRRKCELFTYMRFDAEFTFVVAKPNG.....
QNL13195.1   TQETAIGNFFSRAGLVSIITMPTTGTQNTDG.....YVNWDIDLMGYAQLRRKCELFTYMRFDAEFTFVVAKPNG.....
QNL13194.1   TQETAIGNFFSRAGLVSIITMPTTGTQNTDG.....YVNWDIDLMGYAQLRRKCELFTYMRFDAEFTFVVAKPNG.....
QNL13193.1   TQETAIGNFFSRAGLVSIITMPTTGTQNTDG.....YVNWDIDLMGYAQLRRKCELFTYMRFDAEFTFVVAKPNG.....
QNL13192.1   TQETAIGNFFSRAGLVSIITMPTTGTQNTDG.....YVNWDIDLMGYAQLRRKCELFTYMRFDAEFTFVVAKPNG.....
QNL13191.1   TQETAIGNFFSRAGLVSIITMPTTGTQNTDG.....YVNWDIDLMGYAQLRRKCELFTYMRFDAEFTFVVAKPNG.....
QNL13190.1   TQETAIGNFFSRAGLVSIITMPTTGTQNTDG.....YVNWDIDLMGYAQLRRKCELFTYMRFDAEFTFVVAKPNG.....
QNL13189.1   TQETAIGNFFSRAGLVSIITMPTTGTQNTDG.....YVNWDIDLMGYAQLRRKCELFTYMRFDAEFTFVVAKPNG.....
QNL13188.1   TQETAIGNFFSRAGLVSIITMPTTGTQNTDG.....YVNWDIDLMGYAQLRRKCELFTYMRFDAEFTFVVAKPNG.....
QCY53846.1   TQETAIGNFFSRAGLVSIITMPTTGTQNTDG.....YVNWDIDLMGYAQLRRKCELFTYMRFDAEFTFVVAKPNG.....
BCI65357.1   TQETAIGNFFSRAGLVSIITMPTTGTQNTDG.....YVNWDIDLMGYAQLRRKCELFTYMRFDAEFTFVVAKPNG.....
QNL13227.1   TQETAFGNFFSRAGLVSIITMPTTGTQNTDG.....YVNWDIDLMGYAQLRRKCELFTYMRFDAEFTFVVAKPNG.....
QNL13211.1   TQETAIGNFFSRAGLVSIITMPTTGTQNTDG.....YVNWNIDLMGYAQLRRKCELFTYMRFDAEFTFVVAKPNG.....
QNL13198.1   TQETAIGNFFSRAGLVSIITMPTTGTQNTDG.....YVNWDIDLMGYAQLRRKCELFTYMRFDAEFTFVVAKPNG.....
QNL13197.1   TQETAIGNFFSRAGLVSIITMPTTGTQNTDG.....YVNWDIDLMGYAQLRRKCELFTYMRFDAEFTFVVAKPNG.....
QNL13200.1   TQETAIGNFFSRAGLVSIITMPTTGTQNTDG.....YVNWDIDLMGYAQLRRKCELFTYMRFDAEFTFVVAKPNG.....
QNL13210.1   TQETAIGNFFSRAGLVSIITMPTTGTQNTDG.....YVNWDIDLMGYAQLRRKCELFTYMRFDAEFTFVVAKPNG.....
QNL13205.1   TQETAIGNFFSRAGLVSIITMPTTGTQNTDG.....YVNWDIDLMGYAQLRRKCELFTYMRFDAEFTFVVAKPNG.....
QNL13204.1   TQETAIGNFFSRAGLVSIITMPTTGTQNTDG.....YVNWDIDLMGYAQLRRKCELFTYMRFDAEFTFVVAKPNG.....
QNL13201.1   TQETAIGNFFSRAGLVSIITMPTTGTQNTDG.....YVNWDIDLMGYAQLRRKCELFTYMRFDAEFTFVVAKPNG.....
QNL13199.1   TQETAIGNFFSRAGLVSIITMPTTGTQNTDG.....YVNWDIDLMGYAQLRRKCELFTYMRFDAEFTFVVAKPNG.....
QYC65157.1   TQETAIGNFFSRAGLVSIITMPTTGTQNADG.....YVNWDIDLMGYAQLRRKCELFTYMRFDAEFTFVVAKPNG.....
QYC65138.1   TQETAIGNFFSRAGLVSIITMPTTGTQNADG.....YVNWDIDLMGYAQLRRKCELFTYMRFDAEFTFVVAKPNG.....
QWF08724.1   TQETAIGNFFSRAGLVSIITMPTTGTQNADG.....YVNWDIDLMGYAQLRRKCELFTYMRFDAEFTFVVAKPNG.....
UEP53403.1   TQETAIGNFFSRAGLVSIITMPTTGTQNADG.....YVNWDIDLMGYAQLRRKCELFTYMRFDAEFTFVVAKPNG.....
QWY15843.1   TQETAIGNFFSRAGLVSIITMPTMGTQNADG.....YVNWDIDLMGYAQLRRKCELFTYMRFDAEFTFVVAKPNG.....
QWF08794.1   TQETAIGNFFSRAGLVSIITMPTTGTQNADG.....YVNWDIDLMGYAQLRRKCELFTYMRFDAEFTFVVAKPNG.....
QYC65031.1   TQETAIGNFFSRAGLVSIITMPTTGTQNADG.....YVNWDIDLMGYAQLRRKCELFTYMRFDAEFTFVVAKPNG.....
UEC49856.1   TQETAIGNFFSRAGLVSIITMPTTGTQNTDG.....YVNWDIDLMGYAQLRRKCELFTYMRFDAEFTFVVAKPNG.....
BBH72184.1   TQETAIGNFFSRAGLVSIITMPTTGTQNTDG.....YVNWDIDLMGYAQLRRKCELFTYMRFDAEFTFVVAKPNG.....
QWF08874.1   TQETAIGNFFSRAGLVSIITMPTTGTQNTDG.....YVNWDIDLMGYAQLRRKCELFTYMRFDAEFTFVVAKPNG.....
QWF08873.1   TQETAIGNFFSRAGLVSIITMPTTGTQNTDG.....YVNWDIDLMGYAQLRRKCELFTYMRFDAEFTFVVAKPNG.....
BCG67003.1   TQETAIGNFFSRAGLVSIITMPTTGTQNTDG.....YVNWDIDLMGYAQLRRKCELFTYMRFDAEFTFVVAKPNG.....
QYC65095.1   TQETAIGNFFSRAGLVSIITMPTTGTQNTDG.....YVNWDIDLMGYAQLRRKCELFTYMRFDAEFTFVVAKPNG.....
QOP74933.1   TQETAIGNFFSRAGLVSIITMPTTGTQNTDG.....YVNWDIDLMGYAQLRRKCELFTYMRFDAEFTFVVAKPNG.....
QYC65096.1   TQETAIGNFFSRAGLVSIITMPTTGTQNTDG.....YVNWDIDLMGYAQLRRKCELFTYMRFDAEFTFVVAKPNG.....
BCG67034.1   TQETAIGNFFSRAGLVSIITMPTTGTQNTDG.....YVNWDIDLMGYAQLRRKCELFTYMRFDAEFTFVVAKPNG.....
BCG67031.1   TQETAIGNFFSRAGLVSIITMPTTGTQNTDG.....YVNWDIDLMGYAQLRRKCELFTYMRFDAEFTFVVAKPNG.....
QYC65052.1   TQETAIGNFFSRAGLVSIITMPTTGTQNTDG.....YVNWDIDLMGYAQLRRKCELFTYMRFDAEFTFVVAKPNG.....
QWF08963.1   TQETAIGNFFSRAGLVSIITMPTTGTQNTDG.....YVNWDIDLMGYAQLRRKCELFTYMRFDAEFTFVVAKPNG.....
QWF08939.1   TQETAIGNFFSRAGLVSIITMPTTGTQNTDG.....YVNWDIDLMGYAQLRRKCELFTYMRFDAEFTFVVAKPNG.....
QIL87538.1   TQETAIGNFFSRAGLVSIITMPTTGTQNTDG.....YVNWDIDLMGYAQLRRKCELFTYMRFDAEFTFVVAKPNG.....
QIL87535.1   TQETAIGNFFSRAGLVSIITMPTTGTQNTDG.....YVNWDIDLMGYAQLRRKCELFTYMRFDAEFTFVVAKPNG.....
QWF08964.1   TQETAIGNFFSRAGLVSIITMPTTGTQNTDG.....YVNWNIDLMGYAQLRRKCELFTYMRFDAEFTFVVAKPNG.....
QYC65132.1   TQETAIGNFFSRAGLVSIITMPTTGTQNTDG.....YVNWDIDLMGYAQLRRKCELFTYMRFDAEFTFVVAKPNG.....
QTA73893.1   TQETAIGNFFSRAGLVSIITMPTTGTQNTDG.....YVNWDIDLMGYAQLRRKCELFTYMRFDAEFTFVVAKPNG.....
QWF08751.1   TQETAIGNFFSRAGLVSIITMPTTGTQNTDG.....YVNWDIDLMGYAQLRRKCELFTYMRFDAEFTFVVAKPNG.....
QWY15860.1   TQETAIGNFFSRAGLVSIITMPTTGTQNTDG.....YVNWDIDLMGYAQLRRKCELFTYMRFDAEFTFVVAKPNG.....
QWY15749.1   TQETAIGNFFSRAGLVSIITMPTTGTQNTDG.....YVNWDIDLMGYAQLRRKCELFTYMRFDAEFTFVVAKPNG.....
QWY15744.1   TQETAIGNFFSRAGLVSIITMPTTGTQNTDG.....YVNWDIDLMGYAQLRRKCELFTYMRFDAEFTFVVAKPNG.....
QWY15740.1   TQETAIGNFFSRAGLVSIITMPTTGTQNTDG.....YVNWDIDLMGYAQLRRKCELFTYMRFDAEFTFVVAKPNG.....
QWY15700.1   TQETAIGNFFSRAGLVSIITMPTTGTQNTDG.....YVNWDIDLMGYAQLRRKCELFTYMRFDAEFTFVVAKPNG.....
QWY15698.1   TQETAIGNFFSRAGLVSIITMPTTGTQNTDG.....YVNWDIDLMGYAQLRRKCELFTYMRFDAEFTFVVAKPNG.....
QWY15672.1   TQETAIGNFFSRAGLVSIITMPTTGTQNTDG.....YVNWDIDLMGYAQLRRKCELFTYMRFDAEFTFVVAKPNG.....
pdb|6LHT|B   TQETAIGNFFSRAGLVSIITMPTTDTQNTDG.....YVNWDIDLMGYAQLRRKCELFTYMRFDAEFTFVVAKPNG.....
pdb|6LHT|E   TQETAIGNFFSRAGLVSIITMPTTDTQNTDG.....YVNWDIDLMGYAQLRRKCELFTYMRFDAEFTFVVAKPNG.....
pdb|6LHT|D   TQETAIGNFFSRAGLVSIITMPTTDTQNTDG.....YVNWDIDLMGYAQLRRKCELFTYMRFDAEFTFVVAKPNG.....
pdb|6LHT|C   TQETAIGNFFSRAGLVSIITMPTTDTQNTDG.....YVNWDIDLMGYAQLRRKCELFTYMRFDAEFTFVVAKPNG.....
pdb|6LHT|A   TQETAIGNFFSRAGLVSIITMPTTDTQNTDG.....YVNWDIDLMGYAQLRRKCELFTYMRFDAEFTFVVAKPNG.....
pdb|6LHQ|A   TQETAIGNFFSRAGLVSIITMPTTDTQNTDG.....YVNWDIDLMGYAQLRRKCELFTYMRFDAEFTFVVAKPNG.....
pdb|6LHP|A   TQETAIGNFFSRAGLVSIITMPTTDTQNTDG.....YVNWDIDLMGYAQLRRKCELFTYMRFDAEFTFVVAKPNG.....
pdb|6LHO|A   TQETAIGNFFSRAGLVSIITMPTTDTQNTDG.....YVNWDIDLMGYAQLRRKCELFTYMRFDAEFTFVVAKPNG.....
pdb|6LHL|A   TQETAIGNFFSRAGLVSIITMPTTDTQNTDG.....YVNWDIDLMGYAQLRRKCELFTYMRFDAEFTFVVAKPNG.....
pdb|6LHK|A   TQETAIGNFFSRAGLVSIITMPTTDTQNTDG.....YVNWDIDLMGYAQLRRKCELFTYMRFDAEFTFVVAKPNG.....
pdb|6LHC|A   TQETAIGNFFSRAGLVSIITMPTTDTQNTDG.....YVNWDIDLMGYAQLRRKCELFTYMRFDAEFTFVVAKPNG.....
pdb|6LHB|A   TQETAIGNFFSRAGLVSIITMPTTDTQNTDG.....YVNWDIDLMGYAQLRRKCELFTYMRFDAEFTFVVAKPNG.....
pdb|6LHA|A   TQETAIGNFFSRAGLVSIITMPTTDTQNTDG.....YVNWDIDLMGYAQLRRKCELFTYMRFDAEFTFVVAKPNG.....
QWY15708.1   TQETAIGNFFSRAGLVSIITMPTTGTQNTDG.....YVNWDIDLMGYAQLRRKCELFTYMRFDAEFTFVVAKPNG.....
BBH72101.1   TQETAIGNFFSRAGLVSIITMPTTGTQNTDG.....YVNWDIDLMGYAQLRRKCELFTYMRFDAEFTFVVAKPNG.....
BBH72090.1   TQETAIGNFFSRAGLVSIITMPTTGTQNTDG.....YVNWDIDLMGYAQLRRKCELFTYMRFDAEFTFVVAKPNG.....
BBH72097.1   TQETAIGNFFSRAGLVSIITMPTTGTQNTDG.....YVNWDIDLMGYAQLRRKCELFTYMRFDAEFTFVVAKPNG.....
BBH72089.1   TQETAIGNFFSRAGLVSIITMPTTGTQNTDG.....YVNWDIDLMGYAQLRRKCELFTYMRFDAEFTFVVAKPNG.....
BBH72099.1   TQETAIGNFFSRAGLVSIITMPTTGTQNTDG.....YVNWDIDLMGYAQLRRKCELFTYMRFDAEFTFVVAKPNG.....
BBH72093.1   TQETAIGNFFSRAGLVSIITMPTTGTQNTDG.....YVNWDIDLMGYAQLRRKCELFTYMRFDAEFTFVVAKPNG.....
BBH72096.1   TQETAIGNFFSRAGLVSIITMPTTGTQNTDG.....YVNWDIDLMGYAQLRRKCELFTYMRFDAEFTFVVAKPNG.....
BBH72094.1   TQETAIGNFFSRAGLVSIITMPTTGTQNTDG.....YVNWDIDLMGYAQLRRKCELFTYMRFDAEFTFVVAKPNG.....
BBH72091.1   TQETAIGNFFSRAGLVSIITMPTTGTQNTDG.....YVNWDIDLMGYAQLRRKCELFTYMRFDAEFTFVVAKPNG.....
QOP74916.1   TQETAIGNFFSRAGLVSIITMPTTGTQNTDG.....YVNWDIDLMGYAQLRRKCELFTYMRFDAEFTFVVAKPNG.....
QYC65163.1   TQETAIGNFFSRAGLVSIITMPTTGTQNTDG.....YVNWDIDLMGYAQLRRKCELFTYMRFDAEFTFVVAKPNG.....
QWF08736.1   TQETAIGNFFSRAGLVSIITMPTTGTQNTDG.....YVNWDIDLMGYAQLRRKCELFTYMRFDAEFTFVVAKPNG.....
QWF08735.1   TQETAIGNFFSRAGLVSIITMPTTGTQNTDG.....YVNWDIDLMGYAQLRRKCELFTYMRFDAEFTFVVAKPNG.....
pdb|5TSK|A   TQETAIGNFFSRAGLVSIITMPTTGTQNTDG.....YVNWDIDLMGYAQLRRKCELFTYMRFDAEFTFVVAKPNG.....
pdb|5C9A|A   TQETAIGNFFSRAGLVSIITMPTTGTQNTDG.....YVNWDIDLMGYAQLRRKCELFTYMRFDAEFTFVVAKPNG.....
pdb|5C4W|A   TQETAIGNFFSRAGLVSIITMPTTGTQNTDG.....YVNWDIDLMGYAQLRRKCELFTYMRFDAEFTFVVAKPNG.....
pdb|4JGZ|A   TQETAIGNFFSRAGLVSIITMPTTGTQNTDG.....YVNWDIDLMGYAQLRRKCELFTYMRFDAEFTFVVAKPNG.....
pdb|4JGY|A   TQETAIGNFFSRAGLVSIITMPTTGTQNTDG.....YVNWDIDLMGYAQLRRKCELFTYMRFDAEFTFVVAKPNG.....
QWY15956.1   TQETAIGNFFSRAGLVSIITMPTTGTQNTDG.....YVNWDIDLMGYAQLRRKCELFTYMRFDAEFTFVVAKPNG.....
QWF08811.1   TQETAIGNFFSRAGLVSIITMPTTGTQNTDG.....YVNWDIDLMGYAQLRRKCELFTYMRFDAEFTFVVAKPNG.....
QWF08878.1   TQETAIGNFFSRAGLVSIITMPTTGTQNTDG.....YVNWDIDLMGYAQLRRKCELFTYMRFDAEFTFVVAKPNG.....
QWF08869.1   TQETAIGNFFSRAGLVSIITMPTTGTQNTDG.....YVNWDIDLMGYAQLRRKCELFTYMRFDAEFTFVVAKPNG.....
QWF08831.1   TQETAIGNFFSRAGLVSIITMPTTGTQNTDG.....YVNWDIDLMGYAQLRRKCELFTYMRFDAEFTFVVAKPNG.....
QWF08829.1   TQETAIGNFFSRAGLVSIITMPTTGTQNTDG.....YVNWDIDLMGYAQLRRKCELFTYMRFDAEFTFVVAKPNG.....
QWF08824.1   TQETAIGNFFSRAGLVSIITMPTTGTQNTDG.....YVNWDIDLMGYAQLRRKCELFTYMRFDAEFTFVVAKPNG.....
QWF08822.1   TQETAIGNFFSRAGLVSIITMPTTGTQNTDG.....YVNWDIDLMGYAQLRRKCELFTYMRFDAEFTFVVAKPNG.....
QWF08817.1   TQETAIGNFFSRAGLVSIITMPTTGTQNTDG.....YVNWDIDLMGYAQLRRKCELFTYMRFDAEFTFVVAKPNG.....
QWF08816.1   TQETAIGNFFSRAGLVSIITMPTTGTQNTDG.....YVNWDIDLMGYAQLRRKCELFTYMRFDAEFTFVVAKPNG.....
QWF08798.1   TQETAIGNFFSRAGLVSIITMPTTGTQNTDG.....YVNWDIDLMGYAQLRRKCELFTYMRFDAEFTFVVAKPNG.....
QWF08779.1   TQETAIGNFFSRAGLVSIITMPTTGTQNTDG.....YVNWDIDLMGYAQLRRKCELFTYMRFDAEFTFVVAKPNG.....
QWF08778.1   TQETAIGNFFSRAGLVSIITMPTTGTQNTDG.....YVNWDIDLMGYAQLRRKCELFTYMRFDAEFTFVVAKPNG.....
QWF08772.1   TQETAIGNFFSRAGLVSIITMPTTGTQNTDG.....YVNWDIDLMGYAQLRRKCELFTYMRFDAEFTFVVAKPNG.....
QWF08771.1   TQETAIGNFFSRAGLVSIITMPTTGTQNTDG.....YVNWDIDLMGYAQLRRKCELFTYMRFDAEFTFVVAKPNG.....
QWF08745.1   TQETAIGNFFSRAGLVSIITMPTTGTQNTDG.....YVNWDIDLMGYAQLRRKCELFTYMRFDAEFTFVVAKPNG.....
QWF08744.1   TQETAIGNFFSRAGLVSIITMPTTGTQNTDG.....YVNWDIDLMGYAQLRRKCELFTYMRFDAEFTFVVAKPNG.....
QIH53922.1   TQETAIGNFFSRAGLVSIITMPTTGTQNTDG.....YVNWDIDLMGYAQLRRKCELFTYMRFDAEFTFVVAKPNG.....
QOP74941.1   TQETAIGNFFSRAGLVSIITMPTTGTQNTDG.....YVNWDIDLMGYAQLRRKCELFTYMRFDAEFTFVVAKPNG.....
QWY15812.1   TQETAIGNFFSRAGLVSIITMPTTGTQNTDG.....YVNWDIDLMGYAQLRRKCELFTYMRFDAEFTFVVAKPNG.....
QOP74926.1   TQETAIGNFFSRAGLVSIITMPTTGTQNTDG.....YVNWDIDLMGYAQLRRKCELFTYMRFDAEFTFVVAKPNG.....
QYC65176.1   TQETAIGNFFSRAGLVSIITMPTTGTQNTDG.....YVNWDIDLMGYAQLRRKCELFTYMRFDAEFTFVVAKPNG.....
QYC65172.1   TQETAIGNFFSRAGLVSIITMPTTGTQNTDG.....YVNWDIDLMGYAQLRRKCELFTYMRFDAEFTFVVAKPNG.....
QYC65168.1   TQETAIGNFFSRAGLVSIITMPTTGTQNTDG.....YVNWDIDLMGYAQLRRKCELFTYMRFDAEFTFVVAKPNG.....
QWF08725.1   TQETAIGNFFSRAGLVSIITMPTTGTQNTDG.....YVNWDIDLMGYAQLRRKCELFTYMRFDAEFTFVVAKPNG.....
QYC65028.1   TQETAIGNFFSRAGLVSIITMPTTGTQNTDG.....YVNWDIDLMGYAQLRRKCELFTYMRFDAEFTFVVAKPNG.....
QWY15686.1   TQETAIGNFFSRAGLVSIITMPTTGTQNTDG.....YVNWDIDLMGYAQLRRKCELFTYMRFDAEFTFVVAKPNG.....
QYC65023.1   TQETAIGNFFSRAGLVSIITMPTTGTQNTDG.....YVNWDIDLMGYAQLRRKCELFTYMRFDAEFTFVVAKPNG.....
QYC65025.1   TQETAIGNFFSRAGLVSIITMPTTGTQNTDG.....YVNWDIDLMGYAQLRRKCELFTYMRFDAEFTFVVAKPNG.....
QWY15946.1   TQETAIGNFFSRAGLVSIITMPTTGTQNTDG.....YVNWDIDLMGYAQLRRKCELFTYMRFDAEFTFVVAKPNG.....
BCB92164.1   TQETAIGNFFSRAGLVSIITMPTTGTQNTDG.....YVNWDIDLMGYAQLRRKCELFTYMRFDAEFTFVVAKPNG.....
QOP74935.1   TQETAIGNFFSRAGLVSIITMPTTGTQNTDG.....YVNWDIDLMGYAQLRRKCELFTYMRFDAEFTFVVAKPNG.....
QOP74930.1   TQETAIGNFFSRAGLVSIITMPTTGTQNTDG.....YVNWDIDLMGYAQLRRKCELFTYMRFDAEFTFVVAKPNG.....
QOP74928.1   TQETAIGNFFSRAGLVSIITMPTTGTQNTDG.....YVNWDIDLMGYAQLRRKCELFTYMRFDAEFTFVVAKPNG.....
QOP74927.1   TQETAIGNFFSRAGLVSIITMPTTGTQNTDG.....YVNWDIDLMGYAQLRRKCELFTYMRFDAEFTFVVAKPNG.....
QOP74925.1   TQETAIGNFFSRAGLVSIITMPTTGTQNTDG.....YVNWDIDLMGYAQLRRKCELFTYMRFDAEFTFVVAKPNG.....
QOP74923.1   TQETAIGNFFSRAGLVSIITMPTTGTQNTDG.....YVNWDIDLMGYAQLRRKCELFTYMRFDAEFTFVVAKPNG.....
```

```
QOP74922.1    TQETAIGNFFSRAGLVSIITMPTTGTQNTDG.....YVNWDIDLMGYAQLRRKCELFTYMRFDAEFTFVVAKPNG.....
QOP74920.1    TQETAIGNFFSRAGLVSIITMPTTGTQNTDG.....YVNWDIDLMGYAQLRRKCELFTYMRFDAEFTFVVAKPNG.....
QOP74919.1    TQETAIGNFFSRAGLVSIITMPTTGTQNTDG.....YVNWDIDLMGYAQLRRKCELFTYMRFDAEFTFVVAKPNG.....
QOP74917.1    TQETAIGNFFSRAGLVSIITMPTTGTQNTDG.....YVNWDIDLMGYAQLRRKCELFTYMRFDAEFTFVVAKPNG.....
QHB49260.1    TQETAIGNFFSRAGLVSIITMPTTGTQNTDG.....YVNWDIDLMGYAQLRRKCELFTYMRFDAEFTFVVAKPNG.....
QWF08784.1    TQETAIGNFFSRAGLVSIITMPTTGTQNTDG.....YVNWDIDLMGYAQLRRKCELFTYMRFDAEFTFVVAKPNG.....
QWF08782.1    TQETAIGNFFSRAGLVSIITMPTTGTQNTDG.....YVNWDIDLMGYAQLRRKCELFTYMRFDAEFTFVVAKPNG.....
QWF08777.1    TQETAIGNFFSRAGLVSIITMPTTGTQNTDG.....YVNWDIDLMGYAQLRRKCELFTYMRFDAEFTFVVAKPNG.....
QWF08757.1    TQETAIGNFFSRAGLVSIITMPTTGTQNTDG.....YVNWDIDLMGYAQLRRKCELFTYMRFDAEFTFVVAKPNG.....
QWF08756.1    TQETAIGNFFSRAGLVSIITMPTTGTQNTDG.....YVNWDIDLMGYAQLRRKCELFTYMRFDAEFTFVVAKPNG.....
QWF08755.1    TQETAIGNFFSRAGLVSIITMPTTGTQNTDG.....YVNWDIDLMGYAQLRRKCELFTYMRFDAEFTFVVAKPNG.....
QWF08791.1    TQETAIGNFFSRAGLVSIITMPTTGTQNTDG.....YVNWDIDLMGYAQLRRKCELFTYMRFDAEFTFVVAKPNG.....
QWF08786.1    TQDTAIGNFFSRAGLVSIITMPTTGTQNTDG.....YVNWDIDLMGYAQLRRKCELFTYMRFDAEFTFVVAKPNG.....
QYC65186.1    TQETAIGNFFSRAGLVSIITMPTTGTQNTDG.....YVNWDIDLMGYAQLRRKCELFTYMRFDAEFTFVVAKPNG.....
QWY15722.1    TQETAIGNFFSRAGLVSIITMPTTGTQNTDG.....YVNWDIDLMGYAQLRRKCELFTYMRFDAEFTFVVAKPNG.....
QYC65182.1    TQETAIGNFFSRAGLVSIITMPTTGTQNTDG.....YVNWDIDLMGYAQLRRKCELFTYMRFDAEFTFVVAKPNG.....
QWF08754.1    TQETAIGNFFSRAGLVSIITMPTTGTQNTDG.....YVNWDIDLMGYAQLRRKCELFTYMRFDAEFTFVVAKPNG.....
QWF08748.1    TQETAIGNFFSRAGLVSIITMPTTGTQNTDG.....YVNWDIDLMGYAQLRRKCELFTYMRFDAEFTFVVAKPNG.....
QYC65181.1    TQETAIGNFFSRAGLVSIITMPTTGTQNTDG.....YVNWDIDLMGYAQLRRKCELFTYMRFDAEFTFVVAKPNG.....
QWY15719.1    TQETAIGNFFSRAGLVSIITMPTTGTQNTDG.....YVNWDIDLMGYAQLRRKCELFTYMRFDAEFTFVVAKPNG.....
QYC65180.1    TQETAIGNFFSRAGLVSIITMPTTGTQNTDG.....YVNWDIDLMGYAQLRRKCELFTYMRFDAEFTFVVAKPNG.....
QWY15693.1    TQETAIGNFFSRAGLVSIITMPTTGTQNTDG.....YVNWDIDLMGYAQLRRKCELFTYMRFDAEFTFVVAKPNG.....
QYC65179.1    TQETAIGNFFSRAGLVSIITMPTTGTQNTDG.....YVNWDIDLMGYAQLRRKCELFTYMRFDAEFTFVVAKPNG.....
BCB92156.1    TQETAIGNFFSRAGLVSIITMPTTGTQNTDG.....YVNWDIDLMGYAQLRRKCELFTYMRFDAEFTFVVAKPNG.....
QYC65178.1    TQETAIGNFFSRAGLVSIITMPTTGTQNTDG.....YVNWDIDLMGYAQLRRKCELFTYMRFDAEFTFVVAKPNG.....
pdb|5C8C|A    TQETAIGNFFSRAGLVSIITMPTTGTQNTDG.....YVNWDIDLMGYAQLRRKCELFTYMRFDAEFTFVVAKPNG.....
QWF08864.1    TQETAIGNFFSRAGLVSIITMPTTGTQNTDG.....YVNWDIDLMGYAQLRRKCELFTYMRFDAEFTFVVAKPNG.....
QWF08747.1    TQETAIGNFFSRAGLVSIITMPTTGTQNTDG.....YVNWDIDLMGYAQLRRKCELFTYMRFDAEFTFVVAKPNG.....
QWF08743.1    TQETAIGNFFSRAGLVSIITMPTTGTQNTDG.....YVNWDIDLMGYAQLRRKCELFTYMRFDAEFTFVVAKPNG.....
QWF08741.1    TQETAIGNFFSRAGLVSIITMPTTGTQNTDG.....YVNWDIDLMGYAQLRRKCELFTYMRFDAEFTFVVAKPNG.....
QWF08737.1    TQETAIGNFFSRAGLVSIITMPTTGTQNTDG.....YVNWDIDLMGYAQLRRKCELFTYMRFDAEFTFVVAKPNG.....
QWF08734.1    TQETAIGNFFSRAGLVSIITMPTTGTQNTDG.....YVNWDIDLMGYAQLRRKCELFTYMRFDAEFTFVVAKPNG.....
BBH72119.1    TQETAIGNFFSRAGLVSIITMPTTGTQNTDG.....YVNWDIDLMGYAQLRRKCELFTYMRFDAEFTFVVAKPNG.....
QYC65177.1    TQETAIGNFFSRAGLVSIITMPTTGTQNTDG.....YVNWDIDLMGYAQLRRKCELFTYMRFDAEFTFVVAKPNG.....
QWF08732.1    TQETAIGNFFSRAGLVSIITMPTTGTQNTDG.....YVNWDIDLMGYAQLRRKCELFTYMRFDAEFTFVVAKPNG.....
QWF08730.1    TQETAIGNFFSRAGLVSIITMPTTGTQNTDG.....YVNWDIDLMGYAQLRRKCELFTYMRFDAEFTFVVAKPNG.....
QYC65175.1    TQETAIGNFFSRAGLVSIITMPTTGTQNTDG.....YVNWDIDLMGYAQLRRKCELFTYMRFDAEFTFVVAKPNG.....
QWF08727.1    TQETAIGNFFSRAGLVSIITMPTTGTQNTDG.....YVNWDIDLMGYAQLRRKCELFTYMRFDAEFTFVVAKPNG.....
QYC65173.1    TQETAIGNFFSRAGLVSIITMPTTGTQNTDG.....YVNWDIDLMGYAQLRRKCELFTYMRFDAEFTFVVAKPNG.....
BBH72105.1    TQETAIGNFFSRAGLVSIITMPTTGTQNTDG.....YVNWDIDLMGYAQLRRKCELFTYMRFDAEFTFVVAKPNG.....
QYC65171.1    TQETAIGNFFSRAGLVSIITMPTTGTQNTDG.....YVNWDIDLMGYAQLRRKCELFTYMRFDAEFTFVVAKPNG.....
pdb|5ABJ|A    TQETAIGNFFSRAGLVSIITMPTTGTQNTDG.....YVNWDIDLMGYAQLRRKCELFTYMRFDAEFTFVVAKPNG.....
QWY15716.1    TQETAIGNFFSRAGLVSIITMPTTGTQNTDG.....YVNWDIDLMGYAQLRRKCELFTYMRFDAEFTFVVAKPNG.....
QWY15800.1    TQETAIGNFFSRAGLVSIITMPTTGTQNTDG.....YVNWDIDLMGYAQLRRKCELFTYMRFDAEFTFVVAKPNG.....
QYC65018.1    TQETAIGNFFSRAGLVSIITMPTTGTQNTDG.....YVNWDIDLMGYAQLRRKCELFTYMRFDAEFTFVVAKPNG.....
QWY15712.1    TQETAIGNFFSRAGLVSIITMPTTGTQNTDG.....YVNWDIDLMGYAQLRRKCELFTYMRFDAEFTFVVAKPNG.....
QWY15711.1    TQETAIGNFFSRAGLVSIITMPTTGTQNTDG.....YVNWDIDLMGYAQLRRKCELFTYMRFDAEFTFVVAKPNG.....
QWY15707.1    TQETAIGNFFSRAGLVSIITMPTTGTQNTDG.....YVNWDIDLMGYAQLRRKCELFTYMRFDAEFTFVVAKPNG.....
QWY15688.1    TQETAIGNFFSRAGLVSIITMPTTGTQNTDG.....YVNWDIDLMGYAQLRRKCELFTYMRFDAEFTFVVAKPNG.....
QWY15685.1    TQETAIGNFFSRAGLVSIITMPTTGTQNTDG.....YVNWDIDLMGYAQLRRKCELFTYMRFDAEFTFVVAKPNG.....
QWY15684.1    TQETAIGNFFSRAGLVSIITMPTTGTQNTDG.....YVNWDIDLMGYAQLRRKCELFTYMRFDAEFTFVVAKPNG.....
QYC65170.1    TQETAIGNFFSRAGLVSIITMPTTGTQNTDG.....YVNWDIDLMGYAQLRRKCELFTYMRFDAEFTFVVAKPNG.....
QYC65169.1    TQETAIGNFFSRAGLVSIITMPTTGTQNTDG.....YVNWDIDLMGYAQLRRKCELFTYMRFDAEFTFVVAKPNG.....
QYC65162.1    TQETAIGNFFSRAGLVSIITMPTTGTQNTDG.....YVNWDIDLMGYAQLRRKCELFTYMRFDAEFTFVVAKPNG.....
QYC65099.1    TQETAIGNFFSRAGLVSIITMPTTGTQNTDG.....YVNWDIDLMGYAQLRRKCELFTYMRFDAEFTFVVAKPNG.....
QYC65048.1    TQETAIGNFFSRAGLVSIITMPTTGTQNTDG.....YVNWDIDLMGYAQLRRKCELFTYMRFDAEFTFVVAKPNG.....
QYC65040.1    TQETAIGNFFSRAGLVSIITMPTTGTQNTDG.....YVNWDIDLMGYAQLRRKCELFTYMRFDAEFTFVVAKPNG.....
QYC65035.1    TQETAIGNFFSRAGLVSIITMPTTGTQNTDG.....YVNWDIDLMGYAQLRRKCELFTYMRFDAEFTFVVAKPNG.....
QYC65032.1    TQETAIGNFFSRAGLVSIITMPTTGTQNTDG.....YVNWDIDLMGYAQLRRKCELFTYMRFDAEFTFVVAKPNG.....
QYC65030.1    TQETAIGNFFSRAGLVSIITMPTTGTQNTDG.....YVNWDIDLMGYAQLRRKCELFTYMRFDAEFTFVVAKPNG.....
QYC65027.1    TQETAIGNFFSRAGLVSIITMPTTGTQNTDG.....YVNWDIDLMGYAQLRRKCELFTYMRFDAEFTFVVAKPNG.....
QYC65026.1    TQETAIGNFFSRAGLVSIITMPTTGTQNTDG.....YVNWDIDLMGYAQLRRKCELFTYMRFDAEFTFVVAKPNG.....
QYC65024.1    TQETAIGNFFSRAGLVSIITMPTTGTQNTDG.....YVNWDIDLMGYAQLRRKCELFTYMRFDAEFTFVVAKPNG.....
QYC65022.1    TQETAIGNFFSRAGLVSIITMPTTGTQNTDG.....YVNWDIDLMGYAQLRRKCELFTYMRFDAEFTFVVAKPNG.....
QYC65021.1    TQETAIGNFFSRAGLVSIITMPTTGTQNTDG.....YVNWDIDLMGYAQLRRKCELFTYMRFDAEFTFVVAKPNG.....
QYC65020.1    TQETAIGNFFSRAGLVSIITMPTTGTQNTDG.....YVNWDIDLMGYAQLRRKCELFTYMRFDAEFTFVVAKPNG.....
QYC65019.1    TQETAIGNFFSRAGLVSIITMPTTGTQNTDG.....YVNWDIDLMGYAQLRRKCELFTYMRFDAEFTFVVAKPNG.....
QWF08742.1    TQETAIGNFFSRAGLVSIITMPTTGTQNTDG.....YVNWDIDLMGYAQLRRKCELFTYMRFDAEFTFVVAKPNG.....
QWF08728.1    TQETAIGNFFSRAGLVSIITMPTTGTQNTDG.....YVNWDIDLMGYAQLRRKCELFTYMRFDAEFTFVVAKPNG.....
QWF08726.1    TQETAIGNFFSRAGLVSIITMPTTGAQNTDG.....YVNWDIDLMGYAQLRRKCELFTYMRFDAEFTFVVAKPNG.....
QYC65054.1    TQETAIGNFFSRAGLVSIITMPTTGTQNTDG.....YVNWDIDLLGYAQLRRKCELFTYMRFDAEFTFVVAKPNG.....
QWF08746.1    TQETAIGNFFSRAGLVSIITMPTTGTQNTDG.....YVNWDIDLMGYAQLRRECELFTYMRFDAEFTFVVAKPNG.....
QYC65033.1    TQETAIGNFFSRAGLVSIITMPTTGTQNTDG.....YVNWDIDLMGYAQLRRKCELFTYMRFDAEFTFVVAKPNG.....
QWY15952.1    TQETAIGNFFSRAGLVSIITMPTTGTQNTDG.....YVNWDIDLMGYAQLRRKCELFTYMRFDAEFTFVVAKPNG.....
QYC65042.1    TQETAIGNFFSRAGLVSIITMPTTGTQNTDG.....YVNWDIDLMGYAQLRRKCELFTYMRFDAEFTFVVAKPNG.....
QYC65017.1    TQETAIGNFFSRAGLVSIITMPTTGTQNTDG.....YVNWDIDLMGYAQLRRKCELFTYMRFDAEFTFVVAKPNG.....
QOP74918.1    TQETAIGNFFSRAGLVSIITMPTTGTQNTDG.....YVNWDIDLMGYAQLRRKCELFTYMRFDAEFTFVVAKPNG.....
QWF08731.1    TQETAIGNFFSRAGLVSIITMPTTGTQNTDG.....YVNWDIDLMGYAQLRRKCELFTYMRFDAEFTFVVAKPNG.....
BBH72123.1    TQETAIGNFFSRAGLVSIITMPTTGTQNTDG.....YVNWDIDLMGYAQLRRKCELFTYMRFDAEFTFVVAKPNG.....
BBH72114.1    TQETAIGNFFSRAGLVSIITMPTTGTQNTDG.....YVNWDIDLMGYAQLRRKCELFTYMRFDAEFTFVVAKPNG.....
BBH72092.1    TQETAIGNFFSRAGLVSIITMPTTGTQNTDG.....YVNWDIDLMGYAQLRRKCELFTYMRFDAEFTFVVAKPNG.....
BBH72095.1    TQETAIGNFFSRAGLVSIITMPTTGTQNTDG.....YVNWDIDLMGYAQLRRKCELFTYMRFDAEFTFVVAKPNG.....
BBH72186.1    TQETAIGNFFSRAGLVSIITMPTTGTQNTDG.....YVNWDIDLMGYAQLRRKCELFTYMRFDAEFTFVVAKPNG.....
QWY15975.1    TQETAIGNFFSRAGLVSIITMPTTGTQNTDG.....YVNWDIDLMGYAQLRRKCELFTYMRFDAEFTFVVAKPNG.....
QWY15974.1    TQETAIGNFFSRAGLVSIITMPTTGTQNTDG.....YVNWDIDLMGYAQLRRKCELFTYMRFDAEFTFVVAKPNG.....
QWY15968.1    TQETAIGNFFSRAGLVSIITMPTTGTQNTDG.....YVNWDIDLMGYAQLRRKCELFTYMRFDAEFTFVVAKPNG.....
QWY15960.1    TQETAIGNFFSRAGLVSIITMPTTGTQNTDG.....YVNWDIDLMGYAQLRRKCELFTYMRFDAEFTFVVAKPNG.....
QWY15959.1    TQETAIGNFFSRAGLVSIITMPTTGTQNTDG.....YVNWDIDLMGYAQLRRKCELFTYMRFDAEFTFVVAKPNG.....
QWY15953.1    TQETAIGNFFSRAGLVSIITMPTTGTQNTDG.....YVNWDIDLMGYAQLRRKCELFTYMRFDAEFTFVVAKPNG.....
QWY15951.1    TQETAIGNFFSRAGLVSIITMPTTGTQNTDG.....YVNWDIDLMGYAQLRRKCELFTYMRFDAEFTFVVAKPNG.....
QWY15950.1    TQETAIGNFFSRAGLVSIITMPTTGTQNTDG.....YVNWDIDLMGYAQLRRKCELFTYMRFDAEFTFVVAKPNG.....
QWY15949.1    TQETAIGNFFSRAGLVSIITMPTTGTQNTDG.....YVNWDIDLMGYAQLRRKCELFTYMRFDAEFTFVVAKPNG.....
QWY15944.1    TQETAIGNFFSRAGLVSIITMPTTGTQNTDG.....YVNWDIDLMGYAQLRRKCELFTYMRFDAEFTFVVAKPNG.....
QWY15943.1    TQETAIGNFFSRAGLVSIITMPTTGTQNTDG.....YVNWDIDLMGYAQLRRKCELFTYMRFDAEFTFVVAKPNG.....
QWY15942.1    TQETAIGNFFSRAGLVSIITMPTTGTQNTDG.....YVNWDIDLMGYAQLRRKCELFTYMRFDAEFTFVVAKPNG.....
QWY15939.1    TQETAIGNFFSRAGLVSIITMPTTGTQNTDG.....YVNWDIDLMGYAQLRRKCELFTYMRFDAEFTFVVAKPNG.....
QWY15938.1    TQETAIGNFFSRAGLVSIITMPTTGTQNTDG.....YVNWDIDLMGYAQLRRKCELFTYMRFDAEFTFVVAKPNG.....
QWY15937.1    TQETAIGNFFSRAGLVSIITMPTTGTQNTDG.....YVNWDIDLMGYAQLRRKCELFTYMRFDAEFTFVVAKPNG.....
QWY15936.1    TQETAIGNFFSRAGLVSIITMPTTGTQNTDG.....YVNWDIDLMGYAQLRRKCELFTYMRFDAEFTFVVAKPNG.....
QWY15934.1    TQETAIGNFFSRAGLVSIITMPTTGTQNTDG.....YVNWDIDLMGYAQLRRKCELFTYMRFDAEFTFVVAKPNG.....
QWY15896.1    TQETAIGNFFSRAGLVSIITMPTTGTQNTDG.....YVNWDIDLMGYAQLRRKCELFTYMRFDAEFTFVVAKPNG.....
BCU41098.1    TQETAIGNFFSRAGLVSIITMPTTGTQNTDG.....YVNWDIDLMGYAQLRRKCELFTYMRFDAEFTFVVAKPNG.....
BCR43090.1    TQETAIGNFFSRAGLVSIITMPTTGTQNTDG.....YVNWDIDLMGYAQLRRKCELFTYMRFDAEFTFVVAKPNG.....
QYC65129.1    TQETAIGNFFSRAGLVSIITMPTTGTQNTDG.....YVNWDIDLMGYAQLRRKCELFTYMRFDAEFTFVVAKPNG.....
QWF08961.1    TQETAIGNFFSRAGLVSIITMPTTGTQNTDG.....YVNWDIDLMGYAQLRRKCELFTYMRFDAEFTFVVAKPNG.....
QIL87527.1    TQETAIGNFFSRAGLVSIITMPTTGTQNTDG.....YVNWDIDLMGYAQLRRKCELFTYMRFDAEFTFVVAKPNG.....
QWY15955.1    TQETAIGNFFSRAGLVSIITMPTTGTQNTDG.....YVNWDIDLMGYAQLRRKCELFTYMRFDAEFTFVVAKPNG.....
QWY15954.1    TQETAIGNFFSRAGLVSIITMPTTGTQNTDG.....YVNWDIDLMGYAQLRRKCELFTYMRFDAEFTFVVAKPNG.....
QWY15945.1    TQETAIGNFFSRAGLVSIITMPTTGTQNTDG.....YVNWDIDLMGYAQLRRKCELFTYMRFDAEFTFVVAKPNG.....
QWY15935.1    TQETAIGNFFSRAGLVSIITMPTTGTQNTDG.....YVNWDIDLMGYAQLRRKCELFTYMRFDAEFTFVVAKPNG.....
BCB92163.1    TQETAIGNFFSRAGLVSIISMPTTGTQNTDG.....YVNWDIDLMGYAQLRRKCELFTYMRFDAEFTFVVAKPNG.....
BCB92158.1    TQETAIGNFFSRAGLVSIISMPTTGTQNTDG.....YVNWDIDLMGYAQLRRKCELFTYMRFDAEFTFVVAKPNG.....
BCB92154.1    TQETAIGNFFSRAGLVSIISMPTTGTQNTDG.....YVNWDIDLMGYAQLRRKCELFTYMRFDAEFTFVVAKPNG.....
BCB92152.1    TQETAIGNFFSRAGLVSIISMPTTGTQNTDG.....YVNWDIDLMGYAQLRRKCELFTYMRFDAEFTFVVAKPNG.....
BCB92162.1    TQETAIGNFFSRAGLVSIISMPTTGTQNTDG.....YVNWDIDLMGYAQLRRKCELFTYMRFDAEFTFVVAKPNG.....
BCB92161.1    TQETAIGNFFSRAGLVSIISMPTTGTQNTDG.....YVNWDIDLMGYAQLRRKCELFTYMRFDAEFTFVVAKPNG.....
BCB92160.1    TQETAIGNFFSRAGLVSIISMPTTGTQNTDG.....YVNWDIDLMGYAQLRRKCELFTYMRFDAEFTFVVAKPNG.....
BBH72129.1    TQETAIGNFFSRAGLVSIITMPTAGTQNTDG.....YVNWDIDLMGYAQLRRKCELFTYMRFDAEFTFVVAKPNG.....
QIL87533.1    TQETAIGNFFSRAGLVSIITMPTTGTQNTDG.....YVNWDIDLMGYAQLRRKCELFTYMRFDAEFTFVVAKPNG.....
BBH72150.1    TQETAIGNFFSRAGLVSIITMPTTGTQNTDG.....YVNWDIDLMGYAQLRRKCELFTYMRFDAEFTFVVAKPNG.....
BBH72137.1    TQETAIGNFFSRAGLVSIITMPTTGTQNTDG.....YVNWDIDLMGYAQLRRKCELFTYMRFDAEFTFVVAKPNG.....
BBH72145.1    TQETAIGNFFSRAGLVSIITMPTTGTQNTDG.....YVNWDIDLMGYAQLRRKCELFTYMRFDAEFTFVVAKPNG.....
BCB92157.1    TQETAIGNFFSRAGLVSIITMPTTGTQNTDG.....YVNWDIDLMGYAQLRRKCELFTYMRFDAEFTFVVAKPNG.....
QYC65124.1    TQETAIGNFFSRAGLVSIITMPTTGTQNTDG.....YVNWDIDLMGYAQLRRKCELFTYMRFDAEFTFVVAKPNG.....
QWY15920.1    TQETAIGNFFSRAGLVSIITMPTTGTQNTDG.....YVNWDIDLMGYAQLRRKCELFTYMRFDAEFTFVVAKPNG.....
QWY15973.1    TQETAIGNFFSRAGLVSIITMPTTGTQNTDG.....YVNWDIDLMGYAQLRRKCELFTYMRFDAEFTFVVAKPNG.....
BCU41116.1    TQETAIGNFFSRAGLVSIITMPTTGTQNTDG.....YVNWDIDLMGYAQLRRKCELFTYMRFDAEFTFVVAKPNG.....
QWF08960.1    TQETAIGNFFSRAGLVSIITMPTTGTQNTDG.....YVNWDIDLMGYAQLRRKCELFTYMRFDAEFTFVVAKPNG.....
QWF08958.1    TQETAIGNFFSRAGLVSIITMPTTGTQNTDG.....YVNWDIDLMGYAQLRRKCELFTYMRFDAEFTFVVAKPNG.....
QWF08919.1    TQETAIGNFFSRAGLVSIITMPTTGTQNTDG.....YVNWDIDLMGYAQLRRKCELFTYMRFDAEFTFVVAKPNG.....
QWY15825.1    TQETAIGNFFSRAGLVSIITMPTTGTQNTDG.....YVNWDIDLMGYAQLRRKCELFTYMRFDAEFTFVVAKPNG.....
QWF08953.1    TQETAIGNFFSRAGLVSIITMPTTGTQNTDG.....YVNWDIDLMGYAQLRRKCELFTYMRFDAEFTFVVAKPNG.....
QWF08948.1    TQETAIGNFFSRAGLVSIITMPTTGTQNTDG.....YVNWDIDLMGYAQLRRKCELFTYMRFDAEFTFVVAKPNG.....
QWY15884.1    TQETAIGNFFSRAGLVSIITMPTTGTQNTDG.....YVNWDIDLMGYAQLRRKCELFTYMRFDAEFTFVVAKPNG.....
QWF08946.1    TQETAIGNFFSRAGLVSIITMPTTGTQNTDG.....YVNWDIDLMGYAQLRRKCELFTYMRFDAEFTFVVAKPNG.....
QWF08941.1    TQETAIGNFFSRAGLVSIITMPTTGTQNTDG.....YVNWDIDLMGYAQLRRKCELFTYMRFDAEFTFVVAKPNG.....
QWF08914.1    TQETAIGNFFSRAGLVSIITMPTTGTQNTDG.....YVNWDIDLMGYAQLRRKCELFTYMRFDAEFTFVVAKPNG.....
BCU41097.1    TQETAIGNFFSRAGLVSIITMPTTGTQNTDG.....YVNWDIDLMGYAQLRRKCELFTYMRFDAEFTFVVAKPNG.....
QWY15918.1    TQETAIGNFFSRAGLVSIITMPTTGTQNTDG.....YVNWDIDLMGYAQLRRKCELFTYMRFDAEFTFVVAKPNG.....
BCR43086.1    TQETAIGNFFSRAGLVSIITMPTTGTQNTDG.....YVNWDIDLMGYAQLRRKCELFTYMRFDAEFTFVVAKPNG.....
QWF08935.1    TQETAIGNFFSRAGLVSIITMPTTGTQNTDG.....YVNWDIDLMGYAQLRRKCELFTYMRFDAEFTFVVAKPNG.....
QWY15912.1    TQETAIGNFFSRAGLVSIITMPTTGTQNTDG.....YVNWDIDLMGYAQLRRKCELFTYMRFDAEFTFVVAKPNG.....
QWY15911.1    TQETAIGNFFSRAGLVSIITMPTTGTQNTDG.....YVNWDIDLMGYAQLRRKCELFTYMRFDAEFTFVVAKPNG.....
QWY15887.1    TQETAIGNFFSRAGLVSIITMPTTGTQNTDG.....YVNWDIDLMGYAQLRRKCELFTYMRFDAEFTFVVAKPNG.....
```

```
QWY15882.1   TQETAIGNFFSRAGLVSIITMPTTGTQNTDG.....YVNWDIDLMGYAQLRRKCELFTYMRFDAEFTFVVAKPNG.....
BCG67018.1   TQETAIGNFFSRAGLVSIITMPTTGTQNTDG.....YVNWDIDLMGYAQLRRKCELFTYMRFDAEFTFVVAKPNG.....
QWY15881.1   TQETAIGNFFSRAGLVSIITMPTTGTQNTDG.....YVNWDIDLMGYAQLRRKCELFTYMRFDAEFTFVVAKPNG.....
QWY15875.1   TQETAIGNFFSRAGLVSIITMPTTGTQNTDG.....YVNWDIDLMGYAQLRRKCELFTYMRFDAEFTFVVAKPNG.....
BCG67016.1   TQETAIGNFFSRAGLVSIITMPTTGTQNTDG.....YVNWDIDLMGYAQLRRKCELFTYMRFDAEFTFVVAKPNG.....
QWY15874.1   TQETAIGNFFSRAGLVSIITMPTTGTQNTDG.....YVNWDIDLMGYAQLRRKCELFTYMRFDAEFTFVVAKPNG.....
QWY15853.1   TQETAIGNFFSRAGLVSIITMPTTGTQNTDG.....YVNWDIDLMGYAQLRRKCELFTYMRFDAEFTFVVAKPNG.....
BCG67014.1   TQETAIGNFFSRAGLVSIITMPTTGTQNTDG.....YVNWDIDLMGYAQLRRKCELFTYMRFDAEFTFVVAKPNG.....
BCG67009.1   TQETAIGNFFSRAGLVSIITMPTTGTQNTDG.....YVNWDIDLMGYAQLRRKCELFTYMRFDAEFTFVVAKPNG.....
QWF08933.1   TQETAIGNFFSRAGLVSIITMPTTGTQNTDG.....YVNWDIDLMGYAQLRRKCELFTYMRFDAEFTFVVAKPNG.....
BCG66992.1   TQETAIGNFFSRAGLVSIITMPTTGTQNTDG.....YVNWDIDLMGYAQLRRKCELFTYMRFDAEFTFVVAKPNG.....
BCG66991.1   TQETAIGNFFSRAGLVSIITMPTTGTQNTDG.....YVNWDIDLMGYAQLRRKCELFTYMRFDAEFTFVVAKPNG.....
QWF08898.1   TQETAIGNFFSRAGLVSIITMPTTGTQNTDG.....YVNWDIDLMGYAQLRRKCELFTYMRFDAEFTFVVAKPNG.....
QWF08930.1   TQETAIGNFFSRAGLVSIITMPTTGTQNTDG.....YVNWDIDLMGYAQLRRKCELFTYMRFDAEFTFVVAKPNG.....
QWF08925.1   TQETAIGNFFSRAGLVSIITMPTTGTQNTDG.....YVNWDIDLMGYAQLRRKCELFTYMRFDAEFTFVVAKPNG.....
QWF08913.1   TQETAIGNFFSRAGLVSIITMPTTGTQNTDG.....YVNWDIDLMGYAQLRRKCELFTYMRFDAEFTFVVAKPNG.....
QWF08911.1   TQETAIGNFFSRAGLVSIITMPTTGTQNTDG.....YVNWDIDLMGYAQLRRKCELFTYMRFDAEFTFVVAKPNG.....
BBH72117.1   TQETAIGNFFSRAGLVSIITMPTTGTQNTDG.....YVNWDIDLMGYAQLRRKCELFTYMRFDAEFTFVVAKPNG.....
BBH72116.1   TQETAIGNFFSRAGLVSIITMPTTGTQNTDG.....YVNWDIDLMGYAQLRRKCELFTYMRFDAEFTFVVAKPNG.....
BBH72113.1   TQETAIGNFFSRAGLVSIITMPTTGTQNTDG.....YVNWDIDLMGYAQLRRKCELFTYMRFDAEFTFVVAKPNG.....
BBH72112.1   TQETAIGNFFSRAGLVSIITMPTTGTQNTDG.....YVNWDIDLMGYAQLRRKCELFTYMRFDAEFTFVVAKPNG.....
BBH72108.1   TQETAIGNFFSRAGLVSIITMPTTGTQNTDG.....YVNWDIDLMGYAQLRRKCELFTYMRFDAEFTFVVAKPNG.....
BBH72106.1   TQETAIGNFFSRAGLVSIITMPTTGTQNTDG.....YVNWDIDLMGYAQLRRKCELFTYMRFDAEFTFVVAKPNG.....
BBH72104.1   TQETAIGNFFSRAGLVSIITMPTTGTQNTDG.....YVNWDIDLMGYAQLRRKCELFTYMRFDAEFTFVVAKPNG.....
BBH72211.1   TQETAIGNFFSRAGLVSIITMPTTGTQNTDG.....YVNWDIDLMGYAQLRRKCELFTYMRFDAEFTFVVAKPNG.....
BBH72210.1   TQETAIGNFFSRAGLVSIITMPTTGTQNTDG.....YVNWDIDLMGYAQLRRKCELFTYMRFDAEFTFVVAKPNG.....
BBH72209.1   TQETAIGNFFSRAGLVSIITMPTTGTQNTDG.....YVNWDIDLMGYAQLRRKCELFTYMRFDAEFTFVVAKPNG.....
BBH72208.1   TQETAIGNFFSRAGLVSIITMPTTGTQNTDG.....YVNWDIDLMGYAQLRRKCELFTYMRFDAEFTFVVAKPNG.....
BBH72207.1   TQETAIGNFFSRAGLVSIITMPTTGTQNTDG.....YVNWDIDLMGYAQLRRKCELFTYMRFDAEFTFVVAKPNG.....
BBH72205.1   TQETAIGNFFSRAGLVSIITMPTTGTQNTDG.....YVNWDIDLMGYAQLRRKCELFTYMRFDAEFTFVVAKPNG.....
BBH72201.1   TQETAIGNFFSRAGLVSIITMPTTGTQNTDG.....YVNWDIDLMGYAQLRRKCELFTYMRFDAEFTFVVAKPNG.....
BBH72193.1   TQETAIGNFFSRAGLVSIITMPTTGTQNTDG.....YVNWDIDLMGYAQLRRKCELFTYMRFDAEFTFVVAKPNG.....
BBH72192.1   TQETAIGNFFSRAGLVSIITMPTTGTQNTDG.....YVNWDIDLMGYAQLRRKCELFTYMRFDAEFTFVVAKPNG.....
BBH72190.1   TQETAIGNFFSRAGLVSIITMPTTGTQNTDG.....YVNWDIDLMGYAQLRRKCELFTYMRFDAEFTFVVAKPNG.....
BBH72189.1   TQETAIGNFFSRAGLVSIITMPTTGTQNTDG.....YVNWDIDLMGYAQLRRKCELFTYMRFDAEFTFVVAKPNG.....
BBH72188.1   TQETAIGNFFSRAGLVSIITMPTTGTQNTDG.....YVNWDIDLMGYAQLRRKCELFTYMRFDAEFTFVVAKPNG.....
BBH72187.1   TQETAIGNFFSRAGLVSIITMPTTGTQNTDG.....YVNWDIDLMGYAQLRRKCELFTYMRFDAEFTFVVAKPNG.....
BBH72185.1   TQETAIGNFFSRAGLVSIITMPTTGTQNTDG.....YVNWDIDLMGYAQLRRKCELFTYMRFDAEFTFVVAKPNG.....
BBH72182.1   TQETAIGNFFSRAGLVSIITMPTTGTQNTDG.....YVNWDIDLMGYAQLRRKCELFTYMRFDAEFTFVVAKPNG.....
BBH72181.1   TQETAIGNFFSRAGLVSIITMPTTGTQNTDG.....YVNWDIDLMGYAQLRRKCELFTYMRFDAEFTFVVAKPNG.....
BBH72179.1   TQETAIGNFFSRAGLVSIITMPTTGTQNTDG.....YVNWDIDLMGYAQLRRKCELFTYMRFDAEFTFVVAKPNG.....
BBH72178.1   TQETAIGNFFSRAGLVSIITMPTTGTQNTDG.....YVNWDIDLMGYAQLRRKCELFTYMRFDAEFTFVVAKPNG.....
BBH72177.1   TQETAIGNFFSRAGLVSIITMPTTGTQNTDG.....YVNWDIDLMGYAQLRRKCELFTYMRFDAEFTFVVAKPNG.....
BBH72175.1   TQETAIGNFFSRAGLVSIITMPTTGTQNTDG.....YVNWDIDLMGYAQLRRKCELFTYMRFDAEFTFVVAKPNG.....
BBH72174.1   TQETAIGNFFSRAGLVSIITMPTTGTQNTDG.....YVNWDIDLMGYAQLRRKCELFTYMRFDAEFTFVVAKPNG.....
BBH72172.1   TQETAIGNFFSRAGLVSIITMPTTGTQNTDG.....YVNWDIDLMGYAQLRRKCELFTYMRFDAEFTFVVAKPNG.....
BBH72170.1   TQETAIGNFFSRAGLVSIITMPTTGTQNTDG.....YVNWDIDLMGYAQLRRKCELFTYMRFDAEFTFVVAKPNG.....
BBH72169.1   TQETAIGNFFSRAGLVSIITMPTTGTQNTDG.....YVNWDIDLMGYAQLRRKCELFTYMRFDAEFTFVVAKPNG.....
BBH72168.1   TQETAIGNFFSRAGLVSIITMPTTGTQNTDG.....YVNWDIDLMGYAQLRRKCELFTYMRFDAEFTFVVAKPNG.....
BBH72158.1   TQETAIGNFFSRAGLVSIITMPTTGTQNTDG.....YVNWDIDLMGYAQLRRKCELFTYMRFDAEFTFVVAKPNG.....
BBH72155.1   TQETAIGNFFSRAGLVSIITMPTTGTQNTDG.....YVNWDIDLMGYAQLRRKCELFTYMRFDAEFTFVVAKPNG.....
BBH72154.1   TQETAIGNFFSRAGLVSIITMPTTGTQNTDG.....YVNWDIDLMGYAQLRRKCELFTYMRFDAEFTFVVAKPNG.....
BBH72151.1   TQETAIGNFFSRAGLVSIITMPTTGTQNTDG.....YVNWDIDLMGYAQLRRKCELFTYMRFDAEFTFVVAKPNG.....
BBH72148.1   TQETAIGNFFSRAGLVSIITMPTTGTQNTDG.....YVNWDIDLMGYAQLRRKCELFTYMRFDAEFTFVVAKPNG.....
BBH72144.1   TQETAIGNFFSRAGLVSIITMPTTGTQNTDG.....YVNWDIDLMGYAQLRRKCELFTYMRFDAEFTFVVAKPNG.....
BBH72142.1   TQETAIGNFFSRAGLVSIITMPTTGTQNTDG.....YVNWDIDLMGYAQLRRKCELFTYMRFDAEFTFVVAKPNG.....
BBH72141.1   TQETAIGNFFSRAGLVSIITMPTTGTQNTDG.....YVNWDIDLMGYAQLRRKCELFTYMRFDAEFTFVVAKPNG.....
BBH72140.1   TQETAIGNFFSRAGLVSIITMPTTGTQNTDG.....YVNWDIDLMGYAQLRRKCELFTYMRFDAEFTFVVAKPNG.....
BBH72132.1   TQETAIGNFFSRAGLVSIITMPTTGTQNTDG.....YVNWDIDLMGYAQLRRKCELFTYMRFDAEFTFVVAKPNG.....
BBH72131.1   TQETAIGNFFSRAGLVSIITMPTTGTQNTDG.....YVNWDIDLMGYAQLRRKCELFTYMRFDAEFTFVVAKPNG.....
BBH72130.1   TQETAIGNFFSRAGLVSIITMPTTGTQNTDG.....YVNWDIDLMGYAQLRRKCELFTYMRFDAEFTFVVAKPNG.....
BBH72128.1   TQETAIGNFFSRAGLVSIITMPTTGTQNTDG.....YVNWDIDLMGYAQLRRKCELFTYMRFDAEFTFVVAKPNG.....
BDC16283.1   TQETAIGNFFSRAGLVSIITMPTTGTQNTDG.....YVNWDIDLMGYAQLRRKCELFTYMRFDAEFTFVVAKPNG.....
BDC16271.1   TQETAIGNFFSRAGLVSIITMPTTGTQNTDG.....YVNWDIDLMGYAQLRRKCELFTYMRFDAEFTFVVAKPNG.....
BDC16270.1   TQETAIGNFFSRAGLVSIITMPTTGTQNTDG.....YVNWDIDLMGYAQLRRKCELFTYMRFDAEFTFVVAKPNG.....
BDC16258.1   TQETAIGNFFSRAGLVSIITMPTTGTQNTDG.....YVNWDIDLMGYAQLRRKCELFTYMRFDAEFTFVVAKPNG.....
QIL87526.1   TQETAIGNFFSRAGLVSIITMPTTGTQNTDG.....YVNWDIDLMGYAQLRRKCELFTYMRFDAEFTFVVAKPNG.....
BBH72180.1   TQETAIGNFFSRAGLVSIITMPTTGTQNTDG.....YVNWDIDLMGYAHLRRKCELFTYMPFDAEFTFVVAKPNG.....
BBH72109.1   TQETAIGNFFSRAGLVSIITMPTTGTQNTDG.....YVNWDIDLMGYAQLRRKCELFTYMRFDAEFTFVVAKPNG.....
BBH72115.1   TQETAIGNFFSRAGLVSIITMPTTGTQNTDG.....YVNWDIDLMGYAQLRRKCELFTYMRFDAEFTFVVAKPNG.....
BBH72152.1   TQETAIGNFFSRAGLVSIITMPTTGTQNTDG.....YVNWDIDLMGYAQLRRKCELFTYMRFDAEFTFVVAKPNG.....
BBH72200.1   TQETAIGNFFSRAGLVSIITMPTTGTQNTDG.....YVNWDIDLMGYAQLRRKCELFTYMRFDAEFTFVVAKPNG.....
QWY15844.1   TQETAIGNFFSRAGLVSIITMPTTGTQNTDG.....YINWDIDLMGYAQLRRKCELFTYMRFDAEFTFVVAKPNG.....
BBH72146.1   TQETAIGNFFSRAGLVSIITMPTTGTQNTDG.....YVNWDIDLMGYAQLRRKCELFTYMRFDAEFTFVVAKPNG.....
BBH72173.1   TQETAIGNFFSRAGLVSIITMPTTGTQNTDG.....YVNWDIDLMGYAQLRRKCELFTYMRFDAEFTFVVAKPNG.....
QWY15947.1   TQETAIGNFFSRAGLVSIITMPTTGTQNTDG.....YVNWDIDLMGYAQLRRKCELFTYMRFDAEFTFVVAKPNG.....
BDC16281.1   TQETAIGNFFSRAGLVSIITMPTTGTQNTDG.....YVNWDIDLMGYAQLRRKCELFTYMRFDAEFTFVVAKPNG.....
BBH72167.1   TQETAIGNFFSRAGLVSIITMPTTGTQNTDG.....YVNWDIDLMGYAQLRRKCELFTYMRFDAEFTFVVAKPNG.....
BBH72160.1   TQETAIGNFFSRAGLVSIITMPTTGTQNTDG.....YVNWDIDLMGYAQLRRKCELFTYMRFDAEFTFVVAKPNG.....
BCU41096.1   TQETAIGNFFSRAGLVSIITMPTTGTQNTDG.....YVNWDIDLMGYAQLRRKCELFTYMRFDAEFTFVVAKPNG.....
BCR43102.1   TQETAIGNFFSRAGLVSIITMPTTGTQNTDG.....YVNWDIDLMGYAQLRRKCELFTYMRFDAEFTFVVAKPNG.....
QWF08881.1   TQETAIGNFFSRAGLVSIITMPTTGTQNTDG.....YVNWDIDLMGYAQLRRKCELFTYMRFDAEFTFVVAKPNG.....
QWF08855.1   TQETAIGNFFSRAGLVSIITMPTTGTQNTDG.....YVNWDIDLMGYAQLRRKCELFTYMRFDAEFTFVVAKPNG.....
BBH72195.1   TQETAIGNFFSRAGLVSIITMPTTGTQNTDG.....YVNWDIDLMGYAQLRRKCELFTYMRFDAEFTFVVAKPNG.....
BBH72118.1   TQETAIGNFFSRAGLVSIITMPTTGTQNTDG.....YVNWDIDLMGYAQLRRKCELFTYMRFDAEFTFVVAKPNG.....
BBH72126.1   TQETAIGNFFSRAGLVSIITMPTTGTQNTDG.....YVNWDIDLMGYAQLRRKCELFTYMRFDAEFTFVVAKPNG.....
BBH72127.1   TQETAIGNFFSRAGLVSIITMPTTGTQNTDG.....YVNWDIDLMGYAQLRRKCELFTYMRFDAEFTFVVAKPNG.....
BBH72206.1   TQETAIGNFFSRAGLVSIITMPTTGTQNTDG.....YVNWDIDLMGYAQLRRKCELFTYMRFDAEFTFVVAKPNG.....
BBH72196.1   TQETAIGNFFSRAGLVSIITMPTTGTQNTDG.....YVNWDIDLMGYAQLRRKCELFTYMRFDAEFTFVVAKPNG.....
BBH72171.1   TQETAIGNFFSRAGLVSIITMPTTGTQNTDG.....YVNWDIDLMGYAQLRRKCELFTYMRFDAEFTFVVAKPNG.....
BBH72191.1   TQETAIGNFFSRAGLVSIITMPTTGTQNTDG.....YVNWDIDLMGYAQLRRKCELFTYMRFDAEFTFVVAKPNG.....
BBH72133.1   TQETAIGNFFSRAGLVSIITMPTTGTQNTDG.....YVNWDIDLMGYAQLRRKCELFTYMRFDAEFTFVVAKPNG.....
BBH72139.1   TQETAIGNFFSRAGLVSIITMPTTGTQNTDG.....YVNWDIDLMGYAQLRRKCELFTYMRFDAEFTFVVAKPNG.....
BBH72166.1   TQETAVGNFFNRAGLVSIITMPNTGTQNTNS.....YLNWDIDLMGYAQLLRKCQLFTYMRFDAEFTFVVAKPNG.....
BBH72161.1   TQETAVGNFFNRAGLVSIITMPTTGTQNTNS.....YLNWDIDLMGYAQLRRKCQLFTYMRFDAEFTFVVAKPNG.....
BBH72165.1   TQETAVGNFFNRAGLVSIITMPNTGTQNTNG.....YLNWDIDLMGYAQLLRRCQLFTYMRFDAEFTFVVAKPNG.....
BBH72164.1   TQETAVGNFFSRAGLVSIITMPTTGTQNTNG.....YVNWDIDLMGYAQLLRRCQLFTYMRFDAEFTFVVAKPNG.....
BBH72159.1   TQETAIGNFFSRAGLVSIITMPTTGTQNTNG.....YLNWDIDLMGYAQLRRKCQLFTYMRFDAEFTFVVAKPNG.....
BBH72157.1   TQETAVGNFFSRAGLVSIITMPTTGTQNTDG.....YVNWDIDLMGYAQLRRRCQLFTYMRFDAEFTFVVAKPNG.....
BBH72162.1   TQETAIGNFFSRAGLVSIITMPTTGTQNTDG.....YVNWDIDLMGYAQLRRRCQLFTYMRFDAEFTFVVAKPNG.....
BBH72153.1   TQETAIGNFFSRAGLVSIITMPTTGTQNTDG.....YVNWDIDLMGYAQLRRKCELFTYMRFDAEFTFVVAKPNG.....
BBH72156.1   TQETAIGNFFSRAGLVSIITMPTTGTQNTDG.....YVNWDIDLMGYAQLRRKCQLFTYMRFDAEFTFVVAKPNG.....
BBH72120.1   TQETAIGNFFSRAGLVSIITMPTTGTQNTNG.....YVNWDIDLMGYAQLRRKCELFTYMRFDAEFTFVVAKPNG.....
BBH72198.1   TQETAIGNFFSRAGLVSIITMPTTGTQNTDG.....YVNWDIDLMGYAQLRRKCELFTYMRFDAEFTFVVAKPNG.....
BBH72197.1   TQETAIGNFFSRAGLVSIITMPTTGTQNTDG.....YVNWDIDLMGYAQLRRKCELFTYMRFDAEFTFVVAKPNG.....
QWY15941.1   TQETAIGNFFSRAGLVSIITMPTTGTQNTDG.....YVNWDIDLMGYAQLRRKCELFTYMRFDAEFTFVVAKPNG.....
BBH72121.1   TQETAIGNFFSRAGLVSIITMPTTGTQNTDG.....YVNWDIDLMGYAQLRRKCELFTYMRFDAEFTFVVAKPNG.....
BBH72135.1   TQETAIGNFFSRAGLVSIITMPTTGTQNTDG.....YVNWDIDLMGYAQLRRKCELFTYMRFDAEFTFVVAKPNG.....
QWY15967.1   TQETAIGNFFSRAGLVSIITMPTTGTQNTDG.....YVNWDIDLMGYAQLRRKCELFTYMRFDAEFTFVVAKPNG.....
QWY15964.1   TQETAIGNFFSRAGLVSIITMPTTGTQNTDG.....YVNWDIDLMGYAQLRRKCELFTYMRFDAEFTFVVAKPNG.....
QWY15969.1   TQETAIGNFFSRAGLVSIITMPTTGTQNTDG.....YVNWDIDLMGYAQLRRKCELFTYMRFDAEFTFVVAKPNG.....
QWY15913.1   TQETAIGNFFSRAGLVSIITMPTTGTQNTDG.....YVNWDIDLMGYAQLRRKCELFTYMRFDAEFTFVVAKPNG.....
QWY15908.1   TQETAIGNFFSRAGLVSIITMPTTGTQNTDG.....YVNWDIDLMGYAQLRRKCELFTYMRFDAEFTFVVAKPNG.....
QWY15907.1   TQETAIGNFFSRAGLVSIITMPTTGTQNTDG.....YVNWDIDLMGYAQLRRKCELFTYMRFDAEFTFVVAKPNG.....
QWY15890.1   TQETAIGNFFSRAGLVSIITMPTTGTQNTDG.....YVNWDIDLMGYAQLRRKCELFTYMRFDAEFTFVVAKPNG.....
QWY15889.1   TQETAIGNFFSRAGLVSIITMPTTGTQNTDG.....YVNWDIDLMGYAQLRRKCELFTYMRFDAEFTFVVAKPNG.....
QWY15888.1   TQETAIGNFFSRAGLVSIITMPTTGTQNTDG.....YVNWDIDLMGYAQLRRKCELFTYMRFDAEFTFVVAKPNG.....
QWY15886.1   TQETAIGNFFSRAGLVSIITMPTTGTQNTDG.....YVNWDIDLMGYAQLRRKCELFTYMRFDAEFTFVVAKPNG.....
QWY15885.1   TQETAIGNFFSRAGLVSIITMPTTGTQNTDG.....YVNWDIDLMGYAQLRRKCELFTYMRFDAEFTFVVAKPNG.....
QWY15883.1   TQETAIGNFFSRAGLVSIITMPTTGTQNTDG.....YVNWDIDLMGYAQLRRKCELFTYMRFDAEFTFVVAKPNG.....
QWY15878.1   TQETAIGNFFSRAGLVSIITMPTTGTQNTDG.....YVNWDIDLMGYAQLRRKCELFTYMRFDAEFTFVVAKPNG.....
QWY15876.1   TQETAIGNFFSRAGLVSIITMPTTGTQNTDG.....YVNWDIDLMGYAQLRRKCELFTYMRFDAEFTFVVAKPNG.....
BBH72176.1   TQETAIGNFFSRAGLVSIITMPTTGTQNTDG.....YVNWDIDLMGYAQLRRKCELFTYMRFDAEFTFVVAKPNG.....
QIL87519.1   TQETAIGNFFSRAGLVSIITMPTTGTQNTDG.....YVNWDIDLMGYAQLRRKCELFTYMRFDAEFTFVVAKPNG.....
QWY15906.1   TQETAIGNFFSRAGLVSIITMPTTGTQNTDG.....YVNWDIDLMGYAQLRRKCELFTYMRFDAEFTFVVAKPNG.....
QWY15917.1   TQETAIGNFFSRAGLVSIITMPTTGTQNTDG.....YVNWDIDLMGYAQLRRKCELFTYMRFDAEFTFVVAKPNG.....
QWY15846.1   TQETAIGNFFSRAGLVSIITMPTTGTQNTDG.....YVNWDIDLMGYAQLRRKCELFTYMRFDAEFTFVVAKPNG.....
QWY15901.1   TQETAIGNFFSRAGLVSIITMPTTGTQNTDG.....YVNWDIDLMGYAQLRRKCELFTYMRFDAEFTFVVAKPNG.....
QWY15857.1   TQETAIGNFFSRAGLVSIITMPTTGTQNTDG.....YVNWDIDLMGYAQLRRKCELFTYMRFDAEFTFVVAKPNG.....
QWY15904.1   TQETAIGNFFSRAGLVSIITMPTTGTQNTDG.....YVNWDIDLMGYAQLRRKCELFTYMRFDAEFTFVVAKPNG.....
BBH72204.1   TQETAIGNFFSRAGLVSIITMPTTGTQNTDG.....YVNWDIDLMGYAQLRRKCELFTYMRFDAEFTFVVAKPNG.....
BBH72203.1   TQETAIGNFFSRAGLVSIITMPTTGTQNTDG.....YVNWDIDLMGYAQLRRKCELFTYMRFDAEFTFVVAKPNG.....
BBH72202.1   TQETAIGNFFSRAGLVSIITMPTTGTQNTDG.....YVNWDIDLMGYAQLRRKCELFTYMRFDAEFTFVVAKPNG.....
BDC16266.1   TQETAIGNFFSRAGLVSIITMPTTGTQNTDG.....YVNWDIDLMGYAQLRRKCELFTYMRFDAEFTFVVAKPNG.....
BCR43091.1   TQETAIGNFFSRAGLVSIITMPTTGTQNTDG.....YVNWDIDLMGYAQLRRKCELFTYMRFDAEFTFVVAKPNG.....
QWY15902.1   TQETAIGNFFSRAGLVSIITMPTTGTQNTDG.....YVNWDIDLMGYAQLRRKCELFTYMRFDAEFTFVVAKPNG.....
BBH72226.1   TRETHIEHFFSRSGLVGVMEIDDTGTSG.KG.....FSNWDIDIMAFVQLRRKLEAFTYMRFDAEFTFVTNLENG.....
BBH72222.1   TRETHIEHFFSRSGLVGVMEIDDTGTSG.KG.....FSNWDIDIMAFVQLRRKLEAFTYMRFDAEFTFVTNLENG.....
BBH72012.1   VNEASVEHFYSRAGLVGVVEVKDSGTSL.DG.....YTVWPIDVMGFVQQRRKLELSTYMRFDAEFTFVSNLNDS.....
BBH71965.1   VNEASVEHFYSRAGLVGVVEVKDSGTSL.DG.....YTVWPIDVMGFVQQRRKLELSTYMRFDAEFTFVSNLNDS.....
BBH71934.1   VNEASVEHFYSRAGLVGVVEVKDSGTSL.DG.....YTVWPIDVMGFVQQRRKLELSTYMRFDAEFTFVSNLNDS.....
BBH72009.1   VNEASVEHFYSRAGLVGVVEVKDSGTSL.DG.....YTVWPIDVMGFVQQRRKLELSTYMRFDAEFTFVSNLNDS.....
QWY16175.1   VMETSVEHFFSRSGLAGILIIE.DSGTSTKG.....YATWEIDVMGFVQLRRKLEMFTYMRFDAEFTFITAERNG.....
QWY16167.1   VMETSVEHFFSRSGLAGILIIE.DSGTSTKG.....YATWEIDVMGFVQLRRKLEMFTYMRFDAEFTFITAERNG.....
```

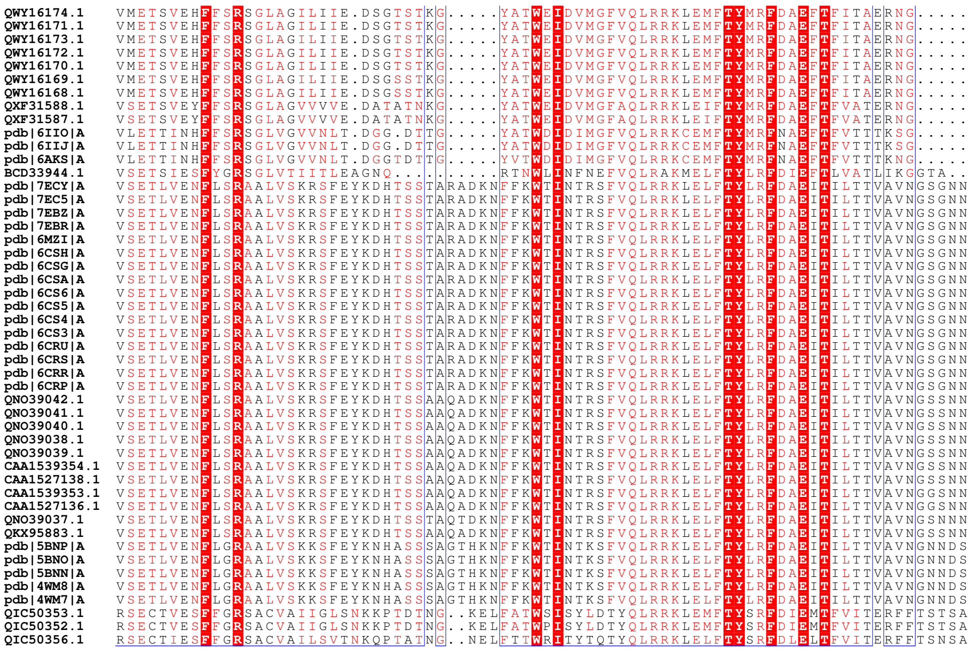
QWY16174.1 VMETSVEHFFSRSGLAGILIIE.DSGTSTKG.....YATWEIDVMGFVQLRRKLEMFTYMRFDAEFTFITAERNG.....
QWY16171.1 VMETSVEHFFSRSGLAGILIIE.DSGTSTKG.....YATWEIDVMGFVQLRRKLEMFTYMRFDAEFTFITAERNG.....
QWY16173.1 VMETSVEHFFSRSGLAGILIIE.DSGTSTKG.....YATWEIDVMGFVQLRRKLEMFTYMRFDAEFTFITAERNG.....
QWY16172.1 VMETSVEHFFSRSGLAGILIIE.DSGTSTKG.....YATWEIDVMGFVQLRRKLEMFTYMRFDAEFTFITAERNG.....
QWY16170.1 VMETSVEHFFSRSGLAGILIIE.DSGTSTKG.....YATWEIDVMGFVQLRRKLEMFTYMRFDAEFTFITAERNG.....
QWY16169.1 VMETSVEHFFSRSGLAGILIIE.DSGSSTKG.....YATWEIDVMGFVQLRRKLEMFTYMRFDAEFTFITAERNG.....
QWY16168.1 VMETSVEHFFSRSGLAGILIIE.DSGSSTKG.....YATWEIDVMGFVQLRRKLEMFTYMRFDAEFTFITAERNG.....
QXF31588.1 VSETSVEYFFSRSGLAGVVVVE.DATATNKG.....YATWEIDVMGFAQLRRKLEIFTYMRFDAEFTFVATERNG.....
QXF31587.1 VSETSVEYFFSRSGLAGVVVVE.DATATNKG.....YATWEIDVMGFAQLRRKLEIFTYMRFDAEFTFVATERNG.....
pdb|6IIO|A VLETTINHFFSRSGLVGVVNLT.DGG.DTTG.....YATWDIDIMGFVQLRRKCEMFTYMRFNAEFTFVTTTKSG.....
pdb|6IIJ|A VLETTINHFFSRSGLVGVVNLT.DGG.DTTG.....YATWDIDIMGFVQLRRKCEMFTYMRFNAEFTFVTTTKSG.....
pdb|6AKS|A VLETTINHFFSRSGLVGVVNLT.DGGTDTTG.....YVTWDIDIMGFVQLRRKCEMFTYMRFNAEFTFVTTTKNG.....
BCD33944.1 VSETSIESFYGRSGLVTIITLEAGNQ..........RTNWLINFNEFVQLRAKMELFTYLRFDIEFTLVATLIKGGTA..
pdb|7ECY|A VSETLVENFLSRAALVSKRSFEYKDHTSSTARADKNFFKWTINTRSFVQLRRKLELFTYLRFDAEITILTTVAVNGSGNN
pdb|7EC5|A VSETLVENFLSRAALVSKRSFEYKDHTSSTARADKNFFKWTINTRSFVQLRRKLELFTYLRFDAEITILTTVAVNGSGNN
pdb|7EBZ|A VSETLVENFLSRAALVSKRSFEYKDHTSSTARADKNFFKWTINTRSFVQLRRKLELFTYLRFDAEITILTTVAVNGSGNN
pdb|7EBR|A VSETLVENFLSRAALVSKRSFEYKDHTSSTARADKNFFKWTINTRSFVQLRRKLELFTYLRFDAEITILTTVAVNGSGNN
pdb|6MZI|A VSETLVENFLSRAALVSKRSFEYKDHTSSTARADKNFFKWTINTRSFVQLRRKLELFTYLRFDAEITILTTVAVNGSGNN
pdb|6CSH|A VSETLVENFLSRAALVSKRSFEYKDHTSSTARADKNFFKWTINTRSFVQLRRKLELFTYLRFDAEITILTTVAVNGSGNN
pdb|6CSG|A VSETLVENFLSRAALVSKRSFEYKDHTSSTARADKNFFKWTINTRSFVQLRRKLELFTYLRFDAEITILTTVAVNGSGNN
pdb|6CSA|A VSETLVENFLSRAALVSKRSFEYKDHTSSTARADKNFFKWTINTRSFVQLRRKLELFTYLRFDAEITILTTVAVNGSGNN
pdb|6CS6|A VSETLVENFLSRAALVSKRSFEYKDHTSSTARADKNFFKWTINTRSFVQLRRKLELFTYLRFDAEITILTTVAVNGSGNN
pdb|6CS5|A VSETLVENFLSRAALVSKRSFEYKDHTSSTARADKNFFKWTINTRSFVQLRRKLELFTYLRFDAEITILTTVAVNGSGNN
pdb|6CS4|A VSETLVENFLSRAALVSKRSFEYKDHTSSTARADKNFFKWTINTRSFVQLRRKLELFTYLRFDAEITILTTVAVNGSGNN
pdb|6CS3|A VSETLVENFLSRAALVSKRSFEYKDHTSSTARADKNFFKWTINTRSFVQLRRKLELFTYLRFDAEITILTTVAVNGSGNN
pdb|6CRU|A VSETLVENFLSRAALVSKRSFEYKDHTSSTARADKNFFKWTINTRSFVQLRRKLELFTYLRFDAEITILTTVAVNGSGNN
pdb|6CRS|A VSETLVENFLSRAALVSKRSFEYKDHTSSTARADKNFFKWTINTRSFVQLRRKLELFTYLRFDAEITILTTVAVNGSGNN
pdb|6CRR|A VSETLVENFLSRAALVSKRSFEYKDHTSSTARADKNFFKWTINTRSFVQLRRKLELFTYLRFDAEITILTTVAVNGSGNN
pdb|6CRP|A VSETLVENFLSRAALVSKRSFEYKDHTSSTARADKNFFKWTINTRSFVQLRRKLELFTYLRFDAEITILTTVAVNGSGNN
QNO39042.1 VSETLVENFLSRAALVSKRSFEYKDHTSSAAQADKNFFKWTINTRSFVQLRRKLELFTYLRFDAEITILTTVAVNGSSNN
QNO39041.1 VSETLVENFLSRAALVSKRSFEYKDHTSSAAQADKNFFKWTINTRSFVQLRRKLELFTYLRFDAEITILTTVAVNGSSNN
QNO39040.1 VSETLVENFLSRAALVSKRSFEYKDHTSSAAQADKNFFKWTINTRSFVQLRRKLELFTYLRFDAEITILTTVAVNGSSNN
QNO39038.1 VSETLVENFLSRAALVSKRSFEYKDHTSSAAQADKNFFKWTINTRSFVQLRRKLELFTYLRFDAEITILTTVAVNGSSNN
QNO39039.1 VSETLVENFLSRAALVSKRSFEYKDHTSSAAQADKNFFKWTINTRSFVQLRRKLELFTYLRFDAEITILTTVAVNGSSNN
CAA1539354.1 VSETLVENFLSRAALVSKRSFEYKDHTSSAAQADKNFFKWTINTRSFVQLRRKLELFTYLRFDAEITILTTVAVNGSSNN
CAA1527138.1 VSETLVENFLSRAALVSKRSFEYKDHTSSAAQADKNFFKWTINTRSFVQLRRKLELFTYLRFDAEITILTTVAVNGSSNN
CAA1539353.1 VSETLVENFLSRAALVSKRSFEYKDHTSSAAQADKNFFKWTINTRSFVQLRRKLELFTYLRFDAEITILTTVAVNGGSNN
CAA1527136.1 VSETLVENFLSRAALVSKRSFEYKDHTSSAAQADKNFFKWTINTRSFVQLRRKLELFTYLRFDAEITILTTVAVNGGSNN
QNO39037.1 VSETLVENFLSRAALVSKRSFEYKDHTSSTAQTDKNFFKWTINTRSFVQLRRKLELFTYLRFDAEITILTTVAVNGSNNN
QKX95883.1 VSETLVENFLSRAALVSKRSFEYKDHTSSAAQADKNFFKWTINTRSFVQLRRKLELFTYLRFDAEITILTTVAVNGSSNN
pdb|5BNP|A VSETLVENFLGRAALVSKKSFEYKNHASSSAGTHKNFFKWTINTKSFVQLRRKLELFTYLRFDAEITILTTVAVNGNNDS
pdb|5BNO|A VSETLVENFLGRAALVSKKSFEYKNHASSSAGTHKNFFKWTINTKSFVQLRRKLELFTYLRFDAEITILTTVAVNGNNDS
pdb|5BNN|A VSETLVENFLGRAALVSKKSFEYKNHASSSAGTHKNFFKWTINTKSFVQLRRKLELFTYLRFDAEITILTTVAVNGNNDS
pdb|4WM8|A VSETLVENFLGRAALVSKKSFEYKNHASSSAGTHKNFFKWTINTKSFVQLRRKLELFTYLRFDAEITILTTVAVNGNNDS
pdb|4WM7|A VSETLVENFLGRAALVSKKSFEYKNHASSSAGTHKNFFKWTINTKSFVQLRRKLELFTYLRFDAEITILTTVAVNGNNDS
QIC50353.1 RSECTVESFFGRSACVAIIGLSNKKPTDTNG..KELFATWSISYLDTYQLRRKLEMFTYSRFDIEMTFVITERFFTSTSA
QIC50352.1 RSECTVESFFGRSACVAIIGLSNKKPTDTNG..KELFATWPISYLDTYQLRRKLEMFTYSRFDIEMTFVITERFFTSTSA
QIC50356.1 RSECTIESFFGRSACVAILSVTNKQPTATNG..NELFTTWRITYTQTYQLRRKLELFTYSRFDLELTFVITERFFTSNSA

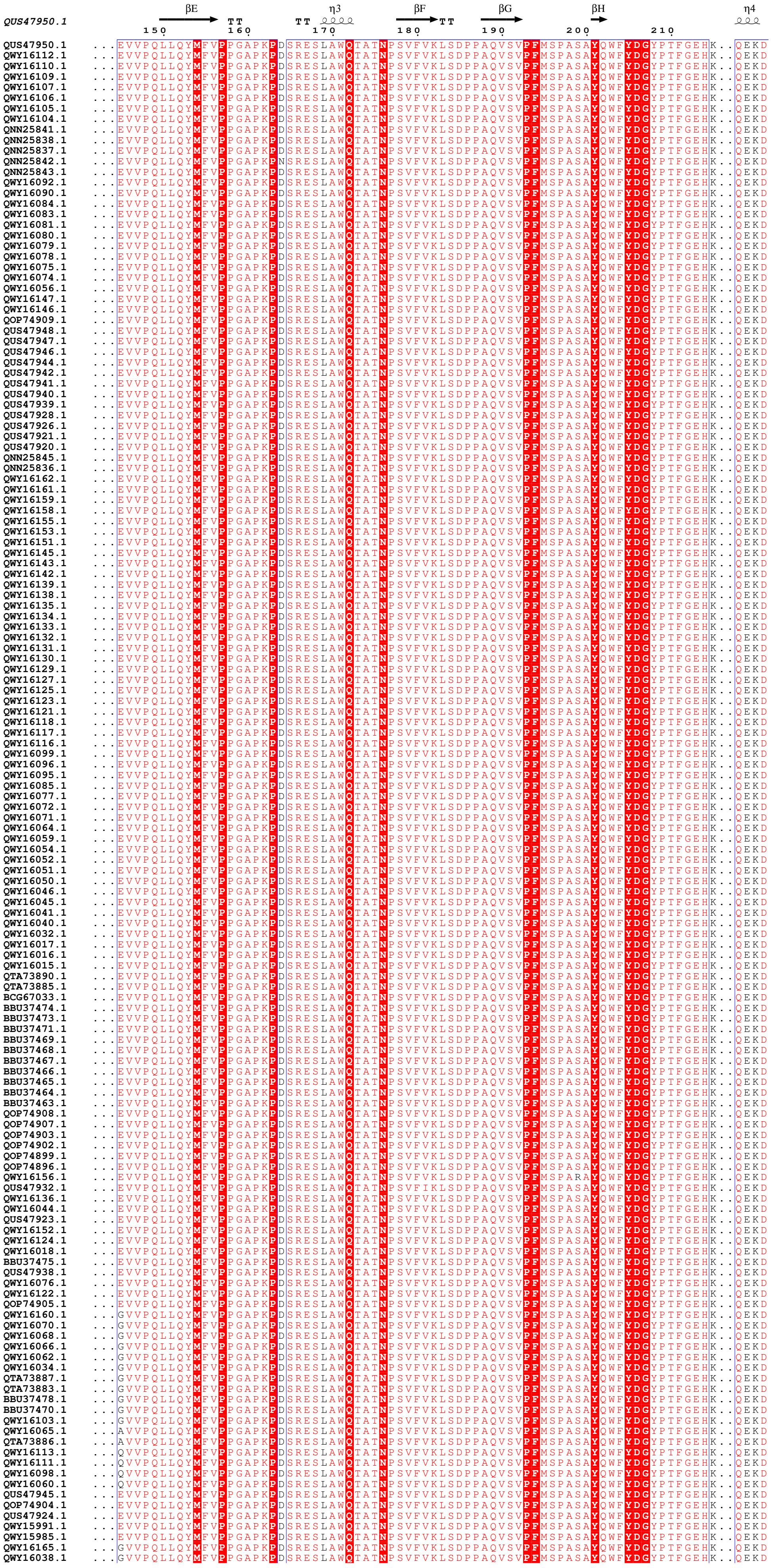
QUS47950.1
βE
βF
βG
βH
η3
η4
150
160
170
180
190
200
210

```
QWY16036.1     ...GVVPQLLQYMFVPPGAPKPDSRESLAWQTATNPSVFVKLSDPPAQVSVPFMSPASAYQWFYDGYPTFGEHK..QEKD
QWY16120.1     ...EVVPQLLQYMFVPPGAPKPDSRESLAWQTATNPSVFVKLSDPPAQVSVPFMSPASAYQWFYDGYPTFGEHK..QEKD
QWY16119.1     ...EVVPQLLQYMFVPPGAPKPDSRESLAWQTATNPSVFVKLSDPPAQVSVPFMSPASAYQWFYDGYPTFGEHK..QEKD
QWY16102.1     ...EVVPQLLQYMFVPPGAPKPDSRESLAWQTATNPSVFVKLSDPPAQVSVPFMSPASAYQWFYDGYPTFGEHK..QEKD
QWY16100.1     ...EVVPQLLQYMFVPPGAPKPDSRESLAWQTATNPSVFVKLSDPPAQVSVPFMSPASAYQWFYDGYPTFGEHK..QEKD
QWY16097.1     ...EVVPQLLQYMFVPPGAPKPDSRESLAWQTATNPSVFVKLSDPPAQVSVPFMSPASAYQWFYDGYPTFGEHK..QEKD
QWY16101.1     ...EVVPQLLQYMFVPPGAPKPDSRESLAWQTATNPSVFVKLSDPPAQVSVPFMSPASAYQWFYDGYPTFGEHK..QEKD
BBU37472.1     ...EVVPQLLQYMFVPPGAPKPDSRESLAWQTATNPSVFVKLSDPPAQVSVPFMSPASAYQWFYDGYPTFGEHK..QEKD
QWY16108.1     ...EVVPQLLQYMFVPPGAPKPDSRESLAWQTATNPSVFVKLSDPPAQVSVPFMSPASAYQWFYDGYPTFGEHK..QEKD
QWY16042.1     ...EVVPQLLQYMFVPPGAPKPDSRESLAWQTATNPSVFVKLSDPPAQVSVPFMSPASAYQWFYDGYPTFGEHK..QEKD
QOP74910.1     ...EVVPQLLQYMFVPPGAPKPDSRESLAWQTATNPSVFVKLSDPPAQVSVPFMSPASAYQWFYDGYPTFGEHK..QEKD
QWY16128.1     ...EVVPQLLQYMFVPPGAPKPDSRESLAWQTATNPSVFVKLPDPPAQVSVPFMSPASAYQWFYDGYPTFGEHK..QEKD
QUS47905.1     ...EVVPQLLQYMFVPPGAPKPDSRESLAWQTATNPSVFVKLSDPPAQVSVPFMSPASAYQWFYDGYPTFGEHK..QEKD
QWY16154.1     ...EVVPQLLQYMFVPPGAPKPESRESLAWQTATNPSVFVKLSDPPAQVSVPFMSPASAYQWFYDGYPTFGEHK..QEKD
QWY16126.1     ...EVVPQLLQYMFVPPGAPKPESRESLAWQTATNPSVFVKLSDPPAQVSVPFMSPASAYQWFYDGYPTFGEHK..QEKD
QWY16049.1     ...EVVPQLLQYMFVPPGAPKPESRESLAWQTATNPSVFVKLSDPPAQVSVPFMSPASAYQWFYDGYPTFGEHK..QEKD
QWY16043.1     ...EVVPQLLQYMFVPPGAPKPESRESLAWQTATNPSVFVKLSDPPAQVSVPFMSPASAYQWFYDGYPTFGEHK..QEKD
QUS47911.1     ...EVVPQLLQYMFVPPGAPKPDSRESLAWQTATNPSVFVKLSDPPAQVSVPFMSPASAYQWFYDGYPTFGEHK..QEKD
BBU37477.1     ...EVVPQLLQYMFVPPGAPKPDSRESLAWQTATNPSVFVKLSDPPAQVSVPFMSPASAYQWFYDGYPTFGEHK..QEKD
QWY16137.1     ...EVVPQLLQYMFVPPGAPKPDSRESLAWQTATNPSVFVKLSDPPAQVSVPFMSPASAYQWFYDGYPTFGEHK..QEKD
QWY16061.1     ...EVVPQLLQYMFVPPGAPKPDSRESLAWQTATNPSVFVKLSDPPAQVSVPFMSPASAYQWFYDGYPTFGEHK..QEKD
QTA73882.1     ...EVVPQLLQYMFVPPGAPKPDSRESLAWQTATNPSVFVKLSDPPAQVSVPFMSPASAYQWFYDGYPTFGEHK..QEKD
QWY16031.1     ...EVVPQLLQYMFVPPGAPKPDSRESLAWQTATNPSVFVKLSDPPAQVSVPFMSPASAYQWFYDGYPTFGEHK..QEKD
BBU37476.1     ...EVVPQLLQYMFVPPGAPKPDSRESLAWQTATNPSVFVKLSDPPAQVSVPFMSPASAYQWFYDGYPTFGEHK..QEKD
QUS47949.1     ...EVVPQLLQYMFVPPGAPKPDSRESLAWQTATNPSVFVKLSDPPAQVSVPFMSPASAYQWFYDGYPTFGEHK..QEKD
QNN25833.1     ...EVVPQLLQYMFVPPGAPKPDSRESLAWQTATNPSVFVKLSDPPAQVSVPFMSPASAYQWFYDGYPTFGEHK..QEKD
QWY16114.1     ...AVVPQLLQYMFVPPGAPKPDSRESFAWQTATNPSVFVKLSDPPAQVSVPFMSPASAYQWFYDGYPTFGEHK..QEKD
QWY16063.1     ...GVVPQLLQYMFVPPGAPKPDSRESLAWQTATNPSVFVKLSDPPAQVSVPFMSPASAYQWFYDGYPTFGEHK..QEKD
QTA73884.1     ...GVVPQLLQYMFVPPGAPKPDSRESLAWQTATNPSVFVKLSDPPAQVSVPFMSPASAYQWFYDGYPTFGEHK..QEKD
QWY16002.1     ...GVVPQLLQYMFVPPGAPKPDSRESLAWQTATNPSVFVKLSDPPAQVSVPFMSPASAYQWFYDGYPTFGEHK..QEKD
QWY15984.1     ...EVVPQLLQYMFVPPGAPKPDSRESLAWQTATNPSVFVKLSDPPAQVSVPFMSPASAYQWFYDGYPTFGEHK..QEKD
QUS47934.1     ...EVVPQLLQYMFVPPGAPKPDSRESLAWQTATNPSVFVKLSDPPAQVSVPFMSPASAYQWFYDGYPTFGEHK..QEKD
QUS47933.1     ...EVVPQLLQYMFVPPGAPKPDSRESLAWQTATNPSVFVKLSDPPAQVSVPFMSPASAYQWFYDGYPTFGEHK..QEKD
QUS47929.1     ...EVVPQLLQYMFVPPGAPKPDSRESLAWQTATNPSVFVKLSDPPAQVSVPFMSPASAYQWFYDGYPTFGEHK..QEKD
QUS47927.1     ...EVVPQLLQYMFVPPGAPKPDSRESLAWQTATNPSVFVKLSDPPAQVSVPFMSPASAYQWFYDGYPTFGEHK..QEKD
QUS47919.1     ...EVVPQLLQYMFVPPGAPKPDSRESLAWQTATNPSVFVKLSDPPAQVSVPFMSPASAYQWFYDGYPTFGEHK..QEKD
QUS47917.1     ...EVVPQLLQYMFVPPGAPKPDSRESLAWQTATNPSVFVKLSDPPAQVSVPFMSPASAYQWFYDGYPTFGEHK..QEKD
QUS47915.1     ...EVVPQLLQYMFVPPGAPKPDSRESLAWQTATNPSVFVKLSDPPAQVSVPFMSPASAYQWFYDGYPTFGEHK..QEKD
QUS47913.1     ...EVVPQLLQYMFVPPGAPKPDSRESLAWQTATNPSVFVKLSDPPAQVSVPFMSPASAYQWFYDGYPTFGEHK..QEKD
QWY16166.1     ...EVVPQLLQYMFVPPGAPKPDSRESLAWQTATNPSVFVKLSDPPAQVSVPFMSPASAYQWFYDGYPTFGEHK..QEKD
QWY16164.1     ...EVVPQLLQYMFVPPGAPKPDSRESLAWQTATNPSVFVKLSDPPAQVSVPFMSPASAYQWFYDGYPTFGEHK..QEKD
QWY16163.1     ...EVVPQLLQYMFVPPGAPKPDSRESLAWQTATNPSVFVKLSDPPAQVSVPFMSPASAYQWFYDGYPTFGEHK..QEKD
QWY16157.1     ...EVVPQLLQYMFVPPGAPKPDSRESLAWQTATNPSVFVKLSDPPAQVSVPFMSPASAYQWFYDGYPTFGEHK..QEKD
QWY16150.1     ...EVVPQLLQYMFVPPGAPKPDSRESLAWQTATNPSVFVKLSDPPAQVSVPFMSPASAYQWFYDGYPTFGEHK..QEKD
QWY16115.1     ...EVVPQLLQYMFVPPGAPKPDSRESLAWQTATNPSVFVKLSDPPAQVSVPFMSPASAYQWFYDGYPTFGEHK..QEKD
QWY16087.1     ...EVVPQLLQYMFVPPGAPKPDSRESLAWQTATNPSVFVKLSDPPAQVSVPFMSPASAYQWFYDGYPTFGEHK..QEKD
QWY16069.1     ...EVVPQLLQYMFVPPGAPKPDSRESLAWQTATNPSVFVKLSDPPAQVSVPFMSPASAYQWFYDGYPTFGEHK..QEKD
QWY16058.1     ...EVVPQLLQYMFVPPGAPKPDSRESLAWQTATNPSVFVKLSDPPAQVSVPFMSPASAYQWFYDGYPTFGEHK..QEKD
QWY16048.1     ...EVVPQLLQYMFVPPGAPKPDSRESLAWQTATNPSVFVKLSDPPAQVSVPFMSPASAYQWFYDGYPTFGEHK..QEKD
QWY16047.1     ...EVVPQLLQYMFVPPGAPKPDSRESLAWQTATNPSVFVKLSDPPAQVSVPFMSPASAYQWFYDGYPTFGEHK..QEKD
QWY16030.1     ...EVVPQLLQYMFVPPGAPKPDSRESLAWQTATNPSVFVKLSDPPAQVSVPFMSPASAYQWFYDGYPTFGEHK..QEKD
QWY16027.1     ...EVVPQLLQYMFVPPGAPKPDSRESLAWQTATNPSVFVKLSDPPAQVSVPFMSPASAYQWFYDGYPTFGEHK..QEKD
QWY16021.1     ...EVVPQLLQYMFVPPGAPKPDSRESLAWQTATNPSVFVKLSDPPAQVSVPFMSPASAYQWFYDGYPTFGEHK..QEKD
QWY16020.1     ...EVVPQLLQYMFVPPGAPKPDSRESLAWQTATNPSVFVKLSDPPAQVSVPFMSPASAYQWFYDGYPTFGEHK..QEKD
QWY16009.1     ...EVVPQLLQYMFVPPGAPKPDSRESLAWQTATNPSVFVKLSDPPAQVSVPFMSPASAYQWFYDGYPTFGEHK..QEKD
QWY16008.1     ...EVVPQLLQYMFVPPGAPKPDSRESLAWQTATNPSVFVKLSDPPAQVSVPFMSPASAYQWFYDGYPTFGEHK..QEKD
QWY16007.1     ...EVVPQLLQYMFVPPGAPKPDSRESLAWQTATNPSVFVKLSDPPAQVSVPFMSPASAYQWFYDGYPTFGEHK..QEKD
QWY16001.1     ...EVVPQLLQYMFVPPGAPKPDSRESLAWQTATNPSVFVKLSDPPAQVSVPFMSPASAYQWFYDGYPTFGEHK..QEKD
QWY16000.1     ...EVVPQLLQYMFVPPGAPKPDSRESLAWQTATNPSVFVKLSDPPAQVSVPFMSPASAYQWFYDGYPTFGEHK..QEKD
QWY15993.1     ...EVVPQLLQYMFVPPGAPKPDSRESLAWQTATNPSVFVKLSDPPAQVSVPFMSPASAYQWFYDGYPTFGEHK..QEKD
QWY15986.1     ...EVVPQLLQYMFVPPGAPKPDSRESLAWQTATNPSVFVKLSDPPAQVSVPFMSPASAYQWFYDGYPTFGEHK..QEKD
QWY15979.1     ...EVVPQLLQYMFVPPGAPKPDSRESLAWQTATNPSVFVKLSDPPAQVSVPFMSPASAYQWFYDGYPTFGEHK..QEKD
QTA73889.1     ...EVVPQLLQYMFVPPGAPKPDSRESLAWQTATNPSVFVKLSDPPAQVSVPFMSPASAYQWFYDGYPTFGEHK..QEKD
QTA73874.1     ...EVVPQLLQYMFVPPGAPKPDSRESLAWQTATNPSVFVKLSDPPAQVSVPFMSPASAYQWFYDGYPTFGEHK..QEKD
QTA73871.1     ...EVVPQLLQYMFVPPGAPKPDSRESLAWQTATNPSVFVKLSDPPAQVSVPFMSPASAYQWFYDGYPTFGEHK..QEKD
QOP74901.1     ...EVVPQLLQYMFVPPGAPKPDSRESLAWQTATNPSVFVKLSDPPAQVSVPFMSPASAYQWFYDGYPTFGEHK..QEKD
QOP74900.1     ...EVVPQLLQYMFVPPGAPKPDSRESLAWQTATNPSVFVKLSDPPAQVSVPFMSPASAYQWFYDGYPTFGEHK..QEKD
QOP74897.1     ...EVVPQLLQYMFVPPGAPKPDSRESLAWQTATNPSVFVKLSDPPAQVSVPFMSPASAYQWFYDGYPTFGEHK..QEKD
QOP74893.1     ...EVVPQLLQYMFVPPGAPKPDSRESLAWQTATNPSVFVKLSDPPAQVSVPFMSPASAYQWFYDGYPTFGEHK..QEKD
QOP74887.1     ...EVVPQLLQYMFVPPGAPKPDSRESLAWQTATNPSVFVKLSDPPAQVSVPFMSPASAYQWFYDGYPTFGEHK..QEKD
QOP74886.1     ...EVVPQLLQYMFVPPGAPKPDSRESLAWQTATNPSVFVKLSDPPAQVSVPFMSPASAYQWFYDGYPTFGEHK..QEKD
QOP74884.1     ...EVVPQLLQYMFVPPGAPKPDSRESLAWQTATNPSVFVKLSDPPAQVSVPFMSPASAYQWFYDGYPTFGEHK..QEKD
QOP74882.1     ...EVVPQLLQYMFVPPGAPKPDSRESLAWQTATNPSVFVKLSDPPAQVSVPFMSPASAYQWFYDGYPTFGEHK..QEKD
QOP74881.1     ...EVVPQLLQYMFVPPGAPKPDSRESLAWQTATNPSVFVKLSDPPAQVSVPFMSPASAYQWFYDGYPTFGEHK..QEKD
QOP74880.1     ...EVVPQLLQYMFVPPGAPKPDSRESLAWQTATNPSVFVKLSDPPAQVSVPFMSPASAYQWFYDGYPTFGEHK..QEKD
QOP74879.1     ...EVVPQLLQYMFVPPGAPKPDSRESLAWQTATNPSVFVKLSDPPAQVSVPFMSPASAYQWFYDGYPTFGEHK..QEKD
QOP74878.1     ...EVVPQLLQYMFVPPGAPKPDSRESLAWQTATNPSVFVKLSDPPAQVSVPFMSPASAYQWFYDGYPTFGEHK..QEKD
pdb|4YVS|M     ...EVVPQLLQYMFVPPGAPKPDSRESLAWQTATNPSVFVKLSDPPAQVSVPFMSPASAYQWFYDGYPTFGEHK..QEKD
pdb|4YVS|J     ...EVVPQLLQYMFVPPGAPKPDSRESLAWQTATNPSVFVKLSDPPAQVSVPFMSPASAYQWFYDGYPTFGEHK..QEKD
pdb|4YVS|G     ...EVVPQLLQYMFVPPGAPKPDSRESLAWQTATNPSVFVKLSDPPAQVSVPFMSPASAYQWFYDGYPTFGEHK..QEKD
pdb|4YVS|D     ...EVVPQLLQYMFVPPGAPKPDSRESLAWQTATNPSVFVKLSDPPAQVSVPFMSPASAYQWFYDGYPTFGEHK..QEKD
pdb|4YVS|A     ...EVVPQLLQYMFVPPGAPKPDSRESLAWQTATNPSVFVKLSDPPAQVSVPFMSPASAYQWFYDGYPTFGEHK..QEKD
pdb|4CEY|A     ...EVVPQLLQYMFVPPGAPKPDSRESLAWQTATNPSVFVKLSDPPAQVSVPFMSPASAYQWFYDGYPTFGEHK..QEKD
pdb|4CEW|A     ...EVVPQLLQYMFVPPGAPKPDSRESLAWQTATNPSVFVKLSDPPAQVSVPFMSPASAYQWFYDGYPTFGEHK..QEKD
pdb|4N53|A     ...EVVPQLLQYMFVPPGAPKPDSRESLAWQTATNPSVFVKLSDPPAQVSVPFMSPASAYQWFYDGYPTFGEHK..QEKD
pdb|4N43|A     ...EVVPQLLQYMFVPPGAPKPDSRESLAWQTATNPSVFVKLSDPPAQVSVPFMSPASAYQWFYDGYPTFGEHK..QEKD
pdb|4RQP|A     ...EVVPQLLQYMFVPPGAPKPDSRESLAWQTATNPSVFVKLSDPPAQVSVPFMSPASAYQWFYDGYPTFGEHK..QEKD
pdb|4RQP|M     ...EVVPQLLQYMFVPPGAPKPDSRESLAWQTATNPSVFVKLSDPPAQVSVPFMSPASAYQWFYDGYPTFGEHK..QEKD
pdb|4RQP|I     ...EVVPQLLQYMFVPPGAPKPDSRESLAWQTATNPSVFVKLSDPPAQVSVPFMSPASAYQWFYDGYPTFGEHK..QEKD
pdb|4RQP|Q     ...EVVPQLLQYMFVPPGAPKPDSRESLAWQTATNPSVFVKLSDPPAQVSVPFMSPASAYQWFYDGYPTFGEHK..QEKD
pdb|4RQP|E     ...EVVPQLLQYMFVPPGAPKPDSRESLAWQTATNPSVFVKLSDPPAQVSVPFMSPASAYQWFYDGYPTFGEHK..QEKD
QWY16033.1     ...EVVPQLLQYMFVPPGAPKPDSRESLAWQTATNPSVFVKLSDPPAQVSVPFMSPASAYQWFYDGYPTFGEHK..QEKD
QWY16029.1     ...EVVPQLLQYMFVPPGAPKPDSRESLAWQTATNPSVFVKLSDPPAQVSVPFMSPASAYQWFYDGYPTFGEHK..QEKD
QWY16028.1     ...EVVPQLLQYMFVPPGAPKPDSRESLAWQTATNPSVFVKLSDPPAQVSVPFMSPASAYQWFYDGYPTFGEHK..QEKD
QWY16025.1     ...EVVPQLLQYMFVPPGAPKPDSRESLAWQTATNPSVFVKLSDPPAQVSVPFMSPASAYQWFYDGYPTFGEHK..QEKD
QUS47943.1     ...EVVPQLLQYMFVPPGAPKPDSRESLAWQTATNPSVFVKLSDPPAQVSVPFMSPASAYQWFYDGYPTFGEHK..QEKD
QOP74914.1     ...EVVPQLLQYMFVPPGAPKPDSRESLAWQTATNPSVFVKLSDPPAQVSVPFMSPASAYQWFYDGYPTFGEHK..QEKD
QUS47916.1     ...EVVPQLLQYMFVPPGAPKPDSRESLAWQTATNPSVFVKLSDPPAQVSVPFMSPASAYQWFYDGYPTFGEHK..QEKD
QWY16148.1     ...EVVPQLLQYMFVPPGAPKPDSRESLAWQTATNPSVFVKLSDPPAQVSVPFMSPASAYQWFYDGYPTFGEHK..QEKD
QOP74906.1     ...EVVPQLLQYMFVPPGAPKPDSRESLAWQTATNPSVFVKLSDPPAQVSVPFMSPASAYQWFYDGYPTFGEHK..QEKD
QOP74895.1     ...EVVPQLLQYMFVPPGAPKPDSRESLAWQTATNPSVFVKLSDPPAQVSVPFMSPASAYQWFYDGYPTFGEHK..QEKD
QOP74890.1     ...EVVPQLLQYMFVPPGAPKPDSRESLAWQTATNPSVFVKLSDPPAQVSVPFMSPASAYQWFYDGYPTFGEHK..QEKD
QOP74889.1     ...EVVPQLLQYMFVPPGAPKPDSRESLAWQTATNPSVFVKLSDPPAQVSVPFMSPASAYQWFYDGYPTFGEHK..QEKD
QWY16149.1     ...EVVPQLLQYMFVPPGAPKPDSRESLAWQTATNPSVFVKLSDPPAQVLVPFMSPASAYQWFYDGYPTFGEHK..QEKD
QOP74888.1     ...EVVPQLLQYMFVPPGAPKPDSRESLAWQTATNPSVFVKLSDPPAQVSVPFMSPASAYQWFYDGYPTFGEHK..QEKD
QOP74894.1     ...EVVPQLLQYMFVPPGAPKPDSRESLAWQTATNPSVFVKLSDPPAQVSVPFMSPASAYQWFYDGYPTFGEHK..QEKD
QUS47930.1     ...EVVPQLLQYMFVPPGAPKPDSRESLAWQTATNPSVFVKLSDPPAQVSVPFMSPASAYQWFYDGYPTFGEHK..QEKD
QWY16053.1     ...EVVPQLLQYMFVPPGAPKPDSRESLAWQTATNPSVFVKLSDPPAQVSVPFMSPASAYQWFYDGYPTFGEHK..QEKD
QWY15997.1     ...EVVPQLLQYMFVPPGAPKPDSRESLAWQTATNPSVFVKLSDPPAQVSVPFMSPASAYQWFYDGYPTFGEHK..QEKD
QWY15996.1     ...EVVPQLLQYMFVPPGAPKPDSRESLAWQTATNPSVFVKLSDPPAQVSVPFMSPASAYQWFYDGYPTFGEHK..QEKD
QWY15995.1     ...EVVPQLLQYMFVPPGAPKPDSRESLAWQTATNPSVFVKLSDPPAQVSVPFMSPASAYQWFYDGYPTFGEHK..QEKD
QTA73878.1     ...EVVPQLLQYMFVPPGAPKPDSRESLAWQTATNPSVFVKLSDPPAQVSVPFMSPASAYQWFYDGYPTFGEHK..QEKD
QTA73877.1     ...EVVPQLLQYMFVPPGAPKPDSRESLAWQTATNPSVFVKLSDPPAQVSVPFMSPASAYQWFYDGYPTFGEHK..QEKD
QTA73876.1     ...EVVPQLLQYMFVPPGAPKPDSRESLAWQTATNPSVFVKLSDPPAQVSVPFMSPASAYQWFYDGYPTFGEHK..QEKD
QUS47914.1     ...GVVPQLLQYMFVPPGAPKPDSRESLAWQTATNPSVFVKLSDPPAQVSVPFMSPASAYQWFYDGYPTFGEHK..QEKD
QWY16003.1     ...GVVPQLLQYMFVPPGAPKPDSRESLAWQTATNPSVFVKLSDPPAQVSVPFMSPASAYQWFYDGYPTFGEHK..QEKD
QWY16067.1     ...GVVPQLLQYMFVPPGAPKPDSRESLAWQTATNPSVFVKLSDPPAQVSVPFMSPASAYQWFYDGYPTFGEHK..QEKD
QWY16057.1     ...GVVPQLLQYMFVPPGAPKPDSRESLAWQTATNPSVFVKLSDPPAQVSVPFMSPASAYQWFYDGYPTFGEHK..QEKD
QTA73888.1     ...GVVPQLLQYMFVPPGAPKPDSRESLAWQTATNPSVFVKLSDPPAQVSVPFMSPASAYQWFYDGYPTFGEHK..QEKD
QWY16086.1     ...GVVPQLLQYMFVPPGAPKPDSRESLAWQTATNPSVFVKLSDPPAQVSVPFMSPASAYQWFYDGYPTFGEHK..QEKD
pdb|4GMP|1     ...GVVPQLLQYMFVPPGAPKPDSRESLAWQTATNPSVFVKLSDPPAQVSVPFMSPASAYQWFYDGYPTFGEHK..QEKD
pdb|3J91|1     ...GVVPQLLQYMFVPPGAPKPDSRESLAWQTATNPSVFVKLSDPPAQVSVPFMSPASAYQWFYDGYPTFGEHK..QEKD
QWY16094.1     ...EVVPQLLQYMFVPPGAPKPDSRESLAWQTATNPSVFVKLSDPPAQVSVPFMSPASAYQWFYDGYPTFGEHK..QEKD
QWY16093.1     ...EVVPQLLQYMFVPPGAPKPDSRESLAWQTATNPSVFVKLSDPPAQVSVPFMSPASAYQWFYDGYPTFGEHK..QEKD
QWY16091.1     ...EVVPQLLQYMFVPPGAPKPDSRESLAWQTATNPSVFVKLSDPPAQVSVPFMSPASAYQWFYDGYPTFGEHK..QEKD
QWY16088.1     ...EVVPQLLQYMFVPPGAPKPDSRESLAWQTATNPSVFVKLSDPPAQVSVPFMSPASAYQWFYDGYPTFGEHK..QEKD
QWY16082.1     ...EVVPQLLQYMFVPPGAPKPDSRESLAWQTATNPSVFVKLSDPPAQVSVPFMSPASAYQWFYDGYPTFGEHK..QEKD
QWY16073.1     ...EVVPQLLQYMFVPPGAPKPDSRESLAWQTATNPSVFVKLSDPPAQVSVPFMSPASAYQWFYDGYPTFGEHK..QEKD
QWY16055.1     ...EVVPQLLQYMFVPPGAPKPDSRESLAWQTATNPSVFVKLSDPPAQVSVPFMSPASAYQWFYDGYPTFGEHK..QEKD
QUS47918.1     ...EVVPQLLQYMFVPPGAPKPDSRESLAWQTATNPSVFVKLSDPPAQVSVPFMSPASAYQWFYDGYPTFGEHK..QEKD
QWY16089.1     ...EVVPQLLQYMFVPPGAPKPDSRGSLAWQTATNPSVFVKLSDPPAQVSVPFMSPASAYQWFYDGYPTFGEHK..QEKD
QWY16037.1     ...EVVPQLLQYMFVPPGAPKPNSRESLAWQTATNPSVFVKLSDPPAQVSVPFMSPASAYQWFYDGYPTFGEHK..QEKD
QOP74891.1     ...EVVPQLLQYMFVPPGAPKPNSRESLAWQTATNPSVFVKLSDPPAQVSVPFMSPASAYQWFYDGYPTFGEHK..QEKD
pdb|3VBS|A     ...EVVPQLLQYMFVPPGAPKPDSRESLAWQTATNPSVFVKLSDPPAQVSVPFMSPASAYQWFYDGYPTFGEHK..QEKD
pdb|3VBO|A     ...EVVPQLLQYMFVPPGAPKPDSRESLAWQTATNPSVFVKLSDPPAQVSVPFMSPASAYQWFYDGYPTFGEHK..QEKD
pdb|3VBH|A     ...EVVPQLLQYMFVPPGAPKPDSRESLAWQTATNPSVFVKLSDPPAQVSVPFMSPASAYQWFYDGYPTFGEHK..QEKD
pdb|3VBF|A     ...EVVPQLLQYMFVPPGAPKPDSRESLAWQTATNPSVFVKLSDPPAQVSVPFMSPASAYQWFYDGYPTFGEHK..QEKD
pdb|6LQD|A     ...EVVPQLLQYMFVPPGAPKPDSRESLAWQTATNPSVFVKLSDPPAQVSVPFMSPASAYQWFYDGYPTFGEHK..QEKD
pdb|5ZUF|A     ...EVVPQLLQYMFVPPGAPKPDSRESLAWQTATNPSVFVKLSDPPAQVSVPFMSPASAYQWFYDGYPTFGEHK..QEKD
pdb|5ZUD|A     ...EVVPQLLQYMFVPPGAPKPDSRESLAWQTATNPSVFVKLSDPPAQVSVPFMSPASAYQWFYDGYPTFGEHK..QEKD
pdb|4CDX|A     ...EVVPQLLQYMFVPPGAPKPDSRESLAWQTATNPSVFVKLSDPPAQVSVPFMSPASAYQWFYDGYPTFGEHK..QEKD
pdb|4CDW|A     ...EVVPQLLQYMFVPPGAPKPDSRESLAWQTATNPSVFVKLSDPPAQVSVPFMSPASAYQWFYDGYPTFGEHK..QEKD
pdb|4CDU|A     ...EVVPQLLQYMFVPPGAPKPDSRESLAWQTATNPSVFVKLSDPPAQVSVPFMSPASAYQWFYDGYPTFGEHK..QEKD
pdb|4CDQ|A     ...EVVPQLLQYMFVPPGAPKPDSRESLAWQTATNPSVFVKLSDPPAQVSVPFMSPASAYQWFYDGYPTFGEHK..QEKD
```

```
QOP74885.1     ...EVVPQLLQYMFVPPGAPKPDSRESLAWQTATNPSVFVKLSDPPAQVSVPFMSPASAYQWFYDGYPTFGEHK..QEKD
QWY16026.1     ...EVVPQLLQYMFVPPGAPKPDSRKSLAWQTATNPSVFVKLSDPPAQVSVPFMSPASAYQWFYDGYPTFGEHK..QEKD
QWY15977.1     ...EVVPQLLQYMFVPPGAPKPDSRESLAGQTATNPSVFVKLSDPPAQVSVPFMSPASAYQWFYDGYPTFGEHK..QEKD
QTA73869.1     ...EVVPQLLQYMFVPPGAPKPDSRESLAGQTATNPSVFVKLSDPPAQVSVPFMSPASAYQWFYDGYPTFGEHK..QEKD
QUS47935.1     ...EVVPQLLQYMFVPPGAPKPDSRESLAWQTATNPSVFVKLSDPPAQVSVPFMSPASAYQWFYDGYPTFGEHK..QEKD
QWY16024.1     ...EVVPQLLQYMFVPPGAPKPDSRESLAWQTATNPSVFVKLSDPPAQVSVPFMSPASAYQWFYDGYPTFGEHK..QEKD
QWY16023.1     ...EVVPQLLQYMFVPPGAPKPDSRESLAWQTATNPSVFVKLSDPPAQVSVPFMSPASAYQWFYDGYPTFGEHK..QEKD
QWY16022.1     ...EVVPQLLQYMFVPPGAPKPDSRESLAWQTATNPSVFVKLSDPPAQVSVPFMSPASAYQWFYDGYPTFGEHK..QEKD
QWY16013.1     ...EVVPQLLQYMFVPPGAPKPDSRESLAWQTATNPSVFVKLSDPPAQVSVPFMSPASAYQWFYDGYPTFGEHK..QEKD
QWY16012.1     ...EVVPQLLQYMFVPPGAPKPDSRESLAWQTATNPSVFVKLSDPPAQVSVPFMSPASAYQWFYDGYPTFGEHK..QEKD
QWY15992.1     ...EVVPQLLQYMFVPPGAPKPDSRESLAWQTATNPSVFVKLSDPPAQVSVPFMSPASAYQWFYDGYPTFGEHK..QEKD
QWY15982.1     ...EVVPQLLQYMFVPPGAPKPDSRESLAWQTATNPSVFVKLSDPPAQVSVPFMSPASAYQWFYDGYPTFGEHK..QEKD
QWY15981.1     ...EVVPQLLQYMFVPPGAPKPDSRESLAWQTATNPSVFVKLSDPPAQVSVPFMSPASAYQWFYDGYPTFGEHK..QEKD
QWY15976.1     ...EVVPQLLQYMFVPPGAPKPDSRESLAWQTATNPSVFVKLSDPPAQVSVPFMSPASAYQWFYDGYPTFGEHK..QEKD
QTA73880.1     ...EVVPQLLQYMFVPPGAPKPDSRESLAWQTATNPSVFVKLSDPPAQVSVPFMSPASAYQWFYDGYPTFGEHK..QEKD
QTA73872.1     ...EVVPQLLQYMFVPPGAPKPDSRESLAWQTATNPSVFVKLSDPPAQVSVPFMSPASAYQWFYDGYPTFGEHK..QEKD
QTA73868.1     ...EVVPQLLQYMFVPPGAPKPDSRESLAWQTATNPSVFVKLSDPPAQVSVPFMSPASAYQWFYDGYPTFGEHK..QEKD
QOP74892.1     ...EVVPQLLQYMFVPPGAPKPDSRESLAWQTATNPSVFVKLSDPPAQVSVPFMSPASAYQWFYDGYPTFGEHK..QEKD
QWY15998.1     ...EVVPQLLQYMFVPPGAPKPDSRESLAWQTATNPSVFVKLSDPPAQVSVPFMSPASAYQWFYDGYPTFGEHK..QEKD
QWY15994.1     ...EVVPQLLQYMFVPPGAPKPDSRESLAWQTATNPSVFVKLSDPPAQVSVPFMSPASAYQWFYDGYPTFGEHK..QEKD
QTA73879.1     ...EVVPQLLQYMFVPPGAPKPDSRESLAWQTATNPSVFVKLSDPPAQVSVPFMSPASAYQWFYDGYPTFGEHK..QEKD
QTA73875.1     ...EVVPQLLQYMFVPPGAPKPDSRESLAWQTATNPSVFVKLSDPPAQVSVPFMSPASAYQWFYDGYPTFGEHK..QEKD
QWY16006.1     ...EVVPQLLQYMFVPPGAPKPDSRESLAWQTATNPSVFVKLSDPPAQVSVPFMSPASAYQWFYDGYPTFGEHK..QEKD
QWY16005.1     ...EVVPQLLQYMFVPPGAPKPDSRESLAWQTATNPSVFVKLSDPPAQVSVPFMSPASAYQWFYDGYPTFGEHK..QEKD
QWY16039.1     ...QVVPQLLQYMFVPPGAPKPDSRESLAWQTATNPSVFVKLSDPPAQVSVPFMSPASAYQWFYDGYPTFGEHK..QEKD
QWY15999.1     ...QVVPQLLQYMFVPPGAPKPDSRESLAWQTATNPSVFVKLSDPPAQVSVPFMSPASAYQWFYDGYPTFGEHK..QEKD
QWY16004.1     ...QVVPQLLQYMFVPPGAPKPDSRESLAWQTATNPSVFVKLSDPPAQVSVPFMSPASAYQWFYDGYPTFGEHK..QEKD
QOP74913.1     ...QVVPQLLQYMFVPPGAPKPDSRESLAWQTATNPSVFVKLSDPPAQVSVPFMSPASAYQWFYDGYPTFGEHK..QEKD
QOP74915.1     ...QVVPQLLQYMFVPPGAPKPDSRESLAWQTATNPSVFVKLSDPPAQVSVPFMSPASAYQWFYDGYPTFGEHK..QEKD
QWY16035.1     ...QVVPQLLQYMFVPPGAPKPNSRESLAWQTATNPSVFVKLSDPPAQVSVPFMSPASAYQWFYDGYPTFGEHK..QEKD
QWY15990.1     ...QVVPQLLQYMFVPPGAPKPDSRESLAWQTATNPSVFVKLSDPPAQVSVPFMSPASAYQWFYDGYPTFGEHK..QEKD
QWY15988.1     ...QVVPQLLQYMFVPPGAPKPDSRESLAWQTATNPSVFVKLSDPPAQVSVPFMSPASAYQWFYDGYPTFGEHK..QEKD
QWY15989.1     ...EVVPQLLQYMFVPPGAPKPDSRESLAWQTATNPSVFVKLSDPPAQVSVPFMSPASAYQWFYDGYPTFGEHK..QEKD
QWY15987.1     ...EVVPQLLQYMFVPPGAPKPDSRESLAWQTATNPSVFVKLSDPPAQVSVPFMSPASAYQWFYDGYPTFGEHK..QEKD
QWY15978.1     ...EVVPQLLQYMFVPPGAPKPDSRESLAWQTATNPSVFVKLSDPPAQVSVPFMSPASAYQWFYDGYPTFGEHK..QEKD
QTA73870.1     ...EVVPQLLQYMFVPPGAPKPDSRESLAWQTATNPSVFVKLSDPPAQVSVPFMSPASAYQWFYDGYPTFGEHK..QEKD
QWY15983.1     ...EVVPQLLQYMFVPPGAPKPDSRESLAWQTATNPSVFVKLSDPPAQVSVPFMSPASAYQWFYDGYPTFGEHK..QEKD
QUS47931.1     ...EVVPQLLQYMFVPPGAPKPDSRESLAWQTATNPSVFVKLSDPPAQVSVPFMSPASAYQWFYDGYPTFGEHK..QEKD
QUS47925.1     ...EVVPQLLQYMFVPPGAPKPDSRESLAWQTATNPSVFVKLSDPPAQVSVPFMSPASAYQWFYDGYPTFGEHK..QEKD
QUS47922.1     ...EVVPQLLQYMFVPPGAPKPDSRESLAWQTATNPSVFVKLSDPPAQVSVPFMSPASAYQWFYDGYPTFGEHK..QEKD
QWY16014.1     ...EVVPQLLQYMFVPPGAPKPDSRESLAWQTATNPSVFVKLSDPPAQVSVPFMSPASAYQWFYDGYPTFGEHK..QEKD
QTA73881.1     ...EVVPQLLQYMFVPPGAPKPDSRESLAWQTATNPSVFVKLSDPPAQVSVPFMSPASAYQWFYDGYPTFGEHK..QEKD
QOP74898.1     ...EVVPQLLQYMFVPPGAPKPDSRESLAWQTATNPSVFVKLSDPPAQVSVPFMSPASAYQWFYDGYPTFGEHK..QEKD
QWY15980.1     ...EVVPQLLQYMFVPPGAPKPDSRESLAWQTATNPSVFVKLSDPPAQVSVPFMSPASAYQWFYDGYPTFGEHK..QEKD
QTA73873.1     ...EVVPQLLQYMFVPPGAPKPDSRESLAWQTATNPSVFVKLSDPPAQVSVPFMSPASAYQWFYDGYPTFGEHK..QEKD
QWY16011.1     ...EVVPQLLQYMFVPPGAPKPDSRESLAWQTATNPSVFVKLSDPPAQVSVPFMSPASAYQWFYDGYPTFGEHK..QEKD
QWY16010.1     ...EVVPQLLQYMFVPPGAPKPDSRESLAWQTATNPSVFVKLSDPPAQVSVPFMSPASAYQWFYDGYPTFGEHK..QEKD
QOP74911.1     ...EVVPQLLQYMFVPPGAPKPDSRESLAWQTATNPSVFVKLSDPPAQVSVPFMSPASAYQWFYDGYPTFGEHK..QEKD
QWY16019.1     ...EVVPQLLQYMFVPPGAPKPDSRESLAWQTATNPSVFVKLSDPPAQVSVPFMSPASAYQWFYDGYPTFGEHK..QEKD
QOP74883.1     ...EVVPQLLQYMFVPPGAPKPDSRESLAWQTATNPSVFVKLSDPPAQVSVPFMSPASAYQWFYDGYPTFGEHK..QEKD
QUS47909.1     ...EVVPQLLQYMFVPPGAPKPDSRESLAWQTATNPSVFVKLSDPPAQVSVPFMSPASAYQWFYDGYPTFGEHK..QEKD
QWY16144.1     ...EVVPQLLQYMFVPPGAPKPDSRESLAWQTATNPSVFVKLSDPPAQVSVPFMSPASAYQWFYDGYPTFGEHK..QEKD
QUS47908.1     ...EVVPQLLQYMFVPPGAPKPDSRESLAWQTATNPSVFVKLSDPPAQVSVPFMSPASAYQWFYDGYPTFGEHK..QEKD
QNN25844.1     ...EVVPQLLQYMFVPPGAPKPDSRESLAWQTATNPSVFVKLSDPPAQVSVPFMSPASAYQWFYDGYPTFGEHK..QEKD
QNN25840.1     ...EVVPQLLQYMFVPPGAPKPDSRESLAWQTATNPSVFVKLSDPPAQVSVPFMSPASAYQWFYDGYPTFGEHK..QEKD
QNN25839.1     ...EVVPQLLQYMFVPPGAPKPDSRESLAWQTATNPSVFVKLSDPPAQVSVPFMSPASAYQWFYDGYPTFGEHK..QEKD
QNN25835.1     ...EVVPQLLQYMFVPPGAPKPDSRESLAWQTATNPSVFVKLSDPPAQVSVPFMSPASAYQWFYDGYPTFGEHK..QEKD
QNN25834.1     ...EVVPQLLQYMFVPPGAPKPDSRESLAWQTATNPSVFVKLSDPPAQVSVPFMSPASAYQWFYDGYPTFGEHK..QEKD
QOP74912.1     ...EVVPQLLQYMFVPPGAPKPDSRESLAWQTATNPSVFVKLSDPPAQVSVPFMSPASAYQWFYDGYPTFGEHK..QEKD
QUS47910.1     ...EVVPQLLQYMYVPTGAPKPDSRESLAWQTATNPSVFVKLSDPPAQVSVPFMSPASAYQWFYDGYPTFGEHK..QEKD
QUS47907.1     ...EVVPQLLQYMYVPPGAPKPDSRESLAWQTATNPSVFVKLSDPPAQVSVPFMSPASAYQWFYDGYPTFGEHK..QEKD
QWY16141.1     ...EVVPQLLQYMFVPPGAPKPDSRESLAWQTATNPSVFVKLSDPPAQVSVPFMSPASAYQWFYDGYPTFGEHK..QEKD
QWY16140.1     ...EVVPQLLQYMFVPPGAPKPDSRESLAWQTATNPSVFVKLSDPPAQVSVPFMSPASAYQWFYDGYPTFGEHK..QEKD
QUS47937.1     ...EVVPQLLQYMFVPPGAPKPDSRESLAWQTATNPSVFVKLSDPPAQVSVPFMSPASAYQWFYDGYPTFGEHK..QEKD
QUS47936.1     ...EVVPQLLQYMFVPPGAPKPDSRESLAWQTATNPSVFVKLSDPPAQVSVPFMSPASAYQWFYDGYPTFGEHK..QEKD
QUS47912.1     ...KVVPQLLQYMYVPPGAPKPDSRESLAWQTATNPSVFVKLSDPPAQVSVPFMSPASAYQWFYDGYPTFGEHK..QEKD
QUS47906.1     ...EVVPQLLQYMYVPPRRPKPDSRESLAWQTATNPSVFVKLSDPPAQVSVPFMSPASAYQWFYDGYPTFGEHK..QEKD
pdb|4YVW|M     ...EVVPQLLQYMFVPPGAPKPDSRESLAWQTATNPSVFVKLSDPPAQVSVPFMSPASAYQWFYDGYPTFGEHL..QAND
pdb|4YVW|J     ...EVVPQLLQYMFVPPGAPKPDSRESLAWQTATNPSVFVKLSDPPAQVSVPFMSPASAYQWFYDGYPTFGEHL..QAND
pdb|4YVW|D     ...EVVPQLLQYMFVPPGAPKPDSRESLAWQTATNPSVFVKLSDPPAQVSVPFMSPASAYQWFYDGYPTFGEHL..QAND
pdb|4YVW|A     ...EVVPQLLQYMFVPPGAPKPDSRESLAWQTATNPSVFVKLSDPPAQVSVPFMSPASAYQWFYDGYPTFGEHL..QAND
pdb|4YVW|E     ...EVVPQLLQYMFVPPGAPKPDSRESLAWQTATNPSVFVKLSDPPAQVSVPFMSPASAYQWFYDGYPTFGEHL..QAND
QPI19797.1     ...QVVPQLLQYMFVPPGAPKPDSRESLAWQTATNPSVFVKLTDPPAQVSVPFMSPASAYQWFYDGYPTFGEHK..QEKD
QPI19781.1     ...QVVPQLLQYMFVPPGAPKPDSRESLAWQTATNPSVFVKLTDPPAQVSVPFMSPASAYQWFYDGYPTFGEHK..QEKD
QPI19795.1     ...QVVPQLLQYMFVPPGAPKPDSRESLAWQTATNPSVFVKLTDPPAQVSVPFMSPASAYQWFYDGYPTFGEHK..QEKD
QPI19794.1     ...QVVPQLLQYMFVPPGAPKPDSRESLAWQTATNPSVFVKLTDPPAQVSVPFMSPASAYQWFYDGYPTFGEHK..QEKD
QPI19793.1     ...QVVPQLLQYMFVPPGAPKPDSRESLAWQTATNPSVFVKLTDPPAQVSVPFMSPASAYQWFYDGYPTFGEHK..QEKD
QPI19792.1     ...QVVPQLLQYMFVPPGAPKPDSRESLAWQTATNPSVFVKLTDPPAQVSVPFMSPASAYQWFYDGYPTFGEHK..QEKD
QPI19790.1     ...QVVPQLLQYMFVPPGAPKPDSRESLAWQTATNPSVFVKLTDPPAQVSVPFMSPASAYQWFYDGYPTFGEHK..QEKD
QPI19789.1     ...QVVPQLLQYMFVPPGAPKPDSRESLAWQTATNPSVFVKLTDPPAQVSVPFMSPASAYQWFYDGYPTFGEHK..QEKD
QPI19788.1     ...QVVPQLLQYMFVPPGAPKPDSRESLAWQTATNPSVFVKLTDPPAQVSVPFMSPASAYQWFYDGYPTFGEHK..QEKD
QPI19787.1     ...QVVPQLLQYMFVPPGAPKPDSRESLAWQTATNPSVFVKLTDPPAQVSVPFMSPASAYQWFYDGYPTFGEHK..QEKD
QPI19785.1     ...QVVPQLLQYMFVPPGAPKPDSRESLAWQTATNPSVFVKLTDPPAQVSVPFMSPASAYQWFYDGYPTFGEHK..QEKD
QPI19784.1     ...QVVPQLLQYMFVPPGAPKPDSRESLAWQTATNPSVFVKLTDPPAQVSVPFMSPASAYQWFYDGYPTFGEHK..QEKD
QPI19782.1     ...QVVPQLLQYMFVPPGAPKPDSRESLAWQTATNPSVFVKLTDPPAQVSVPFMSPASAYQWFYDGYPTFGEHK..QEKD
QPI19780.1     ...QVVPQLLQYMFVPPGAPKPDSRESLAWQTATNPSVFVKLTDPPAQVSVPFMSPASAYQWFYDGYPTFGEHK..QEKD
QPI19777.1     ...QVVPQLLQYMFVPPGAPKPDSRESLAWQTATNPSVFVKLTDPPAQVSVPFMSPASAYQWFYDGYPTFGEHK..QEKD
QPI19762.1     ...QVVPQLLQYMFVPPGAPKPDSRESLAWQTATNPSVFVKLTDPPAQVSVPFMSPASAYQWFYDGYPTFGEHK..QEKD
QPI19755.1     ...QVVPQLLQYMFVPPGAPKPDSRESLAWQTATNPSVFVKLTDPPAQVSVPFMSPASAYQWFYDGYPTFGEHK..QEKD
QPI19754.1     ...QVVPQLLQYMFVPPGAPKPDSRESLAWQTATNPSVFVKLTDPPAQVSVPFMSPASAYQWFYDGYPTFGEHK..QEKD
QPI19748.1     ...QVVPQLLQYMFVPPGAPKPDSRESLAWQTATNPSVFVKLTDPPAQVSVPFMSPASAYQWFYDGYPTFGEHK..QEKD
QPI19747.1     ...QVVPQLLQYMFVPPGAPKPDSRESLAWQTATNPSVFVKLTDPPAQVSVPFMSPASAYQWFYDGYPTFGEHK..QEKD
QPI19744.1     ...QVVPQLLQYMFVPPGAPKPDSRESLAWQTATNPSVFVKLTDPPAQVSVPFMSPASAYQWFYDGYPTFGEHK..QEKD
QPI19743.1     ...QVVPQLLQYMFVPPGAPKPDSRESLAWQTATNPSVFVKLTDPPAQVSVPFMSPASAYQWFYDGYPTFGEHK..QEKD
QPI19739.1     ...QVVPQLLQYMFVPPGAPKPDSRESLAWQTATNPSVFVKLTDPPAQVSVPFMSPASAYQWFYDGYPTFGEHK..QEKD
QPI19778.1     ...QVVPQLLQYMFVPPGAPKPDSRESLAWQTATNPSIFVKLTDPPAQVSVPFMSPASAYQWFYDGYPTFGEHK..QEKD
QPI19796.1     ...QVVPQLLQYMFVPPGAPKPDSRESLAWQTATNPSVFVKLTDPPAQVSVPFMSPASAYQWFYDGYPTFGEHK..QEKD
QPI19753.1     ...QVVPQLLQYMFVPPGAPKPDSRESLAWQTATNPSVFVKLTDPPAQVSVPFMSPASAYQWFYDGYPTFGEHK..QEKD
QPI19791.1     ...QVVPQLLQYMFVPPGAPKPDSRESLAWQTATNPSVFVKLTDPPAQVSVPFMSPASAYQWFYDGYPTFGEHK..QEKD
QPI19786.1     ...QVVPQLLQYMFVPPGAPKPDSRESLAWQTATNPSVFVKLTDPPAQVSVPFMSPASAYQWFYDGYPTFGEHK..QEKD
QPI19779.1     ...QVVPQLLQYMFVPPGAPKPDSRESLAWQTATNPSVFVKLTDPPAQVSVPFMSPASAYQWFYDGYPTFGEHK..QEKD
QPI19742.1     ...QVVPQLLQYMFVPPGAPKPDSRESLAWQTATNPSVFVKLTDPPAQVSVPFMSPASAYQWFYDGYPTFGEHK..QEKD
pdb|4AED|A     ...QVVPQLLQYMFVPPGAPKPESRESLAWQTATNPSVFVKLTDPPAQVSVPFMSPASAYQWFYDGYPTFGEHK..QEKD
pdb|3ZFF|A     ...QVVPQLLQYMFVPPGAPKPESRESLAWQTATNPSVFVKLTDPPAQVSVPFMSPASAYQWFYDGYPTFGEHK..QEKD
pdb|6DIZ|A     ...QVVPQLLQYMFVPPGAPKPESRESLAWQTATNPSVFVKLTDPPAQVSVPFMSPASAYQWFYDGYPTFGEHK..QEKD
pdb|6UH7|A     ...QVVPQLLQYMFVPPGAPKPESRESLAWQTATNPSVFVKLTDPPAQVSVPFMSPASAYQWFYDGYPTFGEHK..QEKD
pdb|6UH6|A     ...QVVPQLLQYMFVPPGAPKPESRESLAWQTATNPSVFVKLTDPPAQVSVPFMSPASAYQWFYDGYPTFGEHK..QEKD
pdb|6UH1|A     ...QVVPQLLQYMFVPPGAPKPESRESLAWQTATNPSVFVKLTDPPAQVSVPFMSPASAYQWFYDGYPTFGEHK..QEKD
QPI19783.1     ...QVVPQLLQYMFVPPGAPKPGSRESLAWQTATNPSVFVKLTDPPAQVSVPFMSPASAYQWFYDGYPTFGEHK..QEKD
QPI19772.1     ...GVVPQLLQYMFVPPGAPKPDSRESLAWQTATNPSVFVKLTDPPAQVSVPFMSPASAYQWFYDGYPTFGEHK..QEKD
QPI19771.1     ...GVVPQLLQYMFVPPGAPKPDSRESLAWQTATNPSVFVKLTDPPAQVSVPFMSPASAYQWFYDGYPTFGEHK..QEKD
QPI19764.1     ...GVVPQLLQYMFVPPGAPKPDSRESLAWQTATNPSVFVKLTDPPAQVSVPFMSPASAYQWFYDGYPTFGEHK..QEKD
QPI19760.1     ...GVVPQLLQYMFVPPGAPKPDSRESLAWQTATNPSVFVKLTDPPAQVSVPFMSPASAYQWFYDGYPTFGEHK..QEKD
QPI19759.1     ...GVVPQLLQYMFVPPGAPKPDSRESLAWQTATNPSVFVKLTDPPAQVSVPFMSPASAYQWFYDGYPTFGEHK..QEKD
QPI19756.1     ...GVVPQLLQYMFVPPGAPKPDSRESLAWQTATNPSVFVKLTDPPAQVSVPFMSPASAYQWFYDGYPTFGEHK..QEKD
QPI19752.1     ...GVVPQLLQYMFVPPGAPKPDSRESLAWQTATNPSVFVKLTDPPAQVSVPFMSPASAYQWFYDGYPTFGEHK..QEKD
QPI19740.1     ...GVVPQLLQYMFVPPGAPKPDSRESLAWQTATNPSVFVKLTDPPAQVSVPFMSPASAYQWFYDGYPTFGEHK..QEKD
QPI19738.1     ...GVVPQLLQYMFVPPGAPKPDSRESLAWQTATNPSVFVKLTDPPAQVSVPFMSPASAYQWFYDGYPTFGEHK..QEKD
QPI19757.1     ...GVVPQLLQYMFVPPGAPKPDSRESLAWQTATNPSVFVKLTDPPAQVSVPFMSPASAYQWFYDGYPTFGEHK..QEKD
QPI19750.1     ...GVVPQLLQYMFVPPGAPKPDSRESLAWQTATNPSVFVKLTDPPAQVSVPFMSPASAYQWFYDGYPTFGEHK..QEKD
QPI19751.1     ...GVVPQLLQYMFVPPGAPKPDSRESLAWQTATNPSVFVKLTDPPAQVSVPFMSPASAYQWFYDGYPTFGEHK..QEKD
QPI19746.1     ...GVVPQLLQYMFVPPGAPKPDSRESLAWQTATNPSVFVKLTDPPAQVSVPFMSPASAYQWFYDGYPTFGEHK..QEKD
QPI19737.1     ...AVVPQLLQYMFVPPGAPKPDSRESLAWQTATNPSVFVKLTDPPAQVSVPFMSPASAYQWFYDGYPTFGEHK..QEKD
QPI19776.1     ...EVVPQLLQYMFVPPGAPKPDSRESLAWQTATNPSVFVKLTDPPAQVSVPFMSPASAYQWFYDGYPTFGEHK..QEKD
QPI19774.1     ...EVVPQLLQYMFVPPGAPKPDSRESLAWQTATNPSVFVKLTDPPAQVSVPFMSPASAYQWFYDGYPTFGEHK..QEKD
QPI19773.1     ...EVVPQLLQYMFVPPGAPKPDSRESLAWQTATNPSVFVKLTDPPAQVSVPFMSPASAYQWFYDGYPTFGEHK..QEKD
QPI19770.1     ...EVVPQLLQYMFVPPGAPKPDSRESLAWQTATNPSVFVKLTDPPAQVSVPFMSPASAYQWFYDGYPTFGEHK..QEKD
QPI19769.1     ...EVVPQLLQYMFVPPGAPKPDSRESLAWQTATNPSVFVKLTDPPAQVSVPFMSPASAYQWFYDGYPTFGEHK..QEKD
QPI19768.1     ...EVVPQLLQYMFVPPGAPKPDSRESLAWQTATNPSVFVKLTDPPAQVSVPFMSPASAYQWFYDGYPTFGEHK..QEKD
QPI19767.1     ...EVVPQLLQYMFVPPGAPKPDSRESLAWQTATNPSVFVKLTDPPAQVSVPFMSPASAYQWFYDGYPTFGEHK..QEKD
QPI19766.1     ...EVVPQLLQYMFVPPGAPKPDSRESLAWQTATNPSVFVKLTDPPAQVSVPFMSPASAYQWFYDGYPTFGEHK..QEKD
QPI19763.1     ...EVVPQLLQYMFVPPGAPKPDSRESLAWQTATNPSVFVKLTDPPAQVSVPFMSPASAYQWFYDGYPTFGEHK..QEKD
QPI19761.1     ...EVVPQLLQYMFVPPGAPKPDSRESLAWQTATNPSVFVKLTDPPAQVSVPFMSPASAYQWFYDGYPTFGEHK..QEKD
QPI19765.1     ...EVVPQLLQYMFVPPGAPKPDSRESLAWQTATNPSVFVKLTDPPAQVSVPFMSPASAYQWFYDGYPTFGEHK..QEKD
QPI19775.1     ...EVVPQLLQYMFVPPGAPKPDSRESLAWQTATNPSVFVKLTDPPAQVSVPFMSPASAYQWFYDGYPTFGEHK..QEKD
QPI19758.1     ...QVVPQLLQYMFVPPGAPKPDSRESLAWQTATNPSVFVKLTDPPAQVSVPFMSPASAYQWFYDGYPTFGEHK..QEKD
QPI19736.1     ...QVVPQLLQYMFVPPGAPKPDSRESLAWQTATNPSVFVKLTDPPAQVSVPFMSPASAYQWFYDGYPTFGEHK..QEKD
QPI19735.1     ...QVVPQLLQYMFVPPGAPKPDSRESLAWQTATNPSVFVKLTDPPAQVSVPFMSPASAYQWFYDGYPTFGEHK..QEKD
QPI19749.1     ...QVVPQLLQYMFVPPGAPKPDSRESLAWQTATNPSVFVKLTDPPAQVSVPFMSPASAYQWFYDGYPTFGEHK..QEKD
QPI19741.1     ...EVVPQLLQYMFVPPGAPKPDSRESLAWQTATNPSVFVKLTDPPAQVSVPFMSPASAYQWFYDGYPTFGEHK..QEKD
QPI19745.1     ...QVVPQLLQYMFVPPGAPKPDSRESLAWQTATNPSVFVKLTDPPAQVSVPFMSPASAYQWFYDGYPTFGEHK..QEKD
pdb|6Z3Q|A     ...QVVPQLLQYMFVPPGAPKPESRESLAWQTATNPSVFVKLTDPPAQVSVPFMSPASAYQWFYDGYPTFGEHK..QEKD
pdb|6Z3P|A     ...QVVPQLLQYMFVPPGAPKPESRESLAWQTATNPSVFVKLTDPPAQVSVPFMSPASAYQWFYDGYPTFGEHK..QEKD
pdb|6I2K|A     ...QVVPQLLQYMFVPPGAPKPESRESLAWQTATNPSVFVKLTDPPAQVSVPFMSPASAYQWFYDGYPTFGEHK..QEKD
```

```
QWF08740.1   ...ELVPQLLQYMYVPPGAPKPTSRDSFAWQTATNPSVFVKMTDPPAQVSVPFMSPASAYQWFYDGYPTFGEHL..QAND
QWF08739.1   ...ELVPQLLQYMYVPPGAPKPTSRDSFAWQTATNPSVFVKMTDPPAQVSVPFMSPASAYQWFYDGYPTFGEHL..QAND
QWF08760.1   ...ELVPQLLQYMYVPPGAPKPTSRDSFAWQTATNPSVFVKMTDPPAQVSVPFMSPASAYQWFYDGYPTFGEHL..QAND
QWF08759.1   ...ELVPQLLQYMYVPPGAPKPTSRDSFAWQTATNPSVFVKMTDPPAQVSVPFMSPASAYQWFYDGYPTFGEHL..QAND
QWF08762.1   ...ELVPQLLQYMYVPPGAPKPTSRDSFAWQTATNPSVFVKMTDPPAQVSVPFMSPASAYQWFYDGYPTFGEHL..QAND
QWF08761.1   ...ELVPQLLQYMYVPPGAPKPTSRDSFAWQTATNPSVFVKMTDPPAQVSVPFMSPASAYQWFYDGYPTFGEHL..QAND
QWF08764.1   ...ELVPQLLQYMYVPPGAPKPTSRDSFAWQTATNPSVFVKMTDPPAQVSVPFMSPASAYQWFYDGYPTFGEHL..QAND
QWF08763.1   ...ELVPQLLQYMYVPPGAPKPTSRDSFAWQTATNPSVFVKMTDPPAQVSVPFMSPASAYQWFYDGYPTFGEHL..QAND
QWF08773.1   ...ELVPQLLQYMYVPPGAPKPTSRDSFAWQTATNPSVFVKMTDPPAQVSVPFMSPASAYQWFYDGYPTFGEHL..QAND
QWF08749.1   ...ELVPQLLQYMYVPPGAPKPTSRDSFAWQTATNPSVFVKMTDPPAQVSVPFMSPASAYQWFYDGYPTFGEHL..QAND
QWF08785.1   ...ELVPQLLQYMYVPPGAPKPTSRDSFAWQTATNPSVFVKMTDPPAQVSVPFMSPASAYQWFYDGYPTFGEHL..QAND
QWF08783.1   ...ELVPQLLQYMYVPPGAPKPTSRDSFAWQTATNPSVFVKMTDPPAQVSVPFMSPASAYQWFYDGYPTFGEHL..QAND
QWF08787.1   ...ELVPQLLQYMYVPPGAPKPTSRDSFAWQTATNPSVFVKMTDPPAQVSVPFMSPASAYQWFYDGYPTFGEHL..QAND
QWF08765.1   ...ELVPQLLQYMYVPPGAPKPTSRDSFAWQTATNPSVFVKMTDPPAQVSVPFMSPASAYQWFYDGYPTFGEHL..QAND
QWF08792.1   ...ELVPQLLQYMYVPPGAPKPTSRDSFAWQTATNPSVFVKMTDPPAQVSVPFMSPASAYQWFYDGYPTFGEHL..QAND
QWF08767.1   ...ELVPQLLQYMYVPPGAPKPTSRDSFAWQTATNPSVFVKMTDPPAQVSVPFMSPASAYQWFYDGYPTFGEHL..QAND
QWF08793.1   ...ELVPQLLQYMYVPPGAPKPTSRDSFAWQTATNPSVFVKMTDPPAQVSVPFMSPASAYQWFYDGYPTFGEHL..QAND
QWF08758.1   ...ELVPQLLQYMYVPPGAPKPTSRDSFAWQTATNPSVFVKMTDPPAQVSVPFMSPASAYQWFYDGYPTFGEHL..QAND
QWF08845.1   ...ELVPQLLQYMYVPPGAPKPTSRDSFAWQTATNPSVFVKMTDPPAQVSVPFMSPASAYQWFYDGYPTFGEHL..QAND
QWF08844.1   ...ELVPQLLQYMYVPPGAPKPTSRDSFAWQTATNPSVFVKMTDPPAQVSVPFMSPASAYQWFYDGYPTFGEHL..QAND
QWF08847.1   ...ELVPQLLQYMYVPPGAPKPTSRDSFAWQTATNPSVFVKMTDPPAQVSVPFMSPASAYQWFYDGYPTFGEHL..QAND
QWF08846.1   ...ELVPQLLQYMYVPPGAPKPTSRDSFAWQTATNPSVFVKMTDPPAQVSVPFMSPASAYQWFYDGYPTFGEHL..QAND
QWF08852.1   ...ELVPQLLQYMYVPPGAPKPTSRDSFAWQTATNPSVFVKMTDPPAQVSVPFMSPASAYQWFYDGYPTFGEHL..QAND
QWF08843.1   ...ELVPQLLQYMYVPPGAPKPTSRDSFAWQTATNPSVFVKMTDPPAQVSVPFMSPASAYQWFYDGYPTFGEHL..QAND
QWF08857.1   ...ELVPQLLQYMYVPPGAPKPTSRDSFAWQTATNPSVFVKMTDPPAQVSVPFMSPASAYQWFYDGYPTFGEHL..QAND
QWF08856.1   ...ELVPQLLQYMYVPPGAPKPTSRDSFAWQTATNPSVFVKMTDPPAQVSVPFMSPASAYQWFYDGYPTFGEHL..QAND
QWF08865.1   ...ELVPQLLQYMYVPPGAPKPTSRDSFAWQTATNPSVFVKMTDPPAQVSVPFMSPASAYQWFYDGYPTFGEHL..QAND
QWF08738.1   ...ELVPQLLQYMYVPPGAPKPTSRDSFAWQTATNPSVFVKMTDPPAQVSVPFMSPASAYQWFYDGYPTFGEHL..QAND
QWF08872.1   ...ELVPQLLQYMYVPPGAPKPTSRDSFAWQTATNPSVFVKMTDPPAQVSVPFMSPASAYQWFYDGYPTFGEHL..QAND
QWF08871.1   ...ELVPQLLQYMYVPPGAPKPTSRDSFAWQTATNPSVFVKMTDPPAQVSVPFMSPASAYQWFYDGYPTFGEHL..QAND
QWF08879.1   ...ELVPQLLQYMYVPPGAPKPTSRDSFAWQTATNPSVFVKMTDPPAQVSVPFMSPASAYQWFYDGYPTFGEHL..QAND
QWF08877.1   ...ELVPQLLQYMYVPPGAPKPTSRDSFAWQTATNPSVFVKMTDPPAQVSVPFMSPASAYQWFYDGYPTFGEHL..QAND
QWF08880.1   ...ELVPQLLQYMYVPPGAPKPTSRDSFAWQTATNPSVFVKMTDPPAQVSVPFMSPASAYQWFYDGYPTFGEHL..QAND
QWF08851.1   ...ELVPQLLQYMYVPPGAPKPTSRDSFAWQTATNPSVFVKMTDPPAQVSVPFMSPASAYQWFYDGYPTFGEHL..QAND
QWF08882.1   ...ELVPQLLQYMYVPPGAPKPTSRDSFAWQTATNPSVFVKMTDPPAQVSVPFMSPASAYQWFYDGYPTFGEHL..QAND
QWF08867.1   ...ELVPQLLQYMYVPPGAPKPTSRDSFAWQTATNPSVFVKMTDPPAQVSVPFMSPASAYQWFYDGYPTFGEHL..QAND
QWF08884.1   ...ELVPQLLQYMYVPPGAPKPTSRDSFAWQTATNPSVFVKMTDPPAQVSVPFMSPASAYQWFYDGYPTFGEHL..QAND
QWF08875.1   ...ELVPQLLQYMYVPPGAPKPTSRDSFAWQTATNPSVFVKMTDPPAQVSVPFMSPASAYQWFYDGYPTFGEHL..QAND
QWF08885.1   ...ELVPQLLQYMYVPPGAPKPTSRDSFAWQTATNPSVFVKMTDPPAQVSVPFMSPASAYQWFYDGYPTFGEHL..QAND
QWF08863.1   ...ELVPQLLQYMYVPPGAPKPTSRDSFAWQTATNPSVFVKMTDPPAQVSVPFMSPASAYQWFYDGYPTFGEHL..QAND
QWF08890.1   ...ELVPQLLQYMYVPPGAPKPTSRDSFAWQTATNPSVFVKMTDPPAQVSVPFMSPASAYQWFYDGYPTFGEHL..QAND
QWF08889.1   ...ELVPQLLQYMYVPPGAPKPTSRDSFAWQTATNPSVFVKMTDPPAQVSVPFMSPASAYQWFYDGYPTFGEHL..QAND
QWF08896.1   ...ELVPQLLQYMYVPPGAPKPTSRDSFAWQTATNPSVFVKMTDPPAQVSVPFMSPASAYQWFYDGYPTFGEHL..QAND
QWF08752.1   ...ELVPQLLQYMYVPPGAPKPTSRDSFAWQTATNPSVFVKMTDPPAQVSVPFMSPASAYQWFYDGYPTFGEHL..QAND
QWF08900.1   ...ELVPQLLQYMYVPPGAPKPTSRDSFAWQTATNPSVFVKMTDPPAQVSVPFMSPASAYQWFYDGYPTFGEHL..QAND
QWF08899.1   ...ELVPQLLQYMYVPPGAPKPTSRDSFAWQTATNPSVFVKMTDPPAQVSVPFMSPASAYQWFYDGYPTFGEHL..QAND
QWF08908.1   ...ELVPQLLQYMYVPPGAPKPTSRDSFAWQTATNPSVFVKMTDPPAQVSVPFMSPASAYQWFYDGYPTFGEHL..QAND
QWF08906.1   ...ELVPQLLQYMYVPPGAPKPTSRDSFAWQTATNPSVFVKMTDPPAQVSVPFMSPASAYQWFYDGYPTFGEHL..QAND
QWF08910.1   ...ELVPQLLQYMYVPPGAPKPTSRDSFAWQTATNPSVFVKMTDPPAQVSVPFMSPASAYQWFYDGYPTFGEHL..QAND
QWF08870.1   ...ELVPQLLQYMYVPPGAPKPTSRDSFAWQTATNPSVFVKMTDPPAQVSVPFMSPASAYQWFYDGYPTFGEHL..QAND
QWF08915.1   ...ELVPQLLQYMYVPPGAPKPTSRDSFAWQTATNPSVFVKMTDPPAQVSVPFMSPASAYQWFYDGYPTFGEHL..QAND
QWF08750.1   ...ELVPQLLQYMYVPPGAPKPTSRDSFAWQTATNPSVFVKMTDPPAQVSVPFMSPASAYQWFYDGYPTFGEHL..QAND
QWF08917.1   ...ELVPQLLQYMYVPPGAPKPTSRDSFAWQTATNPSVFVKMTDPPAQVSVPFMSPASAYQWFYDGYPTFGEHL..QAND
QWF08916.1   ...ELVPQLLQYMYVPPGAPKPTSRDSFAWQTATNPSVFVKMTDPPAQVSVPFMSPASAYQWFYDGYPTFGEHL..QAND
QWF08923.1   ...ELVPQLLQYMYVPPGAPKPTSRDSFAWQTATNPSVFVKMTDPPAQVSVPFMSPASAYQWFYDGYPTFGEHL..QAND
QWF08921.1   ...ELVPQLLQYMYVPPGAPKPTSRDSFAWQTATNPSVFVKMTDPPAQVSVPFMSPASAYQWFYDGYPTFGEHL..QAND
QWF08926.1   ...ELVPQLLQYMYVPPGAPKPTSRDSFAWQTATNPSVFVKMTDPPAQVSVPFMSPASAYQWFYDGYPTFGEHL..QAND
QWF08780.1   ...ELVPQLLQYMYVPPGAPKPTSRDSFAWQTATNPSVFVKMTDPPAQVSVPFMSPASAYQWFYDGYPTFGEHL..QAND
QWF08932.1   ...ELVPQLLQYMYVPPGAPKPTSRDSFAWQTATNPSVFVKMTDPPAQVSVPFMSPASAYQWFYDGYPTFGEHL..QAND
QWF08931.1   ...ELVPQLLQYMYVPPGAPKPTSRDSFAWQTATNPSVFVKMTDPPAQVSVPFMSPASAYQWFYDGYPTFGEHL..QAND
QWF08945.1   ...ELVPQLLQYMYVPPGAPKPTSRDSFAWQTATNPSVFVKMTDPPAQVSVPFMSPASAYQWFYDGYPTFGEHL..QAND
QWF08938.1   ...ELVPQLLQYMYVPPGAPKPTSRDSFAWQTATNPSVFVKMTDPPAQVSVPFMSPASAYQWFYDGYPTFGEHL..QAND
QWF08955.1   ...ELVPQLLQYMYVPPGAPKPTSRDSFAWQTATNPSVFVKMTDPPAQVSVPFMSPASAYQWFYDGYPTFGEHL..QAND
QWF08954.1   ...ELVPQLLQYMYVPPGAPKPTSRDSFAWQTATNPSVFVKMTDPPAQVSVPFMSPASAYQWFYDGYPTFGEHL..QAND
QWF08967.1   ...ELVPQLLQYMYVPPGAPKPTSRDSFAWQTATNPSVFVKMTDPPAQVSVPFMSPASAYQWFYDGYPTFGEHL..QAND
QWF08959.1   ...ELVPQLLQYMYVPPGAPKPTSRDSFAWQTATNPSVFVKMTDPPAQVSVPFMSPASAYQWFYDGYPTFGEHL..QAND
QYC65038.1   ...ELVPQLLQYMYVPPGAPKPTSRDSFAWQTATNPSVFVKMTDPPAQVSVPFMSPASAYQWFYDGYPTFGEHL..QAND
QYC65037.1   ...ELVPQLLQYMYVPPGAPKPTSRDSFAWQTATNPSVFVKMTDPPAQVSVPFMSPASAYQWFYDGYPTFGEHL..QAND
QYC65039.1   ...ELVPQLLQYMYVPPGAPKPTSRDSFAWQTATNPSVFVKMTDPPAQVSVPFMSPASAYQWFYDGYPTFGEHL..QAND
QYC65034.1   ...ELVPQLLQYMYVPPGAPKPTSRDSFAWQTATNPSVFVKMTDPPAQVSVPFMSPASAYQWFYDGYPTFGEHL..QAND
QYC65043.1   ...ELVPQLLQYMYVPPGAPKPTSRDSFAWQTATNPSVFVKMTDPPAQVSVPFMSPASAYQWFYDGYPTFGEHL..QAND
QYC65041.1   ...ELVPQLLQYMYVPPGAPKPTSRDSFAWQTATNPSVFVKMTDPPAQVSVPFMSPASAYQWFYDGYPTFGEHL..QAND
QYC65047.1   ...ELVPQLLQYMYVPPGAPKPTSRDSFAWQTATNPSVFVKMTDPPAQVSVPFMSPASAYQWFYDGYPTFGEHL..QAND
QYC65046.1   ...ELVPQLLQYMYVPPGAPKPTSRDSFAWQTATNPSVFVKMTDPPAQVSVPFMSPASAYQWFYDGYPTFGEHL..QAND
QYC65050.1   ...ELVPQLLQYMYVPPGAPKPTSRDSFAWQTATNPSVFVKMTDPPAQVSVPFMSPASAYQWFYDGYPTFGEHL..QAND
QYC65049.1   ...ELVPQLLQYMYVPPGAPKPTSRDSFAWQTATNPSVFVKMTDPPAQVSVPFMSPASAYQWFYDGYPTFGEHL..QAND
QYC65051.1   ...ELVPQLLQYMYVPPGAPKPTSRDSFAWQTATNPSVFVKMTDPPAQVSVPFMSPASAYQWFYDGYPTFGEHL..QAND
QYC65045.1   ...ELVPQLLQYMYVPPGAPKPTSRDSFAWQTATNPSVFVKMTDPPAQVSVPFMSPASAYQWFYDGYPTFGEHL..QAND
QYC65058.1   ...ELVPQLLQYMYVPPGAPKPTSRDSFAWQTATNPSVFVKMTDPPAQVSVPFMSPASAYQWFYDGYPTFGEHL..QAND
QYC65055.1   ...ELVPQLLQYMYVPPGAPKPTSRDSFAWQTATNPSVFVKMTDPPAQVSVPFMSPASAYQWFYDGYPTFGEHL..QAND
QYC65060.1   ...ELVPQLLQYMYVPPGAPKPTSRDSFAWQTATNPSVFVKMTDPPAQVSVPFMSPASAYQWFYDGYPTFGEHL..QAND
QYC65059.1   ...ELVPQLLQYMYVPPGAPKPTSRDSFAWQTATNPSVFVKMTDPPAQVSVPFMSPASAYQWFYDGYPTFGEHL..QAND
QYC65062.1   ...ELVPQLLQYMYVPPGAPKPTSRDSFAWQTATNPSVFVKMTDPPAQVSVPFMSPASAYQWFYDGYPTFGEHL..QAND
QYC65061.1   ...ELVPQLLQYMYVPPGAPKPTSRDSFAWQTATNPSVFVKMTDPPAQVSVPFMSPASAYQWFYDGYPTFGEHL..QAND
QYC65063.1   ...ELVPQLLQYMYVPPGAPKPTSRDSFAWQTATNPSVFVKMTDPPAQVSVPFMSPASAYQWFYDGYPTFGEHL..QAND
QYC65053.1   ...ELVPQLLQYMYVPPGAPKPTSRDSFAWQTATNPSVFVKMTDPPAQVSVPFMSPASAYQWFYDGYPTFGEHL..QAND
QYC65069.1   ...ELVPQLLQYMYVPPGAPKPTSRDSFAWQTATNPSVFVKMTDPPAQVSVPFMSPASAYQWFYDGYPTFGEHL..QAND
QYC65068.1   ...ELVPQLLQYMYVPPGAPKPTSRDSFAWQTATNPSVFVKMTDPPAQVSVPFMSPASAYQWFYDGYPTFGEHL..QAND
QYC65071.1   ...ELVPQLLQYMYVPPGAPKPTSRDSFAWQTATNPSVFVKMTDPPAQVSVPFMSPASAYQWFYDGYPTFGEHL..QAND
QYC65070.1   ...ELVPQLLQYMYVPPGAPKPTSRDSFAWQTATNPSVFVKMTDPPAQVSVPFMSPASAYQWFYDGYPTFGEHL..QAND
QYC65074.1   ...ELVPQLLQYMYVPPGAPKPTSRDSFAWQTATNPSVFVKMTDPPAQVSVPFMSPASAYQWFYDGYPTFGEHL..QAND
QYC65066.1   ...ELVPQLLQYMYVPPGAPKPTSRDSFAWQTATNPSVFVKMTDPPAQVSVPFMSPASAYQWFYDGYPTFGEHL..QAND
QYC65079.1   ...ELVPQLLQYMYVPPGAPKPTSRDSFAWQTATNPSVFVKMTDPPAQVSVPFMSPASAYQWFYDGYPTFGEHL..QAND
QYC65073.1   ...ELVPQLLQYMYVPPGAPKPTSRDSFAWQTATNPSVFVKMTDPPAQVSVPFMSPASAYQWFYDGYPTFGEHL..QAND
QYC65081.1   ...ELVPQLLQYMYVPPGAPKPTSRDSFAWQTATNPSVFVKMTDPPAQVSVPFMSPASAYQWFYDGYPTFGEHL..QAND
QYC65078.1   ...ELVPQLLQYMYVPPGAPKPTSRDSFAWQTATNPSVFVKMTDPPAQVSVPFMSPASAYQWFYDGYPTFGEHL..QAND
QYC65086.1   ...ELVPQLLQYMYVPPGAPKPTSRDSFAWQTATNPSVFVKMTDPPAQVSVPFMSPASAYQWFYDGYPTFGEHL..QAND
QYC65085.1   ...ELVPQLLQYMYVPPGAPKPTSRDSFAWQTATNPSVFVKMTDPPAQVSVPFMSPASAYQWFYDGYPTFGEHL..QAND
QYC65088.1   ...ELVPQLLQYMYVPPGAPKPTSRDSFAWQTATNPSVFVKMTDPPAQVSVPFMSPASAYQWFYDGYPTFGEHL..QAND
QYC65087.1   ...ELVPQLLQYMYVPPGAPKPTSRDSFAWQTATNPSVFVKMTDPPAQVSVPFMSPASAYQWFYDGYPTFGEHL..QAND
QYC65101.1   ...ELVPQLLQYMYVPPGAPKPTSRDSFAWQTATNPSVFVKMTDPPAQVSVPFMSPASAYQWFYDGYPTFGEHL..QAND
QYC65093.1   ...ELVPQLLQYMYVPPGAPKPTSRDSFAWQTATNPSVFVKMTDPPAQVSVPFMSPASAYQWFYDGYPTFGEHL..QAND
QYC65107.1   ...ELVPQLLQYMYVPPGAPKPTSRDSFAWQTATNPSVFVKMTDPPAQVSVPFMSPASAYQWFYDGYPTFGEHL..QAND
QYC65104.1   ...ELVPQLLQYMYVPPGAPKPTSRDSFAWQTATNPSVFVKMTDPPAQVSVPFMSPASAYQWFYDGYPTFGEHL..QAND
QYC65113.1   ...ELVPQLLQYMYVPPGAPKPTSRDSFAWQTATNPSVFVKMTDPPAQVSVPFMSPASAYQWFYDGYPTFGEHL..QAND
QYC65106.1   ...ELVPQLLQYMYVPPGAPKPTSRDSFAWQTATNPSVFVKMTDPPAQVSVPFMSPASAYQWFYDGYPTFGEHL..QAND
QYC65120.1   ...ELVPQLLQYMYVPPGAPKPTSRDSFAWQTATNPSVFVKMTDPPAQVSVPFMSPASAYQWFYDGYPTFGEHL..QAND
QYC65118.1   ...ELVPQLLQYMYVPPGAPKPTSRDSFAWQTATNPSVFVKMTDPPAQVSVPFMSPASAYQWFYDGYPTFGEHL..QAND
QYC65121.1   ...ELVPQLLQYMYVPPGAPKPTSRDSFAWQTATNPSVFVKMTDPPAQVSVPFMSPASAYQWFYDGYPTFGEHL..QAND
QYC65111.1   ...ELVPQLLQYMYVPPGAPKPTSRDSFAWQTATNPSVFVKMTDPPAQVSVPFMSPASAYQWFYDGYPTFGEHL..QAND
QYC65125.1   ...ELVPQLLQYMYVPPGAPKPTSRDSFAWQTATNPSVFVKMTDPPAQVSVPFMSPASAYQWFYDGYPTFGEHL..QAND
QYC65117.1   ...ELVPQLLQYMYVPPGAPKPTSRDSFAWQTATNPSVFVKMTDPPAQVSVPFMSPASAYQWFYDGYPTFGEHL..QAND
QYC65135.1   ...ELVPQLLQYMYVPPGAPKPTSRDSFAWQTATNPSVFVKMTDPPAQVSVPFMSPASAYQWFYDGYPTFGEHL..QAND
QYC65134.1   ...ELVPQLLQYMYVPPGAPKPTSRDSFAWQTATNPSVFVKMTDPPAQVSVPFMSPASAYQWFYDGYPTFGEHL..QAND
QYC65151.1   ...ELVPQLLQYMYVPPGAPKPTSRDSFAWQTATNPSVFVKMTDPPAQVSVPFMSPASAYQWFYDGYPTFGEHL..QAND
QYC65150.1   ...ELVPQLLQYMYVPPGAPKPTSRDSFAWQTATNPSVFVKMTDPPAQVSVPFMSPASAYQWFYDGYPTFGEHL..QAND
QYC65153.1   ...ELVPQLLQYMYVPPGAPKPTSRDSFAWQTATNPSVFVKMTDPPAQVSVPFMSPASAYQWFYDGYPTFGEHL..QAND
QYC65152.1   ...ELVPQLLQYMYVPPGAPKPTSRDSFAWQTATNPSVFVKMTDPPAQVSVPFMSPASAYQWFYDGYPTFGEHL..QAND
QYC65155.1   ...ELVPQLLQYMYVPPGAPKPTSRDSFAWQTATNPSVFVKMTDPPAQVSVPFMSPASAYQWFYDGYPTFGEHL..QAND
QYC65154.1   ...ELVPQLLQYMYVPPGAPKPTSRDSFAWQTATNPSVFVKMTDPPAQVSVPFMSPASAYQWFYDGYPTFGEHL..QAND
QYC65156.1   ...ELVPQLLQYMYVPPGAPKPTSRDSFAWQTATNPSVFVKMTDPPAQVSVPFMSPASAYQWFYDGYPTFGEHL..QAND
QYC65142.1   ...ELVPQLLQYMYVPPGAPKPTSRDSFAWQTATNPSVFVKMTDPPAQVSVPFMSPASAYQWFYDGYPTFGEHL..QAND
QYC65161.1   ...ELVPQLLQYMYVPPGAPKPTSRDSFAWQTATNPSVFVKMTDPPAQVSVPFMSPASAYQWFYDGYPTFGEHL..QAND
QYC65160.1   ...ELVPQLLQYMYVPPGAPKPTSRDSFAWQTATNPSVFVKMTDPPAQVSVPFMSPASAYQWFYDGYPTFGEHL..QAND
QYC65165.1   ...ELVPQLLQYMYVPPGAPKPTSRDSFAWQTATNPSVFVKMTDPPAQVSVPFMSPASAYQWFYDGYPTFGEHL..QAND
QYC65164.1   ...ELVPQLLQYMYVPPGAPKPTSRDSFAWQTATNPSVFVKMTDPPAQVSVPFMSPASAYQWFYDGYPTFGEHL..QAND
QYC65167.1   ...ELVPQLLQYMYVPPGAPKPTSRDSFAWQTATNPSVFVKMTDPPAQVSVPFMSPASAYQWFYDGYPTFGEHL..QAND
QYC65166.1   ...ELVPQLLQYMYVPPGAPKPTSRDSFAWQTATNPSVFVKMTDPPAQVSVPFMSPASAYQWFYDGYPTFGEHL..QAND
BCB92151.1   ...ELVPQLLQYMYVPPGAPKPTSRDSFAWQTATNPSVFVKMTDPPAQVSVPFMSPASAYQWFYDGYPTFGEHL..QAND
BBU37462.1   ...ELVPQLLQYMYVPPGAPKPTSRDSFAWQTATNPSVFVKMTDPPAQVSVPFMSPASAYQWFYDGYPTFGEHL..QAND
BBU37461.1   ...ELVPQLLQYMYVPPGAPKPTSRDSFAWQTATNPSVFVKMTDPPAQVSVPFMSPASAYQWFYDGYPTFGEHL..QAND
BBU37459.1   ...ELVPQLLQYMYVPPGAPKPTSRDSFAWQTATNPSVFVKMTDPPAQVSVPFMSPASAYQWFYDGYPTFGEHL..QAND
BBU37458.1   ...ELVPQLLQYMYVPPGAPKPTSRDSFAWQTATNPSVFVKMTDPPAQVSVPFMSPASAYQWFYDGYPTFGEHL..QAND
BBU37457.1   ...ELVPQLLQYMYVPPGAPKPTSRDSFAWQTATNPSVFVKMTDPPAQVSVPFMSPASAYQWFYDGYPTFGEHL..QAND
QWF08838.1   ...ELVPQLLQYMYVPPGAPKPTSRDSFAWQTATNPSVFVKMTDPPAQVSVPFMSPASAYQWFYDGYPTFGEHL..QAND
QWF08828.1   ...ELVPQLLQYMYVPPGAPKPTSRDSFAWQTATNPSVFVKMTDPPAQVSVPFMSPASAYQWFYDGYPTFGEHL..QAND
BCG66989.1   ...ELVPQLLQYMYVPPGAPKPTSRDSFAWQTATNPSVFVKMTDPPAQVSVPFMSPASAYQWFYDGYPTFGEHL..QAND
BCG66987.1   ...ELVPQLLQYMYVPPGAPKPTSRDSFAWQTATNPSVFVKMTDPPAQVSVPFMSPASAYQWFYDGYPTFGEHL..QAND
BCB92159.1   ...ELVPQLLQYMYVPPGAPKPTSRDSFAWQTATNPSVFVKMTDPPAQVSVPFMSPASAYQWFYDGYPTFGEHL..QAND
BBU37456.1   ...ELVPQLLQYMYVPPGAPKPTSRDSFAWQTATNPSVFVKMTDPPAQVSVPFMSPASAYQWFYDGYPTFGEHL..QAND
QWY15683.1   ...ELVPQLLQYMYVPPGAPKPTSRDSFAWQTATNPSVFVKMTDPPAQVSVPFMSPASAYQWFYDGYPTFGEHL..QAND
QWY15682.1   ...ELVPQLLQYMYVPPGAPKPTSRDSFAWQTATNPSVFVKMTDPPAQVSVPFMSPASAYQWFYDGYPTFGEHL..QAND
QWY15681.1   ...ELVPQLLQYMYVPPGAPKPTSRDSFAWQTATNPSVFVKMTDPPAQVSVPFMSPASAYQWFYDGYPTFGEHL..QAND
QWY15680.1   ...ELVPQLLQYMYVPPGAPKPTSRDSFAWQTATNPSVFVKMTDPPAQVSVPFMSPASAYQWFYDGYPTFGEHL..QAND
```

QWY15692.1	...ELVPQLLQYMYVPPGAPKPTSRDSFAWQTATNPSVFVKMTDPPAQVSVPFMSPASAYQWFYDGYPTFGEHL..QAND
QWY15671.1	...ELVPQLLQYMYVPPGAPKPTSRDSFAWQTATNPSVFVKMTDPPAQVSVPFMSPASAYQWFYDGYPTFGEHL..QAND
QWY15691.1	...ELVPQLLQYMYVPPGAPKPTSRDSFAWQTATNPSVFVKMTDPPAQVSVPFMSPASAYQWFYDGYPTFGEHL..QAND
QWY15690.1	...ELVPQLLQYMYVPPGAPKPTSRDSFAWQTATNPSVFVKMTDPPAQVSVPFMSPASAYQWFYDGYPTFGEHL..QAND
QWY15696.1	...ELVPQLLQYMYVPPGAPKPTSRDSFAWQTATNPSVFVKMTDPPAQVSVPFMSPASAYQWFYDGYPTFGEHL..QAND
QWY15695.1	...ELVPQLLQYMYVPPGAPKPTSRDSFAWQTATNPSVFVKMTDPPAQVSVPFMSPASAYQWFYDGYPTFGEHL..QAND
QWY15694.1	...ELVPQLLQYMYVPPGAPKPTSRDSFAWQTATNPSVFVKMTDPPAQVSVPFMSPASAYQWFYDGYPTFGEHL..QAND
QWY15678.1	...ELVPQLLQYMYVPPGAPKPTSRDSFAWQTATNPSVFVKMTDPPAQVSVPFMSPASAYQWFYDGYPTFGEHL..QAND
QWY15703.1	...ELVPQLLQYMYVPPGAPKPTSRDSFAWQTATNPSVFVKMTDPPAQVSVPFMSPASAYQWFYDGYPTFGEHL..QAND
QWY15701.1	...ELVPQLLQYMYVPPGAPKPTSRDSFAWQTATNPSVFVKMTDPPAQVSVPFMSPASAYQWFYDGYPTFGEHL..QAND
QWY15699.1	...ELVPQLLQYMYVPPGAPKPTSRDSFAWQTATNPSVFVKMTDPPAQVSVPFMSPASAYQWFYDGYPTFGEHL..QAND
QWY15673.1	...ELVPQLLQYMYVPPGAPKPTSRDSFAWQTATNPSVFVKMTDPPAQVSVPFMSPASAYQWFYDGYPTFGEHL..QAND
QWY15715.1	...ELVPQLLQYMYVPPGAPKPTSRDSFAWQTATNPSVFVKMTDPPAQVSVPFMSPASAYQWFYDGYPTFGEHL..QAND
QWY15714.1	...ELVPQLLQYMYVPPGAPKPTSRDSFAWQTATNPSVFVKMTDPPAQVSVPFMSPASAYQWFYDGYPTFGEHL..QAND
QWY15706.1	...ELVPQLLQYMYVPPGAPKPTSRDSFAWQTATNPSVFVKMTDPPAQVSVPFMSPASAYQWFYDGYPTFGEHL..QAND
QWY15705.1	...ELVPQLLQYMYVPPGAPKPTSRDSFAWQTATNPSVFVKMTDPPAQVSVPFMSPASAYQWFYDGYPTFGEHL..QAND
QTA73914.1	...ELVPQLLQYMYVPPGAPKPTSRDSFAWQTATNPSVFVKMTDPPAQVSVPFMSPASAYQWFYDGYPTFGEHL..QAND
QWY15948.1	...ELVPQLLQYMYVPPGAPKPTSRDSFAWQTATNPSVFVKMTDPPAQVSVPFMSPASAYQWFYDGYPTFGEHL..QAND
QWF08837.1	...ELVPQLLQYMYVPPGAPKPTSRDSFAWQTATNPSVFVKMTDPPAQVSVPFMSPASAYQWFYDGYPTFGEHL..QAND
QWF08804.1	...ELVPQLLQYMYVPPGAPKPTSRDSFAWQTATNPSVFVKMTDPPAQVSVPFMSPASAYQWFYDGYPTFGEHL..QAND
QWF08805.1	...ELVPQLLQYMYVPPGAPKPTSRDSFAWQTATNPSVFVKMTDPPAQVSVPFMSPASAYQWFYDGYPTFGEHL..QAND
QWF08840.1	...ELVPQLLQYMYVPPGAPKPTSRDSFAWQTATNPSVFVKMTDPPAQVSVPFMSPASAYQWFYDGYPTFGEHL..QAND
QWY15792.1	...ELVPQLLQYMYVPPGAPKPTSRDSFAWQTATNPSVFVKMTDPPAQVSVPFMSPASAYQWFYDGYPTFGEHL..QAND
QTA73903.1	...ELVPQLLQYMYVPPGAPKPTSRDSFAWQTATNPSVFVKMTDPPAQVSVPFMSPASAYQWFYDGYPTFGEHL..QAND
QTA73902.1	...ELVPQLLQYMYVPPGAPKPTSRDSFAWQTATNPSVFVKMTDPPAQVSVPFMSPASAYQWFYDGYPTFGEHL..QAND
QTA73896.1	...ELVPQLLQYMYVPPGAPKPTSRDSFAWQTATNPSVFVKMTDPPAQVSVPFMSPASAYQWFYDGYPTFGEHL..QAND
QTA73894.1	...ELVPQLLQYMYVPPGAPKPTSRDSFAWQTATNPSVFVKMTDPPAQVSVPFMSPASAYQWFYDGYPTFGEHL..QAND
QWY15940.1	...ELVPQLLQYMYVPPGAPKPTSRDSFAWQTATNPSVFVKMTDPPAQVSVPFMSPASAYQWFYDGYPTFGEHL..QAND
QWY15928.1	...ELVPQLLQYMYVPPGAPKPTSRDSFAWQTATNPSVFVKMTDPPAQVSVPFMSPASAYQWFYDGYPTFGEHL..QAND
QWY15894.1	...ELVPQLLQYMYVPPGAPKPTSRDSFAWQTATNPSVFVKMTDPPAQVSVPFMSPASAYQWFYDGYPTFGEHL..QAND
BCG67001.1	...ELVPQLLQYMYVPPGAPKPTSRDSFAWQTATNPSVFVKMTDPPAQVSVPFMSPASAYQWFYDGYPTFGEHL..QAND
QWY15872.1	...ELVPQLLQYMYVPPGAPKPTSRDSFAWQTATNPSVFVKMTDPPAQVSVPFMSPASAYQWFYDGYPTFGEHL..QAND
QWY15871.1	...ELVPQLLQYMYVPPGAPKPTSRDSFAWQTATNPSVFVKMTDPPAQVSVPFMSPASAYQWFYDGYPTFGEHL..QAND
QWY15870.1	...ELVPQLLQYMYVPPGAPKPTSRDSFAWQTATNPSVFVKMTDPPAQVSVPFMSPASAYQWFYDGYPTFGEHL..QAND
QWY15869.1	...ELVPQLLQYMYVPPGAPKPTSRDSFAWQTATNPSVFVKMTDPPAQVSVPFMSPASAYQWFYDGYPTFGEHL..QAND
QWY15866.1	...ELVPQLLQYMYVPPGAPKPTSRDSFAWQTATNPSVFVKMTDPPAQVSVPFMSPASAYQWFYDGYPTFGEHL..QAND
BCB92155.1	...ELVPQLLQYMYVPPGAPKPTSRDSFAWQTATNPSVFVKMTDPPAQVSVPFMSPASAYQWFYDGYPTFGEHL..QAND
QWY15862.1	...ELVPQLLQYMYVPPGAPKPTSRDSFAWQTATNPSVFVKMTDPPAQVSVPFMSPASAYQWFYDGYPTFGEHL..QAND
QWY15861.1	...ELVPQLLQYMYVPPGAPKPTSRDSFAWQTATNPSVFVKMTDPPAQVSVPFMSPASAYQWFYDGYPTFGEHL..QAND
QWY15852.1	...ELVPQLLQYMYVPPGAPKPTSRDSFAWQTATNPSVFVKMTDPPAQVSVPFMSPASAYQWFYDGYPTFGEHL..QAND
QWY15828.1	...ELVPQLLQYMYVPPGAPKPTSRDSFAWQTATNPSVFVKMTDPPAQVSVPFMSPASAYQWFYDGYPTFGEHL..QAND
QWY15839.1	...ELVPQLLQYMYVPPGAPKPTSRDSFAWQTATNPSVFVKMTDPPAQVSVPFMSPASAYQWFYDGYPTFGEHL..QAND
QWY15834.1	...ELVPQLLQYMYVPPGAPKPTSRDSFAWQTATNPSVFVKMTDPPAQVSVPFMSPASAYQWFYDGYPTFGEHL..QAND
QWY15810.1	...ELVPQLLQYMYVPPGAPKPTSRDSFAWQTATNPSVFVKMTDPPAQVSVPFMSPASAYQWFYDGYPTFGEHL..QAND
QWY15809.1	...ELVPQLLQYMYVPPGAPKPTSRDSFAWQTATNPSVFVKMTDPPAQVSVPFMSPASAYQWFYDGYPTFGEHL..QAND
QWY15807.1	...ELVPQLLQYMYVPPGAPKPTSRDSFAWQTATNPSVFVKMTDPPAQVSVPFMSPASAYQWFYDGYPTFGEHL..QAND
QWY15806.1	...ELVPQLLQYMYVPPGAPKPTSRDSFAWQTATNPSVFVKMTDPPAQVSVPFMSPASAYQWFYDGYPTFGEHL..QAND
QWY15805.1	...ELVPQLLQYMYVPPGAPKPTSRDSFAWQTATNPSVFVKMTDPPAQVSVPFMSPASAYQWFYDGYPTFGEHL..QAND
QWY15803.1	...ELVPQLLQYMYVPPGAPKPTSRDSFAWQTATNPSVFVKMTDPPAQVSVPFMSPASAYQWFYDGYPTFGEHL..QAND
QWY15793.1	...ELVPQLLQYMYVPPGAPKPTSRDSFAWQTATNPSVFVKMTDPPAQVSVPFMSPASAYQWFYDGYPTFGEHL..QAND
QYC65185.1	...ELVPQLLQYMYVPPGAPKPTSRDSFAWQTATNPSVFVKMTDPPAQVSVPFMSPASAYQWFYDGYPTFGEHL..QAND
QWY15754.1	...ELVPQLLQYMYVPPGAPKPTSRDSFAWQTATNPSVFVKMTDPPAQVSVPFMSPASAYQWFYDGYPTFGEHL..QAND
QWY15736.1	...ELVPQLLQYMYVPPGAPKPTSRDSFAWQTATNPSVFVKMTDPPAQVSVPFMSPASAYQWFYDGYPTFGEHL..QAND
QWY15753.1	...ELVPQLLQYMYVPPGAPKPTSRDSFAWQTATNPSVFVKMTDPPAQVSVPFMSPASAYQWFYDGYPTFGEHL..QAND
QYC65174.1	...ELVPQLLQYMYVPPGAPKPTSRDSFAWQTATNPSVFVKMTDPPAQVSVPFMSPASAYQWFYDGYPTFGEHL..QAND
QWY15730.1	...ELVPQLLQYMYVPPGAPKPTSRDSFAWQTATNPSVFVKMTDPPAQVSVPFMSPASAYQWFYDGYPTFGEHL..QAND
QWY15729.1	...ELVPQLLQYMYVPPGAPKPTSRDSFAWQTATNPSVFVKMTDPPAQVSVPFMSPASAYQWFYDGYPTFGEHL..QAND
QWY15727.1	...ELVPQLLQYMYVPPGAPKPTSRDSFAWQTATNPSVFVKMTDPPAQVSVPFMSPASAYQWFYDGYPTFGEHL..QAND
QWY15726.1	...ELVPQLLQYMYVPPGAPKPTSRDSFAWQTATNPSVFVKMTDPPAQVSVPFMSPASAYQWFYDGYPTFGEHL..QAND
QWY15725.1	...ELVPQLLQYMYVPPGAPKPTSRDSFAWQTATNPSVFVKMTDPPAQVSVPFMSPASAYQWFYDGYPTFGEHL..QAND
QWY15724.1	...ELVPQLLQYMYVPPGAPKPTSRDSFAWQTATNPSVFVKMTDPPAQVSVPFMSPASAYQWFYDGYPTFGEHL..QAND
QWY15723.1	...ELVPQLLQYMYVPPGAPKPTSRDSFAWQTATNPSVFVKMTDPPAQVSVPFMSPASAYQWFYDGYPTFGEHL..QAND
QWY15721.1	...ELVPQLLQYMYVPPGAPKPTSRDSFAWQTATNPSVFVKMTDPPAQVSVPFMSPASAYQWFYDGYPTFGEHL..QAND
QWY15746.1	...ELVPQLLQYMYVPPGAPKPTSRDSFAWQTATNPSVFVKMTDPPAQVSVPFMSPASAYQWFYDGYPTFGEHL..QAND
QWY15745.1	...ELVPQLLQYMYVPPGAPKPTSRDSFAWQTATNPSVFVKMTDPPAQVSVPFMSPASAYQWFYDGYPTFGEHL..QAND
QWY15733.1	...ELVPQLLQYMYVPPGAPKPTSRDSFAWQTATNPSVFVKMTDPPAQVSVPFMSPASAYQWFYDGYPTFGEHL..QAND
QYC65131.1	...ELVPQLLQYMYVPPGAPKPTSRDSFAWQTATNPSVFVKMTDPPAQVSVPFMSPASAYQWFYDGYPTFGEHL..QAND
QWY15813.1	...ELVPQLLQYMYVPPGAPKPTSRDSFAWQTATNPSVFVKMTDPPAQVSVPFMSPASAYQWFYDGYPTFGEHL..QAND
QWY15811.1	...ELVPQLLQYMYVPPGAPKPTSRDSFAWQTATNPSVFVKMTDPPAQVSVPFMSPASAYQWFYDGYPTFGEHL..QAND
QTA73907.1	...ELVPQLLQYMYVPPGAPKPTSRDSFAWQTATNPSVFVKMTDPPAQVSVPFMSPASAYQWFYDGYPTFGEHL..QAND
QTA73906.1	...ELVPQLLQYMYVPPGAPKPTSRDSFAWQTATNPSVFVKMTDPPAQVSVPFMSPASAYQWFYDGYPTFGEHL..QAND
QOP74940.1	...ELVPQLLQYMYVPPGAPKPTSRDSFAWQTATNPSVFVKMTDPPAQVSVPFMSPASAYQWFYDGYPTFGEHL..QAND
QOP74938.1	...ELVPQLLQYMYVPPGAPKPTSRDSFAWQTATNPSVFVKMTDPPAQVSVPFMSPASAYQWFYDGYPTFGEHL..QAND
QOP74937.1	...ELVPQLLQYMYVPPGAPKPTSRDSFAWQTATNPSVFVKMTDPPAQVSVPFMSPASAYQWFYDGYPTFGEHL..QAND
QOP74934.1	...ELVPQLLQYMYVPPGAPKPTSRDSFAWQTATNPSVFVKMTDPPAQVSVPFMSPASAYQWFYDGYPTFGEHL..QAND
QOP74929.1	...ELVPQLLQYMYVPPGAPKPTSRDSFAWQTATNPSVFVKMTDPPAQVSVPFMSPASAYQWFYDGYPTFGEHL..QAND
QOP74924.1	...ELVPQLLQYMYVPPGAPKPTSRDSFAWQTATNPSVFVKMTDPPAQVSVPFMSPASAYQWFYDGYPTFGEHL..QAND
QOP74921.1	...ELVPQLLQYMYVPPGAPKPTSRDSFAWQTATNPSVFVKMTDPPAQVSVPFMSPASAYQWFYDGYPTFGEHL..QAND
QOP74932.1	...ELVPQLLQYMYVPPGAPKPTSRDSFAWQTATNPSVFVKMTDPPAQVSVPFMSPASAYQWFYDGYPTFGEHL..QAND
QOP74931.1	...ELVPQLLQYMYVPPGAPKPTSRDSFAWQTATNPSVFVKMTDPPAQVSVPFMSPASAYQWFYDGYPTFGEHL..QAND
QIL87531.1	...ELVPQLLQYMYVPPGAPKPTSRDSFAWQTATNPSVFVKMTDPPAQVSVPFMSPASAYQWFYDGYPTFGEHL..QAND
QIL87521.1	...ELVPQLLQYMYVPPGAPKPTSRDSFAWQTATNPSVFVKMTDPPAQVSVPFMSPASAYQWFYDGYPTFGEHL..QAND
QIL87523.1	...ELVPQLLQYMYVPPGAPKPTSRDSFAWQTATNPSVFVKMTDPPAQVSVPFMSPASAYQWFYDGYPTFGEHL..QAND
QIL87517.1	...ELVPQLLQYMYVPPGAPKPTSRDSFAWQTATNPSVFVKMTDPPAQVSVPFMSPASAYQWFYDGYPTFGEHL..QAND
QIL87540.1	...ELVPQLLQYMYVPPGAPKPTSRDSFAWQTATNPSVFVKMTDPPAQVSVPFMSPASAYQWFYDGYPTFGEHL..QAND
QIL87539.1	...ELVPQLLQYMYVPPGAPKPTSRDSFAWQTATNPSVFVKMTDPPAQVSVPFMSPASAYQWFYDGYPTFGEHL..QAND
QIH53921.1	...ELVPQLLQYMYVPPGAPKPTSRDSFAWQTATNPSVFVKMTDPPAQVSVPFMSPASAYQWFYDGYPTFGEHL..QAND
QIH53916.1	...ELVPQLLQYMYVPPGAPKPTSRDSFAWQTATNPSVFVKMTDPPAQVSVPFMSPASAYQWFYDGYPTFGEHL..QAND
BBH72194.1	...ELVPQLLQYMYVPPGAPKPTSRDSFAWQTATNPSVFVKMTDPPAQVSVPFMSPASAYQWFYDGYPTFGEHL..QAND
BBH72183.1	...ELVPQLLQYMYVPPGAPKPTSRDSFAWQTATNPSVFVKMTDPPAQVSVPFMSPASAYQWFYDGYPTFGEHL..QAND
BBH72107.1	...ELVPQLLQYMYVPPGAPKPTSRDSFAWQTATNPSVFVKMTDPPAQVSVPFMSPASAYQWFYDGYPTFGEHL..QAND
QWF08825.1	...ELVPQLLQYMYVPPGAPKPTSRDSFAWQTATNPSVFVKMTDPPAQVSVPFMSPASAYQWFYDGYPTFGEHL..QAND
QWF08823.1	...ELVPQLLQYMYVPPGAPKPTSRDSFAWQTATNPSVFVKMTDPPAQVSVPFMSPASAYQWFYDGYPTFGEHL..QAND
QWF08821.1	...ELVPQLLQYMYVPPGAPKPTSRDSFAWQTATNPSVFVKMTDPPAQVSVPFMSPASAYQWFYDGYPTFGEHL..QAND
QWF08818.1	...ELVPQLLQYMYVPPGAPKPTSRDSFAWQTATNPSVFVKMTDPPAQVSVPFMSPASAYQWFYDGYPTFGEHL..QAND
QWF08815.1	...ELVPQLLQYMYVPPGAPKPTSRDSFAWQTATNPSVFVKMTDPPAQVSVPFMSPASAYQWFYDGYPTFGEHL..QAND
QWF08813.1	...ELVPQLLQYMYVPPGAPKPTSRDSFAWQTATNPSVFVKMTDPPAQVSVPFMSPASAYQWFYDGYPTFGEHL..QAND
QWF08812.1	...ELVPQLLQYMYVPPGAPKPTSRDSFAWQTATNPSVFVKMTDPPAQVSVPFMSPASAYQWFYDGYPTFGEHL..QAND
QWF08810.1	...ELVPQLLQYMYVPPGAPKPTSRDSFAWQTATNPSVFVKMTDPPAQVSVPFMSPASAYQWFYDGYPTFGEHL..QAND
QWF08809.1	...ELVPQLLQYMYVPPGAPKPTSRDSFAWQTATNPSVFVKMTDPPAQVSVPFMSPASAYQWFYDGYPTFGEHL..QAND
QWF08808.1	...ELVPQLLQYMYVPPGAPKPTSRDSFAWQTATNPSVFVKMTDPPAQVSVPFMSPASAYQWFYDGYPTFGEHL..QAND
QWF08807.1	...ELVPQLLQYMYVPPGAPKPTSRDSFAWQTATNPSVFVKMTDPPAQVSVPFMSPASAYQWFYDGYPTFGEHL..QAND
QWF08797.1	...ELVPQLLQYMYVPPGAPKPTSRDSFAWQTATNPSVFVKMTDPPAQVSVPFMSPASAYQWFYDGYPTFGEHL..QAND
QWF08841.1	...ELVPQLLQYMYVPPGAPKPTSRDSFAWQTATNPSVFVKMTDPPAQVSVPFMSPASAYQWFYDGYPTFGEHL..QAND
QWF08830.1	...ELVPQLLQYMYVPPGAPKPTSRDSFAWQTATNPSVFVKMTDPPAQVSVPFMSPASAYQWFYDGYPTFGEHL..QAND
QTA73892.1	...ELVPQLLQYMYVPPGAPKPTSRDSFAWQTATNPSVFVKMTDPPAQVSVPFMSPASAYQWFYDGYPTFGEHL..QAND
QTA73901.1	...ELVPQLLQYMYVPPGAPKPTSRDSFAWQTATNPSVFVKMTDPPAQVSVPFMSPASAYQWFYDGYPTFGEHL..QAND
QWY15957.1	...ELVPQLLQYMYVPPGAPKPTSRDSFAWQTATNPSVFVKMTDPPAQVSVPFMSPASAYQWFYDGYPTFGEHL..QAND
UEP53406.1	...ELVPQLLQYMYVPPGAPKPTSRDSFAWQTATNPSVFVKMTDPPAQVSVPFMSPASAYQWFYDGYPTFGEHL..QAND
UEC49858.1	...ELVPQLLQYMYVPPGAPKPTSRDSFAWQTATNPSVFVKMTDPPAQVSVPFMSPASAYQWFYDGYPTFGEHL..QAND
QWY15931.1	...ELVPQLLQYMYVPPGAPKPTSRDSFAWQTATNPSVFVKMTDPPAQVSVPFMSPASAYQWFYDGYPTFGEHL..QAND
QWY15929.1	...ELVPQLLQYMYVPPGAPKPTSRDSFAWQTATNPSVFVKMTDPPAQVSVPFMSPASAYQWFYDGYPTFGEHL..QAND
QWY15880.1	...ELVPQLLQYMYVPPGAPKPTSRDSFAWQTATNPSVFVKMTDPPAQVSVPFMSPASAYQWFYDGYPTFGEHL..QAND
QWY15868.1	...ELVPQLLQYMYVPPGAPKPTSRDSFAWQTATNPSVFVKMTDPPAQVSVPFMSPASAYQWFYDGYPTFGEHL..QAND
BCI65368.1	...ELVPQLLQYMYVPPGAPKPTSRDSFAWQTATNPSVFVKMTDPPAQVSVPFMSPASAYQWFYDGYPTFGEHL..QAND
BCR43097.1	...ELVPQLLQYMYVPPGAPKPTSRDSFAWQTATNPSVFVKMTDPPAQVSVPFMSPASAYQWFYDGYPTFGEHL..QAND
BCG67047.1	...ELVPQLLQYMYVPPGAPKPTSRDSFAWQTATNPSVFVKMTDPPAQVSVPFMSPASAYQWFYDGYPTFGEHL..QAND
BCG67046.1	...ELVPQLLQYMYVPPGAPKPTSRDSFAWQTATNPSVFVKMTDPPAQVSVPFMSPASAYQWFYDGYPTFGEHL..QAND
BCG67041.1	...ELVPQLLQYMYVPPGAPKPTSRDSFAWQTATNPSVFVKMTDPPAQVSVPFMSPASAYQWFYDGYPTFGEHL..QAND
BCG67040.1	...ELVPQLLQYMYVPPGAPKPTSRDSFAWQTATNPSVFVKMTDPPAQVSVPFMSPASAYQWFYDGYPTFGEHL..QAND
BCG67039.1	...ELVPQLLQYMYVPPGAPKPTSRDSFAWQTATNPSVFVKMTDPPAQVSVPFMSPASAYQWFYDGYPTFGEHL..QAND
BCG67038.1	...ELVPQLLQYMYVPPGAPKPTSRDSFAWQTATNPSVFVKMTDPPAQVSVPFMSPASAYQWFYDGYPTFGEHL..QAND
BCG67032.1	...ELVPQLLQYMYVPPGAPKPTSRDSFAWQTATNPSVFVKMTDPPAQVSVPFMSPASAYQWFYDGYPTFGEHL..QAND
BCG67030.1	...ELVPQLLQYMYVPPGAPKPTSRDSFAWQTATNPSVFVKMTDPPAQVSVPFMSPASAYQWFYDGYPTFGEHL..QAND
BCG66995.1	...ELVPQLLQYMYVPPGAPKPTSRDSFAWQTATNPSVFVKMTDPPAQVSVPFMSPASAYQWFYDGYPTFGEHL..QAND
BCG66994.1	...ELVPQLLQYMYVPPGAPKPTSRDSFAWQTATNPSVFVKMTDPPAQVSVPFMSPASAYQWFYDGYPTFGEHL..QAND
QWY15757.1	...ELVPQLLQYMYVPPGAPKPTSRDSFAWQTATNPSVFVKMTDPPAQVSVPFMSPASAYQWFYDGYPTFGEHL..QAND
QWY15756.1	...ELVPQLLQYMYVPPGAPKPTSRDSFAWQTATNPSVFVKMTDPPAQVSVPFMSPASAYQWFYDGYPTFGEHL..QAND
QWY15748.1	...ELVPQLLQYMYVPPGAPKPTSRDSFAWQTATNPSVFVKMTDPPAQVSVPFMSPASAYQWFYDGYPTFGEHL..QAND
QWY15747.1	...ELVPQLLQYMYVPPGAPKPTSRDSFAWQTATNPSVFVKMTDPPAQVSVPFMSPASAYQWFYDGYPTFGEHL..QAND
QWY15720.1	...ELVPQLLQYMYVPPGAPKPTSRDSFAWQTATNPSVFVKMTDPPAQVSVPFMSPASAYQWFYDGYPTFGEHL..QAND
QWY15717.1	...ELVPQLLQYMYVPPGAPKPTSRDSFAWQTATNPSVFVKMTDPPAQVSVPFMSPASAYQWFYDGYPTFGEHL..QAND
QWY15697.1	...ELVPQLLQYMYVPPGAPKPTSRDSFAWQTATNPSVFVKMTDPPAQVSVPFMSPASAYQWFYDGYPTFGEHL..QAND
QWY15689.1	...ELVPQLLQYMYVPPGAPKPTSRDSFAWQTATNPSVFVKMTDPPAQVSVPFMSPASAYQWFYDGYPTFGEHL..QAND
QWY15761.1	...ELVPQLLQYMYVPPGAPKPTSRDSFAWQTATNPSVFVKMTDPPAQVSVPFMSPASAYQWFYDGYPTFGEHL..QAND
QWY15743.1	...ELVPQLLQYMYVPPGAPKPTSRDSFAWQTATNPSVFVKMTDPPAQVSVPFMSPASAYQWFYDGYPTFGEHL..QAND
QWY15759.1	...ELVPQLLQYMYVPPGAPKPTSRDSFAWQTATNPSVFVKMTDPPAQVSVPFMSPASAYQWFYDGYPTFGEHL..QAND
QWY15758.1	...ELVPQLLQYMYVPPGAPKPTSRDSFAWQTATNPSVFVKMTDPPAQVSVPFMSPASAYQWFYDGYPTFGEHL..QAND
QWY15677.1	...ELVPQLLQYMYVPPGAPKPTSRDSFAWQTATNPSVFVKMTDPPAQVSVPFMSPASAYQWFYDGYPTFGEHL..QAND
QWY15675.1	...ELVPQLLQYMYVPPGAPKPTSRDSFAWQTATNPSVFVKMTDPPAQVSVPFMSPASAYQWFYDGYPTFGEHL..QAND
QWY15776.1	...ELVPQLLQYMYVPPGAPKPTSRDSFAWQTATNPSVFVKMTDPPAQVSVPFMSPASAYQWFYDGYPTFGEHL..QAND
QWY15772.1	...ELVPQLLQYMYVPPGAPKPTSRDSFAWQTATNPSVFVKMTDPPAQVSVPFMSPASAYQWFYDGYPTFGEHL..QAND
QWY15770.1	...ELVPQLLQYMYVPPGAPKPTSRDSFAWQTATNPSVFVKMTDPPAQVSVPFMSPASAYQWFYDGYPTFGEHL..QAND
QWY15762.1	...ELVPQLLQYMYVPPGAPKPTSRDSFAWQTATNPSVFVKMTDPPAQVSVPFMSPASAYQWFYDGYPTFGEHL..QAND

```
QWY15732.1    ...ELVPQLLQYMYVPPGAPKPTSRDSFAWQTATNPSVFVKMTDPPAQVSVPFMSPASAYQWFYDGYPTFGEHL..QAND
QWY15731.1    ...ELVPQLLQYMYVPPGAPKPTSRDSFAWQTATNPSVFVKMTDPPAQVSVPFMSPASAYQWFYDGYPTFGEHL..QAND
QWY15780.1    ...ELVPQLLQYMYVPPGAPKPTSRDSFAWQTATNPSVFVKMTDPPAQVSVPFMSPASAYQWFYDGYPTFGEHL..QAND
QWY15779.1    ...ELVPQLLQYMYVPPGAPKPTSRDSFAWQTATNPSVFVKMTDPPAQVSVPFMSPASAYQWFYDGYPTFGEHL..QAND
BCG67006.1    ...ELVPQLLQYMYVPPGAPKPTSRDSFAWQTATNPSVFVKMTDPPAQVSVPFMSPASAYQWFYDGYPTFGEHL..QAND
BCG67004.1    ...ELVPQLLQYMYVPPGAPKPTSRDSFAWQTATNPSVFVKMTDPPAQVSVPFMSPASAYQWFYDGYPTFGEHL..QAND
BCG67019.1    ...ELVPQLLQYMYVPPGAPKPTSRDSFAWQTATNPSVFVKMTDPPAQVSVPFMSPASAYQWFYDGYPTFGEHL..QAND
BCG67002.1    ...ELVPQLLQYMYVPPGAPKPTSRDSFAWQTATNPSVFVKMTDPPAQVSVPFMSPASAYQWFYDGYPTFGEHL..QAND
BCG67036.1    ...ELVPQLLQYMYVPPGAPKPTSRDSFAWQTATNPSVFVKMTDPPAQVSVPFMSPASAYQWFYDGYPTFGEHL..QAND
BCG67035.1    ...ELVPQLLQYMYVPPGAPKPTSRDSFAWQTATNPSVFVKMTDPPAQVSVPFMSPASAYQWFYDGYPTFGEHL..QAND
QWY15785.1    ...ELVPQLLQYMYVPPGAPKPTSRDSFAWQTATNPSVFVKMTDPPAQVSVPFMSPASAYQWFYDGYPTFGEHL..QAND
QWY15784.1    ...ELVPQLLQYMYVPPGAPKPTSRDSFAWQTATNPSVFVKMTDPPAQVSVPFMSPASAYQWFYDGYPTFGEHL..QAND
QWY15789.1    ...ELVPQLLQYMYVPPGAPKPTSRDSFAWQTATNPSVFVKMTDPPAQVSVPFMSPASAYQWFYDGYPTFGEHL..QAND
QYC65159.1    ...ELVPQLLQYMYVPPGAPKPTSRDSFAWQTATNPSVFVKMTDPPAQVSVPFMSPASAYQWFYDGYPTFGEHL..QAND
BCR43101.1    ...ELVPQLLQYMYVPPGAPKPTSRDSFAWQTATNPSVFVKMTDPPAQVSVPFMSPASAYQWFYDGYPTFGEHL..QAND
BCR43098.1    ...ELVPQLLQYMYVPPGAPKPTSRDSFAWQTATNPSVFVKMTDPPAQVSVPFMSPASAYQWFYDGYPTFGEHL..QAND
QWY15873.1    ...ELVPQLLQYMYVPPGAPKPTSRDSFAWQTATNPSVFVKMTDPPAQVSVPFMSPASAYQWFYDGYPTFGEHL..QAND
QWY15865.1    ...ELVPQLLQYMYVPPGAPKPTSRDSFAWQTATNPSVFVKMTDPPAQVSVPFMSPASAYQWFYDGYPTFGEHL..QAND
QWY15900.1    ...ELVPQLLQYMYVPPGAPKPTSRDSFAWQTATNPSVFVKMTDPPAQVSVPFMSPASAYQWFYDGYPTFGEHL..QAND
BCG67049.1    ...ELVPQLLQYMYVPPGAPKPTSRDSFAWQTATNPSVFVKMTDPPAQVSVPFMSPASAYQWFYDGYPTFGEHL..QAND
UEC49853.1    ...ELVPQLLQYMYVPPGAPKPTSRDSFAWQTATNPSVFVKMTDPPAQVSVPFMSPASAYQWFYDGYPTFGEHL..QAND
UEC49848.1    ...ELVPQLLQYMYVPPGAPKPTSRDSFAWQTATNPSVFVKMTDPPAQVSVPFMSPASAYQWFYDGYPTFGEHL..QAND
QTA73899.1    ...ELVPQLLQYMYVPPGAPKPTSRDSFAWQTATNPSVFVKMTDPPAQVSVPFMSPASAYQWFYDGYPTFGEHL..QAND
QTA73898.1    ...ELVPQLLQYMYVPPGAPKPTSRDSFAWQTATNPSVFVKMTDPPAQVSVPFMSPASAYQWFYDGYPTFGEHL..QAND
QTA73905.1    ...ELVPQLLQYMYVPPGAPKPTSRDSFAWQTATNPSVFVKMTDPPAQVSVPFMSPASAYQWFYDGYPTFGEHL..QAND
QTA73904.1    ...ELVPQLLQYMYVPPGAPKPTSRDSFAWQTATNPSVFVKMTDPPAQVSVPFMSPASAYQWFYDGYPTFGEHL..QAND
QTA73909.1    ...ELVPQLLQYMYVPPGAPKPTSRDSFAWQTATNPSVFVKMTDPPAQVSVPFMSPASAYQWFYDGYPTFGEHL..QAND
QTA73908.1    ...ELVPQLLQYMYVPPGAPKPTSRDSFAWQTATNPSVFVKMTDPPAQVSVPFMSPASAYQWFYDGYPTFGEHL..QAND
UEP53408.1    ...ELVPQLLQYMYVPPGAPKPTSRDSFAWQTATNPSVFVKMTDPPAQVSVPFMSPASAYQWFYDGYPTFGEHL..QAND
QWF08839.1    ...ELVPQLLQYMYVPPGAPKPTSRDSFAWQTATNPSVFVKMTDPPAQVSVPFMSPASAYQWFYDGYPTFGEHL..QAND
QWF08799.1    ...ELVPQLLQYMYVPPGAPKPTSRDSFAWQTATNPSVFVKMTDPPAQVSVPFMSPASAYQWFYDGYPTFGEHL..QAND
QWY15771.1    ...ELVPQLLQYMYVPPGAPKPTSRDSFAWQTATNPSVFVKMTDPPAQVSVPFMSPASAYQWFYDGYPTFGEHL..QAND
QTA73891.1    ...ELVPQLLQYMYVPPGAPKPTSRDSFAWQTATNPSVFVKMTDPPAQVSVPFMSPASAYQWFYDGYPTFGEHL..QAND
QWY15670.1    ...ELVPQLLQYMYVPPGAPKPTSRDSFAWQTATNPSVFVKMTDPPAQVSVPFMSPASAYQWFYDGYPTFGEHL..QAND
BBH72149.1    ...ELVPQLLQYMYVPPGAPKPTSRDSFAWQTATNPSVFVKMTDPPAQVSVPFMSPASAYQWFYDGYPTFGEHL..QAND
BBH72143.1    ...ELVPQLLQYMYVPPGAPKPTSRDSFAWQTATNPSVFVKMTDPPAQVSVPFMSPASAYQWFYDGYPTFGEHL..QAND
BBH72163.1    ...ELVPQLLQYMYVPPGAPKPTSRDSFAWQTATNPSVFVKMTDPPAQVSVPFMSPASAYQWFYDGYPTFGEHL..QAND
QTA73895.1    ...ELVPQLLQYMYVPPGAPKPTSRDSFAWQTATNPSVFVKMTDPPAQVSVPFMSPASAYQWFYDGYPTFGEHL..QAND
QWY15674.1    ...ELVPQLLQYMYVPPGAPKPTSRDSFAWQTATNPSVFVKMTDPPAQVSVPFMSPASAYQWFYDGYPTFGEHL..QAND
QWY15710.1    ...ELVPQLLQYMYVPPGAPKPTSRDSFAWQTATNPSVFVKMTDPPAQVSVPFMSPASAYQWFYDGYPTFGEHL..QAND
QWY15709.1    ...ELVPQLLQYMYVPPGAPKPTSRDSFAWQTATNPSVFVKMTDPPAQVSVPFMSPASAYQWFYDGYPTFGEHL..QAND
QWF08934.1    ...ELVPQLLQYMYVPPGAPKPTSRDSFAWQTATNPSVFVKMTDPPAQVSVPFMSPASAYQWFYDGYPTFGEHL..QAND
QWF08836.1    ...ELVPQLLQYMYVPPGAPKPTSRDSFAWQTATNPSVFVKMTDPPAQVSVPFMSPASAYQWFYDGYPTFGEHL..QAND
QLH02014.1    ...ELAPQLLQYMYVPPGAPKPTSRDSFAWQTATNPSVFVKMTDPPAQVSVPFMSPASAYQWFYDGYPTFGEHL..QAND
QWF08796.1    ...ELVPQLLQYMYVPPGAPKPTSRDSFAWQTATNPSVFVKMTDPPAQVSVPFMSPASAYQWFYDGYPTFGEHL..QAND
QYC65090.1    ...ELVPQLLQYMYVPPGAPKPTSRDSFAWQTATNPSVFVKMTDPPAQVSVPFMSPASAYQWFYDGYPTFGEHL..QAND
QTA73900.1    ...ELVPQLLQYMYVPPGAPKPTSRDSFAWQTATNPSVFVKMTDPPAQVSVPFMSPASAYQWFYDGYPTFGEHL..QASD
QWY15679.1    ...ELVPQLLQYMYVPPGAPKPTSRDSFAWQTATNPSVFVKMTDPPAQVSVPFMSPASAYQWFYDGYPTFGEHL..QASD
QWY15751.1    ...ELVPQLLQYMYVPPGAPKPTSRDSFAWQTATNPSVFVKMTDPPAQVSVPFMSPASAYQWFYDGYPTFGEHL..QASD
QWF08795.1    ...ELVPQLLQYMYVPPGAPKPTSRDSFAWQTATNPSVFVKMTDPPAQVSVPFMSPASAYQWFYDGYPTFGEHL..QAND
QWF08789.1    ...ELVPQLLQYMYVPPGAPKPTSRDSFAWQTATNPSVFVKMTDPPAQVSVPFMSPASAYQWFYDGYPTFGEHL..QAND
QYC65158.1    ...ELVPQLLQYMYVPPGAPKPTSRDSFAWQTATNPSVFVKMTDPPAQVSVPFMSPASAYQWFYDGYPTFGEHL..QAND
QYC65109.1    ...ELVPQLLQYMYVPPGAPKPTSRDSFAWQTATNPSVFVKMTDPPAQVSVPFMSPASAYQWFYDGYPTFGEHL..QAND
QWF08883.1    ...ELVPQLLQYMYVPPGAPKPTSRDSFAWQTATNPSVFVKMTDPPAQVSVPFMSPASAYQWFYDGYPTFGEHL..QAND
QWF08866.1    ...ELVPQLLQYMYVPPGAPKPTSRDSFAWQTATNPSVFVKMTDPPAQVSVPFMSPASAYQWFYDGYPTFGEHL..QAND
QWF08833.1    ...ELVPQLLQYMYVPPGAPKPTSRDSFAWQTATNPSVFVKMTDPPAQVSVPFMSPASAYQWFYDGYPTFGEHL..QAND
QWF08801.1    ...ELVPQLLQYMYVPPGAPKPTSRDSFAWQTATNPSVFVKMTDPPAQVSVPFMSPASAYQWFYDGYPTFGEHL..QAND
QWF08842.1    ...ELVPPLLPYMYVPPGAPKPTSRDSFAWQTATNPSVFVKMTDPPAQVSVPFMSPASAYQWFYDGYPTFGEHL..QAND
QWF08806.1    ...ELVPPLLPYMYVPPGAPKPTSRDSFAWQTATNPSVFVKMTDPPAQVSVPFMSPASAYQWFYDGYPTFGEHL..QAND
QYC65065.1    ...ELVPQLLQYMYVPPGAPKPTSRDSFAWQTATNPSVFVKMTDPPAQVSVPFMSPASAYQWFYDGYPTFGEHL..QAND
QYC65044.1    ...ELVPQLLQYMYVPPGAPKPTSRDSFAWQTATNPSVFVKMTDPPAQVSVPFMSPASAYQWFYDGYPTFGEHL..QAND
QWY15863.1    ...ELVPQLLQYMYVPPGAPKPTSRDSFAWQTATNPSVFVKMTDPPAQVSVPFMSPASAYQWFYDGYPTFGEHL..QAND
QTA73916.1    ...ELVPQLLQYMYVPPGAPKPTSRDSFAWQTATNPSVFVKMTDPPAQVSVPFMSPASAYQWFYDGYPTFGEHL..QAND
BCG66990.1    ...ELVPQLLQYMYVPPGAPKPTSRDSFAWQTATNPSVFVKMTDPPAQVSVPFMSPASAYQWFYDGYPTFGEHL..QAND
QTA73915.1    ...ELVPQLLQYMYVPPGAPKPTSRDSFAWQTATNPSVFVKMTDPPAQVSVPFMSPASAYQWFYDGYPTFGEHL..QAND
QWY15932.1    ...ELVPQLLQYMYVPPGAPKPTSRDSFAWQTATNPSVFVKMTDPPAQVSVPFMSPASAYQWFYDGYPTFGEHL..QAND
QWY15867.1    ...ELVPQLLQYMYVPPGAPKPTSRDSFAWQTATNPSVFVKMTDPPAQVSVPFMSPASAYQWFYDGYPTFGEHL..QAND
QWY15859.1    ...ELVPQLLQYMYVPPGAPKPTSRDSFAWQTATNPSVFVKMTDPPAQVSVPFMSPASAYQWFYDGYPTFGEHL..QAND
QWY15858.1    ...ELVPQLLQYMYVPPGAPKPTSRDSFAWQTATNPSVFVKMTDPPAQVSVPFMSPASAYQWFYDGYPTFGEHL..QAND
QWY15782.1    ...ELVPQLLQYMYVPPGAPKPTSRDSFAWQTATNPSVFVKMTDPPAQVSVPFMSPASAYQWFYDGYPTFGEHL..QAND
QWY15781.1    ...ELVPQLLQYMYVPPGAPKPTSRDSFAWQTATNPSVFVKMTDPPAQVSVPFMSPASAYQWFYDGYPTFGEHL..QAND
BCI65355.1    ...ELVPQLLQYMYVPPGAPKPTSRDSFAWQTATNPSVFVKMTDPPAQVSVPFMSPASAYQWFYDGYPTFGEHL..QAND
BCI65374.1    ...ELVPQLLQYMYVPPGAPKPTSRDSFAWQTATNPSVFVKMTDPPAQVSVPFMSPASAYQWFYDGYPTFGEHL..QAND
BCI65373.1    ...ELVPQLLQYMYVPPGAPKPTSRDSFAWQTATNPSVFVKMTDPPAQVSVPFMSPASAYQWFYDGYPTFGEHL..QAND
BCI65372.1    ...ELVPQLLQYMYVPPGAPKPTSRDSFAWQTATNPSVFVKMTDPPAQVSVPFMSPASAYQWFYDGYPTFGEHL..QAND
BCI65371.1    ...ELVPQLLQYMYVPPGAPKPTSRDSFAWQTATNPSVFVKMTDPPAQVSVPFMSPASAYQWFYDGYPTFGEHL..QAND
BCI65369.1    ...ELVPQLLQYMYVPPGAPKPTSRDSFAWQTATNPSVFVKMTDPPAQVSVPFMSPASAYQWFYDGYPTFGEHL..QAND
BCI65367.1    ...ELVPQLLQYMYVPPGAPKPTSRDSFAWQTATNPSVFVKMTDPPAQVSVPFMSPASAYQWFYDGYPTFGEHL..QAND
BCI65365.1    ...ELVPQLLQYMYVPPGAPKPTSRDSFAWQTATNPSVFVKMTDPPAQVSVPFMSPASAYQWFYDGYPTFGEHL..QAND
BCI65364.1    ...ELVPQLLQYMYVPPGAPKPTSRDSFAWQTATNPSVFVKMTDPPAQVSVPFMSPASAYQWFYDGYPTFGEHL..QAND
BCI65363.1    ...ELVPQLLQYMYVPPGAPKPTSRDSFAWQTATNPSVFVKMTDPPAQVSVPFMSPASAYQWFYDGYPTFGEHL..QAND
BCI65362.1    ...ELVPQLLQYMYVPPGAPKPTSRDSFAWQTATNPSVFVKMTDPPAQVSVPFMSPASAYQWFYDGYPTFGEHL..QAND
BCI65361.1    ...ELVPQLLPYMYVPPGAPKPTSRDSFAWQTATNPSVFVKMTDPPAQVSVPFMSPASAYQWFYDGYPTFGEHL..QAND
BCI65360.1    ...ELVPQLLQYMYVPPGAPKPTSRDSFAWQTATNPSVFVKMTDPPAQVSVPFMSPASAYQWFYDGYPTFGEHL..QAND
BCI65359.1    ...ELVPQLLQYMYVPPGAPKPTSRDSFAWQTATNPSVFVKMTDPPAQVSVPFMSPASAYQWFYDGYPTFGEHL..QAND
BCI65358.1    ...ELVPQLLQYMYVPPGAPKPTSRDSFAWQTATNPSVFVKMTDPPAQVSVPFMSPASAYQWFYDGYPTFGEHL..QAND
BCI65356.1    ...ELVPQLLQYMYVPPGAPKPTSRDSFAWQTATNPSVFVKMTDPPAQVSVPFMSPASAYQWFYDGYPTFGEHL..QAND
BCI65354.1    ...ELVPQLLQYMYVPPGAPKPTSRDSFAWQTATNPSVFVKMTDPPAQVSVPFMSPASAYQWFYDGYPTFGEHL..QAND
BCI65353.1    ...ELVPQLLQYMYVPPGAPKPTSRDSFAWQTATNPSVFVKMTDPPAQVSVPFMSPASAYQWFYDGYPTFGEHL..QAND
BCI65352.1    ...ELVPQLLQYMYVPPGAPKPTSRDSFAWQTATNPSVFVKMTDPPAQVSVPFMSPASAYQWFYDGYPTFGEHL..QAND
BCI65351.1    ...ELVPQLLQYMYVPPGAPKPTSRDSFAWQTATNPSVFVKMTDPPAQVSVPFMSPASAYQWFYDGYPTFGEHL..QAND
BCI65350.1    ...ELVPQLLQYMYVPPGAPKPTSRDSFAWQTATNPSVFVKMTDPPAQVSVPFMSPASAYQWFYDGYPTFGEHL..QAND
BCI65349.1    ...ELVPQLLQYMYVPPGAPKPTSRDSFAWQTATNPSVFVKMTDPPAQVSVPFMSPASAYQWFYDGYPTFGEHL..QAND
BCI65348.1    ...ELVPQLLQYMYVPPGAPKPTSRDSFAWQTATNPSVFVKMTDPPAQVSVPFMSPASAYQWFYDGYPTFGEHL..QAND
BCI65347.1    ...ELVPQLLQYMYVPPGAPKPTSRDSFAWQTATNPSVFVKMTDPPAQVSVPFMSPASAYQWFYDGYPTFGEHL..QAND
BCI65346.1    ...ELVPQLLQYMYVPPGAPKPTSRDSFAWQTATNPSVFVKMTDPPAQVSVPFMSPASAYQWFYDGYPTFGEHL..QAND
BCI65345.1    ...ELVPQLLQYMYVPPGAPKPTSRDSFAWQTATNPSVFVKMTDPPAQVSVPFMSPASAYQWFYDGYPTFGEHL..QAND
BCI65344.1    ...ELVPQLLQYMYVPPGAPKPTSRDSFAWQTATNPSVFVKMTDPPAQVSVPFMSPASAYQWFYDGYPTFGEHL..QAND
BCI65343.1    ...ELVPQLLQYMYVPPGAPKPTSRDSFAWQTATNPSVFVKMTDPPAQVSVPFMSPASAYQWFYDGYPTFGEHL..QAND
BCI65342.1    ...ELVPQLLQYMYVPPGAPKPTSRDSFAWQTATNPSVFVKMTDPPAQVSVPFMSPASAYQWFYDGYPTFGEHL..QAND
BCI65341.1    ...ELVPQLLQYMYVPPGAPKPTSRDSFAWQTATNPSVFVKMTDPPAQVSVPFMSPASAYQWFYDGYPTFGEHL..QAND
BCI65340.1    ...ELVPQLLQYMYVPPGAPKPTSRDSFAWQTATNPSVFVKMTDPPAQVSVPFMSPASAYQWFYDGYPTFGEHL..QAND
BCI65339.1    ...ELVPQLLQYMYVPPGAPKPTSRDSFAWQTATNPSVFVKMTDPPAQVSVPFMSPASAYQWFYDGYPTFGEHL..QAND
BCI65338.1    ...ELVPQLLQYMYVPPGAPKPTSRDSFAWQTATNPSVFVKMTDPPAQVSVPFMSPASAYQWFYDGYPTFGEHL..QAND
BCI65337.1    ...ELVPQLLQYMYVPPGAPKPTSRDSFAWQTATNPSVFVKMTDPPAQVSVPFMSPASAYQWFYDGYPTFGEHL..QAND
BCI65336.1    ...ELVPQLLQYMYVPPGAPKPTSRDSFAWQTATNPSVFVKMTDPPAQVSVPFMSPASAYQWFYDGYPTFGEHL..QAND
BCI65335.1    ...ELVPQLLQYMYVPPGAPKPTSRDSFAWQTATNPSVFVKMTDPPAQVSVPFMSPASAYQWFYDGYPTFGEHL..QAND
BCI65334.1    ...ELVPQLLQYMYVPPGAPKPTSRDSFAWQTATNPSVFVKMTDPPAQVSVPFMSPASAYQWFYDGYPTFGEHL..QAND
BCI65333.1    ...ELVPQLLQYMYVPPGAPKPTSRDSFAWQTATNPSVFVKMTDPPAQVSVPFMSPASAYQWFYDGYPTFGEHL..QAND
BCI65332.1    ...ELVPQLLQYMYVPPGAPKPTSRDSFAWQTATNPSVFVKMTDPPAQVSVPFMSPASAYQWFYDGYPTFGEHL..QAND
BCI65331.1    ...ELVPQLLQYMYVPPGAPKPTSRDSFAWQTATNPSVFVKMTDPPAQVSVPFMSPASAYQWFYDGYPTFGEHL..QAND
BCI65330.1    ...ELVPQLLQYMYVPPGAPKPTSRDSFAWQTATNPSVFVKMTDPPAQVSVPFMSPASAYQWFYDGYPTFGEHL..QAND
BCI65328.1    ...ELVPQLLQYMYVPPGAPKPTSRDSFAWQTATNPSVFVKMTDPPAQVSVPFMSPASAYQWFYDGYPTFGEHL..QAND
BCI65327.1    ...ELVPQLLQYMYVPPGAPKPTSRDSFAWQTATNPSVFVKMTDPPAQVSVPFMSPASAYQWFYDGYPTFGEHL..QAND
BCI65326.1    ...ELVPQLLQYMYVPPGAPKPTSRDSFAWQTATNPSVFVKMTDPPAQVSVPFMSPASAYQWFYDGYPTFGEHL..QAND
BCI65325.1    ...ELVPQLLQYMYVPPGAPKPTSRDSFAWQTATNPSVFVKMTDPPAQVSVPFMSPASAYQWFYDGYPTFGEHL..QAND
BCI65324.1    ...ELVPQLLQYMYVPPGAPKPTSRDSFAWQTATNPSVFVKMTDPPAQVSVPFMSPASAYQWFYDGYPTFGEHL..QAND
BCI65323.1    ...ELVPQLLQYMYVPPGAPKPTSRDSFAWQTATNPSVFVKMTDPPAQVSVPFMSPASAYQWFYDGYPTFGEHL..QAND
BCI65322.1    ...ELVPQLLQYMYVPPGAPKPTSRDSFAWQTATNPSVFVKMTDPPAQVSVPFMSPASAYQWFYDGYPTFGEHL..QAND
BCI65321.1    ...ELVPQLLQYMYVPPGAPKPTSRDSFAWQTATNPSVFVKMTDPPAQVSVPFMSPASAYQWFYDGYPTFGEHL..QAND
BCI65320.1    ...ELVPQLLQYMYVPPGAPKPTSRDSFAWQTATNPSVFVKMTDPPAQVSVPFMSPASAYQWFYDGYPTFGEHL..QAND
BCI65319.1    ...ELVPQLLQYMYVPPGAPKPTSRDSFAWQTATNPSVFVKMTDPPAQVSVPFMSPASAYQWFYDGYPTFGEHL..QAND
BCI65318.1    ...ELVPQLLQYMYVPPGAPKPTSRDSFAWQTATNPSVFVKMTDPPAQVSVPFMSPASAYQWFYDGYPTFGEHL..QAND
BCI65317.1    ...ELVPQLLQYMYVPPGAPKPTSRDSFAWQTATNPSVFVKMTDPPAQVSVPFMSPASAYQWFYDGYPTFGEHL..QAND
QYC65119.1    ...ELVPQLLQYMYVPPGAPKPTSRDSFAWQTATNPSVFVKMTDPPAQVSVPFMSPASAYQWFYDGYPTFGEHL..QAND
QWF08905.1    ...ELVPQLLQYMYVPPGAPKPTSRDSFAWQTATNPSVFVKMTDPPAQVSVPFMSPASAYQWFYDGYPTFGEHL..QAND
QWF08814.1    ...ELVPQLLQYMYVPPGAPKPTSRDSFAWQTATNPSVFVKMTDPPAQVSVPFMSPASAYQWFYDGYPTFGEHL..QAND
QWF08776.1    ...ELVPQLLQYMYVPPGAPKPTSRDSFAWQTATNPSVFVKMTDPPAQVSVPFMSPASAYQWFYDGYPTFGEHL..QAND
BCI65370.1    ...ELVPQLLQYMYVPPGAPKPTSRDSFAWQTATNPSVFVKMTDPPAQVSVPFMSPASAYQWFYDGYPTFGEHL..QAND
BCG67048.1    ...ELVPQLLQYMYVPPGAPKPTSRDSFAWQTATNPSVFVKMTDPPAQVSVPFMSPASAYQWFYDGYPTFGEHL..QAND
QWY15933.1    ...ELVPQLLQYMYVPPGAPKPTSRDSFAWQTATNPSVFVKMTDPPAQVSVPFMSPASAYQWFYDGYPTFGEHL..QAND
UEP53404.1    ...ELVPQLLQYMYVPPGAPKPTSRDSFAWQTATNPSVFVKMTDPPAQVSVPFMSPASAYQWFYDGYPTFGEHL..QAND
UEC49857.1    ...ELVPQLLQYMYVPPGAPKPTSRDSFAWQTATNPSVFVKMTDPPAQVSVPFMSPASAYQWFYDGYPTFGEHL..QAND
QYC65076.1    ...ELVPQLLQYMYVPPGAPKPTSRDSFAWQTATNPSVFVKMTDPPAQVSVPFMSPASAYQWFYDGYPTFGEHL..QAND
QWF08819.1    ...ELVPQLLQYMYVPPGAPKPTSRDSFAWQTATNPSVFVKMTDPPAQVSVPFMSPASAYQWFYDGYPTFGEHL..QAND
QYC65082.1    ...ELVPQLLQYMYVPPGAPKPTSRDSFAWQTATNPSVFVKMTDPPAQVSVPFMSPASAYQWFYDGYPTFGEHL..QAND
QWY15927.1    ...ELVPQLLQYMYVPPGAPKPTSRDSFAWQTATNPSVFVKMTDPPAQVSVPFMSPASAYQWFYDGYPTFGEHL..QAND
QOP74944.1    ...ELVPQLLQYMYVPPGAPKPTSRDSFAWQTATNPSVFVKMTDPPAQVSVPFMSPASAYQWFYDGYPTFGEHL..QAND
QYC65139.1    ...ELVPQLLQYMYVPPGAPKPTSRDSFAWQTATNPSVFVKMTDPPAQVSVPFMSPASAYQWFYDGYPTFGEHL..QAND
QYC65136.1    ...ELVPQLLQYMYVPPGAPKPTSRDSFAWQTATNPSVFVKMTDPPAQVSVPFMSPASAYQWFYDGYPTFGEHL..QAND
QYC65130.1    ...ELVPQLLQYMYVPPGAPKPTSRDSFAWQTATNPSVFVKMTDPPAQVSVPFMSPASAYQWFYDGYPTFGEHL..QAND
QYC65115.1    ...ELVPQLLQYMYVPPGAPKPTSRDSFAWQTATNPSVFVKMTDPPAQVSVPFMSPASAYQWFYDGYPTFGEHL..QAND
QYC65114.1    ...ELVPQLLQYMYVPPGAPKPTSRDSFAWQTATNPSVFVKMTDPPAQVSVPFMSPASAYQWFYDGYPTFGEHL..QAND
QYC65105.1    ...ELVPQLLQYMYVPPGAPKPTSRDSFAWQTATNPSVFVKMTDPPAQVSVPFMSPASAYQWFYDGYPTFGEHL..QAND
```

```
QWF08949.1     ...ELVPQLLQYMYVPPGAPKPTSRDSFAWQTATNPSVFVKMTDPPAQVSVPFMSPASAYQWFYDGYPTFGEHL..QAND
QWF08928.1     ...ELVPQLLQYMYVPPGAPKPTSRDSFAWQTATNPSVFVKMTDPPAQVSVPFMSPASAYQWFYDGYPTFGEHL..QAND
QWY15921.1     ...ELVPQLLQYMYVPPGAPKPTSRDSFAWQTATNPSVFVKMTDPPAQVSVPFMSPASAYQWFYDGYPTFGEHL..QADD
QWY15919.1     ...ELVPQLLQYMYVPPGAPKPTSRDSFAWQTATNPSVFVKMTDPPAQVSVPFMSPASAYQWFYDGYPTFGEHL..QADD
QYC65102.1     ...ELVPQLLQYMYVPPGAPKPTSRDSFAWQTATNPSVFVKMTDPPAQVSVPFMSPASAYQWFYDGYPTFGEHL..QAND
BCR43082.1     ...ELVPQLLQYMYVPPGAPKPTSRDSFAWQTATNPSVFVKMTDPPAQVSVPFMSPASAYQWFYDGYPTFGEHL..QAND
QWY15713.1     ...ELVPQLLQYMYVPPGAPKPTSRDSFAWQTATNPSVFVKMTDPPAQVSVPFMSPASAYQWFYDGYPTFGEHL..QAND
BCI65329.1     ...ELVPQLLQYMYVPPGAPKPTSRDSFAWQTATNPSVFVKMTDPPAQVSVPFMSPASAYQWFYDGYPTFGEHL..QAND
QWF08887.1     ...ELVPQLLQYMYVPPGAPKPTSRDSFAWQTATNPSVFVKMTDPPAQVSVPFMSPASAYQWFYDGYPTFGEHL..QAND
QYC65036.1     ...ELVPQLLQYMYVPPGAPKPTSRDSFAWQTATNPSVFVKMTDPPAQVSVPFMSPASAYQWFYDGYPTFGEHL..QAND
QTA73913.1     ...VLVPQLLQYMYVPPGAPKPTSRDSFAWQTATNPSVFVKMTDPPAQVSVPFMSPASAYQWFYDGYPTFGEHL..QAND
QWY15837.1     ...VLVPQLLQYMYVPPGAPKPTSRDSFAWQTATNPSVFVKMTDPPAQVSVPFMSPASAYQWFYDGYPTFGEHL..QAND
QWY15738.1     ...VLVPQLLQYMYVPPGAPKPTSRDSFAWQTATNPSVFVKMTDPPAQVSVPFMSPASAYQWFYDGYPTFGEHL..QAND
QTA73911.1     ...VLVPQLLQYMYVPPGAPKPTSRDSFAWQTATNPSVFVKMTDPPAQVSVPFMSPASAYQWFYDGYPTFGEHL..QAND
QTA73910.1     ...VLVPQLLQYMYVPPGAPKPTSRDSFAWQTATNPSVFVKMTDPPAQVSVPFMSPASAYQWFYDGYPTFGEHL..QAND
QWY15916.1     ...VLVPQLLQYMYVPPGAPKPTSRDSFAWQTATNPSVFVKMTDPPAQVSVPFMSPASAYQWFYDGYPTFGEHL..QAND
QWY15915.1     ...VLVPQLLQYMYVPPGAPKPTSRDSFAWQTATNPSVFVKMTDPPAQVSVPFMSPASAYQWFYDGYPTFGEHL..QAND
QWY15835.1     ...VLVPQLLQYMYVPPGAPKPTSRDSFAWQTATNPSVFVKMTDPPAQVSVPFMSPASAYQWFYDGYPTFGEHL..QAND
QWY15832.1     ...VLVPQLLQYMYVPPGAPKPTSRDSFAWQTATNPSVFVKMTDPPAQVSVPFMSPASAYQWFYDGYPTFGEHL..QAND
QWY15830.1     ...VLVPQLLQYMYVPPGAPKPTSRDSFAWQTATNPSVFVKMTDPPAQVSVPFMSPASAYQWFYDGYPTFGEHL..QAND
QWY15829.1     ...VLVPQLLQYMYVPPGAPKPTSRDSFAWQTATNPSVFVKMTDPPAQVSVPFMSPASAYQWFYDGYPTFGEHL..QAND
QWY15826.1     ...VLVPQLLQYMYVPPGAPKPTSRDSFAWQTATNPSVFVKMTDPPAQVSVPFMSPASAYQWFYDGYPTFGEHL..QAND
QWY15802.1     ...VLVPQLLQYMYVPPGAPKPTSRDSFAWQTATNPSVFVKMTDPPAQVSVPFMSPASAYQWFYDGYPTFGEHL..QAND
QWY15799.1     ...VLVPQLLQYMYVPPGAPKPTSRDSFAWQTATNPSVFVKMTDPPAQVSVPFMSPASAYQWFYDGYPTFGEHL..QAND
QWY15788.1     ...VLVPQLLQYMYVPPGAPKPTSRDSFAWQTATNPSVFVKMTDPPAQVSVPFMSPASAYQWFYDGYPTFGEHL..QAND
QWY15783.1     ...VLVPQLLQYMYVPPGAPKPTSRDSFAWQTATNPSVFVKMTDPPAQVSVPFMSPASAYQWFYDGYPTFGEHL..QAND
QWY15768.1     ...VLVPQLLQYMYVPPGAPKPTSRDSFAWQTATNPSVFVKMTDPPAQVSVPFMSPASAYQWFYDGYPTFGEHL..QAND
QWY15766.1     ...VLVPQLLQYMYVPPGAPKPTSRDSFAWQTATNPSVFVKMTDPPAQVSVPFMSPASAYQWFYDGYPTFGEHL..QAND
QWY15764.1     ...VLVPQLLQYMYVPPGAPKPTSRDSFAWQTATNPSVFVKMTDPPAQVSVPFMSPASAYQWFYDGYPTFGEHL..QAND
QWY15763.1     ...VLVPQLLQYMYVPPGAPKPTSRDSFAWQTATNPSVFVKMTDPPAQVSVPFMSPASAYQWFYDGYPTFGEHL..QAND
QWY15760.1     ...VLVPQLLQYMYVPPGAPKPTSRDSFAWQTATNPSVFVKMTDPPAQVSVPFMSPASAYQWFYDGYPTFGEHL..QAND
QWF08768.1     ...VLVPQLLQYMYVPPGAPKPTSRDSFAWQTATNPSVFVKMTDPPAQVSVPFMSPASAYQWFYDGYPTFGEHL..QAND
QWF08766.1     ...VLVPQLLQYMYVPPGAPKPTSRDSFAWQTATNPSVFVKMTDPPAQVSVPFMSPASAYQWFYDGYPTFGEHL..QAND
QWY15790.1     ...VLVPQLLQYMYVPPGAPKPTSRDSFAWQTATNPSVFVKMTDPPAQVSVPFMSPASAYQWFYDGYPTFGEHL..QAND
QWF08769.1     ...VLVPQLLQYMYVPPGAPKPTSRDSFAWQTATNPSVFVKMTDPPAQVSVPFMSPASAYQWFYDGYPTFGEHL..QAND
QWY15930.1     ...VLVPQLLQYMYVPPGAPKPTSRDSFAWQTATNPSVFVKMTDPPAQVSVPFMSPASAYQWFYDGYPTFGEHL..QAND
QWY15833.1     ...VLVPQLLQYMYVPPGAPRPTSRDSFAWQTATNPSVFVKMTDPPAQVSVPFMSPASAYQWFYDGYPTFGEHL..QAND
QWY15827.1     ...VLVPQLLQYMYVPPGAPRPTSRDSFAWQTATNPSVFVKMTDPPAQVSVPFMSPASAYQWFYDGYPTFGEHL..QAND
QWY15838.1     ...ELVPQLLQYMYVPPGAPRPTSRDSFAWQTATNPSVFVKMTDPPAQVSVPFMSPASAYQWFYDGYPTFGEHL..QAND
BBU37460.1     ...VLVPQLLQYMYVPPGAPKPTSRDSFAWQTATNPSVFVKMTDPPAQVSVPFMSPASAYQWFYDGYPTFGEHL..QAND
QWF08920.1     ...ELVPQLLQYMYVPPGAPKPTSRDSFAWQTATNPSVFVKMTDPPAQVSVPFMSPASAYQWFYDGYPTFGEHL..QAND
QWY15769.1     ...TLVPQLLQYMYVPPGAPKPTSRDSFAWQTATNPSVFVKMTDPPAQVSVPFMSPASAYQWFYDGYPTFGEHL..QAND
QWY15755.1     ...TLVPQLLQYMYVPPGAPKPTSRDSFAWQTATNPSVFVKMTDPPAQVSVPFMSPASAYQWFYDGYPTFGEHL..QAND
QWY15702.1     ...ALVPQLLQYMYVPPGAPKPTSRDSFAWQTATNPSVFVKMTDPPAQVSVPFMSPASAYQWFYDGYPTFGEHL..QAND
QWY15739.1     ...ALVPQLLQYMYVPPGAPKPTSRDSFAWQTATNPSVFVKMTDPPAQVSVPFMSPASAYQWFYDGYPTFGEHL..QAND
QWY15737.1     ...GLVPQLLQYMYVPPGAPKPTSRDSFAWQTATNPSVFVKMTDPPAQVSVPFMSPASAYQWFYDGYPTFGEHL..QAND
QWF08788.1     ...ELVPQLLQYMYVPPGAPKPTSRDSFAWQTATNPSVFVKMTDPPAQVSVPFMSPASAYQWFYDGYPTFGEHL..QAND
QYC65122.1     ...ELVPQLLQYMYVPPGAPKPTSRDSFAWQTATNPSVFVKMTDPPAQVSVPFMSPASAYQWFYDGYPTFGEHL..QAND
QWF08891.1     ...ELVPQLLQYMYVPPGAPKPTSRDSFAWQTATNPSVFVKMTDPPAQVSVPFMSPASAYQWFYDGYPTFGEHL..QAND
QWY15903.1     ...ELVPQLLQYMYVPPGAPKPTSRDSFAWQTATNPSVFVKMTDPPAQVSVPFMSPASAYQWFYDGYPTFGEHL..QAND
BCI65366.1     ...ELVPQLLQYMYVPPGAPKPTSRDSFAWQTATNPSVFVKMTDPPAQVSVPFMSPASAYQWFYDGYPTFGEHL..QAND
BCG67045.1     ...ELVPQLLQYMYVPPGAPKPTSRDSFAWQTATNPSVFVKMTDPPAQVSVPFMSPASAYQWFYDGYPTFGEHL..QAND
BCG67044.1     ...ELVPQLLQYMYVPPGAPKPTSRDSFAWQTATNPSVFVKMTDPPAQVSVPFMSPASAYQWFYDGYPTFGEHL..QAND
QWY15819.1     ...ELVPQLLQYMYVPPGAPKPTSRDSFAWQTATNPSVFVKMTDPPAQVSVPFMSPASAYQWFYDGYPTFGEHL..QAND
QWY15818.1     ...ELVPQLLQYMYVPPGAPKPTSRDSFAWQTATNPSVFVKMTDPPAQVSVPFMSPASAYQWFYDGYPTFGEHL..QAND
QTA73912.1     ...VLVPQLLQYMYVPPGAPKPTSRDSFAWQTATNPSVFVKMTDPPAQVSVPFMSPASAYQWFYDGYPTFGEHL..QAND
QWY15914.1     ...VLVPQLLQYMYVPPGAPKPTSRDSFAWQTATNPSVFVKMTDPPAQVSVPFMSPASAYQWFYDGYPTFGEHL..QAND
QWY15909.1     ...VLVPQLLQYMYVPPGAPKPTSRDSFAWQTATNPSVFVKMTDPPAQVSVPFMSPASAYQWFYDGYPTFGEHL..QAND
QWY15905.1     ...VLVPQLLQYMYVPPGAPKPTSRDSFAWQTATNPSVFVKMTDPPAQVSVPFMSPASAYQWFYDGYPTFGEHL..QAND
QWY15856.1     ...VLVPQLLQYMYVPPGAPKPTSRDSFAWQTATNPSVFVKMTDPPAQVSVPFMSPASAYQWFYDGYPTFGEHL..QAND
QWY15850.1     ...VLVPQLLQYMYVPPGAPKPTSRDSFAWQTATNPSVFVKMTDPPAQVSVPFMSPASAYQWFYDGYPTFGEHL..QAND
QWY15849.1     ...VLVPQLLQYMYVPPGAPKPTSRDSFAWQTATNPSVFVKMTDPPAQVSVPFMSPASAYQWFYDGYPTFGEHL..QAND
QWY15847.1     ...VLVPQLLQYMYVPPGAPKPTSRDSFAWQTATNPSVFVKMTDPPAQVSVPFMSPASAYQWFYDGYPTFGEHL..QAND
QWY15845.1     ...VLVPQLLQYMYVPPGAPKPTSRDSFAWQTATNPSVFVKMTDPPAQVSVPFMSPASAYQWFYDGYPTFGEHL..QAND
QWY15836.1     ...VLVPQLLQYMYVPPGAPKPTSRDSFAWQTATNPSVFVKMTDPPAQVSVPFMSPASAYQWFYDGYPTFGEHL..QAND
QWY15765.1     ...VLVPQLLQYMYVPPGAPKPTSRDSFAWQTATNPSVFVKMTDPPAQVSVPFMSPASAYQWFYDGYPTFGEHL..QAND
QWY15734.1     ...VLVPQLLQYMYVPPGAPKPTSRDSFAWQTATNPSVFVKMTDPPAQVSVPFMSPASAYQWFYDGYPTFGEHL..QAND
QWY15855.1     ...VLVPQLLQYMYVPPGAPKPTSRDSFAWQTATNPSVFVKMTDPPAQVSVPFMSPASAYQWFYDGYPTFGEHL..QAND
QWY15831.1     ...VLVPQLLQYMYVPPGAPKPTSRDSFAWQTATNPSVFVKMTDPPAQVSVPFMSPASAYQWFYDGYPTFGEHL..QAND
QWY15798.1     ...VLVPQLLQYMYVPPGAPKPTSRDSFAWQTATNPSVFVKMTDPPAQVSVPFMSPASAYQWFYDGYPTFGEHL..QAND
QWY15797.1     ...VLVPQLLQYMYVPPGAPKPTSRDSFAWQTATNPSVFVKMTDPPAQVSVPFMSPASAYQWFYDGYPTFGEHL..QAND
QWY15796.1     ...VLVPQLLQYMYVPPGAPKPTSRDSFAWQTATNPSVFVKMTDPPAQVSVPFMSPASAYQWFYDGYPTFGEHL..QAND
QWY15795.1     ...VLVPQLLQYMYVPPGAPKPTSRDSFAWQTATNPSVFVKMTDPPAQVSVPFMSPASAYQWFYDGYPTFGEHL..QAND
QWY15794.1     ...VLVPQLLQYMYVPPGAPKPTSRDSFAWQTATNPSVFVKMTDPPAQVSVPFMSPASAYQWFYDGYPTFGEHL..QAND
QWY15971.1     ...ELVPQLLQYMYVPPGAPKPTSRDSFAWQTATNPSVFVKMTDPPAQVSVPFMSPASAYQWFYDGYPTFGEHL..QAND
QWY15897.1     ...ELVPQLLQYMYVPPGAPKPTSRDSFAWQTATNPSVFVKMTDPPAQVSVPFMSPASAYQWFYDGYPTFGEHL..QAND
QWY15742.1     ...VLVPQLLQYMYVPPGAPKPTSRDSFAWQTATNPSVFVKMTDPPAQVSVPFMSPASAYQWFYDGYPTFGEHL..QAND
QWY15741.1     ...VLVPQLLQYMYVPPGAPKPTSRDSFAWQTATNPSVFVKMTDPPAQVSVPFMSPASAYQWFYDGYPTFGEHL..QAND
QWY15923.1     ...ELVPQLLQYMYVPPGAPKPTSRDSFAWQTATNPSVFVKMTDPPAQVSVPFMSPASAYQWFYDGYPTFGEHL..QAND
QWF08957.1     ...ELVPQLLQYMYVPPGAPKPTSRDSFAWQTATNPSVFVKMTDPPAQVSVPFMSPASAYQWFYDGYPTFGEHL..QAND
UEP53407.1     ...ELVPQLLQYMYVPPGAPKPTSRDSFAWQTATNPSVFVKMTDPPAQVSVPFMSPASAYQWFYDGYPTFGEHL..QAND
QWY15922.1     ...VLVPQLLQYMYVPPGAPKPTSRDSFAWQTATNPSVFVKMTDPPAQVSVPFMSPASAYQWFYDGYPTFGEHL..QAND
QWY15848.1     ...VLVPQLLQYMYVPPGAPKPTSRDSFAWQTATNPSVFVKMTDPPAQVSVPFMSPASAYQWFYDGYPTFGEHL..QAND
UEP53405.1     ...ELVPQLLQYMYVPPGAPKPTSRDSFAWQTATNPSVFVKMTDPPAQVSVPFMSPASAYQWFYDGYPTFGEHL..QAND
UEC49855.1     ...ELVPQLLQYMYVPPGAPKPTSRDSFAWQTATNPSVFVKMTDPPAQVSVPFMSPASAYQWFYDGYPTFGEHL..QAND
QWY15851.1     ...ELVPQLLQYMYVPPGAPKPTSRDSFAWQTATNPSVFVKMTDPPAQVSVPFMSPASAYQWFYDGYPTFGEHL..QAND
QYC65112.1     ...ELVPQLLQYMYVPPGAPKPTSRDSFAWQTATNPSVFVKMTDPPAQVSVPFMSPASAYQWFYDGYPTFGEHL..QAND
QOP74942.1     ...ELVPQLLQYMYVPPGAPKPTSRDSFAWQTATNPSVFVKMTDPPAQVSVPFMSPASAYQWFYDGYPTFGEHL..QAND
QLH02013.1     ...ELVPQLLQYMYVPPGAPKPTSRDSFAWQTATNPSVFVKMTDPPAQVSVPFMSPASAYQWFYDGYPTFGEHL..QAND
QIH53923.1     ...ELVPQLLQYMYVPPGAPKPTSRDSFAWQTATNPSVFVKMTDPPAQVSVPFMSPASAYQWFYDGYPTFGEHL..QAND
QIH53918.1     ...ELVPQLLQYMYVPPGAPKPTSRDSFAWQTATNPSVFVKMTDPPAQVSVPFMSPASAYQWFYDGYPTFGEHL..QAND
QIH53915.1     ...ELVPQLLQYMYVPPGAPKPTSRDSFAWQTATNPSVFVKMTDPPAQVSVPFMSPASAYQWFYDGYPTFGEHL..QAND
QIH53914.1     ...ELVPQLLQYMYVPPGAPKPTSRDSFAWQTATNPSVFVKMTDPPAQVSVPFMSPASAYQWFYDGYPTFGEHL..QAND
QYC65127.1     ...ELVPQLLQYMYVPPGAPKPTSRDSFAWQTATNPSVFVKMTDPPAQVSVPFMSPASAYQWFYDGYPTFGEHL..QAND
QIL87534.1     ...ELVPQLLQYMYVPPGAPKPTSRDSFAWQTATNPSVFVKMTDPPAQVSVPFMSPASAYQWFYDGYPTFGEHL..QAND
QWF08936.1     ...ELVPQLLQYMYVPPGAPKPTSRDSFAWQTATNPSVFVKMTDPPAQVSVPFMSPASAYQWFYDGYPTFGEHL..QAND
QIL87532.1     ...ELVPQLLQYMYVPPGAPKPTSRDSFAWQTATNPSVFVKMTDPPAQVSVPFMSPASAYQWFYDGYPTFGEHL..QAND
QYC65089.1     ...ELVPQLLQYMYVPPGAPKPTSRDSFAWQTATNPSVFVKMTDPPAQVSVPFMSPASAYQWFYDGYPTFGEHL..QAND
QWF08950.1     ...ELVPQLLQYMYVPPGAPKPTSRDSFAWQTATNPSVFVKMTDPPAQVSVPFMSPASAYQWFYDGYPTFGEHL..QAND
QIL87530.1     ...ELVPQLLQYMYVPPGAPKPTSRDSFAWQTATNPSVFVKMTDPPAQVSVPFMSPASAYQWFYDGYPTFGEHL..QAND
QWF08820.1     ...ELVPQLLQYMYVPPGAPKPTSRDSFAWQTATNPSVFVKMTDPPAQVSVPFMSPASAYQWFYDGYPTFGEHL..QAND
QIL87529.1     ...ELVPQLLQYMYVPPGAPKPTSRDSFAWQTATNPSVFVKMTDPPAQVSVPFMSPASAYQWFYDGYPTFGEHL..QAND
QIL87528.1     ...ELVPQLLQYMYVPPGAPKPTSRDSFAWQTATNPSVFVKMTDPPAQVSVPFMSPASAYQWFYDGYPTFGEHL..QAND
QYC65143.1     ...ELVPQLLQYMYVPPGAPKPTSRDSFAWQTATNPSVFVKMTDPPAQVSVPFMSPASAYQWFYDGYPTCGEHL..QAND
QIL87525.1     ...ELVPQLLQYMYVPPGAPKPTSRDSFAWQTATNPSVFVKMTDPPAQVSVPFMSPASAYQWFYDGYPTFGEHL..QAND
QYC65141.1     ...ELVPQLLQYMYVPPGAPKPTSRDSFAWQTATNPSVFVKMTDPPAQVSVPFMSPASAYQWFYDGYPTFGEHL..QAND
QYC65084.1     ...ELVPQLLQYMYVPPGAPKPTSRDSFAWQTATNPSVFVKMTDPPAQVSVPFMSPASAYQWFYDGYPTFGEHL..QAND
QWF08947.1     ...ELVPQLLQYMYVPPGAPKPTSRDSFAWQTATNPSVFVKMTDPPAQVSVPFMSPASAYQWFYDGYPTFGEHL..QAND
QWF08922.1     ...ELVPQLLQYMYVPPGAPKPTSRDSFAWQTATNPSVFVKMTDPPAQVSVPFMSPASAYQWFYDGYPTFGEHL..QAND
QIL87524.1     ...ELVPQLLQYMYVPPGAPKPTSRDSFAWQTATNPSVFVKMTDPPAQVSVPFMSPASAYQWFYDGYPTFGEHL..QAND
QIL87520.1     ...ELVPQLLQYMYVPPGAPKPTSRDSFAWQTATNPSVFVKMTDPPAQVSVPFMSPASAYQWFYDGYPTFGEHL..QAND
QIL87518.1     ...ELVPQLLQYMYVPPGAPKPTSRDSFAWQTATNPSVFVKMTDPPAQVSVPFMSPASAYQWFYDGYPTFGEHL..QAND
QYC65103.1     ...ELVPQLLQYMYVPPGAPKPTSRDSFAWQTATNPSVFVKMTDPPAQVSVPFMSPASAYQWFYDGYPTFGEHL..QAND
QWF08944.1     ...ELVPQLLQYMYVPPGAPKPTSRDSFAWQTATNPSVFVKMTDPPAQVSVPFMSPASAYQWFYDGYPTFGEHL..QAND
QWF08943.1     ...ELVPQLLQYMYVPPGAPKPTSRDSFAWQTATNPSVFVKMTDPPAQVSVPFMSPASAYQWFYDGYPTFGEHL..QAND
QWF08927.1     ...ELVPQLLQYMYVPPGAPKPTSRDSFAWQTATNPSVFVKMTDPPAQVSVPFMSPASAYQWFYDGYPTFGEHL..QAND
QWF08942.1     ...ELVPQLLQYMYVPPGAPKPTSRDSFAWQTATNPSVFVKMTDPPAQVSVPFMSPASAYQWFYDGYPTFGEHL..QAND
QWF08940.1     ...ELVPQLLQYMYVPPGAPKPTSRDSFAWQTATNPSVFVKMTDPPAQVSVPFMSPASAYQWFYDGYPTFGEHL..QAND
QWF08912.1     ...ELVPQLLQYMYVPPGAPKPTSRDSFAWQTATNPSVFVKMTDPPAQVSVPFMSPASAYQWFYDGYPTFGEHL..QAND
QWF08907.1     ...ELVPQLLQYMYVPPGAPKPTSRDSFAWQTATNPSVFVKMTDPPAQVSVPFMSPASAYQWFYDGYPTFGEHL..QAND
QWF08904.1     ...ELVPQLLQYMYVPPGAPKPTSRDSFAWQTATNPSVFVKMTDPPAQVSVPFMSPASAYQWFYDGYPTFGEHL..QAND
QWF08886.1     ...ELVPQLLQYMYVPPGAPKPTSRDSFAWQTATNPSVFVKMTDPPAQVSVPFMSPASAYQWFYDGYPTFGEHL..QAND
QWY15877.1     ...ELVPQLLQYMYVPPGAPKPTSRDSFAWQTATNPSVFVKMTDPPAQVSVPFMSPASAYQWFYDGYPTFGEHL..QAND
QWY15965.1     ...ELVPQLLQYMYVPPGAPKPTSRDSFAWQTATNPSVFVKMTDPPAQVSVPFMSPASAYQWFYDGYPTFGEHL..QAND
UEC49851.1     ...ELVPQLLQYMYVPPGAPKPTSRDSFAWQTATNPSVFVKMTDPPAQVSVPFMSPASAYQWFYDGYPTFGEHL..QAND
UEC49850.1     ...ELVPQLLQYMYVPPGAPKPTSRDSFAWQTATNPSVFVKMTDPPAQVSVPFMSPASAYQWFYDGYPTFGEHL..QAND
UEC49849.1     ...ELVPQLLQYMYVPPGAPKPTSRDSFAWQTATNPSVFVKMTDPPAQVSVPFMSPASAYQWFYDGYPTFGEHL..QAND
QWY15963.1     ...ELVPQLLQYMYVPPGAPKPTSRDSFAWQTATNPSVFVKMTDPPAQVSVPFMSPASAYQWFYDGYPTFGEHL..QAND
QWY15972.1     ...ELVPQLLQYMYVPPGAPKPTSRDSFAWQTATNPSVFVKMTDPPAQVSVPFMSPASAYQWFYDGYPTFGEHL..QAND
QWY15958.1     ...ELVPQLLQYMYVPPGAPKPTSRDSFAWQTATNPSVFVKMTDPPAQVSVPFMSPASAYQWFYDGYPTFGEHL..QAND
QWY15899.1     ...ELVPQLLQYMYVPPGAPKPTSRDSFAWQTATNPSVFVKMTDPPAQVSVPFMSPASAYQWFYDGYPTFGEHL..QAND
QWY15898.1     ...ELVPQLLQYMYVPPGAPKPTSRDSFAWQTATNPSVFVKMTDPPAQVSVPFMSPASAYQWFYDGYPTFGEHL..QAND
QWY15895.1     ...ELVPQLLQYMYVPPGAPKPTSRDSFAWQTATNPSVFVKMTDPPAQVSVPFMSPASAYQWFYDGYPTFGEHL..QAND
QWY15893.1     ...ELVPQLLQYMYVPPGAPKPTSRDSFAWQTATNPSVFVKMTDPPAQVSVPFMSPASAYQWFYDGYPTFGEHL..QAND
QWY15892.1     ...ELVPQLLQYMYVPPGAPKPTSRDSFAWQTATNPSVFVKMTDPPAQVSVPFMSPASAYQWFYDGYPTFGEHL..QAND
QWY15891.1     ...ELVPQLLQYMYVPPGAPKPTSRDSFAWQTATNPSVFVKMTDPPAQVSVPFMSPASAYQWFYDGYPTFGEHL..QAND
QWY15970.1     ...ELVPQLLQYMYVPPGAPKPTSRDSFAWQTATNPSVFVKMTDPPAQVSVPFMSPASAYQWFYDGYPTFGEHL..QAND
QWY15966.1     ...ELVPQLLQYMYVPPGAPKPTSRDSFAWQTATNPSVFVKMTDPPAQVSVPFMSPASAYQWFYDGYPTFGEHL..QAND
QWY15962.1     ...ELVPQLLQYMYVPPGAPKPTSRDSFAWQTATNPSVFVKMTDPPAQVSVPFMSPASAYQWFYDGYPTFGEHL..QAND
QWY15961.1     ...ELVPQLLQYMYVPPGAPKPTSRDSFAWQTATNPSVFVKMTDPPAQVSVPFMSPASAYQWFYDGYPTFGEHL..QAND
QWY15925.1     ...ELVPQLLQYMYVPPGAPKPTSRDSFAWQTATNPSVFVKMTDPPAQVSVPFMSPASAYQWFYDGYPTFGEHL..QAND
QWY15773.1     ...ELVPQLLQYMYVPPGAPKPTSRDSFAWQTATNPSVFVKMTDPPAQVSVPFMSPASAYQWFYDGYPTFGEHL..QAND
QWY15924.1     ...ELVPQLLQYMYVPPGAPKPTSRDSFAWQTATNPSVFVKMTDPPAQVSVPFMSPASAYQWFYDGYPTFGEHL..QAND
QWY15910.1     ...ELVPQLLQYMYVPPGAPKPTSRDSFAWQTATNPSVFVKMTDPPAQVSVPFMSPASAYQWFYDGYPTFGEHL..QAND
```

```
QWY15824.1    ...ELVPQLLQYMYVPPGAPKPTSRDSFAWQTATNPSVFVKMTDPPAQVSVPFMSPASAYQWFYDGYPTFGEHL..QAND
QWY15823.1    ...ELVPQLLQYMYVPPGAPKPTSRDSFAWQTATNPSVFVKMTDPPAQVSVPFMSPASAYQWFYDGYPTFGEHL..QAND
QWY15822.1    ...ELVPQLLQYMYVPPGAPKPTSRDSFAWQTATNPSVFVKMTDPPAQVSVPFMSPASAYQWFYDGYPTFGEHL..QAND
QWY15821.1    ...ELVPQLLQYMYVPPGAPKPTSRDSFAWQTATNPSVFVKMTDPPAQVSVPFMSPASAYQWFYDGYPTFGEHL..QAND
QWY15816.1    ...ELVPQLLQYMYVPPGAPKPTSRDSFAWQTATNPSVFVKMTDPPAQVSVPFMSPASAYQWFYDGYPTFGEHL..QAND
QWY15778.1    ...ELVPQLLQYMYVPPGAPKPTSRDSFAWQTATNPSVFVKMTDPPAQVSVPFMSPASAYQWFYDGYPTFGEHL..QAND
QWY15801.1    ...ELVPQLLQYMYVPPGAPKPTSRDSFAWQTATNPSVFVKMTDPPAQVSVPFMSPASAYQWFYDGYPTFGEHL..QAND
QWY15791.1    ...ELVPQLLQYMYVPPGAPKPTSRDSFAWQTATNPSVFVKMTDPPAQVSVPFMSPASAYQWFYDGYPTFGEHL..QAND
QWY15775.1    ...ELVPQLLQYMYVPPGAPKPTSRDSFAWQTATNPSVFVKMTDPPAQVSVPFMSPASAYQWFYDGYPTFGEHL..QAND
QWY15774.1    ...ELVPQLLQYMYVPPGAPKPTSRDSFAWQTATNPSVFVKMTDPPAQVSVPFMSPASAYQWFYDGYPTFGEHL..QAND
BCR43107.1    ...ELVPQLLQYMYVPPGAPKPTSRDSFAWQTATNPSVFVKMTDPPAQVSVPFMSPASAYQWFYDGYPTFGEHL..QAND
QYC65183.1    ...ELVPQLLQYMYVPPGAPKPTSRDSFAWQTATNPSVFVKMTDPPAQVSVPFMSPASAYQWFYDGYPTFGEHL..QAND
BCR43094.1    ...ELVPQLLQYMYVPPGAPKPTSRDSFAWQTATNPSVFVKMTDPPAQVSVPFMSPASAYQWFYDGYPTFGEHL..QAND
BCR43081.1    ...ELVPQLLQYMYVPPGAPKPTSRDSFAWQTATNPSVFVKMTDPPAQVSVPFMSPASAYQWFYDGYPTFGEHL..QAND
BCG67017.1    ...ELVPQLLQYMYVPPGAPKPTSRDSFAWQTATNPSVFVKMTDPPAQVSVPFMSPASAYQWFYDGYPTFGEHL..QAND
BCG67015.1    ...ELVPQLLQYMYVPPGAPKPTSRDSFAWQTATNPSVFVKMTDPPAQVSVPFMSPASAYQWFYDGYPTFGEHL..QAND
BCG67007.1    ...ELVPQLLQYMYVPPGAPKPTSRDSFAWQTATNPSVFVKMTDPPAQVSVPFMSPASAYQWFYDGYPTFGEHL..QAND
BCG67005.1    ...ELVPQLLQYMYVPPGAPKPTSRDSFAWQTATNPSVFVKMTDPPAQVSVPFMSPASAYQWFYDGYPTFGEHL..QAND
QYC65184.1    ...ELVPQLLQYMYVPPGAPKPTSRDSFAWQTATNPSVFVKMTDPPAQVSVPFMSPASAYQWFYDGYPTFGEHL..QAND
QWF08853.1    ...ELVPQLLQYMYVPPGAPKPTSRDSFAWQTATNPSVFVKMTDPPAQVSVPFMSPASAYQWFYDGYPTFGEHL..QAND
QYC65140.1    ...ELVPQLLQYMYVPPGAPKPTSRDSFAWQTATNPSVFVKMTDPPAQVSVPFMSPASAYQWFYDGYPTFGEHL..QAND
QYC65133.1    ...ELVPQLLQYMYVPPGAPKPTSRDSFAWQTATNPSVFVKMTDPPAQVSVPFMSPASAYQWFYDGYPTFGEHL..QAND
QYC65110.1    ...ELVPQLLQYMYVPPGAPKPTSRDSFAWQTATNPSVFVKMTDPPAQVSVPFMSPASAYQWFYDGYPTFGEHL..QAND
QYC65108.1    ...ELVPQLLQYMYVPPGAPKPTSRDSFAWQTATNPSVFVKMTDPPAQVSVPFMSPASAYQWFYDGYPTFGEHL..QAND
QYC65098.1    ...ELVPQLLQYMYVPPGAPKPTSRDSFAWQTATNPSVFVKMTDPPAQVSVPFMSPASAYQWFYDGYPTFGEHL..QAND
QYC65097.1    ...ELVPQLLQYMYVPPGAPKPTSRDSFAWQTATNPSVFVKMTDPPAQVSVPFMSPASAYQWFYDGYPTFGEHL..QAND
QYC65094.1    ...ELVPQLLQYMYVPPGAPKPTSRDSFAWQTATNPSVFVKMTDPPAQVSVPFMSPASAYQWFYDGYPTFGEHL..QAND
QYC65091.1    ...ELVPQLLQYMYVPPGAPKPTSRDSFAWQTATNPSVFVKMTDPPAQVSVPFMSPASAYQWFYDGYPTFGEHL..QAND
QYC65057.1    ...ELVPQLLQYMYVPPGAPKPTSRDSFAWQTATNPSVFVKMTDPPAQVSVPFMSPASAYQWFYDGYPTFGEHL..QAND
QWF08850.1    ...ELVPQLLQYMYVPPGAPKPTSRDSFAWQTATNPSVFVKMTDPPAQVSVPFMSPASAYQWFYDGYPTFGEHL..QAND
QYC65056.1    ...ELVPQLLQYMYVPPGAPKPTSRDSFAWQTATNPSVFVKMTDPPAQVSVPFMSPASAYQWFYDGYPTFGEHL..QAND
QWF08849.1    ...ELVPQLLQYMYVPPGAPKPTSRDSFAWQTATNPSVFVKMTDPPAQVSVPFMSPASAYQWFYDGYPTFGEHL..QAND
QWF08969.1    ...ELVPQLLQYMYVPPGAPKPTSRDSFAWQTATNPSVFVKMTDPPAQVSVPFMSPASAYQWFYDGYPTFGEHL..QAND
QWF08848.1    ...ELVPQLLQYMYVPPGAPKPTSRDSFAWQTATNPSVFVKMTDPPAQVSVPFMSPASAYQWFYDGYPTFGEHL..QAND
QWF08968.1    ...ELVPQLLQYMYVPPGAPKPTSRDSFAWQTATNPSVFVKMTDPPAQVSVPFMSPASAYQWFYDGYPTFGEHL..QAND
QWF08965.1    ...ELVPQLLQYMYVPPGAPKPTSRDSFAWQTATNPSVFVKMTDPPAQVSVPFMSPASAYQWFYDGYPTFGEHL..QAND
QWF08956.1    ...ELVPQLLQYMYVPPGAPKPTSRDSFAWQTATNPSVFVKMTDPPAQVSVPFMSPASAYQWFYDGYPTFGEHL..QAND
QWF08952.1    ...ELVPQLLQYMYVPPGAPKPTSRDSFAWQTATNPSVFVKMTDPPAQVSVPFMSPASAYQWFYDGYPTFGEHL..QAND
QWF08903.1    ...ELVPQLLQYMYVPPGAPKPTSRDSFAWQTATNPSVFVKMTDPPAQVSVPFMSPASAYQWFYDGYPTFGEHL..QAND
QWF08902.1    ...ELVPQLLQYMYVPPGAPKPTSRDSFAWQTATNPSVFVKMTDPPAQVSVPFMSPASAYQWFYDGYPTFGEHL..QAND
QWF08895.1    ...ELVPQLLQYMYVPPGAPKPTSRDSFAWQTATNPSVFVKMTDPPAQVSVPFMSPASAYQWFYDGYPTFGEHL..QAND
QWF08888.1    ...ELVPQLLQYMYVPPGAPKPTSRDSFAWQTATNPSVFVKMTDPPAQVSVPFMSPASAYQWFYDGYPTFGEHL..QAND
QWF08893.1    ...ELVPQLLQYMYVPPGAPKPTSRDSFAWQTATNPSVFVKMTDPPAQVSVPFMSPASAYQWFYDGYPTFGEHL..QAND
QWF08892.1    ...ELVPQLLQYMYVPPGAPKPTSRDSFAWQTATNPSVFVKMTDPPAQVSVPFMSPASAYQWFYDGYPTFGEHL..QAND
QWF08876.1    ...ELVPQLLQYMYVPPGAPKPTSRDSFAWQTATNPSVFVKMTDPPAQVSVPFMSPASAYQWFYDGYPTFGEHL..QAND
QWF08868.1    ...ELVPQLLQYMYVPPGAPKPTSRDSFAWQTATNPSVFVKMTDPPAQVSVPFMSPASAYQWFYDGYPTFGEHL..QAND
QWF08862.1    ...ELVPQLLQYMYVPPGAPKPTSRDSFAWQTATNPSVFVKMTDPPAQVSVPFMSPASAYQWFYDGYPTFGEHL..QAND
QWF08861.1    ...ELVPQLLQYMYVPPGAPKPTSRDSFAWQTATNPSVFVKMTDPPAQVSVPFMSPASAYQWFYDGYPTFGEHL..QAND
QWF08860.1    ...ELVPQLLQYMYVPPGAPKPTSRDSFAWQTATNPSVFVKMTDPPAQVSVPFMSPASAYQWFYDGYPTFGEHL..QAND
QWF08859.1    ...ELVPQLLQYMYVPPGAPKPTSRDSFAWQTATNPSVFVKMTDPPAQVSVPFMSPASAYQWFYDGYPTFGEHL..QAND
QWF08858.1    ...ELVPQLLQYMYVPPGAPKPTSRDSFAWQTATNPSVFVKMTDPPAQVSVPFMSPASAYQWFYDGYPTFGEHL..QAND
QWF08854.1    ...ELVPQLLQYMYVPPGAPKPTSRDSFAWQTATNPSVFVKMTDPPAQVSVPFMSPASAYQWFYDGYPTFGEHL..QAND
QWF08929.1    ...ELVPQLLQYMYVPPGAPKPTSRDSFAWQTATNPSVFVKMTDPPAQVSVPFMSPASAYQWFYDGYPTFGEHL..QAND
QYC65137.1    ...ELVPQLLQYMYVPPGAPKPTSRDSFAWQTATNPSVFVKMTDPPAQVSVPFMSPASAYQWFYDGYPTFGEHL..QVND
QYC65100.1    ...ELVPQLLQYMYVPPGAPKPTSRDSFAWQTATNPSVFVKMTDPPAQVSVPFMSPASAYQWFYDGYPTFGEHL..QAND
QWY15926.1    ...ELVPQLLQYMYVPPGAPKPTSRDSFAWQTATNPSVFVKMTDPPAQVSVPFMSPASAYQWFYDGYPTFGEHL..QAND
QWF08962.1    ...ELVPQLLQYMYVPPGAPKPNSRDSFAWQTATNPSVFVKMTDPPAQVSVPFMSPASAYQWFYDGYPTFGEHL..QAND
QWF08909.1    ...ELVPQLLQYMYVPPGAPKPISRDSFAWQTATNPSVFVKMTDPPAQVSVPFMSPASAYQWFYDGYPTFGEHL..QAND
QWY15854.1    ...ELVPQLLQYMYVPPGAPKPTSRDSFAWQTATNPSVFVKMTDPPAQVSVPFMSPASAYQWFYDGYPTFGEHL..QAND
QWY15808.1    ...ELVPQLLQYMYVPPGAPKPTSRDSFAWQTATNPSVFVKMTDPPAQVSVPFMSPASAYQWFYDGYPTFGEHL..QAND
QWF08770.1    ...ELVPQLLQYMYVPPGAPKPTSRDSFAWQTATNPSVFVKMTDPPAQVSVPFMSPASAYQWFYDGYPTFGEHL..QAND
QWY15728.1    ...ELVPQLLQYMYVPPGAPKPTSRDSFAWQTATNPSVFVKMTDPPAQVSVPFMSPASAYQWFYDGYPTFGEHL..QAND
QYC65126.1    ...ELVPQLLQYMYVPPGAPKPTSRDSFAWQTATNPSVFVKMTDPPAQVSVPFMSPASAYQWFYDGYPTFGEHL..QAND
QWF08827.1    ...ELVPQLLQYMYVPPGAPKPTSRDSFAWQTATNPSVFVKMTDPPAQVSVPFMSPASAYQWFYDGYPTFGEHL..QAND
QYC65072.1    ...ELVPQLLQYMYVPPGAPKPTSRDSFAWQTATNPSVFVKMTDPPAQVSVPFMSPASAYQWFYDGYPTFGEHL..QAND
QYC65067.1    ...ELVPQLLQYMYVPPGAPKPTSRDSFAWQTATNPSVFVKMTDPPAQVSVPFMSPASAYQWFYDGYPTFGEHL..QAND
QWY15767.1    ...VLVPQLLQYMYVPPGAPKPTSRDSFAWQTATNPSVFVKMTDPPAQVSVPFMSPASAYQWFYDGYPTFGEHL..QAND
QWY15735.1    ...VLVPQLLQYMYVPPGAPKPTSRDSFAWQTATNPSVFVKMTDPPAQVSVPFMSPASAYQWFYDGYPTFGEHL..QAND
BCB92153.1    ...ELVPQLLQYMYVPPGAPKPTSRDSFAWQTATNPSVFVKMTDPPAQVSVPFMSPASAYQWFYDGYPTFGEHL..QAND
QWF08966.1    ...ELVPQLLQYMYVPPGAPKPTSRDSFAWQTATNPSVFVKMTDPPAQVSVPFMSPASAYQWFYDGYPTFGEHL..QAND
QIH53917.1    ...ELVPQLLQYMYVPPGAPKPTSRDSFAWQTATNPSVFVKMTDPPAQVSVPFMSPASAYQWFYDGYPTFGEHL..QAND
QYC65092.1    ...ELVPQLLQYMYVPPGAPKPTSRDSFAWQTATNPSVFVKMTDPPAQVSVPFMSPASAYQWFYDGYPTFGEHL..QAND
QWY15879.1    ...ELVPQLLQYMYVPPGAPKPTSRDSFAWQTATNPSVFVKMTDPPAQVSVPFMSPASAYQWFYDGYPTFGEHL..QAND
QWY15777.1    ...ELVPQLLQYMYVPPGAPKPTSRDSFAWQTATNPSVFVKMTDPPAQVSVPFMSPASAYQWFYDGYPTFGEHL..QAND
BCR43105.1    ...ELVPQLLQYMYVPPGAPKPTSRDSFAWQTATNPSVFVKMTDPPAQVSVPFMSPASAYQWFYDGYPTFGEHL..QAND
QWF08826.1    ...ELVPQLLQYMYVPPGAPKPTSRDSFAWQTATNPSVFVKMTDPPAQVSVPFMSPASAYQWFYDGYPTFGEHL..QAND
BCG67024.1    ...ELVPQLLQYMYVPPGAPKPTSRDSFAWQTATNPSVFVKMTDPPAQVSVPFMSPASAYQWFYDGYPTFGEHL..QAND
BCG66999.1    ...ELVPQLLQYMYVPPGAPKPTSRDSFAWQTATNPSVFVKMTDPPAQVSVPFMSPASAYQWFYDGYPTFGEHL..QAND
QWY15752.1    ...ELVPQLLQYMYVPPGAPKPTSRDSFAWQTATNPSVFVKMTDPPAQVSVPFMSPASAYQWFYDGYPTFGEHL..QAND
BCG67028.1    ...ELVPQLLQYMYVPPGAPKPTSRDSFAWQTATNPSVFVKMTDPPAQVSVPFMSPASAYQWFYDGYPTFGEHL..QAND
BCG67027.1    ...ELVPQLLQYMYVPPGAPKPTSRDSFAWQTATNPSVFVKMTDPPAQVSVPFMSPASAYQWFYDGYPTFGEHL..QAND
BCG66996.1    ...ELVPQLLQYMYVPPGAPKPTSRDSFAWQTATNPSVFVKMTDPPAQVSVPFMSPASAYQWFYDGYPTFGEHL..QAND
BCG67026.1    ...ELVPQLLQYMYVPPGAPKPTSRDSFAWQTATNPSVFVKMTDPPAQVSVPFMSPASAYQWFYDGYPTFGEHL..QAND
BCG67025.1    ...ELVPQLLQYMYVPPGAPKPTSRDSFAWQTATNPSVFVKMTDPPAQVSVPFMSPASAYQWFYDGYPTFGEHL..QAND
BCG67023.1    ...ELVPQLLQYMYVPPGAPKPTSRDSFAWQTATNPSVFVKMTDPPAQVSVPFMSPASAYQWFYDGYPTFGEHL..QAND
BCG67022.1    ...ELVPQLLQYMYVPPGAPKPTSRDSFAWQTATNPSVFVKMTDPPAQVSVPFMSPASAYQWFYDGYPTFGEHL..QAND
BCG67021.1    ...ELVPQLLQYMYVPPGAPKPTSRDSFAWQTATNPSVFVKMTDPPAQVSVPFMSPASAYQWFYDGYPTFGEHL..QAND
BCG67013.1    ...ELVPQLLQYMYVPPGAPKPTSRDSFAWQTATNPSVFVKMTDPPAQVSVPFMSPASAYQWFYDGYPTFGEHL..QAND
BCG67000.1    ...ELVPQLLQYMYVPPGAPKPTSRDSFAWQTATNPSVFVKMTDPPAQVSVPFMSPASAYQWFYDGYPTFGEHL..QAND
BCG66997.1    ...ELVPQLLQYMYVPPGAPKPTSRDSFAWQTATNPSVFVKMTDPPAQVSVPFMSPASAYQWFYDGYPTFGEHL..QAND
BCG66998.1    ...ELVPQLLQYMYVPPGAPKPTSRDSFAWQTATNPSVFVKMTDPPAQVSVPFMSPASAYQWFYDGYPTFGEHL..QAND
QWF08924.1    ...ELVPQLLQYMYVPPGAPKPTSRDSFAWQTATNPSVFVKMTDPPAQVSVPFMSPASAYQWFYDGYPTFGEHL..QAND
QIL87522.1    ...ELVPQLLQYMYVPPGAPKPTSRDSFAWQTATNPSVFVKMTDPPAQVSVPFMSPASAYQWFYDGYPTFGEHL..QAND
QYC65075.1    ...ELVPQLLQYMYVPPGAPKPTSRDSFAWQTATNPSVFVKMTDPPAQVSVPFMSPASAYQWFYDGYPTFGEHL..QAND
QWF08781.1    ...ELVPQLLQYMYVPPGAPKPTSRDSFAWQTATNPSVFVKMTDPPAQVSVPFMSPASAYQWFYDGYPTFGEHL..QAND
QWY15817.1    ...ELVPQLLQYMYVPPGAPKPTSRDSFAWQTATNPSVFVKMTDPPAQVSVPFMSPASAYQWFYDGYPTFGEHP..QAND
QWF08897.1    ...ELVPQLLQYMYVPPGAPKPTSRDSFAWQTATNPSVFVKMTDPPAQVSVPFMSPASAYQWFYDGYPTFGEHP..QAND
QWY15815.1    ...ELVPQLLQYMYVPPGAPKPTSRDSFAWQTATNPSVFVKMTDPPAQVSVPFMSPASAYQWFYDGYPTFGEHP..QAND
QWY15814.1    ...ELVPQLLQYMYVPPGAPKPTSRDSFAWQTATNPSVFVKMTDPPAQVSVPFMSPASAYQWFYDGYPTFGEHP..QAND
QWY15718.1    ...ELVPQLLQYMYVPPGAPKPTSRDSFAWQTATNPSVFVKMTDPPAQVSVPFMSPASAYQWFYDGYPTFGEHP..QAND
QYC65077.1    ...ELVPQLLQYMYVPPGAPKPTSRDSFAWQTATNPSVFVKMTDPPAQVSVPFMSPASAYQWFYDGYPTFGEHR..QAND
QTA73897.1    ...ELVPQLLQYMYVPPGAPKPTSRDSFAWQTATNPSVFVKMTDPPAQVSVPFMSPASAYQWFYDGYPTFGEHL..QAND
QWY15676.1    ...ELVPQLLQYMYVPPGAPKPTSRDSFAWQTATNPSVFVKMTDPPAQVSVPFMSPASAYQWFYDGYPTFGEHL..QAND
QOP74939.1    ...ELVPQLLQYMYVPPGAPKPTSRDSFAWQTATNPSVFVKMTDPPAQVSVPFMSPASAYQWFYDGYPTFGEHL..QAND
QOP74936.1    ...ELVPQLLQYMYVPPGAPKPTSRDSFAWQTATNPSVFVKMTDPPAQVSVPFMSPASAYQWFYDGYPTFGEHL..QAND
QWF08729.1    ...ELVPQLLQYMYVPPGAPKPTSRDSFAWQTATNPSVFVKMTDPPAQVSVPFMSPASAYQWFYDGYPTFGEHL..QAND
QYC65064.1    ...ELVPQLLQYMYVPPGAPKPTSRDSFAWQTATNPSVFVKMTDPPAQVSVPFMSPASAYQWFYDGYPTFGEHL..QAND
QWF08775.1    ...ELVPQLLQYMYVPPGAPKPTSRDSFAWQTATNPSVFVKMTDPPAQVSVPFMSPASAYQWFYDGYPTFGEHL..QAND
QWF08774.1    ...ELVPQLLQYMYVPPGAPKPTSRDSFAWQTATNPSVFVKMTDPPAQVSVPFMSPASAYQWFYDGYPTFGEHL..QAND
QWF08753.1    ...ELVPQLLQYMYVPPGAPKPTSRDSFAWQTATNPSVFVKMTDPPAQVSVPFMSPASAYQWFYDGYPTFGEHL..QAND
QWY15804.1    ...ELVPQLLQYMYVPPGAPKPTSRDSFAWQTATNPSVFVKMTDPPAQVSVPFMSPASAYQWFYDGYPTFGEHL..QAND
QOP74943.1    ...ELVPQLLQYMYVPPGAPKPTSRDSFAWQTATNPSVFVKMTDPPAQVSVPFMSPASAYQWFYDGYPTFGEHL..QAND
QIH53920.1    ...ELVPQLLQYMYVPPGAPKPTSRDSFAWQTATNPSVFVKMTDPPAQVSVPFMSPASAYQWFYDGYPTFGEHL..QAND
QIH53919.1    ...ELVPQLLQYMYVPPGAPKPTSRDSFAWQTATNPSVFVKMTDPPAQVSVPFMSPASAYQWFYDGYPTFGEHL..QAND
QIL87536.1    ...ELVPQLLQYMYVPPGAPKPTSRDSFAWQTATNPSVFVKMTDPPAQVSVPFMSPASAYQWFYDGYPTFGEHL..QAND
UEC49854.1    ...ELVPQLLQYMYVPPGAPKPTSRDSFAWQTATNPSVFVKMTDPPAQVSVPFMSPASAYQWFYDGYPTFGEHL..QAND
UEC49852.1    ...ELVPQLLQYMYVPPGAPKPTSRDSFAWQTATNPSVFVKMTDPPAQVSVPFMSPASAYQWFYDGYPTFGEHL..QAND
QWY15841.1    ...ELVPQLLQYMYVPPGAPKPTSRDSFAWQTATNPSVFVKMTDPPAQVSVPFMSPASAYQWFYDGYPTFGEHL..QAND
QWY15787.1    ...ELVPQLLQYMYVPPGAPKPTSRDSFAWQTATNPSVFVKMTDPPAQVSVPFMSPASAYQWFYDGYPTFGEHL..QAND
QYC65128.1    ...ELVPQLLQYMYVPPGAPKPTSRDSFAWQTATNPSVFVKMTDPPAQVSVPFMSPASAYQWFYDGYPTFGEHL..QAND
QYC65116.1    ...ELVPQLLQYMYVPPGAPKPTSRDSFAWQTATNPSVFVKMTDPPAQVSVPFMSPASAYQWFYDGYPTFGEHL..QAND
QYC65083.1    ...ELVPQLLQYMYVPPGAPKPTSRDSFAWQTATNPSVFVKMTDPPAQVSVPFMSPASAYQWFYDGYPTFGEHL..QAND
QYC65029.1    ...ELVPQLLQYMYVPPGAPKPTSRDSFAWQTATNPSVFVKMTDPPAQVSVPFMSPASAYQWFYDGYPTFGEHL..QAND
QWF08951.1    ...ELVPQLLQYMYVPPGAPKPTSRDSFAWQTATNPSVFVKMTDPPAQVSVPFMSPASAYQWFYDGYPTFGEHL..QAND
QWF08937.1    ...ELVPQLLQYMYVPPGAPKPTSRDSFAWQTATNPSVFVKMTDPPAQVSVPFMSPASAYQWFYDGYPTFGEHL..QAND
QWF08918.1    ...ELVPQLLQYMYVPPGAPKPTSRDSFAWQTATNPSVFVKMTDPPAQVSVPFMSPASAYQWFYDGYPTFGEHL..QAND
QWF08901.1    ...ELVPQLLQYMYVPPGAPKPTSRDSFAWQTATNPSVFVKMTDPPAQVSVPFMSPASAYQWFYDGYPTFGEHL..QAND
QWF08834.1    ...ELVPQLLQYMYVPPGAPKPTSRDSFAWQTATNPSVFVKMTDPPAQVSVPFMSPASAYQWFYDGYPTFGEHL..QAND
QWF08832.1    ...ELVPQLLQYMYVPPGAPKPTSRDSFAWQTATNPSVFVKMTDPPAQVSVPFMSPASAYQWFYDGYPTFGEHL..QAND
QWF08802.1    ...ELVPQLLQYMYVPPGAPKPTSRDSFAWQTATNPSVFVKMTDPPAQVSVPFMSPASAYQWFYDGYPTFGEHL..QAND
QWF08800.1    ...ELVPQLLQYMYVPPGAPKPTSRDSFAWQTATNPSVFVKMTDPPAQVSVPFMSPASAYQWFYDGYPTFGEHL..QAND
QYC65123.1    ...ELVPQLLQYMYVPPGAPKPTSRDSFAWQTATNPSVFVKMTDPPAQVSVPFMSPASAYQWFYDGYPTFGEHL..QAND
QWY15820.1    ...ELVPQLLQYMYVPPGAPKPTSRDSFAWQTATNPSVFVKMTDPPAQVSVPFMSPASAYQWFYDGYPTFGEHL..QAND
QWY15864.1    ...GLVPQLLQYMYVPPGAPKPTSRDSFAWQTATNPSVFVKMTDPPAQVSVPFMSPASAYQWFYDGYPTFGEHL..QAND
QWY15840.1    ...GLVPQLLQYMYVPPGAPKPTSRDSFAWQTATNPSVFVKMTDPPAQVSVPFMSPASAYQWFYDGYPTFGEHL..QAND
QWY15786.1    ...GLVPQLLQYMYVPPGAPKPTSRDSFAWQTATNPSVFVKMTDPPAQVSVPFMSPASAYQWFYDGYPTFGEHL..QAND
QWY15842.1    ...VLVPQLLQYMYVPPGAPKPTSRDSFAWQTATNPSVFVKMTDPPAQVSVPFMSPASAYQWFYDGYPTFGEHL..QAND
QWY15704.1    ...VLVPQLLQYMYVPPGAPKPTSRDSFAWQTATNPSVFVKMTDPPAQVSVPFMSPASAYQWFYDGYPTFGEHL..QAND
QWY15750.1    ...ELVPQLLQYMYVPPGAPKPTSRDSFAWQTATNPSVFVKMTDPPAQVSVPFMSPASAYQWFYDGYPTFGEHL..QAND
QWF08733.1    ...ELVPQLLQYMYVPPGAPKPTSRDSFAWQTATNPSVFVKMTDPPAQVSVPFMSPASAYQWFYDGYPTFGEHL..QAND
QNL13228.1    ...ELVPQLLQYMYVPPGAPKPTSRDSFAWQTATNPSVFVKMTDPPAQVSVPFMSPASAYQWFYDGYPTFGEHL..QAND
QNL13216.1    ...ELVPQLLQYMYVPPGAPKPTSRDSFAWQTATNPSVFVKMTDPPAQVSVPFMSPASAYQWFYDGYPTFGEHL..QAND
QNL13226.1    ...ELVPQLLQYMYVPPGAPKPTSRDSFAWQTATNPSVFVKMTDPPAQVSVPFMSPASAYQWFYDGYPTFGEHL..QAND
QNL13203.1    ...ELVPQLLQYMYVPPGAPKPTSRDSFAWQTATNPSVFVKMTDPPAQVSVPFMSPASAYQWFYDGYPTFGEHL..QAND
```

```
QYC65080.1    ...ELVPQLLQYMYVPPGAPKPTSRDSFAWQTATNPSVFVKMTDPPAQVSVPFMSPASAYQWFYDGYPTFGEHL..QAND
QNL13225.1    ...ELVPQLLQYMYVPPGAPKPTSRDSFAWQTATNPSVFVKMTDPPAQVSVPFMSPASAYQWFYDGYPTFGEHL..QAND
QNL13224.1    ...ELVPQLLQYMYVPPGAPKPTSRDSFAWQTATNPSVFVKMTDPPAQVSVPFMSPASAYQWFYDGYPTFGEHL..QAND
QNL13223.1    ...ELVPQLLQYMYVPPGAPKPTSRDSFAWQTATNPSVFVKMTDPPAQVSVPFMSPASAYQWFYDGYPTFGEHL..QAND
QNL13222.1    ...ELVPQLLQYMYVPPGAPKPTSRDSFAWQTATNPSVFVKMTDPPAQVSVPFMSPASAYQWFYDGYPTFGEHL..QAND
QNL13221.1    ...ELVPQLLQYMYVPPGAPKPTSRDSFAWQTATNPSVFVKMTDPPAQVSVPFMSPASAYQWFYDGYPTFGEHL..QAND
QNL13220.1    ...ELVPQLLQYMYVPPGAPKPTSRDSFAWQTATNPSVFVKMTDPPAQVSVPFMSPASAYQWFYDGYPTFGEHL..QAND
QNL13219.1    ...ELVPQLLQYMYVPPGAPKPTSRDSFAWQTATNPSVFVKMTDPPAQVSVPFMSPASAYQWFYDGYPTFGEHL..QAND
QWF08894.1    ...ELVPQLLQYMYVPPGAPKPTSRDSFAWQTATNPSVFVKMTDPPAQVSVPFMSPASAYQWFYDGYPTFGEHL..QAND
QNL13218.1    ...ELVPQLLQYMYVPPGAPKPTSRDSFAWQTATNPSVFVKMTDPPAQVSVPFMSPASAYQWFYDGYPTFGEHL..QAND
QNL13217.1    ...ELVPQLLQYMYVPPGAPKPTSRDSFAWQTATNPSVFVKMTDPPAQVSVPFMSPASAYQWFYDGYPTFGEHL..QAND
QNL13215.1    ...ELVPQLLQYMYVPPGAPKPTSRDSFAWQTATNPSVFVKMTDPPAQVSVPFMSPASAYQWFYDGYPTFGEHL..QAND
QNL13214.1    ...ELVPQLLQYMYVPPGAPKPTSRDSFAWQTATNPSVFVKMTDPPAQVSVPFMSPASAYQWFYDGYPTFGEHL..QAND
QNL13213.1    ...ELVPQLLQYMYVPPGAPKPTSRDSFAWQTATNPSVFVKMTDPPAQVSVPFMSPASAYQWFYDGYPTFGEHL..QAND
QNL13212.1    ...ELVPQLLQYMYVPPGAPKPTSRDSFAWQTATNPSVFVKMTDPPAQVSVPFMSPASAYQWFYDGYPTFGEHL..QAND
QNL13209.1    ...ELVPQLLQYMYVPPGAPKPTSRDSFAWQTATNPSVFVKMTDPPAQVSVPFMSPASAYQWFYDGYPTFGEHL..QAND
QIL87537.1    ...ELVPQLLQYMYVPPGAPKPTSRDSFAWQTATNPSVFVKMTDPPAQVSVPFMSPASAYQWFYDGYPTFGEHL..QAND
QNL13208.1    ...ELVPQLLQYMYVPPGAPKPTSRDSFAWQTATNPSVFVKMTDPPAQVSVPFMSPASAYQWFYDGYPTFGEHL..QAND
QNL13207.1    ...ELVPQLLQYMYVPPGAPKPTSRDSFAWQTATNPSVFVKMTDPPAQVSVPFMSPASAYQWFYDGYPTFGEHL..QAND
QNL13206.1    ...ELVPQLLQYMYVPPGAPKPTSRDSFAWQTATNPSVFVKMTDPPAQVSVPFMSPASAYQWFYDGYPTFGEHL..QAND
QNL13202.1    ...ELVPQLLQYMYVPPGAPKPTSRDSFAWQTATNPSVFVKMTDPPAQVSVPFMSPASAYQWFYDGYPTFGEHL..QAND
QNL13196.1    ...ELVPQLLQYMYVPPGAPKPTSRDSFAWQTATNPSVFVKMTDPPAQVSVPFMSPASAYQWFYDGYPTFGEHL..QAND
QNL13195.1    ...ELVPQLLQYMYVPPGAPKPTSRDSFAWQTATNPSVFVKMTDPPAQVSVPFMSPASAYQWFYDGYPTFGEHL..QAND
QNL13194.1    ...ELVPQLLQYMYVPPGAPKPTSRDSFAWQTATNPSVFVKMTDPPAQVSVPFMSPASAYQWFYDGYPTFGEHL..QAND
QNL13193.1    ...ELVPQLLQYMYVPPGAPKPTSRDSFAWQTATNPSVFVKMTDPPAQVSVPFMSPASAYQWFYDGYPTFGEHL..QAND
QNL13192.1    ...ELVPQLLQYMYVPPGAPKPTSRDSFAWQTATNPSVFVKMTDPPAQVSVPFMSPASAYQWFYDGYPTFGEHL..QAND
QNL13191.1    ...ELVPQLLQYMYVPPGAPKPTSRDSFAWQTATNPSVFVKMTDPPAQVSVPFMSPASAYQWFYDGYPTFGEHL..QAND
QNL13190.1    ...ELVPQLLQYMYVPPGAPKPTSRDSFAWQTATNPSVFVKMTDPPAQVSVPFMSPASAYQWFYDGYPTFGEHL..QAND
QNL13189.1    ...ELVPQLLQYMYVPPGAPKPTSRDSFAWQTATNPSVFVKMTDPPAQVSVPFMSPASAYQWFYDGYPTFGEHL..QAND
QNL13188.1    ...ELVPQLLQYMYVPPGAPKPTSRDSFAWQTATNPSVFVKMTDPPAQVSVPFMSPASAYQWFYDGYPTFGEHL..QAND
QCY53846.1    ...ELVPQLLQYMYVPPGAPKPTSRDSFAWQTATNPSVFVKMTDPPAQVSVPFMSPASAYQWFYDGYPTFGEHL..QAND
BCI65357.1    ...ELVPQLLQYMYVPPGAPKPTSRDSFAWQTATNPSVFVKMTDPPAQVSVPFMSPASAYQWFYDGYPTFGEHL..QAND
QNL13227.1    ...ELVPQLLQYMYVPPGAPKPTSRDSFAWQTATNPSVFVKMTDPPAQVSVPFMSPASAYQWFYDGYPTFGEHL..QAND
QNL13211.1    ...ELVPQLLQYMYVPPGAPKPTSRDSFAWQTATNPSVFVKMTDPPAQVSVPFMSPASAYQWFYDGYPTFGEHL..QAND
QNL13198.1    ...ELVPQLLQYMYVPPGAPKPTSRDSFAWQTATNPSVFVKMTDPPAQVSVPFMSPASAYQWFYDGYPTFGEHL..QAND
QNL13197.1    ...ELVPQLLQYMYVPPGAPKPTSRDSFAWQTATNPSVFVKMTDPPAQVSVPFMSPASAYQWFYDGYPTFGEHL..QAND
QNL13200.1    ...ELVPQLLQYMYVPPGAPKPTSRDSFAWQTATNPSVFVKMTDPPAQVSVPFMSPASAYQWFYDGYPTFGEHL..QAND
QNL13210.1    ...ELVPQLLQYMYVPPGAPKPTSRDSFAWQTATNPSVFVKMTDPPAQVSVPFMSPASAYQWFYDGYPTFGEHL..QAND
QNL13205.1    ...ELVPQLLQYMYVPPGAPKPTSRDSFAWQTATNPSVFVKMTDPPAQVSVPFMSPASAYQWFYDGYPTFGEHL..QAND
QNL13204.1    ...ELVPQLLQYMYVPPGAPKPTSRDSFAWQTATNPSVFVKMTDPPAQVSVPFMSPASAYQWFYDGYPTFGEHL..QAND
QNL13201.1    ...ELVPQLLQYMYVPPGAPKPTSRDSFAWQTATNPSVFVKMTDPPAQVSVPFMSPASAYQWFYDGYPTFGEHL..QAND
QNL13199.1    ...ELVPQLLQYMYVPPGAPKPTSRDSFAWQTATNPSVFVKMTDPPAQVSVPFMSPASAYQWFYDGYPTFGEHL..QAND
QYC65157.1    ...ELVPQLLQYMYVPPGAPKPTSRDSFAWQTATNPSVFVKMTDPPAQVSVPFMSPASAYQWFYDGYPTFGEHL..QAND
QYC65138.1    ...ELVPQLLQYMYVPPGAPKPTSRDSFAWQTATNPSVFVKMTDPPAQVSVPFMSPASAYQWFYDGYPTFGEHL..QAND
QWF08724.1    ...ELVPQLLQYMYVPPGAPKPTSRDSFAWQTATNPSVFVKMTDPPAQVSVPFMSPASAYQWFYDGYPTFGEHL..QAND
UEP53403.1    ...ELVPQLLQYMYVPPGAPKPTSRDSFAWQTATNPSVFVKMTDPPAQVSVPFMSPASAYQWFYDGYPTFGEHL..QAND
QWY15843.1    ...ELVPQLLQYMYVPPGAPKPTSRDSFAWQTATNPSVFVKMTDPPAQVSVPFMSPASAYQWFYDGYPTFGEHL..QAND
QWF08794.1    ...ELVPQLLQYMYVPPGAPKPTSRDSFAWQTATNPSVFVKMTDPPAQVSVPFMSPASAYQWFYDGYPTFGEHL..QAND
QYC65031.1    ...ELVPQLLQYMYVPPGAPKPTSRDSFAWQTATNPSVFVKMTDPPAQVSVPFMSPASAYQWFYDGYPTFGEHL..QAND
UEC49856.1    ...ELVPQLLQYMYVPPGAPKPTSRDSFAWQTATNPSVFVKMTDPPAQVSVPFMSPASAYQWFYDGYPTFGEHL..QAND
BBH72184.1    ...ELVPQLLQYMYVPPGAPKPTSRDSFAWQTATNPSVFVKMTDPPAQVSVPFMSPASAYQWFYDGYPTFGEHL..QAND
QWF08874.1    ...ELVPQLLQYMYVPPGAPKPTSRDSFAWQTATNPSVFVKMTDPPAQVSVPFMSPASAYQWFYDGYPTFGEHL..QAND
QWF08873.1    ...ELVPQLLQYMYVPPGAPKPTSRDSFAWQTATNPSVFVKMTDPPAQVSVPFMSPASAYQWFYDGYPTFGEHL..QAND
BCG67003.1    ...ELVPQLLQYMYVPPGAPKPTSRDSFAWQTATNPSVFVKMTDPPAQVSVPFMSPASAYQWFYDGYPTFGEHL..QAND
QYC65095.1    ...ELVPQLLQYMYVPPGAPKPTSRDSFAWQTATNPSVFVKMTDPPAQVSVPFMSPASAYQWFYDGYPTFGEHL..QAND
QOP74933.1    ...ELVPQLLQYMYVPPGAPKPTSRDSFAWQTATNPSVFVKMTDPPAQVSVPFMSPASAYQWFYDGYPTFGEHL..QAND
QYC65096.1    ...ELVPQLLQYMYVPPGAPKPTSRDSFAWQTATNPSVFVKMTDPPAQVSVPFMSPASAYQWFYDGYPTFGEHL..QAND
BCG67034.1    ...ELVPQLLQYMYVPPGAPKPTSRDSFAWQTATNPSVFVKMTDPPAQVSVPFMSPASAYQWFYDGYPTFGEHL..QAND
BCG67031.1    ...ELVPQLLQYMYVPPGAPKPTSRDSFAWQTATNPSVFVKMTDPPAQVSVPFMSPASAYQWFYDGYPTFGEHL..QAND
QYC65052.1    ...ELVPQLLQYMYVPPGAPKPTSRDSFAWQTATNPSVFVKMTDPPAQVSVPFMSPASAYQWFYDGYPTFGEHL..QAND
QWF08963.1    ...ELVPQLLQYMYVPPGAPKPTSRDSFAWQTATNPSVFVKMTDPPAQVSVPFMSPASAYQWFYDGYPTFGEHL..QAND
QWF08939.1    ...ELVPQLLQYMYVPPGAPKPTSRDSFAWQTATNPSVFVKMTDPPAQVSVPFMSPASAYQWFYDGYPTFGEHL..QAND
QIL87538.1    ...ELVPQLLQYMYVPPGAPKPTSRDSFAWQTATNPSVFVKMTDPPAQVSVPFMSPASAYQWFYDGYPTFGEHL..QAND
QIL87535.1    ...ELVPQLLQYMYVPPGAPKPTSRDSFAWQTATNPSVFVKMTDPPAQVSVPFMSPASAYQWFYDGYPTFGEHL..QAND
QWF08964.1    ...ELVPQLLQYMYVPPGAPKPTSRDSFAWQTATNPSVFVKMTDPPAQVSVPFMSPASAYQWFYDGYPTFGEHL..QAND
QYC65132.1    ...ELVPQLLQYMYVPPGAPKPTSRDSFAWQTATNPSVFVKMTDPPAQVSVPFMSPASAYQWFYDGYPTFGEHL..QAND
QTA73893.1    ...VLVPQLLQYMYVPPGAPKPTSRDSFAWQTATNPSVFVKMTDPPAQVSVPFMSPASAYQWFYDGYPTFGEHL..QAND
QWF08751.1    ...VLVPQLLQYMYVPPGAPKPTSRDSFAWQTATNPSVFVKMTDPPAQVSVPFMSPASAYQWFYDGYPTFGEHL..QAND
QWY15860.1    ...VLVPQLLQYMYVPPGAPKPTSRDSFAWQTATNPSVFVKMTDPPAQVSVPFMSPASAYQWFYDGYPTFGEHL..QAND
QWY15749.1    ...VLVPQLLQYMYVPPGAPKPTSRDSFAWQTATNPSVFVKMTDPPAQVSVPFMSPASAYQWFYDGYPTFGEHL..QAND
QWY15744.1    ...VLVPQLLQYMYVPPGAPKPTSRDSFAWQTATNPSVFVKMTDPPAQVSVPFMSPASAYQWFYDGYPTFGEHL..QAND
QWY15740.1    ...VLVPQLLQYMYVPPGAPKPTSRDSFAWQTATNPSVFVKMTDPPAQVSVPFMSPASAYQWFYDGYPTFGEHL..QAND
QWY15700.1    ...VLVPQLLQYMYVPPGAPKPTSRDSFAWQTATNPSVFVKMTDPPAQVSVPFMSPASAYQWFYDGYPTFGEHL..QAND
QWY15698.1    ...VLVPQLLQYMYVPPGAPKPTSRDSFAWQTATNPSVFVKMTDPPAQVSVPFMSPASAYQWFYDGYPTFGEHL..QAND
QWY15672.1    ...VLVPQLLQYMYVPPGAPKPTSRDSFAWQTATNPSVFVKMTDPPAQVSVPFMSPASAYQWFYDGYPTFGEHL..QAND
pdb|6LHT|B    ...VLVPQLLQYMYVPPGAPKPTSRDSFAWQTATNPSVFVKMTDPPAQVSVPFMSPASAYQWFYDGYPTFGEHL..QAND
pdb|6LHT|E    ...VLVPQLLQYMYVPPGAPKPTSRDSFAWQTATNPSVFVKMTDPPAQVSVPFMSPASAYQWFYDGYPTFGEHL..QAND
pdb|6LHT|D    ...VLVPQLLQYMYVPPGAPKPTSRDSFAWQTATNPSVFVKMTDPPAQVSVPFMSPASAYQWFYDGYPTFGEHL..QAND
pdb|6LHT|C    ...VLVPQLLQYMYVPPGAPKPTSRDSFAWQTATNPSVFVKMTDPPAQVSVPFMSPASAYQWFYDGYPTFGEHL..QAND
pdb|6LHT|A    ...VLVPQLLQYMYVPPGAPKPTSRDSFAWQTATNPSVFVKMTDPPAQVSVPFMSPASAYQWFYDGYPTFGEHL..QAND
pdb|6LHQ|A    ...VLVPQLLQYMYVPPGAPKPTSRDSFAWQTATNPSVFVKMTDPPAQVSVPFMSPASAYQWFYDGYPTFGEHL..QAND
pdb|6LHP|A    ...VLVPQLLQYMYVPPGAPKPTSRDSFAWQTATNPSVFVKMTDPPAQVSVPFMSPASAYQWFYDGYPTFGEHL..QAND
pdb|6LHO|A    ...VLVPQLLQYMYVPPGAPKPTSRDSFAWQTATNPSVFVKMTDPPAQVSVPFMSPASAYQWFYDGYPTFGEHL..QAND
pdb|6LHL|A    ...VLVPQLLQYMYVPPGAPKPTSRDSFAWQTATNPSVFVKMTDPPAQVSVPFMSPASAYQWFYDGYPTFGEHL..QAND
pdb|6LHK|A    ...VLVPQLLQYMYVPPGAPKPTSRDSFAWQTATNPSVFVKMTDPPAQVSVPFMSPASAYQWFYDGYPTFGEHL..QAND
pdb|6LHC|A    ...VLVPQLLQYMYVPPGAPKPTSRDSFAWQTATNPSVFVKMTDPPAQVSVPFMSPASAYQWFYDGYPTFGEHL..QAND
pdb|6LHB|A    ...VLVPQLLQYMYVPPGAPKPTSRDSFAWQTATNPSVFVKMTDPPAQVSVPFMSPASAYQWFYDGYPTFGEHL..QAND
pdb|6LHA|A    ...VLVPQLLQYMYVPPGAPKPTSRDSFAWQTATNPSVFVKMTDPPAQVSVPFMSPASAYQWFYDGYPTFGEHL..QAND
QWY15708.1    ...VLVPQLLQYMYVPPGAPKPTSRDSFAWQTATNPSVFVKMTDPPAQVSVPFMSPASAYQWFYDGYPTFGEHL..QAND
BBH72101.1    ...ELVPQLLQYMYVPPGAPKPTSRDSFAWQTATNPSVFVKMTDPPAQVSVPFMSPASAYQWFYDGYPTFGEHL..QAND
BBH72090.1    ...ELVPQLLQYMYVPPGAPKPTSRDSFAWQTATNPSVFVKMTDPPAQVSVPFMSPASAYQWFYDGYPTFGEHL..QAND
BBH72097.1    ...ELVPQLLQYMYVPPGAPKPTSRDSFAWQTATNPSVFVKMTDPPAQVSVPFMSPASAYQWFYDGYPTFGEHL..QADD
BBH72089.1    ...ELVPQLLQYMYVPPGAPKPTSRDSFAWQTATNPSVFVKMTDPPAQVSVPFMSPASAYQWFYDGYPTFGEHL..QAND
BBH72099.1    ...ELVPQLLQYMYVPPGAPKPTSRDSFAWQTATNPSVFVKMTDPPAQVSVPFMSPASAYQWFYDGYPTFGEHL..QAND
BBH72093.1    ...ELVPQLLQYMYVPPGAPKPTSRDSFAWQTATNPSVFVKMTDPPAQVSVPFMSPASAYQWFYDGYPTFGEHL..QAND
BBH72096.1    ...ELVPQLLQYMYVPPGAPKPTSRDSFAWQTATNPSVFVKMTDPPAQVSVPFMSPASAYQWFYDGYPTFGEHL..QAND
BBH72094.1    ...VLVPQLLQYMYVPPGAPKPTSRDSFAWQTATNPSVFVKMTDPPAQVSVPFMSPASAYQWFYDGYPTFGEHL..QAND
BBH72091.1    ...VLVPQLLQYMYVPPGAPKPTSRDSFAWQTATNPSVFVKMTDPPAQVSVPFMSPASAYQWFYDGYPTFGEHL..QAND
QOP74916.1    ...ELVPQLLQYMYVPPGAPKPTSRDSFAWQTATNPSVFVKMTDPPAQVSVPFMSPASAYQWFYDGYPTFGEHL..QAND
QYC65163.1    ...ELVPQLLQYMYVPPGAPKPTSRDSFAWQTATNPSVFVKMTDPPAQVSVPFMSPASAYQWFYDGYPTFGEHL..QAND
QWF08736.1    ...ELVPQLLQYMYVPPGAPKPTSRDSFAWQTATNPSVFVKMTDPPAQVSVPFMSPASAYQWFYDGYPTFGEHL..QAND
QWF08735.1    ...ELVPQLLQYMYVPPGAPKPTSRDSFAWQTATNPSVFVKMTDPPAQVSVPFMSPASAYQWFYDGYPTFGEHL..QAND
pdb|5TSK|A    ...ELVPQLLQYMYVPPGAPKPTSRDSFAWQTATNPSVFVKMTDPPAQVSVPFMSPASAYQWFYDGYPTFGEHL..QAND
pdb|5C9A|A    ...ELVPQLLQYMYVPPGAPKPTSRDSFAWQTATNPSVFVKMTDPPAQVSVPFMSPASAYQWFYDGYPTFGEHL..QAND
pdb|5C4W|A    ...ELVPQLLQYMYVPPGAPKPTSRDSFAWQTATNPSVFVKMTDPPAQVSVPFMSPASAYQWFYDGYPTFGEHL..QAND
pdb|4JGZ|A    ...ELVPQLLQYMYVPPGAPKPTSRDSFAWQTATNPSVFVKMTDPPAQVSVPFMSPASAYQWFYDGYPTFGEHL..QAND
pdb|4JGY|A    ...ELVPQLLQYMYVPPGAPKPTSRDSFAWQTATNPSVFVKMTDPPAQVSVPFMSPASAYQWFYDGYPTFGEHL..QAND
QWY15956.1    ...ELVPQLLQYMYVPPGAPKPTSRDSFAWQTATNPSVFVKMTDPPAQVSVPFMSPASAYQWFYDGYPTFGEHL..QAND
QWF08811.1    ...ELVPQLLQYMYVPPGAPKPTSRDSFAWQTATNPSVFVKMTDPPAQVSVPFMSPASAYQWFYDGYPTFGEHL..QAND
QWF08878.1    ...ELVPQLLQYMYVPPGAPKPTSRDSFAWQTATNPSVFVKMTDPPAQVSVPFMSPASAYQWFYDGYPTFGEHL..QAND
QWF08869.1    ...ELVPQLLQYMYVPPGAPKPTSRDSFAWQTATNPSVFVKMTDPPAQVSVPFMSPASAYQWFYDGYPTFGEHL..QAND
QWF08831.1    ...ELVPQLLQYMYVPPGAPKPTSRDSFAWQTATNPSVFVKMTDPPAQVSVPFMSPASAYQWFYDGYPTFGEHL..QAND
QWF08829.1    ...ELVPQLLQYMYVPPGAPKPTSRDSFAWQTATNPSVFVKMTDPPAQVSVPFMSPASAYQWFYDGYPTFGEHL..QAND
QWF08824.1    ...ELVPQLLQYMYVPPGAPKPTSRDSFAWQTATNPSVFVKMTDPPAQVSVPFMSPASAYQWFYDGYPTFGEHL..QAND
QWF08822.1    ...ELVPQLLQYMYVPPGAPKPTSRDSFAWQTATNPSVFVKMTDPPAQVSVPFMSPASAYQWFYDGYPTFGEHL..QAND
QWF08817.1    ...ELVPQLLQYMYVPPGAPKPTSRDSFAWQTATNPSVFVKMTDPPAQVSVPFMSPASAYQWFYDGYPTFGEHL..QAND
QWF08816.1    ...ELVPQLLQYMYVPPGAPKPTSRDSFAWQTATNPSVFVKMTDPPAQVSVPFMSPASAYQWFYDGYPTFGEHL..QAND
QWF08798.1    ...ELVPQLLQYMYVPPGAPKPTSRDSFAWQTATNPSVFVKMTDPPAQVSVPFMSPASAYQWFYDGYPTFGEHL..QAND
QWF08779.1    ...ELVPQLLQYMYVPPGAPKPTSRDSFAWQTATNPSVFVKMTDPPAQVSVPFMSPASAYQWFYDGYPTFGEHL..QAND
QWF08778.1    ...ELVPQLLQYMYVPPGAPKPTSRDSFAWQTATNPSVFVKMTDPPAQVSVPFMSPASAYQWFYDGYPTFGEHL..QAND
QWF08772.1    ...ELVPQLLQYMYVPPGAPKPTSRDSFAWQTATNPSVFVKMTDPPAQVSVPFMSPASAYQWFYDGYPTFGEHL..QAND
QWF08771.1    ...ELVPQLLQYMYVPPGAPKPTSRDSFAWQTATNPSVFVKMTDPPAQVSVPFMSPASAYQWFYDGYPTFGEHL..QAND
QWF08745.1    ...ELVPQLLQYMYVPPGAPKPTSRDSFAWQTATNPSVFVKMTDPPAQVSVPFMSPASAYQWFYDGYPTFGEHL..QAND
QWF08744.1    ...ELVPQLLQYMYVPPGAPKPTSRDSFAWQTATNPSVFVKMTDPPAQVSVPFMSPASAYQWFYDGYPTFGEHL..QAND
QIH53922.1    ...ELVPQLLQYMYVPPGAPKPTSRDSFAWQTATNPSVFVKMTDPPAQVSVPFMSPASAYQWFYDGYPTFGEHL..QAND
QOP74941.1    ...ELVPQLLQYMYVPPGAPKPTSRDSFAWQTATNPSVFVKMTDPPAQVSVPFMSPASAYQWFYDGYPTFGEHL..QAND
QWY15812.1    ...ELVPQLLQYMYVPPGAPKPTSRDSFAWQTATNPSVFVKMTDPPAQVSVPFMSPASAYQWFYDGYPTFGEHL..QAND
QOP74926.1    ...ELVPQLLQYMYVPPGAPKPTSRDSFAWQTATNPSVFVKMTDPPAQVSVPFMSPASAYQWFYDGYPTFGEHL..QAND
QYC65176.1    ...ELVPQLLQYMYVPPGAPKPTSRDSFAWQTATNPSVFVKMTDPPAQVSVPFMSPASAYQWFYDGYPTFGEHL..QAND
QYC65172.1    ...ELVPQLLQYMYVPPGAPKPTSRDSFAWQTATNPSVFVKMTDPPAQVSVPFMSPASAYQWFYDGYPTFGEHL..QAND
QYC65168.1    ...ELVPQLLQYMYVPPGAPKPTSRDSFAWQTATNPSVFVKMTDPPAQVSVPFMSPASAYQWFYDGYPTFGEHL..QAND
QWF08725.1    ...ELVPQLLQYMYVPPGAPKPTSRDSFAWQTATNPSVFVKMTDPPAQVSVPFMSPASAYQWFYDGYPTFGEHL..QAND
QYC65028.1    ...ELVPQLLQYMYVPPGAPKPTSRDSFAWQTATNPSVFVKMTDPPAQVSVPFMSPASAYQWFYDGYPTFGEHL..QAND
QWY15686.1    ...ELVPQLLQYMYVPPGAPKPTSRDSFAWQTATNPSVFVKMTDPPAQVSVPFMSPASAYQWFYDGYPTFGEHL..QAND
QYC65023.1    ...ELVPQLLQYMYVPPGAPKPTSRDSFAWQTATNPSVFVKMTDPPAQVSVPFMSPASAYQWFYDGYPTFGEHL..QAND
QYC65025.1    ...ELVPQLLQYMYVPPGAPKPTSRDSFAWQTATNPSVFVKMTDPPAQVSVPFMSPASAYQWFYDGYPTFGEHL..QAND
QWY15946.1    ...ELVPQLLQYMYVPPGAPKPTSRDSFAWQTATNPSVFVKMTDPPAQVSVPFMSPASAYQWFYDGYPTFGEHL..QAND
BCB92164.1    ...ELVPQLLQYMYVPPGAPKPTSRDSFAWQTATNPSVFVKMTDPPAQVSVPFMSPASAYQWFYDGYPTFGEHL..QAND
QOP74935.1    ...ELVPQLLQYMYVPPGAPKPTSRDSFAWQTATNPSVFVKMTDPPAQVSVPFMSPASAYQWFYDGYPTFGEHL..QAND
QOP74930.1    ...ELVPQLLQYMYVPPGAPKPTSRDSFAWQTATNPSVFVKMTDPPAQVSVPFMSPASAYQWFYDGYPTFGEHL..QAND
QOP74928.1    ...ELVPQLLQYMYVPPGAPKPTSRDSFAWQTATNPSVFVKMTDPPAQVSVPFMSPASAYQWFYDGYPTFGEHL..QAND
QOP74927.1    ...ELVPQLLQYMYVPPGAPKPTSRDSFAWQTATNPSVFVKMTDPPAQVSVPFMSPASAYQWFYDGYPTFGEHL..QAND
QOP74925.1    ...ELVPQLLQYMYVPPGAPKPTSRDSFAWQTATNPSVFVKMTDPPAQVSVPFMSPASAYQWFYDGYPTFGEHL..QAND
QOP74923.1    ...ELVPQLLQYMYVPPGAPKPTSRDSFAWQTATNPSVFVKMTDPPAQVSVPFMSPASAYQWFYDGYPTFGEHL..QAND
```

```
QOP74922.1    ...ELVPQLLQYMYVPPGAPKPTSRDSFAWQTATNPSVFVKMTDPPAQVSVPFMSPASAYQWFYDGYPTFGEHL..QAND
QOP74920.1    ...ELVPQLLQYMYVPPGAPKPTSRDSFAWQTATNPSVFVKMTDPPAQVSVPFMSPASAYQWFYDGYPTFGEHL..QAND
QOP74919.1    ...ELVPQLLQYMYVPPGAPKPTSRDSFAWQTATNPSVFVKMTDPPAQVSVPFMSPASAYQWFYDGYPTFGEHL..QAND
QOP74917.1    ...ELVPQLLQYMYVPPGAPKPTSRDSFAWQTATNPSVFVKMTDPPAQVSVPFMSPASAYQWFYDGYPTFGEHL..QAND
QHB49260.1    ...ELVPQLLQYMYVPPGAPKPTSRDSFAWQTATNPSVFVKMTDPPAQVSVPFMSPASAYQWFYDGYPTFGEHL..QAND
QWF08784.1    ...ELVPQLLQYMYVPPGAPKPTSRDSFAWQTATNPSVFVKMTDPPAQVSVPFMSPASAYQWFYDGYPTFGEHL..QAND
QWF08782.1    ...ELVPQLLQYMYVPPGAPKPTSRDSFAWQTATNPSVFVKMTDPPAQVSVPFMSPASAYQWFYDGYPTFGEHL..QAND
QWF08777.1    ...ELVPQLLQYMYVPPGAPKPTSRDSFAWQTATNPSVFVKMTDPPAQVSVPFMSPASAYQWFYDGYPTFGEHL..QAND
QWF08757.1    ...ELVPQLLQYMYVPPGAPKPTSRDSFAWQTATNPSVFVKMTDPPAQVSVPFMSPASAYQWFYDGYPTFGEHL..QAND
QWF08756.1    ...ELVPQLLQYMYVPPGAPKPTSRDSFAWQTATNPSVFVKMTDPPAQVSVPFMSPASAYQWFYDGYPTFGEHL..QAND
QWF08755.1    ...ELVPQLLQYMYVPPGAPKPTSRDSFAWQTATNPSVFVKMTDPPAQVSVPFMSPASAYQWFYDGYPTFGEHL..QAND
QWF08791.1    ...ELVPQLLQYMYVPPGAPKPTSRDSFAWQTATNPSVFVKMTDPPAQVSVPFMSPXSAYQWFYDGYPTFGEHL..QAND
QWF08786.1    ...ELVPQLLQYMYVPPGAPKPTSRDSFAWQTATNPSVFVKMTDPPAQVSVPFMSPASAYQWFYDGYPTFGEHL..QAND
QYC65186.1    ...ELVPQLLQYMYVPPGAPKPTSRDSFAWQTATNPSVFVKMTDPPAQVSVPFMSPASAYQWFYDGYPTFGEHL..QAND
QWY15722.1    ...ELVPQLLQYMYVPPGAPKPTSRDSFAWQTATNPSVFVKMTDPPAQVSVPFMSPASAYQWFYDGYPTFGEHL..QAND
QYC65182.1    ...ELVPQLLQYMYVPPGAPKPTSRDSFAWQTATNPSVFVKMTDPPAQVSVPFMSPASAYQWFYDGYPTFGEHL..QAND
QWF08754.1    ...ELVPQLLQYMYVPPGAPKPTSRDSFAWQTATNPSVFVKMTDPPAQVSVPFMSPASAYQWFYDGYPTFGEHL..QAND
QWF08748.1    ...ELVPQLLQYMYVPPGAPKPTSRDSFAWQTATNPSVFVKMTDPPAQVSVPFMSPASAYQWFYDGYPTFGEHL..QAND
QYC65181.1    ...ELVPQLLQYMYVPPGAPKPTSRDSFAWQTATNPSVFVKMTDPPAQVSVPFMSPASAYQWFYDGYPTFGEHL..QAND
QWY15719.1    ...ELVPQLLQYMYVPPGAPKPTSRDSFAWQTATNPSVFVKMTDPPAQVSVPFMSPASAYQWFYDGYPTFGEHL..QAND
QYC65180.1    ...ELVPQLLQYMYVPPGAPKPTSRDSFAWQTATNPSVFVKMTDPPAQVSVPFMSPASAYQWFYDGYPTFGEHL..QAND
QWY15693.1    ...ELVPQLLQYMYVPPGAPKPTSRDSFAWQTATNPSVFVKMTDPPAQVSVPFMSPASAYQWFYDGYPTFGEHL..QAND
QYC65179.1    ...ELVPQLLQYMYVPPGAPKPTSRDSFAWQTATNPSVFVKMTDPPAQVSVPFMSPASAYQWFYDGYPTFGEHL..QAND
BCB92156.1    ...ELVPQLLQYMYVPPGAPKPTSRDSFAWQTATNPSVFVKMTDPPAQVSVPFMSPASAYQWFYDGYPTFGEHL..QAND
QYC65178.1    ...ELVPQLLQYMYVPPGAPKPTSRDSFAWQTATNPSVFVKMTDPPAQVSVPFMSPASAYQWFYDGYPTFGEHL..QAND
pdb|5C8C|A    ...ELVPQLLQYMYVPPGAPKPTSRDSFAWQTATNPSVFVKMTDPPAQVSVPFMSPASAYQWFYDGYPTFGEHL..QAND
QWF08864.1    ...ELVPQLLQYMYVPPGAPKPTSRDSFAWQTATNPSVFVKMTDPPAQVSVPFMSPASAYQWFYDGYPTFGEHL..QAND
QWF08747.1    ...ELVPQLLQYMYVPPGAPKPTSRDSFAWQTATNPSVFVKMTDPPAQVSVPFMSPASAYQWFYDGYPTFGEHL..QAND
QWF08743.1    ...ELVPQLLQYMYVPPGAPKPTSRDSFAWQTATNPSVFVKMTDPPAQVSVPFMSPASAYQWFYDGYPTFGEHL..QAND
QWF08741.1    ...ELVPQLLQYMYVPPGAPKPTSRDSFAWQTATNPSVFVKMTDPPAQVSVPFMSPASAYQWFYDGYPTFGEHL..QAND
QWF08737.1    ...ELVPQLLQYMYVPPGAPKPTSRDSFAWQTATNPSVFVKMTDPPAQVSVPFMSPASAYQWFYDGYPTFGEHL..QAND
QWF08734.1    ...ELVPQLLQYMYVPPGAPKPTSRDSFAWQTATNPSVFVKMTDPPAQVSVPFMSPASAYQWFYDGYPTFGEHL..QAND
BBH72119.1    ...ELVPQLLQYMYVPPGAPKPTSRDSFAWQTATNPSVFVKMTDPPAQVSVPFMSPASAYQWFYDGYPTFGEHL..QAND
QYC65177.1    ...ELVPQLLQYMYVPPGAPKPTSRDSFAWQTATNPSVFVKMTDPPAQVSVPFMSPASAYQWFYDGYPTFGEHL..QAND
QWF08732.1    ...ELVPQLLQYMYVPPGAPKPTSRDSFAWQTATNPSVFVKMTDPPAQVSVPFMSPASAYQWFYDGYPTFGEHL..QAND
QWF08730.1    ...ELVPQLLQYMYVPPGAPKPTSRDSFAWQTATNPSVFVKMTDPPAQVSVPFMSPASAYQWFYDGYPTFGEHL..QAND
QYC65175.1    ...ELVPQLLQYMYVPPGAPKPTSRDSFAWQTATNPSVFVKMTDPPAQVSVPFMSPASAYQWFYDGYPTFGEHL..QAND
QWF08727.1    ...ELVPQLLQYMYVPPGAPKPTSRDSFAWQTATNPSVFVKMTDPPAQVSVPFMSPASAYQWFYDGYPTFGEHL..QAND
QYC65173.1    ...ELVPQLLQYMYVPPGAPKPTSRDSFAWQTATNPSVFVKMTDPPAQVSVPFMSPASAYQWFYDGYPTFGEHL..QAND
BBH72105.1    ...ELVPQLLQYMYVPPGAPKPTSRDSFAWQTATNPSVFVKMTDPPAQVSVPFMSPASAYQWFYDGYPTFGEHL..QAND
QYC65171.1    ...ELVPQLLQYMYVPPGAPKPTSRDSFAWQTATNPSVFVKMTDPPAQVSVPFMSPASAYQWFYDGYPTFGEHL..QAND
pdb|5ABJ|A    ...ELVPQLLQYMYVPPGAPKPTSRDSFAWQTATNPSVFVKMTDPPAQVSVPFMSPASAYQWFYDGYPTFGEHL..QAND
QWY15716.1    ...ELVPQLLQYMYVPPGAPKPTSRDSFAWQTATNPSVFVKMTDPPAQVSVPFMSPASAYQWFYDGYPTFGEHL..QAND
QWY15800.1    ...ELVPQLLQYMYVPPGAPKPTSRDSFAWQTATNPSVFVKMTDPPAQVSVPFMSPASAYQWFYDGYPTFGEHL..QAND
QYC65018.1    ...ELVPQLLQYMYVPPGAPKPTSRDSFAWQTATNPSVFVKMTDPPAQVSVPFMSPASAYQWFYDGYPTFGEHL..QAND
QWY15712.1    ...ELVPQLLQYMYVPPGAPKPTSRDSFAWQTATNPSVFVKMTDPPAQVSVPFMSPASAYQWFYDGYPTFGEHL..QAND
QWY15711.1    ...ELVPQLLQYMYVPPGAPKPTSRDSFAWQTATNPSVFVKMTDPPAQVSVPFMSPASAYQWFYDGYPTFGEHL..QAND
QWY15707.1    ...ELVPQLLQYMYVPPGAPKPTSRDSFAWQTATNPSVFVKMTDPPAQVSVPFMSPASAYQWFYDGYPTFGEHL..QAND
QWY15688.1    ...ELVPQLLQYMYVPPGAPKPTSRDSFAWQTATNPSVFVKMTDPPAQVSVPFMSPASAYQWFYDGYPTFGEHL..QAND
QWY15685.1    ...ELVPQLLQYMYVPPGAPKPTSRDSFAWQTATNPSVFVKMTDPPAQVSVPFMSPASAYQWFYDGYPTFGEHL..QAND
QWY15684.1    ...ELVPQLLQYMYVPPGAPKPTSRDSFAWQTATNPSVFVKMTDPPAQVSVPFMSPASAYQWFYDGYPTFGEHL..QAND
QYC65170.1    ...ELVPQLLQYMYVPPGAPKPTSRDSFAWQTATNPSVFVKMTDPPAQVSVPFMSPASAYQWFYDGYPTFGEHL..QAND
QYC65169.1    ...ELVPQLLQYMYVPPGAPKPTSRDSFAWQTATNPSVFVKMTDPPAQVSVPFMSPASAYQWFYDGYPTFGEHL..QAND
QYC65162.1    ...ELVPQLLQYMYVPPGAPKPTSRDSFAWQTATNPSVFVKMTDPPAQVSVPFMSPASAYQWFYDGYPTFGEHL..QAND
QYC65099.1    ...ELVPQLLQYMYVPPGAPKPTSRDSFAWQTATNPSVFVKMTDPPAQVSVPFMSPASAYQWFYDGYPTFGEHL..QAND
QYC65048.1    ...ELVPQLLQYMYVPPGAPKPTSRDSFAWQTATNPSVFVKMTDPPAQVSVPFMSPASAYQWFYDGYPTFGEHL..QAND
QYC65040.1    ...ELVPQLLQYMYVPPGAPKPTSRDSFAWQTATNPSVFVKMTDPPAQVSVPFMSPASAYQWFYDGYPTFGEHL..QAND
QYC65035.1    ...ELVPQLLQYMYVPPGAPKPTSRDSFAWQTATNPSVFVKMTDPPAQVSVPFMSPASAYQWFYDGYPTFGEHL..QAND
QYC65032.1    ...ELVPQLLQYMYVPPGAPKPTSRDSFAWQTATNPSVFVKMTDPPAQVSVPFMSPASAYQWFYDGYPTFGEHL..QAND
QYC65030.1    ...ELVPQLLQYMYVPPGAPKPTSRDSFAWQTATNPSVFVKMTDPPAQVSVPFMSPASAYQWFYDGYPTFGEHL..QAND
QYC65027.1    ...ELVPQLLQYMYVPPGAPKPTSRDSFAWQTATNPSVFVKMTDPPAQVSVPFMSPASAYQWFYDGYPTFGEHL..QAND
QYC65026.1    ...ELVPQLLQYMYVPPGAPKPTSRDSFAWQTATNPSVFVKMTDPPAQVSVPFMSPASAYQWFYDGYPTFGEHL..QAND
QYC65024.1    ...ELVPQLLQYMYVPPGAPKPTSRDSFAWQTATNPSVFVKMTDPPAQVSVPFMSPASAYQWFYDGYPTFGEHL..QAND
QYC65022.1    ...ELVPQLLQYMYVPPGAPKPTSRDSFAWQTATNPSVFVKMTDPPAQVSVPFMSPASAYQWFYDGYPTFGEHL..QAND
QYC65021.1    ...ELVPQLLQYMYVPPGAPKPTSRDSFAWQTATNPSVFVKMTDPPAQVSVPFMSPASAYQWFYDGYPTFGEHL..QAND
QYC65020.1    ...ELVPQLLQYMYVPPGAPKPTSRDSFAWQTATNPSVFVKMTDPPAQVSVPFMSPASAYQWFYDGYPTFGEHL..QAND
QYC65019.1    ...ELVPQLLQYMYVPPGAPKPTSRDSFAWQTATNPSVFVKMTDPPAQVSVPFMSPASAYQWFYDGYPTFGEHL..QAND
QWF08742.1    ...ELVPQLLQYMYVPPGAPKPTSRDSFAWQTATNPSVFVKMTDPPAQVSVPFMSPASAYQWFYDGYPTFGEHL..QAND
QWF08728.1    ...ELVPQLLQYMYVPPGAPKPTSRDSFAWQTATNPSVFVKMTDPPAQVSVPFMSPASAYQWFYDGYPTFGEHL..QAND
QWF08726.1    ...ELVPQLLQYMYVPPGAPKPTSRDSFAWQTATNPSVFVKMTDPPAQVSVPFVSPASAYQWFYDGYPTFGEHL..QAND
QYC65054.1    ...ELVPQLLQYMYVPPGAPKPTSRDSFAWQTATNPSVFVKMTDPPAQVSVPFMSPASAYQWFYDGYPTFGEHL..QAND
QWF08746.1    ...ELVPQLLQYMYVPPGAPKPTSRDSFAWQTATNPSVFVKMTDPPAQVSVPFMSPASAYQWFYDGYPTFGEHL..QAND
QYC65033.1    ...ELVPQLLQYMYVPPGAPKPTSRDSFAWQTATNPSVFVKMTDPPAQVSVPFMSPASAYQWFYDGYPTFGEHL..QAND
QWY15952.1    ...ELVPQLLQYMYVPPGAPKPTSRDSFAWQTATNPSVFVKMTDPPAQVSVPFMSPASAYQWFYDGYPTFGEHL..QAND
QYC65042.1    ...ELVPQLLQYMYVPPGAPKPTSRDSFAWQTATNPSVFVKMTDPPAQVSVPFMSPASAYQWFYDGYPTFGEHL..QAND
QYC65017.1    ...ELVPQLLQYMYVPPGAPKPTSRDSFAWQTATNPSVFVKMTDPPAQVSVPFMSPASAYQWFYDGYPTFGEHL..QAND
QOP74918.1    ...ELVPQLLQYMYVPPGAPKPTSRDSFAWQTATNPSVFVKMTDPPAQVSVPFMSPASAYQWFYDGYPTFGEHL..QAND
QWF08731.1    ...ELVPQLLQYMYVPPGAPKPTSRDSFAWQTATNPSVFVKMTDPPAQVSVPFMSPASAYQWFYDGYPTFGEHL..QAND
BBH72123.1    ...ELVPQLLQYMYVPPGAPKPTSRDSFAWQTATNPSVFVKMTDPPAQVSVPFMSPASAYQWFYDGYPTFGEHL..QAND
BBH72114.1    ...ELVPQLLQYMYVPPGAPKPTSRDSFAWQTATNPSVFVKMTDPPAQVSVPFMSPASAYQWFYDGYPTFGEHL..QAND
BBH72092.1    ...ELVPQLLQYMYVPPGAPKPTSRDSFAWQTATNPSVFVKMTDPPAQVSVPFMSPASAYQWFYDGYPTFGEHL..QAND
BBH72095.1    ...ELVPQLLQYMYVPPGAPKPTSRDSFAWQTATNPSVFVKMTDPPAQVSVPFMSPASAYQWFYDGYPTFGEHL..QAND
BBH72186.1    ...ELVPQLLQYMYVPPGAPKPKSRDSFAWQTATNPSVFVKMTDPPAQVSVPFMSPASAYQWFYDGYPTFGEHL..QAND
QWY15975.1    ...ELVPQLLQYMYVPPGAPKPKSRDSFAWQTATNPSVFVKMTDPPAQVSVPFMSPASAYQWFYDGYPTFGEHL..QAND
QWY15974.1    ...ELVPQLLQYMYVPPGAPKPKSRDSFAWQTATNPSVFVKMTDPPAQVSVPFMSPASAYQWFYDGYPTFGEHL..QAND
QWY15968.1    ...ELVPQLLQYMYVPPGAPKPKSRDSFAWQTATNPSVFVKMTDPPAQVSVPFMSPASAYQWFYDGYPTFGEHL..QAND
QWY15960.1    ...ELVPQLLQYMYVPPGAPKPKSRDSFAWQTATNPSVFVKMTDPPAQVSVPFMSPASAYQWFYDGYPTFGEHL..QAND
QWY15959.1    ...ELVPQLLQYMYVPPGAPKPKSRDSFAWQTATNPSVFVKMTDPPAQVSVPFMSPASAYQWFYDGYPTFGEHL..QAND
QWY15953.1    ...ELVPQLLQYMYVPPGAPKPKSRDSFAWQTATNPSVFVKMTDPPAQVSVPFMSPASAYQWFYDGYPTFGEHL..QAND
QWY15951.1    ...ELVPQLLQYMYVPPGAPKPKSRDSFAWQTATNPSVFVKMTDPPAQVSVPFMSPASAYQWFYDGYPTFGEHL..QAND
QWY15950.1    ...ELVPQLLQYMYVPPGAPKPKSRDSFAWQTATNPSVFVKMTDPPAQVSVPFMSPASAYQWFYDGYPTFGEHL..QAND
QWY15949.1    ...ELVPQLLQYMYVPPGAPKPKSRDSFAWQTATNPSVFVKMTDPPAQVSVPFMSPASAYQWFYDGYPTFGEHL..QAND
QWY15944.1    ...ELVPQLLQYMYVPPGAPKPKSRDSFAWQTATNPSVFVKMTDPPAQVSVPFMSPASAYQWFYDGYPTFGEHL..QAND
QWY15943.1    ...ELVPQLLQYMYVPPGAPKPKSRDSFAWQTATNPSVFVKMTDPPAQVSVPFMSPASAYQWFYDGYPTFGEHL..QAND
QWY15942.1    ...ELVPQLLQYMYVPPGAPKPKSRDSFAWQTATNPSVFVKMTDPPAQVSVPFMSPASAYQWFYDGYPTFGEHL..QAND
QWY15939.1    ...ELVPQLLQYMYVPPGAPKPKSRDSFAWQTATNPSVFVKMTDPPAQVSVPFMSPASAYQWFYDGYPTFGEHL..QAND
QWY15938.1    ...ELVPQLLQYMYVPPGAPKPKSRDSFAWQTATNPSVFVKMTDPPAQVSVPFMSPASAYQWFYDGYPTFGEHL..QAND
QWY15937.1    ...ELVPQLLQYMYVPPGAPKPKSRDSFAWQTATNPSVFVKMTDPPAQVSVPFMSPASAYQWFYDGYPTFGEHL..QAND
QWY15936.1    ...ELVPQLLQYMYVPPGAPKPKSRDSFAWQTATNPSVFVKMTDPPAQVSVPFMSPASAYQWFYDGYPTFGEHL..QAND
QWY15934.1    ...ELVPQLLQYMYVPPGAPKPKSRDSFAWQTATNPSVFVKMTDPPAQVSVPFMSPASAYQWFYDGYPTFGEHL..QAND
QWY15896.1    ...ELVPQLLQYMYVPPGAPKPKSRDSFAWQTATNPSVFVKMTDPPAQVSVPFMSPASAYQWFYDGYPTFGEHL..QAND
BCU41098.1    ...ELVPQLLQYMYVPPGAPKPKSRDSFAWQTATNPSVFVKMTDPPAQVSVPFMSPASAYQWFYDGYPTFGEHL..QAND
BCR43090.1    ...ELVPQLLQYMYVPPGAPKPKSRDSFAWQTATNPSVFVKMTDPPAQVSVPFMSPASAYQWFYDGYPTFGEHL..QAND
QYC65129.1    ...ELVPQLLQYMYVPPGAPKPKSRDSFAWQTATNPSVFVKMTDPPAQVSVPFMSPASAYQWFYDGYPTFGEHL..QAND
QWF08961.1    ...ELVPQLLQYMYVPPGAPKPKSRDSFAWQTATNPSVFVKMTDPPAQVSVPFMSPASAYQWFYDGYPTFGEHL..QAND
QIL87527.1    ...ELVPQLLQYMYVPPGAPKPKSRDSFAWQTATNPSVFVKMTDPPAQVSVPFMSPASAYQWFYDGYPTFGEHL..QAND
QWY15955.1    ...ELVPQLLQYMYVPPGAPKPKSRDSFAWQTATNPSVFVKMTDPPAQVSVPFMSPASAYQWFYDGYPTFGEHL..QAND
QWY15954.1    ...ELVPQLLQYMYVPPGAPKPKSRDSFAWQTATNPSVFVKMTDPPAQVSVPFMSPASAYQWFYDGYPTFGEHL..QAND
QWY15945.1    ...ELVPQLLQYMYVPPGAPKPKSRDSFAWQTATNPSVFVKMTDPPAQVSVPFMSPASAYQWFYDGYPTFGEHL..QAND
QWY15935.1    ...ELVPQLLQYMYVPPGAPKPKSRDSFAWQTATNPSVFVKMTDPPAQVSVPFMSPASAYQWFYDGYPTFGEHL..QAND
BCB92163.1    ...ELVPQLLQYMYVPPGAPKPKSRDSFAWQTATNPSVFVKMTDPPAQVSVPFMSPASAYQWFYDGYPTFGEHL..QAND
BCB92158.1    ...ELVPQLLQYMYVPPGAPKPKSRDSFAWQTATNPSVFVKMTDPPAQVSVPFMSPASAYQWFYDGYPTFGEHL..QAND
BCB92154.1    ...ELVPQLLQYMYVPPGAPKPKSRDSFAWQTATNPSVFVKMTDPPAQVSVPFMSPASAYQWFYDGYPTFGEHL..QAND
BCB92152.1    ...ELVPQLLQYMYVPPGAPKPKSRDSFAWQTATNPSVFVKMTDPPAQVSVPFMSPASAYQWFYDGYPTFGEHL..QAND
BCB92162.1    ...ELVPQLLQYMYVPPGAPKPKSRDSFAWQTATNPSVFVKMTDPPAQVSVPFMSPASAYQWFYDGYPTFGEHL..QAND
BCB92161.1    ...ELVPQLLQYMYVPPGAPKPKSRDSFAWQTATNPSVFVKMTDPPAQVSVPFMSPASAYQWFYDGYPTFGEHL..QAND
BCB92160.1    ...ELVPQLLQYMYVPPGAPKPKSRDSFAWQTATNPSVFVKMTDPPAQVSVPFMSPASAYQWFYDGYPTFGEHL..QAND
BBH72129.1    ...ELVPQLLQYMYVPPGAPKPKSRDSFAWQTATNPSVFVKMTDPPAQVSVPFMSPASAYQWFYDGYPTFGEHL..QAND
QIL87533.1    ...ELVPQLLQYMYVPPGAPKPKSRDSFAWQTATNPSVFVKMTDPPAQVSVPFMSPASAYQWFYDGYPTFGEHL..QAND
BBH72150.1    ...ELVPQLLQYMYVPPGAPKPKSRDSFAWQTATNPSVFVKMTDPPAQVSVPFMSPASAYQWFYDGYPTFGEHL..QAND
BBH72137.1    ...ELVPQLHQYMYVPPGAPKPKSRDSFAWQTATNPSVFVKMTDPPAQVSVPFMSPASAYQWFYDGYPTFGEHL..QAND
BBH72145.1    ...ELVPQLLQYMYVPPGAPKPKSRDSFAWQTATNPSVFVKMTDPPAQVSVPFMSPASAYQWFYDGYPTFGEHL..QAND
BCB92157.1    ...ELVPQLLQYMYVPPGAPKPKSRDSFAWQTATNPSVFVKMTDPPAQVSVPFMSPASAYQWFYDGYPTFGEHL..QAND
QYC65124.1    ...ELVPQLLQYMYVPPGAPKPKSRDSFAWQTATNPSVFVKMTDPPAQVSVPFMSPASAYQWFYDGYPTFGEHL..QAND
QWY15920.1    ...ELVPQLLQYMYVPPGAPKPKSRDSFAWQTATNPSVFVKMTDPPAQVSVPFMSPASAYQWFYDGYPTFGEHL..QAND
QWY15973.1    ...ELVPQLLQYMYVPPGAPKPKSRDSFAWQTATNPSVFVKMTDPPAQVSVPFMSPASAYQWFYDGYPTFGEHL..QAND
BCU41116.1    ...ELVPQLLQYMYVPPGAPKPKSRDSFAWQTATNPSVFVKMTDPPAQVSVPFMSPASAYQWFYDGYPTFGEHL..QAND
QWF08960.1    ...ELVPQLLQYMYVPPGAPKPKSRDSFAWQTATNPSVFVKMTDPPAQVSVPFMSPASAYQWFYDGYPTFGEHL..QAND
QWF08958.1    ...ELVPQLLQYMYVPPGAPKPKSRDSFAWQTATNPSVFVKMTDPPAQVSVPFMSPASAYQWFYDGYPTFGEHL..QAND
QWF08919.1    ...ELVPQLLQYMYVPPGAPKPKSRDSFAWQTATNPSVFVKMTDPPAQVSVPFMSPASAYQWFYDGYPTFGEHL..QAND
QWY15825.1    ...ELVPQLLQYMYVPPGAPKPKSRDSFAWQTATNPSVFVKMTDPPAQVSVPFMSPASAYQWFYDGYPTFGEHL..QAND
QWF08953.1    ...ELVPQLLQYMYVPPGAPKPKSRDSFAWQTATNPSVFVKMTDPPAQVSVPFMSPASAYQWFYDGYPTFGEHL..QAND
QWF08948.1    ...ELVPQLLQYMYVPPGAPKPKSRDSFAWQTATNPSVFVKMTDPPAQVSVPFMSPASAYQWFYDGYPTFGEHL..QAND
QWY15884.1    ...ELVPQLLQYMYVPPGAPKPKSRDSFAWQTATNPSVFVKMTDPPAQVSVPFMSPASAYQWFYDGYPTFGEHL..QAND
QWF08946.1    ...ELVPQLLQYMYVPPGAPKPKSRDSFAWQTATNPSVFVKMTDPPAQVSVPFMSPASAYQWFYDGYPTFGEHL..QAND
QWF08941.1    ...ELVPQLLQYMYVPPGAPKPKSRDSFAWQTATNPSVFVKMTDPPAQVSVPFMSPASAYQWFYDGYPTFGEHL..QAND
QWF08914.1    ...ELVPQLLQYMYVPPGAPKPKSRDSFAWQTATNPSVFVKMTDPPAQVSVPFMSPASAYQWFYDGYPTFGEHL..QAND
BCU41097.1    ...ELVPQLLQYMYVPPGAPKPKSRDSFAWQTATNPSVFVKMTDPPAQVSVPFMSPASAYQWFYDGYPTFGEHL..QAND
QWY15918.1    ...ELVPQLLQYMYVPPGAPKPKSRDSFAWQTATNPSVFVKMTDPPAQVSVPFMSPASAYQWFYDGYPTFGEHL..QAND
BCR43086.1    ...ELVPQLLQYMYVPPGAPKPKSRDSFAWQTATNPSVFVKMTDPPAQVSVPFMSPASAYQWFYDGYPTFGEHL..QAND
QWF08935.1    ...ELVPQLLQYMYVPPGAPKPKSRDSFAWQTATNPSVFVKMTDPPAQVSVPFMSPASAYQWFYDGYPTFGEHL..QAND
QWY15912.1    ...ELVPQLLQYMYVPPGAPKPKSRDSFAWQTATNPSVFVKMTDPPAQVSVPFMSPASAYQWFYDGYPTFGEHL..QAND
QWY15911.1    ...ELVPQLLQYMYVPPGAPKPKSRDSFAWQTATNPSVFVKMTDPPAQVSVPFMSPASAYQWFYDGYPTFGEHL..QAND
QWY15887.1    ...ELVPQLLQYMYVPPGAPKPKSRDSFAWQTATNPSVFVKMTDPPAQVSVPFMSPASAYQWFYDGYPTFGEHL..QAND
```

```
QWY15882.1    ...ELVPQLLQYMYVPPGAPKPKSRDSFAWQTATNPSVFVKMTDPPAQVSVPFMSPASAYQWFYDGYPTFGEHL..QAND
BCG67018.1    ...ELVPQLLQYMYVPPGAPKPKSRDSFAWQTATNPSVFVKMTDPPAQVSVPFMSPASAYQWFYDGYPTFGEHL..QAND
QWY15881.1    ...ELVPQLLQYMYVPPGAPKPKSRDSFAWQTATNPSVFVKMTDPPAQVSVPFMSPASAYQWFYDGYPTFGEHL..QAND
QWY15875.1    ...ELVPQLLQYMYVPPGAPKPKSRDSFAWQTATNPSVFVKMTDPPAQVSVPFMSPASAYQWFYDGYPTFGEHL..QAND
BCG67016.1    ...ELVPQLLQYMYVPPGAPKPKSRDSFAWQTATNPSVFVKMTDPPAQVSVPFMSPASAYQWFYDGYPTFGEHL..QAND
QWY15874.1    ...ELVPQLLQYMYVPPGAPKPKSRDSFAWQTATNPSVFVKMTDPPAQVSVPFMSPASAYQWFYDGYPTFGEHL..QAND
QWY15853.1    ...ELVPQLLQYMYVPPGAPKPKSRDSFAWQTATNPSVFVKMTDPPAQVSVPFMSPASAYQWFYDGYPTFGEHL..QAND
BCG67014.1    ...ELVPQLLQYMYVPPGAPKPKSRDSFAWQTATNPSVFVKMTDPPAQVSVPFMSPASAYQWFYDGYPTFGEHL..QAND
BCG67009.1    ...ELVPQLLQYMYVPPGAPKPKSRDSFAWQTATNPSVFVKMTDPPAQVSVPFMSPASAYQWFYDGYPTFGEHL..QAND
QWF08933.1    ...ELVPQLLQYMYVPPGAPKPKSRDSFAWQTATNPSVFVKMTDPPAQVSVPFMSPASAYQWFYDGYPTFGEHL..QAND
BCG66992.1    ...ELVPQLLQYMYVPPGAPKPKSRDSFAWQTATNPSVFVKMTDPPAQVSVPFMSPASAYQWFYDGYPTFGEHL..QAND
BCG66991.1    ...ELVPQLLQYMYVPPGAPKPKSRDSFAWQTATNPSVFVKMTDPPAQVSVPFMSPASAYQWFYDGYPTFGEHL..QAND
QWF08898.1    ...ELVPQLLQYMYVPPGAPKPKSRDSFAWQTATNPSVFVKMTDPPAQVSVPFMSPASAYQWFYDGYPTFGEHL..QAND
QWF08930.1    ...ELVPQLLQYMYVPPGAPKPKSRDSFAWQTATNPSVFVKMTDPPAQVSVPFMSPASAYQWFYDGYPTFGEHL..QAND
QWF08925.1    ...ELVPQLLQYMYVPPGAPKPKSRDSFAWQTATNPSVFVKMTDPPAQVSVPFMSPASAYQWFYDGYPTFGEHL..QAND
QWF08913.1    ...ELVPQLLQYMYVPPGAPKPKSRDSFAWQTATNPSVFVKMTDPPAQVSVPFMSPASAYQWFYDGYPTFGEHL..QAND
QWF08911.1    ...ELVPQLLQYMYVPPGAPKPKSRDSFAWQTATNPSVFVKMTDPPAQVSVPFMSPASAYQWFYDGYPTFGEHL..QAND
BBH72117.1    ...ELVPQLLQYMYVPPGAPKPKSRDSFAWQTATNPSVFVKMTDPPAQVSVPFMSPASAYQWFYDGYPTFGEHL..QAND
BBH72116.1    ...ELVPQLLQYMYVPPGAPKPKSRDSFAWQTATNPSVFVKMTDPPAQVSVPFMSPASAYQWFYDGYPTFGEHL..QAND
BBH72113.1    ...ELVPQLLQYMYVPPGAPKPKSRDSFAWQTATNPSVFVKMTDPPAQVSVPFMSPASAYQWFYDGYPTFGEHL..QAND
BBH72112.1    ...ELVPQLLQYMYVPPGAPKPKSRDSFAWQTATNPSVFVKMTDPPAQVSVPFMSPASAYQWFYDGYPTFGEHL..QAND
BBH72108.1    ...ELVPQLLQYMYVPPGAPKPKSRDSFAWQTATNPSVFVKMTDPPAQVSVPFMSPASAYQWFYDGYPTFGEHL..QAND
BBH72106.1    ...ELVPQLLQYMYVPPGAPKPKSRDSFAWQTATNPSVFVKMTDPPAQVSVPFMSPASAYQWFYDGYPTFGEHL..QAND
BBH72104.1    ...ELVPQLLQYMYVPPGAPKPKSRDSFAWQTATNPSVFVKMTDPPAQVSVPFMSPASAYQWFYDGYPTFGEHL..QAND
BBH72211.1    ...ELVPQLLQYMYVPPGAPKPKSRDSFAWQTATNPSVFVKMTDPPAQVSVPFMSPASAYQWFYDGYPTFGEHL..QAND
BBH72210.1    ...ELVPQLLQYMYVPPGAPKPKSRDSFAWQTATNPSVFVKMTDPPAQVSVPFMSPASAYQWFYDGYPTFGEHL..QAND
BBH72209.1    ...ELVPQLLQYMYVPPGAPKPKSRDSFAWQTATNPSVFVKMTDPPAQVSVPFMSPASAYQWFYDGYPTFGEHL..QAND
BBH72208.1    ...ELVPQLLQYMYVPPGAPKPKSRDSFAWQTATNPSVFVKMTDPPAQVSVPFMSPASAYQWFYDGYPTFGEHL..QAND
BBH72207.1    ...ELVPQLLQYMYVPPGAPKPKSRDSFAWQTATNPSVFVKMTDPPAQVSVPFMSPASAYQWFYDGYPTFGEHL..QAND
BBH72205.1    ...ELVPQLLQYMYVPPGAPKPKSRDSFAWQTATNPSVFVKMTDPPAQVSVPFMSPASAYQWFYDGYPTFGEHL..QAND
BBH72201.1    ...ELVPQLLQYMYVPPGAPKPKSRDSFAWQTATNPSVFVKMTDPPAQVSVPFMSPASAYQWFYDGYPTFGEHL..QAND
BBH72193.1    ...ELVPQLLQYMYVPPGAPKPKSRDSFAWQTATNPSVFVKMTDPPAQVSVPFMSPASAYQWFYDGYPTFGEHL..QAND
BBH72192.1    ...ELVPQLLQYMYVPPGAPKPKSRDSFAWQTATNPSVFVKMTDPPAQVSVPFMSPASAYQWFYDGYPTFGEHL..QAND
BBH72190.1    ...ELVPQLLQYMYVPPGAPKPKSRDSFAWQTATNPSVFVKMTDPPAQVSVPFMSPASAYQWFYDGYPTFGEHL..QAND
BBH72189.1    ...ELVPQLLQYMYVPPGAPKPKSRDSFAWQTATNPSVFVKMTDPPAQVSVPFMSPASAYQWFYDGYPTFGEHL..QAND
BBH72188.1    ...ELVPQLLQYMYVPPGAPKPKSRDSFAWQTATNPSVFVKMTDPPAQVSVPFMSPASAYQWFYDGYPTFGEHL..QAND
BBH72187.1    ...ELVPQLLQYMYVPPGAPKPKSRDSFAWQTATNPSVFVKMTDPPAQVSVPFMSPASAYQWFYDGYPTFGEHL..QAND
BBH72185.1    ...ELVPQLLQYMYVPPGAPKPKSRDSFAWQTATNPSVFVKMTDPPAQVSVPFMSPASAYQWFYDGYPTFGEHL..QAND
BBH72182.1    ...ELVPQLLQYMYVPPGAPKPKSRDSFAWQTATNPSVFVKMTDPPAQVSVPFMSPASAYQWFYDGYPTFGEHL..QAND
BBH72181.1    ...ELVPQLLQYMYVPPGAPKPKSRDSFAWQTATNPSVFVKMTDPPAQVSVPFMSPASAYQWFYDGYPTFGEHL..QAND
BBH72179.1    ...ELVPQLLQYMYVPPGAPKPKSRDSFAWQTATNPSVFVKMTDPPAQVSVPFMSPASAYQWFYDGYPTFGEHL..QAND
BBH72178.1    ...ELVPQLLQYMYVPPGAPKPKSRDSFAWQTATNPSVFVKMTDPPAQVSVPFMSPASAYQWFYDGYPTFGEHL..QAND
BBH72177.1    ...ELVPQLLQYMYVPPGAPKPKSRDSFAWQTATNPSVFVKMTDPPAQVSVPFMSPASAYQWFYDGYPTFGEHL..QAND
BBH72175.1    ...ELVPQLLQYMYVPPGAPKPKSRDSFAWQTATNPSVFVKMTDPPAQVSVPFMSPASAYQWFYDGYPTFGEHL..QAND
BBH72174.1    ...ELVPQLLQYMYVPPGAPKPKSRDSFAWQTATNPSVFVKMTDPPAQVSVPFMSPASAYQWFYDGYPTFGEHL..QAND
BBH72172.1    ...ELVPQLLQYMYVPPGAPKPKSRDSFAWQTATNPSVFVKMTDPPAQVSVPFMSPASAYQWFYDGYPTFGEHL..QAND
BBH72170.1    ...ELVPQLLQYMYVPPGAPKPKSRDSFAWQTATNPSVFVKMTDPPAQVSVPFMSPASAYQWFYDGYPTFGEHL..QAND
BBH72169.1    ...ELVPQLLQYMYVPPGAPKPKSRDSFAWQTATNPSVFVKMTDPPAQVSVPFMSPASAYQWFYDGYPTFGEHL..QAND
BBH72168.1    ...ELVPQLLQYMYVPPGAPKPKSRDSFAWQTATNPSVFVKMTDPPAQVSVPFMSPASAYQWFYDGYPTFGEHL..QAND
BBH72158.1    ...ELVPQLLQYMYVPPGAPKPKSRDSFAWQTATNPSVFVKMTDPPAQVSVPFMSPASAYQWFYDGYPTFGEHL..QAND
BBH72155.1    ...ELVPQLLQYMYVPPGAPKPKSRDSFAWQTATNPSVFVKMTDPPAQVSVPFMSPASAYQWFYDGYPTFGEHL..QAND
BBH72154.1    ...ELVPQLLQYMYVPPGAPKPKSRDSFAWQTATNPSVFVKMTDPPAQVSVPFMSPASAYQWFYDGYPTFGEHL..QAND
BBH72151.1    ...ELVPQLLQYMYVPPGAPKPKSRDSFAWQTATNPSVFVKMTDPPAQVSVPFMSPASAYQWFYDGYPTFGEHL..QAND
BBH72148.1    ...ELVPQLLQYMYVPPGAPKPKSRDSFAWQTATNPSVFVKMTDPPAQVSVPFMSPASAYQWFYDGYPTFGEHL..QAND
BBH72144.1    ...ELVPQLLQYMYVPPGAPKPKSRDSFAWQTATNPSVFVKMTDPPAQVSVPFMSPASAYQWFYDGYPTFGEHL..QAND
BBH72142.1    ...ELVPQLLQYMYVPPGAPKPKSRDSFAWQTATNPSVFVKMTDPPAQVSVPFMSPASAYQWFYDGYPTFGEHL..QAND
BBH72141.1    ...ELVPQLLQYMYVPPGAPKPKSRDSFAWQTATNPSVFVKMTDPPAQVSVPFMSPASAYQWFYDGYPTFGEHL..QAND
BBH72140.1    ...ELVPQLLQYMYVPPGAPKPKSRDSFAWQTATNPSVFVKMTDPPAQVSVPFMSPASAYQWFYDGYPTFGEHL..QAND
BBH72132.1    ...ELVPQLLQYMYVPPGAPKPKSRDSFAWQTATNPSVFVKMTDPPAQVSVPFMSPASAYQWFYDGYPTFGEHL..QAND
BBH72131.1    ...ELVPQLLQYMYVPPGAPKPKSRDSFAWQTATNPSVFVKMTDPPAQVSVPFMSPASAYQWFYDGYPTFGEHL..QAND
BBH72130.1    ...ELVPQLLQYMYVPPGAPKPKSRDSFAWQTATNPSVFVKMTDPPAQVSVPFMSPASAYQWFYDGYPTFGEHL..QAND
BBH72128.1    ...ELVPQLLQYMYVPPGAPKPKSRDSFAWQTATNPSVFVKMTDPPAQVSVPFMSPASAYQWFYDGYPTFGEHL..QAND
BDC16283.1    ...ELVPQLLQYMYVPPGAPKPKSRDSFAWQTATNPSVFVKMTDPPAQVSVPFMSPASAYQWFYDGYPTFGEHL..QAND
BDC16271.1    ...ELVPQLLQYMYVPPGAPKPKSRDSFAWQTATNPSVFVKMTDPPAQVSVPFMSPASAYQWFYDGYPTFGEHL..QAND
BDC16270.1    ...ELVPQLLQYMYVPPGAPKPKSRDSFAWQTATNPSVFVKMTDPPAQVSVPFMSPASAYQWFYDGYPTFGEHL..QAND
BDC16258.1    ...ELVPQLLQYMYVPPGAPKPKSRDSFAWQTATNPSVFVKMTDPPAQVSVPFMSPASAYQWFYDGYPTFGEHL..QAND
QIL87526.1    ...ELVPQLLQYMYVPPGAPKPKSRDSFAWQTATNPSVFVKMTDPPAQVSVPFMSPASAYQWFYDGYPTFGEHL..QAND
BBH72180.1    ...ELVPQLLQYMYVPPGAPKPKSRDSFAWQTATNPSVFVKMTDPPAQVSVPFMSPASAYQWFYDGYPTFGQHL..QAND
BBH72109.1    ...ELVPQLLQYMYVPPGAPKPKSRDSFAWQTATNPSVFVKMTDPPAQVSVPFMSPASAYQWFYDGYPTFGEHL..QAND
BBH72115.1    ...ELVPQLLQYMYVPPGAPKPKSRDSFAWQTATNPSVFVKMTDPPAQVSVPFMSPASAYQWFYDGYPTFGEHL..QAND
BBH72152.1    ...ELVPQLLQYMYVPPGAPKPKSRDSFAWQTATNPSVFVKMTDPPAQVSVPFMSPASAYQWFYDGYPTFGEHL..QAND
BBH72200.1    ...ELVPQLLQYMYVPPGAPKPKSRDSFAWQTATNPSVFVKMTDPPAQVSVPFMSPASAYQWFYDGYPTFGEHL..QAND
QWY15844.1    ...ELVPQLLQYMYVPPGAPKPKSRDSFAWQTATNPSVFVKMTDPPAQVSVPFMSPASAYQWFYDGYPTFGEHL..QAND
BBH72146.1    ...ELVPQLLQYMYVPPGAPKPKSRDSFAWQTATNPSVFVKMTDPPAQVSVPFMSPASAYQWFYDGYPTFGEHL..QAND
BBH72173.1    ...ELVPQLLQYMYVPPGAPKPKSRDSFAWQTATNPSVFVKMTDPPAQVSVPFMSPASAYQWFYDGYPTFGEHL..QAND
QWY15947.1    ...ELVPQLLQYMYVPPGAPKPKSRDSFAWQTATNPSVFVKMTDPPAQVSVPFMSPASAYQWFYDGYPTFGEHL..QAND
BDC16281.1    ...ELVPQLLQYMYVPPGAPKPKSRDSFAWQTATNPSVFVKMTDPPAQVSVPFMSPASAYQWFYDGYPTFGEHL..QAND
BBH72167.1    ...ELVPQLLQYMYVPPGAPRPKSRDSFAWQTATNPSVFVKMTDPPAQVSVPFMSPASAYQWFYDGYPTFGEHL..QAND
BBH72160.1    ...ELVPQLLQYMYVPPGAPRPKSRDSFAWQTATNPSVFVKMTDPPAQVSVPFMSPASAYQWFYDGYPTFGEHL..QAND
BCU41096.1    ...ELVPQLLQYMYVPPGAPKPKSRDSFAWQTATNPSVFVKMTDPPAQVSVPFMSPASAYQWFYDGYPTFGEHL..QAND
BCR43102.1    ...ELVPQLLQYMYVPPGAPKPKSRDSFAWQTATNPSVFVKMTDPPAQVSVPFMSPASAYQWFYDGYPTFGEHL..QAND
QWF08881.1    ...ELVPQLLQYMYVPPGAPKPKSRDSFAWQTATNPSVFVKMTDPPAQVSVPFMSPASAYQWFYDGYPTFGEHL..QAND
QWF08855.1    ...ELVPQLLQYMYVPPGAPKPKSRDSFAWQTATNPSVFVKMTDPPAQVSVPFMSPASAYQWFYDGYPTFGEHL..QAND
BBH72195.1    ...ELVPQLLQYMYVPPGAPKPKSRDSFAWQTATNPSVFVKMTDPPAQVSVPFMSPASAYQWFYDGYPTFGEHL..QAND
BBH72118.1    ...ELVPQLLQYMYVPPGAPKPKSRDSFAWQTATNPSVFVKMTDPPAQVSVPFMSPASAYQWFYDGYPTFGEHL..QAND
BBH72126.1    ...ELVPQLLQYMYVPPGAPKPKSRDSFAWQTATNPSVFVKMTDPPAQVSVPFMSPASAYQWFYDGYPTFGEHL..QAND
BBH72127.1    ...ELVPQLLQYMYVPPGAPKPKSRDSFAWQTATNPSVFVKMTDPPAQVSVPFMSPASAYQWFYDGYPTFGEHL..QAND
BBH72206.1    ...ELVPQLLQYMYVPPGAPKPKSRDSFAWQTATNPSVFVKMTDPPAQVSVPFMSPASAYQWFYDGYPTFGEHL..QAND
BBH72196.1    ...ELVPQLLQYMYVPPGAPKPKSRDSFAWQTATNPSVFVKMTDPPAQVSVPFMSPASAYQWFYDGYPTFGEHL..QAND
BBH72171.1    ...ELVPQLLQYMYVPPGAPKPKSRDSFAWQTATNPSVFVKMTDPPAQVSVPFMSPASAYQWFYDGYPTFGEHL..QAND
BBH72191.1    ...ELVPQLLQYMYVPPGAPKPKSRDSFAWQTATNPSVFVKMTDPPAQVSVPFMSPASAYQWFYDGYPTFGEHL..QAND
BBH72133.1    ...ELVPQLLQYMYVPPGAPKPKSRDSFAWQTATNPSVFVKMTDPPAQVSVPFMSPASAYQWFYDGYPTFGEHL..QAND
BBH72139.1    ...ELVPQLLQYMYVPPGAPKPKSRDSFAWQTATNPSVFVKMTDPPAQVSVPFMSPASAYQWFYDGYPTFGEHL..QAND
BBH72166.1    ...ELVPQLLQYMYVPPGAPKPKSRDSFAWQTATNPFVFVKMTDPPAQVSVPFMSPASVYQWFYDGYPTFGEHF..QAND
BBH72161.1    ...ELVPQLLQYMYVPPGAPKPKSRDSFAWQTATNPFVFVKMTDPPAQVSVPFMSPASAYQWFYDGYPTFGEHL..QANV
BBH72165.1    ...ELVPQLLQYMYVPPGAPKPKSRDSFAWQTATNPSVFVKMTDPPAQVSVPFMSPASAYQWFYDGYPTFGEHL..QAND
BBH72164.1    ...ELVPQLLQYMYVPPGAPKPKSRDSFAWQTATNPSVFVKMTDPPAQVSVPFMSPASAYQWFYDGYPTFGEHL..QANV
BBH72159.1    ...ELVPQLLQYMYVPPGAPKPKSRDSFAWQTATNRFVFVKMTDPPAQVSVPFMSPASAYQWFYDGYPTFGEHF..QAND
BBH72157.1    ...ELVPQLLQYMYVPPGAPKPKSRDSFAWQTATNPSVFVKMTDPPAQVSVPFMSPASAYQWFYDGYPTFGEHF..QAND
BBH72162.1    ...ELVPQLLQYMYVPPGAPKPKSRDSFAWQTATNPSVFVKMTDPPAQVSVPFMSPASAYQWFYDGYPTFGEHL..QAND
BBH72153.1    ...ELVPQLLQYMYVPPGAPKPKSRDSFAWQTATNPSVFVKMTDPPAQVSVPFMSPASAYQWFYDGYPTFGEHL..QANV
BBH72156.1    ...ELVPQLLQYMYVPPGAPKPKSRDSFAWQTATNPSVFVKMTDPPAQVSVPFMSPASAYQWFYDGYPTFGEHL..QAND
BBH72120.1    ...ELVPQLLQYMYVPPGAPKPKSRDSFAWQTATNPSVFVKMTDPPAQVSVPFMSPASAYQWFYDGYPTFGEHL..QAND
BBH72198.1    ...ELVPQLLQYMYVPPGAPKPKSRDSFAWQTATNPSVFVKMTDPPAQVSVPFMSPASAYQWFYDGYPTFGEHF..QAND
BBH72197.1    ...ELVPQLLQYMYVPPGAPKPKSRDSFAWQTATNPSVFVKMTDPPAQVSVPFMSPASAYQWFYDGYPTFGEHL..QAND
QWY15941.1    ...ELVPQLLQYMYVPPGAPKPKSRDSFAWQTATNPSVFVKMTDPPAQVSVPFMSPASAYQWFYDGYPTFGEHL..QAND
BBH72121.1    ...ELVPQLLQYMYVPPGAPKPKSRDSFAWQTATNPSVFVKMTDPPAQVSVPFMSPASAYQWFYDGYPTFGEHL..QAND
BBH72135.1    ...ELVPQLLQYMYVPPGAPKPKSRDSFAWQTATNPSVFVKMTDPPAQVSVPFMSPASAYQWFYDGYPTFGEHL..QAND
QWY15967.1    ...ELVPQLLQYMYVPPGAPKPKSRDSFAWQTATNPSVFVKMTDPPAQVSVPFMSPASAYQWFYDGYPTFGEHL..QADD
QWY15964.1    ...ELVPQLLQYMYVPPGAPKPKSRDSFAWQTATNPSVFVKMTDPPAQVSVPFMSPASAYQWFYDGYPTFGEHL..QADD
QWY15969.1    ...ELVPQLLQYMYVPPGAPKPKSRDSFAWQTATNPSVFVKMTDPPAQVSVPFMSPASAYQWFYDGYPTFGEHL..QADD
QWY15913.1    ...ELVPQLLQYMYVPPGAPKPKSRDSFAWQTATNPSVFVKMTDPPAQVSVPFMSPASAYQWFYDGYPTFGEHL..QADD
QWY15908.1    ...ELVPQLLQYMYVPPGAPKPKSRDSFAWQTATNPSVFVKMTDPPAQVSVPFMSPASAYQWFYDGYPTFGEHL..QADD
QWY15907.1    ...ELVPQLLQYMYVPPGAPKPKSRDSFAWQTATNPSVFVKMTDPPAQVSVPFMSPASAYQWFYDGYPTFGEHL..QADD
QWY15890.1    ...ELVPQLLQYMYVPPGAPKPKSRDSFAWQTATNPSVFVKMTDPPAQVSVPFMSPASAYQWFYDGYPTFGEHL..QADD
QWY15889.1    ...ELVPQLLQYMYVPPGAPKPKSRDSFAWQTATNPSVFVKMTDPPAQVSVPFMSPASAYQWFYDGYPTFGEHL..QADD
QWY15888.1    ...ELVPQLLQYMYVPPGAPKPKSRDSFAWQTATNPSVFVKMTDPPAQVSVPFMSPASAYQWFYDGYPTFGEHL..QADD
QWY15886.1    ...ELVPQLLQYMYVPPGAPKPKSRDSFAWQTATNPSVFVKMTDPPAQVSVPFMSPASAYQWFYDGYPTFGEHL..QADD
QWY15885.1    ...ELVPQLLQYMYVPPGAPKPKSRDSFAWQTATNPSVFVKMTDPPAQVSVPFMSPASAYQWFYDGYPTFGEHL..QADD
QWY15883.1    ...ELVPQLLQYMYVPPGAPKPKSRDSFAWQTATNPSVFVKMTDPPAQVSVPFMSPASAYQWFYDGYPTFGEHL..QADD
QWY15878.1    ...ELVPQLLQYMYVPPGAPKPKSRDSFAWQTATNPSVFVKMTDPPAQVSVPFMSPASAYQWFYDGYPTFGEHL..QADD
QWY15876.1    ...ELVPQLLQYMYVPPGAPKPKSRDSFAWQTATNPSVFVKMTDPPAQVSVPFMSPASAYQWFYDGYPTFGEHL..QADD
BBH72176.1    ...ELVPQLLQYMYVPPGAPKPKSRDSFAWQTATNPSVFVKMTDPPAQVSVPFMSPASAYQWFYDGYPTFGEHL..QADD
QIL87519.1    ...ELVPQLLQYMYVPPGAPKPKSRDSFAWQTATNPSVFVKMTDPPAQVSVPFMSPASAYQWFYDGYPTFGEHL..QADD
QWY15906.1    ...GLVPQLLQYMYVPPGAPKPKSRDSFAWQTATNPSVFVKMTDPPAQVSVPFMSPASAYQWFYDGYPTFGEHL..QADD
QWY15917.1    ...GLVPQLLQYMYVPPGAPKPKSRDSFAWQTATNPSVFVKMTDPPAQVSVPFMSPASAYQWFYDGYPTFGEHL..QAND
QWY15846.1    ...ALVPQLLQYMYVPPGAPKPKSRDSFAWQTATNPSVFVKMTDPPAQVSVPFMSPASAYQWFYDGYPTFGEHL..QAND
QWY15901.1    ...GLVPQLLQYMYVPPGAPKPKSRDSFAWQTATNPSVFVKMTDPPAQVSVPFMSPASAYQWFYDGYPTFGEHL..QAND
QWY15857.1    ...GLVPQLLQYMYVPPGAPKPKSRDSFAWQTATNPSVFVKMTDPPAQVSVPFMSPASAYQWFYDGYPTFGEHL..QAND
QWY15904.1    ...VLVPQLLQYMYVPPGAPKPKSRDSFAWQTATNPSVFVKMTDPPAQVSVPFMSPASAYQWFYDGYPTFGEHL..QAND
BBH72204.1    ...ELVPQLLQYMYVPPGAPKPKSRDSFAWQTATNPSVFVKMTDPPAQVSVPFMSPASAYQWFYDGYPTFGEHL..QAND
BBH72203.1    ...ELVPQLLQYMYVPPGAPKPKSRDSFAWQTATNPSVFVKMTDPPAQVSVPFMSPASAYQWFYDGYPTFGEHL..QAND
BBH72202.1    ...ELVPQLLQYMYVPPGAPKPKSRDSFAWQTATNPSVFVKMTDPPAQVSVPFMSPASAYQWFYDGYPTFGEHL..QAND
BDC16266.1    ...ELVPQLLQYMYVPPGAPKPKSRDSFAWQTATNPSVFVKMTDPPAQVSVPFMSPASAYQWFYDGYPTFGEHL..QAHD
BCR43091.1    ...ELVPQLLQYMYVPPGAPKPKSRDSFAWQTATNPSVFVKMTDPPAQVSVPFMSPASAYQWFYDGYPTFGEHL..QAND
QWY15902.1    ...ELVPQLLQYMYVPPGAPKPKSRDSFAWQTATNPSVFVKMTDPPAQVSVPFMSPASAYQWFYDGYPTFGEHL..QAND
BBH72226.1    ...LTNNSVIQYMYVPPGAPKPDARESFQWQTATNPSVFQKMDSPPPQVSVPFMSPASAYQWFYDGYPTFGPHS..ETSN
BBH72222.1    ...LTNNSVIQYMYVPPGAPKPDARESFQWQTATNPSVFQKMDSPPPQVSVPFMSPASAYQWFYDGYPTFGPHS..ETSN
BBH72012.1    ...TTPGMLLQYMYVPPGAPKPDSRKSYQWQTATNPSVFAKLSDPPPQVSVPFMSPATAYQWFYDGYPTFGEHK..QATN
BBH71965.1    ...TTPGMLLQYMYVPPGAPKPDSRKSYQWQTATNPSVFAKLSDPPPQVSVPFMSPATAYQWFYDGYPTFGEHK..QATN
BBH71934.1    ...TTPGMLLQYMYVPPGAPKPDSRKSYQWQTATNPSVFAKLSDPPPQVSVPFMSPATAYQWFYDGYPTFGEHK..QATN
BBH72009.1    ...TTPGMLLQYMYVPPGAPKPDSRKSYQWQTATNPSVFAKLSDPPPQVSVPFMSPATAYQWFYDGYPTFGEHK..QATN
QWY16175.1    ...NTSPIPVQYMYVPPGAPVPTGRGTFQWQTATNPSVISKMTDPPAQVSVPFMSPASTYQWFYDGYPTFGEVP..VTTN
QWY16167.1    ...NTSPIPVQYMYVPPGAPVPTGRDTFQWQTATNPSVISKMTDPPAQVSVPFMSPASTYQWFYDGYPTFGEVP..ATTN
```

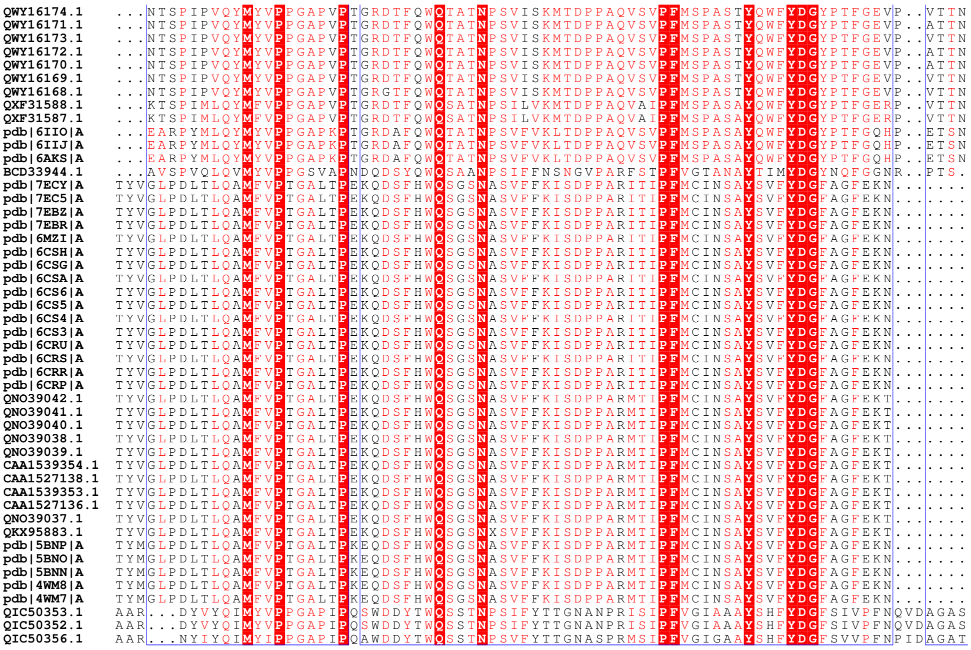
QWY16174.1 ...NTSPIPVQYMYVPPGAPVPTGRDTFQWQTATNPSVISKMTDPPAQVSVPFMSPASTYQWFYDGYPTFGEVP..VTTN
QWY16171.1 ...NTSPIPVQYMYVPPGAPVPTGRDTFQWQTATNPSVISKMTDPPAQVSVPFMSPASTYQWFYDGYPTFGEVP..ATTN
QWY16173.1 ...NTSPIPVQYMYVPPGAPVPTGRDTFQWQTATNPSVISKMTDPPAQVSVPFMSPASTYQWFYDGYPTFGEVP..VTTN
QWY16172.1 ...NTSPIPVQYMYVPPGAPVPTGRDTFQWQTATNPSVISKMTDPPAQVSVPFMSPASTYQWFYDGYPTFGEVP..ATTN
QWY16170.1 ...NTSPIPVQYMYVPPGAPVPTGRDTFQWQTATNPSVISKMTDPPAQVSVPFMSPASTYQWFYDGYPTFGEVP..ATTN
QWY16169.1 ...NTSPIPVQYMYVPPGAPVPTGRDTFQWQTATNPSVISKMTDPPAQVSVPFMSPASTYQWFYDGYPTFGEVP..VTTN
QWY16168.1 ...NTSPIPVQYMYVPPGAPVPTGRGTFQWQTATNPSVISKMTDPPAQVSVPFMSPASTYQWFYDGYPTFGEVP..VTTN
QXF31588.1 ...KTSPIMLQYMFVPPGAPVPTGRDTFQWQSATNPSILVKMTDPPAQVAIPFMSPASAYQWFYDGYPTFGERP..VTTN
QXF31587.1 ...KTSPIMLQYMFVPPGAPVPTGRDTFQWQSATNPSILVKMTDPPAQVAIPFMSPASAYQWFYDGYPTFGERP..VTTN
pdb|6IIO|A ...EARPYMLQYMYVPPGAPKPTGRDAFQWQTATNPSVFVKLTDPPAQVSVPFMSPASAYQWFYDGYPTFGQHP..ETSN
pdb|6IIJ|A ...EARPYMLQYMYVPPGAPKPTGRDAFQWQTATNPSVFVKLTDPPAQVSVPFMSPASAYQWFYDGYPTFGQHP..ETSN
pdb|6AKS|A ...EARPYMLQYMYVPPGAPKPTGRDAFQWQTATNPSVFVKLTDPPAQVSVPFMSPASAYQWFYDGYPTFGQHP..ETSN
BCD33944.1 ...AVSPVQLQVMYVPPGSVAPNDQDSYQWQSAANPSIFFNSNGVPARFSTPFVGTANAYTIMYDGYNQFGGNR..PTS.
pdb|7ECY|A TYVGLPDLTLQAMFVPTGALTPEKQDSFHWQSGSNASVFFKISDPPARITIPFMCINSAYSVFYDGFAGFEKN.......
pdb|7EC5|A TYVGLPDLTLQAMFVPTGALTPEKQDSFHWQSGSNASVFFKISDPPARITIPFMCINSAYSVFYDGFAGFEKN.......
pdb|7EBZ|A TYVGLPDLTLQAMFVPTGALTPEKQDSFHWQSGSNASVFFKISDPPARITIPFMCINSAYSVFYDGFAGFEKN.......
pdb|7EBR|A TYVGLPDLTLQAMFVPTGALTPEKQDSFHWQSGSNASVFFKISDPPARITIPFMCINSAYSVFYDGFAGFEKN.......
pdb|6MZI|A TYVGLPDLTLQAMFVPTGALTPEKQDSFHWQSGSNASVFFKISDPPARITIPFMCINSAYSVFYDGFAGFEKN.......
pdb|6CSH|A TYVGLPDLTLQAMFVPTGALTPEKQDSFHWQSGSNASVFFKISDPPARITIPFMCINSAYSVFYDGFAGFEKN.......
pdb|6CSG|A TYVGLPDLTLQAMFVPTGALTPEKQDSFHWQSGSNASVFFKISDPPARITIPFMCINSAYSVFYDGFAGFEKN.......
pdb|6CSA|A TYVGLPDLTLQAMFVPTGALTPEKQDSFHWQSGSNASVFFKISDPPARITIPFMCINSAYSVFYDGFAGFEKN.......
pdb|6CS6|A TYVGLPDLTLQAMFVPTGALTPEKQDSFHWQSGSNASVFFKISDPPARITIPFMCINSAYSVFYDGFAGFEKN.......
pdb|6CS5|A TYVGLPDLTLQAMFVPTGALTPEKQDSFHWQSGSNASVFFKISDPPARITIPFMCINSAYSVFYDGFAGFEKN.......
pdb|6CS4|A TYVGLPDLTLQAMFVPTGALTPEKQDSFHWQSGSNASVFFKISDPPARITIPFMCINSAYSVFYDGFAGFEKN.......
pdb|6CS3|A TYVGLPDLTLQAMFVPTGALTPEKQDSFHWQSGSNASVFFKISDPPARITIPFMCINSAYSVFYDGFAGFEKN.......
pdb|6CRU|A TYVGLPDLTLQAMFVPTGALTPEKQDSFHWQSGSNASVFFKISDPPARITIPFMCINSAYSVFYDGFAGFEKN.......
pdb|6CRS|A TYVGLPDLTLQAMFVPTGALTPEKQDSFHWQSGSNASVFFKISDPPARITIPFMCINSAYSVFYDGFAGFEKN.......
pdb|6CRR|A TYVGLPDLTLQAMFVPTGALTPEKQDSFHWQSGSNASVFFKISDPPARITIPFMCINSAYSVFYDGFAGFEKN.......
pdb|6CRP|A TYVGLPDLTLQAMFVPTGALTPEKQDSFHWQSGSNASVFFKISDPPARITIPFMCINSAYSVFYDGFAGFEKN.......
QNO39042.1 TYVGLPDLTLQAMFVPTGALTPEKQDSFHWQSGSNASVFFKISDPPARMTIPFMCINSAYSVFYDGFAGFEKT.......
QNO39041.1 TYVGLPDLTLQAMFVPTGALTPEKQDSFHWQSGSNASVFFKISDPPARMTIPFMCINSAYSVFYDGFAGFEKT.......
QNO39040.1 TYVGLPDLTLQAMFVPTGALTPEKQDSFHWQSGSNASVFFKISDPPARMTIPFMCINSAYSVFYDGFAGFEKT.......
QNO39038.1 TYVGLPDLTLQAMFVPTGALTPEKQDSFHWQSGSNASVFFKISDPPARMTIPFMCINSAYSVFYDGFAGFEKT.......
QNO39039.1 TYVGLPDLTLQAMFVPTGALTPEKQDSFHWQSGSNASVFFKISDPPARMTIPFMCINSAYSVFYDGFAGFEKT.......
CAA1539354.1 TYVGLPDLTLQAMFVPTGALTPEKQDSFHWQSGSNASVFFKISDPPARMTIPFMCINSAYSVFYDGFAGFEKT.......
CAA1527138.1 TYVGLPDLTLQAMFVPTGALTPEKQDSFHWQSGSNASVFFKISDPPARMTIPFMCINSAYSVFYDGFAGFEKT.......
CAA1539353.1 TYVGLPDLTLQAMFVPTGALTPEKQDSFHWQSGSNASVFFKISDPPARMTIPFMCINSAYSVFYDGFAGFEKT.......
CAA1527136.1 TYVGLPDLTLQAMFVPTGALTPEKQDSFHWQSGSNASVFFKISDPPARMTIPFMCINSAYSVFYDGFAGFEKT.......
QNO39037.1 TYVGLPDLTLQAMFVPTGALTPEKQDSFHWQSGSNASVFFKISDPPARMTIPFMCINSAYSVFYDGFAGFEKT.......
QKX95883.1 TYVGLPDLTLQAMFVPTGALTPEKQDSFHWQSGSNXSVFFKISDPPARMTIPFMCINSAYSVFYDGFAGFEKT.......
pdb|5BNP|A TYMGLPDLTLQAMFVPTGALTPKEQDSFHWQSGSNASVFFKISDPPARMTIPFMCINSAYSVFYDGFAGFEKN.......
pdb|5BNO|A TYMGLPDLTLQAMFVPTGALTPKEQDSFHWQSGSNASVFFKISDPPARMTIPFMCINSAYSVFYDGFAGFEKN.......
pdb|5BNN|A TYMGLPDLTLQAMFVPTGALTPKEQDSFHWQSGSNASVFFKISDPPARMTIPFMCINSAYSVFYDGFAGFEKN.......
pdb|4WM8|A TYMGLPDLTLQAMFVPTGALTPKEQDSFHWQSGSNASVFFKISDPPARMTIPFMCINSAYSVFYDGFAGFEKN.......
pdb|4WM7|A TYMGLPDLTLQAMFVPTGALTPKEQDSFHWQSGSNASVFFKISDPPARMTIPFMCINSAYSVFYDGFAGFEKN.......
QIC50353.1 AAR...DYVYQIMYVPPGAPIPQSWDDYTWQSSTNPSIFYTTGNANPRISIPFVGIAAAYSHFYDGFSIVPFNQVDAGAS
QIC50352.1 AAR...DYVYQIMYVPPGAPIPQSWDDYTWQSSTNPSIFYTTGNANPRISIPFVGIAAAYSHFYDGFSIVPFNQVDAGAS
QIC50356.1 AAR...NYIYQIMYIPPGAPIPQAWDDYTWQSSTNPSVFYTTGNASPRMSIPFVGIGAAYSHFYDGFSVVPFNPIDAGAT

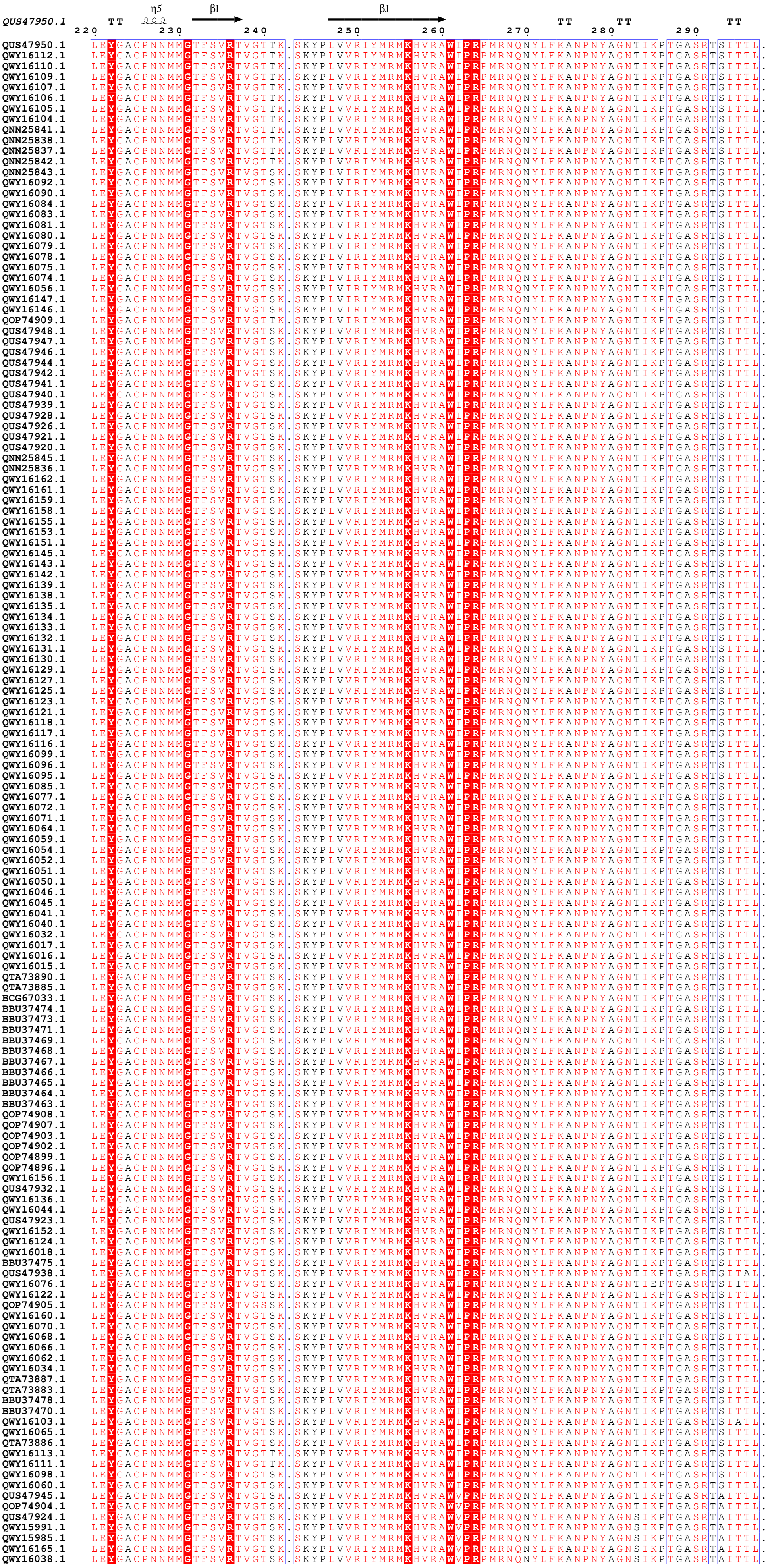
QUS47950.1
η5
βI
βJ
TT
TT
TT
TT
220
230
240
250
260
270
280
290

```
QWY16036.1    LEYGACPNNMMGTFSVRTVGTSK.SKYPLVVRIYMRMKHVRAWVPRPMRNQNYLFKANPNYAGNSIKPTGASRTAITTL.
QWY16120.1    LEYGACPNNMMGTFSVRTVGTSK.SKYPLVVRIYMRMKHVRAWVPRPMRNQNYLFKANPNYAGNTIKPTGASRTSITTL.
QWY16119.1    LEYGACPNNMMGTFSVRTVGTSK.SKYPLVVRIYMRMKHVRAWVPRPMRNQNYLFKANPNYAGNTIKPTGASRTSITTL.
QWY16102.1    LEYGACPNNMMGTFSVRTVGTSK.SKYPLVVRIYMRMKHVRAWVPRPMRNQNYLFKANPNYAGNTIKPTGASRTSITTL.
QWY16100.1    LEYGACPNNMMGTFSVRTVGTSK.SKYPLVVRIYMRMKHVRAWVPRPMRNQNYLFKANPNYAGNTIKPTGASRTSITTL.
QWY16097.1    LEYGACPNNMMGTFSVRTVGTSK.SKYPLVVRIYMRMKHVRAWVPRPMRNQNYLFKANPNYAGNTIKPTGASRTSITTL.
QWY16101.1    LEYGACPNNMMGTFSVRTVGTSK.SKYPLVIRIYMRMKHVRAWVPRPMRNQNYLFKANPNYAGNTIKPTGASRTSITTL.
BBU37472.1    LEYGACPNNMMGTFSVRTVGTSK.SKYPLVVRIYMRMKHVRAWIPRPMRNQNYLFKANPNYAGNIIKPTGASRTSITTL.
QWY16108.1    LEYGACPNNMMGTFSVRTVGTSK.SKYPLVVRIYMRMKHVRAWIPRPMRNQNYLFKANPNYAGNTIKPTGASRTSITTL.
QWY16042.1    LEYGACPNNMMGTFSVRTVGTSK.SKYPLVVRIYMRMKHVRAWIPRPMRNQNYLFKANPNYAGNTIKPTGASRTSITTL.
QOP74910.1    LEYGACPNNMMGTFSVRTVGTSK.SKYPLVVRIYMRMKHVRAWIPRPMRNQNYLFKANPNYAGNTIKPTGASRSSITTL.
QWY16128.1    LEYGACPNNMMGTFSVRTVGTSK.SKYPLVVRIYMRMKHVRAWIPRPMRNQNYLFKANPNYAGNTIKPTGASRTSITTL.
QUS47905.1    LEYGACPNNMMGTFSVRTVGTSK.SKYPLVVRIYMRMKHVRAWIPRPMRNQNYLFKANPNYAGNTIKPTGASRTSITTL.
QWY16154.1    LEYGACPNNMMGTFSVRTVGTSK.SKYPLVVRIYMRMKHVRAWIPRPMRNQNYLFKANPNYAGNTIKPTGASRTSITTL.
QWY16126.1    LEYGACPNNMMGTFSVRTVGTSK.SKYPLVVRIYMRMKHVRAWIPRPMRNQNYLFKANPNYAGNTIKPTGASRTSITTL.
QWY16049.1    LEYGACPNNMMGTFSVRTVGTSK.SKYPLVVRIYMRMKHVRAWIPRPMRNQNYLFKANPNYAGNSIKPTGASRTSITTL.
QWY16043.1    LEYGACPNNMMGTFSVRTVGTSK.SKYPLVVRIYMRMKHVRAWIPRPMRNQNYLFKANPNYAGNSIKPTGASRTSITTL.
QUS47911.1    LEYGACPNNMMGTFSVRTVGTSK.SKYPLVIRIYMRMKHVRAWIPRPMRNQNYLFKANPNYAGNTIKPTGASRTAITTL.
BBU37477.1    LEYGACPNNMMGTFSVRTVGTSK.SKYPLVVRIYMRMKHVRAWIPRPMRNQNYLFKANPNYAGNTIKPTGASRTSITTL.
QWY16137.1    LEYGACPNNMMGTFSVRTVGTSK.SKYPLVVRIYMRMKHVRAWIPRPMRNQNYLFKANPNYAGNTIKPTGTSRTSITTL.
QWY16061.1    LEYGACPNNMMGTFSVRTVGTSK.SKYPLVVRIYMRMKHVRAWIPRPMRNQNYLFKANPNYAGNTIKPTGTSRTSITTL.
QTA73882.1    LEYGACPNNMMGTFSVRTVGTSK.SKYPLVVRIYMRMKHVRAWIPRPMRNQNYLFKANPNYAGNTIKPTGTSRTSITTL.
QWY16031.1    LEYGACPNNMMGTFSVRTVGTSK.SKYPLVVRIYMRMKHVRAWIPRPMRNQNYLFKANPNYAGNTIKPTGASRTSITTL.
BBU37476.1    LEYGACPNNMMGTFSVRTVGTSK.SKYPLVVRIYMRMKHVRAWIPRPMRNQNYLFKANPNYAGNTIKPTGASRTSITTL.
QUS47949.1    LDYGACPNNMMGTFSVRTVGTTK.SKYPLVVRIYMRMKHVRAWIPRPMRNQNYLFKANPNYAGNTIKPTGASRTSITTL.
QNN25833.1    LEYGACPNNMMGTFSVRTVGTTK.SKYPLVVRIYMRMKHVRAWIPRPMRNQNYLFKANPNYAGNTIKPTGASRTSITTL.
QWY16114.1    LEYGACPNNMMGTFSVRTVGTTK.SKYPLVVRIYMRMKHVRAWIPRPMRNQNYLFKANPNYAGNTIKPTGASRTSITTL.
QWY16063.1    LEYGACPNNMMGTFSVRTVGTSK.SKYPLVVRIYMRMKHVRAWIPRPMRNQNYLFKANPNYAGNTIKPTGASRTSITTL.
QTA73884.1    LEYGACPNNMMGTFSVRTVGTSK.SKYPLVVRIYMRMKHVRAWIPRPMRNQNYLFKANPNYAGNTIKPTGASRTSITTL.
QWY16002.1    LEYGACPNNMMGTFSVRTVGTSK.SKYPLVVRIYMRMKHVRAWIPRPMRNQNYLFKANPNYAGNSIKPTGASRTAITTL.
QWY15984.1    LEYGACPNNMMGTFSVRTVGTSK.SKYPLVVRIYMRMKHVRAWIPRPMRNQNYLFKANPNYAGNSIKPTGASRTVITTL.
QUS47934.1    LEYGACPNNMMGTFSVRTVGTSK.SKYPLVVRIYMRMKHVRAWIPRPMRNQNYLFKANPNYAGNSIKPTGASRTAITTL.
QUS47933.1    LEYGACPNNMMGTFSVRTVGTSK.SKYPLVVRIYMRMKHVRAWIPRPMRNQNYLFKANPNYAGNSIKPTGASRTAITTL.
QUS47929.1    LEYGACPNNMMGTFSVRTVGTSK.SKYPLVVRIYMRMKHVRAWIPRPMRNQNYLFKANPNYAGNSIKPTGASRTAITTL.
QUS47927.1    LEYGACPNNMMGTFSVRTVGTSK.SKYPLVVRIYMRMKHVRAWIPRPMRNQNYLFKANPNYAGNSIKPTGASRTAITTL.
QUS47919.1    LEYGACPNNMMGTFSVRTVGTSK.SKYPLVVRIYMRMKHVRAWIPRPMRNQNYLFKANPNYAGNSIKPTGASRTAITTL.
QUS47917.1    LEYGACPNNMMGTFSVRTVGTSK.SKYPLVVRIYMRMKHVRAWIPRPMRNQNYLFKANPNYAGNSIKPTGASRTAITTL.
QUS47915.1    LEYGACPNNMMGTFSVRTVGTSK.SKYPLVVRIYMRMKHVRAWIPRPMRNQNYLFKANPNYAGNSIKPTGASRTAITTL.
QUS47913.1    LEYGACPNNMMGTFSVRTVGTSK.SKYPLVVRIYMRMKHVRAWIPRPMRNQNYLFKANPNYAGNSIKPTGASRTAITTL.
QWY16166.1    LEYGACPNNMMGTFSVRTVGTSK.SKYPLVVRIYMRMKHVRAWIPRPMRNQNYLFKANPNYAGNSIKPTGASRTAITTL.
QWY16164.1    LEYGACPNNMMGTFSVRTVGTSK.SKYPLVVRIYMRMKHVRAWIPRPMRNQNYLFKANPNYAGNSIKPTGASRTAITTL.
QWY16163.1    LEYGACPNNMMGTFSVRTVGTSK.SKYPLVVRIYMRMKHVRAWIPRPMRNQNYLFKANPNYAGNSIKPTGASRTAITTL.
QWY16157.1    LEYGACPNNMMGTFSVRTVGTSK.SKYPLVVRIYMRMKHVRAWIPRPMRNQNYLFKANPNYAGNSIKPTGASRTAITTL.
QWY16150.1    LEYGACPNNMMGTFSVRTVGTSK.SKYPLVVRIYMRMKHVRAWIPRPMRNQNYLFKANPNYAGNSIKPTGASRTAITTL.
QWY16115.1    LEYGACPNNMMGTFSVRTVGTSK.SKYPLVVRIYMRMKHVRAWIPRPMRNQNYLFKANPNYAGNSIKPTGASRTAITTL.
QWY16087.1    LEYGACPNNMMGTFSVRTVGTSK.SKYPLVVRIYMRMKHVRAWIPRPMRNQNYLFKANPNYAGNSIKPTGASRTAITTL.
QWY16069.1    LEYGACPNNMMGTFSVRTVGTSK.SKYPLVVRIYMRMKHVRAWIPRPMRNQNYLFKANPNYAGNSIKPTGASRTAITTL.
QWY16058.1    LEYGACPNNMMGTFSVRTVGTSK.SKYPLVVRIYMRMKHVRAWIPRPMRNQNYLFKANPNYAGNSIKPTGASRTAITTL.
QWY16048.1    LEYGACPNNMMGTFSVRTVGTSK.SKYPLVVRIYMRMKHVRAWIPRPMRNQNYLFKANPNYAGNSIKPTGASRTAITTL.
QWY16047.1    LEYGACPNNMMGTFSVRTVGTSK.SKYPLVVRIYMRMKHVRAWIPRPMRNQNYLFKANPNYAGNSIKPTGASRTAITTL.
QWY16030.1    LEYGACPNNMMGTFSVRTVGTSK.SKYPLVVRIYMRMKHVRAWIPRPMRNQNYLFKANPNYAGNSIKPTGASRTAITTL.
QWY16027.1    LEYGACPNNMMGTFSVRTVGTSK.SKYPLVVRIYMRMKHVRAWIPRPMRNQNYLFKANPNYAGNSIKPTGASRTAITTL.
QWY16021.1    LEYGACPNNMMGTFSVRTVGTSK.SKYPLVVRIYMRMKHVRAWIPRPMRNQNYLFKANPNYAGNSIKPTGASRTAITTL.
QWY16020.1    LEYGACPNNMMGTFSVRTVGTSK.SKYPLVVRIYMRMKHVRAWIPRPMRNQNYLFKANPNYAGNSIKPTGASRTAITTL.
QWY16009.1    LEYGACPNNMMGTFSVRTVGTSK.SKYPLVVRIYMRMKHVRAWIPRPMRNQNYLFKANPNYAGNSIKPTGASRTAITTL.
QWY16008.1    LEYGACPNNMMGTFSVRTVGTSK.SKYPLVVRIYMRMKHVRAWIPRPMRNQNYLFKANPNYAGNSIKPTGASRTAITTL.
QWY16007.1    LEYGACPNNMMGTFSVRTVGTSK.SKYPLVVRIYMRMKHVRAWIPRPMRNQNYLFKANPNYAGNSIKPTGASRTAITTL.
QWY16001.1    LEYGACPNNMMGTFSVRTVGTSK.SKYPLVVRIYMRMKHVRAWIPRPMRNQNYLFKANPNYAGNSIKPTGASRTAITTL.
QWY16000.1    LEYGACPNNMMGTFSVRTVGTSK.SKYPLVVRIYMRMKHVRAWIPRPMRNQNYLFKANPNYAGNSIKPTGASRTAITTL.
QWY15993.1    LEYGACPNNMMGTFSVRTVGTSK.SKYPLVVRIYMRMKHVRAWIPRPMRNQNYLFKANPNYAGNSIKPTGASRTAITTL.
QWY15986.1    LEYGACPNNMMGTFSVRTVGTSK.SKYPLVVRIYMRMKHVRAWIPRPMRNQNYLFKANPNYAGNSIKPTGASRTAITTL.
QWY15979.1    LEYGACPNNMMGTFSVRTVGTSK.SKYPLVVRIYMRMKHVRAWIPRPMRNQNYLFKANPNYAGNSIKPTGASRTAITTL.
QTA73889.1    LEYGACPNNMMGTFSVRTVGTSK.SKYPLVVRIYMRMKHVRAWIPRPMRNQNYLFKANPNYAGNSIKPTGASRTAITTL.
QTA73874.1    LEYGACPNNMMGTFSVRTVGTSK.SKYPLVVRIYMRMKHVRAWIPRPMRNQNYLFKANPNYAGNSIKPTGASRTAITTL.
QTA73871.1    LEYGACPNNMMGTFSVRTVGTSK.SKYPLVVRIYMRMKHVRAWIPRPMRNQNYLFKANPNYAGNSIKPTGASRTAITTL.
QOP74901.1    LEYGACPNNMMGTFSVRTVGTSK.SKYPLVVRIYMRMKHVRAWIPRPMRNQNYLFKANPNYAGNSIKPTGASRTAITTL.
QOP74900.1    LEYGACPNNMMGTFSVRTVGTSK.SKYPLVVRIYMRMKHVRAWIPRPMRNQNYLFKANPNYAGNSIKPTGASRTAITTL.
QOP74897.1    LEYGACPNNMMGTFSVRTVGTSK.SKYPLVVRIYMRMKHVRAWIPRPMRNQNYLFKANPNYAGNSIKPTGASRTAITTL.
QOP74893.1    LEYGACPNNMMGTFSVRTVGTSK.SKYPLVVRIYMRMKHVRAWIPRPMRNQNYLFKANPNYAGNSIKPTGASRTAITTL.
QOP74887.1    LEYGACPNNMMGTFSVRTVGTSK.SKYPLVVRIYMRMKHVRAWIPRPMRNQNYLFKANPNYAGNSIKPTGASRTAITTL.
QOP74886.1    LEYGACPNNMMGTFSVRTVGTSK.SKYPLVVRIYMRMKHVRAWIPRPMRNQNYLFKANPNYAGNSIKPTGASRTAITTL.
QOP74884.1    LEYGACPNNMMGTFSVRTVGTSK.SKYPLVVRIYMRMKHVRAWIPRPMRNQNYLFKANPNYAGNSIKPTGASRTAITTL.
QOP74882.1    LEYGACPNNMMGTFSVRTVGTSK.SKYPLVVRIYMRMKHVRAWIPRPMRNQNYLFKANPNYAGNSIKPTGASRTAITTL.
QOP74881.1    LEYGACPNNMMGTFSVRTVGTSK.SKYPLVVRIYMRMKHVRAWIPRPMRNQNYLFKANPNYAGNSIKPTGASRTAITTL.
QOP74880.1    LEYGACPNNMMGTFSVRTVGTSK.SKYPLVVRIYMRMKHVRAWIPRPMRNQNYLFKANPNYAGNSIKPTGASRTAITTL.
QOP74879.1    LEYGACPNNMMGTFSVRTVGTSK.SKYPLVVRIYMRMKHVRAWIPRPMRNQNYLFKANPNYAGNSIKPTGASRTAITTL.
QOP74878.1    LEYGACPNNMMGTFSVRTVGTSK.SKYPLVVRIYMRMKHVRAWIPRPMRNQNYLFKANPNYAGNSIKPTGASRTAITTL.
pdb|4YVS|M    LEYGACPNNMMGTFSVRTVGTSK.SKYPLVVRIYMRMKHVRAWIPRPMRNQNYLFKANPNYAGNSIKPTGASRTAITTL.
pdb|4YVS|J    LEYGACPNNMMGTFSVRTVGTSK.SKYPLVVRIYMRMKHVRAWIPRPMRNQNYLFKANPNYAGNSIKPTGASRTAITTL.
pdb|4YVS|G    LEYGACPNNMMGTFSVRTVGTSK.SKYPLVVRIYMRMKHVRAWIPRPMRNQNYLFKANPNYAGNSIKPTGASRTAITTL.
pdb|4YVS|D    LEYGACPNNMMGTFSVRTVGTSK.SKYPLVVRIYMRMKHVRAWIPRPMRNQNYLFKANPNYAGNSIKPTGASRTAITTL.
pdb|4YVS|A    LEYGACPNNMMGTFSVRTVGTSK.SKYPLVVRIYMRMKHVRAWIPRPMRNQNYLFKANPNYAGNSIKPTGASRTAITTL.
pdb|4CEY|A    LEYGACPNNMMGTFSVRTVGTSK.SKYPLVVRIYMRMKHVRAWIPRPMRNQNYLFKANPNYAGNSIKPTGASRTAITTL.
pdb|4CEW|A    LEYGACPNNMMGTFSVRTVGTSK.SKYPLVVRIYMRMKHVRAWIPRPMRNQNYLFKANPNYAGNSIKPTGASRTAITTL.
pdb|4N53|A    LEYGACPNNMMGTFSVRTVGTSK.SKYPLVVRIYMRMKHVRAWIPRPMRNQNYLFKANPNYAGNSIKPTGASRTAITTL.
pdb|4N43|A    LEYGACPNNMMGTFSVRTVGTSK.SKYPLVVRIYMRMKHVRAWIPRPMRNQNYLFKANPNYAGNSIKPTGASRTAITTL.
pdb|4RQP|A    LEYGACPNNMMGTFSVRTVGTSK.SKYPLVVRIYMRMKHVRAWIPRPMRNQNYLFKANPNYAGNSIKPTGASRTAITTL.
pdb|4RQP|M    LEYGACPNNMMGTFSVRTVGTSK.SKYPLVVRIYMRMKHVRAWIPRPMRNQNYLFKANPNYAGNSIKPTGASRTAITTL.
pdb|4RQP|I    LEYGACPNNMMGTFSVRTVGTSK.SKYPLVVRIYMRMKHVRAWIPRPMRNQNYLFKANPNYAGNSIKPTGASRTAITTL.
pdb|4RQP|Q    LEYGACPNNMMGTFSVRTVGTSK.SKYPLVVRIYMRMKHVRAWIPRPMRNQNYLFKANPNYAGNSIKPTGASRTAITTL.
pdb|4RQP|E    LEYGACPNNMMGTFSVRTVGTSK.SKYPLVVRIYMRMKHVRAWIPRPMRNQNYLFKANPNYAGNSIKPTGASRTAITTL.
QWY16033.1    LEYGACPNNMMGTFSVRTVGTSK.SKYPLVVRIYMRMKHVRAWIPRPMRNQNYLFKANPNYAGNSIKPTGASRTAITTL.
QWY16029.1    LEYGACPNNMMGTFSVRTVGTSK.SKYPLVVRIYMRMKHVRAWIPRPMRNQNYLFKANPNYAGNSIKPTGASRTAITTL.
QWY16028.1    LEYGACPNNMMGTFSVRTVGTSK.SKYPLVVRIYMRMKHVRAWIPRPMRNQNYLFKANPNYAGNSIKPTGASRTAITTL.
QWY16025.1    LEYGACPNNMMGTFSVRTVGTSK.SKYPLVVRIYMRMKHVRAWIPRPMRNQNYLFKANPNYAGNSIKPTGASRTAITTL.
QUS47943.1    LEYGACPNNMMGTFSVRTVGTSK.SKYPLVVRIYMRMKHVRAWIPRPMRNQNYLFKANPNYAGNSIKPTGASRTAITTL.
QOP74914.1    LEYGACPNNMMGTFSVRTVGASK.SKYPLVVRIYMRMKHVRAWIPRPMRNQNYLFKANPNYAGNSIKPTGASRTAITTL.
QUS47916.1    LEYGACPNNMMGTFSVRTVGTSK.SKYPLVVRIYMRMKHVRAWIPRPMRNQNYLFKANPNYAGNTIKPTGASRTAITTL.
QWY16148.1    LEYGACPNNMMGTFSVRTVGTSK.SKYPLVVRIYMRMKHVRAWIPRPMRNQNYLFKANPNYAGNTIKPTGASRTAITTL.
QOP74906.1    LEYGACPNNMMGTFSVRTVGTSK.SKYPLVVRIYMRMKHVRAWIPRPMRNQNYLFKANPNYAGNTIKPTGASRTAITTL.
QOP74895.1    LEYGACPNNMMGTFSVRTVGTSK.SKYPLVVRIYMRMKHVRAWIPRPMRNQNYLFKANPNYAGNTIKPTGASRTAITTL.
QOP74890.1    LEYGACPNNMMGTFSVRTVGTSK.SKYPLVVRIYMRMKHVRAWIPRPMRNQNYLFKANPNYAGNTIKPTGASRTAITTL.
QOP74889.1    LEYGACPNNMMGTFSVRTVGTSK.SKYPLVVRIYMRMKHVRAWIPRPMRNQNYLFKANPNYAGNTIKPTGASRTAITTL.
QWY16149.1    LEYGACPNNMMGTFSVRTVGTSK.SKYPLVVRIYMRMKHVRAWIPRPMRNQNYLFKANPNYAGNTIKPTGASRTAITTL.
QOP74888.1    LEYGACPNNMMGTFSVRTVGTSK.SKYPLVVRIYMRMKHVRAWIPRPMRNQNYLFKANPNYAGNTIKPTGASRTAITTL.
QOP74894.1    LEYGACPNNMMGTFSVRTVGISK.SKYPLVVRIYMRMKHVRAWIPRPMRNQNYLFKANPNYAGNTIKPTGASRTAITTL.
QUS47930.1    LEYGACPNNMMGTFSVRTVGTSK.SKYPLVVRIYMRMKHVRAWIPRPMRNQNYLFKANPNYAGNSIKPTGASRTAITTL.
QWY16053.1    LEYGACPNNMMGTFSVRTVGTSK.SKYPLVVRIYMRMKHVRAWIPRPMRNQNYLFKANPNYAGNSIKPTGASRTAITTL.
QWY15997.1    LEYGACPNNMMGTFSVRTVGTSK.SKYPLVVRIYMRMKHVRAWIPRPMRNQNYLFKANPNYAGNSIKPTGASRTAITTL.
QWY15996.1    LEYGACPNNMMGTFSVRTVGTSK.SKYPLVVRIYMRMKHVRAWIPRPMRNQNYLFKANPNYAGNSIKPTGASRTAITTL.
QWY15995.1    LEYGACPNNMMGTFSVRTVGTSK.SKYPLVVRIYMRMKHVRAWIPRPMRNQNYLFKANPNYAGNSIKPTGASRTAITTL.
QTA73878.1    LEYGACPNNMMGTFSVRTVGTSK.SKYPLVVRIYMRMKHVRAWIPRPMRNQNYLFKANPNYAGNSIKPTGASRTAITTL.
QTA73877.1    LEYGACPNNMMGTFSVRTVGTSK.SKYPLVVRIYMRMKHVRAWIPRPMRNQNYLFKANPNYAGNSIKPTGASRTAITTL.
QTA73876.1    LEYGACPNNMMGTFSVRTVGTSK.SKYPLVVRIYMRMKHVRAWIPRPMRNQNYLFKANPNYAGNSIKPTGASRTAITTL.
QUS47914.1    LEYGACPNNMMGTFSVRTVGTSK.SKYPLVVRIYMRMKHVRAWIPRPMRNQNYLFKANPNYAGNSIKPTGASRTAITTL.
QWY16003.1    LEYGACPNNMMGTFSVRTVGTSK.SKYPLVVRIYMRMKHVRAWIPRPMRNQNYLFKANPNYAGNSIKPTGASRTAITTL.
QWY16067.1    LEYGACPNNMMGTFSVRTVGTSK.SKYPLVVRIYMRMKHVRAWIPRPMRNQNYLFKANPNYAGNSIKPTGASRTAITTL.
QWY16057.1    LEYGACPNNMMGTFSVRTVGTSK.SKYPLVVRIYMRMKHVRAWIPRPMRNQNYLFKANPNYAGNSIKPTGASRTAITTL.
QTA73888.1    LEYGACPNNMMGTFSVRTVGTSK.SKYPLVVRIYMRMKHVRAWIPRPMRNQNYLFKANPNYAGNSIKPTGASRTAITTL.
QWY16086.1    LEYGACPNNMMGTFSVRTVGTSK.SKYPLVVRIYMRMKHVRAWIPRPMRNQNYLFKANPNYAGNSIKPTGASRTAITTL.
pdb|4GMP|1    LEYGACPNNMMGTFSVRTVGTSK.SKYPLVVRIYMRMKHVRAWIPRPMRNQNYLFKANPNYAGNSIKPTGASRTAITTL.
pdb|3J91|1    LEYGACPNNMMGTFSVRTVGTSK.SKYPLVVRIYMRMKHVRAWIPRPMRNQNYLFKANPNYAGNSIKPTGASRTAITTL.
QWY16094.1    LEYGACPNNMMGTFSVRTVGTSK.SKYPLVVRIYMRMKHVRAWIPRPMRNQNYLFKANPNYAGNSIKPTGASRTAITTL.
QWY16093.1    LEYGACPNNMMGTFSVRTVGTSK.SKYPLVVRIYMRMKHVRAWIPRPMRNQNYLFKANPNYAGNSIKPTGASRTAITTL.
QWY16091.1    LEYGACPNNMMGTFSVRTVGTSK.SKYPLVVRIYMRMKHVRAWIPRPMRNQNYLFKANPNYAGNSIKPTGASRTAITTL.
QWY16088.1    LEYGACPNNMMGTFSVRTVGTSK.SKYPLVVRIYMRMKHVRAWIPRPMRNQNYLFKANPNYAGNSIKPTGASRTAITTL.
QWY16082.1    LEYGACPNNMMGTFSVRTVGTSK.SKYPLVVRIYMRMKHVRAWIPRPMRNQNYLFKANPNYAGNSIKPTGASRTAITTL.
QWY16073.1    LEYGACPNNMMGTFSVRTVGTSK.SKYPLVVRIYMRMKHVRAWIPRPMRNQNYLFKANPNYAGNSIKPTGASRTAITTL.
QWY16055.1    LEYGACPNNMMGTFSVRTVGTSK.SKYPLVVRIYMRMKHVRAWIPRPMRNQNYLFKANPNYAGNSIKPTGASRTAITTL.
QUS47918.1    LEYGACPNNMMGTFSVRTVGTSK.SKYPLVVRIYMRMKHVRAWIPRPMRNQNYLFKANPNYAGNSIKPTGASRTAITTL.
QWY16089.1    LEYGACPNNMMGTFSVRTVGTSK.SKYPLVVRIYMRMKHVRAWIPRPMRNQNYLFKANPNYAGNSIKPTGASRTAITTL.
QWY16037.1    LEYGACPNNMMGTFSVRTVGTSK.SKYPLVVRIYMRMKHVRAWIPRPMRNQNYLFKANPNYAGNSIKPTGASRTAITTL.
QOP74891.1    LEYGACPNNMMGTFSVRTVGTSK.SKYPLVVRIYMRMKHVRAWIPRPMRNQNYLFKANPNYAGNSIKPTGASRTAITTL.
pdb|3VBS|A    LEYGAMPNNMMGTFSVRTVGTSK.SKYPLVVRIYMRMKHVRAWIPRPMRNQNYLFKANPNYAGNSIKPTGASRTAITTL.
pdb|3VBO|A    LEYGAMPNNMMGTFSVRTVGTSK.SKYPLVVRIYMRMKHVRAWIPRPMRNQNYLFKANPNYAGNSIKPTGASRTAITTL.
pdb|3VBH|A    LEYGAMPNNMMGTFSVRTVGTSK.SKYPLVVRIYMRMKHVRAWIPRPMRNQNYLFKANPNYAGNSIKPTGASRTAITTL.
pdb|3VBF|A    LEYGAMPNNMMGTFSVRTVGTSK.SKYPLVVRIYMRMKHVRAWIPRPMRNQNYLFKANPNYAGNSIKPTGASRTAITTL.
pdb|6LQD|A    LEYGAMPNNMMGTFSVRTVGTSK.SKYPLVVRIYMRMKHVRAWIPRPMRNQNYLFKANPNYAGNSIKPTGASRTAITTL.
pdb|5ZUF|A    LEYGAMPNNMMGTFSVRTVGTSK.SKYPLVVRIYMRMKHVRAWIPRPMRNQNYLFKANPNYAGNSIKPTGASRTAITTL.
pdb|5ZUD|A    LEYGAMPNNMMGTFSVRTVGTSK.SKYPLVVRIYMRMKHVRAWIPRPMRNQNYLFKANPNYAGNSIKPTGASRTAITTL.
pdb|4CDX|A    LEYGACPNNMMGTFSVRTVGTSK.SKYPLVVRIYMRMKHVRAWIPRPMRNQNYLFKANPNYAGNSIKPTGASRTAITTL.
pdb|4CDW|A    LEYGACPNNMMGTFSVRTVGTSK.SKYPLVVRIYMRMKHVRAWIPRPMRNQNYLFKANPNYAGNSIKPTGASRTAITTL.
pdb|4CDU|A    LEYGACPNNMMGTFSVRTVGTSK.SKYPLVVRIYMRMKHVRAWIPRPMRNQNYLFKANPNYAGNSIKPTGASRTAITTL.
pdb|4CDQ|A    LEYGACPNNMMGTFSVRTVGTSK.SKYPLVVRIYMRMKHVRAWIPRPMRNQNYLFKANPNYAGNSIKPTGASRTAITTL.
```

```
QOP74885.1   LEYGACPNNMMGTFSVRTVGTSK.SKYPLVVRIYMRMKHVRAWIPRPMRNQNYLFKANPNYAGNSIKPTGASRTAITTL.
QWY16026.1   LEYGACPNNMMGTFSVRTVGTSK.SKYPLVVRIYMRMKHVRAWIPRPMRNQNYLFKANPNYAGNSIKPTGASRTAITTL.
QWY15977.1   LEYGACPNNMMGTFSVRTVGTSK.SKYPLVIRIYMRMKHVRAWIPRPMRNQNYLFKANPNYAGNSIKPTGASRTAITTL.
QTA73869.1   LEYGACPNNMMGTFSVRTVGTSK.SKYPLVIRIYMRMKHVRAWIPRPMRNQNYLFKANPNYAGNSIKPTGASRTAITTL.
QUS47935.1   LEYGACPNNMMGTFSVRTVGTSK.SKYPLVIRIYMRMKHVRAWIPRPMRNQNYLFKANPNYAGNSIKPTGASRTAITTL.
QWY16024.1   LEYGACPNNMMGTFSVRTVGTSK.SKYPLVIRIYMRMKHVRAWIPRPMRNQNYLFKANPNYAGNSIKPTGASRTAITTL.
QWY16023.1   LEYGACPNNMMGTFSVRTVGTSK.SKYPLVIRIYMRMKHVRAWIPRPMRNQNYLFKANPNYAGNSIKPTGASRTAITTL.
QWY16022.1   LEYGACPNNMMGTFSVRTVGTSK.SKYPLVIRIYMRMKHVRAWIPRPMRNQNYLFKANPNYAGNSIKPTGASRTAITTL.
QWY16013.1   LEYGACPNNMMGTFSVRTVGTSK.SKYPLVIRIYMRMKHVRAWIPRPMRNQNYLFKANPNYAGNSIKPTGASRTAITTL.
QWY16012.1   LEYGACPNNMMGTFSVRTVGTSK.SKYPLVIRIYMRMKHVRAWIPRPMRNQNYLFKANPNYAGNSIKPTGASRTAITTL.
QWY15992.1   LEYGACPNNMMGTFSVRTVGTSK.SKYPLVIRIYMRMKHVRAWIPRPMRNQNYLFKANPNYAGNSIKPTGASRTAITTL.
QWY15982.1   LEYGACPNNMMGTFSVRTVGTSK.SKYPLVIRIYMRMKHVRAWIPRPMRNQNYLFKANPNYAGNSIKPTGASRTAITTL.
QWY15981.1   LEYGACPNNMMGTFSVRTVGTSK.SKYPLVIRIYMRMKHVRAWIPRPMRNQNYLFKANPNYAGNSIKPTGASRTAITTL.
QWY15976.1   LEYGACPNNMMGTFSVRTVGTSK.SKYPLVIRIYMRMKHVRAWIPRPMRNQNYLFKANPNYAGNSIKPTGASRTAITTL.
QTA73880.1   LEYGACPNNMMGTFSVRTVGTSK.SKYPLVIRIYMRMKHVRAWIPRPMRNQNYLFKANPNYAGNSIKPTGASRTAITTL.
QTA73872.1   LEYGACPNNMMGTFSVRTVGTSK.SKYPLVIRIYMRMKHVRAWIPRPMRNQNYLFKANPNYAGNSIKPTGASRTAITTL.
QTA73868.1   LEYGACPNNMMGTFSVRTVGTSK.SKYPLVIRIYMRMKHVRAWIPRPMRNQNYLFKANPNYAGNSIKPTGASRTAITTL.
QOP74892.1   LEYGACPNNMMGTFSVRTVGTSK.SKYPLVIRIYMRMKHVRAWIPRPMRNQNYLFKANPNYAGNSIKPTGASRTAITTL.
QWY15998.1   LEYGACPNNMMGTFSVRTVGTSK.SKYPLVIRIYMRMKHVRAWIPRPMRNQNYLFKANPNYAGNSIKPTGASRTAITTL.
QWY15994.1   LEYGACPNNMMGTFSVRTVGTSK.SKYPLVIRIYMRMKHVRAWIPRPMRNQNYLFKANPNYAGNSIKPTGASRTAITTL.
QTA73879.1   LEYGACPNNMMGTFSVRTVGTSK.SKYPLVIRIYMRMKHVRAWIPRPMRNQNYLFKANPNYAGNSIKPTGASRTAITTL.
QTA73875.1   LEYGACPNNMMGTFSVRTVGTSK.SKYPLVIRIYMRMKHVRAWIPRPMRNQNYLFKANPNYAGNSIKPTGASRTAITTL.
QWY16006.1   LEYGACPNNMMGTFSVRTVGTSK.SKYPLVIRIYMRMKHVRAWIPRPVRNQNYLFKANPNYAGNSIKPTGASRTAITTL.
QWY16005.1   LEYGACPNNMMGTFSVRTVGTSK.SKYPLVIRIYMRMKHVRAWIPRPVRNQNYLFKANPNYAGNSIKPTGASRTAITTL.
QWY16039.1   LEYGACPNNMMGTFSVRTVGTSK.SKYPLVIRIYMRMKHVRAWIPRPMRNQNYLFKANPNYAGNSIKPTGASRTAITTL.
QWY15999.1   LEYGACPNNMMGTFSVRTVGTSK.SKYPLVIRIYMRMKHVRAWIPRPMRNQNYLFKANPNYAGNSIKPTGASRTSITTL.
QWY16004.1   LEYGACPNNMMGTFSVRTVGTSK.SKYPLVVRIYMRMKHVRAWIPRPMRNQNYLFKANPNYAGNSIKPTGASRTAITTL.
QOP74913.1   LEYGACPNNMMGTFSVRTVGTSK.SKYPLVVRIYMRMKHVRAWIPRPMRNQNYLFKANPNYAGNSIKPTGASRTAITTL.
QOP74915.1   LEYGACPNNMMGTFSVRTVGTSK.SKYPLVVRIYMRMKHVRAWIPRPMRNQNYLFKANPNYAGNSIKPTGASRTAITTL.
QWY16035.1   LEYGACPNNMMGTFSVRTVGTSK.SKYPLVVRIYMRMKHVRAWIPRPMRNQNYLFKANPNYAGNSIKPTGASRTAITTL.
QWY15990.1   LEYGACPNNMMGTFSVRTVGTSK.SKYPLVVRIYMRMKHVRAWIPRPMRNQNYLFKANPNYAGNSIKPTGASRTVITTL.
QWY15988.1   LEYGACPNNMMGTFSVRTVGTSK.SKYPLVVRIYMRMKHVRAWIPRPMRNQNYLFKANPNYAGNSIKPTGASRTVITTL.
QWY15989.1   LEYGACPNNMMGTFSVRTVGTSK.SKYPLVVRIYMRMKHVRAWIPRPMRNQNYLFKANPNYAGNSIKPTGASRTVITTL.
QWY15987.1   LEYGACPNNMMGTFSVRTVGTSK.SKYPLVVRIYMRMKHVRAWIPRPMRNQNYLFKANPNYAGNSIKPTGASRTVITTL.
QWY15978.1   LEYGACPNNMMGTFSVRTVGTSK.SKYPLVVRIYMRMKHVRAWIPRPMRNQNYLFKANPNYAGNSIKPTGASRTVITTL.
QTA73870.1   LEYGACPNNMMGTFSVRTVGTSK.SKYPLVVRIYMRMKHVRAWIPRPMRNQNYLFKANPNYAGNSIKPTGASRTVITTL.
QWY15983.1   LEYGACPNNMMGTFSVRTVGTSK.SKYPLVIRIYMRMKHVRAWIPRPMRNQNYLFKANPNYAGNSIKPTGASRTVITTL.
QUS47931.1   LEYGACPNNMMGTFSVRTVGTSK.SKYPLVIRIYMRMKHVRAWIPRPMRNQNYLFKANPNYAGNSIKPTGASRTAITTL.
QUS47925.1   LEYGACPNNMMGTFSVRTVGTSK.SKYPLVIRIYMRMKHVRAWIPRPMRNQNYLFKANPNYAGNSIKPTGASRTAITTL.
QUS47922.1   LEYGACPNNMMGTFSVRTVGTSK.SKYPLVIRIYMRMKHVRAWIPRPMRNQNYLFKANPNYAGNSIKPTGASRTAITTL.
QWY16014.1   LEYGACPNNMMGTFSVRTVGTSK.SKYPLVIRIYMRMKHVRAWIPRPMRNQNYLFKANPNYAGNSIKPTGASRTAITTL.
QTA73881.1   LEYGACPNNMMGTFSVRTVGTSK.SKYPLVIRIYMRMKHVRAWIPRPMRNQNYLFKANPNYAGNSIKPTGASRTAITTL.
QOP74898.1   LEYGACPNNMMGTFSVRTVGTSK.SKYPLVIRIYMRMKHVRAWIPRPMRNQNYLFKANPNYAGNSIKPTGASRTAITTL.
QWY15980.1   LEYGACPNNMMGTFSVRTVGTSK.SKYPLVIRIYMRMKHVRAWIPRPMRNQNYLFKANPNYAGNSIKPTGASRTAITTL.
QTA73873.1   LEYGACPNNMMGTFSVRTVGTSK.SKYPLVIRIYMRMKHVRAWIPRPMRNQNYLFKANPNYAGNSIKPTGASRTAITTL.
QWY16011.1   LEYGACPNNMMGTFSVRTVGTSK.SKYPLVIRIYMRMKHVRAWIPRPMRNQNYLFKANPNYAGNSIKPTGASRKAITTL.
QWY16010.1   LEYGACPNNMMGTFSVRTVGTSK.SKYPLVIRIYMRMKHVRAWIPRPMRNQNYLFKANPNYAGNSIKPTGASRKAITTL.
QOP74911.1   LEYGACPNNMMGTFSVRTVGTSK.SKYPLVVRIYMRMKHVRAWIPRPMRNQNYLFKANPNYAGNSIKPTGASRTAITTL.
QWY16019.1   LEYGACPNNMMGTFSVRTVGTSK.SKYPLVIRIYMRMKHVRAWIPRPMRNQNYLFKANPNYAGNSIKPTGASRTAITTL.
QOP74883.1   LEYGACPNNMMGTFSVRTVGTSK.SKYPLVVRIYMRMKHVRAWIPRPMRNQNYLFKANPNYAGNSIKPTGTSRTAITTL.
QUS47909.1   LEYGACPNNMMGTFSVRTVGTSK.SKYPLVVRIYMRMKHVRAWIPRPMRNQNYLFKANPNYAGSSIKPTGTSRTAITTL.
QWY16144.1   LEYGACPNNMMGTFSVRTVGTSK.SKYPLVVRIYMRMKHVRAWIPRPMRNQNYLFKANPNYAGSSIKPTGTSRTAITTL.
QUS47908.1   LEYGACPNNMMGTFSVRTVGTSK.SKYPLVVRIYMRMKHVRAWIPRPMRNQNYLFKANPNYAGNSIKPTGTSRTAITTL.
QNN25844.1   LEYGACPNNMMGTFSVRTVGTSK.SKYPLVIRIYMRMKHVRAWVPRPMRNQNYLFKANPNYAGNSIKPTGTSRTAITTL.
QNN25840.1   LEYGACPNNMMGTFSVRTVGTSK.SKYPLVIRIYMRMKHVRAWVPRPMRNQNYLFKANPNYAGNSIKPTGTSRTAITTL.
QNN25839.1   LEYGACPNNMMGTFSVRTVGTSK.SKYPLVIRIYMRMKHVRAWVPRPMRNQNYLFKANPNYAGNSIKPTGTSRTAITTL.
QNN25835.1   LEYGACPNNMMGTFSVRTVGTSK.SKYPLVIRIYMRMKHVRAWVPRPMRNQNYLFKANPNYAGNSIKPTGTSRTAITTL.
QNN25834.1   LEYGACPNNMMGTFSVRTVGTSK.SKYPLVIRIYMRMKHVRAWVPRPMRNQNYLFKANPNYAGNSIKPTGTSRTAITTL.
QOP74912.1   LEYGACPNNMMGTFSVRTVGTSK.SKYPLVVRIYMRMKHVRAWIPRPMRNQNYLFKANPNYAGNSIKPTGTSRTAITTL.
QUS47910.1   LEYGACPNNMMGTFSVRTVGTSK.SKYPLVVRIYMRMKHVRAWIPRPMRNQNYLFKANPNYAGNSIKPTGASRTAITTL.
QUS47907.1   LEYGACPNNMMGTFSVRTVGTSK.SKYPLVVRIYMRMKHVRAWIPRPMRNQNYLFKANPNYAGNSIKPTGTSRTAITTL.
QWY16141.1   LEYGACPNNMMGTFSVRTVGTSK.SKYPLVVRIYMRMKHVRAWIPRPMRNQNYLFKANPNYAGNTIKPTGASRTSITTL.
QWY16140.1   LEYGACPNNMMGTFSVRTVGTSK.SKYPLVVRIYMRMKHVRAWIPRPMRNQNYLFKANPNYAGNTIKPTGASRTSITTL.
QUS47937.1   LEYGACPNNMMGTFSVRTVGTSK.SKYPLVVRIYMRMKHVRAWIPRPMRNQNYLFKANPNYAGNTIKPTGTSRTSITTL.
QUS47936.1   LEYGACPNNMMGTFSVRTVGTSK.SKYPIVVRIYMRMKHVRAWIPRPMRNQNYLFKANPNYAGNTIKPTGTSRTSITTL.
QUS47912.1   LEYGACPNNMMGTFSVRTVGTSK.SKYPLVVRIYMRMKHVRAWIPRPMRNQNYLFKANPNYAGNSIKPTGASRTAITTL.
QUS47906.1   LEYGACPNNMMGTFSVRTVGTSK.SKYPLVIRIYMRMKHVRAWIPRPMRNQNYLFKANPNYAGNSIKPTGASRTAITTL.
pdb|4YVW|M   LDYGACPNNMMGTFSVRTVGTSK.SKYPLVVRIYMRMKHVRAWIPRPMRNQNYLFKANPNYAGNSIKPTGASRTAITTL.
pdb|4YVW|J   LDYGACPNNMMGTFSVRTVGTSK.SKYPLVVRIYMRMKHVRAWIPRPMRNQNYLFKANPNYAGNSIKPTGASRTAITTL.
pdb|4YVW|D   LDYGACPNNMMGTFSVRTVGTSK.SKYPLVVRIYMRMKHVRAWIPRPMRNQNYLFKANPNYAGNSIKPTGASRTAITTL.
pdb|4YVW|A   LDYGACPNNMMGTFSVRTVGTSK.SKYPLVVRIYMRMKHVRAWIPRPMRNQNYLFKANPNYAGNSIKPTGASRTAITTL.
pdb|4YVW|E   LDYGACPNNMMGTFSVRTVGTSK.SKYPLVVRIYMRMKHVRAWIPRPMRNQNYLFKANPNYAGNSIKPTGASRTAITTL.
QPI19797.1   LEYGACPNNMMGTFSVRTVGSSK.SKYPLVVRIYMRMKHVRAWIPRPMRNQNYLFKANPNYAGNSIKPTGTSRTAITTL.
QPI19781.1   LEYGACPNNMMGTFSVRTVGSSK.SKYPLVVRIYMRMKHVRAWIPRPMRNQNYLFKANPNYAGNSIKPTGTSRTAITTL.
QPI19795.1   LEYGACPNNMMGTFSVRTVGSSK.SKYPLVVRIYMRMKHVRAWIPRPMRNQNYLFKANPNYAGNSIKPTGTSRTAITTL.
QPI19794.1   LEYGACPNNMMGTFSVRTVGSSK.SKYPLVVRIYMRMKHVRAWIPRPMRNQNYLFKANPNYAGNSIKPTGTSRTAITTL.
QPI19793.1   LEYGACPNNMMGTFSVRTVGSSK.SKYPLVVRIYMRMKHVRAWIPRPMRNQNYLFKANPNYAGNSIKPTGTSRTAITTL.
QPI19792.1   LEYGACPNNMMGTFSVRTVGSSK.SKYPLVVRIYMRMKHVRAWIPRPMRNQNYLFKANPNYAGNSIKPTGTSRTAITTL.
QPI19790.1   LEYGACPNNMMGTFSVRTVGSSK.SKYPLVVRIYMRMKHVRAWIPRPMRNQNYLFKANPNYAGNSIKPTGTSRTAITTL.
QPI19789.1   LEYGACPNNMMGTFSVRTVGSSK.SKYPLVVRIYMRMKHVRAWIPRPMRNQNYLFKANPNYAGNSIKPTGTSRTAITTL.
QPI19788.1   LEYGACPNNMMGTFSVRTVGSSK.SKYPLVVRIYMRMKHVRAWIPRPMRNQNYLFKANPNYAGNSIKPTGTSRTAITTL.
QPI19787.1   LEYGACPNNMMGTFSVRTVGSSK.SKYPLVVRIYMRMKHVRAWIPRPMRNQNYLFKANPNYAGNSIKPTGTSRTAITTL.
QPI19785.1   LEYGACPNNMMGTFSVRTVGSSK.SKYPLVVRIYMRMKHVRAWIPRPMRNQNYLFKANPNYAGNSIKPTGTSRTAITTL.
QPI19784.1   LEYGACPNNMMGTFSVRTVGSSK.SKYPLVVRIYMRMKHVRAWIPRPMRNQNYLFKANPNYAGNSIKPTGTSRTAITTL.
QPI19782.1   LEYGACPNNMMGTFSVRTVGSSK.SKYPLVVRIYMRMKHVRAWIPRPMRNQNYLFKANPNYAGNSIKPTGTSRTAITTL.
QPI19780.1   LEYGACPNNMMGTFSVRTVGSSK.SKYPLVVRIYMRMKHVRAWIPRPMRNQNYLFKANPNYAGNSIKPTGTSRTAITTL.
QPI19777.1   LEYGACPNNMMGTFSVRTVGSSK.SKYPLVVRIYMRMKHVRAWIPRPMRNQNYLFKANPNYAGNSIKPTGTSRTAITTL.
QPI19762.1   LEYGACPNNMMGTFSVRTVGSSK.SKYPLVVRIYMRMKHVRAWIPRPMRNQNYLFKANPNYAGNSIKPTGTSRTAITTL.
QPI19755.1   LEYGACPNNMMGTFSVRTVGSSK.SKYPLVVRIYMRMKHVRAWIPRPMRNQNYLFKANPNYAGNSIKPTGTSRTAITTL.
QPI19754.1   LEYGACPNNMMGTFSVRTVGSSK.SKYPLVVRIYMRMKHVRAWIPRPMRNQNYLFKANPNYAGNSIKPTGTSRTAITTL.
QPI19748.1   LEYGACPNNMMGTFSVRTVGSSK.SKYPLVVRIYMRMKHVRAWIPRPMRNQNYLFKANPNYAGNSIKPTGTSRTAITTL.
QPI19747.1   LEYGACPNNMMGTFSVRTVGSSK.SKYPLVVRIYMRMKHVRAWIPRPMRNQNYLFKANPNYAGNSIKPTGTSRTAITTL.
QPI19744.1   LEYGACPNNMMGTFSVRTVGSSK.SKYPLVVRIYMRMKHVRAWIPRPMRNQNYLFKANPNYAGNSIKPTGTSRTAITTL.
QPI19743.1   LEYGACPNNMMGTFSVRTVGSSK.SKYPLVVRIYMRMKHVRAWIPRPMRNQNYLFKANPNYAGNSIKPTGTSRTAITTL.
QPI19739.1   LEYGACPNNMMGTFSVRTVGSSK.SKYPLVVRIYMRMKHVRAWIPRPMRNQNYLFKANPNYAGNSIKPTGTSRTAITTL.
QPI19778.1   LEYGACPNNMMGTFSVRTVGSSK.SKYPLVVRIYMRMKHVRAWIPRPMRNQNYLFKANPNYAGNSIKPTGTSRTAITTL.
QPI19796.1   LEYGACPNNMMGTFSVRTVGSSK.SKYPLVVRIYMRMKHVRAWIPRPMRNQNYLFKANPNYAGNSIKPTGTSRTAITTL.
QPI19753.1   LEYGACPNNMMGTFSVRTVGSSK.SKYPLVVRIYMRMKHVRAWIPRPMRNQNYLFKANPNYAGNSIKPTGTSRTAITTL.
QPI19791.1   LEYGACPNNMMGTFSVRTVGSSK.SKYPLVVRIYMRMKHVRAWIPRPMRNQNYLFKANPNYAGNSIKPTGTSRTAITTL.
QPI19786.1   LEYGACPNNMMGTFSVRTVGSSK.SKYPLVVRIYMRMKHVRAWIPRPMRNQNYSFKANPNYAGNSIKPTGTSRTAITTL.
QPI19779.1   LEYGACPNNMMGTFSVRTVGSSK.SKYPLVVRIYMRMKHVRAWIPRPMRNQNYLFKANPNYAGNSIKPTGTSRTAITTL.
QPI19742.1   LEYGACPNNMMGTFSVRTVGSSK.SKYPLVVRIYMRMKHVRAWIPRPMRNQNYLFKANPNYAGNSIKPTGTSRTAITTL.
pdb|4AED|A   LEYGACPNNMMGTFSVRNVGSSK.SKYPLVVRIYMRMKHVRAWIPRPMRNQNYLFKANPNYAGNSIKPTGTSRTAITTL.
pdb|3ZFF|A   LEYGACPNNMMGTFSVRNVGSSK.SKYPLVVRIYMRMKHVRAWIPRPMRNQNYLFKANPNYAGNSIKPTGTSRTAITTL.
pdb|6DIZ|A   LEYGACPNNMMGTFSVRTVGSSK.SKYPLVVRIYMRMKHVRAWIPRPMRNQNYLFKANPNYAGNSIKPTGTSRSAITTL.
pdb|6UH7|A   LEYGACPNNMMGTFSVRTVGSSK.SKYPLVVRIYMRMKHVRAWIPRPMRNQNYLFKANPNYAGNSIKPTGTSRSAITTL.
pdb|6UH6|A   LEYGACPNNMMGTFSVRTVGSSK.SKYPLVVRIYMRMKHVRAWIPRPMRNQNYLFKANPNYAGNSIKPTGTSRSAITTL.
pdb|6UH1|A   LEYGACPNNMMGTFSVRTVGSSK.SKYPLVVRIYMRMKHVRAWIPRPMRNQNYLFKANPNYAGNSIKPTGTSRSAITTL.
QPI19783.1   LEYGACPNNMMGTFSVRTVGSSK.SKYPLVVRIYMRMKHVRAWIPRPMRNQNYLFKANPNYAGNSIKPTGTSRTAITTL.
QPI19772.1   LEYGACPNNMMGTFSVRTVGSSK.SKYPLVVRIYMRMKHVRAWIPRPMRNQNYLFKANPNYAGNSIKPTGTSRTAITTL.
QPI19771.1   LEYGACPNNMMGTFSVRTVGSSK.SKYPLVVRIYMRMKHVRAWIPRPMRNQNYLFKANPNYAGNSIKPTGTSRTAITTL.
QPI19764.1   LEYGACPNNMMGTFSVRTVGSSK.SKYPLVVRIYMRMKHVRAWIPRPMRNQNYLFKANPNYAGNSIKPTGTSRTAITTL.
QPI19760.1   LEYGACPNNMMGTFSVRTVGSSK.SKYPLVVRIYMRMKHVRAWIPRPMRNQNYLFKANPNYAGNSIKPTGTSRTAITTL.
QPI19759.1   LEYGACPNNMMGTFSVRTVGSSK.SKYPLVVRIYMRMKHVRAWIPRPMRNQNYLFKANPNYAGNSIKPTGTSRTAITTL.
QPI19756.1   LEYGACPNNMMGTFSVRTVGSSK.SKYPLVVRIYMRMKHVRAWIPRPMRNQNYLFKANPNYAGNSIKPTGTSRTAITTL.
QPI19752.1   LEYGACPNNMMGTFSVRTVGSSK.SKYPLVVRIYMRMKHVRAWIPRPMRNQNYLFKANPNYAGNSIKPTGTSRTAITTL.
QPI19740.1   LEYGACPNNMMGTFSVRTVGSSK.SKYPLVVRIYMRMKHVRAWIPRPMRNQNYLFKANPNYAGNSIKPTGTSRTAITTL.
QPI19738.1   LEYGACPNNMMGTFSVRTVGSSK.SKYPLVVRIYMRMKHVRAWIPRPMRNQNYLFKANPNYAGNSIKPTGTSRTAITTL.
QPI19757.1   LEYGACPNNMMGTFSVRTVGSSK.SKYPLVVRIYMRMKHVRAWIPRPMRNQNYLFKANPNYTGNSIKPTGTSRTAITTL.
QPI19750.1   LEYGACPNNMMGTFSVRTVGSSK.SKYPLVVRIYMRMKHVRAWIPRPMRNQNYLFKANPNYAGNSIKPTGTSRTAITTL.
QPI19751.1   LEYGACPNNMMGTFSVRTVGSSK.SKYPLVVRIYMRMKHVRAWIPRPMRNQNYLFKANPNYTGNSIKPTGTSRTAITTL.
QPI19746.1   LEYGACPNNMMGTFSVRTVGSLK.SKYPLVVRIYMRMKHVRAWIPRPMRNQNYLFKANPNYAGNSIKPTGTSRTAITTL.
QPI19737.1   LEYGACPNNMMGTFSVRTVGSSK.SKYPLVVRIYMRMKHVRAWIPRPMRNQNYLFKANPNYAGNSIKPTGTSRTAITTL.
QPI19776.1   LEYGACPNNMMGTFSVRTVGSSK.SKYPLVVRIYMRMKHVRAWIPRPMRNQNYLFKANPNYAGNSIKPTGTSRTAITTL.
QPI19774.1   LEYGACPNNMMGTFSVRTVGSSK.SKYPLVVRIYMRMKHVRAWIPRPMRNQNYLFKANPNYAGNSIKPTGTSRTAITTL.
QPI19773.1   LEYGACPNNMMGTFSVRTVGSSK.SKYPLVVRIYMRMKHVRAWIPRPMRNQNYLFKANPNYAGNSIKPTGTSRTAITTL.
QPI19770.1   LEYGACPNNMMGTFSVRTVGSSK.SKYPLVVRIYMRMKHVRAWIPRPMRNQNYLFKANPNYAGNSIKPTGTSRTAITTL.
QPI19769.1   LEYGACPNNMMGTFSVRTVGSSK.SKYPLVVRIYMRMKHVRAWIPRPMRNQNYLFKANPNYAGNSIKPTGTSRTAITTL.
QPI19768.1   LEYGACPNNMMGTFSVRTVGSSK.SKYPLVVRIYMRMKHVRAWIPRPMRNQNYLFKANPNYAGNSIKPTGTSRTAITTL.
QPI19767.1   LEYGACPNNMMGTFSVRTVGSSK.SKYPLVVRIYMRMKHVRAWIPRPMRNQNYLFKANPNYAGNSIKPTGTSRTAITTL.
QPI19766.1   LEYGACPNNMMGTFSVRTVGSSK.SKYPLVVRIYMRMKHVRAWIPRPMRNQNYLFKANPNYAGNSIKPTGTSRTAITTL.
QPI19763.1   LEYGACPNNMMGTFSVRTVGSSK.SKYPLVVRIYMRMKHVRAWIPRPMRNQNYLFKANPNYAGNSIKPTGTSRTAITTL.
QPI19761.1   LEYGACPNNMMGTFSVRTVGSSK.SKYPLVVRIYMRMKHVRAWIPRPMRNQNYLFKANPNYAGNSIKPTGTSRTAITTL.
QPI19765.1   LEYGACPNNMMGTFSVRTVGSSK.SKYPLVVGIYMRMKHVRAWIPRPMRNQNYLFKANPNYAGNSIKPTGTSRTAITTL.
QPI19775.1   LEYGACPNNMMGTFSVRTVGSSK.SKYPLVVRIYMRMKHVRAWIPRPMRNQNYLFKANPNYAGNSIKPTGTSRTAITTL.
QPI19758.1   LEYGACPNNMMGTFSVRTVGSSK.SKYPLVVRIYMRMKHVRAWIPRPMRNQNYLFKANPNYTGNSIKPTGTSRTAITTL.
QPI19736.1   LEYGACPNNMMGTFSVRTVGSSK.SKYPLVVRIYMRMKHVRAWIPRPMRNQNYLFKANPNYTGNSIKPTGTSRTAITTL.
QPI19735.1   LEYGACPNNMMGTFSVRTVGSSK.SKYPLVVRIYMRMKHVRAWIPRPMRNQNYLFKANPNYTGNSIKPTGTSRTAITTL.
QPI19749.1   LEYGACPNNMMGTFSVRTVGSSK.SKYPLVVRIYMRMKHVRAWIPRPMRNQNYLFKANPNYTGNSIKPTGTSRAAITTL.
QPI19741.1   LEYGACPNNMMGTFSVRTVGSSK.SKYPLVVRIYMRMKHVRAWIPRPMRNQNYLFKANPNYTGNSIKPTGTSRTAITTL.
QPI19745.1   LEYGACPNNMMGTFSVRTVGSSK.SKYPLVVRIYMRMKHVRAWIPRPMRNQNYLFKANPNYAGNSIKPTGTSRTAITTL.
pdb|6Z3Q|A   LEYGACPNNMMGTFSVRTVGSSK.SKYALVVRIYMRMKHVRAWIPRPMRNQNYLFKANPNYAGDSIKPTGTSRNAITTL.
pdb|6Z3P|A   LEYGACPNNMMGTFSVRTVGSSK.SKYALVVRIYMRMKHVRAWIPRPMRNQNYLFKANPNYAGDSIKPTGTSRNAITTL.
pdb|6I2K|A   LEYGACPNNMMGTFSVRTVGSSK.SKYALVVRIYMRMKHVRAWIPRPMRNQNYLFKANPNYAGDSIKPTGTSRNAITTL.
```

```
QWF08740.1    LDYGQCPNNMMGTFSIRTVGTEK.SPHSITLRVYMRIKHVRAWIPRPLRNQPYLFKTNPNYKGNDIKCTSTSRDKITTL.
QWF08739.1    LDYGQCPNNMMGTFSIRTVGTEK.SPHSITLRVYMRIKHVRAWIPRPLRNQPYLFKTNPNYKGNDIKCTSTSRDKITTL.
QWF08760.1    LDYGQCPNNMMGTFSIRTVGTEK.SPHSITLRVYMRIKHVRAWIPRPLRNQPYLFKTNPNYKGNDIKCTSTSRDKITTL.
QWF08759.1    LDYGQCPNNMMGTFSIRTVGTEK.SPHSITLRVYMRIKHVRAWIPRPLRNQPYLFKTNPNYKGNDIKCTSTSRDKITTL.
QWF08762.1    LDYGQCPNNMMGTFSIRTVGTEK.SPHSITLRVYMRIKHVRAWIPRPLRNQPYLFKTNPNYKGNDIKCTSTSRDKITTL.
QWF08761.1    LDYGQCPNNMMGTFSIRTVGTEK.SPHSITLRVYMRIKHVRAWIPRPLRNQPYLFKTNPNYKGNDIKCTSTSRDKITTL.
QWF08764.1    LDYGQCPNNMMGTFSIRTVGTEK.SPHSITLRVYMRIKHVRAWIPRPLRNQPYLFKTNPNYKGNDIKCTSTSRDKITTL.
QWF08763.1    LDYGQCPNNMMGTFSIRTVGTEK.SPHSITLRVYMRIKHVRAWIPRPLRNQPYLFKTNPNYKGNDIKCTSTSRDKITTL.
QWF08773.1    LDYGQCPNNMMGTFSIRTVGTEK.SPHSITLRVYMRIKHVRAWIPRPLRNQPYLFKTNPNYKGNDIKCTSTSRDKITTL.
QWF08749.1    LDYGQCPNNMMGTFSIRTVGTEK.SPHSITLRVYMRIKHVRAWIPRPLRNQPYLFKTNPNYKGNDIKCTSTSRDKITTL.
QWF08785.1    LDYGQCPNNMMGTFSIRTVGTEK.SPHSITLRVYMRIKHVRAWIPRPLRNQPYLFKTNPNYKGNDIKCTSTSRDKITTL.
QWF08783.1    LDYGQCPNNMMGTFSIRTVGTEK.SPHSITLRVYMRIKHVRAWIPRPLRNQPYLFKTNPNYKGNDIKCTSTSRDKITTL.
QWF08787.1    LDYGQCPNNMMGTFSIRTVGTEK.SPHSITLRVYMRIKHVRAWIPRPLRNQPYLFKTNPNYKGNDIKCTSTSRDKITTL.
QWF08765.1    LDYGQCPNNMMGTFSIRTVGTEK.SPHSITLRVYMRIKHVRAWIPRPLRNQPYLFKTNPNYKGNDIKCTSTSRDKITTL.
QWF08792.1    LDYGQCPNNMMGTFSIRTVGTEK.SPHSITLRVYMRIKHVRAWIPRPLRNQPYLFKTNPNYKGNDIKCTSTSRDKITTL.
QWF08767.1    LDYGQCPNNMMGTFSIRTVGTEK.SPHSITLRVYMRIKHVRAWIPRPLRNQPYLFKTNPNYKGNDIKCTSTSRDKITTL.
QWF08793.1    LDYGQCPNNMMGTFSIRTVGTEK.SPHSITLRVYMRIKHVRAWIPRPLRNQPYLFKTNPNYKGNDIKCTSTSRDKITTL.
QWF08758.1    LDYGQCPNNMMGTFSIRTVGTEK.SPHSITLRVYMRIKHVRAWIPRPLRNQPYLFKTNPNYKGNDIKCTSTSRDKITTL.
QWF08845.1    LDYGQCPNNMMGTFSIRTVGTEK.SPHSITLRVYMRIKHVRAWIPRPLRNQPYLFKTNPNYKGNDIKCTSTSRDKITTL.
QWF08844.1    LDYGQCPNNMMGTFSIRTVGTEK.SPHSITLRVYMRIKHVRAWIPRPLRNQPYLFKTNPNYKGNDIKCTSTSRDKITTL.
QWF08847.1    LDYGQCPNNMMGTFSIRTVGTEK.SPHSITLRVYMRIKHVRAWIPRPLRNQPYLFKTNPNYKGNDIKCTSTSRDKITTL.
QWF08846.1    LDYGQCPNNMMGTFSIRTVGTEK.SPHSITLRVYMRIKHVRAWIPRPLRNQPYLFKTNPNYKGNDIKCTSTSRDKITTL.
QWF08852.1    LDYGQCPNNMMGTFSIRTVGTEK.SPHSITLRVYMRIKHVRAWIPRPLRNQPYLFKTNPNYKGNDIKCTSTSRDKITTL.
QWF08843.1    LDYGQCPNNMMGTFSIRTVGTEK.SPHSITLRVYMRIKHVRAWIPRPLRNQPYLFKTNPNYKGNDIKCTSTSRDKITTL.
QWF08857.1    LDYGQCPNNMMGTFSIRTVGTEK.SPHSITLRVYMRIKHVRAWIPRPLRNQPYLFKTNPNYKGNDIKCTSTSRDKITTL.
QWF08856.1    LDYGQCPNNMMGTFSIRTVGTEK.SPHSITLRVYMRIKHVRAWIPRPLRNQPYLFKTNPNYKGNDIKCTSTSRDKITTL.
QWF08865.1    LDYGQCPNNMMGTFSIRTVGTEK.SPHSITLRVYMRIKHVRAWIPRPLRNQPYLFKTNPNYKGNDIKCTSTSRDKITTL.
QWF08738.1    LDYGQCPNNMMGTFSIRTVGTEK.SPHSITLRVYMRIKHVRAWIPRPLRNQPYLFKTNPNYKGNDIKCTSTSRDKITTL.
QWF08872.1    LDYGQCPNNMMGTFSIRTVGTEK.SPHSITLRVYMRIKHVRAWIPRPLRNQPYLFKTNPNYKGNDIKCTSTSRDKITTL.
QWF08871.1    LDYGQCPNNMMGTFSIRTVGTEK.SPHSITLRVYMRIKHVRAWIPRPLRNQPYLFKTNPNYKGNDIKCTSTSRDKITTL.
QWF08879.1    LDYGQCPNNMMGTFSIRTVGTEK.SPHSITLRVYMRIKHVRAWIPRPLRNQPYLFKTNPNYKGNDIKCTSTSRDKITTL.
QWF08877.1    LDYGQCPNNMMGTFSIRTVGTEK.SPHSITLRVYMRIKHVRAWIPRPLRNQPYLFKTNPNYKGNDIKCTSTSRDKITTL.
QWF08880.1    LDYGQCPNNMMGTFSIRTVGTEK.SPHSITLRVYMRIKHVRAWIPRPLRNQPYLFKTNPNYKGNDIKCTSTSRDKITTL.
QWF08851.1    LDYGQCPNNMMGTFSIRTVGTEK.SPHSITLRVYMRIKHVRAWIPRPLRNQPYLFKTNPNYKGNDIKCTSTSRDKITTL.
QWF08882.1    LDYGQCPNNMMGTFSIRTVGTEK.SPHSITLRVYMRIKHVRAWIPRPLRNQPYLFKTNPNYKGNDIKCTSTSRDKITTL.
QWF08867.1    LDYGQCPNNMMGTFSIRTVGTEK.SPHSITLRVYMRIKHVRAWIPRPLRNQPYLFKTNPNYKGNDIKCTSTSRDKITTL.
QWF08884.1    LDYGQCPNNMMGTFSIRTVGTEK.SPHSITLRVYMRIKHVRAWIPRPLRNQPYLFKTNPNYKGNDIKCTSTSRDKITTL.
QWF08875.1    LDYGQCPNNMMGTFSIRTVGTEK.SPHSITLRVYMRIKHVRAWIPRPLRNQPYLFKTNPNYKGNDIKCTSTSRDKITTL.
QWF08885.1    LDYGQCPNNMMGTFSIRTVGTEK.SPHSITLRVYMRIKHVRAWIPRPLRNQPYLFKTNPNYKGNDIKCTSTSRDKITTL.
QWF08863.1    LDYGQCPNNMMGTFSIRTVGTEK.SPHSITLRVYMRIKHVRAWIPRPLRNQPYLFKTNPNYKGNDIKCTSTSRDKITTL.
QWF08890.1    LDYGQCPNNMMGTFSIRTVGTEK.SPHSITLRVYMRIKHVRAWIPRPLRNQPYLFKTNPNYKGNDIKCTSTSRDKITTL.
QWF08889.1    LDYGQCPNNMMGTFSIRTVGTEK.SPHSITLRVYMRIKHVRAWIPRPLRNQPYLFKTNPNYKGNDIKCTSTSRDKITTL.
QWF08896.1    LDYGQCPNNMMGTFSIRTVGTEK.SPHSITLRVYMRIKHVRAWIPRPLRNQPYLFKTNPNYKGNDIKCTSTSRDKITTL.
QWF08752.1    LDYGQCPNNMMGTFSIRTVGTEK.SPHSITLRVYMRIKHVRAWIPRPLRNQPYLFKTNPNYKGNDIKCTSTSRDKITTL.
QWF08900.1    LDYGQCPNNMMGTFSIRTVGTEK.SPHSITLRVYMRIKHVRAWIPRPLRNQPYLFKTNPNYKGNDIKCTSTSRDKITTL.
QWF08899.1    LDYGQCPNNMMGTFSIRTVGTEK.SPHSITLRVYMRIKHVRAWIPRPLRNQPYLFKTNPNYKGNDIKCTSTSRDKITTL.
QWF08908.1    LDYGQCPNNMMGTFSIRTVGTEK.SPHSITLRVYMRIKHVRAWIPRPLRNQPYLFKTNPNYKGNDIKCTSTSRDKITTL.
QWF08906.1    LDYGQCPNNMMGTFSIRTVGTEK.SPHSITLRVYMRIKHVRAWIPRPLRNQPYLFKTNPNYKGNDIKCTSTSRDKITTL.
QWF08910.1    LDYGQCPNNMMGTFSIRTVGTEK.SPHSITLRVYMRIKHVRAWIPRPLRNQPYLFKTNPNYKGNDIKCTSTSRDKITTL.
QWF08870.1    LDYGQCPNNMMGTFSIRTVGTEK.SPHSITLRVYMRIKHVRAWIPRPLRNQPYLFKTNPNYKGNDIKCTSTSRDKITTL.
QWF08915.1    LDYGQCPNNMMGTFSIRTVGTEK.SPHSITLRVYMRIKHVRAWIPRPLRNQPYLFKTNPNYKGNDIKCTSTSRDKITTL.
QWF08750.1    LDYGQCPNNMMGTFSIRTVGTEK.SPHSITLRVYMRIKHVRAWIPRPLRNQPYLFKTNPNYKGNDIKCTSTSRDKITTL.
QWF08917.1    LDYGQCPNNMMGTFSIRTVGTEK.SPHSITLRVYMRIKHVRAWIPRPLRNQPYLFKTNPNYKGNDIKCTSTSRDKITTL.
QWF08916.1    LDYGQCPNNMMGTFSIRTVGTEK.SPHSITLRVYMRIKHVRAWIPRPLRNQPYLFKTNPNYKGNDIKCTSTSRDKITTL.
QWF08923.1    LDYGQCPNNMMGTFSIRTVGTEK.SPHSITLRVYMRIKHVRAWIPRPLRNQPYLFKTNPNYKGNDIKCTSTSRDKITTL.
QWF08921.1    LDYGQCPNNMMGTFSIRTVGTEK.SPHSITLRVYMRIKHVRAWIPRPLRNQPYLFKTNPNYKGNDIKCTSTSRDKITTL.
QWF08926.1    LDYGQCPNNMMGTFSIRTVGTEK.SPHSITLRVYMRIKHVRAWIPRPLRNQPYLFKTNPNYKGNDIKCTSTSRDKITTL.
QWF08780.1    LDYGQCPNNMMGTFSIRTVGTEK.SPHSITLRVYMRIKHVRAWIPRPLRNQPYLFKTNPNYKGNDIKCTSTSRDKITTL.
QWF08932.1    LDYGQCPNNMMGTFSIRTVGTEK.SPHSITLRVYMRIKHVRAWIPRPLRNQPYLFKTNPNYKGNDIKCTSTSRDKITTL.
QWF08931.1    LDYGQCPNNMMGTFSIRTVGTEK.SPHSITLRVYMRIKHVRAWIPRPLRNQPYLFKTNPNYKGNDIKCTSTSRDKITTL.
QWF08945.1    LDYGQCPNNMMGTFSIRTVGTEK.SPHSITLRVYMRIKHVRAWIPRPLRNQPYLFKTNPNYKGNDIKCTSTSRDKITTL.
QWF08938.1    LDYGQCPNNMMGTFSIRTVGTEK.SPHSITLRVYMRIKHVRAWIPRPLRNQPYLFKTNPNYKGNDIKCTSTSRDKITTL.
QWF08955.1    LDYGQCPNNMMGTFSIRTVGTEK.SPHSITLRVYMRIKHVRAWIPRPLRNQPYLFKTNPNYKGNDIKCTSTSRDKITTL.
QWF08954.1    LDYGQCPNNMMGTFSIRTVGTEK.SPHSITLRVYMRIKHVRAWIPRPLRNQPYLFKTNPNYKGNDIKCTSTSRDKITTL.
QWF08967.1    LDYGQCPNNMMGTFSIRTVGTEK.SPHSITLRVYMRIKHVRAWIPRPLRNQPYLFKTNPNYKGNDIKCTSTSRDKITTL.
QWF08959.1    LDYGQCPNNMMGTFSIRTVGTEK.SPHSITLRVYMRIKHVRAWIPRPLRNQPYLFKTNPNYKGNDIKCTSTSRDKITTL.
QYC65038.1    LDYGQCPNNMMGTFSIRTVGTEK.SPHSITLRVYMRIKHVRAWIPRPLRNQPYLFKTNPNYKGNDIKCTSTSRDKITTL.
QYC65037.1    LDYGQCPNNMMGTFSIRTVGTEK.SPHSITLRVYMRIKHVRAWIPRPLRNQPYLFKTNPNYKGNDIKCTSTSRDKITTL.
QYC65039.1    LDYGQCPNNMMGTFSIRTVGTEK.SPHSITLRVYMRIKHVRAWIPRPLRNQPYLFKTNPNYKGNDIKCTSTSRDKITTL.
QYC65034.1    LDYGQCPNNMMGTFSIRTVGTEK.SPHSITLRVYMRIKHVRAWIPRPLRNQPYLFKTNPNYKGNDIKCTSTSRDKITTL.
QYC65043.1    LDYGQCPNNMMGTFSIRTVGTEK.SPHSITLRVYMRIKHVRAWIPRPLRNQPYLFKTNPNYKGNDIKCTSTSRDKITTL.
QYC65041.1    LDYGQCPNNMMGTFSIRTVGTEK.SPHSITLRVYMRIKHVRAWIPRPLRNQPYLFKTNPNYKGNDIKCTSTSRDKITTL.
QYC65047.1    LDYGQCPNNMMGTFSIRTVGTEK.SPHSITLRVYMRIKHVRAWIPRPLRNQPYLFKTNPNYKGNDIKCTSTSRDKITTL.
QYC65046.1    LDYGQCPNNMMGTFSIRTVGTEK.SPHSITLRVYMRIKHVRAWIPRPLRNQPYLFKTNPNYKGNDIKCTSTSRDKITTL.
QYC65050.1    LDYGQCPNNMMGTFSIRTVGTEK.SPHSITLRVYMRIKHVRAWIPRPLRNQPYLFKTNPNYKGNDIKCTSTSRDKITTL.
QYC65049.1    LDYGQCPNNMMGTFSIRTVGTEK.SPHSITLRVYMRIKHVRAWIPRPLRNQPYLFKTNPNYKGNDIKCTSTSRDKITTL.
QYC65051.1    LDYGQCPNNMMGTFSIRTVGTEK.SPHSITLRVYMRIKHVRAWIPRPLRNQPYLFKTNPNYKGNDIKCTSTSRDKITTL.
QYC65045.1    LDYGQCPNNMMGTFSIRTVGTEK.SPHSITLRVYMRIKHVRAWIPRPLRNQPYLFKTNPNYKGNDIKCTSTSRDKITTL.
QYC65058.1    LDYGQCPNNMMGTFSIRTVGTEK.SPHSITLRVYMRIKHVRAWIPRPLRNQPYLFKTNPNYKGNDIKCTSTSRDKITTL.
QYC65055.1    LDYGQCPNNMMGTFSIRTVGTEK.SPHSITLRVYMRIKHVRAWIPRPLRNQPYLFKTNPNYKGNDIKCTSTSRDKITTL.
QYC65060.1    LDYGQCPNNMMGTFSIRTVGTEK.SPHSITLRVYMRIKHVRAWIPRPLRNQPYLFKTNPNYKGNDIKCTSTSRDKITTL.
QYC65059.1    LDYGQCPNNMMGTFSIRTVGTEK.SPHSITLRVYMRIKHVRAWIPRPLRNQPYLFKTNPNYKGNDIKCTSTSRDKITTL.
QYC65062.1    LDYGQCPNNMMGTFSIRTVGTEK.SPHSITLRVYMRIKHVRAWIPRPLRNQPYLFKTNPNYKGNDIKCTSTSRDKITTL.
QYC65061.1    LDYGQCPNNMMGTFSIRTVGTEK.SPHSITLRVYMRIKHVRAWIPRPLRNQPYLFKTNPNYKGNDIKCTSTSRDKITTL.
QYC65063.1    LDYGQCPNNMMGTFSIRTVGTEK.SPHSITLRVYMRIKHVRAWIPRPLRNQPYLFKTNPNYKGNDIKCTSTSRDKITTL.
QYC65053.1    LDYGQCPNNMMGTFSIRTVGTEK.SPHSITLRVYMRIKHVRAWIPRPLRNQPYLFKTNPNYKGNDIKCTSTSRDKITTL.
QYC65069.1    LDYGQCPNNMMGTFSIRTVGTEK.SPHSITLRVYMRIKHVRAWIPRPLRNQPYLFKTNPNYKGNDIKCTSTSRDKITTL.
QYC65068.1    LDYGQCPNNMMGTFSIRTVGTEK.SPHSITLRVYMRIKHVRAWIPRPLRNQPYLFKTNPNYKGNDIKCTSTSRDKITTL.
QYC65071.1    LDYGQCPNNMMGTFSIRTVGTEK.SPHSITLRVYMRIKHVRAWIPRPLRNQPYLFKTNPNYKGNDIKCTSTSRDKITTL.
QYC65070.1    LDYGQCPNNMMGTFSIRTVGTEK.SPHSITLRVYMRIKHVRAWIPRPLRNQPYLFKTNPNYKGNDIKCTSTSRDKITTL.
QYC65074.1    LDYGQCPNNMMGTFSIRTVGTEK.SPHSITLRVYMRIKHVRAWIPRPLRNQPYLFKTNPNYKGNDIKCTSTSRDKITTL.
QYC65066.1    LDYGQCPNNMMGTFSIRTVGTEK.SPHSITLRVYMRIKHVRAWIPRPLRNQPYLFKTNPNYKGNDIKCTSTSRDKITTL.
QYC65079.1    LDYGQCPNNMMGTFSIRTVGTEK.SPHSITLRVYMRIKHVRAWIPRPLRNQPYLFKTNPNYKGNDIKCTSTSRDKITTL.
QYC65073.1    LDYGQCPNNMMGTFSIRTVGTEK.SPHSITLRVYMRIKHVRAWIPRPLRNQPYLFKTNPNYKGNDIKCTSTSRDKITTL.
QYC65081.1    LDYGQCPNNMMGTFSIRTVGTEK.SPHSITLRVYMRIKHVRAWIPRPLRNQPYLFKTNPNYKGNDIKCTSTSRDKITTL.
QYC65078.1    LDYGQCPNNMMGTFSIRTVGTEK.SPHSITLRVYMRIKHVRAWIPRPLRNQPYLFKTNPNYKGNDIKCTSTSRDKITTL.
QYC65086.1    LDYGQCPNNMMGTFSIRTVGTEK.SPHSITLRVYMRIKHVRAWIPRPLRNQPYLFKTNPNYKGNDIKCTSTSRDKITTL.
QYC65085.1    LDYGQCPNNMMGTFSIRTVGTEK.SPHSITLRVYMRIKHVRAWIPRPLRNQPYLFKTNPNYKGNDIKCTSTSRDKITTL.
QYC65088.1    LDYGQCPNNMMGTFSIRTVGTEK.SPHSITLRVYMRIKHVRAWIPRPLRNQPYLFKTNPNYKGNDIKCTSTSRDKITTL.
QYC65087.1    LDYGQCPNNMMGTFSIRTVGTEK.SPHSITLRVYMRIKHVRAWIPRPLRNQPYLFKTNPNYKGNDIKCTSTSRDKITTL.
QYC65101.1    LDYGQCPNNMMGTFSIRTVGTEK.SPHSITLRVYMRIKHVRAWIPRPLRNQPYLFKTNPNYKGNDIKCTSTSRDKITTL.
QYC65093.1    LDYGQCPNNMMGTFSIRTVGTEK.SPHSITLRVYMRIKHVRAWIPRPLRNQPYLFKTNPNYKGNDIKCTSTSRDKITTL.
QYC65107.1    LDYGQCPNNMMGTFSIRTVGTEK.SPHSITLRVYMRIKHVRAWIPRPLRNQPYLFKTNPNYKGNDIKCTSTSRDKITTL.
QYC65104.1    LDYGQCPNNMMGTFSIRTVGTEK.SPHSITLRVYMRIKHVRAWIPRPLRNQPYLFKTNPNYKGNDIKCTSTSRDKITTL.
QYC65113.1    LDYGQCPNNMMGTFSIRTVGTEK.SPHSITLRVYMRIKHVRAWIPRPLRNQPYLFKTNPNYKGNDIKCTSTSRDKITTL.
QYC65106.1    LDYGQCPNNMMGTFSIRTVGTEK.SPHSITLRVYMRIKHVRAWIPRPLRNQPYLFKTNPNYKGNDIKCTSTSRDKITTL.
QYC65120.1    LDYGQCPNNMMGTFSIRTVGTEK.SPHSITLRVYMRIKHVRAWIPRPLRNQPYLFKTNPNYKGNDIKCTSTSRDKITTL.
QYC65118.1    LDYGQCPNNMMGTFSIRTVGTEK.SPHSITLRVYMRIKHVRAWIPRPLRNQPYLFKTNPNYKGNDIKCTSTSRDKITTL.
QYC65121.1    LDYGQCPNNMMGTFSIRTVGTEK.SPHSITLRVYMRIKHVRAWIPRPLRNQPYLFKTNPNYKGNDIKCTSTSRDKITTL.
QYC65111.1    LDYGQCPNNMMGTFSIRTVGTEK.SPHSITLRVYMRIKHVRAWIPRPLRNQPYLFKTNPNYKGNDIKCTSTSRDKITTL.
QYC65125.1    LDYGQCPNNMMGTFSIRTVGTEK.SPHSITLRVYMRIKHVRAWIPRPLRNQPYLFKTNPNYKGNDIKCTSTSRDKITTL.
QYC65117.1    LDYGQCPNNMMGTFSIRTVGTEK.SPHSITLRVYMRIKHVRAWIPRPLRNQPYLFKTNPNYKGNDIKCTSTSRDKITTL.
QYC65135.1    LDYGQCPNNMMGTFSIRTVGTEK.SPHSITLRVYMRIKHVRAWIPRPLRNQPYLFKTNPNYKGNDIKCTSTSRDKITTL.
QYC65134.1    LDYGQCPNNMMGTFSIRTVGTEK.SPHSITLRVYMRIKHVRAWIPRPLRNQPYLFKTNPNYKGNDIKCTSTSRDKITTL.
QYC65151.1    LDYGQCPNNMMGTFSIRTVGTEK.SPHSITLRVYMRIKHVRAWIPRPLRNQPYLFKTNPNYKGNDIKCTSTSRDKITTL.
QYC65150.1    LDYGQCPNNMMGTFSIRTVGTEK.SPHSITLRVYMRIKHVRAWIPRPLRNQPYLFKTNPNYKGNDIKCTSTSRDKITTL.
QYC65153.1    LDYGQCPNNMMGTFSIRTVGTEK.SPHSITLRVYMRIKHVRAWIPRPLRNQPYLFKTNPNYKGNDIKCTSTSRDKITTL.
QYC65152.1    LDYGQCPNNMMGTFSIRTVGTEK.SPHSITLRVYMRIKHVRAWIPRPLRNQPYLFKTNPNYKGNDIKCTSTSRDKITTL.
QYC65155.1    LDYGQCPNNMMGTFSIRTVGTEK.SPHSITLRVYMRIKHVRAWIPRPLRNQPYLFKTNPNYKGNDIKCTSTSRDKITTL.
QYC65154.1    LDYGQCPNNMMGTFSIRTVGTEK.SPHSITLRVYMRIKHVRAWIPRPLRNQPYLFKTNPNYKGNDIKCTSTSRDKITTL.
QYC65156.1    LDYGQCPNNMMGTFSIRTVGTEK.SPHSITLRVYMRIKHVRAWIPRPLRNQPYLFKTNPNYKGNDIKCTSTSRDKITTL.
QYC65142.1    LDYGQCPNNMMGTFSIRTVGTEK.SPHSITLRVYMRIKHVRAWIPRPLRNQPYLFKTNPNYKGNDIKCTSTSRDKITTL.
QYC65161.1    LDYGQCPNNMMGTFSIRTVGTEK.SPHSITLRVYMRIKHVRAWIPRPLRNQPYLFKTNPNYKGNDIKCTSTSRDKITTL.
QYC65160.1    LDYGQCPNNMMGTFSIRTVGTEK.SPHSITLRVYMRIKHVRAWIPRPLRNQPYLFKTNPNYKGNDIKCTSTSRDKITTL.
QYC65165.1    LDYGQCPNNMMGTFSIRTVGTEK.SPHSITLRVYMRIKHVRAWIPRPLRNQPYLFKTNPNYKGNDIKCTSTSRDKITTL.
QYC65164.1    LDYGQCPNNMMGTFSIRTVGTEK.SPHSITLRVYMRIKHVRAWIPRPLRNQPYLFKTNPNYKGNDIKCTSTSRDKITTL.
QYC65167.1    LDYGQCPNNMMGTFSIRTVGTEK.SPHSITLRVYMRIKHVRAWIPRPLRNQPYLFKTNPNYKGNDIKCTSTSRDKITTL.
QYC65166.1    LDYGQCPNNMMGTFSIRTVGTEK.SPHSITLRVYMRIKHVRAWIPRPLRNQPYLFKTNPNYKGNDIKCTSTSRDKITTL.
BCB92151.1    LDYGQCPNNMMGTFSIRTVGTEK.SPHSITLRVYMRIKHVRAWIPRPLRNQPYLFKTNPNYKGNDIKCTSTSRDKITTL.
BBU37462.1    LDYGQCPNNMMGTFSIRTVGTEK.SPHSITLRVYMRIKHVRAWIPRPLRNQPYLFKTNPNYKGNDIKCTSTSRDKITTL.
BBU37461.1    LDYGQCPNNMMGTFSIRTVGTEK.SPHSITLRVYMRIKHVRAWIPRPLRNQPYLFKTNPNYKGNDIKCTSTSRDKITTL.
BBU37459.1    LDYGQCPNNMMGTFSIRTVGTEK.SPHSITLRVYMRIKHVRAWIPRPLRNQPYLFKTNPNYKGNDIKCTSTSRDKITTL.
BBU37458.1    LDYGQCPNNMMGTFSIRTVGTEK.SPHSITLRVYMRIKHVRAWIPRPLRNQPYLFKTNPNYKGNDIKCTSTSRDKITTL.
BBU37457.1    LDYGQCPNNMMGTFSIRTVGTEK.SPHSITLRVYMRIKHVRAWIPRPLRNQPYLFKTNPNYKGNDIKCTSTSRDKITTL.
QWF08838.1    LDYGQCPNNMMGTFSIRTVGTEK.SPHSITLRVYMRIKHVRAWIPRPLRNQPYLFKTNPNYKGNDIKCTSTSRDKITTL.
QWF08828.1    LDYGQCPNNMMGTFSIRTVGTEK.SPHSITLRVYMRIKHVRAWIPRPLRNQPYLFKTNPNYKGNDIKCTSTSRDKITTL.
BCG66989.1    LDYGQCPNNMMGTFSIRTVGTEK.SPHSITLRVYMRIKHVRAWIPRPLRNQPYLFKTNPNYKGNDIKCTSTSRDKITTL.
BCG66987.1    LDYGQCPNNMMGTFSIRTVGTEK.SPHSITLRVYMRIKHVRAWIPRPLRNQPYLFKTNPNYKGNDIKCTSTSRDKITTL.
BCB92159.1    LDYGQCPNNMMGTFSIRTVGTEK.SPHSITLRVYMRIKHVRAWIPRPLRNQPYLFKTNPNYKGNDIKCTSTSRDKITTL.
BBU37456.1    LDYGQCPNNMMGTFSIRTVGTEK.SPHSITLRVYMRIKHVRAWIPRPLRNQPYLFKTNPNYKGNDIKCTSTSRDKITTL.
QWY15683.1    LDYGQCPNNMMGTFSIRTVGTEK.SPHSITLRVYMRIKHVRAWIPRPLRNQPYLFKTNPNYKGNDIKCTSTSRDKITTL.
QWY15682.1    LDYGQCPNNMMGTFSIRTVGTEK.SPHSITLRVYMRIKHVRAWIPRPLRNQPYLFKTNPNYKGNDIKCTSTSRDKITTL.
QWY15681.1    LDYGQCPNNMMGTFSIRTVGTEK.SPHSITLRVYMRIKHVRAWIPRPLRNQPYLFKTNPNYKGNDIKCTSTSRDKITTL.
QWY15680.1    LDYGQCPNNMMGTFSIRTVGTEK.SPHSITLRVYMRIKHVRAWIPRPLRNQPYLFKTNPNYKGNDIKCTSTSRDKITTL.
```

```
QWY15692.1   LDYGQCPNNMMGTFSIRTVGTEK.SPHSITLRVYMRIKHVRAWIPRPLRNQPYLFKTNPNYKGNDIKCTSTSRDKITTL.
QWY15671.1   LDYGQCPNNMMGTFSIRTVGTEK.SPHSITLRVYMRIKHVRAWIPRPLRNQPYLFKTNPNYKGNDIKCTSTSRDKITTL.
QWY15691.1   LDYGQCPNNMMGTFSIRTVGTEK.SPHSITLRVYMRIKHVRAWIPRPLRNQPYLFKTNPNYKGNDIKCTSTSRDKITTL.
QWY15690.1   LDYGQCPNNMMGTFSIRTVGTEK.SPHSITLRVYMRIKHVRAWIPRPLRNQPYLFKTNPNYKGNDIKCTSTSRDKITTL.
QWY15696.1   LDYGQCPNNMMGTFSIRTVGTEK.SPHSITLRVYMRIKHVRAWIPRPLRNQPYLFKTNPNYKGNDIKCTSTSRDKITTL.
QWY15695.1   LDYGQCPNNMMGTFSIRTVGTEK.SPHSITLRVYMRIKHVRAWIPRPLRNQPYLFKTNPNYKGNDIKCTSTSRDKITTL.
QWY15694.1   LDYGQCPNNMMGTFSIRTVGTEK.SPHSITLRVYMRIKHVRAWIPRPLRNQPYLFKTNPNYKGNDIKCTSTSRDKITTL.
QWY15678.1   LDYGQCPNNMMGTFSIRTVGTEK.SPHSITLRVYMRIKHVRAWIPRPLRNQPYLFKTNPNYKGNDIKCTSTSRDKITTL.
QWY15703.1   LDYGQCPNNMMGTFSIRTVGTEK.SPHSITLRVYMRIKHVRAWIPRPLRNQPYLFKTNPNYKGNDIKCTSTSRDKITTL.
QWY15701.1   LDYGQCPNNMMGTFSIRTVGTEK.SPHSITLRVYMRIKHVRAWIPRPLRNQPYLFKTNPNYKGNDIKCTSTSRDKITTL.
QWY15699.1   LDYGQCPNNMMGTFSIRTVGTEK.SPHSITLRVYMRIKHVRAWIPRPLRNQPYLFKTNPNYKGNDIKCTSTSRDKITTL.
QWY15673.1   LDYGQCPNNMMGTFSIRTVGTEK.SPHSITLRVYMRIKHVRAWIPRPLRNQPYLFKTNPNYKGNDIKCTSTSRDKITTL.
QWY15715.1   LDYGQCPNNMMGTFSIRTVGTEK.SPHSITLRVYMRIKHVRAWIPRPLRNQPYLFKTNPNYKGNDIKCTSTSRDKITTL.
QWY15714.1   LDYGQCPNNMMGTFSIRTVGTEK.SPHSITLRVYMRIKHVRAWIPRPLRNQPYLFKTNPNYKGNDIKCTSTSRDKITTL.
QWY15706.1   LDYGQCPNNMMGTFSIRTVGTEK.SPHSITLRVYMRIKHVRAWIPRPLRNQPYLFKTNPNYKGNDIKCTSTSRDKITTL.
QWY15705.1   LDYGQCPNNMMGTFSIRTVGTEK.SPHSITLRVYMRIKHVRAWIPRPLRNQPYLFKTNPNYKGNDIKCTSTSRDKITTL.
QTA73914.1   LDYGQCPNNMMGTFSIRTVGTEK.SPHSITLRVYMRIKHVRAWIPRPLRNQPYLFKTNPNYKGNDIKCTSTSRDKITTL.
QWY15948.1   LDYGQCPNNMMGTFSIRTVGTEK.SPHSITLRVYMRIKHVRAWIPRPLRNQPYLFKTNPNYKGNDIKCTSTSRDKITTL.
QWF08837.1   LDYGQCPNNMMGTFSIRTVGTEK.SPHSITLRVYMRIKHVRAWIPRPLRNQPYLFKTNPNYKGNDIKCTSTSRDKITTL.
QWF08804.1   LDYGQCPNNMMGTFSIRTVGTEK.SPHSITLRVYMRIKHVRAWIPRPLRNQPYLFKTNPNYKGNDIKCTSTSRDKITTL.
QWF08805.1   LDYGQCPNNMMGTFSIRTVGTEK.SPHSITLRVYMRIKHVRAWIPRPLRNQPYLFKTNPNYKGNDIKCTSTSRDKITTL.
QWF08840.1   LDYGQCPNNMMGTFSIRTVGTEK.SPHSITLRVYMRIKHVRAWIPRPLRNQPYLFKTNPNYKGNDIKCTSTSRDKITTL.
QWY15792.1   LDYGQCPNNMMGTFSIRTVGTEK.SPHSITLRVYMRIKHVRAWIPRPLRNQPYLFKTNPNYKGNDIKCTSTSRDKITTL.
QTA73903.1   LDYGQCPNNMMGTFSIRTVGTEK.SPHSITLRVYMRIKHVRAWIPRPLRNQPYLFKTNPNYKGNDIKCTSTSRDKITTL.
QTA73902.1   LDYGQCPNNMMGTFSIRTVGTEK.SPHSITLRVYMRIKHVRAWIPRPLRNQPYLFKTNPNYKGNDIKCTSTSRDKITTL.
QTA73896.1   LDYGQCPNNMMGTFSIRTVGTEK.SPHSITLRVYMRIKHVRAWIPRPLRNQPYLFKTNPNYKGNDIKCTSTSRDKITTL.
QTA73894.1   LDYGQCPNNMMGTFSIRTVGTEK.SPHSITLRVYMRIKHVRAWIPRPLRNQPYLFKTNPNYKGNDIKCTSTSRDKITTL.
QWY15940.1   LDYGQCPNNMMGTFSIRTVGTEK.SPHSITLRVYMRIKHVRAWIPRPLRNQPYLFKTNPNYKGNDIKCTSTSRDKITTL.
QWY15928.1   LDYGQCPNNMMGTFSIRTVGTEK.SPHSITLRVYMRIKHVRAWIPRPLRNQPYLFKTNPNYKGNDIKCTSTSRDKITTL.
QWY15894.1   LDYGQCPNNMMGTFSIRTVGTEK.SPHSITLRVYMRIKHVRAWIPRPLRNQPYLFKTNPNYKGNDIKCTSTSRDKITTL.
BCG67001.1   LDYGQCPNNMMGTFSIRTVGTEK.SPHSITLRVYMRIKHVRAWIPRPLRNQPYLFKTNPNYKGNDIKCTSTSRDKITTL.
QWY15872.1   LDYGQCPNNMMGTFSIRTVGTEK.SPHSITLRVYMRIKHVRAWIPRPLRNQPYLFKTNPNYKGNDIKCTSTSRDKITTL.
QWY15871.1   LDYGQCPNNMMGTFSIRTVGTEK.SPHSITLRVYMRIKHVRAWIPRPLRNQPYLFKTNPNYKGNDIKCTSTSRDKITTL.
QWY15870.1   LDYGQCPNNMMGTFSIRTVGTEK.SPHSITLRVYMRIKHVRAWIPRPLRNQPYLFKTNPNYKGNDIKCTSTSRDKITTL.
QWY15869.1   LDYGQCPNNMMGTFSIRTVGTEK.SPHSITLRVYMRIKHVRAWIPRPLRNQPYLFKTNPNYKGNDIKCTSTSRDKITTL.
QWY15866.1   LDYGQCPNNMMGTFSIRTVGTEK.SPHSITLRVYMRIKHVRAWIPRPLRNQPYLFKTNPNYKGNDIKCTSTSRDKITTL.
BCB92155.1   LDYGQCPNNMMGTFSIRTVGTEK.SPHSITLRVYMRIKHVRAWIPRPLRNQPYLFKTNPNYKGNDIKCTSTSRDKITTL.
QWY15862.1   LDYGQCPNNMMGTFSIRTVGTEK.SPHSITLRVYMRIKHVRAWIPRPLRNQPYLFKTNPNYKGNDIKCTSTSRDKITTL.
QWY15861.1   LDYGQCPNNMMGTFSIRTVGTEK.SPHSITLRVYMRIKHVRAWIPRPLRNQPYLFKTNPNYKGNDIKCTSTSRDKITTL.
QWY15852.1   LDYGQCPNNMMGTFSIRTVGTEK.SPHSITLRVYMRIKHVRAWIPRPLRNQPYLFKTNPNYKGNDIKCTSTSRDKITTL.
QWY15828.1   LDYGQCPNNMMGTFSIRTVGTEK.SPHSITLRVYMRIKHVRAWIPRPLRNQPYLFKTNPNYKGNDIKCTSTSRDKITTL.
QWY15839.1   LDYGQCPNNMMGTFSIRTVGTEK.SPHSITLRVYMRIKHVRAWIPRPLRNQPYLFKTNPNYKGNDIKCTSTSRDKITTL.
QWY15834.1   LDYGQCPNNMMGTFSIRTVGTEK.SPHSITLRVYMRIKHVRAWIPRPLRNQPYLFKTNPNYKGNDIKCTSTSRDKITTL.
QWY15810.1   LDYGQCPNNMMGTFSIRTVGTEK.SPHSITLRVYMRIKHVRAWIPRPLRNQPYLFKTNPNYKGNDIKCTSTSRDKITTL.
QWY15809.1   LDYGQCPNNMMGTFSIRTVGTEK.SPHSITLRVYMRIKHVRAWIPRPLRNQPYLFKTNPNYKGNDIKCTSTSRDKITTL.
QWY15807.1   LDYGQCPNNMMGTFSIRTVGTEK.SPHSITLRVYMRIKHVRAWIPRPLRNQPYLFKTNPNYKGNDIKCTSTSRDKITTL.
QWY15806.1   LDYGQCPNNMMGTFSIRTVGTEK.SPHSITLRVYMRIKHVRAWIPRPLRNQPYLFKTNPNYKGNDIKCTSTSRDKITTL.
QWY15805.1   LDYGQCPNNMMGTFSIRTVGTEK.SPHSITLRVYMRIKHVRAWIPRPLRNQPYLFKTNPNYKGNDIKCTSTSRDKITTL.
QWY15803.1   LDYGQCPNNMMGTFSIRTVGTEK.SPHSITLRVYMRIKHVRAWIPRPLRNQPYLFKTNPNYKGNDIKCTSTSRDKITTL.
QWY15793.1   LDYGQCPNNMMGTFSIRTVGTEK.SPHSITLRVYMRIKHVRAWIPRPLRNQPYLFKTNPNYKGNDIKCTSTSRDKITTL.
QYC65185.1   LDYGQCPNNMMGTFSIRTVGTEK.SPHSITLRVYMRIKHVRAWIPRPLRNQPYLFKTNPNYKGNDIKCTSTSRDKITTL.
QWY15754.1   LDYGQCPNNMMGTFSIRTVGTEK.SPHSITLRVYMRIKHVRAWIPRPLRNQPYLFKTNPNYKGNDIKCTSTSRDKITTL.
QWY15736.1   LDYGQCPNNMMGTFSIRTVGTEK.SPHSITLRVYMRIKHVRAWIPRPLRNQPYLFKTNPNYKGNDIKCTSTSRDKITTL.
QWY15753.1   LDYGQCPNNMMGTFSIRTVGTEK.SPHSITLRVYMRIKHVRAWIPRPLRNQPYLFKTNPNYKGNDIKCTSTSRDKITTL.
QYC65174.1   LDYGQCPNNMMGTFSIRTVGTEK.SPHSITLRVYMRIKHVRAWIPRPLRNQPYLFKTNPNYKGNDIKCTSTSRDKITTL.
QWY15730.1   LDYGQCPNNMMGTFSIRTVGTEK.SPHSITLRVYMRIKHVRAWIPRPLRNQPYLFKTNPNYKGNDIKCTSTSRDKITTL.
QWY15729.1   LDYGQCPNNMMGTFSIRTVGTEK.SPHSITLRVYMRIKHVRAWIPRPLRNQPYLFKTNPNYKGNDIKCTSTSRDKITTL.
QWY15727.1   LDYGQCPNNMMGTFSIRTVGTEK.SPHSITLRVYMRIKHVRAWIPRPLRNQPYLFKTNPNYKGNDIKCTSTSRDKITTL.
QWY15726.1   LDYGQCPNNMMGTFSIRTVGTEK.SPHSITLRVYMRIKHVRAWIPRPLRNQPYLFKTNPNYKGNDIKCTSTSRDKITTL.
QWY15725.1   LDYGQCPNNMMGTFSIRTVGTEK.SPHSITLRVYMRIKHVRAWIPRPLRNQPYLFKTNPNYKGNDIKCTSTSRDKITTL.
QWY15724.1   LDYGQCPNNMMGTFSIRTVGTEK.SPHSITLRVYMRIKHVRAWIPRPLRNQPYLFKTNPNYKGNDIKCTSTSRDKITTL.
QWY15723.1   LDYGQCPNNMMGTFSIRTVGTEK.SPHSITLRVYMRIKHVRAWIPRPLRNQPYLFKTNPNYKGNDIKCTSTSRDKITTL.
QWY15721.1   LDYGQCPNNMMGTFSIRTVGTEK.SPHSITLRVYMRIKHVRAWIPRPLRNQPYLFKTNPNYKGNDIKCTSTSRDKITTL.
QWY15746.1   LDYGQCPNNMMGTFSIRTVGTEK.SPHSITLRVYMRIKHVRAWIPRPLRNQPYLFKTNPNYKGNDIKCTSTSRDKITTL.
QWY15745.1   LDYGQCPNNMMGTFSIRTVGTEK.SPHSITLRVYMRIKHVRAWIPRPLRNQPYLFKTNPNYKGNDIKCTSTSRDKITTL.
QWY15733.1   LDYGQCPNNMMGTFSIRTVGTEK.SPHSITLRVYMRIKHVRAWIPRPLRNQPYLFKTNPNYKGNDIKCTSTSRDKITTL.
QYC65131.1   LDYGQCPNNMMGTFSIRTVGTEK.SPHSITLRVYMRIKHVRAWIPRPLRNQPYLFKTNPNYKGNDIKCTSTSRDKITTL.
QWY15813.1   LDYGQCPNNMMGTFSIRTVGTEK.SPHSITLRVYMRIKHVRAWIPRPLRNQPYLFKTNPNYKGNDIKCTSTSRDKITTL.
QWY15811.1   LDYGQCPNNMMGTFSIRTVGTEK.SPHSITLRVYMRIKHVRAWIPRPLRNQPYLFKTNPNYKGNDIKCTSTSRDKITTL.
QTA73907.1   LDYGQCPNNMMGTFSIRTVGTEK.SPHSITLRVYMRIKHVRAWIPRPLRNQPYLFKTNPNYKGNDIKCTSTSRDKITTL.
QTA73906.1   LDYGQCPNNMMGTFSIRTVGTEK.SPHSITLRVYMRIKHVRAWIPRPLRNQPYLFKTNPNYKGNDIKCTSTSRDKITTL.
QOP74940.1   LDYGQCPNNMMGTFSIRTVGTEK.SPHSITLRVYMRIKHVRAWIPRPLRNQPYLFKTNPNYKGNDIKCTSTSRDKITT..
QOP74938.1   LDYGQCPNNMMGTFSIRTVGTEK.SPHSITLRVYMRIKHVRAWIPRPLRNQPYLFKTNPNYKGNDIKCTSTSRDKITT..
QOP74937.1   LDYGQCPNNMMGTFSIRTVGTEK.SPHSITLRVYMRIKHVRAWIPRPLRNQPYLFKTNPNYKGNDIKCTSTSRDKITT..
QOP74934.1   LDYGQCPNNMMGTFSIRTVGTEK.SPHSITLRVYMRIKHVRAWIPRPLRNQPYLFKTNPNYKGNDIKCTSTSRDKITT..
QOP74929.1   LDYGQCPNNMMGTFSIRTVGTEK.SPHSITLRVYMRIKHVRAWIPRPLRNQPYLFKTNPNYKGNDIKCTSTSRDKITT..
QOP74924.1   LDYGQCPNNMMGTFSIRTVGTEK.SPHSITLRVYMRIKHVRAWIPRPLRNQPYLFKTNPNYKGNDIKCTSTSRDKITT..
QOP74921.1   LDYGQCPNNMMGTFSIRTVGTEK.SPHSITLRVYMRIKHVRAWIPRPLRNQPYLFKTNPNYKGNDIKCTSTSRDKITT..
QOP74932.1   LDYGQCPNNMMGTFSIRTVGTEK.SPHSITLRVYMRIKHVRAWIPRPLRNQPYLFKTNPNYKGNDIKCTSTSRDKITT..
QOP74931.1   LDYGQCPNNMMGTFSIRTVGTEK.SPHSITLRVYMRIKHVRAWIPRPLRNQPYLFKTNPNYKGNDIKCTSTSRDKITT..
QIL87531.1   LDYGQCPNNMMGTFSIRTVGTEK.SPHSITLRVYMRIKHVRAWIPRPLRNQPYLFKTNPNYKGNDIKCTSTSRDKITTL.
QIL87521.1   LDYGQCPNNMMGTFSIRTVGTEK.SPHSITLRVYMRIKHVRAWIPRPLRNQPYLFKTNPNYKGNDIKCTSTSRDKITTL.
QIL87523.1   LDYGQCPNNMMGTFSIRTVGTEK.SPHSITLRVYMRIKHVRAWIPRPLRNQPYLFKTNPNYKGNDIKCTSTSRDKITTL.
QIL87517.1   LDYGQCPNNMMGTFSIRTVGTEK.SPHSITLRVYMRIKHVRAWIPRPLRNQPYLFKTNPNYKGNDIKCTSTSRDKITTL.
QIL87540.1   LDYGQCPNNMMGTFSIRTVGTEK.SPHSITLRVYMRIKHVRAWIPRPLRNQPYLFKTNPNYKGNDIKCTSTSRDKITTL.
QIL87539.1   LDYGQCPNNMMGTFSIRTVGTEK.SPHSITLRVYMRIKHVRAWIPRPLRNQPYLFKTNPNYKGNDIKCTSTSRDKITTL.
QIH53921.1   LDYGQCPNNMMGTFSIRTVGTEK.SPHSITLRVYMRIKHVRAWIPRPLRNQPYLFKTNPNYKGNDIKCTSTSRDKITTL.
QIH53916.1   LDYGQCPNNMMGTFSIRTVGTEK.SPHSITLRVYMRIKHVRAWIPRPLRNQPYLFKTNPNYKGNDIKCTSTSRDKITTL.
BBH72194.1   LDYGQCPNNMMGTFSIRTVGTEK.SPHSITLRVYMRIKHVRAWIPRPLRNQPYLFKTNPNYKGNDIKCTSTSRDKITTL.
BBH72183.1   LDYGQCPNNMMGTFSIRTVGTEK.SPHSITLRVYMRIKHVRAWIPRPLRNQPYLFKTNPNYKGNDIKCTSTSRDKITTL.
BBH72107.1   LDYGQCPNNMMGTFSIRTVGTEK.SPHSITLRVYMRIKHVRAWIPRPLRNQPYLFKTNPNYKGNDIKCTSTSRDKITTL.
QWF08825.1   LDYGQCPNNMMGTFSIRTVGTEK.SPHSITLRVYMRIKHVRAWIPRPLRNQPYLFKTNPNYKGNDIKCTSTSRDKITTL.
QWF08823.1   LDYGQCPNNMMGTFSIRTVGTEK.SPHSITLRVYMRIKHVRAWIPRPLRNQPYLFKTNPNYKGNDIKCTSTSRDKITTL.
QWF08821.1   LDYGQCPNNMMGTFSIRTVGTEK.SPHSITLRVYMRIKHVRAWIPRPLRNQPYLFKTNPNYKGNDIKCTSTSRDKITTL.
QWF08818.1   LDYGQCPNNMMGTFSIRTVGTEK.SPHSITLRVYMRIKHVRAWIPRPLRNQPYLFKTNPNYKGNDIKCTSTSRDKITTL.
QWF08815.1   LDYGQCPNNMMGTFSIRTVGTEK.SPHSITLRVYMRIKHVRAWIPRPLRNQPYLFKTNPNYKGNDIKCTSTSRDKITTL.
QWF08813.1   LDYGQCPNNMMGTFSIRTVGTEK.SPHSITLRVYMRIKHVRAWIPRPLRNQPYLFKTNPNYKGNDIKCTSTSRDKITTL.
QWF08812.1   LDYGQCPNNMMGTFSIRTVGTEK.SPHSITLRVYMRIKHVRAWIPRPLRNQPYLFKTNPNYKGNDIKCTSTSRDKITTL.
QWF08810.1   LDYGQCPNNMMGTFSIRTVGTEK.SPHSITLRVYMRIKHVRAWIPRPLRNQPYLFKTNPNYKGNDIKCTSTSRDKITTL.
QWF08809.1   LDYGQCPNNMMGTFSIRTVGTEK.SPHSITLRVYMRIKHVRAWIPRPLRNQPYLFKTNPNYKGNDIKCTSTSRDKITTL.
QWF08808.1   LDYGQCPNNMMGTFSIRTVGTEK.SPHSITLRVYMRIKHVRAWIPRPLRNQPYLFKTNPNYKGNDIKCTSTSRDKITTL.
QWF08807.1   LDYGQCPNNMMGTFSIRTVGTEK.SPHSITLRVYMRIKHVRAWIPRPLRNQPYLFKTNPNYKGNDIKCTSTSRDKITTL.
QWF08797.1   LDYGQCPNNMMGTFSIRTVGTEK.SPHSITLRVYMRIKHVRAWIPRPLRNQPYLFKTNPNYKGNDIKCTSTSRDKITTL.
QWF08841.1   LDYGQCPNNMMGTFSIRTVGTEK.SPHSITLRVYMRIKHVRAWIPRPLRNQPYLFKTNPNYKGNDIKCTSTSRDKITTL.
QWF08830.1   LDYGQCPNNMMGTFSIRTVGTEK.SPHSITLRVYMRIKHVRAWIPRPLRNQPYLFKTNPNYKGNDIKCTSTSRDKITTL.
QTA73892.1   LDYGQCPNNMMGTFSIRTVGTEK.SPHSITLRVYMRIKHVRAWIPRPLRNQPYLFKTNPNYKGNDIKCTSTSRDKITTL.
QTA73901.1   LDYGQCPNNMMGTFSIRTVGTEK.SPHSITLRVYMRIKHVRAWIPRPLRNQPYLFKTNPNYKGNDIKCTSTSRDKITTL.
QWY15957.1   LDYGQCPNNMMGTFSIRTVGTEK.SPHSITLRVYMRIKHVRAWIPRPLRNQPYLFKTNPNYKGNDIKCTSTSRDKITTL.
UEP53406.1   LDYGQCPNNMMGTFSIRTVGTEK.SPHSITLRVYMRIKHVRAWIPRPLRNQPYLFKTNPNYKGNDIKCTSTSRDKITTL.
UEC49858.1   LDYGQCPNNMMGTFSIRTVGTEK.SPHSITLRVYMRIKHVRAWIPRPLRNQPYLFKTNPNYKGNDIKCTSTSRDKITTL.
QWY15931.1   LDYGQCPNNMMGTFSIRTVGTEK.SPHSITLRVYMRIKHVRAWIPRPLRNQPYLFKTNPNYKGNDIKCTSTSRDKITTL.
QWY15929.1   LDYGQCPNNMMGTFSIRTVGTEK.SPHSITLRVYMRIKHVRAWIPRPLRNQPYLFKTNPNYKGNDIKCTSTSRDKITTL.
QWY15880.1   LDYGQCPNNMMGTFSIRTVGTEK.SPHSITLRVYMRIKHVRAWIPRPLRNQPYLFKTNPNYKGNDIKCTSTSRDKITTL.
QWY15868.1   LDYGQCPNNMMGTFSIRTVGTEK.SPHSITLRVYMRIKHVRAWIPRPLRNQPYLFKTNPNYKGNDIKCTSTSRDKITTL.
BCI65368.1   LDYGQCPNNMMGTFSIRTVGTEK.SPHSITLRVYMRIKHVRAWIPRPLRNQPYLFKTNPNYKGNDIKCTSTSRDKITTL.
BCR43097.1   LDYGQCPNNMMGTFSIRTVGTEK.SPHSITLRVYMRIKHVRAWIPRPLRNQPYLFKTNPNYKGNDIKCTSTSRDKITTL.
BCG67047.1   LDYGQCPNNMMGTFSIRTVGTEK.SPHSITLRVYMRIKHVRAWIPRPLRNQPYLFKTNPNYKGNDIKCTSTSRDKITTL.
BCG67046.1   LDYGQCPNNMMGTFSIRTVGTEK.SPHSITLRVYMRIKHVRAWIPRPLRNQPYLFKTNPNYKGNDIKCTSTSRDKITTL.
BCG67041.1   LDYGQCPNNMMGTFSIRTVGTEK.SPHSITLRVYMRIKHVRAWIPRPLRNQPYLFKTNPNYKGNDIKCTSTSRDKITTL.
BCG67040.1   LDYGQCPNNMMGTFSIRTVGTEK.SPHSITLRVYMRIKHVRAWIPRPLRNQPYLFKTNPNYKGNDIKCTSTSRDKITTL.
BCG67039.1   LDYGQCPNNMMGTFSIRTVGTEK.SPHSITLRVYMRIKHVRAWIPRPLRNQPYLFKTNPNYKGNDIKCTSTSRDKITTL.
BCG67038.1   LDYGQCPNNMMGTFSIRTVGTEK.SPHSITLRVYMRIKHVRAWIPRPLRNQPYLFKTNPNYKGNDIKCTSTSRDKITTL.
BCG67032.1   LDYGQCPNNMMGTFSIRTVGTEK.SPHSITLRVYMRIKHVRAWIPRPLRNQPYLFKTNPNYKGNDIKCTSTSRDKITTL.
BCG67030.1   LDYGQCPNNMMGTFSIRTVGTEK.SPHSITLRVYMRIKHVRAWIPRPLRNQPYLFKTNPNYKGNDIKCTSTSRDKITTL.
BCG66995.1   LDYGQCPNNMMGTFSIRTVGTEK.SPHSITLRVYMRIKHVRAWIPRPLRNQPYLFKTNPNYKGNDIKCTSTSRDKITTL.
BCG66994.1   LDYGQCPNNMMGTFSIRTVGTEK.SPHSITLRVYMRIKHVRAWIPRPLRNQPYLFKTNPNYKGNDIKCTSTSRDKITTL.
QWY15757.1   LDYGQCPNNMMGTFSIRTVGTEK.SPHSITLRVYMRIKHVRAWIPRPLRNQPYLFKTNPNYKGNDIKCTSTSRDKITTL.
QWY15756.1   LDYGQCPNNMMGTFSIRTVGTEK.SPHSITLRVYMRIKHVRAWIPRPLRNQPYLFKTNPNYKGNDIKCTSTSRDKITTL.
QWY15748.1   LDYGQCPNNMMGTFSIRTVGTEK.SPHSITLRVYMRIKHVRAWIPRPLRNQPYLFKTNPNYKGNDIKCTSTSRDKITTL.
QWY15747.1   LDYGQCPNNMMGTFSIRTVGTEK.SPHSITLRVYMRIKHVRAWIPRPLRNQPYLFKTNPNYKGNDIKCTSTSRDKITTL.
QWY15720.1   LDYGQCPNNMMGTFSIRTVGTEK.SPHSITLRVYMRIKHVRAWIPRPLRNQPYLFKTNPNYKGNDIKCTSTSRDKITTL.
QWY15717.1   LDYGQCPNNMMGTFSIRTVGTEK.SPHSITLRVYMRIKHVRAWIPRPLRNQPYLFKTNPNYKGNDIKCTSTSRDKITTL.
QWY15697.1   LDYGQCPNNMMGTFSIRTVGTEK.SPHSITLRVYMRIKHVRAWIPRPLRNQPYLFKTNPNYKGNDIKCTSTSRDKITTL.
QWY15689.1   LDYGQCPNNMMGTFSIRTVGTEK.SPHSITLRVYMRIKHVRAWIPRPLRNQPYLFKTNPNYKGNDIKCTSTSRDKITTL.
QWY15761.1   LDYGQCPNNMMGTFSIRTVGTEK.SPHSITLRVYMRIKHVRAWIPRPLRNQPYLFKTNPNYKGNDIKCTSTSRDKITTL.
QWY15743.1   LDYGQCPNNMMGTFSIRTVGTEK.SPHSITLRVYMRIKHVRAWIPRPLRNQPYLFKTNPNYKGNDIKCTSTSRDKITTL.
QWY15759.1   LDYGQCPNNMMGTFSIRTVGTEK.SPHSITLRVYMRIKHVRAWIPRPLRNQPYLFKTNPNYKGNDIKCTSTSRDKITTL.
QWY15758.1   LDYGQCPNNMMGTFSIRTVGTEK.SPHSITLRVYMRIKHVRAWIPRPLRNQPYLFKTNPNYKGNDIKCTSTSRDKITTL.
QWY15677.1   LDYGQCPNNMMGTFSIRTVGTEK.SPHSITLRVYMRIKHVRAWIPRPLRNQPYLFKTNPNYKGNDIKCTSTSRDKITTL.
QWY15675.1   LDYGQCPNNMMGTFSIRTVGTEK.SPHSITLRVYMRIKHVRAWIPRPLRNQPYLFKTNPNYKGNDIKCTSTSRDKITTL.
QWY15776.1   LDYGQCPNNMMGTFSIRTVGTEK.SPHSITLRVYMRIKHVRAWIPRPLRNQPYLFKTNPNYKGNDIKCTSTSRDKITTL.
QWY15772.1   LDYGQCPNNMMGTFSIRTVGTEK.SPHSITLRVYMRIKHVRAWIPRPLRNQPYLFKTNPNYKGNDIKCTSTSRDKITTL.
QWY15770.1   LDYGQCPNNMMGTFSIRTVGTEK.SPHSITLRVYMRIKHVRAWIPRPLRNQPYLFKTNPNYKGNDIKCTSTSRDKITTL.
QWY15762.1   LDYGQCPNNMMGTFSIRTVGTEK.SPHSITLRVYMRIKHVRAWIPRPLRNQPYLFKTNPNYKGNDIKCTSTSRDKITTL.
```

```
QWY15732.1   LDYGQCPNNMMGTFSIRTVGTEK.SPHSITLRVYMRIKHVRAWIPRPLRNQPYLFKTNPNYKGNDIKCTSTSRDKITTL.
QWY15731.1   LDYGQCPNNMMGTFSIRTVGTEK.SPHSITLRVYMRIKHVRAWIPRPLRNQPYLFKTNPNYKGNDIKCTSTSRDKITTL.
QWY15780.1   LDYGQCPNNMMGTFSIRTVGTEK.SPHSITLRVYMRIKHVRAWIPRPLRNQPYLFKTNPNYKGNDIKCTSTSRDKITTL.
QWY15779.1   LDYGQCPNNMMGTFSIRTVGTEK.SPHSITLRVYMRIKHVRAWIPRPLRNQPYLFKTNPNYKGNDIKCTSTSRDKITTL.
BCG67006.1   LDYGQCPNNMMGTFSIRTVGTEK.SPHSITLRVYMRIKHVRAWIPRPLRNQPYLFKTNPNYKGNDIKCTSTSRDKITTL.
BCG67004.1   LDYGQCPNNMMGTFSIRTVGTEK.SPHSITLRVYMRIKHVRAWIPRPLRNQPYLFKTNPNYKGNDIKCTSTSRDKITTL.
BCG67019.1   LDYGQCPNNMMGTFSIRTVGTEK.SPHSITLRVYMRIKHVRAWIPRPLRNQPYLFKTNPNYKGNDIKCTSTSRDKITTL.
BCG67002.1   LDYGQCPNNMMGTFSIRTVGTEK.SPHSITLRVYMRIKHVRAWIPRPLRNQPYLFKTNPNYKGNDIKCTSTSRDKITTL.
BCG67036.1   LDYGQCPNNMMGTFSIRTVGTEK.SPHSITLRVYMRIKHVRAWIPRPLRNQPYLFKTNPNYKGNDIKCTSTSRDKITTL.
BCG67035.1   LDYGQCPNNMMGTFSIRTVGTEK.SPHSITLRVYMRIKHVRAWIPRPLRNQPYLFKTNPNYKGNDIKCTSTSRDKITTL.
QWY15785.1   LDYGQCPNNMMGTFSIRTVGTEK.SPHSITLRVYMRIKHVRAWIPRPLRNQPYLFKTNPNYKGNDIKCTSTSRDKITTL.
QWY15784.1   LDYGQCPNNMMGTFSIRTVGTEK.SPHSITLRVYMRIKHVRAWIPRPLRNQPYLFKTNPNYKGNDIKCTSTSRDKITTL.
QWY15789.1   LDYGQCPNNMMGTFSIRTVGTEK.SPHSITLRVYMRIKHVRAWIPRPLRNQPYLFKTNPNYKGNDIKCTSTSRDKITTL.
QYC65159.1   LDYGQCPNNMMGTFSIRTVGTEK.SPHSITLRVYMRIKHVRAWIPRPLRNQPYLFKTNPNYKGNDIKCTSTSRDKITTL.
BCR43101.1   LDYGQCPNNMMGTFSIRTVGTEK.SPHSITLRVYMRIKHVRAWIPRPLRNQPYLFKTNPNYKGNDIKCTSTSRDKITTL.
BCR43098.1   LDYGQCPNNMMGTFSIRTVGTEK.SPHSITLRVYMRIKHVRAWIPRPLRNQPYLFKTNPNYKGNDIKCTSTSRDKITTL.
QWY15873.1   LDYGQCPNNMMGTFSIRTVGTEK.SPHSITLRVYMRIKHVRAWIPRPLRNQPYLFKTNPNYKGNDIKCTSTSRDKITTL.
QWY15865.1   LDYGQCPNNMMGTFSIRTVGTEK.SPHSITLRVYMRIKHVRAWIPRPLRNQPYLFKTNPNYKGNDIKCTSTSRDKITTL.
QWY15900.1   LDYGQCPNNMMGTFSIRTVGTEK.SPHSITLRVYMRIKHVRAWIPRPLRNQPYLFKTNPNYKGNDIKCTSTSRDKITTL.
BCG67049.1   LDYGQCPNNMMGTFSIRTVGTEK.SPHSITLRVYMRIKHVRAWIPRPLRNQPYLFKTNPNYKGNDIKCTSTSRDKITTL.
UEC49853.1   LDYGQCPNNMMGTFSIRTVGTEK.SPHSITLRVYMRIKHVRAWIPRPLRNQPYLFKTNPNYKGNDIKCTSTSRDKITTL.
UEC49848.1   LDYGQCPNNMMGTFSIRTVGTEK.SPHSITLRVYMRIKHVRAWIPRPLRNQPYLFKTNPNYKGNDIKCTSTSRDKITTL.
QTA73899.1   LDYGQCPNNMMGTFSIRTVGTEK.SPHSITLRVYMRIKHVRAWIPRPLRNQPYLFKTNPNYKGNDIKCTSTSRDKITTL.
QTA73898.1   LDYGQCPNNMMGTFSIRTVGTEK.SPHSITLRVYMRIKHVRAWIPRPLRNQPYLFKTNPNYKGNDIKCTSTSRDKITTL.
QTA73905.1   LDYGQCPNNMMGTFSIRTVGTEK.SPHSITLRVYMRIKHVRAWIPRPLRNQPYLFKTNPNYKGNDIKCTSTSRDKITTL.
QTA73904.1   LDYGQCPNNMMGTFSIRTVGTEK.SPHSITLRVYMRIKHVRAWIPRPLRNQPYLFKTNPNYKGNDIKCTSTSRDKITTL.
QTA73909.1   LDYGQCPNNMMGTFSIRTVGTEK.SPHSITLRVYMRIKHVRAWIPRPLRNQPYLFKTNPNYKGNDIKCTSTSRDKITTL.
QTA73908.1   LDYGQCPNNMMGTFSIRTVGTEK.SPHSITLRVYMRIKHVRAWIPRPLRNQPYLFKTNPNYKGNDIKCTSTSRDKITTL.
UEP53408.1   LDYGQCPNNMMGTFSIRTVGTEK.SPHSITLRVYMRIKHVRAWIPRPLRNQPYLFKTNPNYKGNDIKCTSTSRDKITTL.
QWF08839.1   LDYGQCPNNMMGTFSIRTVGTEK.SPHSITLRVYMRIKHVRAWIPRPLRNQPYLFKTNPNYKGNDIKCTSTSRDKITTL.
QWF08799.1   LDYGQCPNNMMGTFSIRTVGTEK.SPHSITLRVYMRIKHVRAWIPRPLRNQPYLFKTNPNYKGNDIKCTSTSRDKITTL.
QWY15771.1   LDYGQCPNNMMGTFSIRTVGTEK.SPHSITLRVYMRIKHVRAWIPRPLRNQPYLFKTNPNYKGNDIKCTSTSKDKITTL.
QTA73891.1   LDYGQCPNNMMGTFSIRTVGTEK.SPHSITLRVYMRIKHVRAWIPRPLRNHPYLFKTNPNYKGNDIKCTSTSRDKITTL.
QWY15670.1   LDYGQCPNNMMGTFSIRTVGTEK.SPHSITLRVYMRIKHVRAWIPRPLRNHPYLFKTNPNYKGNDIKCTSTSRDKITTL.
BBH72149.1   LDYGQCPNNMMGTFSIRTVGTEK.SPHSITLRVYMRIKHVRAWIPRPLRNQPYLFKTNPNYKGNDIKCTSTSRDKITTL.
BBH72143.1   LDYGQCPNNMMGTFSIRTVGTEK.SPHSITLRVYMRIKHVRAWIPRPLRNQPYLFKTNPNYKGNDIKCTSTSRDKITTL.
BBH72163.1   LDYGQCPNNLMGTFSIRTVGTEK.SPHSITLRVYMRIKHVRAWIPRPLRNQPYLFKTNPNYKGNDIKCTSTSRDKITTL.
QTA73895.1   LDYGQCPNNMMGTFSIRTVGTEK.SPHSITLRVYMRIKHVRAWIPRPLRNQPYLFKTNPNYKGNDIKCTSTSRDKIPTL.
QWY15674.1   LDYGQCPNNMMGTFSIRTVGTEK.SPHSITLRVYMRIKHVRAWIPRPLRNQPYLFKTNPNYKGNDIKCTSTSRDKIPTL.
QWY15710.1   LDYGQCPNNMMGTFSIRTVGTEK.SPHSITLRVYMRIKHVRAWIPRPLKNQPYLFKTNPNYKGNDIKCTSTSRDKITTL.
QWY15709.1   LDYGQCPNNMMGTFSIRTVGTEK.SPHSITLRVYMRIKHVRAWIPRPLKNQPYLFKTNPNYKGNDIKCTSTSRDKITTL.
QWF08934.1   LDYGQCPNNMMGTFSIRTVGTEK.SPHSITLRVYMRIKHVRAWIPRPLRNQPYLFKTNPNYKGNDIKCTSTSRDKITTL.
QWF08836.1   LDYGQCPNNMMGTFSIRTVGTEK.SPHSITLRVYMRIKHVRAWIPRPLRNQPYLFKTNPNYKGNDIKCTSTSRDKITTL.
QLH02014.1   LDYGQCPNNMMGTFSIRTVGTEK.SPHSITLRVYMRIKHVRAWIPRPLRNQPYLFKTNPNYKGNDIKCTSTSRDKITTL.
QWF08796.1   LDYGQCPNNMMGTFSIRTVGTEK.SPHSITLRVYMRIKHVRAWIPRPLRNQPYLFKTNPNYKGNDIMCTSTSRDKITTL.
QYC65090.1   LDYGQCPNNMMGTFSIRTVGTEK.SPHSITLRVYMRIKHVRAWIPRPLRNQPYLFKTNPNYKGNDIKCTSTSRDKITTL.
QTA73900.1   LDYGQCPNNMMGTFSIRTVGTEK.SPHSITLRVYMRIKHVRAWIPRPLRNQPYLFKTNPNYKGNDIKCTSTSRDKITTL.
QWY15679.1   LDYGQCPNNMMGTFSIRTVGTEK.SPHSITLRVYMRIKHVRAWIPRPLRNQPYLFKTNPNYKGNDIKCTSTSRDKITTL.
QWY15751.1   LEYGQCPNNMMGTFSIRTVGAEK.SPHSITLRVYMRIKHVRAWIPRPLRNQPYLFKTNPNYKGNDIKCTSTSRDKITTL.
QWF08795.1   LDYGQCPNNMMGTFSIRTVGTEK.SPHSITLRVYMRIKHVRAWIPRPVRNQPYLFKTNPNYKGNDIKCTSTSRDKITTL.
QWF08789.1   LEYGQCPNNMMGTFSIRTVGTEK.SPHSITLRVYMRIKHVRAWIPRPVRNQPYLFKTNPNYKGNDIKCTSTSRDKITTL.
QYC65158.1   LDYGQCPNNMMGTFSIRTVGIEK.SPHSITLRVYMRIKHVRAWIPRPLRNQPYLFKTNPNYKGNDIKCTSTSRDKITTL.
QYC65109.1   LDYGQCPNNMMGTFSIRTVGIEK.SPHSITLRVYMRIKHVRAWIPRPLRNQPYLFKTNPNYKGNDIKCTSTSRDKITTL.
QWF08883.1   LDYGQCPNNMMGTFSIRTVGIEK.SPHSITLRVYMRIKHVRAWIPRPLRNQPYLFKTNPNYKGNDIKCTSTSRDKITTL.
QWF08866.1   LDYGQCPNNMMGTFSIRTVGTEK.SPHSITLRVYMRIKHVRAWIPRPLRNQPYLFKTNPNYKGNDIKCTSTSRDKITTL.
QWF08833.1   LDYGQCPNNMMGTFSIRTVGTEK.SPHSITLRVYMRIKHVRAWIPRPLRNQPYLFKTNPNYKGNDIKCTSTSRDKITTL.
QWF08801.1   LDYGQCPNNMMGTFSIRTVGTEK.SPHSITLRVYMRIKHVRAWIPRPLRNQPYLFKTNPNYKGNDIKCTSTSRDKITTL.
QWF08842.1   LDYGQCPNNMMGTFSIRTVGTEK.SPHSITLRVYMRIKHVRAWIPRPLRNQPYLFKTNPNYKGNDIKCTSTSRDKITTL.
QWF08806.1   LDYGQCPNNMMGTFSIRTVGTEK.SPHSITLRVYMRIKHVRAWIPRPLRNQPYLFKTNPNYKGNDIKCTSTSRDKITTL.
QYC65065.1   LDYGQCPNNMMGTFSIRTVGTEK.SPHSITLRVYMRIKHVRAWIPRPLRNQPYLFKTNPNYKGSDIKCTSTSRDKITTL.
QYC65044.1   LDYGQCPNNMMGTFSIRTVGTEK.SPHSITLRVYMRIKHVRAWIPRPLRNQPYLFKTNPNYKGSDIKCTSTSRDKITTL.
QWY15863.1   LDYGQCPNNMMGTFSIRTVGTEK.SPHSITLRVYMRIKHVRAWIPRPLRNQPYLFKTNPNYKGIDIKCTSTSRDKITTL.
QTA73916.1   LDYGQCPNNMMGTFSIRTVGTEK.SPHSITLRVYMRIKHVRAWIPRPLRNQPYLFKTNPNYKGNDIKCTSTSRDKITTL.
BCG66990.1   LDYGQCPNNMMGTFSIRTVGTEK.SPHSITLRVYMRIKHVRAWIPRPLRNQPYLFKTNPNYKGNDIKCTSTSRDKITTL.
QTA73915.1   LDYGQCPNNMMGTFSIRTVGTEK.SPHSITLRVYMRIKHVRAWIPRPLRNQPYLFKTNPNYKGNDIKCTSTSRDKITTL.
QWY15932.1   LDYGQCPNNMMGTFSIRTVGTEK.SPHSITLRVYMRIKHVRAWIPRPLRNQPYLFKTNPNYKGNDIKCTSTSRDKITTL.
QWY15867.1   LDYGQCPNNMMGTFSIRTVGTEK.SPHSITLRVYMRIKHVRAWIPRPLRNQPYLFKTNPNYKGNDIKCTSTSRDKITTL.
QWY15859.1   LDYGQCPNNMMGTFSIRTVGTEK.SPHSITLRVYMRIKHVRAWIPRPLRNQPYLFKTNPNYKGNDIKCTSTSRDKITTL.
QWY15858.1   LDYGQCPNNMMGTFSIRTVGTEK.SPHSITLRVYMRIKHVRAWIPRPLRNQPYLFKTNPNYKGNDIKCTSTSRDKITTL.
QWY15782.1   LDYGQCPNNMMGTFSIRTVGTEK.SPHSITLRVYMRIKHVRAWIPRPLRNQPYLFKTNPNYKGNDIKCTSTSRDKITTL.
QWY15781.1   LDYGQCPNNMMGTFSIRTVGTEK.SPHSITLRVYMRIKHVRAWIPRPLRNQPYLFKTNPNYKGNDIKCTSTSRDKITTL.
BCI65355.1   LDYGQCPNNMMGTFSIRTVGTEK.SPHSITLRVYMRIKHVRAWIPRPLRNQPYLFKTNPNYKGNDIKCTSTSRDKITTL.
BCI65374.1   LDYGQCPNNMMGTFSIRTVGTEK.SPHSITLRVYMRIKHVRAWIPRPLRNQPYLFKTNPNYKGNDIKCTSTSRDKITTL.
BCI65373.1   LDYGQCPNNMMGTFSIRTVGTEK.SPHSITLRVYMRIKHVRAWIPRPLRNQPYLFKTNPNYKGNDIKCTSTSRDKITTL.
BCI65372.1   LDYGQCPNNMMGTFSIRTVGTEK.SPHSITLRVYMRIKHVRAWIPRPLRNQPYLFKTNPNYKGNDIKCTSTSRDKITTL.
BCI65371.1   LDYGQCPNNMMGTFSIRTVGTEK.SPHSITLRVYMRIKHVRAWIPRPLRNQPYLFKTNPNYKGNDIKCTSTSRDKITTL.
BCI65369.1   LDYGQCPNNMMGTFSIRTVGTEK.SPHSITLRVYMRIKHVRAWIPRPLRNQPYLFKTNPNYKGNDIKCTSTSRDKITTL.
BCI65367.1   LDYGQCPNNMMGTFSIRTVGTEK.SPHSITLRVYMRIKHVRAWIPRPLRNQPYLFKTNPNYKGNDIKCTSTSRDKITTL.
BCI65365.1   LDYGQCPNNMMGTFSIRTVGTEK.SPHSITLRVYMRIKHVRAWIPRPLRNQPYLFKTNPNYKGNDIKCTSTSRDKITTL.
BCI65364.1   LDYGQCPNNMMGTFSIRTVGTEK.SPHSITLRVYMRIKHVRAWIPRPLRNQPYLFKTNPNYKGNDIKCTSTSRDKITTL.
BCI65363.1   LDYGQCPNNMMGTFSIRTVGTEK.SPHSITLRVYMRIKHVRAWIPRPLRNQPYLFKTNPNYKGNDIKCTSTSRDKITTL.
BCI65362.1   LDYGQCPNNMMGTFSIRTVGTEK.SPHSITLRVYMRIKHVRAWIPRPLRNQPYLFKTNPNYKGNDIKCTSTSRDKITTL.
BCI65361.1   LDYGQCPNNMMGTFSIRTVGTEK.SPHSITLRVYMRIKHVRAWIPRPLRNQPYLFKTNPNYKGNDIKCTSTSRDKITTL.
BCI65360.1   LDYGQCPNNMMGTFSIRTVGTEK.SPHSITLRVYMRIKHVRAWIPRPLRNQPYLFKTNPNYKGNDIKCTSTSRDKITTL.
BCI65359.1   LDYGQCPNNMMGTFSIRTVGTEK.SPHSITLRVYMRIKHVRAWIPRPLRNQPYLFKTNPNYKGNDIKCTSTSRDKITTL.
BCI65358.1   LDYGQCPNNMMGTFSIRTVGTEK.SPHSITLRVYMRIKHVRAWIPRPLRNQPYLFKTNPNYKGNDIKCTSTSRDKITTL.
BCI65356.1   LDYGQCPNNMMGTFSIRTVGTEK.SPHSITLRVYMRIKHVRAWIPRPLRNQPYLFKTNPNYKGNDIKCTSTSRDKITTL.
BCI65354.1   LDYGQCPNNMMGTFSIRTVGTEK.SPHSITLRVYMRIKHVRAWIPRPLRNQPYLFKTNPNYKGNDIKCTSTSRDKITTL.
BCI65353.1   LDYGQCPNNMMGTFSIRTVGTEK.SPHSITLRVYMRIKHVRAWIPRPLRNQPYLFKTNPNYKGNDIKCTSTSRDKITTL.
BCI65352.1   LDYGQCPNNMMGTFSIRTVGTEK.SPHSITLRVYMRIKHVRAWIPRPLRNQPYLFKTNPNYKGNDIKCTSTSRDKITTL.
BCI65351.1   LDYGQCPNNMMGTFSIRTVGTEK.SPHSITLRVYMRIKHVRAWIPRPLRNQPYLFKTNPNYKGNDIKCTSTSRDKITTL.
BCI65350.1   LDYGQCPNNMMGTFSIRTVGTEK.SPHSITLRVYMRIKHVRAWIPRPLRNQPYLFKTNPNYKGNDIKCTSTSRDKITTL.
BCI65349.1   LDYGQCPNNMMGTFSIRTVGTEK.SPHSITLRVYMRIKHVRAWIPRPLRNQPYLFKTNPNYKGNDIKCTSTSRDKITTL.
BCI65348.1   LDYGQCPNNMMGTFSIRTVGTEK.SPHSITLRVYMRIKHVRAWIPRPLRNQPYLFKTNPNYKGNDIKCTSTSRDKITTL.
BCI65347.1   LDYGQCPNNMMGTFSIRTVGTEK.SPHSITLRVYMRIKHVRAWIPRPLRNQPYLFKTNPNYKGNDIKCTSTSRDKITTL.
BCI65346.1   LDYGQCPNNMMGTFSIRTVGTEK.SPHSITLRVYMRIKHVRAWIPRPLRNQPYLFKTNPNYKGNDIKCTSTSRDKITTL.
BCI65345.1   LDYGQCPNNMMGTFSIRTVGTEK.SPHSITLRVYMRIKHVRAWIPRPLRNQPYLFKTNPNYKGNDIKCTSTSRDKITTL.
BCI65344.1   LDYGQCPNNMMGTFSIRTVGTEK.SPHSITLRVYMRIKHVRAWIPRPLRNQPYLFKTNPNYKGNDIKCTSTSRDKITTL.
BCI65343.1   LDYGQCPNNMMGTFSIRTVGTEK.SPHSITLRVYMRIKHVRAWIPRPLRNQPYLFKTNPNYKGNDIKCTSTSRDKITTL.
BCI65342.1   LDYGQCPNNMMGTFSIRTVGTEK.SPHSITLRVYMRIKHVRAWIPRPLRNQPYLFKTNPNYKGNDIKCTSTSRDKITTL.
BCI65341.1   LDYGQCPNNMMGTFSIRTVGTEK.SPHSITLRVYMRIKHVRAWIPRPLRNQPYLFKTNPNYKGNDIKCTSTSRDKITTL.
BCI65340.1   LDYGQCPNNMMGTFSIRTVGTEK.SPHSITLRVYMRIKHVRAWIPRPLRNQPYLFKTNPNYKGNDIKCTSTSRDKITTL.
BCI65339.1   LDYGQCPNNMMGTFSIRTVGTEK.SPHSITLRVYMRIKHVRAWIPRPLRNQPYLFKTNPNYKGNDIKCTSTSRDKITTL.
BCI65338.1   LDYGQCPNNMMGTFSIRTVGTEK.SPHSITLRVYMRIKHVRAWIPRPLRNQPYLFKTNPNYKGNDIKCTSTSRDKITTL.
BCI65337.1   LDYGQCPNNMMGTFSIRTVGTEK.SPHSITLRVYMRIKHVRAWIPRPLRNQPYLFKTNPNYKGNDIKCTSTSRDKITTL.
BCI65336.1   LDYGQCPNNMMGTFSIRTVGTEK.SPHSITLRVYMRIKHVRAWIPRPLRNQPYLFKTNPNYKGNDIKCTSTSRDKITTL.
BCI65335.1   LDYGQCPNNMMGTFSIRTVGTEK.SPHSITLRVYMRIKHVRAWIPRPLRNQPYLFKTNPNYKGNDIKCTSTSRDKITTL.
BCI65334.1   LDYGQCPNNMMGTFSIRTVGTEK.SPHSITLRVYMRIKHVRAWIPRPLRNQPYLFKTNPNYKGNDIKCTSTSRDKITTL.
BCI65333.1   LDYGQCPNNMMGTFSIRTVGTEK.SPHSITLRVYMRIKHVRAWIPRPLRNQPYLFKTNPNYKGNDIKCTSTSRDKITTL.
BCI65332.1   LDYGQCPNNMMGTFSIRTVGTEK.SPHSITLRVYMRIKHVRAWIPRPLRNQPYLFKTNPNYKGNDIKCTSTSRDKITTL.
BCI65331.1   LDYGQCPNNMMGTFSIRTVGTEK.SPHSITLRVYMRIKHVRAWIPRPLRNQPYLFKTNPNYKGNDIKCTSTSRDKITTL.
BCI65330.1   LDYGQCPNNMMGTFSIRTVGTEK.SPHSITLRVYMRIKHVRAWIPRPLRNQPYLFKTNPNYKGNDIKCTSTSRDKITTL.
BCI65328.1   LDYGQCPNNMMGTFSIRTVGTEK.SPHSITLRVYMRIKHVRAWIPRPLRNQPYLFKTNPNYKGNDIKCTSTSRDKITTL.
BCI65327.1   LDYGQCPNNMMGTFSIRTVGTEK.SPHSITLRVYMRIKHVRAWIPRPLRNQPYLFKTNPNYKGNDIKCTSTSRDKITTL.
BCI65326.1   LDYGQCPNNMMGTFSIRTVGTEK.SPHSITLRVYMRIKHVRAWIPRPLRNQPYLFKTNPNYKGNDIKCTSTSRDKITTL.
BCI65325.1   LDYGQCPNNMMGTFSIRTVGTEK.SPHSITLRVYMRIKHVRAWIPRPLRNQPYLFKTNPNYKGNDIKCTSTSRDKITTL.
BCI65324.1   LDYGQCPNNMMGTFSIRTVGTEK.SPHSITLRVYMRIKHVRAWIPRPLRNQPYLFKTNPNYKGNDIKCTSTSRDKITTL.
BCI65323.1   LDYGQCPNNMMGTFSIRTVGTEK.SPHSITLRVYMRIKHVRAWIPRPLRNQPYLFKTNPNYKGNDIKCTSTSRDKITTL.
BCI65322.1   LDYGQCPNNMMGTFSIRTVGTEK.SPHSITLRVYMRIKHVRAWIPRPLRNQPYLFKTNPNYKGNDIKCTSTSRDKITTL.
BCI65321.1   LDYGQCPNNMMGTFSIRTVGTEK.SPHSITLRVYMRIKHVRAWIPRPLRNQPYLFKTNPNYKGNDIKCTSTSRDKITTL.
BCI65320.1   LDYGQCPNNMMGTFSIRTVGTEK.SPHSITLRVYMRIKHVRAWIPRPLRNQPYLFKTNPNYKGNDIKCTSTSRDKITTL.
BCI65319.1   LDYGQCPNNMMGTFSIRTVGTEK.SPHSITLRVYMRIKHVRAWIPRPLRNQPYLFKTNPNYKGNDIKCTSTSRDKITTL.
BCI65318.1   LDYGQCPNNMMGTFSIRTVGTEK.SPHSITLRVYMRIKHVRAWIPRPLRNQPYLFKTNPNYKGNDIKCTSTSRDKITTL.
BCI65317.1   LDYGQCPNNMMGTFSIRTVGTEK.SPHSITLRVYMRIKHVRAWIPRPLRNQPYLFKTNPNYKGNDIKCTSTSRDKITTL.
QYC65119.1   LDYGQCPNNMMGTFSIRTVGTEK.SPHSITLRVYMRIKHVRAWIPRPLRNQPYLFKTNPNYKGNDIKCTSTSRDKITTL.
QWF08905.1   LDYGQCPNNMMGTFSIRTVGTEK.SPHSITLRVYMRIKHVRAWIPRPLRNQPYLFKTNPNYKGNDIKCTSTSRDKITTL.
QWF08814.1   LDYGQCPNNMMGTFSIRTVGTEK.SPHSITLRVYMRIKHVRAWIPRPLRNQPYLFKTNPNYKGNDIKCTSTSRDKITTL.
QWF08776.1   LDYGQCPNNMMGTFSIRTVGTEK.SPHSITLRVYMRIKHVRAWIPRPLRNQPYLFKTNPNYKGNDIKCTSTSRDKITTL.
BCI65370.1   LDYGQCPNNMMGTFSIRTVGTEK.SPHSITLRVYMRIKHVRAWIPRPLRSQPYLFKTNPNYKGNDIKCTSTSRDKITTL.
BCG67048.1   LDYGQCPNNMMGTFSIRTVGTEK.SPHSITLRVYMRIKHVRAWIPRPLRNQPYLFKTNPNYKGNDIKCTSTSRDKITTL.
QWY15933.1   LDYGQCPNNMMGTFSIRTVGTEK.SPHSITLRVYMRIKHVRAWIPRPLRNQPYLFKTNPNYKGNDIKCTSTSRDKITTL.
UEP53404.1   LDYGQCPNNMMGTFSIRTVGTEK.SPHSITLRVYMRIKHVRAWIPRPLRNQPYLFKTNPNYKGNDIKCTSTSRDKITTL.
UEC49857.1   LDYGQCPNNMMGTFSIRTVGTEK.SPHSITLRVYMRIKHVRAWIPRPLRNQPYLFKTNPNYKGNDIKCTSTSRDKITTL.
QYC65076.1   LDYGQCPNNMMGTFSIRTVGTEK.SPHSITLRVYMRIKHVRAWIPRPLRNQPYLFKTNPNYKGNDIKCTSTSRDKITTL.
QWF08819.1   LDYGQCPNNMMGTFSIRTVGTEK.SPHSITLRVYMRIKHVRAWIPRPLRNQPYLFKTNPNYKGNDIKCTSTSRDKITTL.
QYC65082.1   LDYGQCPNNMMGTFSIRTVGTEK.SPHSITLRVYMRIKHVRAWIPRPLRNQPYLFKTNPNYKGNDIKCTSTSRDKITTL.
QWY15927.1   LDYGQCPNNMMGTFSVRTVGTEK.SPHSITLRVYMRIKHVRAWIPRPLRNQPYLFKTNPNYKGNDIKCTSTSRDKITTL.
QOP74944.1   LDYGQCPNNMMGTFSVRTVGTEK.SPHSITLRVYMRIKHVRAWIPRPLRNQPYLFKTNPNYKGNDIKCTSTSRDKITT..
QYC65139.1   LDYGQCPNNMMGTFSVRTVGTEK.SPHSITLRVYMRIKHVRAWIPRPLRNQPYLFKTNPNYKGNDIKCTSTSRDKITTL.
QYC65136.1   LDYGQCPNNMMGTFSVRTVGTEK.SPHSITLRVYMRIKHVRAWIPRPLRNQPYLFKTNPNYKGNDIKCTSTSRDKITTL.
QYC65130.1   LDYGQCPNNMMGTFSVRTVGTEK.SPHSITLRVYMRIKHVRAWIPRPLRNQPYLFKTNPNYKGNDIKCTSTSRDKITTL.
QYC65115.1   LDYGQCPNNMMGTFSVRTVGTEK.SPHSITLRVYMRIKHVRAWIPRPLRNQPYLFKTNPNYKGNDIKCTSTSRDKITTL.
QYC65114.1   LDYNQCPNNMMGTFSVRTVGTEK.SPHSITLRVYMRIKHVRAWIPRPLRNQPYLFKTNPNYKGNDIKCTSTSRDKITTL.
QYC65105.1   LDYGQCPNNMMGTFSVRTVGTEK.SPHSITLRVYMRIKHVRAWIPRPLRNQPYLFKTNPNYKGNDIKCTSTSRDKITTL.
```

QWF08949.1	LDYGQCPNNMMGTFSVRTVGTEK.SPHSITLRVYMRIKHVRAWIPRPLRNQPYLFKTNPNYKGNDIKCTSTSRDKITTL.
QWF08928.1	LDYGQCPNNMMGTFSVRTVGTEK.SPHSITLRVYMRIKHVRAWIPRPLRNQPYLFKTNPNYKGNDIKCTSTSRDKITTL.
QWY15921.1	LDYGQCPNNMMGTFSVRTVGTEK.SPHSITLRVYMRIKHVRAWIPRPLRNQPYLFKTNPNYKGNDIKCTSTSRDKITTL.
QWY15919.1	LDYGQCPNNMMGTFSVRTVGTEK.SPHSITLRVYMRIKHVRAWIPRPLRNQPYLFKTNPNYKGNDIKCTSTSRDKITTL.
QYC65102.1	LDYGQCPNNMMGTFSVRTVGTEK.SPHSITLRVYMRIKHVRAWIPRPLRNQPYLFKTNPNYKGSDIKCTSTSRDKITTL.
BCR43082.1	LDYGQCPNNMMGTFSVRTVGTEK.SPHSITLRVYMRIKHVRAWIPRPLRNQPYLFKTNPNYKGNDIKCTSTSRDKITTL.
QWY15713.1	LDYGQCPNNMMGTFSIRTVGTEK.SPHSITLRVYMRIKHVRAWIPRPLRNQPNLFKTNPNYKGNDIKWTSTSRDKITTL.
BCI65329.1	LDYGQCPNNMMGTFSIRTVGTEK.SPHSITLRVYMRIKHVRAWIPRPLRNQPYLFKTNPNYKGNDIKCTSTSRDKITTL.
QWF08887.1	LDYGQCPNNMMGTFSIRTVGTEK.SPHSITLRVYMRIKHVRAWIPRPLRNQPYLFKTNPNYKGNDIKCTSTSRDKITTL.
QYC65036.1	LDYGQCPNNMMGTFSIRTVGTEK.SPHSITLRVYMRIKHVRAWIPRPLRNQPYLFKTNPNYKGNDIKCTSTSRDKITTL.
QTA73913.1	LDYGQCPNNMMGTFSIRTVGTEK.SPHSITLRVYMRIKHVRAWIPRPLRNQPYLFKTNPNYKGNDIKCTSTSRDKITTL.
QWY15837.1	LDYGQCPNNMMGTFSIRTVGTEK.SPHSITLRVYMRIKHVRAWIPRPLRNQPYLFKTNPNYKGNDIKCTSTSRDKITTL.
QWY15738.1	LDYGQCPNNMMGTFSIRTVGTEK.SPHSITLRVYMRIKHVRAWIPRPLRNQPYLFKTNPNYKGNDIKCTSTSRDKITTL.
QTA73911.1	LDYGQCPNNMMGTFSIRTVGTEK.SPHSITLRVYMRIKHVRAWIPRPLRNQPYLFKTNPNYKGNDIKCTSTSRDKITTL.
QTA73910.1	LDYGQCPNNMMGTFSIRTVGTEK.SPHSITLRVYMRIKHVRAWIPRPLRNQPYLFKTNPNYKGNDIKCTSTSRDKITTL.
QWY15916.1	LDYGQCPNNMMGTFSIRTVGTEK.SPHSITLRVYMRIKHVRAWIPRPLRNQPYLFKTNPNYKGNDIKCTSTSRDKITTL.
QWY15915.1	LDYGQCPNNMMGTFSIRTVGTEK.SPHSITLRVYMRIKHVRAWIPRPLRNQPYLFKTNPNYKGNDIKCTSTSRDKITTL.
QWY15835.1	LDYGQCPNNMMGTFSIRTVGTEK.SPHSITLRVYMRIKHVRAWIPRPLRNQPYLFKTNPNYKGNDIKCTSTSRDKITTL.
QWY15832.1	LDYGQCPNNMMGTFSIRTVGTEK.SPHSITLRVYMRIKHVRAWIPRPLRNQPYLFKTNPNYKGNDIKCTSTSRDKITTL.
QWY15830.1	LDYGQCPNNMMGTFSIRTVGTEK.SPHSITLRVYMRIKHVRAWIPRPLRNQPYLFKTNPNYKGNDIKCTSTSRDKITTL.
QWY15829.1	LDYGQCPNNMMGTFSIRTVGTEK.SPHSITLRVYMRIKHVRAWIPRPLRNQPYLFKTNPNYKGNDIKCTSTSRDKITTL.
QWY15826.1	LDYGQCPNNMMGTFSIRTVGTEK.SPHSITLRVYMRIKHVRAWIPRPLRNQPYLFKTNPNYKGNDIKCTSTSRDKITTL.
QWY15802.1	LDYGQCPNNMMGTFSIRTVGTEK.SPHSITLRVYMRIKHVRAWIPRPLRNQPYLFKTNPNYKGNDIKCTSTSRDKITTL.
QWY15799.1	LDYGQCPNNMMGTFSIRTVGTEK.SPHSITLRVYMRIKHVRAWIPRPLRNQPYLFKTNPNYKGNDIKCTSTSRDKITTL.
QWY15788.1	LDYGQCPNNMMGTFSIRTVGTEK.SPHSITLRVYMRIKHVRAWIPRPLRNQPYLFKTNPNYKGNDIKCTSTSRDKITTL.
QWY15783.1	LDYGQCPNNMMGTFSIRTVGTEK.SPHSITLRVYMRIKHVRAWIPRPLRNQPYLFKTNPNYKGNDIKCTSTSRDKITTL.
QWY15768.1	LDYGQCPNNMMGTFSIRTVGTEK.SPHSITLRVYMRIKHVRAWIPRPLRNQPYLFKTNPNYKGNDIKCTSTSRDKITTL.
QWY15766.1	LDYGQCPNNMMGTFSIRTVGTEK.SPHSITLRVYMRIKHVRAWIPRPLRNQPYLFKTNPNYKGNDIKCTSTSRDKITTL.
QWY15764.1	LDYGQCPNNMMGTFSIRTVGTEK.SPHSITLRVYMRIKHVRAWIPRPLRNQPYLFKTNPNYKGNDIKCTSTSRDKITTL.
QWY15763.1	LDYGQCPNNMMGTFSIRTVGTEK.SPHSITLRVYMRIKHVRAWIPRPLRNQPYLFKTNPNYKGNDIKCTSTSRDKITTL.
QWY15760.1	LDYGQCPNNMMGTFSIRTVGTEK.SPHSITLRVYMRIKHVRAWIPRPLRNQPYLFKTNPNYKGNDIKCTSTSRDKITTL.
QWF08768.1	LDYGQCPNNMMGTFSIRTVGTEK.SPHSITLRVYMRIKHVRAWIPRPLRNQPYLFKTNPNYKGNDIKCTSTSRDKITTL.
QWF08766.1	LDYGQCPNNMMGTFSIRTVGTEK.SPHSITLRVYMRIKHVRAWIPRPLRNQPYLFKTNPNYKGNDIKCTSTSRDKITTL.
QWY15790.1	LDYGQCPNNMMGTFSIRTVGTEK.SPHSITLRVYMRIKHVRAWIPRPLRNQPYLFKTNPNYKGNDIKCTSTSRDKITTL.
QWF08769.1	LDYGQCPNNMMGTFSIRTVGTEK.SPHSITLRVYMRIKHVRAWIPRPLRNQPYLFKTNPNYKGNDIKCTSTSRDKITTL.
QWY15930.1	LDYGQCPNNMMGTFSIRTVGTEK.SPHSITLRVYMRIKHVRAWIPRPLRNQPYLFKTNPNYKGNDIKCTSTSRDKITTL.
QWY15833.1	LDYGQCPNNMMGTFSIRTVGTEK.SPHSITLRVYMRIKHVRAWIPRPLRNQPYLFKTNPNYKGNDIKCTSTSRDKITTL.
QWY15827.1	LDYGQCPNNMMGTFSIRTVGTEK.SPHSITLRVYMRIKHVRAWIPRPLRNQPYLFKTNPNYKGNDIKCTSTSRDKITTL.
QWY15838.1	LDYGQCPNNMMGTFSIRTVGTEK.SPHSITLRVYMRIKHVRAWIPRPLRNQPYLFKTNPNYKGNDIKCTSTSRDKITTL.
BBU37460.1	LDYGQCPNNMMGTFSIRTVGTEK.SPHSITLRVYMRIKHVRAWIPRPLRNQPYLFKTNPNYKGNDIKCTSTSRDKITTL.
QWF08920.1	LDYGQCPNNMMGTFSIRTVGTEK.SPHSITLRVYMRIKHVRAWIPRPLRNQPYLFKTNPNYKGNDIKCTSTSRDKITTL.
QWY15769.1	LDYGQCPNNMMGTFSIRTVGTEK.SPHSITLRVYMRIKHVRAWIPRPLRNQPYLFKTNPNYKGNDIKCTSTSRDKITTL.
QWY15755.1	LDYGQCPNNMMGTFSIRTVGTEK.SPHSITLRVYMRIKHVRAWIPRPLRNQPYLFKTNPNYKGNDIKCTSTSRDKITTL.
QWY15702.1	LDYGQCPNNMMGTFSIRTVGTEK.SPHSITLRMYMRIKHVRAWIPRPLRNQPYLFKTNPNYKGNDIKCTSTSRDKITTL.
QWY15739.1	LDYGQCPNNMMGTFSIRTVGTEK.SPHSIALRVYMRIKHVRAWIPRPLRNQPYLFKTNPNYKGNDIKCTSTSRDKITTL.
QWY15737.1	LDYGQCPNNMMGTFSIRTVGTEK.SPHSIALRVYMRIKHVRAWIPRPLRNQPYLFKTNPNYKGNDIKCTSASRDKITTL.
QWF08788.1	LDYGQCPNNMMGTFSIRTVGTEK.SPHSIALRVYMRIKHVRAWIPRPLRNQPYLFKTNPNYKGNDIKCTSTSRDKITTL.
QYC65122.1	LDYGQCPNNMMGTFSIRTVGTEK.SPHSITLRVYMRIKHVRAWIPRPLRNQPYLFKTNPNYKGNDVKCTSTSRDKITTL.
QWF08891.1	LDYGQCPNNMMGTFSIRTVGTEK.SPHSITLRVYMRIKHVRAWIPRPLRNQPYLFKTNPNYKGNDMKCTSTSRDKITTL.
QWY15903.1	LDYGQCPNNMMGTFSIRTVGTEK.SPHSVTLRVYMRIKHVRAWIPRPLRNQPYLFKTNPNYKGNDIKCTSTSRDKITTL.
BCI65366.1	LDYGQCPNNMMGTFSIRTVGTEK.SPHSVTLRVYMRIKHVRAWIPRPLRNQPYLFKTNPNYKGNDIKCTSTSRDKITTL.
BCG67045.1	LDYGQCPNNMMGTFSIRTVGTEK.SPHSVTLRVYMRIKHVRAWIPRPLRNQPYLFKTNPNYKGNDIKCTSTSRDKITTL.
BCG67044.1	LDYGQCPNNMMGTFSIRTVGTEK.SPHSVTLRVYMRIKHVRAWIPRPLRNQPYLFKTNPNYKGNDIKCTSTSRDKITTL.
QWY15819.1	LDYGQCPNNMMGTFSIRTVGTEK.SPHSVTLRVYMRIKHVRAWIPRPLRNQPYLFKTNPNYKGNDIKCTSTSRDKITTL.
QWY15818.1	LDYGQCPNNMMGTFSIRTVGTEK.SPHSVTLRVYMRIKHVRAWIPRPLRNQPYLFKTNPNYKGNDIKCTSTSRDKITTL.
QTA73912.1	LDYGQCPNNMMGTFSIRTVGTEK.SPHSITLRVYMRIKHVRAWIPRPLRNQPYLFKTNPNYKGNDIKCTSTSRDKITTL.
QWY15914.1	LDYGQCPNNMMGTFSIRTVGTEK.SPHSITLRVYMRIKHVRAWIPRPLRNQPYLFKTNPNYKGNDIKCTSTSRDKITTL.
QWY15909.1	LDYGQCPNNMMGTFSIRTVGTEK.SPHSITLRVYMRIKHVRAWIPRPLRNQPYLFKTNPNYKGNDIKCTSTSRDKITTL.
QWY15905.1	LDYGQCPNNMMGTFSIRTVGTEK.SPHSITLRVYMRIKHVRAWIPRPLRNQPYLFKTNPNYKGNDIKCTSTSRDKITTL.
QWY15856.1	LDYGQCPNNMMGTFSIRTVGTEK.SPHSITLRVYMRIKHVRAWIPRPLRNQPYLFKTNPNYKGNDIKCTSTSRDKITTL.
QWY15850.1	LDYGQCPNNMMGTFSIRTVGTEK.SPHSITLRVYMRIKHVRAWIPRPLRNQPYLFKTNPNYKGNDIKCTSTSRDKITTL.
QWY15849.1	LDYGQCPNNMMGTFSIRTVGTEK.SPHSITLRVYMRIKHVRAWIPRPLRNQPYLFKTNPNYKGNDIKCTSTSRDKITTL.
QWY15847.1	LDYGQCPNNMMGTFSIRTVGTEK.SPHSITLRVYMRIKHVRAWIPRPLRNQPYLFKTNPNYKGNDIKCTSTSRDKITTL.
QWY15845.1	LDYGQCPNNMMGTFSIRTVGTEK.SPHSITLRVYMRIKHVRAWIPRPLRNQPYLFKTNPNYKGNDIKCTSTSRDKITTL.
QWY15836.1	LDYGQCPNNMMGTFSIRTVGTEK.SPHSITLRVYMRIKHVRAWIPRPLRNQPYLFKTNPNYKGNDIKCTSTSRDKITTL.
QWY15765.1	LDYGQCPNNMMGTFSIRTVGTEK.SPHSITLRVYMRIKHVRAWIPRPLRNQPYLFKTNPNYKGNDIKCTSTSRDKITTL.
QWY15734.1	LDYGQCPNNMMGTFSIRTVGTEK.SPHSITLRVYMRIKHVRAWIPRPLRNQPYLFKTNPNYKGNDIKCTSTSRDKITTL.
QWY15855.1	LDYGQCPNNMMGTFSIRTVGTEK.SPHSITLRVYMRIKHVRAWIPRPLRNQPYLFKTNPNYKGNDIKCTSTSRDKITTL.
QWY15831.1	LDYGQCPNNMMGTFSIRTVGTEK.SPHSITLRVYMRIKHVRAWIPRPLKNQPYLFKTNPNYKGNDIKCTSTSRDKITTL.
QWY15798.1	LDYGQCPNNMMGTFSIRTVGTEK.SPHSITLRVYMRIKHVRAWIPRPLRNQPYLFKTNPNYKGNDIKCTSASRDKITTL.
QWY15797.1	LDYGQCPNNMMGTFSIRTVGTEK.SPHSITLRVYMRIKHVRAWIPRPLRNQPYLFKTNPNYKGNDIKCTSASRDKITTL.
QWY15796.1	LDYGQCPNNMMGTFSIRTVGTEK.SPHSITLRVYMRIKHVRAWIPRPLRNQPYLFKTNPNYKGNDIKCTSASRDKITTL.
QWY15795.1	LDYGQCPNNMMGTFSIRTVGTEK.SPHSITLRVYMRIKHVRAWIPRPLRNQPYLFKTNPNYKGNDIKCTSASRDKITTL.
QWY15794.1	LDYGQCPNNMMGTFSIRTVGTEK.SPHSITLRVYMRIKHVRAWIPRPLRNQPYLFKTNPNYKGNDIKCTSASRDKITTL.
QWY15971.1	LDYGQCPNNMMGTFSIRTVGTEK.SPHSITLRVYMRIKHVRAWIPRPLRNQPYLFKTNPNYKGNDIKCTSTSRDKITTL.
QWY15897.1	LDYGQCPNNMMGTFSIRTVGTEK.SPHSITLRVYMRIKHVRAWIPRPLRNQPYLFKTNPNYKGNDIKCTSTSRDKITTL.
QWY15742.1	LDYGQCPNNMMGTFSIRTVGTEK.SPHSITLRVYMRIKHVRAWIPRPLRNQPYLFKTNPNYKGNDIKCTSTSRDKITTL.
QWY15741.1	LDYGQCPNNMMGTFSIRTVGTEK.SPHSITLRVYMRIKHVRAWIPRPLRNQPYLFKTNPNYKGNDIKCTSTSRDKITTL.
QWY15923.1	LDYGQCPNNMMGTFSIRTVGTEK.SPHSITLRVYMRIKHVRAWIPRPLRNQPYLFKTNPNYKGNDIKCTSTSRDKITTL.
QWF08957.1	LDYGQCPNNMMGTFSIRTVGTEK.SPHSITLRVYMRIKHVRAWIPRPLRNQPYLFKTNPNYKGNDIKCTSTSRDKITTL.
UEP53407.1	LDYGQCPNNMMGTFSIRTVGTEK.SPHSITLRVYMRIKHVRAWIPRPLRNQPYLFKTNPNYKGNDIKCTSPSRDKITTL.
QWY15922.1	LDYGQCPNNMMGTFSIRTVGTEK.SPHSITLRVYMRIKHVRAWIPRPLRNQPYLFKTNPNYKGNDIKCTSTSRDKITTL.
QWY15848.1	LDYGQCPNNMMGTFSIRTVGTEK.SPHSITLRVYMRIKHVRAWIPRPLRNQPYLFKTNPNYKGNDIKCTSTSRDKITTL.
UEP53405.1	LDYGQCPNNMMGTFSIRTVGTEK.SPHSITLRVYMRIKHVRAWIPRPLRNQPYLFKTNPNYKGNDIKCTSTSRDKITTL.
UEC49855.1	LDYGQCPNNMMGTFSIRTVGTEK.SPHSITLRVYMRIKHVRAWIPRPLRNQPYLFKTNPNYKGNDIKCTSTSRDKITTL.
QWY15851.1	LDYGQCPNNMMGTFSIRTVGTEK.SPHSITLRVYMRIKHVRAWIPRPLRNQPYLFKTNPNYKGNDIKCTSTSRDKITTL.
QYC65112.1	LDYGQCPNNMMGTFSIRTVGTEK.SPHSITLRVYMRIKHVRAWIPRPLRNQPYLFKTNPNYKGNDIKCTSTSRDKITTL.
QOP74942.1	LDYGQCPNNMMGTFSIRTVGTEK.SPHSITLRVYMRIKHVRAWIPRPLRNQPYLFKTNPNYKGNDIKCTSTSRDKITT..
QLH02013.1	LDYGQCPNNMMGTFSIRTVGTEK.SPHSITLRVYMRIKHVRAWIPRPLRNQPYLFKTNPNYKGNDIKCTSTSRDKITTL.
QIH53923.1	LDYGQCPNNMMGTFSIRTVGTEK.SPHSITLRVYMRIKHVRAWIPRPLRNQPYLFKTNPNYKGNDIKCTSTSRDKITTL.
QIH53918.1	LDYGQCPNNMMGTFSIRTVGTEK.SPHSITLRVYMRIKHVRAWIPRPLRNQPYLFKTNPNYKGNDIKCTSTSRDKITTL.
QIH53915.1	LDYGQCPNNMMGTFSIRTVGTEK.SPHSITLRVYMRIKHVRAWIPRPLRNQPYLFKTNPNYKGNDIKCTSTSRDKITTL.
QIH53914.1	LDYGQCPNNMMGTFSIRTVGTEK.SPHSITLRVYMRIKHVRAWIPRPLRNQPYLFKTNPNYKGNDIKCTSTSRDKITTL.
QYC65127.1	LDYGQCPNNMMGTFSIRTVGTEK.SPHSITLRVYMRIKHVRAWIPRPLRNQPYLFKTNPNYKGNDIKCTSTSRDKITTL.
QIL87534.1	LDYGQCPNNMMGTFSIRTVGTEK.SPHSITLRVYMRIKHVRAWIPRPLRNQPYLFKTNPNYKGNDIKCTSTSRDKITTL.
QWF08936.1	LDYGQCPNNMMGTFSIRTVGTEK.SPHSITLRVYMRIKHVRAWIPRPLRNQPYLFKTNPNYKGNDIKCTSTSRDKITTL.
QIL87532.1	LDYGQCPNNMMGTFSIRTVGTEK.SPHSITLRVYMRIKHVRAWIPRPLRNQPYLFKTNPNYKGNDIKCTSTSRDKITTL.
QYC65089.1	LDYGQCPNNMMGTFSIRTVGTEK.SPHSITLRVYMRIKHVRAWIPRPLRNQPYLFKTNPNYKGNDIKCTSTSRDKITTL.
QWF08950.1	LDYGQCPNNMMGTFSIRTVGTEK.SPHSITLRVYMRIKHVRAWIPRPLRNQPYLFKTNPNYKGNDIKCTSTSRDKITTL.
QIL87530.1	LDYGQCPNNMMGTFSIRTVGTEK.SPHSITLRVYMRIKHVRAWIPRPLRNQPYLFKTNPNYKGNDIKCTSTSRDKITTL.
QWF08820.1	LDYGQCPNNMMGTFSIRTVGTEK.SPHSITLRVYMRIKHVRAWIPRPLRNQPYLFKTNPNYKGNDIKCTSTSRDKITTL.
QIL87529.1	LDYGQCPNNMMGTFSIRTVGTEK.SPHSITLRVYMRIKHVRAWIPRPLRNQPYLFKTNPNYKGNDIKCTSTSRDKITTL.
QIL87528.1	LDYGQCPNNMMGTFSIRTVGTEK.SPHSITLRVYMRIKHVRAWIPRPLRNQPYLFKTNPNYKGNDIKCTSTSRDKITTL.
QYC65143.1	LDYGQCPNNMMGTFSIRTVGTEK.SPHSITLRVYMRIKHVRAWIPRPLRNQPYLFKTNPNYKGNDIKCTSTSRDKITTL.
QIL87525.1	LDYGQCPNNMMGTFSIRTVGTEK.SPHSITLRVYMRIKHVRAWIPRPLRNQPYLFKTNPNYKGNDIKCTSTSRDKITTL.
QYC65141.1	LDYGQCPNNMMGTFSIRTVGTEK.SPHSITLRVYMRIKHVRAWIPRPLRNQPYLFKTNPNYKGNDIKCTSTSRDKITTL.
QYC65084.1	LDYGQCPNNMMGTFSIRTVGTEK.SPHSITLRVYMRIKHVRAWIPRPLRNQPYLFKTNPNYKGNDIKCTSTSRDKITTL.
QWF08947.1	LDYGQCPNNMMGTFSIRTVGTEK.SPHSITLRVYMRIKHVRAWIPRPLRNQPYLFKTNPNYKGNDIKCTSTSRDKITTL.
QWF08922.1	LDYGQCPNNMMGTFSIRTVGTEK.SPHSITLRVYMRIKHVRAWIPRPLRNQPYLFKTNPNYKGNDIKCTSTSRDKITTL.
QIL87524.1	LDYGQCPNNMMGTFSIRTVGTEK.SPHSITLRVYMRIKHVRAWIPRPLRNQPYLFKTNPNYKGNDIKCTSTSRDKITTL.
QIL87520.1	LDYGQCPNNMMGTFSIRTVGTEK.SPHSITLRVYMRIKHVRAWIPRPLRNQPYLFKTNPNYKGNDIKCTSTSRDKITTL.
QIL87518.1	LDYGQCPNNMMGTFSIRTVGTEK.SPHSITLRVYMRIKHVRAWIPRPLRNQPYLFKTNPNYKGNDIKCTSTSRDKITTL.
QYC65103.1	LDYGQCPNNMMGTFSIRTVGTEK.SPHSITLRVYMRIKHVRAWIPRPLRNQPYLFKTNPNYKGNDIKCTSTSRDKITTL.
QWF08944.1	LDYGQCPNNMMGTFSIRTVGTEK.SPHSITLRVYMRIKHVRAWIPRPLRNQPYLFKTNPNYKGNDIKCTSTSRDKITTL.
QWF08943.1	LDYGQCPNNMMGTFSIRTVGTEK.SPHSITLRVYMRIKHVRAWIPRPLRNQPYLFKTNPNYKGNDIKCTSTSRDKITTL.
QWF08927.1	LDYGQCPNNMMGTFSIRTVGTEK.SPHSITLRVYMRIKHVRAWIPRPLRNQPYLFKTNPNYKGNDIKCTSTSRDKITTL.
QWF08942.1	LDYGQCPNNMMGTFSIRTVGTEK.SPHSITLRVYMRIKHVRAWIPRPLRNQPYLFKTNPNYKGNDIKCTSTSRDKITTL.
QWF08940.1	LDYGQCPNNMMGTFSIRTVGTEK.SPHSITLRVYMRIKHVRAWIPRPLRNQPYLFKTNPNYKGNDIKCTSTSRDKITTL.
QWF08912.1	LDYGQCPNNMMGTFSIRTVGTEK.SPHSITLRVYMRIKHVRAWIPRPLRNQPYLFKTNPNYKGNDIKCTSTSRDKITTL.
QWF08907.1	LDYGQCPNNMMGTFSIRTVGTEK.SPHSITLRVYMRIKHVRAWIPRPLRNQPYLFKTNPNYKGNDIKCTSTSRDKITTL.
QWF08904.1	LDYGQCPNNMMGTFSIRTVGTEK.SPHSITLRVYMRIKHVRAWIPRPLRNQPYLFKTNPNYKGNDIKCTSTSRDKITTL.
QWF08886.1	LDYGQCPNNMMGTFSIRTVGTEK.SPHSITLRVYMRIKHVRAWIPRPLRNQPYLFKTNPNYKGNDIKCTSTSRDKITTL.
QWY15877.1	LDYGQCPNNMMGTFSIRTVGTEK.SPHSITLRVYMRIKHVRAWIPRPLRNQPYLFKTNPNYKGNDIKCTSTSRDKITTL.
QWY15965.1	LDYGQCPNNMMGTFSIRTVGTEK.SPHSITLRVYMRIKHVRAWIPRPLRNQPYLFKTNPNYKGNDIKCTSTSRDKITTL.
UEC49851.1	LDYGQCPNNMMGTFSIRTVGTEK.SPHSITLRVYMRIKHVRAWIPRPLRNQPYLFKTNPNYKGNDIKCTSTSRDKITTL.
UEC49850.1	LDYGQCPNNMMGTFSIRTVGTEK.SPHSITLRVYMRIKHVRAWIPRPLRNQPYLFKTNPNYKGNDIKCTSTSRDKITTL.
UEC49849.1	LDYGQCPNNMMGTFSIRTVGTEK.SPHSITLRVYMRIKHVRAWIPRPLRNQPYLFKTNPNYKGNDIKCTSTSRDKITTL.
QWY15963.1	LDYGQCPNNMMGTFSIRTVGTEK.SPHSITLRVYMRIKHVRAWIPRPLRNQPYLFKTNPNYKGNDIKCTSTSRDKITTL.
QWY15972.1	LDYGQCPNNMMGTFSIRTVGTEK.SPHSITLRVYMRIKHVRAWIPRPLRNQPYLFKTNPNYKGNDIKCTSTSRDKITTL.
QWY15958.1	LDYGQCPNNMMGTFSIRTVGTEK.SPHSITLRVYMRIKHVRAWIPRPLRNQPYLFKTNPNYKGNDIKCTSTSRDKITTL.
QWY15899.1	LDYGQCPNNMMGTFSIRTVGTEK.SPHSITLRVYMRIKHVRAWIPRPLRNQPYLFKTNPNYKGNDIKCTSTSRDKITTL.
QWY15898.1	LDYGQCPNNMMGTFSIRTVGTEK.SPHSITLRVYMRIKHVRAWIPRPLRNQPYLFKTNPNYKGNDIKCTSTSRDKITTL.
QWY15895.1	LDYGQCPNNMMGTFSIRTVGTEK.SPHSITLRVYMRIKHVRAWIPRPLRNQPYLFKTNPNYKGNDIKCTSTSRDKITTL.
QWY15893.1	LDYGQCPNNMMGTFSIRTVGTEK.SPHSITLRVYMRIKHVRAWIPRPLRNQPYLFKTNPNYKGNDIKCTSTSRDKITTL.
QWY15892.1	LDYGQCPNNMMGTFSIRTVGTEK.SPHSITLRVYMRIKHVRAWIPRPLRNQPYLFKTNPNYKGNDIKCTSTSRDKITTL.
QWY15891.1	LDYGQCPNNMMGTFSIRTVGTEK.SPHSITLRVYMRIKHVRAWIPRPLRNQPYLFKTNPNYKGNDIKCTSTSRDKITTL.
QWY15970.1	LDYGQCPNNMMGTFSIRTVGTEK.SPHSITLRVYMRIKHVRAWIPRPLRNQPYLFKTNPNYKGNDIKCTSTSRDKITTL.
QWY15966.1	LDYGQCPNNMMGTFSIRTVGTEK.SPHSITLRVYMRIKHVRAWIPRPLRNQPYLFKTNPNYKGNDIKCTSTSRDKITTL.
QWY15962.1	LDYGQCPNNMMGTFSIRTVGTEK.SPHSITLRVYMRIKHVRAWIPRPLRNQPYLFKTNPNYKGNDIKCTSTSRDKITTL.
QWY15961.1	LDYGQCPNNMMGTFSIRTVGTEK.SPHSITLRVYMRIKHVRAWIPRPLRNQPYLFKTNPNYKGNDIKCTSTSRDKITTL.
QWY15925.1	LDYGQCPNNMMGTFSIRTVGTEK.SPHSITLRVYMRIKHVRAWIPRPLRNQPYLFKTNPNYKGNDIKCTSTSRDKITTL.
QWY15773.1	LDYGQCPNNMMGTFSIRTVGTEK.SPHSITLRVYMRIKHVRAWIPRPLRNQPYLFKTNPNYKGNDIKCTSTSRDKITTL.
QWY15924.1	LDYGQCPNNMMGTFSIRTVGTEK.SPHSITLRVYMRIKHVRAWIPRPLRNQPYLFKTNPNYKGNDIKCTSTSRDKITTL.
QWY15910.1	LDYGQCPNNMMGTFSIRTVGTEK.SPHSITLRVYMRIKHVRAWIPRPLRNQPYLFKTNPNYKGNDIKCTSTSRDKITTL.

```
QWY15824.1   LDYGQCPNNMMGTFSIRTVGTEK.SPHSITLRVYMRIKHVRAWIPRPLRNQPYLFKTNPNYKGNDIKCTSTSRDKITTL.
QWY15823.1   LDYGQCPNNMMGTFSIRTVGTEK.SPHSITLRVYMRIKHVRAWIPRPLRNQPYLFKTNPNYKGNDIKCTSTSRDKITTL.
QWY15822.1   LDYGQCPNNMMGTFSIRTVGTEK.SPHSITLRVYMRIKHVRAWIPRPLRNQPYLFKTNPNYKGNDIKCTSTSRDKITTL.
QWY15821.1   LDYGQCPNNMMGTFSIRTVGTEK.SPHSITLRVYMRIKHVRAWIPRPLRNQPYLFKTNPNYKGNDIKCTSTSRDKITTL.
QWY15816.1   LDYGQCPNNMMGTFSIRTVGTEK.SPHSITLRVYMRIKHVRAWIPRPLRNQPYLFKTNPNYKGNDIKCTSTSRDKITTL.
QWY15778.1   LDYGQCPNNMMGTFSIRTVGTEK.SPHSITLRVYMRIKHVRAWIPRPLRNQPYLFKTNPNYKGNDIKCTSTSRDKITTL.
QWY15801.1   LDYGQCPNNMMGTFSIRTVGTEK.SPHSITLRVYMRIKHVRAWIPRPLRNQPYLFKTNPNYKGNDIKCTSTSRDKITTL.
QWY15791.1   LDYGQCPNNMMGTFSIRTVGTEK.SPHSITLRVYMRIKHVRAWIPRPLRNQPYLFKTNPNYKGNDIKCTSTSRDKITTL.
QWY15775.1   LDYGQCPNNMMGTFSIRTVGTEK.SPHSITLRVYMRIKHVRAWIPRPLRNQPYLFKTNPNYKGNDIKCTSTSRDKITTL.
QWY15774.1   LDYGQCPNNMMGTFSIRTVGTEK.SPHSITLRVYMRIKHVRAWIPRPLRNQPYLFKTNPNYKGNDIKCTSTSRDKITTL.
BCR43107.1   LDYGQCPNNMMGTFSIRTVGTEK.SPHSITLRVYMRIKHVRAWIPRPLRNQPYLFKTNPNYKGNDIKCTSTSRDKITTL.
QYC65183.1   LDYGQCPNNMMGTFSIRTVGTEK.SPHSITLRVYMRIKHVRAWIPRPLRNQPYLFKTNPNYKGNDIKCTSTSRDKITTL.
BCR43094.1   LDYGQCPNNMMGTFSIRTVGTEK.SPHSITLRVYMRIKHVRAWIPRPLRNQPYLFKTNPNYKGNDIKCTSTSRDKITTL.
BCR43081.1   LDYGQCPNNMMGTFSIRTVGTEK.SPHSITLRVYMRIKHVRAWIPRPLRNQPYLFKTNPNYKGNDIKCTSTSRDKITTL.
BCG67017.1   LDYGQCPNNMMGTFSIRTVGTEK.SPHSITLRVYMRIKHVRAWIPRPLRNQPYLFKTNPNYKGNDIKCTSTSRDKITTL.
BCG67015.1   LDYGQCPNNMMGTFSIRTVGTEK.SPHSITLRVYMRIKHVRAWIPRPLRNQPYLFKTNPNYKGNDIKCTSTSRDKITTL.
BCG67007.1   LDYGQCPNNMMGTFSIRTVGTEK.SPHSITLRVYMRIKHVRAWIPRPLRNQPYLFKTNPNYKGNDIKCTSTSRDKITTL.
BCG67005.1   LDYGQCPNNMMGTFSIRTVGTEK.SPHSITLRVYMRIKHVRAWIPRPLRNQPYLFKTNPNYKGNDIKCTSTSRDKITTL.
QYC65184.1   LDYGQCPNNMMGTFSIRTVGTEK.SPHSITLRVYMRIKHVRAWIPRPLRNQPYLFKTNPNYKGNDIKCTSTSRDKITTL.
QWF08853.1   LDYGQCPNNMMGTFSIRTVGTEK.SPHSITLRVYMRIKHVRAWIPRPLRNQPYLFKTNPNYKGNDIKCTSTSRDKITTL.
QYC65140.1   LDYGQCPNNMMGTFSIRTVGTEK.SPHSITLRVYMRIKHVRAWIPRPLRNQPYLFKTNPNYKGNDIKCTSTSRDKITTL.
QYC65133.1   LDYGQCPNNMMGTFSIRTVGTEK.SPHSITLRVYMRIKHVRAWIPRPLRNQPYLFKTNPNYKGNDIKCTSTSRDKITTL.
QYC65110.1   LDYGQCPNNMMGTFSIRTVGTEK.SPHSITLRVYMRIKHVRAWIPRPLRNQPYLFKTNPNYKGNDIKCTSTSRDKITTL.
QYC65108.1   LDYGQCPNNMMGTFSIRTVGTEK.SPHSITLRVYMRIKHVRAWIPRPLRNQPYLFKTNPNYKGNDIKCTSTSRDKITTL.
QYC65098.1   LDYGQCPNNMMGTFSIRTVGTEK.SPHSITLRVYMRIKHVRAWIPRPLRNQPYLFKTNPNYKGNDIKCTSTSRDKITTL.
QYC65097.1   LDYGQCPNNMMGTFSIRTVGTEK.SPHSITLRVYMRIKHVRAWIPRPLRNQPYLFKTNPNYKGNDIKCTSTSRDKITTL.
QYC65094.1   LDYGQCPNNMMGTFSIRTVGTEK.SPHSITLRVYMRIKHVRAWIPRPLRNQPYLFKTNPNYKGNDIKCTSTSRDKITTL.
QYC65091.1   LDYGQCPNNMMGTFSIRTVGTEK.SPHSITLRVYMRIKHVRAWIPRPLRNQPYLFKTNPNYKGNDIKCTSTSRDKITTL.
QYC65057.1   LDYGQCPNNMMGTFSIRTVGTEK.SPHSITLRVYMRIKHVRAWIPRPLRNQPYLFKTNPNYKGNDIKCTSTSRDKITTL.
QWF08850.1   LDYGQCPNNMMGTFSIRTVGTEK.SPHSITLRVYMRIKHVRAWIPRPLRNQPYLFKTNPNYKGNDIKCTSTSRDKITTL.
QYC65056.1   LDYGQCPNNMMGTFSIRTVGTEK.SPHSITLRVYMRIKHVRAWIPRPLRNQPYLFKTNPNYKGNDIKCTSTSRDKITTL.
QWF08849.1   LDYGQCPNNMMGTFSIRTVGTEK.SPHSITLRVYMRIKHVRAWIPRPLRNQPYLFKTNPNYKGNDIKCTSTSRDKITTL.
QWF08969.1   LDYGQCPNNMMGTFSIRTVGTEK.SPHSITLRVYMRIKHVRAWIPRPLRNQPYLFKTNPNYKGNDIKCTSTSRDKITTL.
QWF08848.1   LDYGQCPNNMMGTFSIRTVGTEK.SPHSITLRVYMRIKHVRAWIPRPLRNQPYLFKTNPNYKGNDIKCTSTSRDKITTL.
QWF08968.1   LDYGQCPNNMMGTFSIRTVGTEK.SPHSITLRVYMRIKHVRAWIPRPLRNQPYLFKTNPNYKGNDIKCTSTSRDKITTL.
QWF08965.1   LDYGQCPNNMMGTFSIRTVGTEK.SPHSITLRVYMRIKHVRAWIPRPLRNQPYLFKTNPNYKGNDIKCTSTSRDKITTL.
QWF08956.1   LDYGQCPNNMMGTFSIRTVGTEK.SPHSITLRVYMRIKHVRAWIPRPLRNQPYLFKTNPNYKGNDIKCTSTSRDKITTL.
QWF08952.1   LDYGQCPNNMMGTFSIRTVGTEK.SPHSITLRVYMRIKHVRAWIPRPLRNQPYLFKTNPNYKGNDIKCTSTSRDKITTL.
QWF08903.1   LDYGQCPNNMMGTFSIRTVGTEK.SPHSITLRVYMRIKHVRAWIPRPLRNQPYLFKTNPNYKGNDIKCTSTSRDKITTL.
QWF08902.1   LDYGQCPNNMMGTFSIRTVGTEK.SPHSITLRVYMRIKHVRAWIPRPLRNQPYLFKTNPNYKGNDIKCTSTSRDKITTL.
QWF08895.1   LDYGQCPNNMMGTFSIRTVGTEK.SPHSITLRVYMRIKHVRAWIPRPLRNQPYLFKTNPNYKGNDIKCTSTSRDKITTL.
QWF08888.1   LDYGQCPNNMMGTFSIRTVGTEK.SPHSITLRVYMRIKHVRAWIPRPLRNQPYLFKTNPNYKGNDIKCTSTSRDKITTL.
QWF08893.1   LDYGQCPNNMMGTFSIRTVGTEK.SPHSITLRVYMRIKHVRAWIPRPLRNQPYLFKTNPNYKGNDIKCTSTSRDKITTL.
QWF08892.1   LDYGQCPNNMMGTFSIRTVGTEK.SPHSITLRVYMRIKHVRAWIPRPLRNQPYLFKTNPNYKGNDIKCTSTSRDKITTL.
QWF08876.1   LDYGQCPNNMMGTFSIRTVGTEK.SPHSITLRVYMRIKHVRAWIPRPLRNQPYLFKTNPNYKGNDIKCTSTSRDKITTL.
QWF08868.1   LDYGQCPNNMMGTFSIRTVGTEK.SPHSITLRVYMRIKHVRAWIPRPLRNQPYLFKTNPNYKGNDIKCTSTSRDKITTL.
QWF08862.1   LDYGQCPNNMMGTFSIRTVGTEK.SPHSITLRVYMRIKHVRAWIPRPLRNQPYLFKTNPNYKGNDIKCTSTSRDKITTL.
QWF08861.1   LDYGQCPNNMMGTFSIRTVGTEK.SPHSITLRVYMRIKHVRAWIPRPLRNQPYLFKTNPNYKGNDIKCTSTSRDKITTL.
QWF08860.1   LDYGQCPNNMMGTFSIRTVGTEK.SPHSITLRVYMRIKHVRAWIPRPLRNQPYLFKTNPNYKGNDIKCTSTSRDKITTL.
QWF08859.1   LDYGQCPNNMMGTFSIRTVGTEK.SPHSITLRVYMRIKHVRAWIPRPLRNQPYLFKTNPNYKGNDIKCTSTSRDKITTL.
QWF08858.1   LDYGQCPNNMMGTFSIRTVGTEK.SPHSITLRVYMRIKHVRAWIPRPLRNQPYLFKTNPNYKGNDIKCTSTSRDKITTL.
QWF08854.1   LDYGQCPNNMMGTFSIRTVGTEK.SPHSITLRVYMRIKHVRAWIPRPLRNQPYLFKTNPNYKGNDIKCTSTSRDKITTL.
QWF08929.1   LDYGQCPNNMMGTFSIRTVGTEK.SPHSITLRVYMRIKHVRAWIPRPLRNQPYLFKTNPNYKGNDIKCTSTSRDKITTL.
QYC65137.1   LDYGQCPNNMMGTFSIRTVGTEK.SPHSITLRVYMRIKHVRAWIPRPLRNQPYLFKTNPNYKGNDIKCTSTSRDKITTL.
QYC65100.1   LDYGQCPNNMMGTFSIRTVGTEK.SPHSITLRVYMRIKHVRAWIPRPLRNQPYLFKTNPNYKGNDIKCTSTSRDRITTL.
QWY15926.1   LDYGQCPNNMMGTFSIRTVGTEK.SPHSITLRVYMRIKHVRAWIPRPLRNQPYLFKTNPNYKGNDIKCTSTSRDKITTL.
QWF08962.1   LDYGQCPNNMMGTFSIRTVGTEK.SPHSITLRVYMRIKHVRAWIPRPLRNQPYLFKTNPNYKGNDIKCTSTSRDKITTL.
QWF08909.1   LDYGQCPNNMMGTFSIRTVGTEK.SPHSITLRVYMRIKHVRAWIPRPLRNQPYLFKTNPNYKGNDIKCTSTSRDKITTL.
QWY15854.1   LDYGQCPNNMMGTFSIRTVGTKK.SPHSITLRVYMRIKHVRAWIPRPLRNQPYLFKTNPNYKGNDIKCTSTSRDKITTL.
QWY15808.1   LDYGQCPNNMMGTFSIRTVGTEK.SPHSITLRVYMRIKHVRAWIPRPLRNQPYLFKTNPNYKGNDIKCTSTSRDKITTL.
QWF08770.1   LDYGQCPNNMMGTFSIRTVGTEK.SPHSITLRVYMRIKHVRAWIPRPLRNQPYLFKTNPNYKGNDIKCTSTSRDKITTL.
QWY15728.1   LDYGQCPNNMMGTFSIRTVGTEK.SPHSITLRVYMRIKHVRAWIPRPLRNQPYLFKTNPNYKGNDIKCTSTSRDKITTL.
QYC65126.1   LDYGQCPNNMMGTFSIRTVGTEK.SPHSITLRVYMRIKHVRAWIPRPLRNQPYLFKTNPNYKGNDIKCTSTSRDKITTL.
QWF08827.1   LDYGQCPNNMMGTFSIRTVGTEK.SPHSITLRVYMRIKHVRAWIPRPLRNQPYLFKTNPNYKGNDIKCTSTSRDKITTL.
QYC65072.1   LDYGQCPNNMMGTFSIRTVGTEK.SPHSITLRVYMRIKHVRAWIPRPLRNQPYLFKTNPNYKGNDIKCTSTSRDKITTL.
QYC65067.1   LDYGQCPNNMMGTFSIRTVGTEK.SPHSITLRVYMRIKHVRAWIPRPLRNQPYLFKTNPNYKGNDIKCTSTSRDKITTL.
QWY15767.1   LDYGQCPNNMMGTFSIRTVGTEK.SPHSITLRVYMRIKHVRAWIPRPLRNQPYLFKTNPNYKGNDIKCTSTSRDKITTL.
QWY15735.1   LDYGQCPNNMMGTFSIRTVGTEK.SPHSITLRVYMRIKHVRAWIPRPLRNQPYLFKTNPNYKGNDIKCTSTSRDKITTL.
BCB92153.1   LDYGQCPNNMMGTFSIRTVGTEK.SPHSITLRVYMRIKHVRAWIPRPLRNQPYLFKTNPNYKGNDIKCTSTSRDKITTL.
QWF08966.1   LDYGQCPNNMMGTFSIRTVGTEK.SPHSITLRVYMRIKHVRAWIPRPLRNQPYLFKTNPNYKGNDIKCTSTSRDKITTL.
QIH53917.1   LDYGQCPNNMMGTFSIRTVGTEK.SPHSITLRVYMRIKHVRAWIPRPLRNQPYLFKTNPNYKGNDIKCTSTSRDKITTL.
QYC65092.1   LDYGQCPNNMMGTFSIRTVGTEK.SPHSITLRVYMRIKHVRAWIPRPLRNQPYLFKTNPNYKGNDIKCTSTSRDKITTL.
QWY15879.1   LDYGQCPNNMMGTFSIRTVGTEK.SPHSITLRVYMRIKHVRAWIPRPLRNQPYLFKTNPNYKGNDIKCTSTSRDKITTL.
QWY15777.1   LDYGQCPNNMMGTFSIRTVGTEK.SPHSITLRVYMRIKHVRAWIPRPLRNQPYLFKTNPNYKGNDIKCTSTSRDKITTL.
BCR43105.1   LDYGQCPNNMMGTFSIRTVGTEK.SPHSITLRVYMRIKHVRAWIPRPLRNQPYLFKTNPNYKGNDIKCTSTSRDKITTL.
QWF08826.1   LDYGQCPNNMMGTFSIRTVGTEK.SPHSITLRVYMRIKHVRAWIPRPLRNQPYLFKTNPNYKGNDIKCTSTSRDKITTL.
BCG67024.1   LDYGQCPNNMMGTFSIRTVGTEK.SPHSITLRVYMRIKHVRAWIPRPLRNQPYLFKTNPNYKGNDIKCTSTSRDKITTL.
BCG66999.1   LDYGQCPNNMMGTFSIRTVGTEK.SPHSITLRVYMRIKHVRAWIPRPLRNQPYLFKTNPNYKGNDIKCTSTSRDKITTL.
QWY15752.1   LDYGQCPNNMMGTFSIRTVGTEK.SPHSITLRVYMRIKHVRAWIPRPLRNQPYLFKTNPNYKGNDIKCTSTSRDKITTL.
BCG67028.1   LDYGQCPNNMMGTFSIRTVGTEK.SPHSITLRVYMRIKHVRAWIPRPLRNQPYLFKTNPNYKGNDIKCTSTSRDKITTL.
BCG67027.1   LDYGQCPNNMMGTFSIRTVGTEK.SPHSITLRVYMRIKHVRAWIPRPLRNQPYLFKTNPNYKGNDIKCTSTSRDKITTL.
BCG66996.1   LDYGQCPNNMMGTFSIRTVGTEK.SPHSITLRVYMRIKHVRAWIPRPLRNQPYLFKTNPNYKGNDIKCTSTSRDKITTL.
BCG67026.1   LDYGQCPNNMMGTFSIRTVGTEK.SPHSITLRVYMRIKHVRAWIPRPLRNQPYLFKTNPNYKGNDIKCTSTSRDKITTL.
BCG67025.1   LDYGQCPNNMMGTFSIRTVGTEK.SPHSITLRVYMRIKHVRAWIPRPLRNQPYLFKTNPNYKGNDIKCTSTSRDKITTL.
BCG67023.1   LDYGQCPNNMMGTFSIRTVGTEK.SPHSITLRVYMRIKHVRAWIPRPLRNQPYLFKTNPNYKGNDIKCTSTSRDKITTL.
BCG67022.1   LDYGQCPNNMMGTFSIRTVGTEK.SPHSITLRVYMRIKHVRAWIPRPLRNQPYLFKTNPNYKGNDIKCTSTSRDKITTL.
BCG67021.1   LDYGQCPNNMMGTFSIRTVGTEK.SPHSITLRVYMRIKHVRAWIPRPLRNQPYLFKTNPNYKGNDIKCTSTSRDKITTL.
BCG67013.1   LDYGQCPNNMMGTFSIRTVGTEK.SPHSITLRVYMRIKHVRAWIPRPLRNQPYLFKTNPNYKGNDIKCTSTSRDKITTL.
BCG67000.1   LDYGQCPNNMMGTFSIRTVGTEK.SPHSITLRVYMRIKHVRAWIPRPLRNQPYLFKTNPNYKGNDIKCTSTSRDKITTL.
BCG66997.1   LDYGQCPNNMMGTFSIRTVGTEK.SPHSITLRVYMRIKHVRAWIPRPLRNQPYLFKTNPNYKGNDIKCTSTSRDKITTL.
BCG66998.1   LDYGQCPNNMMGTFSIRTVGTEK.SPHSITLRVYMRIKHVRAWIPRPLRNQPYLFKTNPNYKGNDIKCTSTSRDKITTL.
QWF08924.1   LDYGQCPNNMMGTFSIRTVGTEK.SPHSITLRVYMRIKHVRAWIPRPLRNQPYLFKTNPNYKGNDIKCTSTSRDKITTL.
QIL87522.1   LDYGQCPNNMMGTFSIRTVGTEK.SPHSITLRVYMRIKHVRAWIPRPLRNQPYLFKTNPNYKGNDIKCTSTSRDKITTL.
QYC65075.1   LDYGQCPNNMMGTFSIRTVGTEK.SPHSITLRVYMRIKHVRAWIPRPLRNQPYLFKTNPNYKGNDIKCTSTSRDKITTL.
QWF08781.1   LDYGQCPNNMMGTFSIRTVGTEK.SPHSITLRVYMRIKHVRAWIPRPLRNQPYLFKTNPNYKGNDIKCTSTSRDKITTL.
QWY15817.1   LDYGQCPNNMMGTFSIRTVGTEK.SPHSITLRVYMRIKHVRAWIPRPLRNQPYLFKTNPNYKGNDFKCTSTSRDKITTL.
QWF08897.1   LDYGQCPNNMMGTFSIRTVGTEK.SPHSITLRVYMRIKHVRAWIPRPLRNQPYLFKTNPNYKGNDIKCTSTSRDKITTL.
QWY15815.1   LDYGQCPNNMMGTFSIRTVGTEK.SPHSITLRVYMRIKHVRAWIPRPLRNQPYLFKTNPNYKGNDIKCTSTSRDKITTL.
QWY15814.1   LDYGQCPNNMMGTFSIRTVGTEK.SPHSITLRVYMRIKHVRAWIPRPLRNQPYLFKTNPNYKGNDIKCTSTSRDKITTL.
QWY15718.1   LDYGQCPNNMMGTFSIRTVGTEK.SPHSITLRVYMRIKHVRAWIPRPLRNQPYLFKTNPNYKGNDIKCTSTSRDKITTL.
QYC65077.1   LDYGQCPNNMMGTFSIRTVGTEK.SPHSITLRVYMRIKHVRAWIPRPLRNQPYLFKTNPNYKGNDIKCTSTSRDKITTL.
QTA73897.1   LDYGQCPNNMMGTFSIRTVGTEK.SPHSITLRVYMRIKHVRAWIPRPLRNQPYLFKTNPNYKGNDIKCTSASRDKITTL.
QWY15676.1   LDYGQCPNNMMGTFSIRTVGTEK.SPHSITLRVYMRIKHVRAWIPRPLRNQPYLFKTNPNYKGNDIKCTSASRDKITTL.
QOP74939.1   LDYGQCPNNMMGTFSIRTVGTEK.SPHSITLRVYMRIKHVRAWIPRPLRNQPYLFKTNPNYKGNDIKCTSASRDKITT..
QOP74936.1   LDYGQCPNNMMGTFSIRTVGTEK.SPHSITLRVYMRIKHVRAWIPRPLRNQPYLFKTNPNYKGNDIKCTSASRDKITT..
QWF08729.1   LDYGQCPNNMMGTFSIRTVGTEK.SPHSITLRVYMRIKHVRAWIPRPLRNQPYLFKTNPNYKGNDIKCTSASRDKITTL.
QYC65064.1   LDYGQCPNNMMGTFSIRTVGTEK.SPHSITLRVYMRIKHVRAWIPRPLRNQPYLFKTNPNYKGNDIKCTSASRDKITTL.
QWF08775.1   LDYGQCPNNMMGTFSIRTVGTEK.SPHSITLRVYMRIKHVRAWIPRPLRNQPYLFKTNPNYKGNDIKCTSASRDKITTL.
QWF08774.1   LDYGQCPNNMMGTFSIRTVGTEK.SPHSITLRVYMRIKHVRAWIPRPLRNQPYLFKTNPNYKGNDIKCTSASRDKITTL.
QWF08753.1   LDYGQCPNNMMGTFSIRTVGTEK.SPHSITLRVYMRIKHVRAWIPRPLRNQPYLFKTNPNYKGNDIKCTSASRDKITTL.
QWY15804.1   LDYGQCPNNMMGTFSVRTVGTEK.SPHSITLRVYMRIKHVRAWIPRPLRNQPYLFKTNPNYKGNDIKCTSASRDKITTL.
QOP74943.1   LDYGQCPNNMMGTFSVRTVGTEK.SPHSITLRVYMRIKHVRAWIPRPLRNQPYLFKTNPNYKGNDIKCTSTSRDKITT..
QIH53920.1   LDYGQCPNNMMGTFSVRTVGTEK.SPHSITLRVYMRIKHVRAWIPRPLRNQPYLFKTNPNYKGNDIKCTSTSRDKITTL.
QIH53919.1   LDYGQCPNNMMGTFSVRTVGTEK.SPHSITLRVYMRIKHVRAWIPRPLRNQPYLFKTNPNYKGNDIKCTSTSRDKITTL.
QIL87536.1   LDYGQCPNNMMGTFSVRTVGTEK.SPHSITLRVYMRIKHVRAWIPRPLRNQPYLFKTNPNYKGNDIKCTSTSRDKITTL.
UEC49854.1   LDYGQCPNNMMGTFSVRTVGTEK.SPHSITLRVYMRIKHVRAWIPRPLRNQPYLFKTNPNYKGNDIKCTSTSRDKITTL.
UEC49852.1   LDYGQCPNNMMGTFSVRTVGTEK.SPHSITLRVYMRIKHVRAWIPRPLRNQPYLFKTNPNYKGNDIKCTSTSRDKITTL.
QWY15841.1   LDYGQCPNNMMGTFSVRTVGTEK.SPHSITLRVYMRIKHVRAWIPRPLRNQPYLFKTNPNYKGNDIKCTSTSRDKITTL.
QWY15787.1   LDYGQCPNNMMGTFSVRTVGTEK.SPHSITLRVYMRIKHVRAWIPRPLRNQPYLFKTNPNYKGNDIKCTSTSRDKITTL.
QYC65128.1   LDYGQCPNNMMGTFSVRTVGTEK.SPHSITLRVYMRIKHVRAWIPRPLRNQPYLFKTNPNYKGNDIKCTSTSRDKITTL.
QYC65116.1   LDYGQCPNNMMGTFSVRTVGTEK.SPHSITLRVYMRIKHVRAWIPRPLRNQPYLFKTNPNYKGNDIKCTSTSRDKITTL.
QYC65083.1   LDYGQCPNNMMGTFSVRTVGTEK.SPHSITLRVYMRIKHVRAWIPRPLRNQPYLFKTNPNYKGNDIKCTSTSRDKITTL.
QYC65029.1   LDYGQCPNNMMGTFSVRTVGTEK.SPHSITLRVYMRIKHVRAWIPRPLRNQPYLFKTNPNYKGNDIKCTSTSRDKITTL.
QWF08951.1   LDYGQCPNNMMGTFSVRTVGTEK.SPHSITLRVYMRIKHVRAWIPRPLRNQPYLFKTNPNYKGNDIKCTSTSRDKITTL.
QWF08937.1   LDYGQCPNNMMGTFSVRTVGTEK.SPHSITLRVYMRIKHVRAWIPRPLRNQPYLFKTNPNYKGNDIKCTSTSRDKITTL.
QWF08918.1   LDYGQCPNNMMGTFSVRTVGTEK.SPHSITLRVYMRIKHVRAWIPRPLRNQPYLFKTNPNYKGNDIKCTSTSRDKITTL.
QWF08901.1   LDYGQCPNNMMGTFSVRTVGTEK.SPHSITLRVYMRIKHVRAWIPRPLRNQPYLFKTNPNYKGNDIKCTSTSRDKITTL.
QWF08834.1   LDYGQCPNNMMGTFSVRTVGTEK.SPHSITLRVYMRIKHVRAWIPRPLRNQPYLFKTNPNYKGNDIKCTSTSRDKITTL.
QWF08832.1   LDYGQCPNNMMGTFSVRTVGTEK.SPHSITLRVYMRIKHVRAWIPRPLRNQPYLFKTNPNYKGNDIKCTSTSRDKITTL.
QWF08802.1   LDYGQCPNNMMGTFSVRTVGTEK.SPHSITLRVYMRIKHVRAWIPRPLRNQPYLFKTNPNYKGNDIKCTSTSRDKITTL.
QWF08800.1   LDYGQCPNNMMGTFSVRTVGTEK.SPHSITLRVYMRIKHVRAWIPRPLRNQPYLFKTNPNYKGNDIKCTSTSRDKITTL.
QYC65123.1   LDYGQCPNNMMGTFSVRTVGTEK.SPHSITLRVYMRVKHVRAWIPRPLRNQPYLFKTNPNYKGNDIKCTSTSRDKITTL.
QWY15820.1   LDYGQCPNNMMGTFSVRTVGTEK.SPHSITLRVYMRIKHVRAWIPRPLRNQPYLFKTNPNYKGNDIKCTSTSRDKITTL.
QWY15864.1   LDYGQCPNNMMGTFSVRTVGTEK.SPHSITLRLYMRIKHVRAWIPRPLRNQPYLFKTNPNYKGNDIKCTSTSRDKITTL.
QWY15840.1   LDYGQCPNNMMGTFSVRTVGTEK.SPHSITLRLYMRIKHVRAWIPRPLRNQPYLFKTNPNYKGNDIKCTSTSRDKITTL.
QWY15786.1   LDYGQCPNNMMGTFSVRTVGTEK.SPHSITLRVYMRIKHVRAWIPRPLRNQPYLFKTNPNYKGNDIKCTSTSRDKITTL.
QWY15842.1   LDYGQCPNNMMGTFSVRTVGTEK.SPHSITLRVYMRIKHVRAWIPRPLRNQPYLFKTNPNYKGNDIKCTSTSRDKITTL.
QWY15704.1   LDYGQCPNNMMGTFSVRTVGTEK.SPHSITLRVYMRIKHVRAWIPRPLRNQPYLFKTNPNYKGNDIKCTSTSRDKITTL.
QWY15750.1   LDYGQCPNNMMGTFSVRTVGTEK.SPHSITLRVYMRIKHVRAWIPRPLRNQPYLFKTNPNYKGNDIKCTSTSRDKITTL.
QWF08733.1   LDYGQCPNNMMGTFSVRTVGTEK.SPHSITLRVYMRIKHVRAWIPRPLRNQPYLFKTNPNYKGNDIRCTSTSRDKITTL.
QNL13228.1   LDYGQCPNNMMGTFSVRTVGAEK.SPHSITLRVYMRIKHVRAWIPRPLRNQPYLFKTNPNYKGNDIKCTSTSRDKITTL.
QNL13216.1   LDYGQCPNNMMGTFSVRTVGAEK.SPHSITLRVYMRIKHVRAWIPRPLRNQPYLFKTNPNYKGNDIKCTSTSRDKITTL.
QNL13226.1   LDYGQCPNNMMGTFSVRTVGAEK.SPHSITLRVYMRIKHVRAWIPRPLRNQPYLFKTNPNYKGNDIKCTSTSRDKITTL.
QNL13203.1   LDYGQCPNNMMGTFSVRTVGAEK.SPHSITLRVYMRIKHVRAWIPRPLRNQPYLFKTNPNYKGNDIKCTSTSRDKITTL.
```

```
QYC65080.1    LDYGQCPNNMMGTFSVRTVGAEK.SPHSITLRVYMRIKHVRAWIPRPLRNQPYLFKTNPNYKGNDIKCTSTSRDKITTL.
QNL13225.1    LDYGQCPNNMMGTFSVRTVGAEK.SPHSITLRVYMRIKHVRAWIPRPLRNQPYLFKTNPNYKGNDIKCTSTSRDKITTL.
QNL13224.1    LDYGQCPNNMMGTFSVRTVGAEK.SPHSITLRVYMRIKHVRAWIPRPLRNQPYLFKTNPNYKGNDIKCTSTSRDKITTL.
QNL13223.1    LDYGQCPNNMMGTFSVRTVGAEK.SPHSITLRVYMRIKHVRAWIPRPLRNQPYLFKTNPNYKGNDIKCTSTSRDKITTL.
QNL13222.1    LDYGQCPNNMMGTFSVRTVGAEK.SPHSITLRVYMRIKHVRAWIPRPLRNQPYLFKTNPNYKGNDIKCTSTSRDKITTL.
QNL13221.1    LDYGQCPNNMMGTFSVRTVGAEK.SPHSITLRVYMRIKHVRAWIPRPLRNQPYLFKTNPNYKGNDIKCTSTSRDKITTL.
QNL13220.1    LDYGQCPNNMMGTFSVRTVGAEK.SPHSITLRVYMRIKHVRAWIPRPLRNQPYLFKTNPNYKGNDIKCTSTSRDKITTL.
QNL13219.1    LDYGQCPNNMMGTFSVRTVGAEK.SPHSITLRVYMRIKHVRAWIPRPLRNQPYLFKTNPNYKGNDIKCTSTSRDKITTL.
QWF08894.1    LDYGQCPNNMMGTFSVRTVGAEK.SPHSITLRVYMRIKHVRAWIPRPLRNQPYLFKTNPNYKGNDIKCTSTSRDKITTL.
QNL13218.1    LDYGQCPNNMMGTFSVRTVGAEK.SPHSITLRVYMRIKHVRAWIPRPLRNQPYLFKTNPNYKGNDIKCTSTSRDKITTL.
QNL13217.1    LDYGQCPNNMMGTFSVRTVGAEK.SPHSITLRVYMRIKHVRAWIPRPLRNQPYLFKTNPNYKGNDIKCTSTSRDKITTL.
QNL13215.1    LDYGQCPNNMMGTFSVRTVGAEK.SPHSITLRVYMRIKHVRAWIPRPLRNQPYLFKTNPNYKGNDIKCTSTSRDKITTL.
QNL13214.1    LDYGQCPNNMMGTFSVRTVGAEK.SPHSITLRVYMRIKHVRAWIPRPLRNQPYLFKTNPNYKGNDIKCTSTSRDKITTL.
QNL13213.1    LDYGQCPNNMMGTFSVRTVGAEK.SPHSITLRVYMRIKHVRAWIPRPLRNQPYLFKTNPNYKGNDIKCTSTSRDKITTL.
QNL13212.1    LDYGQCPNNMMGTFSVRTVGAEK.SPHSITLRVYMRIKHVRAWIPRPLRNQPYLFKTNPNYKGNDIKCTSTSRDKITTL.
QNL13209.1    LDYGQCPNNMMGTFSVRTVGAEK.SPHSITLRVYMRIKHVRAWIPRPLRNQPYLFKTNPNYKGNDIKCTSTSRDKITTL.
QIL87537.1    LDYGQCPNNMMGTFSVRTVGAEK.SPHSITLRVYMRIKHVRAWIPRPLRNQPYLFKTNPNYKGNDIKCTSTSRDKITTL.
QNL13208.1    LDYGQCPNNMMGTFSVRTVGAEK.SPHSITLRVYMRIKHVRAWIPRPLRNQPYLFKTNPNYKGNDIKCTSTSRDKITTL.
QNL13207.1    LDYGQCPNNMMGTFSVRTVGAEK.SPHSITLRVYMRIKHVRAWIPRPLRNQPYLFKTNPNYKGNDIKCTSTSRDKITTL.
QNL13206.1    LDYGQCPNNMMGTFSVRTVGAEK.SPHSITLRVYMRIKHVRAWIPRPLRNQPYLFKTNPNYKGNDIKCTSTSRDKITTL.
QNL13202.1    LDYGQCPNNMMGTFSVRTVGAEK.SPHSITLRVYMRIKHVRAWIPRPLRNQPYLFKTNPNYKGNDIKCTSTSRDKITTL.
QNL13196.1    LDYGQCPNNMMGTFSVRTVGAEK.SPHSITLRVYMRIKHVRAWIPRPLRNQPYLFKTNPNYKGNDIKCTSTSRDKITTL.
QNL13195.1    LDYGQCPNNMMGTFSVRTVGAEK.SPHSITLRVYMRIKHVRAWIPRPLRNQPYLFKTNPNYKGNDIKCTSTSRDKITTL.
QNL13194.1    LDYGQCPNNMMGTFSVRTVGAEK.SPHSITLRVYMRIKHVRAWIPRPLRNQPYLFKTNPNYKGNDIKCTSTSRDKITTL.
QNL13193.1    LDYGQCPNNMMGTFSVRTVGAEK.SPHSITLRVYMRIKHVRAWIPRPLRNQPYLFKTNPNYKGNDIKCTSTSRDKITTL.
QNL13192.1    LDYGQCPNNMMGTFSVRTVGAEK.SPHSITLRVYMRIKHVRAWIPRPLRNQPYLFKTNPNYKGNDIKCTSTSRDKITTL.
QNL13191.1    LDYGQCPNNMMGTFSVRTVGAEK.SPHSITLRVYMRIKHVRAWIPRPLRNQPYLFKTNPNYKGNDIKCTSTSRDKITTL.
QNL13190.1    LDYGQCPNNMMGTFSVRTVGAEK.SPHSITLRVYMRIKHVRAWIPRPLRNQPYLFKTNPNYKGNDIKCTSTSRDKITTL.
QNL13189.1    LDYGQCPNNMMGTFSVRTVGAEK.SPHSITLRVYMRIKHVRAWIPRPLRNQPYLFKTNPNYKGNDIKCTSTSRDKITTL.
QNL13188.1    LDYGQCPNNMMGTFSVRTVGAEK.SPHSITLRVYMRIKHVRAWIPRPLRNQPYLFKTNPNYKGNDIKCTSTSRDKITTL.
QCY53846.1    LDYGQCPNNMMGTFSVRTVGAEK.SPHSITLRVYMRIKHVRAWIPRPLRNQPYLFKTNPNYKGNDIKCTSTSRDKITTL.
BCI65357.1    LDYGQCPNNMMGTFSVRTVGAEK.SPHSITLRVYMRIKHVRAWIPRPLRNQPYLFKTNPNYKGNDIKCTSTSRDKITTL.
QNL13227.1    LDYGQCPNNMMGTFSVRTVGAEK.SPHSITLRVYMRIKHVRAWIPRPLRNQPYLFKTNPNYKGNDIKCTSTSRDKITTL.
QNL13211.1    LDYGQCPNNMMGTFSVRTVGAEK.SPHSITLRVYMRIKHVRAWIPRPLRNQPYLFKTNPNYKGNDIKCTSTSRDKITTL.
QNL13198.1    LDYGQCPNNMMGTFSVRTVGAEK.SPHSITLRVYMRIKHVRAWIPRPLRNQPYLFKTNPNYKGNDIKCTSTSRDKITTL.
QNL13197.1    LDYGQCPNNMMGTFSVRTVGAEK.SPHSITLRVYMRIKHVRAWIPRPLRNQPYLFKTNPNYKGNDIKCTSTSRDKITTL.
QNL13200.1    LDYGQCPNNMMGTFSVRTVGAEK.SPHSITLRVYMRIKHVRAWIPRPLRNQPYLFKTNPNYKGNDIKCTSTSRDKITTL.
QNL13210.1    LDYGQCPNNMMGTFSVRTVGAEK.SPHSITLRVYMRIKHVRAWIPRPLRNQPYLFKTNPNYKGNDIKCTSTSRDKITTL.
QNL13205.1    LDYGQCPNNMMGTFSVRTVGAEK.SPHSITLRVYMRIKHVRAWIPRPLRNQPYLFKTNPNYKGNDIKCTSTSRDKITTL.
QNL13204.1    LDYGQCPNNMMGTFSVRTVGAEK.SPHSITLRVYMRIKHVRAWIPRPLRNQPYLFKTNPNYKGNDIKCTSTSRDKITTL.
QNL13201.1    LDYGQCPNNMMGTFSVRTVGAEK.SPHSITLRVYMRIKHVRAWIPRPLRNQPYLFKTNPNYKGNDIKCTSTSRDKITTL.
QNL13199.1    LDYGQCPNNMMGTFSVRTVGAEK.SPHSITLRVYMRIKHVRAWIPRPLRNQPYLFKTNPNYKGNDIKCTSTSRDKITTL.
QYC65157.1    LDYGQCPNNMMGTFSIRTVGTEK.SPHSITLRVYMRIKHVRAWIPRPLRNQPYLFKTNPNYKGNDIKCTSTSRDKITTL.
QYC65138.1    LDYGQCPNNMMGTFSIRTVGTEK.SPHSITLRVYMRIKHVRAWIPRPLRNQPYLFKTNPNYKGNDIKCTSTSRDKITTL.
QWF08724.1    LDYGQCPNNMMGTFSIRTVGTEK.SPHSITLRVYMRIKHVRAWIPRPLRNQPYLFKTNPNYKGNDIKCTSTSRDKITTL.
UEP53403.1    LDYGQCPNNMMGTFSIRTVGTEK.SPHSITLRVYMRIKHVRAWIPRPLRNQPYLFKTNPNYKGNDIKCTSTSRDKITTL.
QWY15843.1    LDYGQCPNNMMGTFSVRTVGTKK.SPHSITLRVYMRIKHVRAWIPRPLRNQPYLFKTNPNYKGNDIKCTSTSRDKITTL.
QWF08794.1    LDYGQCPNNMMGTFSVRTVGTEK.SPHSITLRVYMRIKHVRAWIPRPLRNQPYLFKTNPNYKGNDIKCTSTSRDKITTL.
QYC65031.1    LDYGQCPNNMMGTFSIRTVGTEK.SPHSITLRVYMRIKHVRAWIPRPLRNQPYLFKTNPNYKGNDIKCTSTSRDKITTL.
UEC49856.1    LDYGQCPNNMMGTFSIRTVGTEK.SPHSITLRVYMRIKHVRAWIPRPLRNQPYLFKTNPNYKGNDIKCTSTSRDKITTL.
BBH72184.1    LDYGQCPNNMMGTFSIRTVGTEK.SPHSITLRVYMRIKHVRAWIPRPLRNQPYLFKTNPNYKGNDIKCTSTSRDKITTL.
QWF08874.1    LDYGQCPNNMMGTFSIRTVGTEK.SPHSITLRVYMRIKHVRAWIPRPLRNQPYLFKTNPNYKGNDIKCTSTSRDKITTL.
QWF08873.1    LDYGQCPNNMMGTFSIRTVGTEK.SPHSITLRVYMRIKHVRAWIPRPLRNQPYLFKTNPNYKGNDIKCTSTSRDKITTL.
BCG67003.1    LDYGQCPNNMMGTFSIRTVGTEK.SPHSITLRVYMRIKHVRAWIPRPLRNQPYLFKTNPNYKGNDIKCTSTSRDKITTL.
QYC65095.1    LDYGQCPNNMMGTFSIRTVGTEK.SPHSITLRVYMRIKHVRAWIPRPLRNQPYLFKTNPNYKGNDIKCTSTSRDKITTL.
QOP74933.1    LDYGQCPNNMMGTFSIRTVGTEK.SPHSITLRVYMRIKHVRAWIPRPLRNQPYLFKTNPNYKGNDIKCTSTSRDKITT..
QYC65096.1    LDYGQCPNNMMGTFSIRTVGTEK.SPHSITLRVYMRIKHVRAWIPRPLRNQPYLFKTNPNYKGNDIKCTSTSRDKITTL.
BCG67034.1    LDYGQCPNNMMGTFSIRTVGTEK.SPHSITLRVYMRIKHVRAWIPRPLRNQPYLFKTNPNYKGNDIKCTSTSRDKITTL.
BCG67031.1    LDYGQCPNNMMGTFSIRTVGTEK.SPHSITLRVYMRIKHVRAWIPRPLRNQPYLFKTNPNYKGNDIKCTSTSRDKITTL.
QYC65052.1    LDYGQCPNNMMGTFSIRTVGTEK.SPHSITLRVYMRIKHVRAWIPRPLRNQPYLFKTNPNYKGNDIKCTSTSRDKITTL.
QWF08963.1    LDYGQCPNNMMGTFSIRTVGTEK.SPHSITLRVYMRIKHVRAWIPRPLRNQPYLFKTNPNYKGNDIKCTSTSRDKITTL.
QWF08939.1    LDYGQCPNNMMGTFSIRTVGTEK.SPHSITLRVYMRIKHVRAWIPRPLRNQPYLFKTNPNYKGNDIKCTSTSRDKITTL.
QIL87538.1    LDYGQCPNNMMGTFSIRTVGTEK.SPHSITLRVYMRIKHVRAWIPRPLRNQPYLFKTNPNYKGNDIKCTSTSRDKITTL.
QIL87535.1    LDYGQCPNNMMGTFSIRTVGTEK.SPHSITLRVYMRIKHVRAWIPRPLRNQPYLFKTNPNYKGNDIKCTSTSRDKITTL.
QWF08964.1    LDYGQCPNNMMGTFSIRTVGTEK.SPHSITLRVYMRIKHVRAWIPRPLRNQPYLFKTNPNYKGNDIKCTSTSRDKITTL.
QYC65132.1    LDYGQCPNNMMGTFSIRTVGTEK.SPHSITLRVYMRIKHVRAWIPRPLRNQPYLFKTNPNYKGNDIKCTSTSRDKITTL.
QTA73893.1    LDYGQCPNNMMGTFSIRTVGTEK.SPHSITLRVYMRIKHVRAWIPRPLRNQPYLFKTNPNYKGNDIKCTSTSRDKITTL.
QWF08751.1    LDYGQCPNNMMGTFSIRTVGTEK.SPHSITLRVYMRIKHVRAWIPRPLRNQPYLFKTNPNYKGNDIKCTSTSRDKITTL.
QWY15860.1    LDYGQCPNNMMGTFSIRTVGTEK.SPHSITLRVYMRIKHVRAWIPRPLRNQPYLFKTNPNYKGNDIKCTSTSRDKITTL.
QWY15749.1    LDYGQCPNNMMGTFSIRTVGTEK.SPHSITLRVYMRIKHVRAWIPRPLRNQPYLFKTNPNYKGNDIKCTSTSRDKITTL.
QWY15744.1    LDYGQCPNNMMGTFSIRTVGTEK.SPHSITLRVYMRIKHVRAWIPRPLRNQPYLFKTNPNYKGNDIKCTSTSRDKITTL.
QWY15740.1    LDYGQCPNNMMGTFSIRTVGTEK.SPHSITLRVYMRIKHVRAWIPRPLRNQPYLFKTNPNYKGNDIKCTSTSRDKITTL.
QWY15700.1    LDYGQCPNNMMGTFSIRTVGTEK.SPHSITLRVYMRIKHVRAWIPRPLRNQPYLFKTNPNYKGNDIKCTSTSRDKITTL.
QWY15698.1    LDYGQCPNNMMGTFSIRTVGTEK.SPHSITLRVYMRIKHVRAWIPRPLRNQPYLFKTNPNYKGNDIKCTSTSRDKITTL.
QWY15672.1    LDYGQCPNNMMGTFSIRTVGTEK.SPHSITLRVYMRIKHVRAWIPRPLRNQPYLFKTNPNYKGNDIKCTSTSRDKITTL.
pdb|6LHT|B    LDYGQCPNNMMGTFSIRTVGTEK.SPHSITLRVYMRIKHVRAWIPRPLRNQPYLFKTNPNYKGNDIKCTSTSRDKITTL.
pdb|6LHT|E    LDYGQCPNNMMGTFSIRTVGTEK.SPHSITLRVYMRIKHVRAWIPRPLRNQPYLFKTNPNYKGNDIKCTSTSRDKITTL.
pdb|6LHT|D    LDYGQCPNNMMGTFSIRTVGTEK.SPHSITLRVYMRIKHVRAWIPRPLRNQPYLFKTNPNYKGNDIKCTSTSRDKITTL.
pdb|6LHT|C    LDYGQCPNNMMGTFSIRTVGTEK.SPHSITLRVYMRIKHVRAWIPRPLRNQPYLFKTNPNYKGNDIKCTSTSRDKITTL.
pdb|6LHT|A    LDYGQCPNNMMGTFSIRTVGTEK.SPHSITLRVYMRIKHVRAWIPRPLRNQPYLFKTNPNYKGNDIKCTSTSRDKITTL.
pdb|6LHQ|A    LDYGQCPNNMMGTFSIRTVGTEK.SPHSITLRVYMRIKHVRAWIPRPLRNQPYLFKTNPNYKGNDIKCTSTSRDKITTL.
pdb|6LHP|A    LDYGQCPNNMMGTFSIRTVGTEK.SPHSITLRVYMRIKHVRAWIPRPLRNQPYLFKTNPNYKGNDIKCTSTSRDKITTL.
pdb|6LHO|A    LDYGQCPNNMMGTFSIRTVGTEK.SPHSITLRVYMRIKHVRAWIPRPLRNQPYLFKTNPNYKGNDIKCTSTSRDKITTL.
pdb|6LHL|A    LDYGQCPNNMMGTFSIRTVGTEK.SPHSITLRVYMRIKHVRAWIPRPLRNQPYLFKTNPNYKGNDIKCTSTSRDKITTL.
pdb|6LHK|A    LDYGQCPNNMMGTFSIRTVGTEK.SPHSITLRVYMRIKHVRAWIPRPLRNQPYLFKTNPNYKGNDIKCTSTSRDKITTL.
pdb|6LHC|A    LDYGQCPNNMMGTFSIRTVGTEK.SPHSITLRVYMRIKHVRAWIPRPLRNQPYLFKTNPNYKGNDIKCTSTSRDKITTL.
pdb|6LHB|A    LDYGQCPNNMMGTFSIRTVGTEK.SPHSITLRVYMRIKHVRAWIPRPLRNQPYLFKTNPNYKGNDIKCTSTSRDKITTL.
pdb|6LHA|A    LDYGQCPNNMMGTFSIRTVGTEK.SPHSITLRVYMRIKHVRAWIPRPLRNQPYLFKTNPNYKGNDIKCTSTSRDKITTL.
QWY15708.1    LDYGQCPNNMMGTFSIRTVGTEK.SPHSITLRVYMRIKHVRAWIPRPLRNQPYLFKTNPNYKGNDIKCPSTSRDKITTL.
BBH72101.1    LDYGQCPNNMMGTFSIRTVGTEK.SPHSITLRVYMRIKHVRAWIPRPLRNQPYLFKTNPNYKGNDINSSFNRRDNLTTL.
BBH72090.1    LDYGQCPNNMMGTFSIRTVGTEK.SPHSITLRVYMRIKHVRAWIPRPLRNQPYLFKTNPNYKGNDINCTSTSRDKLTTL.
BBH72097.1    LDYGQCPNNMMGTFSIRTVGTEK.SPHSITLRVYMRIKHVRAWIPRPLRNQPYLFKTNPNYKGNDINCTSTSRDKLTTL.
BBH72089.1    LDYGQCPNNMMGTFSIRTVGTEK.SPHSITLRVYMRIKHVRAWIPRPLRNQPYLFKTNPNYKGNDINCTSTSRDKITTL.
BBH72099.1    LDYGQCPNNMMGTFSIRTVGTEK.SPHSITLRVYMRIKHVRAWIPRPLRNQPYLFKTNPNYKGNDINCTSTSRDKLTTL.
BBH72093.1    LDYGQCPNNMMGTFSIRTVGTEK.SPHSITLRVYMRIKHVRAWIPRPLRNQPYLFKTNPNYKGNDINCTSTSRDKITTL.
BBH72096.1    LDYGQCPNNMMGTFSIRTVGTEK.SPHSITLRVYMRIKHVRAWIPRPLRNQPYLFKTNPNYKGSDINCTSTSRDKITTL.
BBH72094.1    LDYGQCPNNMMGTFSIRTVGTEK.SPHSITLRVYMRIKHVRAWIPRPLRNQPYLFKTNPNYKGNDINCTSTSRDKITTL.
BBH72091.1    LDYGQCPNNMMGTFSIRTVGTEK.SPHSITLRVYMRIKHVRAWIPRPLRNQPYLFKTNPNYKGNDINCTSTSRDKITTL.
QOP74916.1    LDYGQCPNNMMGTFSIRTVGIEK.SPHSITLRVYMRIKHVRAWIPRPLRNQPYLFKTNPNYKGNDIKCTSTSRDKITT..
QYC65163.1    LDYGQCPNNMMGTFSIRTVGIEK.SPHSITLRVYMRIKHVRAWIPRPLRNQPYLFKTNPNYKGNDIKCTSTSRDKITTL.
QWF08736.1    LDYGQCPNNMMGTFSIRTVGIEK.SPHSITLRVYMRIKHVRAWIPRPLRNQPYLFKTNPNYKGNDIKCTSTSRDKITTL.
QWF08735.1    LDYGQCPNNMMGTFSIRTVGIEK.SPHSITLRVYMRIKHVRAWIPRPLRNQPYLFKTNPNYKGNDIKCTSTSRDKITTL.
pdb|5TSK|A    LDYGQCPNNMMGTFSIRTVGIEK.SPHSITLRVYMRIKHVRAWIPRPLRNQPYLFKTNPNYKGNDIKCTSTSRDKITTL.
pdb|5C9A|A    LDYGQCPNNMMGTFSIRTVGIEK.SPHSITLRVYMRIKHVRAWIPRPLRNQPYLFKTNPNYKGNDIKCTSTSRDKITTL.
pdb|5C4W|A    LDYGQCPNNMMGTFSIRTVGIEK.SPHSITLRVYMRIKHVRAWIPRPLRNQPYLFKTNPNYKGNDIKCTSTSRDKITTL.
pdb|4JGZ|A    LDYGQCPNNMMGTFSIRTVGIEK.SPHSITLRVYMRIKHVRAWIPRPLRNQPYLFKTNPNYKGNDIKCTSTSRDKITTL.
pdb|4JGY|A    LDYGQCPNNMMGTFSIRTVGIEK.SPHSITLRVYMRIKHVRAWIPRPLRNQPYLFKTNPNYKGNDIKCTSTSRDKITTL.
QWY15956.1    LDYGQCPNNMMGTFSIRTVGIEK.SPHSITLRVYMRIKHVRAWIPRPLRNQPYLFKTNPNYKGNDIKCTSTSRDKITTL.
QWF08811.1    LDYGQCPNNMMGTFSIRTVGIEK.SPHSITLRVYMRIKHVRAWIPRPLRNQPYLFKTNPNYKGNDIKCTSTSRDKITTL.
QWF08878.1    LDYGQCPNNMMGTFSIRTVGTEK.SPHSITLRVYMRIKHVRAWIPRPLRNQPYLFKTNPNYKGNDIKCTSTSRDKITTL.
QWF08869.1    LDYGQCPNNMMGTFSIRTVGTEK.SPHSITLRVYMRIKHVRAWIPRPLRNQPYLFKTNPNYKGNDIKCTSTSRDKITTL.
QWF08831.1    LDYGQCPNNMMGTFSIRTVGTEK.SPHSITLRVYMRIKHVRAWIPRPLRNQPYLFKTNPNYKGNDIKCTSTSRDKITTL.
QWF08829.1    LDYGQCPNNMMGTFSIRTVGTEK.SPHSITLRVYMRIKHVRAWIPRPLRNQPYLFKTNPNYKGNDIKCTSTSRDKITTL.
QWF08824.1    LDYGQCPNNMMGTFSIRTVGTEK.SPHSITLRVYMRIKHVRAWIPRPLRNQPYLFKTNPNYKGNDIKCTSTSRDKITTL.
QWF08822.1    LDYGQCPNNMMGTFSIRTVGTEK.SPHSITLRVYMRIKHVRAWIPRPLRNQPYLFKTNPNYKGNDIKCTSTSRDKITTL.
QWF08817.1    LDYGQCPNNMMGTFSIRTVGTEK.SPHSITLRVYMRIKHVRAWIPRPLRNQPYLFKTNPNYKGNDIKCTSTSRDKITTL.
QWF08816.1    LDYGQCPNNMMGTFSIRTVGTEK.SPHSITLRVYMRIKHVRAWIPRPLRNQPYLFKTNPNYKGNDIKCTSTSRDKITTL.
QWF08798.1    LDYGQCPNNMMGTFSIRTVGTEK.SPHSITLRVYMRIKHVRAWIPRPLRNQPYLFKTNPNYKGNDIKCTSTSRDKITTL.
QWF08779.1    LDYGQCPNNMMGTFSIRTVGTEK.SPHSITLRVYMRIKHVRAWIPRPLRNQPYLFKTNPNYKGNDIKCTSTSRDKITTL.
QWF08778.1    LDYGQCPNNMMGTFSIRTVGTEK.SPHSITLRVYMRIKHVRAWIPRPLRNQPYLFKTNPNYKGNDIKCTSTSRDKITTL.
QWF08772.1    LDYGQCPNNMMGTFSIRTVGTEK.SPHSITLRVYMRIKHVRAWIPRPLRNQPYLFKTNPNYKGNDIKCTSTSRDKITTL.
QWF08771.1    LDYGQCPNNMMGTFSIRTVGTEK.SPHSITLRVYMRIKHVRAWIPRPLRNQPYLFKTNPNYKGNDIKCTSTSRDKITTL.
QWF08745.1    LDYGQCPNNMMGTFSIRTVGTEK.SPHSITLRVYMRIKHVRAWIPRPLRNQPYLFKTNPNYKGNDIKCTSTSRDKITTL.
QWF08744.1    LDYGQCPNNMMGTFSIRTVGTEK.SPHSITLRVYMRIKHVRAWIPRPLRNQPYLFKTNPNYKGNDIKCTSTSRDKITTL.
QIH53922.1    LDYGQCPNNMMGTFSIRTVGTEK.SPHSITLRVYMRIKHVRAWIPRPLRNQPYLFKTNPNYKGNDIKCTSTSRDKITTL.
QOP74941.1    LDYGQCPNNMMGTFSIRTVGTEK.SPHSITLRVYMRIKHVRAWIPRPLRNQPYLFKTNPNYKGNDIKCTSTSRDKITT..
QWY15812.1    LDYGQCPNNMMGTFSIRTVGTEK.SPHSITLRVYMRIKHVRAWIPRPLRNQPYLFKTNPNYKGNDIKCTSTSRDKITTL.
QOP74926.1    LDYGQCPNNMMGTFSIRTVGTEK.SPHSITLRVYMRIKHVRAWIPRPLRNQPYLFKTNPNYKGNDIKCTSTSRDKITT..
QYC65176.1    LDYGQCPNNMMGTFSIRTVGTEK.SPHSITLRVYMRIKHVRAWIPRPLRNQPYLFKTNPNYKGNDIKCTSTSRDKITTL.
QYC65172.1    LDYGQCPNNMMGTFSIRTVGTEK.SPHSITLRVYMRIKHVRAWIPRPLRNQPYLFKTNPNYKGNDIKCTSTSRDKITTL.
QYC65168.1    LDYGQCPNNMMGTFSIRTVGTEK.SPHSITLRVYMRIKHVRAWIPRPLRNQPYLFKTNPNYKGNDIKCTSTSRDKITTL.
QWF08725.1    LDYGQCPNNMMGTFSIRTVGTEK.SPHSITLRVYMRIKHVRAWIPRPLRNQPYLFKTNPNYKGNDIKCTSTSRDKITTL.
QYC65028.1    LDYGQCPNNMMGTFSIRTVGTEK.SPHSITLRVYMRIKHVRAWIPRPLRNQPYLFKTNPNYKGNDIKCTSTSRDKITTL.
QWY15686.1    LDYGQCPNNMMGTFSIRTVGTEK.SPHSITLRVYMRIKHVRAWIPRPLRNQPYLFKTNPNYKGIDIKCTSTSRDKITTL.
QYC65023.1    LDYGQCPNNMMGTFSIRTVGTEK.SPHSITLRVYMRIKHVRAWIPRPLRNQPYLFKTNPNYKGIDIKCTSTSRDKITTL.
QYC65025.1    LDYGQCPNNMMGTFSIRTVGTEK.SPHSITLRVYMRIKHVRAWIPRPLRNQPYLFKTNPNYKGNDIKCTSTSRDKITTL.
QWY15946.1    LDYGQCPNNMMGTFSIRTVGTEK.SPHSITLRVYMRIKHVRAWIPRPLRNQPYLFKTNPNYKGNDIKCTSTSRDKITTL.
BCB92164.1    LDYGQCPNNMMGTFSIRTVGTEK.SPHSITLRVYMRIKHVRAWIPRPLRNQPYLFKTNPNYKGNDIKCTSTSRDKITTL.
QOP74935.1    LDYGQCPNNMMGTFSIRTVGTEK.SPHSITLRVYMRIKHVRAWIPRPLRNQPYLFKTNPNYKGNDIKCTSTSRDKITT..
QOP74930.1    LDYGQCPNNMMGTFSIRTVGTEK.SPHSITLRVYMRIKHVRAWIPRPLRNQPYLFKTNPNYKGNDIKCTSTSRDKITT..
QOP74928.1    LDYGQCPNNMMGTFSIRTVGTEK.SPHSITLRVYMRIKHVRAWIPRPLRNQPYLFKTNPNYKGNDIKCTSTSRDKITT..
QOP74927.1    LDYGQCPNNMMGTFSIRTVGTEK.SPHSITLRVYMRIKHVRAWIPRPLRNQPYLFKTNPNYKGNDIKCTSTSRDKITT..
QOP74925.1    LDYGQCPNNMMGTFSIRTVGTEK.SPHSITLRVYMRIKHVRAWIPRPLRNQPYLFKTNPNYKGNDIKCTSTSRDKITT..
QOP74923.1    LDYGQCPNNMMGTFSIRTVGTEK.SPHSITLRVYMRIKHVRAWIPRPLRNQPYLFKTNPNYKGNDIKCTSTSRDKITT..
```

```
QOP74922.1    LDYGQCPNNMMGTFSIRTVGTEK.SPHSITLRVYMRIKHVRAWIPRPLRNQPYLFKTNPNYKGNDIKCTSTSRDKITT..
QOP74920.1    LDYGQCPNNMMGTFSIRTVGTEK.SPHSITLRVYMRIKHVRAWIPRPLRNQPYLFKTNPNYKGNDIKCTSTSRDKITT..
QOP74919.1    LDYGQCPNNMMGTFSIRTVGTEK.SPHSITLRVYMRIKHVRAWIPRPLRNQPYLFKTNPNYKGNDIKCTSTSRDKITT..
QOP74917.1    LDYGQCPNNMMGTFSIRTVGTEK.SPHSITLRVYMRIKHVRAWIPRPLRNQPYLFKTNPNYKGNDIKCTSTSRDKITT..
QHB49260.1    LDYGQCPNNMMGTFSIRTVGTEK.SPHSITLRVYMRIKHVRAWIPRPLRNQPYLFKTNPNYKGNDIKCTSTSRDKITT..
QWF08784.1    LDYGQCPNNMMGTFSIRTVGTEK.SPHSITLRVYMRIKHVRAWIPRPLRNQPYLFKTNPNYKGNDIKCTSTSRDKITTL.
QWF08782.1    LDYGQCPNNMMGTFSIRTVGTEK.SPHSITLRVYMRIKHVRAWIPRPLRNQPYLFKTNPNYKGNDIKCTSTSRDKITTL.
QWF08777.1    LDYGQCPNNMMGTFSIRTVGTEK.SPHSITLRVYMRIKHVRAWIPRPLRNQPYLFKTNPNYKGNDIKCTSTSRDKITTL.
QWF08757.1    LDYGQCPNNMMGTFSIRTVGTEK.SPHSITLRVYMRIKHVRAWIPRPLRNQPYLFKTNPNYKGNDIKCTSTSRDKITTL.
QWF08756.1    LDYGQCPNNMMGTFSIRTVGTEK.SPHSITLRVYMRIKHVRAWIPRPLRNQPYLFKTNPNYKGNDIKCTSTSRDKITTL.
QWF08755.1    LDYGQCPNNMMGTFSIRTVGTEK.SPHSITLRVYMRIKHVRAWIPRPLRNQPYLFKTNPNYKGNDIKCTSTSRDKITTL.
QWF08791.1    LDYGQCPNNMMGTFSIRTVGTEK.SPHSITLRVYMRIKHVRAWIPRPLRNQPYLFKTNPNYKGNDIKCTSTSRDKITTL.
QWF08786.1    LDYGQCPNNMMGTFSIRTVGTEK.SPHSITLRVYMRIKHVRAWIPRPLRNQPYLFKTNPNYKGNDIKCTSTSRDKITTL.
QYC65186.1    LDYGQCPNNMMGTFSIRTVGTEK.SPHSITLRVYMRIKHVRAWIPRPLRNQPYLFKTNPNYKGNDIKCTSTSRDKITTL.
QWY15722.1    LDYGQCPNNMMGTFSIRTVGTEK.SPHSITLRVYMRIKHVRAWIPRPLRNQPYLFKTNPNYKGNDIKCTSTSRDKITTL.
QYC65182.1    LDYGQCPNNMMGTFSIRTVGTEK.SPHSITLRVYMRIKHVRAWIPRPLRNQPYLFKTNPNYKGNDIKCTSTSRDKITTL.
QWF08754.1    LDYGQCPNNMMGTFSIRTVGTEK.SPHSITLRVYMRIKHVRAWIPRPLRNQPYLFKTNPNYKGNDIKCTSTSRDKITTL.
QWF08748.1    LDYGQCPNNMMGTFSIRTVGTEK.SPHSITLRVYMRIKHVRAWIPRPLRNQPYLFKTNPNYKGNDIKCTSTSRDKITTL.
QYC65181.1    LDYGQCPNNMMGTFSIRTVGTEK.SPHSITLRVYMRIKHVRAWIPRPLRNQPYLFKTNPNYKGNDIKCTSTSRDKITTL.
QWY15719.1    LDYGQCPNNMMGTFSIRTVGTEK.SPHSITLRVYMRIKHVRAWIPRPLRNQPYLFKTNPNYKGNDIKCTSTSRDKITTL.
QYC65180.1    LDYGQCPNNMMGTFSIRTVGTEK.SPHSITLRVYMRIKHVRAWIPRPLRNQPYLFKTNPNYKGNDIKCTSTSRDKITTL.
QWY15693.1    LDYGQCPNNMMGTFSIRTVGTEK.SPHSITLRVYMRIKHVRAWIPRPLRNQPYLFKTNPNYKGNDIKCTSTSRDKITTL.
QYC65179.1    LDYGQCPNNMMGTFSIRTVGTEK.SPHSITLRVYMRIKHVRAWIPRPLRNQPYLFKTNPNYKGNDIKCTSTSRDKITTL.
BCB92156.1    LDYGQCPNNMMGTFSIRTVGTEK.SPHSITLRVYMRIKHVRAWIPRPLRNQPYLFKTNPNYKGNDIKCTSTSRDKITTL.
QYC65178.1    LDYGQCPNNMMGTFSIRTVGTEK.SPHSITLRVYMRIKHVRAWIPRPLRNQPYLFKTNPNYKGNDIKCTSTSRDKITTL.
pdb|5C8C|A    LDYGQCPNNMMGTFSIRTVGTEK.SPHSITLRVYMRIKHVRAWIPRPLRNQPYLFKTNPNYKGNDIKCTSTSRDKITTL.
QWF08864.1    LDYGQCPNNMMGTFSIRTVGTEK.SPHSITLRVYMRIKHVRAWIPRPLRNQPYLFKTNPNYKGNDIKCTSTSRDKITTL.
QWF08747.1    LDYGQCPNNMMGTFSIRTVGTEK.SPHSITLRVYMRIKHVRAWIPRPLRNQPYLFKTNPNYKGNDIKCTSTSRDKITTL.
QWF08743.1    LDYGQCPNNMMGTFSIRTVGTEK.SPHSITLRVYMRIKHVRAWIPRPLRNQPYLFKTNPNYKGNDIKCTSTSRDKITTL.
QWF08741.1    LDYGQCPNNMMGTFSIRTVGTEK.SPHSITLRVYMRIKHVRAWIPRPLRNQPYLFKTNPNYKGNDIKCTSTSRDKITTL.
QWF08737.1    LDYGQCPNNMMGTFSIRTVGTEK.SPHSITLRVYMRIKHVRAWIPRPLRNQPYLFKTNPNYKGNDIKCTSTSRDKITTL.
QWF08734.1    LDYGQCPNNMMGTFSIRTVGTEK.SPHSITLRVYMRIKHVRAWIPRPLRNQPYLFKTNPNYKGNDIKCTSTSRDKITTL.
BBH72119.1    LDYGQCPNNMMGTFSIRTVGTEK.SPHSITLRVYMRIKHVRAWIPRPLRNQPYLFKTNPNYKGNDIKCTSTSRDKITTL.
QYC65177.1    LDYGQCPNNMMGTFSIRTVGTEK.SPHSITLRVYMRIKHVRAWIPRPLRNQPYLFKTNPNYKGNDIKCTSTSRDKITTL.
QWF08732.1    LDYGQCPNNMMGTFSIRTVGTEK.SPHSITLRVYMRIKHVRAWIPRPLRNQPYLFKTNPNYKGNDIKCTSTSRDKITTL.
QWF08730.1    LDYGQCPNNMMGTFSIRTVGTEK.SPHSITLRVYMRIKHVRAWIPRPLRNQPYLFKTNPNYKGNDIKCTSTSRDKITTL.
QYC65175.1    LDYGQCPNNMMGTFSIRTVGTEK.SPHSITLRVYMRIKHVRAWIPRPLRNQPYLFKTNPNYKGNDIKCTSTSRDKITTL.
QWF08727.1    LDYGQCPNNMMGTFSIRTVGTEK.SPHSITLRVYMRIKHVRAWIPRPLRNQPYLFKTNPNYKGNDIKCTSTSRDKITTL.
QYC65173.1    LDYGQCPNNMMGTFSIRTVGTEK.SPHSITLRVYMRIKHVRAWIPRPLRNQPYLFKTNPNYKGNDIKCTSTSRDKITTL.
BBH72105.1    LDYGQCPNNMMGTFSIRTVGTEK.SPHSITLRVYMRIKHVRAWIPRPLRNQPYLFKTNPNYKGNDIKCTSTSRDKITTL.
QYC65171.1    LDYGQCPNNMMGTFSIRTVGTEK.SPHSITLRVYMRIKHVRAWIPRPLRNQPYLFKTNPNYKGNDIKCTSTSRDKITTL.
pdb|5ABJ|A    LDYGQCPNNMMGTFSIRTVGTEK.SPHSITLRVYMRIKHVRAWIPRPLRNQPYLFKTNPNYKGNDIKCTSTSRDKITTL.
QWY15716.1    LDYGQCPNNMMGTFSIRTVGTEK.SPHSITLRVYMRIKHVRAWIPRPLRNQPYLFKTNPNYKGNDIKCTSTSRDKITTL.
QWY15800.1    LDYGQCPNNMMGTFSIRTVGTEK.SPHSITLRVYMRIKHVRAWIPRPLRNQPYLFKTNPNYKGNDIKCTSTSRDKITTL.
QYC65018.1    LDYGQCPNNMMGTFSIRTVGTEK.SPHSITLRVYMRIKHVRAWIPRPLRNQPYLFKTNPNYKGNDIKCTSTSRDKITTL.
QWY15712.1    LDYGQCPNNMMGTFSIRTVGTEK.SPHSITLRVYMRIKHVRAWIPRPLRNQPYLFKTNPNYKGNDIKCTSTSRDKITTL.
QWY15711.1    LDYGQCPNNMMGTFSIRTVGTEK.SPHSITLRVYMRIKHVRAWIPRPLRNQPYLFKTNPNYKGNDIKCTSTSRDKITTL.
QWY15707.1    LDYGQCPNNMMGTFSIRTVGTEK.SPHSITLRVYMRIKHVRAWIPRPLRNQPYLFKTNPNYKGNDIKCTSTSRDKITTL.
QWY15688.1    LDYGQCPNNMMGTFSIRTVGTEK.SPHSITLRVYMRIKHVRAWIPRPLRNQPYLFKTNPNYKGNDIKCTSTSRDKITTL.
QWY15685.1    LDYGQCPNNMMGTFSIRTVGTEK.SPHSITLRVYMRIKHVRAWIPRPLRNQPYLFKTNPNYKGNDIKCTSTSRDKITTL.
QWY15684.1    LDYGQCPNNMMGTFSIRTVGTEK.SPHSITLRVYMRIKHVRAWIPRPLRNQPYLFKTNPNYKGNDIKCTSTSRDKITTL.
QYC65170.1    LDYGQCPNNMMGTFSIRTVGTEK.SPHSITLRVYMRIKHVRAWIPRPLRNQPYLFKTNPNYKGNDIKCTSTSRDKITTL.
QYC65169.1    LDYGQCPNNMMGTFSIRTVGTEK.SPHSITLRVYMRIKHVRAWIPRPLRNQPYLFKTNPNYKGNDIKCTSTSRDKITTL.
QYC65162.1    LDYGQCPNNMMGTFSIRTVGTEK.SPHSITLRVYMRIKHVRAWIPRPLRNQPYLFKTNPNYKGNDIKCTSTSRDKITTL.
QYC65099.1    LDYGQCPNNMMGTFSIRTVGTEK.SPHSITLRVYMRIKHVRAWIPRPLRNQPYLFKTNPNYKGNDIKCTSTSRDKITTL.
QYC65048.1    LDYGQCPNNMMGTFSIRTVGTEK.SPHSITLRVYMRIKHVRAWIPRPLRNQPYLFKTNPNYKGNDIKCTSTSRDKITTL.
QYC65040.1    LDYGQCPNNMMGTFSIRTVGTEK.SPHSITLRVYMRIKHVRAWIPRPLRNQPYLFKTNPNYKGNDIKCTSTSRDKITTL.
QYC65035.1    LDYGQCPNNMMGTFSIRTVGTEK.SPHSITLRVYMRIKHVRAWIPRPLRNQPYLFKTNPNYKGNDIKCTSTSRDKITTL.
QYC65032.1    LDYGQCPNNMMGTFSIRTVGTEK.SPHSITLRVYMRIKHVRAWIPRPLRNQPYLFKTNPNYKGNDIKCTSTSRDKITTL.
QYC65030.1    LDYGQCPNNMMGTFSIRTVGTEK.SPHSITLRVYMRIKHVRAWIPRPLRNQPYLFKTNPNYKGNDIKCTSTSRDKITTL.
QYC65027.1    LDYGQCPNNMMGTFSIRTVGTEK.SPHSITLRVYMRIKHVRAWIPRPLRNQPYLFKTNPNYKGNDIKCTSTSRDKITTL.
QYC65026.1    LDYGQCPNNMMGTFSIRTVGTEK.SPHSITLRVYMRIKHVRAWIPRPLRNQPYLFKTNPNYKGNDIKCTSTSRDKITTL.
QYC65024.1    LDYGQCPNNMMGTFSIRTVGTEK.SPHSITLRVYMRIKHVRAWIPRPLRNQPYLFKTNPNYKGNDIKCTSTSRDKITTL.
QYC65022.1    LDYGQCPNNMMGTFSIRTVGTEK.SPHSITLRVYMRIKHVRAWIPRPLRNQPYLFKTNPNYKGNDIKCTSTSRDKITTL.
QYC65021.1    LDYGQCPNNMMGTFSIRTVGTEK.SPHSITLRVYMRIKHVRAWIPRPLRNQPYLFKTNPNYKGNDIKCTSTSRDKITTL.
QYC65020.1    LDYGQCPNNMMGTFSIRTVGTEK.SPHSITLRVYMRIKHVRAWIPRPLRNQPYLFKTNPNYKGNDIKCTSTSRDKITTL.
QYC65019.1    LDYGQCPNNMMGTFSIRTVGTEK.SPHSITLRVYMRIKHVRAWIPRPLRNQPYLFKTNPNYKGNDIKCTSTSRDKITTL.
QWF08742.1    LDYGQCPNNMMGTFSIRTVGTEK.SPHSITLRVYVRIKHVRAWIPRPLRNQPYLFKTNPNYKGNDIKCTSTSRDKITTL.
QWF08728.1    LDYGQCPNNMMGTFSIRTVGTEK.SPHSITLRVYMRIKHVRAWIPRPLRNQPYLFKTNPNYKGNDIKCTSTSRDKITTL.
QWF08726.1    LDYGQCPNNMMGTFSIRTVGTEK.SPHSITLRVYMRIKHVRAWIPRPLRNQPYLFKTNPNYKGNDIKCTSTSRDKITTL.
QYC65054.1    LDYGQCPNNMMGTFSIRTVGTEK.SPHSITLRVYMRIKHVRAWIPRPLRNQPYLFKTNPNYKGNDIKCTSTSRDKITTL.
QWF08746.1    LDYGQCPNNMMGTFSIRTVGTEK.SPHSITLRVYMRIKHVRAWIPRPLRNQPYLFKTNPNYKGNDIKCTSTSRDKITTL.
QYC65033.1    LDYGQCPNNMMGTFSIRTVGTEK.SPHSITLRVYMRIKHVRAWIPRPLRNQPYLFKTNPNYKGNDIKCTSTSRDKITTL.
QWY15952.1    LDYGQCPNNMMGTFSIRTVGTEK.SPHSITLRVYMRIKHVRAWIPRPLRNQPYLFKTNPNYKGNDIKCTSTSRDKITTL.
QYC65042.1    LDYGQCPNNMMGTFSIRTVGTEK.SPHSITLRVYMRIKHVRAWIPRPLRNQPYLFKTNPNYKGNDIKCTSNSRDKITTL.
QYC65017.1    LDYGQCPNNMMGTFSIRTVGTEK.SPHSITLRVYMRIKHVRAWIPRPLRNQPYLFKTNPNYKGNDIKCTSTSRDKITTL.
QOP74918.1    LDYGQCPNNMMGTFSIRTVGTEK.SPHSITLRVYMRIKHVRAWIPRPLRNQPYLFKTNPNYKGNDIKCTSSSRDKITT..
QWF08731.1    LDYGQCPNNMMGTFSIRTVGTEK.SPHSITLRVYMRIKHVRAWIPRPLRNQPYLFKTNPNYKGNDIKCTSTSRDKITTL.
BBH72123.1    LDYGQCPNNMMGTFSIRTVGTEK.SPHSITLRVYMRIKHVRAWIPRPLRNQPYLFKTNPNYKGNDIKCISTSRVKITTL.
BBH72114.1    LDYGQCPNNMMGTFSIRTVGTEK.SPHSITLRVYMRIKHVRAWIPRPLRNQPYLFKTNPNYKGNDIKCISTSRDKITTL.
BBH72092.1    LDYGQCPNNMMGTFSIRTVGTEK.SPHSITLRVYMRIKHVRAWIPRPLRNQPYLFKTNPNYKGNDIKCTSTSRDKITTL.
BBH72095.1    LDYGQCPNNMMGTFSIRTVGTEK.SPHSITLRVYMRIKHVRAWIPRPLRNQPYLFKTNPNYKGNDIKCTSTSRVKITTL.
BBH72186.1    LDYGQCPNNMMGTFSIRTVGTEK.SPHSITLRVYMRIKHVRAWIPRPLRNQPYLFKTNPNYKGNDIKCTSTSRDKITTL.
QWY15975.1    LDYGQCPNNMMGTFSIRTVGTEK.SPHSITLRIYMRIKHVRAWIPRPLRNQPYLFKTNPNYKGNDIKCTSTSRDKITTL.
QWY15974.1    LDYGQCPNNMMGTFSIRTVGTEK.SPHSITLRIYMRIKHVRAWIPRPLRNQPYLFKTNPNYKGNDIKCTSTSRDKITTL.
QWY15968.1    LDYGQCPNNMMGTFSIRTVGTEK.SPHSITLRIYMRIKHVRAWIPRPLRNQPYLFKTNPNYKGNDIKCTSTSRDKITTL.
QWY15960.1    LDYGQCPNNMMGTFSIRTVGTEK.SPHSITLRIYMRIKHVRAWIPRPLRNQPYLFKTNPNYKGNDIKCTSTSRDKITTL.
QWY15959.1    LDYGQCPNNMMGTFSIRTVGTEK.SPHSITLRIYMRIKHVRAWIPRPLRNQPYLFKTNPNYKGNDIKCTSTSRDKITTL.
QWY15953.1    LDYGQCPNNMMGTFSIRTVGTEK.SPHSITLRIYMRIKHVRAWIPRPLRNQPYLFKTNPNYKGNDIKCTSTSRDKITTL.
QWY15951.1    LDYGQCPNNMMGTFSIRTVGTEK.SPHSITLRIYMRIKHVRAWIPRPLRNQPYLFKTNPNYKGNDIKCTSTSRDKITTL.
QWY15950.1    LDYGQCPNNMMGTFSIRTVGTEK.SPHSITLRIYMRIKHVRAWIPRPLRNQPYLFKTNPNYKGNDIKCTSTSRDKITTL.
QWY15949.1    LDYGQCPNNMMGTFSIRTVGTEK.SPHSITLRIYMRIKHVRAWIPRPLRNQPYLFKTNPNYKGNDIKCTSTSRDKITTL.
QWY15944.1    LDYGQCPNNMMGTFSIRTVGTEK.SPHSITLRIYMRIKHVRAWIPRPLRNQPYLFKTNPNYKGNDIKCTSTSRDKITTL.
QWY15943.1    LDYGQCPNNMMGTFSIRTVGTEK.SPHSITLRIYMRIKHVRAWIPRPLRNQPYLFKTNPNYKGNDIKCTSTSRDKITTL.
QWY15942.1    LDYGQCPNNMMGTFSIRTVGTEK.SPHSITLRIYMRIKHVRAWIPRPLRNQPYLFKTNPNYKGNDIKCTSTSRDKITTL.
QWY15939.1    LDYGQCPNNMMGTFSIRTVGTEK.SPHSITLRIYMRIKHVRAWIPRPLRNQPYLFKTNPNYKGNDIKCTSTSRDKITTL.
QWY15938.1    LDYGQCPNNMMGTFSIRTVGTEK.SPHSITLRIYMRIKHVRAWIPRPLRNQPYLFKTNPNYKGNDIKCTSTSRDKITTL.
QWY15937.1    LDYGQCPNNMMGTFSIRTVGTEK.SPHSITLRIYMRIKHVRAWIPRPLRNQPYLFKTNPNYKGNDIKCTSTSRDKITTL.
QWY15936.1    LDYGQCPNNMMGTFSIRTVGTEK.SPHSITLRIYMRIKHVRAWIPRPLRNQPYLFKTNPNYKGNDIKCTSTSRDKITTL.
QWY15934.1    LDYGQCPNNMMGTFSIRTVGTEK.SPHSITLRIYMRIKHVRAWIPRPLRNQPYLFKTNPNYKGNDIKCTSTSRDKITTL.
QWY15896.1    LDYGQCPNNMMGTFSIRTVGTEK.SPHSITLRIYMRIKHVRAWIPRPLRNQPYLFKTNPNYKGNDIKCTSTSRDKITTL.
BCU41098.1    LDYGQCPNNMMGTFSIRTVGTEK.SPHSITLRIYMRIKHVRAWIPRPLRNQPYLFKTNPNYKGNDIKCTSTSRDKITTL.
BCR43090.1    LDYGQCPNNMMGTFSIRTVGTEK.SPHSITLRIYMRIKHVRAWIPRPLRNQPYLFKTNPNYKGNDIKCTSTSRDKITTL.
QYC65129.1    LDYGQCPNNMMGTFSIRTVGTEK.SPHSITLRIYMRIKHVRAWIPRPLRNQPYLFKTNPNYKGNDIKCTSTSRDKITTL.
QWF08961.1    LDYGQCPNNMMGTFSIRTVGTEK.SPHSITLRIYMRIKHVRAWIPRPLRNQPYLFKTNPNYKGNDIKCTSTSRDKITTL.
QIL87527.1    LDYGQCPNNMMGTFSIRTVGTEK.SPHSITLRIYMRIKHVRAWIPRPLRNQPYLFKTNPNYKGNDIKCTSTSRDKITTL.
QWY15955.1    LDYGQCPNNMMGTFSIRTVGTEK.SPHSITLRIYMRIKHVRAWIPRPLRNQPYLFKTNPNYKGNDIKCTSTSRDKITTL.
QWY15954.1    LDYGQCPNNMMGTFSIRTVGTEK.SPHSITLRIYMRIKHVRAWIPRPLRNQPYLFKTNPNYKGNDIKCTSTSRDKITTL.
QWY15945.1    LDYGQCPNNMMGTFSIRTVGTEK.SPHSITLRIYMRIKHVRAWIPRPLRNQPYLFKTNPNYKGNDIKCTSTSRDKITTL.
QWY15935.1    LDYGQCPNNMMGTFSIRTVGTEK.SPHSITLRIYMRIKHVRAWIPRPLRNQPYLFKTNPNYKGNDIKCTSTSRDKITTL.
BCB92163.1    LDYGQCPNNMMGTFSIRTVGTEK.SPHSITLRIYMRIKHVRAWIPRPLRNQPYLFKTNPNYKGNDIKCTSTSRDKITTL.
BCB92158.1    LDYGQCPNNMMGTFSIRTVGTEK.SPHSITLRIYMRIKHVRAWIPRPLRNQPYLFKTNPNYKGNDIKCTSTSRDKITTL.
BCB92154.1    LDYGQCPNNMMGTFSIRTVGTEK.SPHSITLRIYMRIKHVRAWIPRPLRNQPYLFKTNPNYKGNDIKCTSTSRDKITTL.
BCB92152.1    LDYGQCPNNMMGTFSIRTVGTEK.SPHSITLRIYMRIKHVRAWIPRPLRNQPYLFKTNPNYKGNDIKCTSTSRDKITTL.
BCB92162.1    LDYGQCPNNMMGTFSIRTVGTEK.SPHSITLRIYMRIKHVRAWIPRPLRNQPYLFKTNPNYKGDDIKCTSTSRDKITTL.
BCB92161.1    LDYGQCPNNMMGTFSIRTVGTEK.SPHSITLRIYMRIKHVRAWIPRPLRNQPYLFKTNPNYKGDDIKCTSTSRDKITTL.
BCB92160.1    LDYGQCPNNMMGTFSIRTVGTEK.SPHSITLRIYMRIKHVRAWIPRPLRNQPYLFKTNPNYKGDDIKCTSTSRDKITTL.
BBH72129.1    LDYGQCPNNMMGTFSIRTVGTEK.SPHSITLRIYMRIKHVRAWIPRPLRNQPYLFKTNPNYKGDDIKCTSTSRDKITTL.
QIL87533.1    LDYGQCPNNMMGTFSIRTVGTEK.SPHSITLRIYMRIKHVRAWIPRPLRNQPYLFKTNPNYKGDDIKCTSTSRDKITTL.
BBH72150.1    LDYGQCQNNMMGTFSIRTVGTEK.SPHSITLRIYMRIKHVRAWNPRPLRNQPYLFKTNPNYKGNDIKCTSTSRDKITTL.
BBH72137.1    LDYGQCPNNMMGTFSIRTVGTEK.SPHSITMRIYMRIKHVRAWIPRPLRNQPYLFKTNPNYKGDDIKCTSTSRDKITTL.
BBH72145.1    LDYGQCPNNMMGTFSIRTVGTEK.SPHSITLRIYMRIKHVRAWIPRPLRNQPYLFKTNPNYKGNDIKCTSTSRDKITTL.
BCB92157.1    LDYGQCPNNMMGTFSIRTVGTEK.SPHSITLRIYMRIKHVRAWIPRPLRNQPYLFKTNPNYKGNDIKCTSTSRDKITTL.
QYC65124.1    LDYGQCPNNMMGTFSIRTVGTEK.SPHSITLRIYMRIKHVRAWIPRPLRNQPYLFKTNPNYKGNDIKCTSTSRDKITTL.
QWY15920.1    LDYGQCPNNMMGTFSIRTVGTEK.SPHSITLRIYMRIKHVRAWIPRPLRNQPYLFKTNPNYKGNDIKCTSTSRDKITTL.
QWY15973.1    LDYGQCPNNMMGTFSIRTVGTEK.SPHSITLRIYMRIKHVRAWIPRPLRNQPYLFKTNPNYKGNDIKCTSTSRDKITTL.
BCU41116.1    LDYGQCPNNMMGTFSIRTVGTEK.SPHSITLRIYMRIKHVRAWIPRPLRNQPYLFKTNPNYKGNDIKCTSTSRDKITTL.
QWF08960.1    LDYGQCPNNMMGTFSIRTVGTEK.SPHSITLRIYMRIKHVRAWIPRPLRNQPYLFKTNPNYKGNDIKCTSTSRDKITTL.
QWF08958.1    LDYGQCPNNMMGTFSIRTVGTEK.SPHSITLRIYMRIKHVRAWIPRPLRNQPYLFKTNPNYKGNDIKCTSTSRDKITTL.
QWF08919.1    LDYGQCPNNMMGTFSIRTVGTEK.SPHSITLRIYMRIKHVRAWIPRPLRNQPYLFKTNPNYKGNDIKCTSTSRDKITTL.
QWY15825.1    LDYGQCPNNMMGTFSIRTVGTEK.SPHSITLRIYMRIKHVRAWIPRPLRNQPYLFKTNPNYKGNDIKCTSTSRDKITTL.
QWF08953.1    LDYGQCPNNMMGTFSIRTVGTEK.SPHSITLRIYMRIKHVRAWIPRPLRNQPYLFKTNPNYKGNDIKCTSTSRDKITTL.
QWF08948.1    LDYGQCPNNMMGTFSIRTVGTEK.SPHSITLRIYMRIKHVRAWIPRPLRNQPYLFKTNPNYKGNDIKCTSTSRDKITTL.
QWY15884.1    LDYGQCPNNMMGTFSIRTVGTEK.SPHSITLRIYMRIKHVRAWIPRPLRNQPYLFKTNPNYKGNDIKCTSTSRDKITTL.
QWF08946.1    LDYGQCPNNMMGTFSIRTVGTEK.SPHSITLRIYMRIKHVRAWIPRPLRNQPYLFKTNPNYKGNDIKCTSTSRDKITTL.
QWF08941.1    LDYGQCPNNMMGTFSIRTVGTEK.SPHSITLRIYMRIKHVRAWIPRPLRNQPYLFKTNPNYKGNDIKCTSTSRDKITTL.
QWF08914.1    LDYGQCPNNMMGTFSIRTVGTEK.SPHSITLRIYMRIKHVRAWIPRPLRNQPYLFKTNPNYKGNDIKCTSTSRDKITTL.
BCU41097.1    LDYGQCPNNMMGTFSIRTVGTEK.SPHSITLRIYMRIKHVRAWIPRPLRNQPYLFKTNPNYKGNDIKCTSTSRDKITTL.
QWY15918.1    LDYGQCPNNMMGTFSIRTVGTEK.SPHSITLRIYMRIKHVRAWIPRPLRNQPYLFKTNPNYKGNDIKCTSTSRDKITTL.
BCR43086.1    LDYGQCPNNMMGTFSIRTVGTEK.SPHSITLRIYMRIKHVRAWIPRPLRNQPYLFKTNPNYKGNDIKCTSTSRDKITTL.
QWF08935.1    LDYGQCPNNMMGTFSIRTVGTEK.SPHSITLRIYMRIKHVRAWIPRPLRNQPYLFKTNPNYKGNDIKCTSTSRDKITTL.
QWY15912.1    LDYGQCPNNMMGTFSIRTVGTEK.SPHSITLRIYMRIKHVRAWIPRPLRNQPYLFKTNPNYKGNDIKCTSTSRDKITTL.
QWY15911.1    LDYGQCPNNMMGTFSIRTVGTEK.SPHSITLRIYMRIKHVRAWIPRPLRNQPYLFKTNPNYKGNDIKCTSTSRDKITTL.
QWY15887.1    LDYGQCPNNMMGTFSIRTVGTEK.SPHSITLRIYMRIKHVRAWIPRPLRNQPYLFKTNPNYKGNDIKCTSTSRDKITTL.
```

```
QWY15882.1   LDYGQCPNNMMGTFSIRTVGTEK.SPHSITLRIYMRIKHVRAWIPRPLRNQPYLFKTNPNYKGNDIKCTSTSRDKITTL.
BCG67018.1   LDYGQCPNNMMGTFSIRTVGTEK.SPHSITLRIYMRIKHVRAWIPRPLRNQPYLFKTNPNYKGNDIKCTSTSRDKITTL.
QWY15881.1   LDYGQCPNNMMGTFSIRTVGTEK.SPHSITLRIYMRIKHVRAWIPRPLRNQPYLFKTNPNYKGNDIKCTSTSRDKITTL.
QWY15875.1   LDYGQCPNNMMGTFSIRTVGTEK.SPHSITLRIYMRIKHVRAWIPRPLRNQPYLFKTNPNYKGNDIKCTSTSRDKITTL.
BCG67016.1   LDYGQCPNNMMGTFSIRTVGTEK.SPHSITLRIYMRIKHVRAWIPRPLRNQPYLFKTNPNYKGNDIKCTSTSRDKITTL.
QWY15874.1   LDYGQCPNNMMGTFSIRTVGTEK.SPHSITLRIYMRIKHVRAWIPRPLRNQPYLFKTNPNYKGNDIKCTSTSRDKITTL.
QWY15853.1   LDYGQCPNNMMGTFSIRTVGTEK.SPHSITLRIYMRIKHVRAWIPRPLRNQPYLFKTNPNYKGNDIKCTSTSRDKITTL.
BCG67014.1   LDYGQCPNNMMGTFSIRTVGTEK.SPHSITLRIYMRIKHVRAWIPRPLRNQPYLFKTNPNYKGNDIKCTSTSRDKITTL.
BCG67009.1   LDYGQCPNNMMGTFSIRTVGTEK.SPHSITLRIYMRIKHVRAWIPRPLRNQPYLFKTNPNYKGNDIKCTSTSRDKITTL.
QWF08933.1   LDYGQCPNNMMGTFSIRTVGTEK.SPHSITLRIYMRIKHVRAWIPRPLRNQPYLFKTNPNYKGNDIKCTSTSRDKITTL.
BCG66992.1   LDYGQCPNNMMGTFSIRTVGTEK.SPHSITLRIYMRIKHVRAWIPRPLRNQPYLFKTNPNYKGNDIKCTSTSRDKITTL.
BCG66991.1   LDYGQCPNNMMGTFSIRTVGTEK.SPHSITLRIYMRIKHVRAWIPRPLRNQPYLFKTNPNYKGNDIKCTSTSRDKITTL.
QWF08898.1   LDYGQCPNNMMGTFSIRTVGTEK.SPHSITLRIYMRIKHVRAWIPRPLRNQPYLFKTNPNYKGNDIKCTSTSRDKITTL.
QWF08930.1   LDYGQCPNNMMGTFSIRTVGTEK.SPHSITLRIYMRIKHVRAWIPRPLRNQPYLFKTNPNYKGNDIKCTSTSRDKITTL.
QWF08925.1   LDYGQCPNNMMGTFSIRTVGTEK.SPHSITLRIYMRIKHVRAWIPRPLRNQPYLFKTNPNYKGNDIKCTSTSRDKITTL.
QWF08913.1   LDYGQCPNNMMGTFSIRTVGTEK.SPHSITLRIYMRIKHVRAWIPRPLRNQPYLFKTNPNYKGNDIKCTSTSRDKITTL.
QWF08911.1   LDYGQCPNNMMGTFSIRTVGTEK.SPHSITLRIYMRIKHVRAWIPRPLRNQPYLFKTNPNYKGNDIKCTSTSRDKITTL.
BBH72117.1   LDYGQCPNNMMGTFSIRTVGTEK.SPHSITLRIYMRIKHVRAWIPRPLRNQPYLFKTNPNYKGNDIKCTSTSRDKITTL.
BBH72116.1   LDYGQCPNNMMGTFSIRTVGTEK.SPHSITLRIYMRIKHVRAWIPRPLRNQPYLFKTNPNYKGNDIKCTSTSRDKITTL.
BBH72113.1   LDYGQCPNNMMGTFSIRTVGTEK.SPHSITLRIYMRIKHVRAWIPRPLRNQPYLFKTNPNYKGNDIKCTSTSRDKITTL.
BBH72112.1   LDYGQCPNNMMGTFSIRTVGTEK.SPHSITLRIYMRIKHVRAWIPRPLRNQPYLFKTNPNYKGNDIKCTSTSRDKITTL.
BBH72108.1   LDYGQCPNNMMGTFSIRTVGTEK.SPHSITLRIYMRIKHVRAWIPRPLRNQPYLFKTNPNYKGNDIKCTSTSRDKITTL.
BBH72106.1   LDYGQCPNNMMGTFSIRTVGTEK.SPHSITLRIYMRIKHVRAWIPRPLRNQPYLFKTNPNYKGNDIKCTSTSRDKITTL.
BBH72104.1   LDYGQCPNNMMGTFSIRTVGTEK.SPHSITLRIYMRIKHVRAWIPRPLRNQPYLFKTNPNYKGNDIKCTSTSRDKITTL.
BBH72211.1   LDYGQCPNNMMGTFSIRTVGTEK.SPHSITLRIYMRIKHVRAWIPRPLRNQPYLFKTNPNYKGNDIKCTSTSRDKITTL.
BBH72210.1   LDYGQCPNNMMGTFSIRTVGTEK.SPHSITLRIYMRIKHVRAWIPRPLRNQPYLFKTNPNYKGNDIKCTSTSRDKITTL.
BBH72209.1   LDYGQCPNNMMGTFSIRTVGTEK.SPHSITLRIYMRIKHVRAWIPRPLRNQPYLFKTNPNYKGNDIKCTSTSRDKITTL.
BBH72208.1   LDYGQCPNNMMGTFSIRTVGTEK.SPHSITLRIYMRIKHVRAWIPRPLRNQPYLFKTNPNYKGNDIKCTSTSRDKITTL.
BBH72207.1   LDYGQCPNNMMGTFSIRTVGTEK.SPHSITLRIYMRIKHVRAWIPRPLRNQPYLFKTNPNYKGNDIKCTSTSRDKITTL.
BBH72205.1   LDYGQCPNNMMGTFSIRTVGTEK.SPHSITLRIYMRIKHVRAWIPRPLRNQPYLFKTNPNYKGNDIKCTSTSRDKITTL.
BBH72201.1   LDYGQCPNNMMGTFSIRTVGTEK.SPHSITLRIYMRIKHVRAWIPRPLRNQPYLFKTNPNYKGNDIKCTSTSRDKITTL.
BBH72193.1   LDYGQCPNNMMGTFSIRTVGTEK.SPHSITLRIYMRIKHVRAWIPRPLRNQPYLFKTNPNYKGNDIKCTSTSRDKITTL.
BBH72192.1   LDYGQCPNNMMGTFSIRTVGTEK.SPHSITLRIYMRIKHVRAWIPRPLRNQPYLFKTNPNYKGNDIKCTSTSRDKITTL.
BBH72190.1   LDYGQCPNNMMGTFSIRTVGTEK.SPHSITLRIYMRIKHVRAWIPRPLRNQPYLFKTNPNYKGNDIKCTSTSRDKITTL.
BBH72189.1   LDYGQCPNNMMGTFSIRTVGTEK.SPHSITLRIYMRIKHVRAWIPRPLRNQPYLFKTNPNYKGNDIKCTSTSRDKITTL.
BBH72188.1   LDYGQCPNNMMGTFSIRTVGTEK.SPHSITLRIYMRIKHVRAWIPRPLRNQPYLFKTNPNYKGNDIKCTSTSRDKITTL.
BBH72187.1   LDYGQCPNNMMGTFSIRTVGTEK.SPHSITLRIYMRIKHVRAWIPRPLRNQPYLFKTNPNYKGNDIKCTSTSRDKITTL.
BBH72185.1   LDYGQCPNNMMGTFSIRTVGTEK.SPHSITLRIYMRIKHVRAWIPRPLRNQPYLFKTNPNYKGNDIKCTSTSRDKITTL.
BBH72182.1   LDYGQCPNNMMGTFSIRTVGTEK.SPHSITLRIYMRIKHVRAWIPRPLRNQPYLFKTNPNYKGNDIKCTSTSRDKITTL.
BBH72181.1   LDYGQCPNNMMGTFSIRTVGTEK.SPHSITLRIYMRIKHVRAWIPRPLRNQPYLFKTNPNYKGNDIKCTSTSRDKITTL.
BBH72179.1   LDYGQCPNNMMGTFSIRTVGTEK.SPHSITLRIYMRIKHVRAWIPRPLRNQPYLFKTNPNYKGNDIKCTSTSRDKITTL.
BBH72178.1   LDYGQCPNNMMGTFSIRTVGTEK.SPHSITLRIYMRIKHVRAWIPRPLRNQPYLFKTNPNYKGNDIKCTSTSRDKITTL.
BBH72177.1   LDYGQCPNNMMGTFSIRTVGTEK.SPHSITLRIYMRIKHVRAWIPRPLRNQPYLFKTNPNYKGNDIKCTSTSRDKITTL.
BBH72175.1   LDYGQCPNNMMGTFSIRTVGTEK.SPHSITLRIYMRIKHVRAWIPRPLRNQPYLFKTNPNYKGNDIKCTSTSRDKITTL.
BBH72174.1   LDYGQCPNNMMGTFSIRTVGTEK.SPHSITLRIYMRIKHVRAWIPRPLRNQPYLFKTNPNYKGNDIKCTSTSRDKITTL.
BBH72172.1   LDYGQCPNNMMGTFSIRTVGTEK.SPHSITLRIYMRIKHVRAWIPRPLRNQPYLFKTNPNYKGNDIKCTSTSRDKITTL.
BBH72170.1   LDYGQCPNNMMGTFSIRTVGTEK.SPHSITLRIYMRIKHVRAWIPRPLRNQPYLFKTNPNYKGNDIKCTSTSRDKITTL.
BBH72169.1   LDYGQCPNNMMGTFSIRTVGTEK.SPHSITLRIYMRIKHVRAWIPRPLRNQPYLFKTNPNYKGNDIKCTSTSRDKITTL.
BBH72168.1   LDYGQCPNNMMGTFSIRTVGTEK.SPHSITLRIYMRIKHVRAWIPRPLRNQPYLFKTNPNYKGNDIKCTSTSRDKITTL.
BBH72158.1   LDYGQCPNNMMGTFSIRTVGTEK.SPHSITLRIYMRIKHVRAWIPRPLRNQPYLFKTNPNYKGNDIKCTSTSRDKITTL.
BBH72155.1   LDYGQCPNNMMGTFSIRTVGTEK.SPHSITLRIYMRIKHVRAWIPRPLRNQPYLFKTNPNYKGNDIKCTSTSRDKITTL.
BBH72154.1   LDYGQCPNNMMGTFSIRTVGTEK.SPHSITLRIYMRIKHVRAWIPRPLRNQPYLFKTNPNYKGNDIKCTSTSRDKITTL.
BBH72151.1   LDYGQCPNNMMGTFSIRTVGTEK.SPHSITLRIYMRIKHVRAWIPRPLRNQPYLFKTNPNYKGNDIKCTSTSRDKITTL.
BBH72148.1   LDYGQCPNNMMGTFSIRTVGTEK.SPHSITLRIYMRIKHVRAWIPRPLRNQPYLFKTNPNYKGNDIKCTSTSRDKITTL.
BBH72144.1   LDYGQCPNNMMGTFSIRTVGTEK.SPHSITLRIYMRIKHVRAWIPRPLRNQPYLFKTNPNYKGNDIKCTSTSRDKITTL.
BBH72142.1   LDYGQCPNNMMGTFSIRTVGTEK.SPHSITLRIYMRIKHVRAWIPRPLRNQPYLFKTNPNYKGNDIKCTSTSRDKITTL.
BBH72141.1   LDYGQCPNNMMGTFSIRTVGTEK.SPHSITLRIYMRIKHVRAWIPRPLRNQPYLFKTNPNYKGNDIKCTSTSRDKITTL.
BBH72140.1   LDYGQCPNNMMGTFSIRTVGTEK.SPHSITLRIYMRIKHVRAWIPRPLRNQPYLFKTNPNYKGNDIKCTSTSRDKITTL.
BBH72132.1   LDYGQCPNNMMGTFSIRTVGTEK.SPHSITLRIYMRIKHVRAWIPRPLRNQPYLFKTNPNYKGNDIKCTSTSRDKITTL.
BBH72131.1   LDYGQCPNNMMGTFSIRTVGTEK.SPHSITLRIYMRIKHVRAWIPRPLRNQPYLFKTNPNYKGNDIKCTSTSRDKITTL.
BBH72130.1   LDYGQCPNNMMGTFSIRTVGTEK.SPHSITLRIYMRIKHVRAWIPRPLRNQPYLFKTNPNYKGNDIKCTSTSRDKITTL.
BBH72128.1   LDYGQCPNNMMGTFSIRTVGTEK.SPHSITLRIYMRIKHVRAWIPRPLRNQPYLFKTNPNYKGNDIKCTSTSRDKITTL.
BDC16283.1   LDYGQCPNNMMGTFSIRTVGTEK.SPHSITLRIYMRIKHVRAWIPRPLRNQPYLFKTNPNYKGNDIKCTSTSRDKITTL.
BDC16271.1   LDYGQCPNNMMGTFSIRTVGTEK.SPHSITLRIYMRIKHVRAWIPRPLRNQPYLFKTNPNYKGNDIKCTSTSRDKITTL.
BDC16270.1   LDYGQCPNNMMGTFSIRTVGTEK.SPHSITLRIYMRIKHVRAWIPRPLRNQPYLFKTNPNYKGNDIKCTSTSRDKITTL.
BDC16258.1   LDYGQCPNNMMGTFSIRTVGTEK.SPHSITLRIYMRIKHVRAWIPRPLRNQPYLFKTNPNYKGNDIKCTSTSRDKITTL.
QIL87526.1   LDYGQCPNNMMGTFSIRTVGTEK.SPHSITLRIYMRIKHVRAWIPRPLRNQPYLFKTNPNYKGNDIKCTSTSRDKITTL.
BBH72180.1   LDYGQCPNNMMGTFSIRTVGTEK.SPHSITLRIYMRIKHVRAWIPRPLRNQPYLFKTNPNYKGNDIKCTSTSRDKITTL.
BBH72109.1   LDYGQCPNNMMGTFSIRTVGTEK.SPQSITLRIYMRIKHVRAWIPRPLRNQPYLFETNPNYKGNDIKCTSTSRDKITTL.
BBH72115.1   LDYGQCPNNMMGTFSIRTVGTEK.SPHSITLRIYMRIKHVRAWIPRPLRNQPYLFKTNPNYKGNDIKCTSTSIDKITTL.
BBH72152.1   LDYGQCPNNMMGTFSIRTVGTEK.SPHPITLRIYMRIKHVRAWIPRPLRNQPYLFKTNPNYKGNDIKCTSTSRDKITTL.
BBH72200.1   LDYGQCPNNMMGTFSIRTVGTEK.SPHSITLRIYMRIKHVRAWIPRPLRNQPYLFKANPNYKGNDIKCTSTSRDKITTL.
QWY15844.1   LDYGQCPNNMMGTFSIRTVGTEK.SPHSITLRIYMRIKHVRAWIPRPLRNQPYLFKTNPNYKGNDIKCTSTSRDKITTL.
BBH72146.1   LDYGQCPNNMMGTFSIRTVGTEK.SPHSITLRIYMRIKHVRAWIPRPLRNQPYLFKTNPNYKGNDIKCTSTSRDKISTL.
BBH72173.1   LDYGQCPNNMMGTFSIRTVGTEK.SPHSITLRIYMRIKHVRAWIPRPLRNQPYLFKTNPNYKGNDIKCTSTSRDKITTL.
QWY15947.1   LDYGQCPNNMMGTFSIRTVGTEK.SPHSITLRIYMRIKHVRAWIPRPLKNQPYLFKTNPNYKGNDIKCTSTSRDKITPL.
BDC16281.1   LDYGQCPNNMMGTFSIRTVGTEK.SPHSITLRIYMRIKHVRAWIPRPLKNQPYLFKTNPNYKGNDIKCTSTSRDKITTL.
BBH72167.1   LDYGQCPNNMMGTFSIRTVGTEK.SPHSITLRIYMRIKHVRAWIPRPLRNQPYLFKTNPNYKGNDIKCTSTSRDKITTL.
BBH72160.1   LDYGQCPNNMMGTFSIRTVGTEK.SPHSITLRIYMRIKHVRAWIPRPLRNQPYLFKTNPNYKGNDIKCTSTSRDKITTL.
BCU41096.1   LDYGQCPNNMMGTFSIRTVGTEK.SPHSITLRIYMRIKHVRAWIPRPLRNQPYLFKTNPNYKGNDIKCTSTSRDKITTL.
BCR43102.1   LDYGQCPNNMMGTFSIRTVGTEK.SPHSITLRIYMRIKHVRAWIPRPLRNQPYLFKTNPNYKGNDIKCTSTSRDKITTL.
QWF08881.1   LDYGQCPNNMMGTFSIRTVGTEK.SPHSITLRIYMRIKHVRAWIPRPLRNQPYLFKTNPNYKGNDIKCTSTSRDKITTL.
QWF08855.1   LDYGQCPNNMMGTFSIRTVGTEK.SPHSITLRIYMRIKHVRAWIPRPLRNQPYLFKTNPNYKGNDIKCTSTSRDKITTL.
BBH72195.1   LDYGQCPNNMMGTFSIRTVGTEK.SPHSITLRIYMRIKHVRAWIPRPLRNQPYLFKTNPNYKGNDIKCTSTSRDKITTL.
BBH72118.1   LDYGQCPNNMMGTFSIRTVGTEK.SPHSITLRIYMRIKHVRAWIPRPLRNQPYLFKTNPNYKGNDIKCISTSRVKITT..
BBH72126.1   LDYGQCPNNMMGTFSIRTVGTEK.SPHSITLRIYMRIKHVRAWIPRPLRNQPYLFKTNPNYKGNDIKCISTSRVKITTL.
BBH72127.1   LDYGQCPNNMMGTFSIRTVGTEK.SPHSITLRIYMRIKHVRAWIPRPLRNQPYLFKTNPNYKGNDIKCTRTSRDKITTL.
BBH72206.1   LDYGQCPNNMMGTFSIRTVGTEK.SPHSITLRIYMRIKHVRAWIPRPLRNQPYLFKTNPNYKGNDIKCTRTSRDKITTL.
BBH72196.1   LDYGQCPNNMMGTFSIRTVGTEK.SPHSITLRIYMRIKHVRAWIPRPLRNQPYLFKTNPNYKGNDIKCTVTNRDKITTL.
BBH72171.1   LDYGQCPNNMMGTFSIRTVGTEK.SPHSITLRIYMRIKHVRAWIPRPLRNQPYLFKTNPNYKGNDIKCTSTSRDKITTL.
BBH72191.1   LDYGQCPNNMMGTFSIRTGGNEK.SPHSITLRIYMRIKHVRAWIPRLLRNQPYLFKTNPNYKGNDIKCTSTSRDKITTL.
BBH72133.1   LDYGQCPNNMMGTFSIRTVGNEK.SPHSITLRIYMRIKHVRAWIPRPLRNQPYLFKTNPNYKGNDIKCTSTSRDKITTL.
BBH72139.1   LDYGQCPNNMMGNFSIRTVGNEK.SPHSITLRIYMRIKHVRAWIPRPLRNQPYLFKTNPNYKGNDIKCTSTSRDKITTL.
BBH72166.1   LDYGQCPNNMMGTFSIRSVGIEK.SSHSITLRIYLRIKHVRAWIPRPLRNQPYLFKTNPNYKGNDIKCTSTSRDKITTL.
BBH72161.1   LDYGQCPNNMMGTFSIRSVGTEK.SPHSITVRIYLRIKHVRAWIPRPLRNQPYLFKTNPNYKGNDIKCNSTSRDKITTL.
BBH72165.1   LDYGQCPNNMMGTFSIRTVGTEK.SPHSITLRIYMRIKHVRAWIPRPLRNQPYLFKTNPNYKGNDIKCTSTSRDKITTL.
BBH72164.1   LDYGQCPNNMMGTFSIRTVGIEK.SPHSITLRIYMRIKHVRAWIPRLLRNQPYLFKTNPNYKGNDIKCTSTSRDKITTL.
BBH72159.1   LDYGQCPNNMMGTFSIRTVGTEK.SPHSITVRIYLRIKHVRAWIPRPLRNQPYLFKTNPNYKGNDIKCTSTSRDKITTL.
BBH72157.1   LDYGQCPNNMMGTFSIRTVGTEK.SSHSITVRIYLRIKLVRAWIPRPLRNQPYLFKTNPNYKGNDIKCISTSRDKITTL.
BBH72162.1   LDYGQCPNNMMGTFSIRTVGIEK.SPHSITLRIYMRIKHVRAWIPRPLRNQPYLFKTNPNYKGNDIKCTSTSRDKITTL.
BBH72153.1   LDYGQCPNNMMGTLSMRSVGIEK.SPHSITLRIYLRIKHVRAWIPRPLRNQPYLFKTNPNYKGNDIKCNSTSRDKITTL.
BBH72156.1   LDYGQCPNNMMGTFSIRTVGTEK.SPHSITLRIYMRIKHVRAWIPRPLRNQPYLFKTNPNYKGNDIKCTSTSRDKITTL.
BBH72120.1   LDYGQCPNNMMGTFSIRTVGTEK.SPHSITLRIYMRIKHVRAWIPRPLRNQPYLFKTNPNYKGNDIKCTSTSRDKITTL.
BBH72198.1   LDYGQCPNNMMGTFSIRTVGTEK.SPHSITLRIYMRIKHVRAWIPRPLRNQPYLFKTNPNYKGNDIKCTSTSRDKITTL.
BBH72197.1   LDYGQCPNNMMGTFSIRTVGTEK.SPHSITLRIYMRIKHVRAWIPRPLRNQPYLFKTNPNYKGNDIKCTSTSRDKITTL.
QWY15941.1   LDYGQCPNNMMGTFSIRTVGTEK.SPHSITLRIYMRIKHVRAWIPRPLRNQPYLFKTNPNYKGNDIKCTSASRDKITTL.
BBH72121.1   LDYGQCPNNMMGTFSIRTVGTEK.SPHSITLRIYMRIKHVRAWIPRPLRNQPYLFKTNPNYKGNDINCTSTSRDKITTY.
BBH72135.1   LDYGQCPNNMMGTFSIRTVGTEK.SPHSITLRIYMRIKHVRAWIPRPLRNQPYLFKTNPNYKGNDINCTSTSRDKITTD.
QWY15967.1   LDYGQCPNNMMGTFSIRTVGTEK.SPHSITLRIYMRIKHVRAWIPRPLRNQPYLFKTNPNYKGNDIKCTSTSRDKITTL.
QWY15964.1   LDYGQCPNNMMGTFSIRTVGTEK.SPHSITLRIYMRIKHVRAWIPRPLRNQPYLFKTNPNYKGNDIKCTSTSRDKITTL.
QWY15969.1   LDYGQCPYNMMGTFSIRTVGTEK.SPHSITLRIYMRIKHVRAWIPRPLRNQPYLFKTNPNYKGNDIKCTSTSRDKITTL.
QWY15913.1   LDYGQCPNNMMGTFSIRTVGTEK.SPHSITLRIYMRIKHVRAWIPRPLRNQPYLFKTNPNYKGNDIKCTSTSRDKITTL.
QWY15908.1   LDYGQCPNNMMGTFSIRTVGTEK.SPHSITLRIYMRIKHVRAWIPRPLRNQPYLFKTNPNYKGNDIKCTSTSRDKITTL.
QWY15907.1   LDYGQCPNNMMGTFSIRTVGTEK.SPHSITLRIYMRIKHVRAWIPRPLRNQPYLFKTNPNYKGNDIKCTSTSRDKITTL.
QWY15890.1   LDYGQCPNNMMGTFSIRTVGTEK.SPHSITLRIYMRIKHVRAWIPRPLRNQPYLFKTNPNYKGNDIKCTSTSRDKITTL.
QWY15889.1   LDYGQCPNNMMGTFSIRTVGTEK.SPHSITLRIYMRIKHVRAWIPRPLRNQPYLFKTNPNYKGNDIKCTSTSRDKITTL.
QWY15888.1   LDYGQCPNNMMGTFSIRTVGTEK.SPHSITLRIYMRIKHVRAWIPRPLRNQPYLFKTNPNYKGNDIKCTSTSRDKITTL.
QWY15886.1   LDYGQCPNNMMGTFSIRTVGTEK.SPHSITLRIYMRIKHVRAWIPRPLRNQPYLFKTNPNYKGNDIKCTSTSRDKITTL.
QWY15885.1   LDYGQCPNNMMGTFSIRTVGTEK.SPHSITLRIYMRIKHVRAWIPRPLRNQPYLFKTNPNYKGNDIKCTSTSRDKITTL.
QWY15883.1   LDYGQCPNNMMGTFSIRTVGTEK.SPHSITLRIYMRIKHVRAWIPRPLRNQPYLFKTNPNYKGNDIKCTSTSRDKITTL.
QWY15878.1   LDYGQCPNNMMGTFSIRTVGTEK.SPHSITLRIYMRIKHVRAWIPRPLRNQPYLFKTNPNYKGNDIKCTSTSRDKITTL.
QWY15876.1   LDYGQCPNNMMGTFSIRTVGTEK.SPHSITLRIYMRIKHVRAWIPRPLRNQPYLFKTNPNYKGNDIKCTSTSRDKITTL.
BBH72176.1   LDYGQCPNNMMGTFSIRTVGTEK.SPHSITLRIYMRIKHVRAWIPRPLRNQPYLFKTNPNYKGNDIKCTSTSRDKITTL.
QIL87519.1   LDYGQCPNNMMGTFSIRTVGTEK.SPHSITLRIYMRIKHVRAWIPRPLRNQPYLFKTNPNYKGNDIKCTSTSRDKITTL.
QWY15906.1   LDYGQCPNNMMGTFSIRTVGTEK.SPHSITLRIYMRIKHVRAWIPRPLRNQPYLFKTNPNYKGNDIKCTSTSRDKITTL.
QWY15917.1   LDYGQCPNNMMGTFSIRTVGTEK.SPHSIALRIYMRIKHVRAWIPRPLRNQPYLFKTNPNYKGNDIKCTSTSRDKITTL.
QWY15846.1   LDYGQCPNNMMGTFSIRTVGTEK.SPHSIALRIYMRIKHVRAWIPRPLRNQPYLFKTNPNYKGNDIKCTSTSRDKITTL.
QWY15901.1   LDYGQCPNNMMGTFSIRTVGTEK.SPHSITLRIYMRIKHVRAWIPRPLRNQPYLFKTNPNYKGNDIKCTSTSRDRITTL.
QWY15857.1   LDYGQCPNNMMGTFSIRTVGTEK.SPHSITLRIYMRIKHVRAWIPRPLRNQPYLFKTNPNYKGNDIKCTSTSRDKITTL.
QWY15904.1   LDYGQCPNNMMGTFSIRTVGTEK.SPHSITLRIYMRIKHVRAWIPRPLRNQPYLFKTNPNYKGNDIKCTSTSRDKITTL.
BBH72204.1   LDYGQCPNNMMGTFSVRTVGTEK.SPHSITLRIYMRIKHVRAWIPRPLRNQPYLFKTNPNYKGNDIKCTSTSRDKITTL.
BBH72203.1   LDYGQCPNNMMGTFSVRTVGTEK.SPHSITLRIYMRIKHVRAWIPRPLRNQPYLFKTNPNYKGNDIKCTSTSRDKITTL.
BBH72202.1   LDYGQCPNNMMGTFSVRTVGTEK.SPHSITLRIYMRIKHVRAWIPRPLRNQPYLFKTNPNYKGNDIKCTSTSRDKITTL.
BDC16266.1   LDYGQCPNNMMGTFSVRTVGTEK.SPHSITLRIYMRIKHVRAWIPRPLRNQPYLFKTNPNYKGNDIKCTSTSRDKITTL.
BCR43091.1   LDYGQCPNNMMGTFSVRTVGTEK.SPHSITLRIYMRIKHVRAWIPRPLRNQPYLFKTNPNYKGNDIKCTSTSRDKITTL.
QWY15902.1   LDYGQCPNNMMGTFSIRTVGTEK.SPHSITLRIYMRIKHVRAWIPRPLRNQPYLFKTNPNYKGNDIKCTSTSRDKITTL.
BBH72226.1   LSYGQCPNNMLGTFSARVVSKQV.TSQKFQIRIYLRLKRVRAWVPRPLRSQPYIYKNYPTY.GTTIQHLAKDRRKITET.
BBH72222.1   LSYGQCPNNMLGTFSARVVSKQV.TSQKFQIRIYLRLKRVRAWVPRPLRSQPYIYKNYPTY.GTTIQHLAKDRRKITET.
BBH72012.1   LQYGQCPNNMMGHFAIRTVSEST.TGKNVHVRVYMRIKHVRAWVPRPLRSQAYMVKNYPTY.SQTITNTATDRASITTTD
BBH71965.1   LQYGQCPNNMMGHFAIRTVSEST.TGKNVHVRVYMRIKHVRAWVPRPLRSQAYMVKNYPTY.SQTITNTATDRASITTTD
BBH71934.1   LQYGQCPNNMMGHFAIRTVSEST.TGKNVHVRVYMRIKHVRAWVPRPLRSQAYMVKNYPTY.SQTITNTATDRASITTTD
BBH72009.1   LQYGQCPNNMMGHFAIRTVSEST.TGKNVHVRVYMRIKHVRAWVPRPLRSQAYMVKNYPTY.SQTITNTATDRASITTTD
QWY16175.1   LNYGQCPNNKMGTFCIRMVSGVS.TGKDVTVRIFMKLKHVRAWVPRPIRSQPYLLKNYPNFDKANIADASSNRTSITTT.
QWY16167.1   LNYGQCPNNKMGTFCIRMVSGVS.TGKDVTVRIFMKLKHVRAWVPRPIRSQPYLLKNYPNFDKANIADASSNRTSITTT.
```

```
QWY16174.1     LNYGQCPNNKMGTFCIRMVSGVS.TGKDVTVRIFMKLKHVRAWVPRPIRSQPYLLKNYPNFDKANIVDASSNRTSIVTT.
QWY16171.1     LNYGQCPNNKMGTFCIRMVSGVS.TGKDVTVRIFMKLKHVRAWVPRPIRSQPYLLKNYPNFDKANIVDASSNRTSIVTT.
QWY16173.1     LNYGQCPNNKMGTFCIRMVSGVS.TGKDVTVRIFMKLKHVRAWVPRPIRSQPYLLKNYPNFDKANIVDASSNRTSIITT.
QWY16172.1     LNYGQCPNNKMGTFCIRMVSGVS.TGKDVTVRIFMKLKHVRAWVPRPIRSQPYLLKNYPNFDKANIVDASSNRTSIITT.
QWY16170.1     LNYGQCPNNKMGTFCIRMVSGVS.TGKDVTVRIFMKLKHVRAWVPRPIRSQPYLLKNYPNFDKANIVDASSNRTSIITT.
QWY16169.1     LNYGQCPNNKMGTFCIRMVSGVS.TGKDVTVRIFMKLKHVRAWVPRPIRSQPYLLKNYPNFDKANIVDASFNRTSITTT.
QWY16168.1     LNYGQCPNNKMGTFCIRMVSGVS.TGKDVTVRIFMKLKHVRAWVPRPIRSQPYLLKNYPNFDKANIVDASFNRTSITTT.
QXF31588.1     MNYGQCPNNKMGTFCMRTVSGEA.SGKNITVRIFMRLKHVRAWVPRPIRSQLYLLKNYPNFDNTKILNASHNRTSITANT
QXF31587.1     MNYGQCPNNKMGTFCMRTVSGEA.SGKNITVRIFMRLKHVRAWVPRPIRSQLYLLKNYPNFDNTKILNASHNRTSITANT
pdb|6IIO|A     TTYGLCPNNMMGTFAVRVVSREA.SQLKLQTRVYMKLKHVRAWVPRPIRSQPYLLKNFPNYDSSKITNSARDRSSIKQAN
pdb|6IIJ|A     TTYGLCPNNMMGTFAVRVVSREA.SQLKLQTRVYMKLKHVRAWVPRPIRSQPYLLKNFPNYDSSKITNSARDRSSIKQAN
pdb|6AKS|A     TTYGLCPNNMMGTFAVRVVSREA.SQLKLQTRVYMKLKHVRAWVPRPIRSQPYLLKNFPNYDSSKITNSARDRSSIKQAN
BCD33944.1     .DYGRINSSHMGHLALRAVAPLA.TGEVVKLRVYAKPKHVRAWGPRAPRMSPYVEKATNLFSNPTN..IVPNRANVTTAG
pdb|7ECY|A     GLYGINPADTIGNLCVRIVNEHQPVGFTVTVRVYMKPKHIKAWAPRPPRTLPYMSIANANYKGKERAPNALSAIIGNRDS
pdb|7EC5|A     GLYGINPADTIGNLCVRIVNEHQPVGFTVTVRVYMKPKHIKAWAPRPPRTLPYMSIANANYKGKERAPNALSAIIGNRDS
pdb|7EBZ|A     GLYGINPADTIGNLCVRIVNEHQPVGFTVTVRVYMKPKHIKAWAPRPPRTLPYMSIANANYKGKERAPNALSAIIGNRDS
pdb|7EBR|A     GLYGINPADTIGNLCVRIVNEHQPVGFTVTVRVYMKPKHIKAWAPRPPRTLPYMSIANANYKGKERAPNALSAIIGNRDS
pdb|6MZI|A     GLYGINPADTIGNLCVRIVNEHQPVGFTVTVRVYMKPKHIKAWAPRPPRTLPYMSIANANYKGKERAPNALSAIIGNRDS
pdb|6CSH|A     GLYGINPADTIGNLCVRIVNEHQPVGFTVTVRVYMKPKHIKAWAPRPPRTLPYMSIANANYKGKERAPNALSAIIGNRDS
pdb|6CSG|A     GLYGINPADTIGNLCVRIVNEHQPVGFTVTVRVYMKPKHIKAWAPRPPRTLPYMSIANANYKGKERAPNALSAIIGNRDS
pdb|6CSA|A     GLYGINPADTIGNLCVRIVNEHQPVGFTVTVRVYMKPKHIKAWAPRPPRTLPYMSIANANYKGKERAPNALSAIIGNRDS
pdb|6CS6|A     GLYGINPADTIGNLCVRIVNEHQPVGFTVTVRVYMKPKHIKAWAPRPPRTLPYMSIANANYKGKERAPNALSAIIGNRDS
pdb|6CS5|A     GLYGINPADTIGNLCVRIVNEHQPVGFTVTVRVYMKPKHIKAWAPRPPRTLPYMSIANANYKGKERAPNALSAIIGNRDS
pdb|6CS4|A     GLYGINPADTIGNLCVRIVNEHQPVGFTVTVRVYMKPKHIKAWAPRPPRTLPYMSIANANYKGKERAPNALSAIIGNRDS
pdb|6CS3|A     GLYGINPADTIGNLCVRIVNEHQPVGFTVTVRVYMKPKHIKAWAPRPPRTLPYMSIANANYKGKERAPNALSAIIGNRDS
pdb|6CRU|A     GLYGINPADTIGNLCVRIVNEHQPVGFTVTVRVYMKPKHIKAWAPRPPRTLPYMSIANANYKGKERAPNALSAIIGNRDS
pdb|6CRS|A     GLYGINPADTIGNLCVRIVNEHQPVGFTVTVRVYMKPKHIKAWAPRPPRTLPYMSIANANYKGKERAPNALSAIIGNRDS
pdb|6CRR|A     GLYGINPADTIGNLCVRIVNEHQPVGFTVTVRVYMKPKHIKAWAPRPPRTLPYMSIANANYKGKERAPNALSAIIGNRDS
pdb|6CRP|A     GLYGINPADTIGNLCVRIVNEHQPVGFTVTVRVYMKPKHIKAWAPRPPRTLPYMSIANANYKGKERAPNALSAIIGNRDS
QNO39042.1     GLYGINPADTIGNLCVRIVNEHQPVGFTVTVRVYMKPKHIKAWAPRPPRTLPYMSIANANYKGKERAPNALSAIIGNRDS
QNO39041.1     GLYGINPADTIGNLCVRIVNEHQPVGFTVTVRVYMKPKHIKAWAPRPPRTLPYMSIANANYKGKERAPNALSAIIGNRDS
QNO39040.1     GLYGINPADTIGNLCVRIVNEHQPVGFTVTVRVYMKPKHIKAWAPRPPRTLPYMSIANANYKGKERAPNALNAIIGNRDS
QNO39038.1     GLYGINPADTIGNLCVRIVNEHQPVGFTVTVRVYMKPKHIKAWAPRPPRTLPYMSIANANYKGKERAPNALNAIIGNRDS
QNO39039.1     GLYGINPADTIGNLCVRIVNEHQPVGFTVTVRVYMKPKHIKAWAPRPPRTLPYMSIANANYKGKERAPNALNAIIGNRDS
CAA1539354.1   GLYGINPADTIGNLCVRIVNEHQPVGFTVTVRVYMKPKHIKAWAPRPPRTLPYMSIANANYKGXERAPNALNAIIGNRDN
CAA1527138.1   GLYGINPADTIGNLCVRIVNEHQPVGFTVTVRVYMKPKHIKAWAPRPPRTLPYMSIANANYKGXERAPNALNAIIGNRDN
CAA1539353.1   GLYGINPADTIGNLCVRIVNEHQPVGFTVTVRVYMKPKHIKAWAPRPPRTLPYMSIANANYKGKERAPNALNAIIGNRDN
CAA1527136.1   GLYGINPADTIGNLCVRIVNEHQPVGFTVTVRVYMKPKHIKAWAPRPPRTLPYMSIANANYKGKERAPNALNAIIGNRDN
QNO39037.1     GLYGINPADTIGNLCVRIVNEHQPVGFTVTVRVYMKPKHIKAWAPRPPRTLPYMSIANANYKGKERAPNALNAIIGNRDS
QKX95883.1     GLYGINPADTIGNLCVRIVNEHQPVGFTVTVRVYMKPKHIKAWAPRPPRTLPYMSIANTNYKGKERAXNALNAIIGNRD.
pdb|5BNP|A     GLYGINPADTIGNLCVRIVNEHQPVGFTVTVRVYMKPKHIKAWAPRPPRTMPYMSIANANYKGRDTAPNTLNAIIGNRAS
pdb|5BNO|A     GLYGINPADTIGNLCVRIVNEHQPVGFTVTVRVYMKPKHIKAWAPRPPRTMPYMSIANANYKGRDTAPNTLNAIIGNRAS
pdb|5BNN|A     GLYGINPADTIGNLCVRIVNEHQPVGFTVTVRVYMKPKHIKAWAPRPPRTMPYMSIANANYKGRDTAPNTLNAIIGNRAS
pdb|4WM8|A     GLYGINPADTIGNLCVRIVNEHQPVGFTVTVRVYMKPKHIKAWAPRPPRTMPYMSIANANYKGRDTAPNTLNAIIGNRAS
pdb|4WM7|A     GLYGINPADTIGNLCVRIVNEHQPVGFTVTVRVYMKPKHIKAWAPRPPRTMPYMSIANANYKGRDTAPNTLNAIIGNRAS
QIC50353.1     NKYGYSSINDFGTLAVRIVNEYDPVTIDAKIRVYLKPKHVKVWCPRPPRAVAYNGPT.VNFS...ENPSVMTQVADIRTY
QIC50352.1     NKYGYSSINDFGTLAIRIVNEYDPVTIDAKIRVYLKPKHVKVWCPRPPRAVAYNGPT.VNFS...ENPSVMTQVADIRTY
QIC50356.1     NKYGYSSINDFGTMAVRIVNEYDPVTINAKIRVYMKPKHIKVWCPRPPRAMPYVNSS.VDFK...PTDKVMTQVASIRTY
```

QUS47950.1

QUS47950.1	....
QWY16112.1	....
QWY16110.1	....
QWY16109.1	....
QWY16107.1	....
QWY16106.1	....
QWY16105.1	....
QWY16104.1	....
QNN25841.1	....
QNN25838.1	....
QNN25837.1	....
QNN25842.1	....
QNN25843.1	....
QWY16092.1	....
QWY16090.1	....
QWY16084.1	....
QWY16083.1	....
QWY16081.1	....
QWY16080.1	....
QWY16079.1	....
QWY16078.1	....
QWY16075.1	....
QWY16074.1	....
QWY16056.1	....
QWY16147.1	....
QWY16146.1	....
QOP74909.1	....
QUS47948.1	....
QUS47947.1	....
QUS47946.1	....
QUS47944.1	....
QUS47942.1	....
QUS47941.1	....
QUS47940.1	....
QUS47939.1	....
QUS47928.1	....
QUS47926.1	....
QUS47921.1	....
QUS47920.1	....
QNN25845.1	....
QNN25836.1	....
QWY16162.1	....
QWY16161.1	....
QWY16159.1	....
QWY16158.1	....
QWY16155.1	....
QWY16153.1	....
QWY16151.1	....
QWY16145.1	....
QWY16143.1	....
QWY16142.1	....
QWY16139.1	....
QWY16138.1	....
QWY16135.1	....
QWY16134.1	....
QWY16133.1	....
QWY16132.1	....
QWY16131.1	....
QWY16130.1	....
QWY16129.1	....
QWY16127.1	....
QWY16125.1	....
QWY16123.1	....
QWY16121.1	....
QWY16118.1	....
QWY16117.1	....
QWY16116.1	....
QWY16099.1	....
QWY16096.1	....
QWY16095.1	....
QWY16085.1	....
QWY16077.1	....
QWY16072.1	....
QWY16071.1	....
QWY16064.1	....
QWY16059.1	....
QWY16054.1	....
QWY16052.1	....
QWY16051.1	....
QWY16050.1	....
QWY16046.1	....
QWY16045.1	....
QWY16041.1	....
QWY16040.1	....
QWY16032.1	....
QWY16017.1	....
QWY16016.1	....
QWY16015.1	....
QTA73890.1	....
QTA73885.1	....
BCG67033.1	....
BBU37474.1	....
BBU37473.1	....
BBU37471.1	....
BBU37469.1	....
BBU37468.1	....
BBU37467.1	....
BBU37466.1	....
BBU37465.1	....
BBU37464.1	....
BBU37463.1	....
QOP74908.1	....
QOP74907.1	....
QOP74903.1	....
QOP74902.1	....
QOP74899.1	....
QOP74896.1	....
QWY16156.1	....
QUS47932.1	....
QWY16136.1	....
QWY16044.1	....
QUS47923.1	....
QWY16152.1	....
QWY16124.1	....
QWY16018.1	....
BBU37475.1	....
QUS47938.1	....
QWY16076.1	....
QWY16122.1	....
QOP74905.1	....
QWY16160.1	....
QWY16070.1	....
QWY16068.1	....
QWY16066.1	....
QWY16062.1	....
QWY16034.1	....
QTA73887.1	....
QTA73883.1	....
BBU37478.1	....
BBU37470.1	....
QWY16103.1	....
QWY16065.1	....
QTA73886.1	....
QWY16113.1	....
QWY16111.1	....
QWY16098.1	....
QWY16060.1	....
QUS47945.1	....
QOP74904.1	....
QUS47924.1	....
QWY15991.1	....
QWY15985.1	....
QWY16165.1	....
QWY16038.1	....

```
QWY16036.1    ..........
QWY16120.1    ..........
QWY16119.1    ..........
QWY16102.1    ..........
QWY16100.1    ..........
QWY16097.1    ..........
QWY16101.1    ..........
BBU37472.1    ..........
QWY16108.1    ..........
QWY16042.1    ..........
QOP74910.1    ..........
QWY16128.1    ..........
QUS47905.1    ..........
QWY16154.1    ..........
QWY16126.1    ..........
QWY16049.1    ..........
QWY16043.1    ..........
QUS47911.1    ..........
BBU37477.1    ..........
QWY16137.1    ..........
QWY16061.1    ..........
QTA73882.1    ..........
QWY16031.1    ..........
BBU37476.1    ..........
QUS47949.1    ..........
QNN25833.1    ..........
QWY16114.1    ..........
QWY16063.1    ..........
QTA73884.1    ..........
QWY16002.1    ..........
QWY15984.1    ..........
QUS47934.1    ..........
QUS47933.1    ..........
QUS47929.1    ..........
QUS47927.1    ..........
QUS47919.1    ..........
QUS47917.1    ..........
QUS47915.1    ..........
QUS47913.1    ..........
QWY16166.1    ..........
QWY16164.1    ..........
QWY16163.1    ..........
QWY16157.1    ..........
QWY16150.1    ..........
QWY16115.1    ..........
QWY16087.1    ..........
QWY16069.1    ..........
QWY16058.1    ..........
QWY16048.1    ..........
QWY16047.1    ..........
QWY16030.1    ..........
QWY16027.1    ..........
QWY16021.1    ..........
QWY16020.1    ..........
QWY16009.1    ..........
QWY16008.1    ..........
QWY16007.1    ..........
QWY16001.1    ..........
QWY16000.1    ..........
QWY15993.1    ..........
QWY15986.1    ..........
QWY15979.1    ..........
QTA73889.1    ..........
QTA73874.1    ..........
QTA73871.1    ..........
QOP74901.1    ..........
QOP74900.1    ..........
QOP74897.1    ..........
QOP74893.1    ..........
QOP74887.1    ..........
QOP74886.1    ..........
QOP74884.1    ..........
QOP74882.1    ..........
QOP74881.1    ..........
QOP74880.1    ..........
QOP74879.1    ..........
QOP74878.1    ..........
pdb|4YVS|M    ..........
pdb|4YVS|J    ..........
pdb|4YVS|G    ..........
pdb|4YVS|D    ..........
pdb|4YVS|A    ..........
pdb|4CEY|A    ..........
pdb|4CEW|A    ..........
pdb|4N53|A    ..........
pdb|4N43|A    ..........
pdb|4RQP|A    ..........
pdb|4RQP|M    ..........
pdb|4RQP|I    ..........
pdb|4RQP|Q    ..........
pdb|4RQP|E    ..........
QWY16033.1    ..........
QWY16029.1    ..........
QWY16028.1    ..........
QWY16025.1    ..........
QUS47943.1    ..........
QOP74914.1    ..........
QUS47916.1    ..........
QWY16148.1    ..........
QOP74906.1    ..........
QOP74895.1    ..........
QOP74890.1    ..........
QOP74889.1    ..........
QWY16149.1    ..........
QOP74888.1    ..........
QOP74894.1    ..........
QUS47930.1    ..........
QWY16053.1    ..........
QWY15997.1    ..........
QWY15996.1    ..........
QWY15995.1    ..........
QTA73878.1    ..........
QTA73877.1    ..........
QTA73876.1    ..........
QUS47914.1    ..........
QWY16003.1    ..........
QWY16067.1    ..........
QWY16057.1    ..........
QTA73888.1    ..........
QWY16086.1    ..........
pdb|4GMP|1    ..........
pdb|3J91|1    ..........
QWY16094.1    ..........
QWY16093.1    ..........
QWY16091.1    ..........
QWY16088.1    ..........
QWY16082.1    ..........
QWY16073.1    ..........
QWY16055.1    ..........
QUS47918.1    ..........
QWY16089.1    ..........
QWY16037.1    ..........
QOP74891.1    ..........
pdb|3VBS|A    ..........
pdb|3VBO|A    ..........
pdb|3VBH|A    ..........
pdb|3VBF|A    ..........
pdb|6LQD|A    ..........
pdb|5ZUF|A    ..........
pdb|5ZUD|A    ..........
pdb|4CDX|A    ..........
pdb|4CDW|A    ..........
pdb|4CDU|A    ..........
pdb|4CDQ|A    ..........
```

```
QOP74885.1    ...........
QWY16026.1    ...........
QWY15977.1    ...........
QTA73869.1    ...........
QUS47935.1    ...........
QWY16024.1    ...........
QWY16023.1    ...........
QWY16022.1    ...........
QWY16013.1    ...........
QWY16012.1    ...........
QWY15992.1    ...........
QWY15982.1    ...........
QWY15981.1    ...........
QWY15976.1    ...........
QTA73880.1    ...........
QTA73872.1    ...........
QTA73868.1    ...........
QOP74892.1    ...........
QWY15998.1    ...........
QWY15994.1    ...........
QTA73879.1    ...........
QTA73875.1    ...........
QWY16006.1    ...........
QWY16005.1    ...........
QWY16039.1    ...........
QWY15999.1    ...........
QWY16004.1    ...........
QOP74913.1    ...........
QOP74915.1    ...........
QWY16035.1    ...........
QWY15990.1    ...........
QWY15988.1    ...........
QWY15989.1    ...........
QWY15987.1    ...........
QWY15978.1    ...........
QTA73870.1    ...........
QWY15983.1    ...........
QUS47931.1    ...........
QUS47925.1    ...........
QUS47922.1    ...........
QWY16014.1    ...........
QTA73881.1    ...........
QOP74898.1    ...........
QWY15980.1    ...........
QTA73873.1    ...........
QWY16011.1    ...........
QWY16010.1    ...........
QOP74911.1    ...........
QWY16019.1    ...........
QOP74883.1    ...........
QUS47909.1    ...........
QWY16144.1    ...........
QUS47908.1    ...........
QNN25844.1    ...........
QNN25840.1    ...........
QNN25839.1    ...........
QNN25835.1    ...........
QNN25834.1    ...........
QOP74912.1    ...........
QUS47910.1    ...........
QUS47907.1    ...........
QWY16141.1    ...........
QWY16140.1    ...........
QUS47937.1    ...........
QUS47936.1    ...........
QUS47912.1    ...........
QUS47906.1    ...........
pdb|4YVW|M    ...........
pdb|4YVW|J    ...........
pdb|4YVW|D    ...........
pdb|4YVW|A    ...........
pdb|4YVW|E    ...........
QPI19797.1    ...........
QPI19781.1    ...........
QPI19795.1    ...........
QPI19794.1    ...........
QPI19793.1    ...........
QPI19792.1    ...........
QPI19790.1    ...........
QPI19789.1    ...........
QPI19788.1    ...........
QPI19787.1    ...........
QPI19785.1    ...........
QPI19784.1    ...........
QPI19782.1    ...........
QPI19780.1    ...........
QPI19777.1    ...........
QPI19762.1    ...........
QPI19755.1    ...........
QPI19754.1    ...........
QPI19748.1    ...........
QPI19747.1    ...........
QPI19744.1    ...........
QPI19743.1    ...........
QPI19739.1    ...........
QPI19778.1    ...........
QPI19796.1    ...........
QPI19753.1    ...........
QPI19791.1    ...........
QPI19786.1    ...........
QPI19779.1    ...........
QPI19742.1    ...........
pdb|4AED|A    ...........
pdb|3ZFF|A    ...........
pdb|6DIZ|A    ...........
pdb|6UH7|A    ...........
pdb|6UH6|A    ...........
pdb|6UH1|A    ...........
QPI19783.1    ...........
QPI19772.1    ...........
QPI19771.1    ...........
QPI19764.1    ...........
QPI19760.1    ...........
QPI19759.1    ...........
QPI19756.1    ...........
QPI19752.1    ...........
QPI19740.1    ...........
QPI19738.1    ...........
QPI19757.1    ...........
QPI19750.1    ...........
QPI19751.1    ...........
QPI19746.1    ...........
QPI19737.1    ...........
QPI19776.1    ...........
QPI19774.1    ...........
QPI19773.1    ...........
QPI19770.1    ...........
QPI19769.1    ...........
QPI19768.1    ...........
QPI19767.1    ...........
QPI19766.1    ...........
QPI19763.1    ...........
QPI19761.1    ...........
QPI19765.1    ...........
QPI19775.1    ...........
QPI19758.1    ...........
QPI19736.1    ...........
QPI19735.1    ...........
QPI19749.1    ...........
QPI19741.1    ...........
QPI19745.1    ...........
pdb|6Z3Q|A    ...........
pdb|6Z3P|A    ...........
pdb|6I2K|A    ...........
```

```
QWF08740.1    ...........
QWF08739.1    ...........
QWF08760.1    ...........
QWF08759.1    ...........
QWF08762.1    ...........
QWF08761.1    ...........
QWF08764.1    ...........
QWF08763.1    ...........
QWF08773.1    ...........
QWF08749.1    ...........
QWF08785.1    ...........
QWF08783.1    ...........
QWF08787.1    ...........
QWF08765.1    ...........
QWF08792.1    ...........
QWF08767.1    ...........
QWF08793.1    ...........
QWF08758.1    ...........
QWF08845.1    ...........
QWF08844.1    ...........
QWF08847.1    ...........
QWF08846.1    ...........
QWF08852.1    ...........
QWF08843.1    ...........
QWF08857.1    ...........
QWF08856.1    ...........
QWF08865.1    ...........
QWF08738.1    ...........
QWF08872.1    ...........
QWF08871.1    ...........
QWF08879.1    ...........
QWF08877.1    ...........
QWF08880.1    ...........
QWF08851.1    ...........
QWF08882.1    ...........
QWF08867.1    ...........
QWF08884.1    ...........
QWF08875.1    ...........
QWF08885.1    ...........
QWF08863.1    ...........
QWF08890.1    ...........
QWF08889.1    ...........
QWF08896.1    ...........
QWF08752.1    ...........
QWF08900.1    ...........
QWF08899.1    ...........
QWF08908.1    ...........
QWF08906.1    ...........
QWF08910.1    ...........
QWF08870.1    ...........
QWF08915.1    ...........
QWF08750.1    ...........
QWF08917.1    ...........
QWF08916.1    ...........
QWF08923.1    ...........
QWF08921.1    ...........
QWF08926.1    ...........
QWF08780.1    ...........
QWF08932.1    ...........
QWF08931.1    ...........
QWF08945.1    ...........
QWF08938.1    ...........
QWF08955.1    ...........
QWF08954.1    ...........
QWF08967.1    ...........
QWF08959.1    ...........
QYC65038.1    ...........
QYC65037.1    ...........
QYC65039.1    ...........
QYC65034.1    ...........
QYC65043.1    ...........
QYC65041.1    ...........
QYC65047.1    ...........
QYC65046.1    ...........
QYC65050.1    ...........
QYC65049.1    ...........
QYC65051.1    ...........
QYC65045.1    ...........
QYC65058.1    ...........
QYC65055.1    ...........
QYC65060.1    ...........
QYC65059.1    ...........
QYC65062.1    ...........
QYC65061.1    ...........
QYC65063.1    ...........
QYC65053.1    ...........
QYC65069.1    ...........
QYC65068.1    ...........
QYC65071.1    ...........
QYC65070.1    ...........
QYC65074.1    ...........
QYC65066.1    ...........
QYC65079.1    ...........
QYC65073.1    ...........
QYC65081.1    ...........
QYC65078.1    ...........
QYC65086.1    ...........
QYC65085.1    ...........
QYC65088.1    ...........
QYC65087.1    ...........
QYC65101.1    ...........
QYC65093.1    ...........
QYC65107.1    ...........
QYC65104.1    ...........
QYC65113.1    ...........
QYC65106.1    ...........
QYC65120.1    ...........
QYC65118.1    ...........
QYC65121.1    ...........
QYC65111.1    ...........
QYC65125.1    ...........
QYC65117.1    ...........
QYC65135.1    ...........
QYC65134.1    ...........
QYC65151.1    ...........
QYC65150.1    ...........
QYC65153.1    ...........
QYC65152.1    ...........
QYC65155.1    ...........
QYC65154.1    ...........
QYC65156.1    ...........
QYC65142.1    ...........
QYC65161.1    ...........
QYC65160.1    ...........
QYC65165.1    ...........
QYC65164.1    ...........
QYC65167.1    ...........
QYC65166.1    ...........
BCB92151.1    ...........
BBU37462.1    ...........
BBU37461.1    ...........
BBU37459.1    ...........
BBU37458.1    ...........
BBU37457.1    ...........
QWF08838.1    ...........
QWF08828.1    ...........
BCG66989.1    ...........
BCG66987.1    ...........
BCB92159.1    ...........
BBU37456.1    ...........
QWY15683.1    ...........
QWY15682.1    ...........
QWY15681.1    ...........
QWY15680.1    ...........
```

```
QWY15692.1     ...........
QWY15671.1     ...........
QWY15691.1     ...........
QWY15690.1     ...........
QWY15696.1     ...........
QWY15695.1     ...........
QWY15694.1     ...........
QWY15678.1     ...........
QWY15703.1     ...........
QWY15701.1     ...........
QWY15699.1     ...........
QWY15673.1     ...........
QWY15715.1     ...........
QWY15714.1     ...........
QWY15706.1     ...........
QWY15705.1     ...........
QTA73914.1     ...........
QWY15948.1     ...........
QWF08837.1     ...........
QWF08804.1     ...........
QWF08805.1     ...........
QWF08840.1     ...........
QWY15792.1     ...........
QTA73903.1     ...........
QTA73902.1     ...........
QTA73896.1     ...........
QTA73894.1     ...........
QWY15940.1     ...........
QWY15928.1     ...........
QWY15894.1     ...........
BCG67001.1     ...........
QWY15872.1     ...........
QWY15871.1     ...........
QWY15870.1     ...........
QWY15869.1     ...........
QWY15866.1     ...........
BCB92155.1     ...........
QWY15862.1     ...........
QWY15861.1     ...........
QWY15852.1     ...........
QWY15828.1     ...........
QWY15839.1     ...........
QWY15834.1     ...........
QWY15810.1     ...........
QWY15809.1     ...........
QWY15807.1     ...........
QWY15806.1     ...........
QWY15805.1     ...........
QWY15803.1     ...........
QWY15793.1     ...........
QYC65185.1     ...........
QWY15754.1     ...........
QWY15736.1     ...........
QWY15753.1     ...........
QYC65174.1     ...........
QWY15730.1     ...........
QWY15729.1     ...........
QWY15727.1     ...........
QWY15726.1     ...........
QWY15725.1     ...........
QWY15724.1     ...........
QWY15723.1     ...........
QWY15721.1     ...........
QWY15746.1     ...........
QWY15745.1     ...........
QWY15733.1     ...........
QYC65131.1     ...........
QWY15813.1     ...........
QWY15811.1     ...........
QTA73907.1     ...........
QTA73906.1     ...........
QOP74940.1     ...........
QOP74938.1     ...........
QOP74937.1     ...........
QOP74934.1     ...........
QOP74929.1     ...........
QOP74924.1     ...........
QOP74921.1     ...........
QOP74932.1     ...........
QOP74931.1     ...........
QIL87531.1     ...........
QIL87521.1     ...........
QIL87523.1     ...........
QIL87517.1     ...........
QIL87540.1     ...........
QIL87539.1     ...........
QIH53921.1     ...........
QIH53916.1     ...........
BBH72194.1     ...........
BBH72183.1     ...........
BBH72107.1     ...........
QWF08825.1     ...........
QWF08823.1     ...........
QWF08821.1     ...........
QWF08818.1     ...........
QWF08815.1     ...........
QWF08813.1     ...........
QWF08812.1     ...........
QWF08810.1     ...........
QWF08809.1     ...........
QWF08808.1     ...........
QWF08807.1     ...........
QWF08797.1     ...........
QWF08841.1     ...........
QWF08830.1     ...........
QTA73892.1     ...........
QTA73901.1     ...........
QWY15957.1     ...........
UEP53406.1     ...........
UEC49858.1     ...........
QWY15931.1     ...........
QWY15929.1     ...........
QWY15880.1     ...........
QWY15868.1     ...........
BCI65368.1     ...........
BCR43097.1     ...........
BCG67047.1     ...........
BCG67046.1     ...........
BCG67041.1     ...........
BCG67040.1     ...........
BCG67039.1     ...........
BCG67038.1     ...........
BCG67032.1     ...........
BCG67030.1     ...........
BCG66995.1     ...........
BCG66994.1     ...........
QWY15757.1     ...........
QWY15756.1     ...........
QWY15748.1     ...........
QWY15747.1     ...........
QWY15720.1     ...........
QWY15717.1     ...........
QWY15697.1     ...........
QWY15689.1     ...........
QWY15761.1     ...........
QWY15743.1     ...........
QWY15759.1     ...........
QWY15758.1     ...........
QWY15677.1     ...........
QWY15675.1     ...........
QWY15776.1     ...........
QWY15772.1     ...........
QWY15770.1     ...........
QWY15762.1     ...........
```

```
QWY15732.1    ...........
QWY15731.1    ...........
QWY15780.1    ...........
QWY15779.1    ...........
BCG67006.1    ...........
BCG67004.1    ...........
BCG67019.1    ...........
BCG67002.1    ...........
BCG67036.1    ...........
BCG67035.1    ...........
QWY15785.1    ...........
QWY15784.1    ...........
QWY15789.1    ...........
QYC65159.1    ...........
BCR43101.1    ...........
BCR43098.1    ...........
QWY15873.1    ...........
QWY15865.1    ...........
QWY15900.1    ...........
BCG67049.1    ...........
UEC49853.1    ...........
UEC49848.1    ...........
QTA73899.1    ...........
QTA73898.1    ...........
QTA73905.1    ...........
QTA73904.1    ...........
QTA73909.1    ...........
QTA73908.1    ...........
UEP53408.1    ...........
QWF08839.1    ...........
QWF08799.1    ...........
QWY15771.1    ...........
QTA73891.1    ...........
QWY15670.1    ...........
BBH72149.1    ...........
BBH72143.1    ...........
BBH72163.1    ...........
QTA73895.1    ...........
QWY15674.1    ...........
QWY15710.1    ...........
QWY15709.1    ...........
QWF08934.1    ...........
QWF08836.1    ...........
QLH02014.1    ...........
QWF08796.1    ...........
QYC65090.1    ...........
QTA73900.1    ...........
QWY15679.1    ...........
QWY15751.1    ...........
QWF08795.1    ...........
QWF08789.1    ...........
QYC65158.1    ...........
QYC65109.1    ...........
QWF08883.1    ...........
QWF08866.1    ...........
QWF08833.1    ...........
QWF08801.1    ...........
QWF08842.1    ...........
QWF08806.1    ...........
QYC65065.1    ...........
QYC65044.1    ...........
QWY15863.1    ...........
QTA73916.1    ...........
BCG66990.1    ...........
QTA73915.1    ...........
QWY15932.1    ...........
QWY15867.1    ...........
QWY15859.1    ...........
QWY15858.1    ...........
QWY15782.1    ...........
QWY15781.1    ...........
BCI65355.1    ...........
BCI65374.1    ...........
BCI65373.1    ...........
BCI65372.1    ...........
BCI65371.1    ...........
BCI65369.1    ...........
BCI65367.1    ...........
BCI65365.1    ...........
BCI65364.1    ...........
BCI65363.1    ...........
BCI65362.1    ...........
BCI65361.1    ...........
BCI65360.1    ...........
BCI65359.1    ...........
BCI65358.1    ...........
BCI65356.1    ...........
BCI65354.1    ...........
BCI65353.1    ...........
BCI65352.1    ...........
BCI65351.1    ...........
BCI65350.1    ...........
BCI65349.1    ...........
BCI65348.1    ...........
BCI65347.1    ...........
BCI65346.1    ...........
BCI65345.1    ...........
BCI65344.1    ...........
BCI65343.1    ...........
BCI65342.1    ...........
BCI65341.1    ...........
BCI65340.1    ...........
BCI65339.1    ...........
BCI65338.1    ...........
BCI65337.1    ...........
BCI65336.1    ...........
BCI65335.1    ...........
BCI65334.1    ...........
BCI65333.1    ...........
BCI65332.1    ...........
BCI65331.1    ...........
BCI65330.1    ...........
BCI65328.1    ...........
BCI65327.1    ...........
BCI65326.1    ...........
BCI65325.1    ...........
BCI65324.1    ...........
BCI65323.1    ...........
BCI65322.1    ...........
BCI65321.1    ...........
BCI65320.1    ...........
BCI65319.1    ...........
BCI65318.1    ...........
BCI65317.1    ...........
QYC65119.1    ...........
QWF08905.1    ...........
QWF08814.1    ...........
QWF08776.1    ...........
BCI65370.1    ...........
BCG67048.1    ...........
QWY15933.1    ...........
UEP53404.1    ...........
UEC49857.1    ...........
QYC65076.1    ...........
QWF08819.1    ...........
QYC65082.1    ...........
QWY15927.1    ...........
QOP74944.1    ...........
QYC65139.1    ...........
QYC65136.1    ...........
QYC65130.1    ...........
QYC65115.1    ...........
QYC65114.1    ...........
QYC65105.1    ...........
```

```
QWF08949.1    ...........
QWF08928.1    ...........
QWY15921.1    ...........
QWY15919.1    ...........
QYC65102.1    ...........
BCR43082.1    ...........
QWY15713.1    ...........
BCI65329.1    ...........
QWF08887.1    ...........
QYC65036.1    ...........
QTA73913.1    ...........
QWY15837.1    ...........
QWY15738.1    ...........
QTA73911.1    ...........
QTA73910.1    ...........
QWY15916.1    ...........
QWY15915.1    ...........
QWY15835.1    ...........
QWY15832.1    ...........
QWY15830.1    ...........
QWY15829.1    ...........
QWY15826.1    ...........
QWY15802.1    ...........
QWY15799.1    ...........
QWY15788.1    ...........
QWY15783.1    ...........
QWY15768.1    ...........
QWY15766.1    ...........
QWY15764.1    ...........
QWY15763.1    ...........
QWY15760.1    ...........
QWF08768.1    ...........
QWF08766.1    ...........
QWY15790.1    ...........
QWF08769.1    ...........
QWY15930.1    ...........
QWY15833.1    ...........
QWY15827.1    ...........
QWY15838.1    ...........
BBU37460.1    ...........
QWF08920.1    ...........
QWY15769.1    ...........
QWY15755.1    ...........
QWY15702.1    ...........
QWY15739.1    ...........
QWY15737.1    ...........
QWF08788.1    ...........
QYC65122.1    ...........
QWF08891.1    ...........
QWY15903.1    ...........
BCI65366.1    ...........
BCG67045.1    ...........
BCG67044.1    ...........
QWY15819.1    ...........
QWY15818.1    ...........
QTA73912.1    ...........
QWY15914.1    ...........
QWY15909.1    ...........
QWY15905.1    ...........
QWY15856.1    ...........
QWY15850.1    ...........
QWY15849.1    ...........
QWY15847.1    ...........
QWY15845.1    ...........
QWY15836.1    ...........
QWY15765.1    ...........
QWY15734.1    ...........
QWY15855.1    ...........
QWY15831.1    ...........
QWY15798.1    ...........
QWY15797.1    ...........
QWY15796.1    ...........
QWY15795.1    ...........
QWY15794.1    ...........
QWY15971.1    ...........
QWY15897.1    ...........
QWY15742.1    ...........
QWY15741.1    ...........
QWY15923.1    ...........
QWF08957.1    ...........
UEP53407.1    ...........
QWY15922.1    ...........
QWY15848.1    ...........
UEP53405.1    ...........
UEC49855.1    ...........
QWY15851.1    ...........
QYC65112.1    ...........
QOP74942.1    ...........
QLH02013.1    ...........
QIH53923.1    ...........
QIH53918.1    ...........
QIH53915.1    ...........
QIH53914.1    ...........
QYC65127.1    ...........
QIL87534.1    ...........
QWF08936.1    ...........
QIL87532.1    ...........
QYC65089.1    ...........
QWF08950.1    ...........
QIL87530.1    ...........
QWF08820.1    ...........
QIL87529.1    ...........
QIL87528.1    ...........
QYC65143.1    ...........
QIL87525.1    ...........
QYC65141.1    ...........
QYC65084.1    ...........
QWF08947.1    ...........
QWF08922.1    ...........
QIL87524.1    ...........
QIL87520.1    ...........
QIL87518.1    ...........
QYC65103.1    ...........
QWF08944.1    ...........
QWF08943.1    ...........
QWF08927.1    ...........
QWF08942.1    ...........
QWF08940.1    ...........
QWF08912.1    ...........
QWF08907.1    ...........
QWF08904.1    ...........
QWF08886.1    ...........
QWY15877.1    ...........
QWY15965.1    ...........
UEC49851.1    ...........
UEC49850.1    ...........
UEC49849.1    ...........
QWY15963.1    ...........
QWY15972.1    ...........
QWY15958.1    ...........
QWY15899.1    ...........
QWY15898.1    ...........
QWY15895.1    ...........
QWY15893.1    ...........
QWY15892.1    ...........
QWY15891.1    ...........
QWY15970.1    ...........
QWY15966.1    ...........
QWY15962.1    ...........
QWY15961.1    ...........
QWY15925.1    ...........
QWY15773.1    ...........
QWY15924.1    ...........
QWY15910.1    ...........
```

```
QWY15824.1    ...........
QWY15823.1    ...........
QWY15822.1    ...........
QWY15821.1    ...........
QWY15816.1    ...........
QWY15778.1    ...........
QWY15801.1    ...........
QWY15791.1    ...........
QWY15775.1    ...........
QWY15774.1    ...........
BCR43107.1    ...........
QYC65183.1    ...........
BCR43094.1    ...........
BCR43081.1    ...........
BCG67017.1    ...........
BCG67015.1    ...........
BCG67007.1    ...........
BCG67005.1    ...........
QYC65184.1    ...........
QWF08853.1    ...........
QYC65140.1    ...........
QYC65133.1    ...........
QYC65110.1    ...........
QYC65108.1    ...........
QYC65098.1    ...........
QYC65097.1    ...........
QYC65094.1    ...........
QYC65091.1    ...........
QYC65057.1    ...........
QWF08850.1    ...........
QYC65056.1    ...........
QWF08849.1    ...........
QWF08969.1    ...........
QWF08848.1    ...........
QWF08968.1    ...........
QWF08965.1    ...........
QWF08956.1    ...........
QWF08952.1    ...........
QWF08903.1    ...........
QWF08902.1    ...........
QWF08895.1    ...........
QWF08888.1    ...........
QWF08893.1    ...........
QWF08892.1    ...........
QWF08876.1    ...........
QWF08868.1    ...........
QWF08862.1    ...........
QWF08861.1    ...........
QWF08860.1    ...........
QWF08859.1    ...........
QWF08858.1    ...........
QWF08854.1    ...........
QWF08929.1    ...........
QYC65137.1    ...........
QYC65100.1    ...........
QWY15926.1    ...........
QWF08962.1    ...........
QWF08909.1    ...........
QWY15854.1    ...........
QWY15808.1    ...........
QWF08770.1    ...........
QWY15728.1    ...........
QYC65126.1    ...........
QWF08827.1    ...........
QYC65072.1    ...........
QYC65067.1    ...........
QWY15767.1    ...........
QWY15735.1    ...........
BCB92153.1    ...........
QWF08966.1    ...........
QIH53917.1    ...........
QYC65092.1    ...........
QWY15879.1    ...........
QWY15777.1    ...........
BCR43105.1    ...........
QWF08826.1    ...........
BCG67024.1    ...........
BCG66999.1    ...........
QWY15752.1    ...........
BCG67028.1    ...........
BCG67027.1    ...........
BCG66996.1    ...........
BCG67026.1    ...........
BCG67025.1    ...........
BCG67023.1    ...........
BCG67022.1    ...........
BCG67021.1    ...........
BCG67013.1    ...........
BCG67000.1    ...........
BCG66997.1    ...........
BCG66998.1    ...........
QWF08924.1    ...........
QIL87522.1    ...........
QYC65075.1    ...........
QWF08781.1    ...........
QWY15817.1    ...........
QWF08897.1    ...........
QWY15815.1    ...........
QWY15814.1    ...........
QWY15718.1    ...........
QYC65077.1    ...........
QTA73897.1    ...........
QWY15676.1    ...........
QOP74939.1    ...........
QOP74936.1    ...........
QWF08729.1    ...........
QYC65064.1    ...........
QWF08775.1    ...........
QWF08774.1    ...........
QWF08753.1    ...........
QWY15804.1    ...........
QOP74943.1    ...........
QIH53920.1    ...........
QIH53919.1    ...........
QIL87536.1    ...........
UEC49854.1    ...........
UEC49852.1    ...........
QWY15841.1    ...........
QWY15787.1    ...........
QYC65128.1    ...........
QYC65116.1    ...........
QYC65083.1    ...........
QYC65029.1    ...........
QWF08951.1    ...........
QWF08937.1    ...........
QWF08918.1    ...........
QWF08901.1    ...........
QWF08834.1    ...........
QWF08832.1    ...........
QWF08802.1    ...........
QWF08800.1    ...........
QYC65123.1    ...........
QWY15820.1    ...........
QWY15864.1    ...........
QWY15840.1    ...........
QWY15786.1    ...........
QWY15842.1    ...........
QWY15704.1    ...........
QWY15750.1    ...........
QWF08733.1    ...........
QNL13228.1    ...........
QNL13216.1    ...........
QNL13226.1    ...........
QNL13203.1    ...........
```

QYC65080.1	....
QNL13225.1	....
QNL13224.1	....
QNL13223.1	....
QNL13222.1	....
QNL13221.1	....
QNL13220.1	....
QNL13219.1	....
QWF08894.1	....
QNL13218.1	....
QNL13217.1	....
QNL13215.1	....
QNL13214.1	....
QNL13213.1	....
QNL13212.1	....
QNL13209.1	....
QIL87537.1	....
QNL13208.1	....
QNL13207.1	....
QNL13206.1	....
QNL13202.1	....
QNL13196.1	....
QNL13195.1	....
QNL13194.1	....
QNL13193.1	....
QNL13192.1	....
QNL13191.1	....
QNL13190.1	....
QNL13189.1	....
QNL13188.1	....
QCY53846.1	....
BCI65357.1	....
QNL13227.1	....
QNL13211.1	....
QNL13198.1	....
QNL13197.1	....
QNL13200.1	....
QNL13210.1	....
QNL13205.1	....
QNL13204.1	....
QNL13201.1	....
QNL13199.1	....
QYC65157.1	....
QYC65138.1	....
QWF08724.1	....
UEP53403.1	....
QWY15843.1	....
QWF08794.1	....
QYC65031.1	....
UEC49856.1	....
BBH72184.1	....
QWF08874.1	....
QWF08873.1	....
BCG67003.1	....
QYC65095.1	....
QOP74933.1	....
QYC65096.1	....
BCG67034.1	....
BCG67031.1	....
QYC65052.1	....
QWF08963.1	....
QWF08939.1	....
QIL87538.1	....
QIL87535.1	....
QWF08964.1	....
QYC65132.1	....
QTA73893.1	....
QWF08751.1	....
QWY15860.1	....
QWY15749.1	....
QWY15744.1	....
QWY15740.1	....
QWY15700.1	....
QWY15698.1	....
QWY15672.1	....
pdb|6LHT|B	....
pdb|6LHT|E	....
pdb|6LHT|D	....
pdb|6LHT|C	....
pdb|6LHT|A	....
pdb|6LHQ|A	....
pdb|6LHP|A	....
pdb|6LHO|A	....
pdb|6LHL|A	....
pdb|6LHK|A	....
pdb|6LHC|A	....
pdb|6LHB|A	....
pdb|6LHA|A	....
QWY15708.1	....
BBH72101.1	....
BBH72090.1	....
BBH72097.1	....
BBH72089.1	....
BBH72099.1	....
BBH72093.1	....
BBH72096.1	....
BBH72094.1	....
BBH72091.1	....
QOP74916.1	....
QYC65163.1	....
QWF08736.1	....
QWF08735.1	....
pdb|5TSK|A	....
pdb|5C9A|A	....
pdb|5C4W|A	....
pdb|4JGZ|A	....
pdb|4JGY|A	....
QWY15956.1	....
QWF08811.1	....
QWF08878.1	....
QWF08869.1	....
QWF08831.1	....
QWF08829.1	....
QWF08824.1	....
QWF08822.1	....
QWF08817.1	....
QWF08816.1	....
QWF08798.1	....
QWF08779.1	....
QWF08778.1	....
QWF08772.1	....
QWF08771.1	....
QWF08745.1	....
QWF08744.1	....
QIH53922.1	....
QOP74941.1	....
QWY15812.1	....
QOP74926.1	....
QYC65176.1	....
QYC65172.1	....
QYC65168.1	....
QWF08725.1	....
QYC65028.1	....
QWY15686.1	....
QYC65023.1	....
QYC65025.1	....
QWY15946.1	....
BCB92164.1	....
QOP74935.1	....
QOP74930.1	....
QOP74928.1	....
QOP74927.1	....
QOP74925.1	....
QOP74923.1	....

```
QOP74922.1    ...........
QOP74920.1    ...........
QOP74919.1    ...........
QOP74917.1    ...........
QHB49260.1    ...........
QWF08784.1    ...........
QWF08782.1    ...........
QWF08777.1    ...........
QWF08757.1    ...........
QWF08756.1    ...........
QWF08755.1    ...........
QWF08791.1    ...........
QWF08786.1    ...........
QYC65186.1    ...........
QWY15722.1    ...........
QYC65182.1    ...........
QWF08754.1    ...........
QWF08748.1    ...........
QYC65181.1    ...........
QWY15719.1    ...........
QYC65180.1    ...........
QWY15693.1    ...........
QYC65179.1    ...........
BCB92156.1    ...........
QYC65178.1    ...........
pdb|5C8C|A    ...........
QWF08864.1    ...........
QWF08747.1    ...........
QWF08743.1    ...........
QWF08741.1    ...........
QWF08737.1    ...........
QWF08734.1    ...........
BBH72119.1    ...........
QYC65177.1    ...........
QWF08732.1    ...........
QWF08730.1    ...........
QYC65175.1    ...........
QWF08727.1    ...........
QYC65173.1    ...........
BBH72105.1    ...........
QYC65171.1    ...........
pdb|5ABJ|A    ...........
QWY15716.1    ...........
QWY15800.1    ...........
QYC65018.1    ...........
QWY15712.1    ...........
QWY15711.1    ...........
QWY15707.1    ...........
QWY15688.1    ...........
QWY15685.1    ...........
QWY15684.1    ...........
QYC65170.1    ...........
QYC65169.1    ...........
QYC65162.1    ...........
QYC65099.1    ...........
QYC65048.1    ...........
QYC65040.1    ...........
QYC65035.1    ...........
QYC65032.1    ...........
QYC65030.1    ...........
QYC65027.1    ...........
QYC65026.1    ...........
QYC65024.1    ...........
QYC65022.1    ...........
QYC65021.1    ...........
QYC65020.1    ...........
QYC65019.1    ...........
QWF08742.1    ...........
QWF08728.1    ...........
QWF08726.1    ...........
QYC65054.1    ...........
QWF08746.1    ...........
QYC65033.1    ...........
QWY15952.1    ...........
QYC65042.1    ...........
QYC65017.1    ...........
QOP74918.1    ...........
QWF08731.1    ...........
BBH72123.1    ...........
BBH72114.1    ...........
BBH72092.1    ...........
BBH72095.1    ...........
BBH72186.1    ...........
QWY15975.1    ...........
QWY15974.1    ...........
QWY15968.1    ...........
QWY15960.1    ...........
QWY15959.1    ...........
QWY15953.1    ...........
QWY15951.1    ...........
QWY15950.1    ...........
QWY15949.1    ...........
QWY15944.1    ...........
QWY15943.1    ...........
QWY15942.1    ...........
QWY15939.1    ...........
QWY15938.1    ...........
QWY15937.1    ...........
QWY15936.1    ...........
QWY15934.1    ...........
QWY15896.1    ...........
BCU41098.1    ...........
BCR43090.1    ...........
QYC65129.1    ...........
QWF08961.1    ...........
QIL87527.1    ...........
QWY15955.1    ...........
QWY15954.1    ...........
QWY15945.1    ...........
QWY15935.1    ...........
BCB92163.1    ...........
BCB92158.1    ...........
BCB92154.1    ...........
BCB92152.1    ...........
BCB92162.1    ...........
BCB92161.1    ...........
BCB92160.1    ...........
BBH72129.1    ...........
QIL87533.1    ...........
BBH72150.1    ...........
BBH72137.1    ...........
BBH72145.1    ...........
BCB92157.1    ...........
QYC65124.1    ...........
QWY15920.1    ...........
QWY15973.1    ...........
BCU41116.1    ...........
QWF08960.1    ...........
QWF08958.1    ...........
QWF08919.1    ...........
QWY15825.1    ...........
QWF08953.1    ...........
QWF08948.1    ...........
QWY15884.1    ...........
QWF08946.1    ...........
QWF08941.1    ...........
QWF08914.1    ...........
BCU41097.1    ...........
QWY15918.1    ...........
BCR43086.1    ...........
QWF08935.1    ...........
QWY15912.1    ...........
QWY15911.1    ...........
QWY15887.1    ...........
```

```
QWY15882.1     ...........
BCG67018.1     ...........
QWY15881.1     ...........
QWY15875.1     ...........
BCG67016.1     ...........
QWY15874.1     ...........
QWY15853.1     ...........
BCG67014.1     ...........
BCG67009.1     ...........
QWF08933.1     ...........
BCG66992.1     ...........
BCG66991.1     ...........
QWF08898.1     ...........
QWF08930.1     ...........
QWF08925.1     ...........
QWF08913.1     ...........
QWF08911.1     ...........
BBH72117.1     ...........
BBH72116.1     ...........
BBH72113.1     ...........
BBH72112.1     ...........
BBH72108.1     ...........
BBH72106.1     ...........
BBH72104.1     ...........
BBH72211.1     ...........
BBH72210.1     ...........
BBH72209.1     ...........
BBH72208.1     ...........
BBH72207.1     ...........
BBH72205.1     ...........
BBH72201.1     ...........
BBH72193.1     ...........
BBH72192.1     ...........
BBH72190.1     ...........
BBH72189.1     ...........
BBH72188.1     ...........
BBH72187.1     ...........
BBH72185.1     ...........
BBH72182.1     ...........
BBH72181.1     ...........
BBH72179.1     ...........
BBH72178.1     ...........
BBH72177.1     ...........
BBH72175.1     ...........
BBH72174.1     ...........
BBH72172.1     ...........
BBH72170.1     ...........
BBH72169.1     ...........
BBH72168.1     ...........
BBH72158.1     ...........
BBH72155.1     ...........
BBH72154.1     ...........
BBH72151.1     ...........
BBH72148.1     ...........
BBH72144.1     ...........
BBH72142.1     ...........
BBH72141.1     ...........
BBH72140.1     ...........
BBH72132.1     ...........
BBH72131.1     ...........
BBH72130.1     ...........
BBH72128.1     ...........
BDC16283.1     ...........
BDC16271.1     ...........
BDC16270.1     ...........
BDC16258.1     ...........
QIL87526.1     ...........
BBH72180.1     ...........
BBH72109.1     ...........
BBH72115.1     ...........
BBH72152.1     ...........
BBH72200.1     ...........
QWY15844.1     ...........
BBH72146.1     ...........
BBH72173.1     ...........
QWY15947.1     ...........
BDC16281.1     ...........
BBH72167.1     ...........
BBH72160.1     ...........
BCU41096.1     ...........
BCR43102.1     ...........
QWF08881.1     ...........
QWF08855.1     ...........
BBH72195.1     ...........
BBH72118.1     ...........
BBH72126.1     ...........
BBH72127.1     ...........
BBH72206.1     ...........
BBH72196.1     ...........
BBH72171.1     ...........
BBH72191.1     ...........
BBH72133.1     ...........
BBH72139.1     ...........
BBH72166.1     ...........
BBH72161.1     ...........
BBH72165.1     ...........
BBH72164.1     ...........
BBH72159.1     ...........
BBH72157.1     ...........
BBH72162.1     ...........
BBH72153.1     ...........
BBH72156.1     ...........
BBH72120.1     ...........
BBH72198.1     ...........
BBH72197.1     ...........
QWY15941.1     ...........
BBH72121.1     ...........
BBH72135.1     ...........
QWY15967.1     ...........
QWY15964.1     ...........
QWY15969.1     ...........
QWY15913.1     ...........
QWY15908.1     ...........
QWY15907.1     ...........
QWY15890.1     ...........
QWY15889.1     ...........
QWY15888.1     ...........
QWY15886.1     ...........
QWY15885.1     ...........
QWY15883.1     ...........
QWY15878.1     ...........
QWY15876.1     ...........
BBH72176.1     ...........
QIL87519.1     ...........
QWY15906.1     ...........
QWY15917.1     ...........
QWY15846.1     ...........
QWY15901.1     ...........
QWY15857.1     ...........
QWY15904.1     ...........
BBH72204.1     ...........
BBH72203.1     ...........
BBH72202.1     ...........
BDC16266.1     ...........
BCR43091.1     ...........
QWY15902.1     ...........
BBH72226.1     ...........
BBH72222.1     ...........
BBH72012.1     YEGGV......
BBH71965.1     YEGGV......
BBH71934.1     YEGG.......
BBH72009.1     NEGG.......
QWY16175.1     ...........
QWY16167.1     ...........
```

```
QWY16174.1    ...........
QWY16171.1    ...........
QWY16173.1    ...........
QWY16172.1    ...........
QWY16170.1    ...........
QWY16169.1    ...........
QWY16168.1    ...........
QXF31588.1    ...........
QXF31587.1    ...........
pdb|6IIO|A    M..........
pdb|6IIJ|A    M..........
pdb|6AKS|A    ...........
BCD33944.1    AFG........
pdb|7ECY|A    VKTMPHNIVNT
pdb|7EC5|A    VKTMPHNIVNT
pdb|7EBZ|A    VKTMPHNIVNT
pdb|7EBR|A    VKTMPHNIVNT
pdb|6MZI|A    VKTMPHNIVNT
pdb|6CSH|A    VKTMPHNIVNT
pdb|6CSG|A    VKTMPHNIVNT
pdb|6CSA|A    VKTMPHNIVNT
pdb|6CS6|A    VKTMPHNIVNT
pdb|6CS5|A    VKTMPHNIVNT
pdb|6CS4|A    VKTMPHNIVNT
pdb|6CS3|A    VKTMPHNIVNT
pdb|6CRU|A    VKTMPHNIVNT
pdb|6CRS|A    VKTMPHNIVNT
pdb|6CRR|A    VKTMPHNIVNT
pdb|6CRP|A    VKTMPHNIVNT
QNO39042.1    VKTMPHNIVTT
QNO39041.1    VKTMPHNIVTT
QNO39040.1    VKTMPHNIVTT
QNO39038.1    VKTMPHNIVTT
QNO39039.1    VKTMPHNIVTT
CAA1539354.1  VKTMPHNIVTT
CAA1527138.1  VKTMPHNIVTT
CAA1539353.1  VKTMPHNIVTT
CAA1527136.1  VKTMPHNIVTT
QNO39037.1    VKTMPHNIVTT
QKX95883.1    ...........
pdb|5BNP|A    VTTMPHNIVTT
pdb|5BNO|A    VTTMPHNIVTT
pdb|5BNN|A    VTTMPHNIVTT
pdb|4WM8|A    VTTMPHNIVTT
pdb|4WM7|A    VTTMPHNIVTT
QIC50353.1    ...........
QIC50352.1    ...........
QIC50356.1    ...........
```
