# Supplementary material for: Conserved Residues Adjacent to ß-Barrel and Loop Intersection among Enterovirus VP1 Affect Viral Replication: Potential Target for Anti-Enteroviral Development
Source: Viruses. 2022 Feb 10;14(2):364. doi: 10.3390/v14020364 (PMC8877150; doi:10.3390/v14020364)
Supplement: Supplementary file 1 [file viruses-14-00364-s001.zip › supplementary Table S1.pdf]

Table S1. Overlapping extension PCR primer sequences for alanine substitution.

	Primer Name	Sequence (5′ -> 3′)
VP1-R86A	AG2696GC-F	GATAGCTTCTTCAGCGCAGCAGGATTAG
	AG2696GC-R	CTAATCCTGCTGCGCTGAAGAAGCTATC
VP1-E134A	A2841C-F	CGTTTTGACGCAGCGTTCACCTTT
	A2841C-R	AAAGGTGAACGCTGCGTCAAAACG
VP1-P157A	C2909G-F	CATGTTTGTAGCACCCGGAGCCC
	C2909G-R	GGGCTCCGGGTGCTACAAACATG
VP1-P193A	C3017G-F	GCACAGGTTTCTGTTGCATTCATGTCAC
	C3017G-R	GTGACATGAATGCAACAGAAACCTGTGC
VP1-G231A	G3132C-F	CAAACAACATGATGGCTACGTTCTCAGTG
	G3132C-R	CACTGAGAACGTAGCCATCATGTTGTTTG
VP1-K256A	AA3206GC-F	CATGAGGATGGCGCACGTCAGG
	AA3206GC-R	CCTGACGTGCGCCATCCTCATG
